# Borane catalysed annulative sulfenylation of internal alkynes: towards the synthesis and study of fused heterocycles

**DOI:** 10.1039/d6sc01123c

**Published:** 2026-04-29

**Authors:** Sampurna Das, Milan Pramanik, Johannes Westphäling, Abhishek Kumar Gupta, Thomas Wirth, Niklaas J. Buurma, Eli Zysman-Colman, Mu-Hyun Baik, Rebecca L. Melen

**Affiliations:** a Cardiff Catalysis Institute, School of Chemistry, Cardiff University, Translational Research Hub Maindy Road, Cathays Cardiff CF24 4HQ Cymru/Wales UK MelenR@cardiff.ac.uk; b Department of Chemistry, Korea Advanced Institute of Science and Technology (KAIST) Daejeon 34141 Republic of Korea mbaik2805@kaist.ac.kr; c Center for Catalytic Hydrocarbon Functionalizations, Institute for Basic Science (IBS) Daejeon 34141 Republic of Korea; d Organic Semiconductor Centre, EaStCHEM School of Chemistry, University of St Andrews North Haugh, St. Andrews KY16 9ST Scotland UK; e School of Chemistry, Cardiff University Park Place, Main Building Cardiff CF10 3AT Cymru/Wales UK

## Abstract

Herein, we disclose a B(C_6_F_5_)_3_-catalysed intramolecular cyclisation reaction of *N*-protected alkynyl anilines and phenols to generate 5-membered heterocycles, including 3-sulfenyl indoles (17 examples, up to 91% yield) and benzo[*b*]furans (9 examples, up to 90% yield), in good yields with several functional group tolerances. This protocol was adapted into an annulative π-extension (APEX) reaction when using diyne derivatives of aniline and phenol, which effectively led to sulfenylated polyaromatic heterocycles, such as benzo[*a*]carbazole or naphtho[1,2-*b*]benzofurans. These products exhibit fluorescence from locally excited states, consistent with their large singlet-triplet energy gaps. Additionally, the cyclisation of aryl propargyl ethers, 4-diphenylbut-1-yne and a tosyl-protected propargylaniline afforded sulfenylated 6-membered products (7 examples, up to 94% yield). Density functional theory (DFT) calculations, corroborated by initial kinetics, helped to understand the order and rate of the reaction and support a mechanism in which thiirenium ions are involved as key intermediates in the formation of the observed products.

## Abstract 2

This work presents an amenable and efficient B(C_6_F_5_)_3_-catalysed route for the construction of sulfur-containing heterocycles *via* thiirenium-ion intermediates.

## Graphical abstract

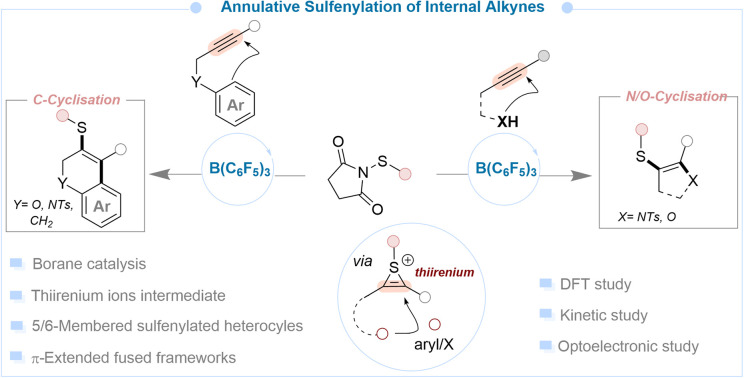

## Introduction

**Fig. 1:**
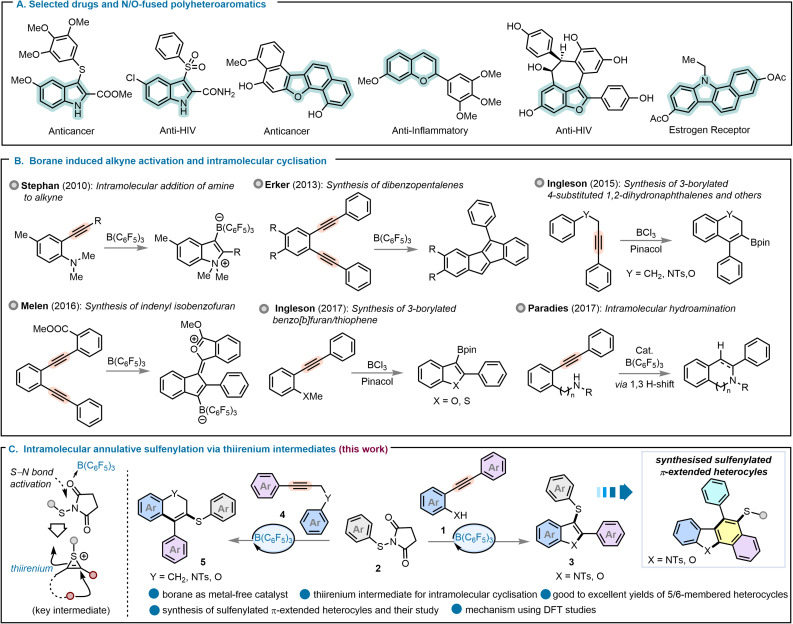
(A) Selected drugs and N/O-fused polyheterocycles. (B) Overview of borane-induced alkyne activation and intramolecular cyclisation reactions. (C) This work: intramolecular annulative sulfenylation *via* thiirenium-ion intermediates.

Heterocyclic structures are central to modern chemical science owing to their structural diversity and biological relevance.^1^ Among them indoles and benzofurans constitute a distinctive and synthetically valuable class of heterocycles in medicinal chemistry and materials science.^2–6^ Sulfenylindoles, in particular, are frequently encountered in numerous biologically active compounds, including agents with anti-cancer and anti-acquired immune deficiency syndrome (*e.g.* AIDS) properties. Partially unsaturated fused six-membered rings, exemplified by 2*H*-chromenes and 1,2-dihydronaphthalenes are likewise widespread in bioactive molecules, photochromic materials, and biopolymers (see Fig. 1A for examples).^7–10^ Accordingly, the development of general, efficient, and reliable synthetic methods for accessing these privileged frameworks remains a central pursuit in contemporary organic synthesis.

While significant developments have been made in the synthesis of indoles, only a limited number of protocols for the synthesis of 3-sulfenylindoles *via* intramolecular cyclisation strategies have been reported to date. These methodologies predominantly rely on PdCl_2_-catalysed systems,^11^ Fe/I_2_ co catalysis,^12^ and stoichiometric hypervalent iodine reagents such as PIDA (diacetoxyiodobenzene) and PhICl_2_ (dichloroiodobenzene).^13,14^ Closely related approaches have been employed for the construction of 3-sulfenylbenzofuran frameworks, utilising PdCl_2_/I_2_,^15^ ligand-chelated palladium catalysts,^16^ FeCl_3_,^17^ and I_2_/PTSA (*p*-toluenesulfonic acid) systems.^18^ These transformations often suffer from inherent limitations, including the use of toxic transition metals, stoichiometric halogenated reagents, and harsh reaction conditions that collectively contribute to environmental burdens through the generation of considerable waste and hazardous by-products. In contrast, only a single report has demonstrated the synthesis of 3-sulfenyl 1,2-dihydronaphthalenes *via* triflic anhydride-activated sulfoxides.^19^ Nevertheless, there remains a conspicuous gap in the literature concerning the development of analogous strategies for accessing 3-sulfenyl 2*H*-chromenes and 3-sulfenyldihydroquinolines. These underscore unaddressed problems for seeking environmentally benign, metal-free, and atom-economic protocols that can expand the chemical space of 5/6-membered and highly unsaturated sulfenylated heterocycles in a unified reaction sequence.

The synthesis of fused aromatic and heterocyclic systems can be efficiently achieved through the annulative π-extension (APEX) of functionalised alkynes.^20^ Consequently, the strategic installation of alkynyl units with preorganised functional groups has emerged as a state-of-the-art practice for the synthesis of structurally diverse cyclic scaffolds. Alkyne activation and intramolecular cyclisation reactions have been explored by transition metal catalysts, iodine reagents and Lewis acid catalysis as modern, green alternatives.^21–23^ More recently, borane catalysis has seen extensive use in alkyne activation through boron-centred Lewis adducts.^24^ Pioneering contributions of Stephan,^24^ Erker,^25^ and Ingleson,^27,28^ highlighted in Fig. 1B, have remarkably advanced this domain. Stephan *et al.* demonstrated that B(C_6_F_5_)_3_ can promote cyclisation *via* a frustrated Lewis pair (FLP), a strategy later used by Erker to synthesise polycyclic aromatic hydrocarbons (PAHs).^25,26^ Our group has also employed FLP-mediated domino reactions to construct π-conjugated heterocycles.^27^

In parallel, Ingleson utilised BCl_3_ in cascade reactions to synthesise various 3-borylated products.^28,29^ A major drawback of these methods is their reliance on stoichiometric reagents, which limits their application. While a more recent report by Paradies and co-workers on a catalytic hydroamination shows promise,^30^ the development of a general, catalytic approach for borane-mediated cascade cyclisations remains a significant goal.

In this work, we describe a B(C_6_F_5_)_3_-catalysed protocol for the synthesis of 5- and 6-membered sulfenylated heterocycles. Thiiranium ion intermediates have been studied for the stereoselective and regioselective activation of functionalised alkenes,^31,32^ and we have previously established that thiiranium ion intermediates play a key role in facilitating regiodivergent sulfenylation reactions.^33^ We envisioned that APEX reactions *via* a similar thiirenium ion intermediate (derived from an alkyne rather than alkene) and subsequent intramolecular cyclisation could provide an efficient route to diverse medium-sized heterocycles under mild conditions. We hypothesised that the B(C_6_F_5_)_3_-catalysed cyclisation of 2-alkynylanilines or phenols with *N*-(arylthio) succinimides would afford 3-sulfenylated indoles or furans incorporating a privileged heterocyclic scaffold alongside sulfur-derived redox activity and enhanced lipophilicity. This strategy could then be subsequently extended from the synthesis of five-membered heterocycles to six-membered analogues, enabling access to 3-sulfenylated 2*H*-chromenes, dihydroquinolines, and dihydronaphthalenes.

## Results and discussion

**Fig. 2:**
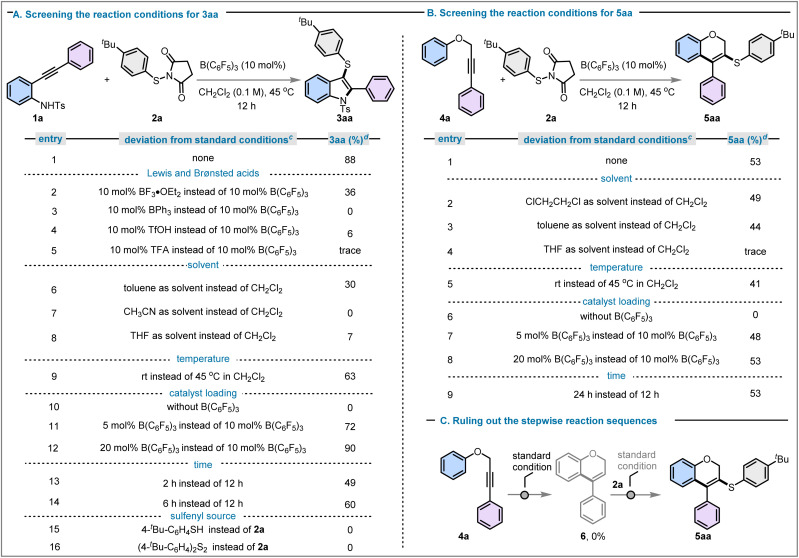
(A) Optimisation of the reaction conditions for the synthesis of 3aa. (B) Optimisation of the reaction conditions for the synthesis of 5aa. (C) Control experiments indicate that a stepwise reaction is unfavourable. ^*c*^Standard conditions: 1a/4a (0.1 mmol, 1.0 equiv.), 2a (0.1 mmol, 1.0 equiv.) in 0.1 M CH_2_Cl_2_ solvent at 45 °C for 12 h; ^*d*^Isolated yields.

To initiate this study, we selected *N*-tosyl-2-alkynylaniline (1a) as a model substrate and investigated its reaction with *N*-(*p-tert*-butylthio)succinimide (2a) as the sulfenylating agent under B(C_6_F_5_)_3_ catalysis. This transformation was chosen to probe the feasibility of a tandem sulfenylation-cyclisation process leading to 3-sulfenylated indoles. A systematic evaluation of the reaction parameters, including catalyst loading, solvent, temperature, and reaction time, was therefore undertaken, and the results of this optimisation study are summarised in Fig. 2. The 3-sulfenylindole product 3aa was obtained in 88% yield upon treatment with 10 mol% B(C_6_F_5_)_3_ in CH_2_Cl_2_ (0.1 M) at 45 °C over 12 hours (Fig. 2A, entry 1). We subsequently investigated the influence of Lewis and Brønsted acids, solvents, temperature, catalyst loading, time and sulfenylating agent on the yield of the desired product. Substituting B(C_6_F_5_)_3_ with other boron Lewis acids, such as trifluoroboron etherate (BF_3_·OEt_2_) and triphenylborane (BPh_3_), led to reduced/no reactivity under otherwise unchanged reaction conditions (Fig. 2A, entries 2 and 3). To evaluate the potential of Brønsted acid catalysis for the synthesis of 3aa, triflic acid (TfOH) and trifluoroacetic acid (TFA) were trialed, however both resulted in negligible or no yield (see Fig. 2A, entries 4 and 5). These results highlight the necessity of a strong Lewis acid for the efficient formation of 3-sulfenylindole 3aa. To evaluate solvent effects, a range of solvents were screened for this transformation. None of the solvents examined outperformed methylene chloride, with obtained yields of 3aa of 30% in toluene, 0% in acetonitrile and 7% in THF (Fig. 2A, entries 6–8). Conducting the reaction at room temperature resulted in a yield of 63%, clearly indicating that slightly elevated temperatures are needed to obtain satisfactory conversions and yields of 3aa (Fig. 2A, entry 9). No product formation was observed in the absence of a catalyst (Fig. 2A, entry 10), and catalyst loadings of 5 mol% and 20 mol% resulted in 72% and 90% yield, respectively (Fig. 2A, entries 11 and 12). Thus, catalyst loadings above 10 mol% did not result in significantly higher yields of 3aa (compare Fig. 2A, entry 1), whereas 5 mol% catalyst loading resulted in a noticeable drop in yield of the desired product. A reaction time of 12 h was found to be necessary to reach high yields, as reducing the reaction time to 2 h or 6 h resulted in only 49% and 60% yield of 3aa, respectively (Fig. 2A, entries 13 and 14). Other sulfenylating agents, such as 4-*tert*-butyl benzenethiol and the corresponding disulfide, were also employed, yet neither produced the desired product (Fig. 2A, entries 15 and 16).

We then applied the optimal reaction conditions to activate other internal alkynes, specifically the propargyl ether 4a (Fig. 2B). Using the conditions previously optimised for the synthesis of 3aa above, this afforded the six-membered sulfenylated 2*H*-chromene 5aa in 53% yield (Fig. 2B, entry 1).

This prompted us to optimise the reaction conditions towards the formation of the six-membered sulfenylated 2*H*-chromene product 5aa. Among the solvents examined, 1,2-dichloroethane and toluene gave 49% and 44% yield of 5aa, respectively, whereas THF was again found to be ineffective for this transformation (Fig. 2B, entries 2–4). Reaction at ambient temperature produced 5aa in 41% yield (Fig. 2B, entry 5). As expected, the reaction did not proceed without a borane catalyst (Fig. 2B, entry 6), and neither a decrease (5 mol%) nor an increase (20 mol%) of catalyst loading could provide better yields (48% and 53%, respectively) than the standard loading of 10 mol% B(C_6_F_5_)_3_ (Fig. 2B, entries 7 and 8). The extension of reaction time beyond 12 h did not show enhancement in product yield (Fig. 2B, entry 9). As none of these modifications led to an increased yield of 5aa, the reaction conditions previously identified as optimal for the formation of 3aa were also adopted for this class of products.

We subsequently sought to elucidate whether the reaction proceeds *via* a stepwise pathway (comprising initial heterocycle formation followed by thiol incorporation), or through a concerted process involving simultaneous cyclisation and thiol trapping. The employment of propargyl ether 4a (Fig. 2C) with 10 mol% B(C_6_F_5_)_3_ did not lead to the anticipated intermediate 2*H*-chromene 6. Thus, we concluded that the reaction did not proceed through the initial formation of a 2*H*-chromene.

**Fig. 3:**
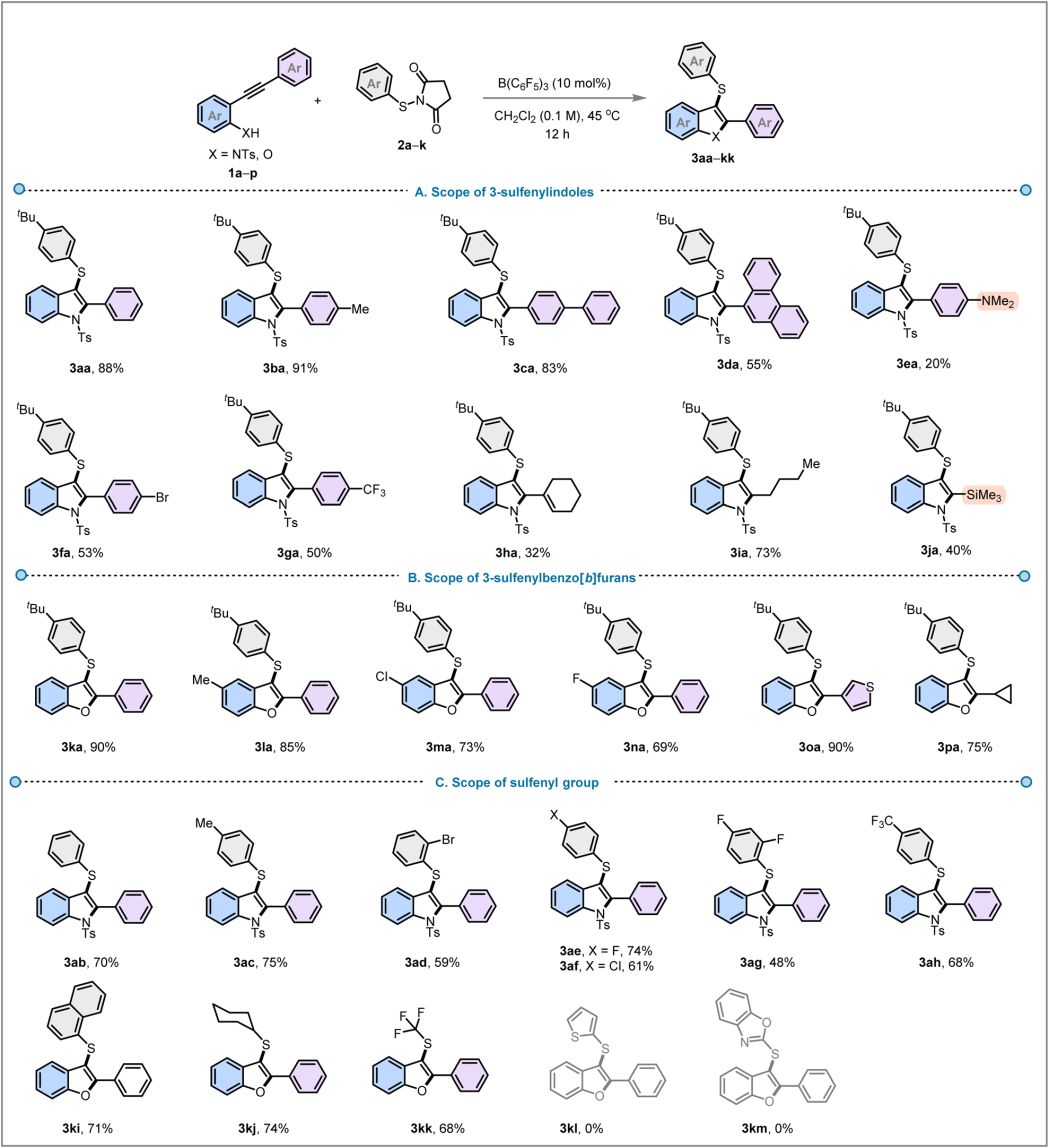
(A) Reaction scope for the formation of 3-sulfenyl indoles using *N*-(*p-tert*-butylthio)succinimide (2a). (B) Reaction scope for the formation of 3-sulfenyl benzo[*b*]furans using *N*-(*p-tert*-butylthio)succinimide (2a). (C) Reaction scope for the synthesis of 3-sulfenyl indoles and 3-sulfenyl benzo[*b*]furans using various thiosuccinimides 2.

With the optimised reaction conditions for both substrate classes in hand, we evaluated the scope of *N*-tosyl-2-alkynylanilines 1 in combination with *N*-(*p-tert*-butylthio)succinimide (2a) (Fig. 3A). *N*-tosyl-2-alkynylanilines (1a–d) with different aryl groups such as tolyl, biphenyl, phenanthrene, reacted efficiently under the standard reaction conditions to provide 3-sulfenylindoles (3aa–3da) in 55–91% yield. Electron-rich *N*-tosyl-2-alkynylanilines bearing an *N*,*N*-dimethylaniline (1e) group produced lower yields (3ea, 20%), likely due to competing N–B coordination between B(C_6_F_5_)_3_ and the NMe_2_ group, which deactivates both the B(C_6_F_5_)_3_ catalyst and the alkyne. Electron-withdrawing substituents, such as –Br and –CF_3_, on the starting material (1f and 1g, respectively) also led to moderate yields of 53% (3fa) and 50% (3ga), respectively. This is likely because these groups render the alkyne more electron-deficient, hampering the formation of the thiirenium ion intermediates (*vide infra*). Alkyl substituted alkynylanilines (1h and 1i) were likewise tolerated under the optimised reaction conditions, generating indoles 3ha and 3ia in 32% and 73% yield. The trimethylsilyl (TMS) substituted indole 3ja was formed in 40% yield, offering potential for derivatisation by manipulation of the TMS group. In 2-trimethylsilyl-3-sulfenyl indoles, this dual substitution pattern enables orthogonal reactivity: while the sulfenyl group at C-3 can undergo oxidation or substitution, the TMS group allows for diversification at C-2 *via* halogenation, nucleophilic trapping or cross coupling.^34^ Next, we turned our attention towards the implementation of our reaction strategy towards the synthesis of 3-sulfenylbenzofurans from 2-alkynylphenols (1) with *N*-(*p-tert*-butylthio)succinimide (2a) (Fig. 3B). Alkynyl phenols 1 substituted with –H, –Me, –Cl groups produced the corresponding 3-sulfenyl benzo[*b*]furans (3ka–3na) with 69–90% yields. 2-Alkynylphenols with thiophene and cyclopropane moieties also smoothly reacted with the thiosuccinimide 2a to yield unsaturated heterocycles 3oa and 3pa in 90% and 75% yield, respectively.

The substrate scope of the thiosuccinimide derivatives (2) was then examined for both with *N*-tosyl-2-alkynylanilines and 2-alkynylphenols (1, X = NTs and O, respectively) (Fig. 3C). Unsubstituted and substituted sulfenylating agents (2b–h) bearing *p*-Me, *o*-Br, *p*-Cl, *p*-F, *o*/*p*-F, *p*-CF_3_ substituted phenyl groups delivered corresponding products 3ab–3ah efficiently in 48–75% yield. Thiosuccinimides with polyaromatic naphthyl (2i) or aliphatic cyclohexyl (2j) substituents also reacted effectively under this annulative C–S coupling strategy to provide products 3ki and 3kj in 71% and 74% yield, respectively. Interestingly, the 3-trifluoromethylthiolated benzofuran 3kk was also synthesised from 2-alkynylphenol and trifluoromethyl substituted thiosuccinimide 2k in 68% yield. The incorporation of the SCF_3_ moiety into the benzofuran core is valuable as it can enhance pharmacokinetic profiles due to its strong electron-withdrawing and lipophilic nature, potentially translating into improved membrane permeability, metabolic stability, and biological activity against diverse targets.^35^ Thiophene and benzoxazole substituted thiosuccinimides 2l and 2m on the other hand showed no reactivity.

**Fig. 4:**
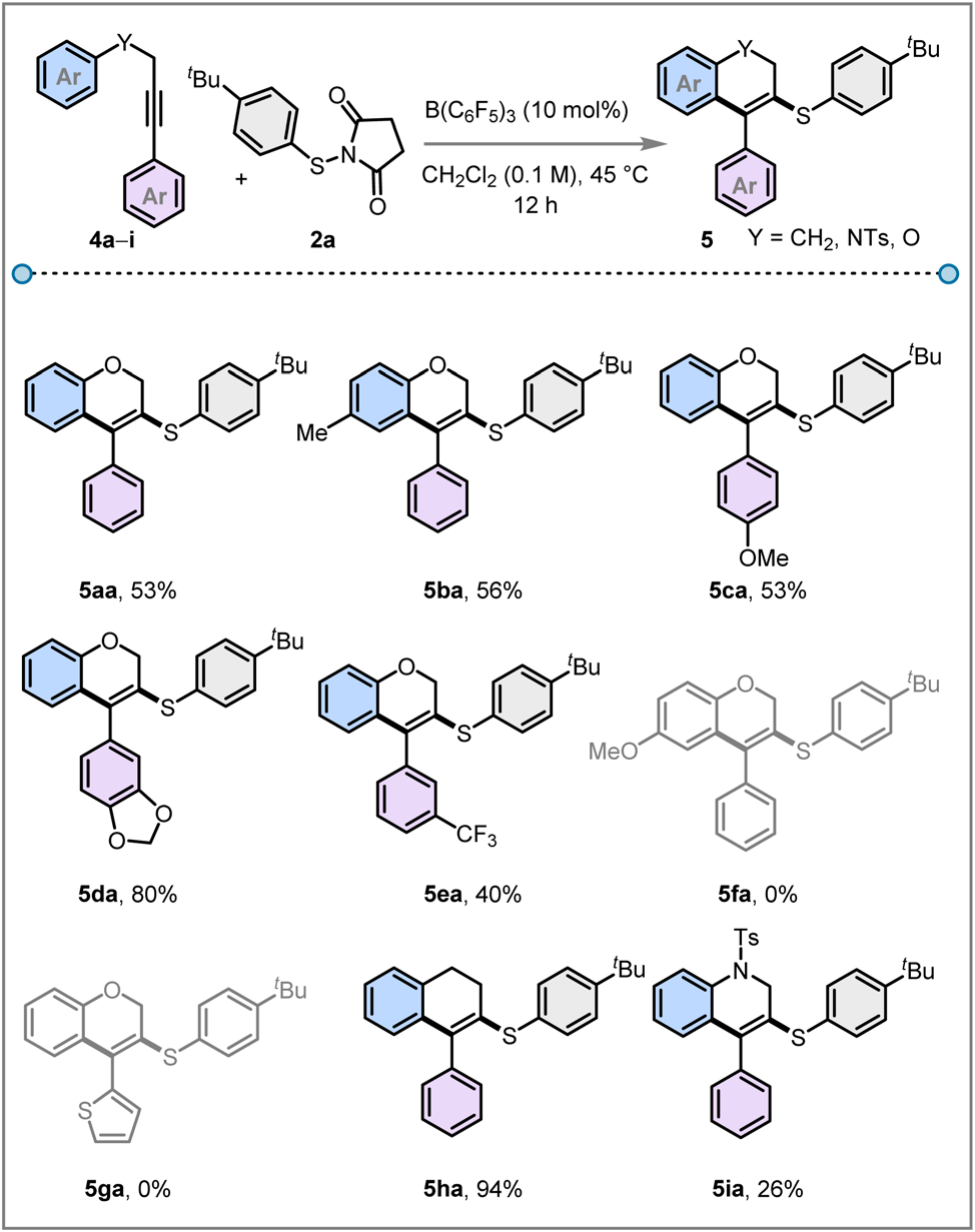
Scope of 3-sulfenyl 2*H*-chromenes and related compounds (5).

Finally, we explored the intramolecular cyclisation strategy towards the construction of six-membered sulfenylated 2*H*-chromene products 5 from aryl propargyl ethers 4 with thiosuccinimide 2a (Fig. 4). Various substituted propargyl ethers (4a–4e) underwent the annulative sulfenylation reaction, resulting in the formation of six-membered 3-sulfenyl 2*H*-chromenes (5aa–5ea) in 40–80% yields. Notably, the presence of a *p*-OMe substituent on the phenyl ether of the alkyne (4f) did not lead to the desired cyclised product (5fa). Also, the thiophene-substituted propargyl ether (4g) failed to react under the standard reaction conditions. This methodology was then further extended to synthesising 3-sulfenyl dihydronaphthalenes starting from 1,4-diphenylbut-1-yne 4h which afforded 5ha in 94% yield. In addition to this, the herein described intramolecular cascade cyclisation protocol was proved to be effective for the activation of a tosyl-protected amine containing alkyne 4i, enabling the synthesis of a 3-sulfenyl dihydroquinoline framework 5ia in 26% yield.

**Fig. 5:**
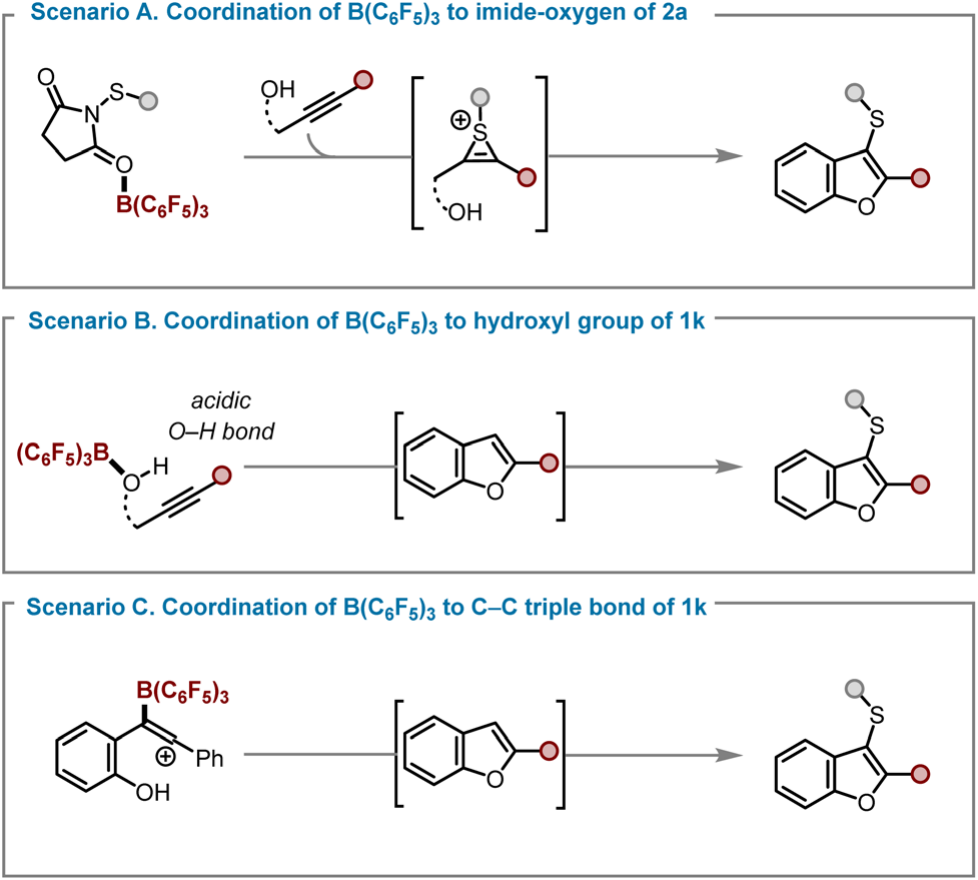
Summary of mechanistic scenarios for the formation of final products.

To gain deeper insight into the reaction mechanism, we performed extensive Density Functional Theory (DFT) calculations at the ωB97M-V/def2-TZVPD//PBEh-3c/C-PCM(DCM)^36–39^ level of theory, using alkynyl phenol 1k as the model substrate. Depending on the preferred coordination mode of B(C_6_F_5_)_3_ with the reaction components, several mechanistic pathways were considered and are summarised in Fig. 5.

**Fig. 6:**
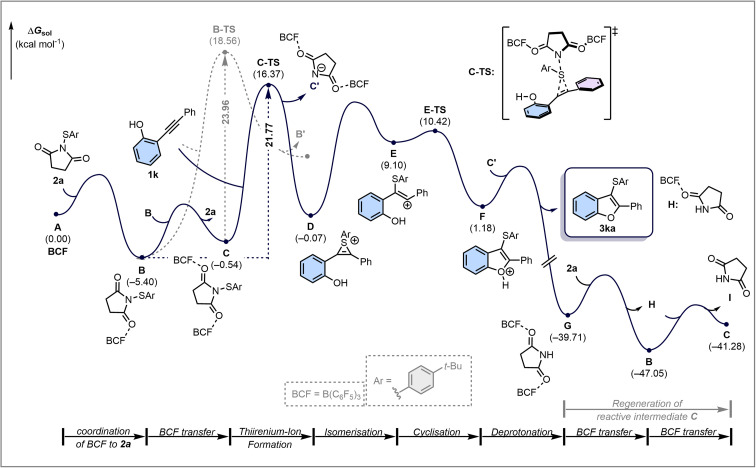
Reaction energy profile of mechanistic scenario A – B(C_6_F_5_)_3_ coordination to the imide-oxygen of thiosuccinimide 2a. Energies are Gibbs free energies at the ωB97M-V/def2-TZVPD//PBEh-3c/C-PCM(DCM) level of theory. Relative energies are given in kcal mol^−1^

In scenario A (Fig. 5), B(C_6_F_5_)_3_ coordinates to the imide-oxygen of 2a, enabling the subsequent transfer of the arylsulfenyl group to the alkynyl phenol 1k, forming a thiirenium ion intermediate that then rapidly cyclises to the observed product.^40^ Alternative possibilities include scenario B (Fig. 5), in which B(C_6_F_5_)_3_ binds to the hydroxyl group of 1k to initiate a Brønsted acid-catalysed process, and scenario C (Fig. 5), in which direct coordination to the C–C triple bond of 1k forms a benzofuran derivative that undergo subsequent sulfenylation. However, Scenario B was found to have a substantially higher activation barrier and is therefore unlikely to operate under the experimental conditions. Scenario C is energetically feasible, but in disagreement with the observed rate law of this reaction (see below and SI for a detailed discussion). Scenario A is therefore proposed to be the operative mechanism under the experimental conditions and is discussed in greater detail below. The reaction sequence, summarised in Fig. 6, begins with coordination of B(C_6_F_5_)_3_ (BCF) A to the thiosuccinimide 2a, forming the adduct B in an exergonic process (Δ*G* = −5.4 kcal mol^−1^). Formation of the thiirenium ion intermediate D can proceed *via* two distinct pathways. In the first step, B transfers the arylsulfenyl group to alkynyl phenol 1k through electrophilic addition, with a barrier of 24.0 kcal mol^−1^. Alternatively, intermediate C, generated from two molecules of B through coordination of both imide oxygens to B(C_6_F_5_)_3_ and regeneration of free 2a, can also deliver the arylsulfenyl group to 1k. Although formation of C is endergonic by 4.9 kcal mol^−1^, the subsequent arylsulfenyl group transfer from C occurs more readily than from B, indicated by barrier heights of 21.7 and 24.0 kcal mol^−1^, respectively. Once formed, intermediate D quickly isomerises to E and cyclises to give F. The oxonium ion in F is deprotonated by C’, the imide generated earlier during thiirenium ion formation to yield 3ka, the experimentally observed product. One of the Lewis acids bound in intermediate G subsequently transfers from G to a new molecule of 2a, generating succinimide H and regenerating B. Intermediate H then donates its coordinated B(C_6_F_5_)_3_ to B, reforming the reactive intermediate C. Notably, preferential coordination of B(C_6_F_5_)_3_ to the succinimide in H, rather than to thiosuccinimide 2a, slightly retards this transfer, raising the overall catalytic barrier by 0.9 kcal mol^−1^. The mechanistic framework remains essentially unchanged for other substrates examined in this study (1a and 4a), which exhibit identical key steps and comparable activation barriers for thiirenium ion formation and product generation (see SI, Schemes S19 and S20). The formation of 3-sulfenyl-2*H*-chromene was likewise investigated in detail, with computational analysis also performed (see SI, Scheme S19).

**Fig. 7:**
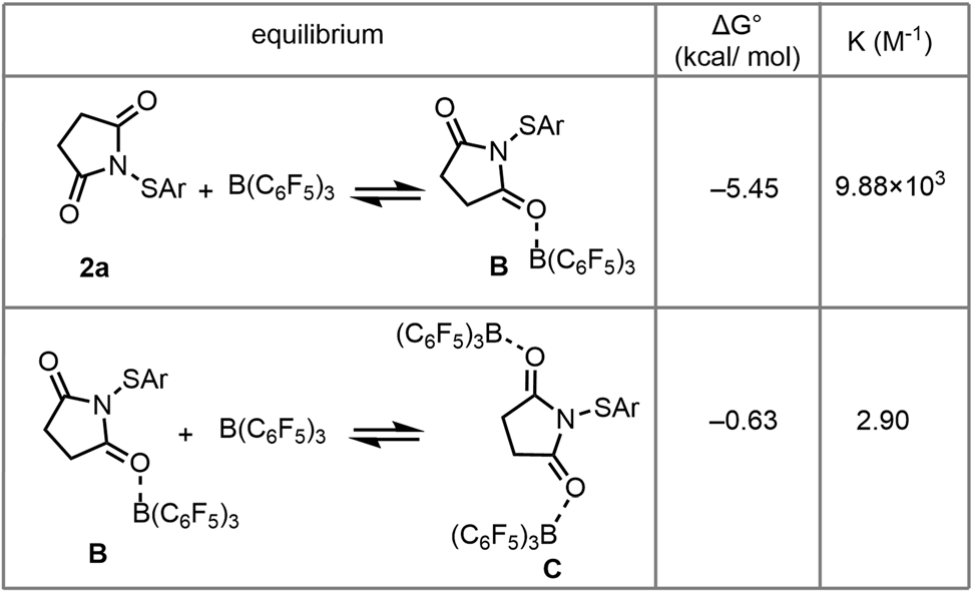
Computationally predicted equilibrium constants for the formation of complexes B and C from 2a and B(C_6_F_5_)_3_.

The reaction profile in Fig. 6 predicts that (1) 2a and B(C_6_F_5_)_3_ form B and C in an equilibrium before the rate-determining step, (2) the reaction is subject to inhibition by the by-product succinimide I because succinimide interacts strongly with B(C_6_F_5_)_3_, forming complex H, and (3) the initial reaction rate (*i.e.* the rate in the presence of negligible succinimide I) is given by the equation *rate* = *k* × [C] × [1a] and the reaction should therefore be first order in 1a. The calculations (*vide supra*) predict the equilibrium constants in Fig. 7. To confirm the equilibrium formation of B and C from B(C_6_F_5_)_3_ and 2a, B(C_6_F_5_)_3_ was titrated with 2a (Fig. S144). At molar ratios 2a:B(C_6_F_5_)_3_ up to 1.0, the titration shows clear changes in ^11^B and ^19^F chemical shifts of B(C_6_F_5_)_3_, with a break in the titration curve at a molar ratio of 1 : 1. These observations are in agreement with formation of a 1 : 1 complex with an affinity constant that is high enough to ensure full complexation under these conditions, *i.e.* in line with the calculated equilibrium constant of 9.88 × 10^3^ M^−1^. At low molar ratios 2a : B(C_6_F_5_)_3_ (between 0 and 0.2), the curves show some deviation from what otherwise looks like a simple 1 : 1 equilibrium. We attribute this deviation to the formation of complex C with an equilibrium constant that only results in partial complex formation, *i.e.* in line with the calculated equilibrium constant of 2.90 M^−1^. The overall shape of the curve is therefore in agreement with equilibrium formation of B and C, with C only forming to a measurable extent at low 2a : B(C_6_F_5_)_3_, *i.e.* high B(C_6_F_5_)_3_ : 2a. Kinetic studies were next carried out to confirm the other features predicted by the reaction profile. Because the reaction profile predicts inhibition by succinimide, we used initial rate kinetics to study the reaction. We followed the reaction using ^1^H NMR spectroscopy for 45 minutes, replacing the solvent with CDCl_3_. The experiments involved a reference experiment under optimised reaction conditions (100 mM 1a, 100 mM 2a, 10 mM B(C_6_F_5_)_3_, 45 °C) and an experiment involving 100 mM added succinimide I to confirm inhibition by the by-product (Fig. S145).

Fig. S145 shows that the reaction with added succinimide does not proceed, confirming the strong inhibitory effect of succinimide predicted by our calculations. The reference reaction, on the other hand, proceeded significantly in the dead time before the first spectrum was recorded (estimated to be ∼5 minutes), having reached conversions of almost 10% in the dead time. The reference trace does not extrapolate to 0% conversion at *t* = 0 s, even when taking into account the estimated dead time. This observation suggests that the reaction rate has already decreased significantly relative to initial rates, which again is in line with significant inhibition by succinimide. The initial experiments, therefore, show that the uninhibited initial rate of the reaction is significantly higher than anticipated.

**Fig. 8:**
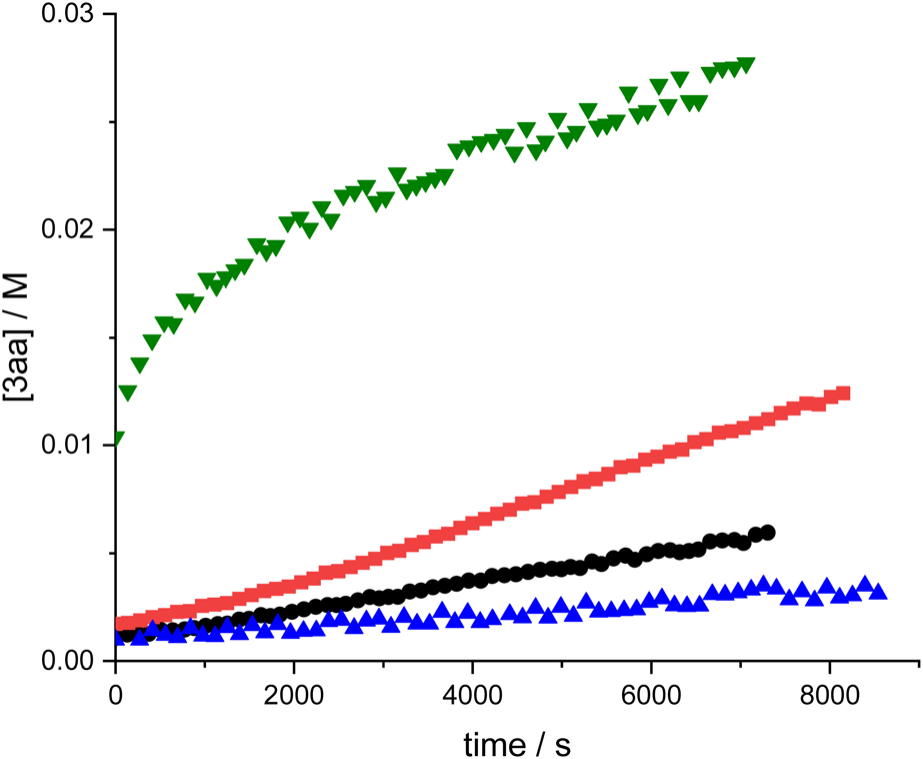
Concentration of 3aa as a function of time in experiments involving 50 mM 1a, 50 mM 2a, 5 mM B(C_6_F_5_)_3_ (reference experiment, (•), 100 mM 1a, 50 mM 2a, 5 mM B(C_6_F_5_)_3_ (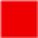), 50 mM 1a, 100 mM 2a, 5 mM B(C_6_F_5_)_3_(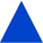), 50 mM 1a, 50 mM 2a, 10 mM B(C_6_F_5_)_3_(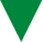); all in CDCl_3_, at 25 °C and using dichloroethane as internal standard.

To reduce the initial rate of the reaction, a second set of experiments was carried out, using concentrations of 50 mM 1a, 50 mM 2a, 5 mM B(C_6_F_5_)_3_, and a reaction temperature of 25 °C. As before, concentrations of 1a, 2a and B(C_6_F_5_)_3_ were doubled one by one, and the reaction was followed for 2 hours using ^1^H NMR spectroscopy (Fig. 8).

**Fig. 9:**
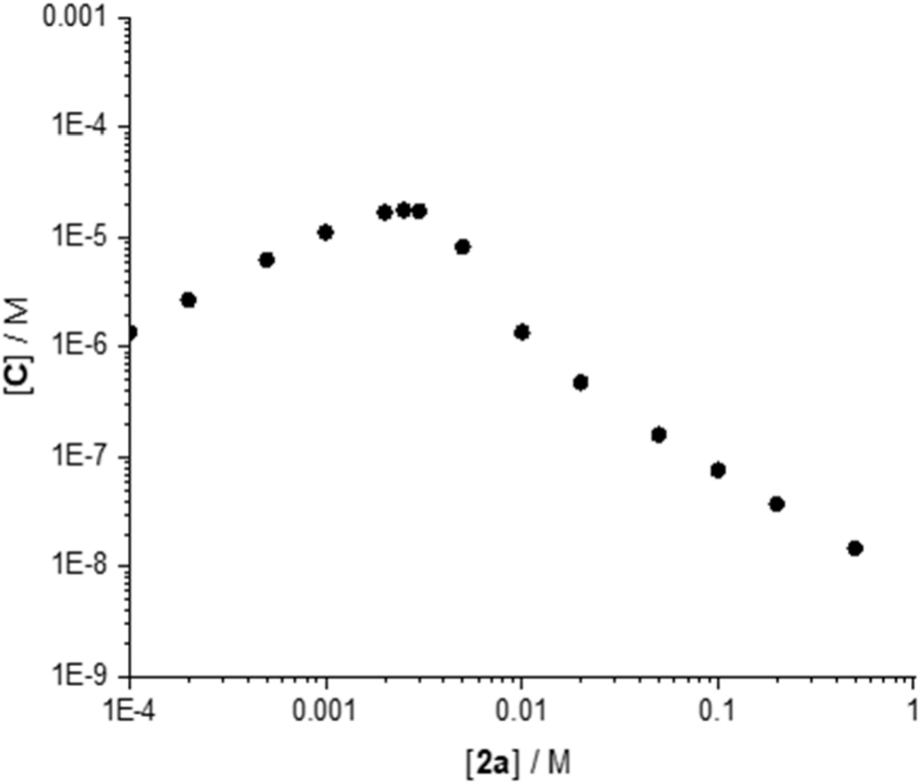
Concentration of C for a solution containing 5 mM B(C_6_F_5_)_3_ in the presence of varying concentrations of 2a, for the equilibrium system as defined in Fig. 7.

Fig. 8 shows that the initial rate doubles upon doubling the concentration of 1a, as expected for a reaction involving one molecule of 1a in the rate-determining step and as predicted by the anticipated rate law. Doubling the concentration of 2a, on the contrary, decreases the initial rate of the reaction. At first glance, this rate decrease is surprising because the reaction involves one molecule of 2a in the rate-determining step and one might therefore expect the reaction rate to increase with increasing concentrations of 2a. We note, however, that our titration above shows that, for constant [B(C_6_F_5_)_3_], C only forms to a measurable extent at low 2a:B(C_6_F_5_)_3_ (high B(C_6_F_5_)_3_:2a). An excess of 2a relative to B(C_6_F_5_)_3_, as is typical for reaction conditions, will therefore drive the equilibrium to B, reducing the concentration of reactive species C. To illustrate this behaviour, we simulated the equilibrium concentrations of C for a 5 mM solution of B(C_6_F_5_)_3_ (the concentrations used in the kinetic experiments here) in the presence of varying concentrations of 2a (Fig. 9 and Table S4).

Fig. 9 shows that, for concentrations of 2a up to 2.5 mM, *i.e.* ½ × [B(C_6_F_5_)_3_], [C] increases. Upon further increase of [2a] above ½ × [B(C_6_F_5_)_3_], however, the concentration of C decreases with increasing concentration of 2a. This decrease in [C] results in the observed decreased initial rate of the reaction with increasing [2a]. The behaviour of the equilibrium also explains the higher reaction rate upon doubling the concentration of B(C_6_F_5_)_3_ from 5 mM to 10 mM in the presence of 50 mM 2a; the concentration of [C] increases more than 4-fold upon doubling the concentration of B(C_6_F_5_)_3_ (Table S4).

Overall, the changes in the initial rates are in excellent agreement with the predictions of the calculated reaction profile and support the involvement of doubly-activated C on the reaction pathway. The inhibition by by-product succinimide explains the required longer reaction times to obtain synthetically acceptable yields, as well as the limited gains in yield upon much increased reaction times beyond the initial fast reaction phase.

**Fig. 10:**
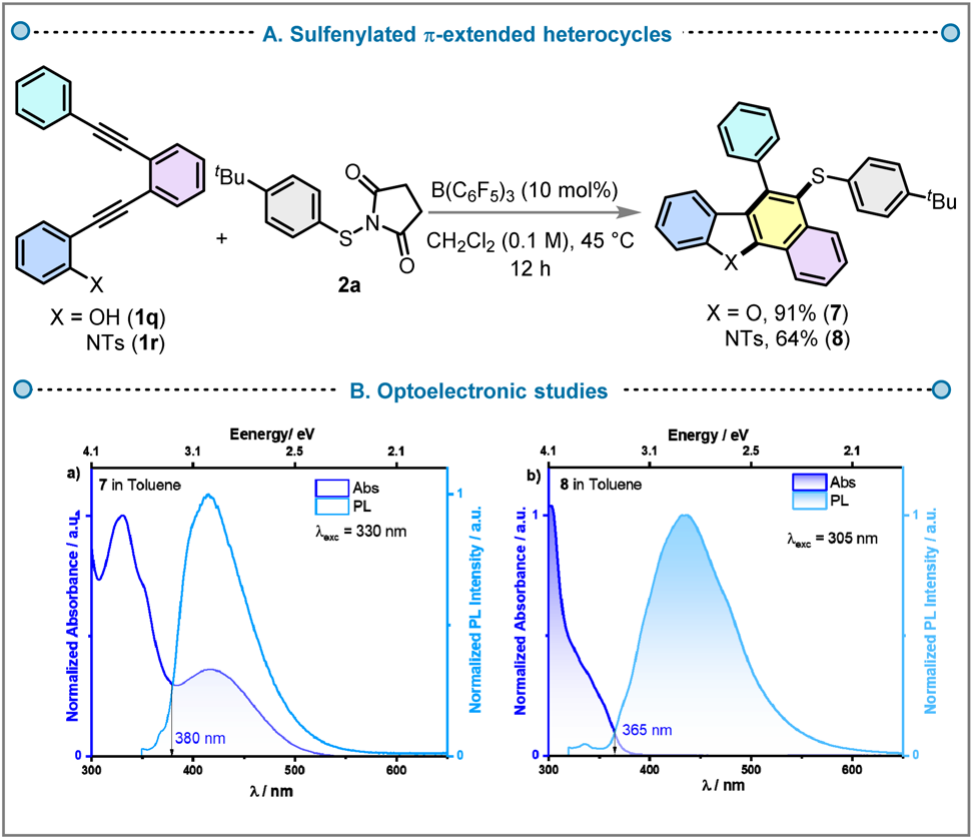
(A) Synthesis of sulfenylated benzo[*a*]carbazole or naphtho[1,2-*b*] benzofuran and their properties. (B) Molar absorptivity and normalised PL spectra of (a) 7 (*λ*_exc_ = 330 nm) and (b) 8 (*λ*_exc_ = 305 nm) in toluene.

To highlight the versatility of this methodology, we applied this methodology to the synthesis π-extended heteroaromatics through an APEX strategy (Fig. 10). The polyyne-type *N*-tosylanilines and 2-alkynyl phenol (1q and 1r) reacted with thiosuccinimide 2a to access highly π-extended heteroaromatics, including sulfenylated naphtho[1,2-*b*]benzofuran 7 and benzo[*a*]carbazole 8 in 91% and 64% yield, respectively. Naphtho[1,2-*b*]benzofuran and benzo[*a*]carbazole are significant heterocyclic frameworks found in bioactive natural products and synthetic compounds exhibiting properties such as anticancer and antimicrobial activities.^41–43^

These cores are valuable in optoelectronic applications like OLEDs, OPVs, and OFETs due to their excellent charge transport, luminescence efficiency, thermal stability, and chemical resilience, making them ideal for flexible, high-performance electronic devices.^44,45^ The introduction of a C–S bond in naphtho[1,2-*b*]benzofuran and benzo[*a*]carbazole can modulate their electronic properties, enhancing fluorescence, solubility, and charge transport, thereby improving their performance in optoelectronic applications and material stability. To understand the optoelectronic behaviour of the core molecule 7 and its derivative 8, we performed DFT calculations. The ground-state geometries were optimised at the PBE0/6-31G(d,p) level of theory in the gas phase, starting from structures initially generated in Chem3D Fig. S1. The electron density distributions in these two molecules are similar, localised mainly on the central core of naphthobenzofuran in 7 and benzocarbazole in 8. The highest occupied molecular orbital (HOMO) and lowest unoccupied molecular orbital (LUMO) are distributed across the whole molecule of 7, with HOMO/LUMO levels of −5.57/−1.09 eV. The HOMO level of 8 is relatively stabilised compared to that of 7 at −5.71 eV, while the LUMO is more pronouncedly stabilised to −1.46 eV, due to the presence of the tosyl (−NTs) group, which reduces the electron-donating strength of the carbazole. The HOMO–LUMO gap, Δ*E*_HOMO–LUMO_, thus decreases from 4.48 eV for 7 to 4.25 eV for 8. The excited-state properties were computed using time-dependent density functional theory (TD-DFT) within the Tamm-Dancoff approximation (TDA-DFT) on the optimised ground-state geometries. The oscillator strength (*f*) of the S_0_→S_1_ transition in 7 and 8 is identical (0.081), indicating substantial HOMO–LUMO overlap. The S_1_ energy of 7 is 3.71 eV, compared with 3.61 eV for 8 (Fig. S1 and S2), consistent with the trend in HOMO–LUMO gaps. The T_1_ energies are 2.79 eV for 7 and 2.64 eV for 8. The large S_1_–T_1_ gap (Δ*E*_ST_) indicates locally excited (LE) character and precludes thermally activated delayed fluorescence (TADF), consistent with the observed fluorescence (Fig. S2). Cyclic voltammetry (CV) and differential pulse voltammetry (DPV) measurements in CH_2_Cl_2_ with 0.1 M [^*n*^Bu_4_N]PF_6_ as the supporting electrolyte were used to infer the HOMO/LUMO levels of 7 and 8. The voltammograms are shown in Fig. S3 and the data are summarised in Table S2. The CV of 7 shows quasi-reversible oxidation and reversible wave at *E*_ox_ at 0.95 V and −1.72 V *vs.* SCE, which are assigned to the oxidation of the benzofuran^46^ and reduction of naphthalene,^47,48^ respectively. The corresponding HOMO/LUMO levels are −5.30/−2.63 eV. The CV of 8 only showed an irreversible oxidation wave at *E*_ox_ at 1.36 V *vs.* SCE, assigned to the oxidation of the carbazole,^49,50^ with a corresponding HOMO level of −5.75 eV. No reduction was observed in the CV of 8, so the LUMO level was estimated from the HOMO level and the optical band gap (*E*_opt_ = 3.39 eV), itself determined from the intersection of the normalised absorption and emission spectra in toluene (Fig. S4). The LUMO of 8 is thus at −2.36 eV, while the LUMO of 7, estimated in the same manner (*E*_opt_ at 3.27 eV) is −2.03 eV. The trend in the HOMO and LUMO levels aligns with the DFT calculations. The photophysical properties of 7 and 8 were studied in toluene solution (Fig. 10B and Table S3 in SI). There are three distinct absorption bands in the UV-Vis absorption spectra of 7, while there are only two bands in the spectrum of 8. The strong absorption band at 330 nm in 7 corresponds to a LE π–π* transition (*ε* = 16.7 × 10^3^ M^−1^ cm^−1^) while the LE band in 8 peaks at 305 nm (*ε* = 15.2 × 10^3^ M^−1^ cm^−1^. There is a weak charge transfer (CT) band observed for 7 at 415 nm (*ε* = 6.0 × 10^3^ M^−1^ cm^−1^), absent in 8, attributed to a transition from the *tert*-butyl-phenylthiodibenzofuran donor to the phenylnaphthalene acceptor (Fig. S1). Both molecules are weakly emissive in toluene when excited into their LE bands, emitting at 415 nm and 437 nm and having photoluminescence quantum yields (*Ø*_PL_) of 0.5% for 7 and 0.4% for 8 (Fig. 10B). There is also a very weak emission for 7 at 650 nm upon excitation into the CT band at 415 nm (Fig. S7). This could result from the population of a low-lying CT state in this compound. Time-resolved PL measurements in toluene at room temperature under air showed biexponential decay kinetics, with average PL lifetimes, *τ*_avg_, of 3.8 ns for 7 and 4.2 ns for 8 (Table S3 and Fig. S4).

## Conclusions

In summary, this work presents an amenable and efficient B(C_6_F_5_)_3_-catalysed metal-free route for the construction of sulfur-containing heterocycles *via* thiirenium ion intermediates from internal alkynes. The methodology not only offers a variety of 3-sulfenylated indoles and benzo[*b*]furans with broad substrate scope but also empowers the synthesis of structurally extended highly unsaturated heterocycles like benzo[*a*]carbazoles and naphtho[1,2-*b*]benzofurans exhibiting emission properties. Moreover, the ability to tune ring size from 5- to 6-membered frameworks in one pot, including sulfenylated 2*H*-chromenes, and dihydroquinoline and dihydronaphthalene derivatives, further highlights the synthetic flexibility of this protocol. The mechanistic insights support an intramolecular cyclisation *via* activation of internal alkynes through the formation of thiirenium ions, paving a unified pathway for diverse heterocyclic frameworks and opening new avenues in main group catalysed heterocycle synthesis.

## Author contributions

S. D., M. P., and R. L. M. conceptualised this work and designed the experiments. S. D. performed the experiments and analysed the data. S. D., M. P. and R. L. M drafted the initial manuscript. M.-H. B., T. W. and R. L. M. supervised the project. S. D. performed the kinetic experiments guided by. N. B. N. B. interpreted and analysed the kinetic data. J. W. performed and analysed the DFT calculations for the mechanistic investigation. J. W. and M.-H. B. wrote the mechanistic discussion in this work. A. K. G. and E. Z.-C. performed the optoelectronic studies, including data curation, formal analysis, theoretical calculations, and preparation of the corresponding section of the manuscript. All authors proofread and contributed to the final version of the manuscript.

## Conflicts of interest

The authors declare no competing financial interests.

## Supplementary Material

SC-OLF-D6SC01123C-s001

SC-OLF-D6SC01123C-s002

## Data Availability

The data supporting this article have been included as part of the Electronic supplementary information (SI). Supplementary information: experimental data for this article, including analytical spectra, are available. Cartesian coordinates, energies and frequencies of optimised structures used for the mechanistic investigation are presented in the supporting information of this work. Information about the data that underpins the results presented in this article can be found in the Cardiff University data catalogue at DOI: https://doi.org/10.17035/cardiff.31169080. See DOI: https://doi.org/10.1039/d6sc01123c.
