# Supplementary material for: Borane catalysed annulative sulfenylation of internal alkynes: towards the synthesis and study of fused heterocycles
Source: Chem Sci. 2026 Apr 29;17(23):11682–92. doi: 10.1039/d6sc01123c (PMC13151699; doi:10.1039/d6sc01123c)
Supplement: SC-017-D6SC01123C-s001 [file SC-017-D6SC01123C-s001.pdf]

Supplementary Information (SI) for Chemical Science.
This journal is © The Royal Society of Chemistry 2026

Electronic Supporting Information for

Borane Catalysed Annulative Sulfenylation of Internal Alkynes: Towards the Synthesis and Study of Fused Heterocycles

Sampurna Das,[a†] Milan Pramanik,[a†] Johannes Westphäling,[b,c] Abhishek Kumar Gupta,[d] Thomas Wirth,[e] Eli Zysman-Colman,[d] Niklaas J. Buurma,[e] Mu-Hyun Baik,[*,b,c] and Rebecca L. Melen[*,a]

[a] Ms Sampurna Das, Dr Milan Pramanik, Prof. Dr Rebecca L. Melen

Cardiff Catalysis Institute, School of Chemistry, Cardiff University, Translational Research Hub, Maindy Road, Cathays, Cardiff, CF24 4HQ, Cymru/Wales, U.K. Email: MelenR@cardiff.ac.uk.

[b] Mr Johannes Westphäling, Prof. Dr Mu-Hyun Baik

Department of Chemistry, Korea Advanced Institute of Science and Technology (KAIST), Daejeon 34141, Republic of Korea. Email: mbaik2805@kaist.ac.kr.

[c] Mr Johannes Westphäling, Prof. Dr Mu-Hyun Baik

Center for Catalytic Hydrocarbon Functionalisations, Institute for Basic Science (IBS), Daejeon 34141, Republic of Korea.

[d] Dr Abhishek Kumar Gupta, Prof. Dr Eli Zysman-Colman

EaStCHEM School of Chemistry, University of St Andrews, North Haugh, St. Andrews KY16 9ST, Scotland, U.K.

[e] Prof. Dr Thomas Wirth, Dr Niklaas J. Buurma

School of Chemistry, Cardiff University, Park Place, Main Building, Cardiff, CF10 3AT, Cymru/Wales, U.K.

† These authors contributed equally.

Table of contents

1. Experimental details	S3
2. Synthesis and spectral characterisation of starting materials	S4–S21
3. Synthesis and characterisation of products	S22–S39
4. Computational details	S40–S74
5. DFT calculations for PAHs	S75
6. Electrochemistry	S75
7. Photophysical measurements	S75–S81
8. References	S82–S83
9. NMR spectra	S84–S217
10. Kinetic studies	S218–S222

1. Experimental

1.1 General experimental

All reactions and manipulations were carried out under an atmosphere of dry, O_2-free nitrogen using standard double-manifold techniques with a rotary oil pump. A nitrogen-filled glove box (MBraun) was used to manipulate solids, including the storage of starting materials, ambient temperature reactions, product recovery and sample preparation for analysis. Solvents were dried by employing a solvent purification system MB SPS-800 and stored under a nitrogen atmosphere. Anhydrous (with Sure/Seal) 1,2-dichloroethane (1,2-$C_2H_4Cl_2$) was purchased from Merck and dried over molecular sieves before use. Chemicals were purchased from commercial suppliers and used as received. Thin-layer chromatography (TLC) was performed on pre-coated aluminium sheets of Merck silica gel 60 F254 (0.20 mm). ^{1}H, ^{13}C and ^{19}F NMR spectra were recorded on a Bruker Avance II 400 or Bruker Avance 500 spectrometer. ^{13}C NMR spectra were measured as ^{1}H decoupled. Yields are given as isolated yields. ^{13}C and ^{1}H NMR chemical shifts are expressed as parts per million (ppm, δ) downfield of tetramethylsilane (TMS) and are referenced to $CDCl_3$ (7.26/77.16 ppm) as an internal standard. The description of signals includes s = singlet, d = doublet, t = triplet, q = quartet, and m = multiplet. All coupling constants are absolute values and are expressed in Hertz (Hz). All spectra were analysed assuming a first order approximation. IR-Spectra were measured on a Shimadzu IR Affinity-1 photo-spectrometer. Mass spectra were measured on a Waters LCT Premier/XE or a Waters GCT Premier spectrometer. Ions were generated by the Atmospheric Solids, Analysis Probe (ASAP), Electrospray (ES), or Electron Ionisation (EI). The molecular ion peaks values quoted for either molecular ion (M^+), molecular ion plus or minus hydrogen ($M+H^+$, $M-H^-$), molecular ion plus sodium ($M+Na^+$).

2. Synthesis and characterisation of starting materials

2.1 Synthesis and characterisation of starting materials 1a–1r.

General procedure for the synthesis of 2-alkynylbenzenamines:

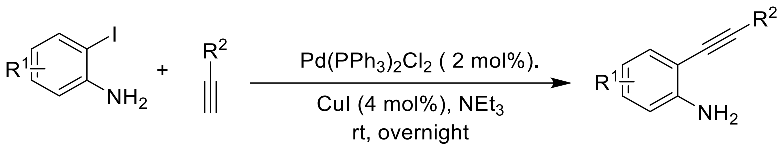

Scheme S1. Synthesis of 2-alkynylbenzenamines.

Adapting a known procedure, all *N*-protected 2-alkynylbenzenamines **1a–j** were prepared by reacting CuI (35 mg, 0.18 mmol, 4 mol%), $PdCl_2(PPh_3)_2$ (64 mg, 0.09 mmol, 2 mol%) and the corresponding alkyne (5.70 mmol, 1.1 equiv.) with a solution of the 2-iodoaniline (4.56 mmol, 1.0 equiv.) in Et_3N (2.5 mL, 18.24 mmol, 4.0 equiv.) at room temperature. The mixture was allowed to react overnight, concentrated under reduced pressure, and submitted to flash column chromatography on silica gel to afford the corresponding 2-alkynylbenzenamine compound which was then used directly for the synthesis of **1a**–**j**.

General procedure a for the synthesis of **1a**–**j**:

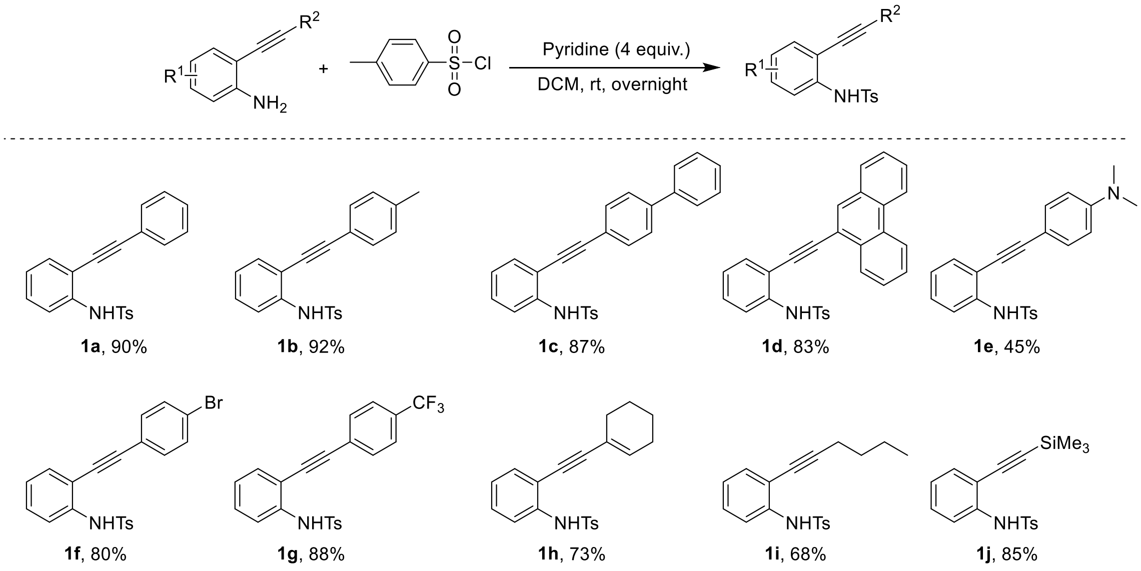

Scheme S2. Synthesis of *N*-protected 2-alkynylbenzenamines.

To a solution of the 2-alkynylbenzenamine (1.0 equiv.) prepared in step 1 and pyridine (4.0 equiv.) in 20 mL of dichloromethane was added 4-methylbenzene-1-sulfonyl chloride (1.3 equiv.) at 0 °C. The reaction solution was warmed to room temperature. After the reaction was complete, as

monitored by TLC, water was added, and the mixture was extracted with ether (3 × 30 mL). The combined organic phases were washed with brine and dried over anhydrous $MgSO_4$. The solvent was evaporated under reduced pressure, and the residue was purified by column chromatography on silica gel to afford the target compound. The spectroscopic data of the *N*-protected 2-alkynylbenzenamines are given below.

Synthesis of 4-Methyl-N-(2-(phenylethynyl)phenyl)benzenesulfonamide, **1a**[2]

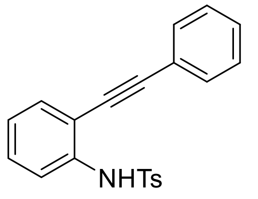

Synthesised in accordance with *General Procedure a* using 2-(ethynyl) benzenamine (500 mg, 2.57 mmol, 1 equiv.) and pyridine (0.79 mL, 10.28 mmol, 4 equiv.) in 20 mL of dichloromethane was added 4-methylbenzene-1-sulfonyl chloride (640 mg, 3.34 mmol, 1.3 equiv.). The crude product was purified via column chromatography using hexane/ethyl acetate (10:1 v/v) as eluent. The desired compound **1a** was obtained as a white solid. Yield: 804 mg, 2.31 mmol, 90%. **^{1}H NMR** (400 MHz, $CDCl_3$) δ: 7.69–7.65 (m, 2H), 7.62 (d, *J* = 8.5 Hz, 1H), 7.48–7.44 (m, 2H), 7.41–7.36 (m, 4H), 7.32–7.27 (m, 1H), 7.20–7.16 (m, 3H), 7.06 (td, *J* = 7.6, 1.2 Hz, 1H), 2.34 (s, 3H); **^{13}C NMR** (101 MHz, $CDCl_3$) δ: 144.1, 137.7, 136.7, 132.1, 131.7, 129.78, 129.76, 129.2, 128.7, 127.4, 124.7, 122.2, 120.4, 114.8, 96.3, 83.8, 21.7. The NMR data are in agreement with the previously reported literature values.

Synthesis of 4-methyl-N-(2-(p-tolylethynyl) phenyl)benzenesulfonamide, **1b**[2]

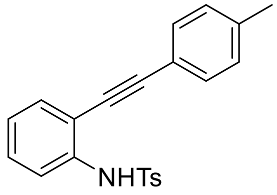

Synthesised in accordance with *General Procedure a* using 2-(p-tolylethynyl) aniline (500 mg, 2.38 mmol, 1 equiv.) and pyridine (0.73 mL, 9.52 mmol, 4 equiv.) in 20 mL of dichloromethane was added 4-methylbenzene-1-sulfonyl chloride (590 mg, 3.09 mmol, 1.3 equiv.). The crude product was purified via column chromatography using hexane/ethyl acetate (10:1 v/v) as eluent. The desired compound **1b** was obtained as a white solid. Yield: 791 mg, 2.18 mmol, 92%. **^{1}H NMR** (400 MHz, $CDCl_3$) δ: 7.69–7.65 (m, 2H), 7.62 (dd, *J* = 8.4, 1.4 Hz, 1H), 7.37–7.35 (m, 3H), 7.30–7.28 (m, 1H), 7.23–7.15 (m, 5H), 7.06 (td, *J* = 7.6, 1.2 Hz, 1H), 2.40 (s, 3H), 2.34 (s, 3H); **^{13}C NMR** (101 MHz, $CDCl_3$) δ: 144.1, 139.5, 137.6, 136.2, 132.0, 131.6, 129.7, 129.6, 127.7, 127.4, 124.7, 120.4, 119.0, 114.9, 96.6, 83.2, 21.74, 21.69. The NMR data are in agreement with the previously reported literature values.

Synthesis of N-(2-([1,1'-biphenyl]-4-ylethynyl)phenyl)-4-methylbenzenesulfonamide, **1c**[3]

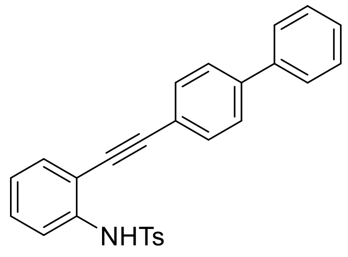

Synthesised in accordance with *General Procedure a* using 2-([1,1'-biphenyl]-4-ylethynyl)aniline (500 mg, 1.85 mmol, 1 equiv.) and pyridine (0.57 mL, 7.44 mmol, 4 equiv.) in 20 mL of dichloromethane was added 4-methylbenzene-1-sulfonyl chloride (460 mg, 2.40 mmol, 1.3 equiv.). The crude product was purified via column chromatography using hexane/ethyl acetate (15:1 v/v) as eluent. The desired compound **1c** was obtained as a white solid. Yield: 682 mg, 1.60 mmol, 87%. **^{1}H NMR** (400 MHz, $CDCl_3$) δ: 7.71–7.67 (m, 2H), 7.65–7.61 (m, 5H), 7.55–7.52 (m, 2H), 7.50–7.45 (m, 2H), 7.42–7.37 (m, 2H), 7.33–7.27 (m, 1H), 7.23–7.17 (m, 3H), 7.08 (td, J = 7.6, 1.2 Hz, 1H), 2.35 (s, 3H); **^{13}C NMR** (101 MHz, $CDCl_3$) δ: 144.2, 141.1, 140.2, 137.7, 136.2, 132.2, 129.78, 129.78, 129.1, 128.1, 127.44, 127.36, 127.2, 124.7, 121.0, 120.5, 114.8, 84.5, 96.2, 21.7. The NMR data are in agreement with the previously reported literature values.

Synthesis of 4-methyl-N-(2-(phenanthren-9-ylethynyl)phenyl)benzenesulfonamide, **1d**[4]

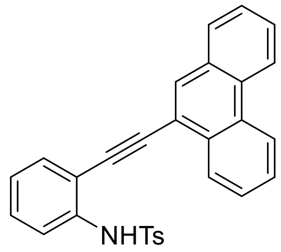

Synthesised in accordance with *General Procedure a* using 2-(phenanthren-9-ylethynyl)aniline (500 mg, 1.70 mmol, 1 equiv.) and pyridine (0.52 mL, 6.80 mmol, 4 equiv.) in 20 mL of dichloromethane was added 4-methylbenzene-1-sulfonyl chloride (422 mg, 2.21 mmol, 1.3 equiv.). The crude product was purified via column chromatography using hexane/ethyl acetate (15:1 v/v) as eluent. The desired compound **1d** was obtained as a yellow solid. Yield: 630 mg, 1.41 mmol, 83%. **^{1}H NMR** (400 MHz, $CDCl_3$) δ: 8.76–8.68 (m, 2H), 8.32–8.28 (m, 1H), 8.03 (s, 1H), 7.92 (d, J = 7.8, 1H), 7.77–7.63 (m, 7H), 7.54 (dd, J = 7.8, 1.6 Hz, 1H), 7.39–7.33 (m, 2H), 7.17–7.10 (m, 3H), 2.28 (s, 3H); **^{13}C NMR** (101 MHz, $CDCl_3$) δ: 144.1, 137.7, 136.3, 132.6, 132.4, 131.1, 130.8, 130.7, 130.3, 129.9, 129.8, 128.9, 128.1, 127.5, 127.43, 127.39, 127.3, 126.7, 124.8, 123.1, 122.8, 120.7, 118.6, 115.0, 94.6, 88.2, 21.6. The NMR data are in agreement with the previously reported literature values.

Synthesis of N-(2-((4-(dimethylamino)phenyl)ethynyl)phenyl)-4-methylbenzenesulfonamide, **1e**

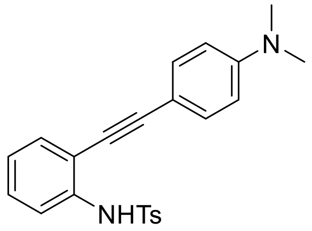

Synthesised in accordance with *General Procedure a* using 4-((2-aminophenyl) ethynyl)-*N,N*-dimethylaniline (500 mg, 2.11 mmol, 1 equiv.) and pyridine (0.65 mL, 8.44 mmol, 4 equiv.) in 20 mL of dichloromethane was added 4-methylbenzene-1-sulfonyl chloride (524 mg, 2.75 mmol, 1.3 equiv.). The crude product was purified via column chromatography using hexane/ethyl acetate (15:1 v/v) as eluent. The desired compound **1e** was obtained as a yellow oil. Yield: 370 mg, 0.94 mmol, 45%. **^{1}H NMR** (400 MHz, $CDCl_3$) δ: 7.69–7.66 (m, 2H), 7.61 (d, *J* = 8.3 Hz, 1H), 7.40–7.31 (m, 4H), 7.25–7.21 (m, 1H), 7.16 (dd, *J* = 8.6, 0.8 Hz, 2H), 7.03 (td, *J* = 7.6, 1.2 Hz, 1H), 6.70–6.65 (m, 2H), 3.03 (s, 6H), 2.34 (s, 3H); **^{13}C NMR** (101 MHz, $CDCl_3$) δ: 150.7, 144.0, 137.3, 136.2, 133.8, 131.6, 129.7, 128.8, 127.4, 124.6, 120.2, 115.7, 111.9, 108.6, 97.9, 81.9, 40.3, 21.7; **HRMS** (ES^+) m/z: $[M+H]^+$ calculated for $[C_{23}H_{23}N_2O_2S]^+$: 391.1480, found 391.1480; **IR ν_{max}** (cm^{-1}): 2972, 2271, 1686, 1593, 1522, 1492, 1366, 1162, 1092, 902, 818, 724, 652.

Synthesis of N-(2-((4-bromophenyl)ethynyl)phenyl)-4-methylbenzenesulfonamide, **1f**[5]

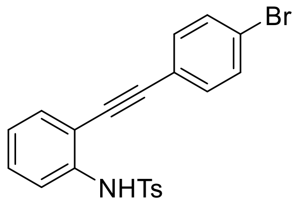

Synthesised in accordance with *General Procedure a* using 2-((4-bromophenyl)ethynyl)aniline (500 mg, 1.83 mmol, 1 equiv.) and pyridine (0.56 mL, 7.34 mmol, 4 equiv.) in 20 mL of dichloromethane was added 4-methylbenzene-1-sulfonyl chloride (453 mg, 2.38 mmol, 1.3 equiv.). The crude product was purified via column chromatography using hexane/ethyl acetate (15:1 v/v) as eluent. The desired compound **1f** was obtained as a colourless solid. Yield: 624 mg, 1.46 mmol, 80%. **^{1}H NMR** (400 MHz, $CDCl_3$) δ: 7.66 (d, *J* = 8.4 Hz, 2H), 7.61 (d, *J* = 8.3 Hz, 1H), 7.54–7.50 (m, 2H), 7.37 (dd, *J* = 7.8, 1.6 Hz, 1H), 7.34–7.28 (m, 3H), 7.17 (d, *J* = 7.9 Hz, 3H), 7.07 (td, *J* = 7.6, 1.2 Hz, 1H), 2.34 (s, 3H); **^{13}C NMR** (101 MHz, $CDCl_3$) δ: 144.2, 137.7, 136.2, 133.0, 132.2, 131.0, 130.0, 129.8, 127.4, 124.8, 123.5, 121.1, 120.6, 114.5, 95.0, 85.0, 21.7. The NMR data are in agreement with the previously reported literature values.

Synthesis of 4-methyl-N-(2-((4-(trifluoromethyl)phenyl)ethynyl)phenyl)benzenesulfonamide, **1g**[6]

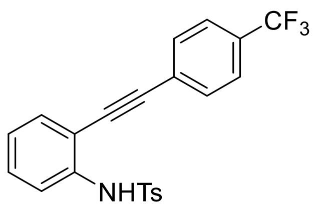

Synthesised in accordance with *General Procedure a* using 2-((4-(trifluoromethyl)phenyl)ethynyl)aniline (500 mg, 1.91 mmol, 1 equiv.) and pyridine (0.59 mL, 7.64 mmol, 4 equiv.) in 20 mL of dichloromethane was added 4-methylbenzene-1-sulfonyl chloride (474 mg, 2.48 mmol, 1.3 equiv.). The crude product was purified via column chromatography using hexane/ethyl acetate (10:1 v/v) as eluent. The desired compound **1g** was obtained as a white solid. Yield: 697 mg, 1.68 mmol, 88%. **^{1}H NMR** (400 MHz, $CDCl_3$) δ: 7.69–7.60 (m, 5H), 7.56 (d, J = 8.8 Hz, 2H), 7.40 (dd, J = 7.8, 1.5 Hz, 1H), 7.35 (d, J = 7.7 Hz, 1H), 7.18 (dd, J = 8.6, 0.8 Hz, 2H), 7.13 (s, 1H), 7.09 (td, J = 7.6, 1.2 Hz, 1H), 2.35 (s, 3H); **^{13}C NMR** (101 MHz, $CDCl_3$) δ: 144.3, 137.9, 136.3, 132.4, 131.9, 131.0, 130.4, 129.8, 127.4, 126.0, 125.6 (q, J = 12 Hz), 124.8, 120.6, 114.0, 94.6, 86.2, 21.7; **^{19}F NMR** (376 MHz, $CDCl_3$) δ: -62.88. The NMR data are in agreement with the previously reported literature values.

Synthesis of N-(2-(cyclohex-1-en-1-ylethynyl)phenyl)-4-methylbenzenesulfonamide, **1h**[2]

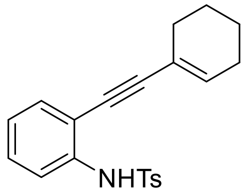

Synthesised in accordance with *General Procedure a* using 2-(cyclohex-1-en-1-ylethynyl)aniline (500 mg, 2.53 mmol, 1 equiv.) and pyridine (0.78 mL, 10.12 mmol, 4 equiv.) in 20 mL of dichloromethane was added 4-methylbenzene-1-sulfonyl chloride (625 mg, 3.28 mmol, 1.3 equiv.). The crude product was purified via column chromatography using hexane/ethyl acetate (15:1 v/v) as eluent. The desired compound **1h** was obtained as a yellow liquid. Yield: 649 mg, 1.84 mmol, 73%. **^{1}H NMR** (400 MHz, $CDCl_3$) δ: 7.68–7.64 (m, 2H), 7.57 (dd, J = 8.3, 1.3 Hz, 1H), 7.25–7.14 (m, 5H), 7.00 (td, J = 7.6, 1.2 Hz, 1H), 6.22–6.19 (m, 1H), 2.36 (s, 3H), 2.26–2.20 (m, 4H), 1.72–1.64 (m, 4H); **^{13}C NMR** (101 MHz, $CDCl_3$) δ: 144.0, 137.5, 136.9, 136.2, 131.8, 129.7, 129.2, 127.4, 124.5, 120.0, 119.8, 115.0, 98.4, 81.2, 29.3, 25.9, 22.3, 21.7, 21.5. The NMR data are in agreement with the previously reported literature values.

Synthesis of N-(2-(hex-1-yn-1-yl)phenyl)-4-methylbenzenesulfonamide, **1i**[7]

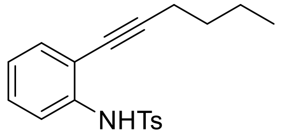

Synthesised in accordance with *General Procedure a* using 2-(hex-1-yn-1-yl)aniline (500 mg, 2.26 mmol, 1 equiv.) and pyridine (0.70 mL, 9.04 mmol, 4 equiv.) in 20 mL of dichloromethane was added 4-methylbenzene-1-sulfonyl chloride (560 mg, 2.93 mmol, 1.3 equiv.). The crude product was

purified via column chromatography using hexane/ethyl acetate (15:1 v/v) as eluent. The desired compound **1i** was obtained as a yellow oil. Yield: 503 mg, 1.53 mmol, 68%. **^{1}H NMR** (400 MHz, $CDCl_3$) δ: 7.75 (d, *J* = 8.3, 2H), 7.56 (d, *J* = 8.3, 1H), 7.25–7.18 (m, 5H), 6.98 (td, *J* = 7.6, 1.1 Hz, 1H), 2.41 (t, *J* = 7.0 Hz, 2H), 2.37 (s, 3H), 1.62–1.56 (m, 2H), 1.52–1.40 (m, 2H), 0.97 (t, *J* = 7.3 Hz, 3H); **^{13}C NMR** (101 MHz, $CDCl_3$) δ: 144.0, 137.7, 136.3, 132.0, 129.7, 128.9, 127.4, 124.3, 119.5, 115.0, 98.0, 75.5, 30.8, 22.2, 21.7, 19.3, 13.7. The NMR data are in agreement with the previously reported literature values.

Synthesis of 4-methyl-N-(2-((trimethylsilyl)ethynyl)phenyl)benzenesulfonamide, **1j**[8]

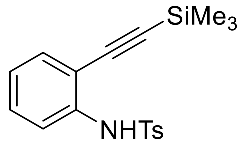

Synthesised in accordance with *General Procedure a* using 2-((trimethylsilyl)ethynyl)aniline (500 mg, 2.26 mmol, 1 equiv.) and pyridine (0.89 mL, 11.48 mmol, 4 equiv.) in 20 mL of dichloromethane was added 4-methylbenzene-1-sulfonyl chloride (711 mg, 3.73 mmol, 1.3 equiv.). The crude product was purified via column chromatography using hexane/ethyl acetate (10:1 v/v) as eluent. The desired compound **1j** was obtained as a white solid. Yield: 660 mg, 1.92 mmol, 85%. **^{1}H NMR** (400 MHz, $CDCl_3$) δ: 7.64 (d, *J* = 8.4, 2H), 7.62–7.58 (m, 1H), 7.31–7.26 (m, 2H), 7.25–7.17 (m, 3H), 7.00 (td, *J* = 7.6, 1.1 Hz, 1H), 2.37 (s, 3H), 0.27 (s, 9H); **^{13}C NMR** (101 MHz, $CDCl_3$) δ: 144.1, 138.3, 136.2, 132.1, 129.9, 129.7, 127.4, 124.4, 119.9, 114.3, 102.4, 99.6, 21.7, 0.0. The NMR data are in agreement with the previously reported literature values.

General procedure b for the synthesis of **1k–p**:

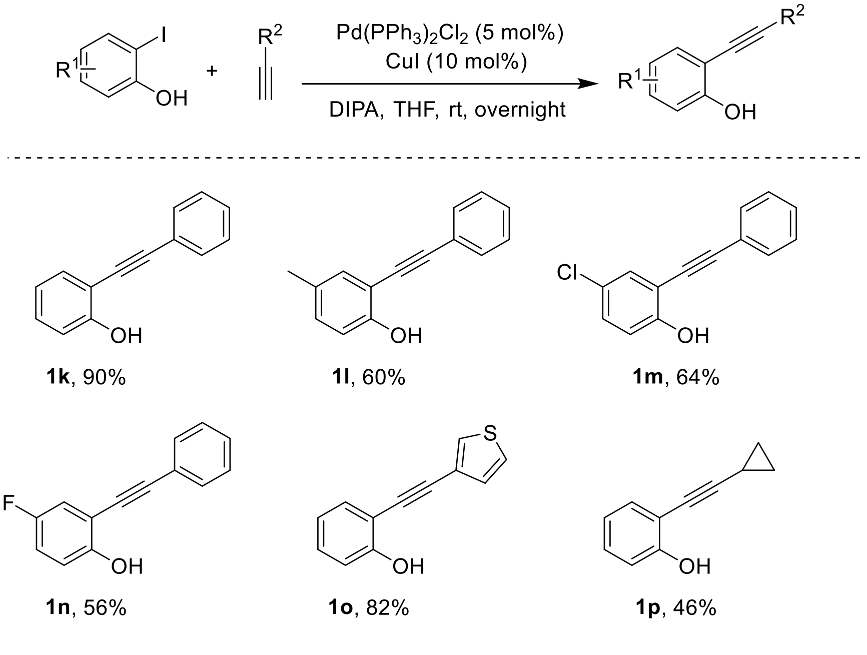

Scheme S3. Synthesis of 2-alkynylphenols.

Adapting known procedures, all 2-alkynylphenol **1k–p** were prepared by reacting CuI (42 mg, 0.22 mmol, 10 mol%), $PdCl_2(PPh_3)_2$ (30 mg, 0.11 mmol, 5 mol%) and the corresponding alkyne (2.48 mmol, 1.1 equiv.) with a stirred solution of corresponding 2-iodophenol (2.26 mmol, 1.0 equiv.) in 25 mL THF at room temperature. Diisopropylamine (DIPA, 2.56 mmol, 1.2 equiv.) was then added dropwise. The mixture was allowed to react overnight, concentrated under reduced pressure, and submitted to flash column chromatography on silica gel to afford the target compound. The spectroscopic data of the starting materials are given below.

Synthesis of 2-(phenylethynyl)phenol, **1k**[9]

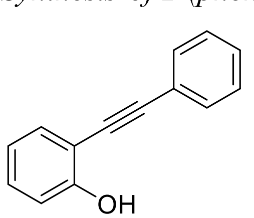

Synthesised in accordance with *General Procedure b*. CuI (42 mg, 0.1 mmol, 10 mol%), $PdCl_2(PPh_3)_2$ (80 mg, 0.11 mmol, 5 mol%) and ethynylbenzene (0.27 mL, 2.48 mmol, 1.1 equiv.) were added to a stirred solution of 2-iodophenol (500 mg, 2.26 mmol, 1.0 equiv.) in 25 mL of THF at room temperature. DIPA (0.38 mL, 2.71 mmol, 1.2 equiv.) was then added dropwise. The crude product was purified via column chromatography using hexane/ethyl acetate (10:1 v/v) as eluent. The desired compound **1k** was obtained as a brown solid. Yield: 395 mg, 2.03 mmol, 90%. **^{1}H NMR** (400 MHz, $CDCl_3$) δ: 7.60–7.53 (m, 2H), 7.45 (d, J = 7.6 Hz, 1H), 7.41–7.36 (m, 3H), 7.29 (t, J = 7.7 Hz, 1H), 7.01 (d, J = 8.3 Hz, 1H), 6.93 (t, J = 7.6 Hz, 1H), 5.88 (s, 1H); **^{13}C NMR** (101 MHz, $CDCl_3$) δ: 156.6, 131.8, 131.7, 130.6, 129.0, 128.6, 122.5, 120.6, 114.8, 109.7, 96.5, 83.1. The NMR data are in agreement with the previously reported literature values.

Synthesis of 4-methyl-2-(phenylethynyl)phenol, **1l**[10]

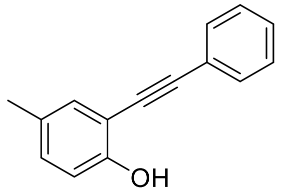

Synthesised in accordance with *General Procedure b*. CuI (41 mg, 0.2 mmol, 10 mol%), $PdCl_2(PPh_3)_2$ (81 mg, 0.11 mmol, 5 mol%) and ethynylbenzene (0.25 mL, 2.35 mmol, 1.1 equiv.) were added to a stirred solution of 2-iodo-4-methylphenol (500 mg, 2.13 mmol, 1.0 equiv.) in 25 mL of THF at room temperature. DIPA (0.35 mL, 2.55 mmol, 1.2 equiv.) was then added dropwise. The crude product was purified via column chromatography using hexane/ethyl acetate (10:1 v/v) as eluent. The desired compound **1l** was obtained as a yellow liquid. Yield: 266 mg, 1.27 mmol, 60%. **^{1}H NMR** (400 MHz, $CDCl_3$) δ: 7.56–7.51 (m, 2H), 7.39–7.36 (m, 3H), 7.24–7.23 (m, 1H), 7.09–7.06 (m, 1H), 6.88 (d, J = 8.3 Hz, 1H), 5.68 (d, J = 2.4 Hz, 1H), 2.28 (s, 3H); **^{13}C NMR** (101

MHz, $CDCl_3$) δ: 154.5, 131.9, 131.7, 131.4, 129.8, 128.9, 128.6, 122.6, 114.6, 109.3, 96.2, 83.4, 20.5. The NMR data are in agreement with the previously reported literature values.

Synthesis of 4-chloro -2-(phenylethynyl)phenol, **1m**[10]

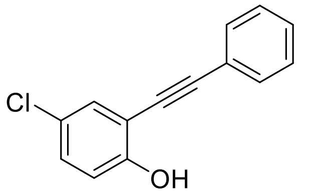

Synthesised in accordance with *General Procedure b.* CuI (38 mg, 0.2 mmol, 10 mol%), $PdCl_2(PPh_3)_2$ (69 mg, 0.09 mmol, 5 mol%) and ethynylbenzene (0.24 mL, 2.16 mmol, 1.1 equiv.) were added to a stirred solution of 2-iodo-4-chlorophenol (500 mg, 1.96 mmol, 1.0 equiv.) in 25 mL of THF at room temperature. DIPA (0.32 mL, 2.35 mmol, 1.2 equiv.) was then added dropwise. The crude product was purified via column chromatography using hexane/ethyl acetate (10:1 v/v) as eluent. The desired compound **1m** was obtained as a yellow liquid. Yield: 286 mg, 1.25 mmol, 64%. **^{1}H NMR** (400 MHz, $CDCl_3$) δ: 7.56–7.52 (m, 2H), 7.41–7.37 (m, 4H), 7.22 (dd, J = 8.8, 2.5 Hz, 1H), 6.92 (d, J = 8.8 Hz, 1H), 5.81 (s, 1H); **^{13}C NMR** (101 MHz, $CDCl_3$) δ: 155.3, 131.8, 131.1, 130.6, 129.3, 128.7, 125.2, 122.0, 116.2, 111.22, 97.4, 81.9. The NMR data are in agreement with the previously reported literature values.

Synthesis of 4-fluoro-2-(phenylethynyl)phenol, **1n**[10]

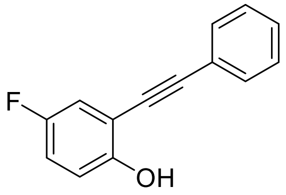

Synthesised in accordance with *General Procedure b.* CuI (40 mg, 0.2 mmol, 10 mol%), $PdCl_2(PPh_3)_2$ (74 mg, 0.10 mmol, 5 mol%) and ethynylbenzene (0.25 mL, 2.31 mmol, 1.1 equiv.) were added to a stirred solution of 2-iodo-4-fluorophenol (500 mg, 2.10 mmol, 1.0 equiv.) in 25 mL of THF at room temperature. DIPA (0.35 mL, 2.52 mmol, 1.2 equiv.) was then added dropwise. The crude product was purified via column chromatography using hexane/ethyl acetate (10:1 v/v) as eluent. The desired compound **1n** was obtained as a yellow liquid. Yield: 250 mg, 1.17 mmol, 56%. **^{1}H NMR** (400 MHz, $CDCl_3$) δ: 7.86–7.84 (m, 2H), 7.48–7.42 (m, 3H), 7.39–7.36 (m, 1H), 7.23 (dd, J = 8.6, 2.6 Hz, 1H), 7.02–6.97 (m, 2H); **^{13}C NMR** (101 MHz, $CDCl_3$) δ: 160.9, 157.9, 132.7, 129.1, 129.0, 128.6, 125.2, 112.0 (d, J = 21.0 Hz), 111.8 (d, J = 4.4 Hz), 106.5 (d, J = 24.5 Hz), 101.5 (d, J = 3.6 Hz), 87.8; **^{19}F NMR** (376 MHz, $CDCl_3$) δ: -121.0. The NMR data are in agreement with the previously reported literature values.

Synthesis of 2-(thiophen-3-ylethynyl)phenol, **1o**[10]

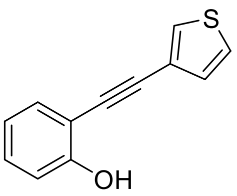

Synthesised in accordance with *General Procedure b*. CuI (47 mg, 0.2 mmol, 10 mol%), $PdCl_2(PPh_3)_2$ (87 mg, 0.12 mmol, 5 mol%) and 3-ethynylthiophene (0.24 mL, 2.49 mmol, 1.1 equiv.) were added to a stirred solution of 2-iodo phenol (500 mg, 2.26 mmol, 1.0 equiv.) in 25 mL of THF at room temperature. DIPA (0.41 mL, 2.98 mmol, 1.2 equiv.) was then added dropwise. The crude product was purified via column chromatography using hexane/ethyl acetate (10:1 v/v) as eluent. The desired compound **1o** was obtained as a white solid. Yield: 371 mg, 1.85 mmol, 82%. **^{1}H NMR** (400 MHz, $CDCl_3$) δ: 7.57 (dd, J = 3.0, 1.3 Hz, 1H), 7.41 (dd, J = 7.7, 1.7 Hz, 1H), 7.34 (dd, J = 5.0, 2.9 Hz, 1H), 7.29–7.26 (m, 1H), 7.21 (dd, J = 5.0, 1.1 Hz, 1H), 6.98 (dd, J = 8.3, 1.3 Hz, 1H), 6.91 (td, J = 7.6, 1.1 Hz, 1H), 5.81 (s, 1H); **^{13}C NMR** (101 MHz, $CDCl_3$) δ: 156.6, 131.8, 130.6, 129.9, 129.5, 125.9, 121.5, 120.6, 114.8, 109.7, 91.5, 82.7. The NMR data are in agreement with the previously reported literature values.

Synthesis of 2-(cyclopropylethynyl)phenol, **1p**[10]

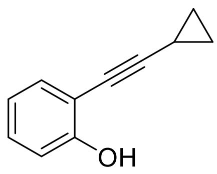

Synthesised in accordance with *General Procedure b*. CuI (43 mg, 0.22 mmol, 10 mol%), $PdCl_2(PPh_3)_2$ (80 mg, 0.11 mmol, 5 mol%) and ethynylcyclopropane (0.21 mL, 2.49 mmol, 1.1 equiv.) were added to a stirred solution of 2-iodo phenol (500 mg, 2.26 mmol, 1.0 equiv.) in 25 mL of THF at room temperature. DIPA (0.38 mL, 2.71 mmol, 1.2 equiv.) was then added dropwise. The crude product was purified via column chromatography using hexane/ethyl acetate (10:1 v/v) as eluent. The desired compound **1p** was obtained as a yellow liquid. Yield: 164 mg, 1.03 mmol, 46%. **^{1}H NMR** (400 MHz, $CDCl_3$) δ: 7.29–7.25 (m, 2H), 7.21–7.16 (m, 1H), 6.91 (dd, J = 8.3, 1.3 Hz, 1H), 6.82 (td, J = 7.5, 1.2 Hz, 1H), 1.55–1.48 (m, 1H), 0.96–0.89 (m, 2H), 0.86–0.82 (m, 2H); **^{13}C NMR** (101 MHz, $CDCl_3$) δ: 156.9, 131.7, 129.7, 120.3, 114.4, 107.4, 101.2, 69.7, 9.2, 0.3. The NMR data are in agreement with the previously reported literature values.

Synthesis of 2-((2-(phenylethynyl)phenyl) ethynyl)phenol, **1q**

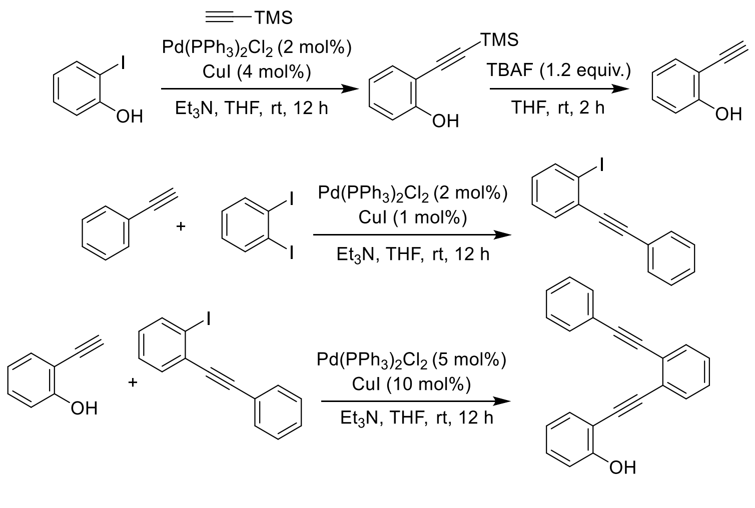

Scheme S4. Synthesis of 2-((2-(phenylethynyl)phenyl) ethynyl)phenol, **1q**.

To a dried Schlenk flask, a solution of 2-iodophenol (1.00 g, 4.54 mmol) in 40 mL of THF and Et_3N (10 mL), CuI (35 mg, 0.182 mmol, 4 mol%), $Pd(PPh_3)_2Cl_2$ (64 mg, 0.091 mmol, 2 mol%), and ethynyltrimethylsilane (1.28 mL, 9.09 mmol, 2.0 equiv.) were added at room temperature. The mixture was stirred for 12 h and quenched by addition of saturated aqueous NH_4Cl (1 × 30 mL), at room temperature. The aqueous phase was extracted with CH_2Cl_2 (3 × 30 mL) and the layers were separated. The combined organic layers were washed with brine (1 × 40 mL), dried over $MgSO_4$, filtered, and concentrated in vacuo. The residue was purified by column chromatography as an orange viscous oil to give intermediate 2-((trimethylsilyl)ethynyl)phenol.

To a solution of 2-((trimethylsilyl)ethynyl)phenol (700 mg, 3.68 mmol) in 35 mL of dry THF, TBAF (4.6 mL, 1.0 M in THF, 1.2 equiv.) was added. The mixture was stirred at room temperature for 2 h. The mixture was quenched by the addition of saturated aqueous NH_4Cl (1 × 30 mL), at room temperature. The aqueous phase was extracted with diethyl ether (3 × 30 mL), and the layers were separated. The combined organic layers were washed with brine (1 × 40 mL), dried over $MgSO_4$, filtered, and concentrated in vacuo to afford 2-ethynylphenol as a brown oil.

To a solution of phenylacetylene (0.4 mL, 3.6 mmol, 1.2 equiv.) in Et_3N (8 mL), $Pd(PPh_3)_2Cl_2$ (42.8 mg, 0.061 mmol, 2 mol%), CuI (5.9 mg, 0.031 mmol, 1 mol%), and 1,2-diiodobenzene (1.0 g, 3.06 mmol) were added. The mixture was quenched by the addition of saturated aqueous NH_4Cl (1 × 30 mL), at room temperature. The aqueous phase was extracted with EtOAc (3 × 30 mL) and the layers were separated. The combined organic layers were washed with brine (1 × 40 mL), dried

over $MgSO_4$, filtered, and concentrated in vacuo. The residue was purified by column chromatography as to afford 1-iodo-2-(phenylethynyl)benzene as a white solid.

To a solution of 1-iodo-2-(phenylethynyl)benzene (480 mg, 1.57 mmol) in Et_3N (4 mL), $Pd(PPh_3)_2Cl_2$ (55 mg, 0.078 mmol, 5 mol%), CuI (29.8 mg, 0.15 mmol, 10 mol%), and 2-ethynylphenol (223 mg, 1.89 mmol, 1.2 equiv.) in Et_3N (3 mL) were added. The mixture was quenched by the addition of saturated aqueous NH_4Cl (1 × 30 mL), at room temperature. The aqueous phase was extracted with EtOAc (3 × 30 mL) and the layers were separated. The combined organic layers were washed with brine (1 × 40 mL), dried over $MgSO_4$, filtered, and concentrated in vacuo. The crude product was purified *via* column chromatography using hexane/ethyl acetate (15:1 v/v) as eluent. The desired compound was obtained as a yellow solid. Yield: 222 mg, 0.75 mmol, 48%. **^{1}H NMR** (400 MHz, $CDCl_3$) δ: 7.63–7.57 (m, 4H), 7.45 (dd, *J* = 7.7, 1.7 Hz, 1H), 7.39–7.34 (m, 5H), 7.30–7.25 (m, 1H), 7.00–6.97 (m, 1H), 6.91 (td, *J* = 7.5, 1.1 Hz, 1H), 6.36 (s, 1H); **^{13}C NMR** (101 MHz, $CDCl_3$) δ: 157.2, 132.5, 132.1, 131.5, 131.3, 130.9, 128.9, 128.5, 128.4, 125.6, 124.8, 122.7, 120.4, 114.9, 109.6, 95.7, 94.1, 88.4, 87.6; **HRMS** (ES^+) m/z: $[M+H]^+$ calculated for $[C_{22}H_{15}O]^+$: 295.1127, found 295.1123; **IR ν_{max}** (cm^{-1}): 3054, 2963, 2243, 1599, 1492, 1444, 1175, 900, 808, 730.

Synthesis of 4-methyl-N-(2-((2 (phenylethynyl)phenyl)ethynyl)phenyl)benzenesulfonamide, **1r**

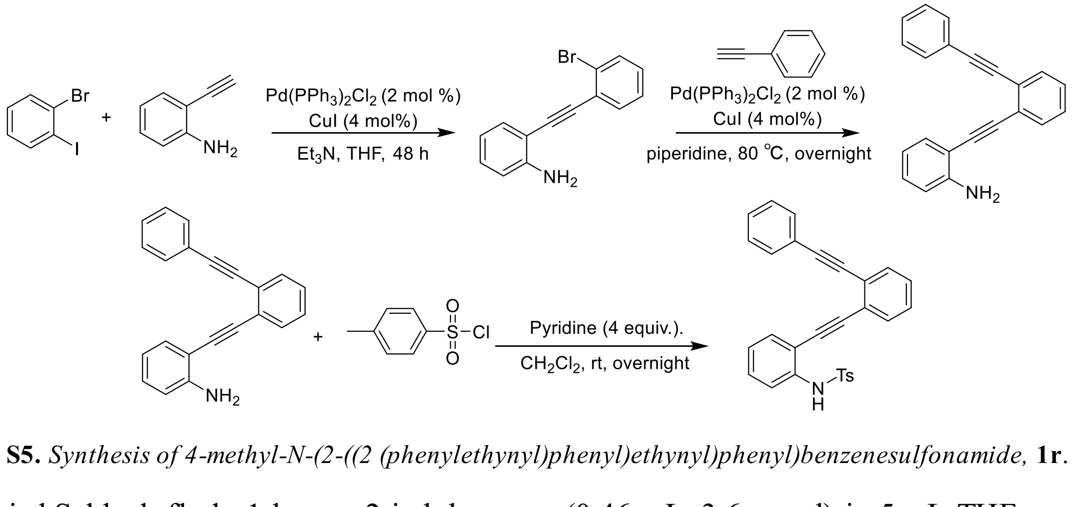

Scheme S5. *Synthesis of 4-methyl-N-(2-((2 (phenylethynyl)phenyl)ethynyl)phenyl)benzenesulfonamide,* **1r**.

To a dried Schlenk flask, 1-bromo-2-iodobenzene (0.46 mL, 3.6 mmol) in 5 mL THF was added Et_3N (2.5 mL), CuI (16.8 mg, 0.088 mmol, 4 mol%), $PdCl_2(PPh_3)_2$ (62 mg, 0.088 mmol, 2 mol%), and ethynylaniline (0.47 mL, 4.2 mmol) at room temperature under a nitrogen atmosphere. The mixture was stirred for 48 h and quenched by the addition of saturated aqueous NH_4Cl (1 × 30

mL) and brine (1 × 40 mL), dried over $MgSO_4$, filtered, and concentrated in vacuo. The residue was purified by column chromatography to afford 2-[(2-bromophenyl)ethynyl]aniline (0.46 g, 95% yield) as a pale-yellow liquid.

A mixture of 2-[(2-bromophenyl)ethynyl]aniline (950 mg, 3.49 mmol), CuI (65 mg, 0.34 mmol, 4 mol%), $PdCl_2(PPh_3)_2$ (250 mg, 0.34 mmol, 2 mol%), piperidine (1.0 mL), and ethynylbenzene (1.14 mL, 10.47 mmol) was stirred at 80 °C for overnight under a nitrogen atmosphere. The mixture was quenched by the addition of a saturated aqueous solution of NH_4Cl (1 × 30 mL), at room temperature. The aqueous phase was extracted with EtOAc (3 × 30 mL) and the layers were separated. The combined organic layers were washed with brine (1 × 40 mL), dried over $MgSO_4$, filtered, and concentrated in vacuo. The residue was purified by column chromatography to 2-((2-(phenylethynyl)phenyl)ethynyl)aniline. Yield: 738 mg, 2.51 mmol, 72%.

To a solution of 2-((2-(phenylethynyl)phenyl)ethynyl)aniline (950 mg, 3.23 mmol) and pyridine (1.0 mL, 12.92 mmol) in 20 mL of dichloromethane was added 4-methylbenzene-1-sulfonyl chloride (800 mg, 4.19 mmol) at 0 °C. The reaction solution was warmed to room temperature. After the reaction was complete, as monitored by TLC, water was added, and the mixture was extracted with Et_2O (3 × 30 mL). The combined organic phase was washed with brine (1 × 40 mL)and dried over $MgSO_4$. The solvent was evaporated under reduced pressure, and the crude product was purified via column chromatography using hexane/ethyl acetate (15:1 v/v) as eluent. The desired compound was obtained as a yellow sticky liquid. Yield: 572 mg, 1.28 mmol, 56%. **^{1}H NMR** (400 MHz, $CDCl_3$) δ: 8.34–8.32 (m, 1H), 7.67–7.60 (m, 2H), 7.51–7.47 (m, 1H), 7.45–7.41 (m, 4H), 7.40–7.37 (m, 1H), 7.31–7.28 (m, 1H), 7.25–7.17 (m, 3H), 7.07–7.04 (m, 2H), 6.92–6.90 (m, 2H), 6.82 (d, *J* = 0.8 Hz, 1H), 2.05 (s, 3H); **^{13}C NMR** (101 MHz, $CDCl_3$) δ: 144.5, 139.5, 137.9, 134.7, 134.6, 132.3, 131.9, 131.5, 130.5, 129.6, 129.4, 128.5, 128.2, 127.4, 127.0, 124.9, 124.3,123.35, 123.27, 120.9, 116.4, 115.2, 93.1, 88.9, 21.4; **HRMS** (ES^+) m/z: $[M+H]^+$ calculated for $[C_{29}H_{22}NO_2S]^+$: 448.1377, found 448.1371; **IR ν_{max}** (cm^{-1}): 3076, 2911, 2260, 1595, 1444, 1373, 1243, 1172, 1088, 1019, 902, 764, 728, 654.

2.2 Synthesis of *N*-thiosuccinimide starting materials, 2a–m

General procedure c for the synthesis of **2a**–**m**.

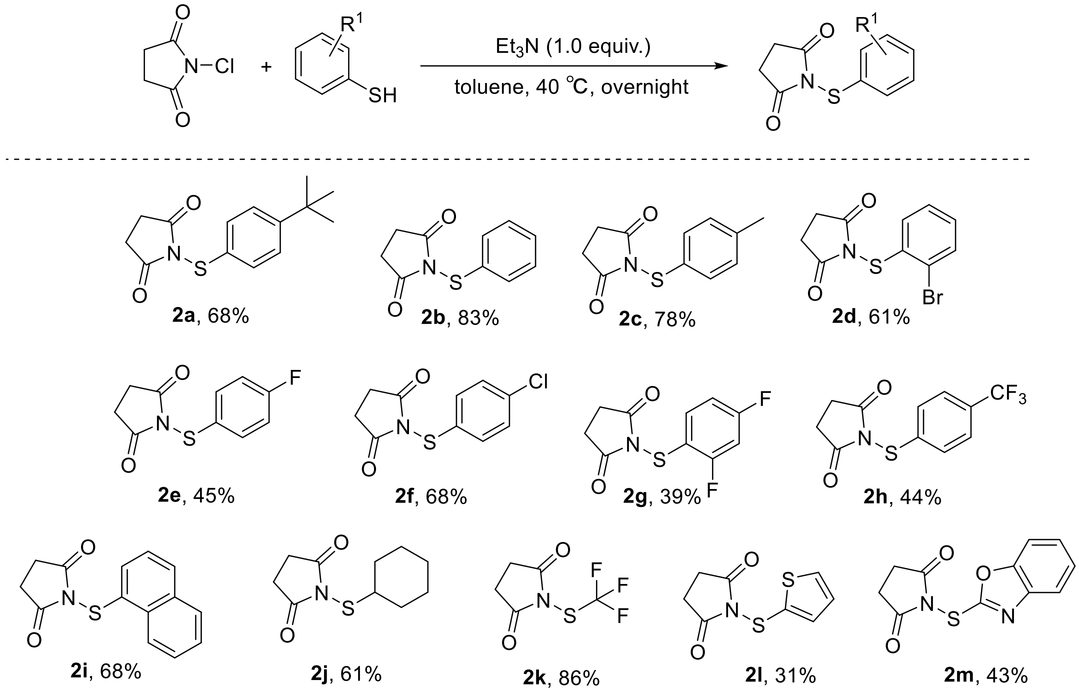

Scheme S6. Synthesis and structures of *N*-thiosuccinimides.

All the *N*-thiosuccinimide compounds (**2a–m**) were prepared using our previous literature procedure.[11] To a stirred solution of the corresponding thiol (7.53 mmol, 1.0 equiv.) in 20 mL of toluene, *N*-chlorosuccinimide (1.01 g, 7.53 mmol, 1 equiv.) was added to the reaction mixture at room temperature under a nitrogen atmosphere. After one hour, triethylamine (7.53 mmol, 1.0 equiv.) in dry toluene (10 mL) was added dropwise over a period of 30 minutes to the reaction mixture. The mixture was allowed to stir at 40 °C overnight. The reaction was diluted by the addition of 60 mL of diethyl ether. The resulting white precipitate was filtered. The filtrate was concentrated to afford the crude product. The pure compound was obtained after column chromatography using ethyl acetate and hexane (30:70) as eluent. The NMR data were in agreement with the previously reported compounds.[11]

2.3 Synthesis of aryl propargyl ethers/amides, and but-1-yne-1,4-diyldibenzene, 4a–i

General synthesis for propargyl ether reagents:

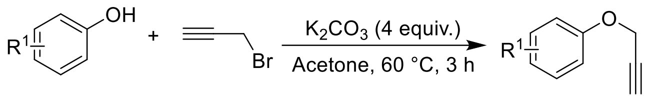

Scheme S7. Synthesis of aryl propargyl ethers.

Adapting a known procedure, all aryl propargyl ethers were prepared using the corresponding phenol (10.0 mmol, 1.0 equiv.) in acetone (20 mL), followed by the addition of K_2CO_3 (40.0 mmol, 4.0 equiv.). The suspension was stirred for 10 min, and then propargyl bromide (12.0 mmol, 1.2 equiv.) was added via a syringe at room temperature. The reaction mixture was stirred overnight at reflux temperature for 3 hours. The solvent was removed under reduced pressure, and the crude product was purified by flash column chromatography to give the desired product.

General procedure d for the synthesis of **4a-g**.

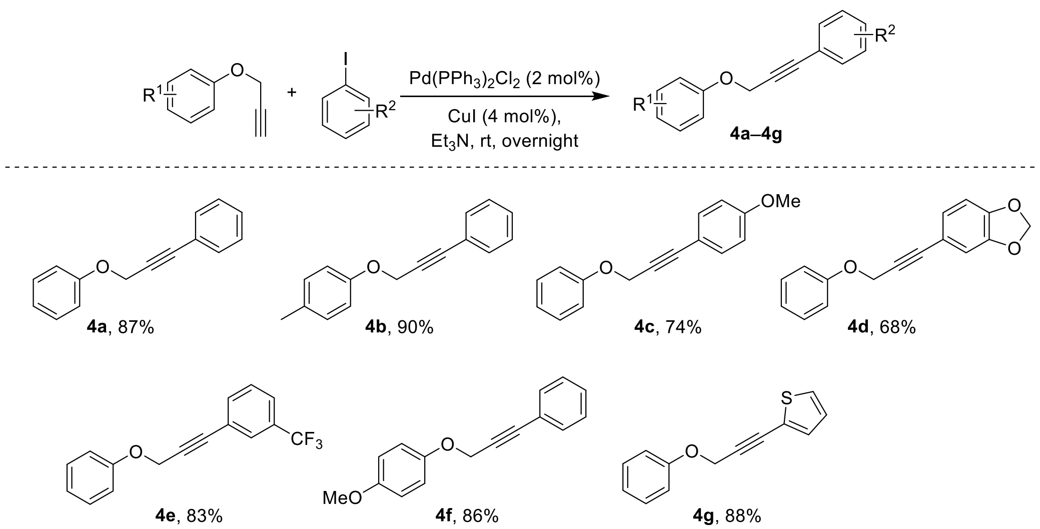

Scheme S8. Synthesis of aryl propargyl ethers.

To a dried Schlenk flask was added $Pd(PPh_3)_2Cl_2$ (2 mol%), CuI (4 mol%), iodobenzene (12.0 mmol, 1.2 equiv.), the propargyl ether (10.0 mmol, 1.0 equiv.) and freshly distilled Et_3N (20 mL) under a nitrogen atmosphere. The resulting mixture was stirred overnight at room temperature. The solvent was removed under reduced pressure, and the crude product was purified by flash column chromatography to give the desired product. The spectroscopic data of the starting materials are given below.

Synthesis of (3-phenoxyprop-1-yn-1-yl)benzene, **4a**[12]

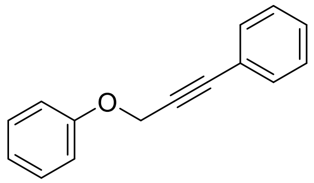

Synthesised in accordance with *General Procedure d*. CuI (57 mg, 0.30 mmol, 4 mol%), $PdCl_2(PPh_3)_2$ (106 mg, 0.15 mmol, 2 mol%) and iodobenzene (1.01 mL, 9.07 mmol, 1.2 equiv.) were added to a stirred solution of (prop-2-yn-1-yloxy) benzene (0.97 mL, 7.56 mmol, 1.0 equiv.) in 20 mL of Et_3N at room temperature. The crude product was purified via column chromatography using hexane/ethyl acetate (5:1 v/v) as eluent. The desired compound **4a** was obtained as a white solid. Yield: 1.36 g, 6.57 mmol, 87%. **^{1}H NMR** (400 MHz, $CDCl_3$) δ: 7.48–7.44 (m, 2H), 7.37–7.30 (m, 5H), 7.08–7.00 (m, 3H), 4.93 (s, 2H); **^{13}C NMR** (101 MHz, $CDCl_3$) δ: 157.1, 131.9, 129.6, 128.8, 128.4, 122.4, 121.6, 115.1, 87.3, 84.1, 56.8. The NMR data are in agreement with the previously reported literature values.

Synthesis of 1-methyl-4-((3-phenylprop-2-yn-1-yl)oxy)benzene, **4b**[13]

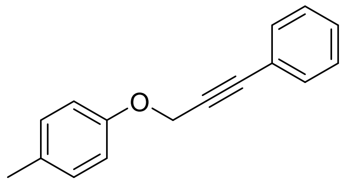

Synthesised in accordance with *General Procedure d*. CuI (52 mg, 0.27 mmol, 4 mol%), $PdCl_2(PPh_3)_2$ (96 mg, 0.13 mmol, 2 mol%) and iodobenzene (0.91 mL, 8.20 mmol, 1.2 equiv.) were added to a stirred solution of 1-methyl-4-(prop-2-yn-1-yloxy)benzene (1.00 g, 6.84 mmol, 1.0 equiv.) in 20 mL of Et_3N at room temperature. The crude product was purified via column chromatography using hexane/ethyl acetate (5:1 v/v) as eluent. The desired compound **4b** was obtained as a white solid. yield: 1.36 g, 6.15 mmol, 90%. **^{1}H NMR** (400 MHz, $CDCl_3$) δ: 7.45–7.42 (m, 2H), 7.32–7.28 (m, 3H), 7.13–7.10 (m, 2H), 6.95–6.92 (m, 2H), 4.88 (s, 2H), 2.30 (s, 3H); **^{13}C NMR** (101 MHz, $CDCl_3$) δ: 155.9, 131.9, 130.9, 130.0, 128.8, 128.4, 122.5, 115.0, 87.1, 84.3, 56.1, 20.7. The NMR data are in agreement with the previously reported literature values.

Synthesis of 1-methoxy-4-(3-phenoxyprop-1-yn-1-yl)benzene **4c**[14]

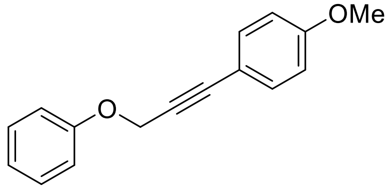

Synthesised in accordance with *General Procedure d*. CuI (58 mg, 0.30 mmol, 4 mol%), $PdCl_2(PPh_3)_2$ (106 mg, 0.15 mmol, 2 mol%) and 1-iodo-4-methoxybenzene (2.12 g, 9.07 mmol, 1.2 equiv.) were added to a stirred solution of (prop-2-yn-1-yloxy)benzene (0.96 mL, 7.56 mmol, 1.0 equiv.) in 20 mL of Et_3N at room temperature. The crude product was purified via column chromatography using hexane/ethyl acetate (5:1 v/v) as eluent.

The desired compound **4c** was obtained as a white solid. yield: 1.33 g, 5.59 mmol, 74%. **^{1}H NMR** (400 MHz, $CDCl_3$) δ: 7.40–7.36 (m, 2H), 7.34–7.29 (m, 2H), 7.06–6.97 (m, 3H), 6.85–6.81 (m, 2H), 4.90 (s, 2H), 3.81 (s, 3H); **^{13}C NMR** (101 MHz, $CDCl_3$) δ: 160.0, 158.0, 138.3, 133.5, 129.6, 121.5, 115.1, 114.0, 87.3, 82.7, 56.9, 55.4. The NMR data are in agreement with the previously reported literature values.

Synthesis of 5-(3-phenoxyprop-1-yn-1-yl)benzo[d][1,3]dioxole, **4d**[14]

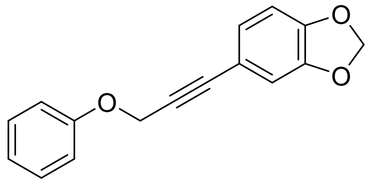

Synthesised in accordance with *General Procedure d*. CuI (58 mg, 0.30 mmol, 4 mol%), $PdCl_2(PPh_3)_2$ (106 mg, 0.15 mmol, 2 mol%) and 5-iodobenzo[d][1,3]dioxole (1.17 mL, 9.07 mmol, 1.2 equiv.) were added to a stirred solution of prop-2-yn-1-yloxy)benzene (0.97 mL, 7.56 mmol, 1.0 equiv.) in 20 mL of Et_3N at room temperature. The crude product was purified via column chromatography using hexane/ethyl acetate (5:1 v/v) as eluent. The desired compound **4d** was obtained as a white solid. yield: 1.29 g, 4.93 mmol, 68%.**^{1}H NMR** (400 MHz, $CDCl_3$) δ: 7.34–7.29 (m, 2H), 7.05–6.95 (m, 4H), 6.88 (d, J = 1.6 Hz, 1H), 6.74 (d, J = 8.0 Hz, 1H), 5.96 (s, 2H), 4.89 (s, 2H); **^{13}C NMR** (101 MHz, $CDCl_3$) δ: 157.1, 148.3, 147.5, 129.6, 126.8, 121.5, 115.6, 115.1, 111.9, 108.5, 101.5, 87.2, 82.5, 56.8. The NMR data are in agreement with the previously reported literature values.

Synthesis of 1-((3-phenylprop-2-yn-1-yl)oxy)-3-(trifluoromethyl)benzene, **4e**[15]

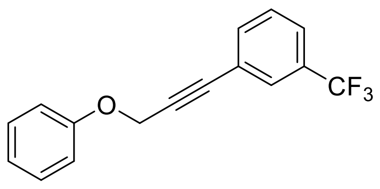

Synthesised in accordance with *General Procedure d*. CuI (58 mg, 0.30 mmol, 4 mol%), $PdCl_2(PPh_3)_2$ (108 mg, 0.15 mmol, 2 mol%) and 1-iodo-3-(trifluoromethyl)benzene (2.46 g, 9.07 mmol, 1.2 equiv.) were added to a stirred solution of (prop-2-yn-1-yloxy)benzene (0.97 mL, 7.56 mmol, 1.0 equiv.) in 20 mL of Et_3N at room temperature. The crude product was purified via column chromatography using hexane/ethyl acetate (5:1 v/v) as eluent. The desired compound **4e** was obtained as yellow oil. yield: 1.73 g, 6.27 mmol, 83%. **^{1}H NMR** (400 MHz, $CDCl_3$) δ: 7.70 (s, 1H), 7.63–7.55 (m, 2H), 7.46–7.41 (m, 1H), 7.36–7.31 (m, 2H), 7.06–7.00 (m, 3H), 4.93 (s, 2H); **^{13}C NMR** (101 MHz, $CDCl_3$) δ: 157.8, 135.0, 131.3, 130.9, 129.7, 128.8 (q, J = 12.0 Hz), 125.4 (q, J = 12.0 Hz), 125.1, 123.4, 121.8, 115.1, 85.7, 85.7, 56.6; **^{19}F NMR** (376 MHz, $CDCl_3$) δ: -63.00. The NMR data are in agreement with the previously reported literature values.

Synthesis of 1-methoxy-4-((3-phenylprop-2-yn-1-yl)oxy)benzene, **4f**[16]

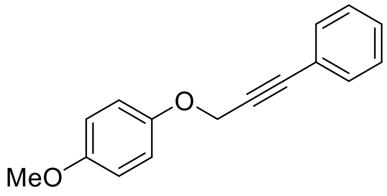

Synthesised in accordance with General Procedure d. CuI (46 mg, 0.24 mmol, 4 mol%), $PdCl_2(PPh_3)_2$ (86 mg, 0.12 mmol, 2 mol%) and iodo benzene (0.82 mL, 7.39 mmol, 1.2 equiv.) were added to a stirred solution of 1-methoxy-4-(prop-2-yn-1-yloxy)benzene (1.00 g, 6.16 mmol, 1.0 equiv.) in 20 mL of Et_3N at room temperature. The crude product was purified via column chromatography using hexane/ethyl acetate (5:1 v/v) as eluent. The desired compound **4f** was obtained as a white solid. yield: 1.27 g, 5.33 mmol, 86%. **^{1}H NMR (**400 MHz, $CDCl_3$) δ: 7.46–7.43 (m, 2H), 7.32–7.28 (m, 3H), 7.01–6.97 (m, 2H), 6.89–6.84 (m, 2H), 4.86 (s, 2H), 3.78 (s, 3H); **^{13}C NMR** (101 MHz, $CDCl_3$) δ: 154.5, 152.1, 131.9, 128.7, 128.4, 122.5, 116.4, 114.7, 87.1, 84.4, 57.6, 55.8. The NMR data are in agreement with the previously reported literature values.

Synthesis of 2-(3-phenoxyprop-1-yn-1-yl)thiophene, **4g**[17]

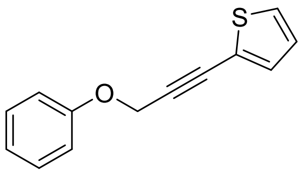

Synthesised in accordance with *General Procedure d*. CuI (58 mg, 0.30 mmol, 4 mol%), $PdCl_2(PPh_3)_2$ (108 mg, 0.15 mmol, 2 mol%) and 2-iodothiophene (1.90 g, 9.07 mmol, 1.2 equiv.) were added to a stirred solution of (prop-2-yn-1-yloxy)benzene (0.97 mL, 7.56 mmol, 1.0 equiv.) in 20 mL of Et_3N at room temperature. The crude product was purified via column chromatography using hexane/ethyl acetate (5:1 v/v) as eluent. The desired compound **4g** was obtained as a white solid. yield: 1.42 g, 6.62 mmol, 88%. **^{1}H NMR** (400 MHz, $CDCl_3$) δ: 7.35–7.29 (m, 2H), 7.27–7.26 (m, 1H), 7.23 (dd, J = 3.7, 1.3 Hz, 1H), 7.04–7.00 (m, 3H), 6.99–6.95 (m, 1H), 4.92 (s, 2H); **^{13}C NMR** (101 MHz, $CDCl_3$) δ 157.9, 132.9, 129.6, 127.8, 127.1, 122.3, 121.7, 115.1, 88.1, 80.6, 56.8. The NMR data are in agreement with the previously reported literature values.

Synthesis of but-1-yne-1,4-diyldibenzene, **4h**[18]

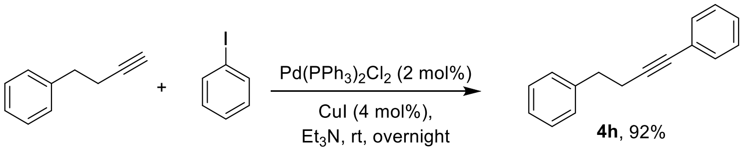

Scheme S9. Synthesis of but-1-yne-1,4-diyldibenzene.

CuI (60 mg, 0.30 mmol, 4 mol%), $PdCl_2(PPh_3)_2$ (100 mg, 0.15 mmol, 2 mol%) and iodobenzene (1.03 mL, 9.21 mmol, 1.2 equiv.) were added to a stirred solution of but-3-yn-1-yl benzene (7.10 mL, 7.68 mmol, 1.0 equiv.) in 20 mL of Et_3N at room temperature. The crude product was purified via column chromatography using hexane/ethyl acetate (5:1 v/v) as eluent. The desired compound **4h** was obtained as a colourless oil. yield: 1.45 g, 7.06 mmol, 92%. **^{1}H NMR** (400 MHz, $CDCl_3$) δ: 7.42–7.37 (m, 2H), 7.37–7.28 (m, 7H), 7.28–7.23 (m, 1H), 2.96 (t, *J* = 7.5 Hz, 2H), 2.72 (t, *J* = 7.5 Hz, 2H); **^{13}C NMR** (101 MHz, $CDCl_3$) δ: 140.8, 131.7, 128.7, 128.5, 128.3, 127.8, 126.4, 124.0, 89.6, 81.5, 35.4, 21.8. The NMR data are in agreement with the previously reported literature values.

Synthesis of 4-methyl-N-phenyl-N-(3-phenylprop-2-yn-1-yl)benzenesulfonamide, **4i**[19]

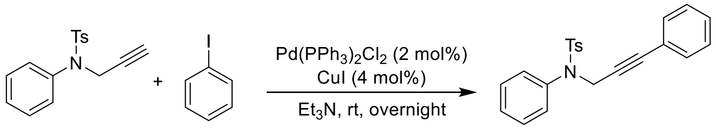

4i, 74%

Scheme S10. Synthesis of 4-methyl-*N*-phenyl-*N*-(3-phenylprop-2-yn-1-yl)benzenesulfonamide.

To a dried Schlenk flask was added $Pd(PPh_3)_2Cl_2$ (50 mg, 0.07 mmol, 2 mol%), CuI (27 mg, 0.14 mmol, 4 mol%), iodobenzene (0.46 mL, 4.20 mmol, 1.2 equiv.), 4-methyl-*N*-phenyl-*N*-(prop-2-yn-1-yl)benzenesulfonamide (1.00 g, 3.50 mmol, 1.0 equiv.) and freshly distilled Et_3N (20 mL) under a nitrogen atmosphere. The resulting mixture was stirred overnight at room temperature. The solvent was removed under reduced pressure. The crude product was purified via column chromatography using hexane/ethyl acetate (10:1 v/v) as eluent. The desired compound **4i** was obtained as a white solid. yield: 936 mg, 2.59 mmol, 74%. **^{1}H NMR** (400 MHz, $CDCl_3$) δ: 7.61–7.57 (m, 2H), 7.34–7.31 (m, 5H), 7.31–7.26 (m, 2H), 7.26–7.23 (m, 1H), 7.20–7.15 (m, 4H), 4.67 (s, 2H), 2.36 (s, 3H); **^{13}C NMR** (101 MHz, $CDCl_3$) δ: 143.6, 140.0, 139.9, 131.6, 129.4, 129.2,128.7, 128.6, 128.33, 128.27, 128.0, 122.5, 85.8, 83.8, 42.2, 21.7. The NMR data are in agreement with the previously reported literature values.

3. Synthesis and characterisation of products

3.1 Synthesis of 2-phenyl-1-tosyl-1*H*-indoles and 2-phenylbenzofurans, 3aa–3kk

General procedure e for the synthesis of 2-phenyl-1-tosyl-1*H*-indoles or 2-phenylbenzofurans, **3aa–3kk**

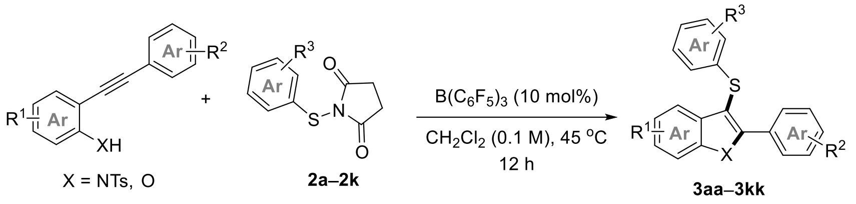

Scheme S11. Synthesis of 2-phenyl-1-tosyl-1*H*-indoles or 2-phenylbenzofurans.

In the glovebox, a glass microwave vial was charged with the protected 2-alkynyl aniline or phenol **1** (0.1 mmol, 1.0 equiv.), and 0.5 mL of dry CH_2Cl_2 was added to another vial equipped *N*-thiosuccinimide **2** (0.1 mmol, 1.0 equiv.) plus $B(C_6F_5)_3$ (0.01 mmol, 10 mol%) with a magnetic stirrer. All reactions were carried out at 45 °C for 12 h. After the reaction time, the CH_2Cl_2 was removed in vacuo, and the crude compound was purified via preparative thin-layer chromatography using hexane/ethyl acetate as eluent to give the pure product.

Synthesis of 3-((4-(tert-butyl)phenyl)thio)-2-phenyl-1-tosyl-1H-indole, **3aa**[18]

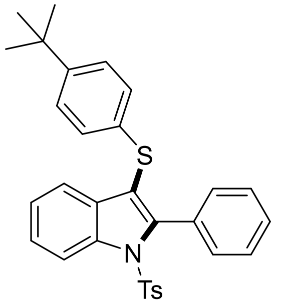

Synthesised in accordance with *General Procedure e* using 4-methyl-*N*-(2-(phenylethynyl)phenyl)benzenesulfonamide (**1a**) (35 mg, 0.1 mmol), 1-((4-(*tert*-butyl)phenyl)thio)pyrrolidine-2,5-dione (**2a**) (27 mg, 0.1 mmol), and $B(C_6F_5)_3$ (5.11 mg, 0.01 mmol). The desired product **3aa** was obtained as a colourless liquid. R_f = 0.40 (10% ethyl acetate in hexane). Yield: 45 mg, 0.087 mmol, 88%. **^{1}H NMR** (400 MHz, $CDCl_3$) δ: 8.42–8.39 (m, 1H), 7.47–7.40 (m, 7H), 7.33 (dd, *J* = 8.5, 1.9 Hz, 2H), 7.29–7.20 (m, 1H), 7.13–7.08 (m, 4H), 6.77–6.74 (m, 2H), 2.37 (s, 3H), 1.25 (s, 9H); **^{13}C NMR** (101 MHz, $CDCl_3$) δ: 148.6, 145.0, 144.1, 137.6, 134.1, 133.1, 131.7, 131.5, 130.3, 129.5, 129.3, 127.3, 127.0, 126.6, 125.9, 125.8, 124.8, 120.5, 116.6, 114.3, 34.4, 31.4, 21.7. The NMR data are in agreement with the previously reported literature values.

Synthesis of 3-((4-(tert-butyl)phenyl)thio)-2-(p-tolyl)-1-tosyl-1H-indole, **3ba**

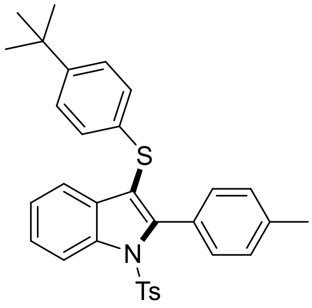

Synthesised in accordance with *General Procedure e* using 4-methyl-*N*-(2-(*p*-tolylethynyl)phenyl)benzenesulfonamide (**1b**) (37 mg, 0.1 mmol), 1-((4-(*tert*-butyl)phenyl)thio)pyrrolidine-2,5-dione (**2a**) (27 mg, 0.1 mmol), and $B(C_6F_5)_3$ (5.11 mg, 0.01 mmol). The desired product **3ba** was obtained as a colourless liquid. R_f = 0.5 (10% ethyl acetate in hexane). Yield: 48 mg, 0.090 mmol, 91%. **^{1}H NMR** (400 MHz, $CDCl_3$) δ: 8.41–8.38 (m, 1H), 7.44–7.39 (m, 2H), 7.36–7.27 (m, 5H), 7.24–7.21 (m, 2H), 7.13–7.08 (m, 4H), 6.76–6.73 (m, 2H), 2.44 (s, 3H), 2.37 (s, 3H), 1.25 (s, 9H); **^{13}C NMR** (101 MHz, $CDCl_3$) δ: 148.5, 145.4, 144.9, 139.3, 137.7, 134.1, 133.2, 131.6, 131.5, 129.5, 128.1, 127.4, 127.0, 126.4, 125.9, 125.7, 124.8, 120.4, 116.7, 114.0, 34.4, 31.4, 21.74, 21.70; **HRMS** (ES^+) m/z: $[M+H]^+$ calculated for $[C_{32}H_{32}NO_2S_2]^+$:526.1867, found 526.1874; **IR** $\mathbf{\nu_{max}}$ (cm^{-1}): 2963, 2238, 1593, 1492, 1444, 1371, 1187, 1175, 1097, 1019, 903, 821, 719, 667.

Synthesis of 2-([1,1'-biphenyl]-4-yl)-3-((4-(tert-butyl)phenyl)thio)-1-tosyl-1H-indole, **3ca**

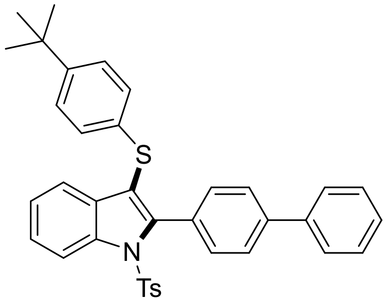

Synthesised in accordance with *General Procedure e* using ([1,1'-biphenyl]-4-ylethynyl)phenyl)-4-methylbenzenesulfonamide (**1c**) (43 mg, 0.1 mmol), 1-((4-(*tert*-butyl)phenyl)thio)pyrrolidine-2,5-dione (**2a**) (27 mg, 0.1 mmol), and $B(C_6F_5)_3$ (5.11 mg, 0.01 mmol). The desired product **3ca** was obtained as a white solid. R_f = 0.40 (10% ethyl acetate in hexane). Yield: 49 mg, 0.083 mmol, 83%.**^{1}H NMR** (400 MHz, $CDCl_3$) δ: 8.44–8.41 (m, 1H), 7.71–7.64 (m, 4H), 7.50–7.44 (m, 6H), 7.42–7.34 (m, 3H), 7.29 (td, J = 7.4, 1.0 Hz, 1H), 7.14–7.09 (m, 4H), 6.80–6.75 (m, 2H), 2.37 (s, 3H), 1.26 (s, 9H); **^{13}C NMR** (101 MHz, $CDCl_3$) δ: 148.6, 145.0, 144.9, 141.9, 140.6, 137.8, 134.9, 133.1, 132.1, 131.7, 129.7, 129.5, 129.2, 128.9, 127.7, 127.3, 127.1, 126.5, 125.95, 125.89, 124.9, 120.5, 116.7, 114.5, 34.43, 31.36, 21.7; **HRMS** (ES^+) m/z: $[M+H]^+$ calculated for $[C_{37}H_{34}NO_2S_2]^+$: 588.2034, found 588.2031; **IR** $\mathbf{\nu_{max}}$ (cm^{-1}): 2970, 2264, 1593, 1481, 1442, 1371, 1175, 1172, 1097, 1086, 1006, 905, 842, 818, 717, 693.

Synthesis of 3-((4-(tert-butyl)phenyl)thio)-2-(phenanthren-9-yl)-1-tosyl-1H-indole, **3da**

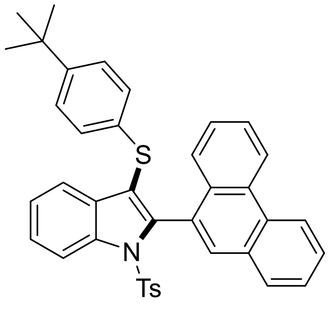

Synthesised in accordance with *General Procedure e* using 4-methyl-*N*-(2-(phenanthren-9-ylethynyl)phenyl)benzenesulfonamide (**1d**) (45 mg, 0.1 mmol), 1-((4-(*tert*-butyl)phenyl)thio)pyrrolidine-2,5-dione (**2a**) (27 mg, 0.1 mmol), and $B(C_6F_5)_3$ (5.11 mg, 0.01 mmol). The desired product **3da** was obtained as a white solid. R_f = 0.40 (20% ethyl acetate in hexane). Yield: 34 mg, 0.055 mmol, 55%. **^{1}H NMR** (400 MHz, $CDCl_3$) δ: 8.75–8.69 (m, 2H), 8.50 (dt, *J* = 8.5, 0.9 Hz, 1H), 7.76–7.71 (m, 2H), 7.65–7.59 (m, 3H), 7.52–7.46 (m, 2H), 7.40 (s, 1H), 7.38–7.34 (m, 2H), 7.30–7.26 (m, 2H), 7.05–7.02 (m, 2H), 6.98–6.96 (m, 2H), 6.87–6.84 (m, 2H), 2.31 (s, 3H), 1.21 (s, 9H); **^{13}C NMR** (101 MHz, $CDCl_3$) δ: 149.0, 145.0, 141.7, 137.3, 135.6, 132.5, 132.4, 131.6, 131.2, 130.9, 130.7, 129.9, 129.4, 129.2, 128.1, 127.7, 127.3, 127.1, 126.9, 126.8, 126.7, 126.6, 125.82, 125.79, 124.3, 122.9, 122.7, 120.7, 115.7, 115.7, 34.4, 31.3, 21.7; **HRMS** (ES^+) m/z: $[M+H]^+$ calculated for $[C_{39}H_{32}NO_2S_2]^+$: 610.1852, found 610.1874; **IR ν_{max}** (cm^{-1}): 3082, 2952, 2877, 2240, 1593, 1490, 1448, 1337, 1281, 1187, 1183, 1172, 1090, 950, 814, 723, 665.

Synthesis of 4-(3-((4-(tert-butyl)phenyl)thio)-1-tosyl-1H-indol-2-yl)-N,N-dimethylaniline, **3ea**

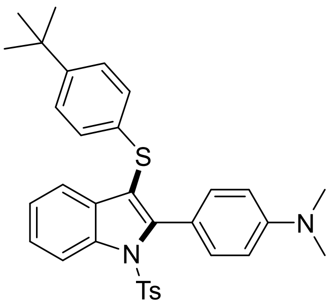

Synthesised in accordance with *general procedure e* using *N*-(2-((4-(dimethylamino)phenyl)ethynyl)phenyl)-4-methylbenzenesulfonamide (**1e**) (39 mg, 0.1 mmol), 1-((4-(*tert*-butyl)phenyl)thio)pyrrolidine-2,5-dione (**2a**) (27 mg, 0.1 mmol), and $B(C_6F_5)_3$ (5.11 mg, 0.01 mmol). The desired product **3ea** was obtained as a colourless oil. R_f = 0.40 (20% ethyl acetate in hexane). Yield: 11 mg, 0.019 mmol, 20%. **^{1}H NMR** (400 MHz, $CDCl_3$) δ: 8.24–8.22 (m, 1H), 7.38–7.34 (m, 6H), 7.32–7.27 (m, 1H), 7.24–7.19 (m, 3H), 7.13 (d, *J* = 8.4 Hz, 1H), 7.09 (d, *J* = 2.1 Hz, 1H), 6.95–6.91 (m, 2H), 6.37 (d, *J* = 0.8 Hz, 1H), 2.91 (s, 6H), 2.25 (s, 3H), 1.31 (s, 9H); **^{13}C NMR** (101 MHz, $CDCl_3$) δ: 152.9, 150.7, 144.4, 141.8, 138.2, 134.6, 132.4, 131.9, 131.4, 130.7, 129.8, 129.3, 126.9, 126.5, 124.7, 124.4, 120.6, 118.2, 116.7, 113.2, 44.6, 34.7, 31.4, 21.6; **HRMS** (ES^+) m/z: $[M+H]^+$ calculated for $[C_{33}H_{35}N_2O_2S_2]^+$: 555.2146, found 555.2140; **IR ν_{max}** (cm^{-1}): 2789, 2355, 2012, 1984, 1977, 1653, 1576, 1431, 1334, 1168, 974, 907, 829, 732, 698, 667.

Synthesis of 3-((4-(tert-butyl)phenyl)thio)-2-(4-bromophenyl)-1-tosyl-1H-indole, **3fa**

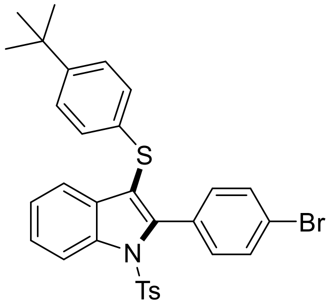

Synthesised in accordance with *General Procedure e* using *N*-(2-((4-bromophenyl)ethynyl)phenyl)-4-methylbenzenesulfonamide (**1f**) (43 mg, 0.1 mmol), 1-((4-(*tert*-butyl)phenyl)thio)pyrrolidine-2,5-dione (**2a**) (27 mg, 0.1 mmol), and $B(C_6F_5)_3$ (5.11 mg, 0.01 mmol). The desired product **3fa** was obtained as a white solid. R_f = 0.45 (10% ethyl acetate in hexane). Yield: 31 mg, 0.053 mmol, 53%. **^{1}H NMR** (400 MHz, $CDCl_3$) δ: 8.40–8.36 (m, 1H), 7.55–7.51 (m, 2H), 7.45–7.41 (m, 2H), 7.32–7.27 (m, 3H), 7.25–7.23 (m, 2H), 7.13–7.07 (m, 4H), 6.73–6.69 (m, 2H), 1.24 (s, 9H); **^{13}C NMR** (101 MHz, $CDCl_3$) δ: 148.8, 145.2, 143.7, 137.7, 134.9, 133.1, 132.8, 131.5, 130.6, 129.6, 129.2, 127.0, 126.6, 126.1, 126.0, 124.1, 123.9, 120.6, 116.7, 115.0, 34.5, 31.4, 21.8; **HRMS** (ES^+) m/z: $[M+H]^+$ calculated for $[C_{31}H_{28}NO_2S_2Br]^+$: 591.0735, found 591.0724; **IR ν_{max}** (cm^{-1}): 2946, 2898, 1591, 1479, 1442, 1373, 1170, 1174, 1093, 1086, 1010, 823, 754, 747, 663.

Synthesis of 3-((4-(tert-butyl)phenyl)thio)-1-tosyl-2-(4-(trifluoromethyl)phenyl)-1H-indole, **3ga**

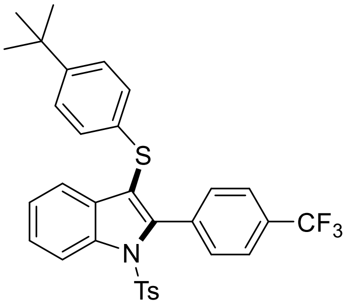

Synthesised in accordance with *General Procedure e* using 4-methyl *N*-(2-((4-(trifluoromethyl)phenyl)ethynyl)phenyl)benzenesulfonamide (**1g**) (42 mg, 0.1 mmol), 1-((4-(*tert*-butyl)phenyl)thio)pyrrolidine-2,5-dione (**2a**) (27 mg, 0.1 mmol), and $B(C_6F_5)_3$ (5.11 mg, 0.01 mmol). The desired product **3ga** was obtained as a white solid. R_f = 0.40 (10% ethyl acetate in hexane). Yield: 29 mg, 0.050 mmol, 50%. **^{1}H NMR** (400 MHz, $CDCl_3$) δ: 8.40–8.37 (m, 1H), 7.67–7.64 (m, 2H), 7.52–7.45 (m, 4H), 7.32–7.27 (m, 3H), 7.14–7.07 (m, 4H), 6.73–6.69 (m, 2H), 2.37 (s, 3H), 1.24 (s, 9H); **^{13}C NMR** (101 MHz, $CDCl_3$) δ: 148.1, 145.3, 143.1, 137.8, 134.7, 134.0, 132.6, 131.9, 131.2, 130.8, 129.7, 126.5 (q, *J* = 3.5 Hz), 125.0, 124.4, 124.34, 124.30, 124.26, 123.0, 120.7, 116.7, 115.9, 34.5, 31.4, 21.9; **^{19}F NMR** (376 MHz, $CDCl_3$) δ: -62.58; **HRMS** (ES^+) m/z: $[M+H]^+$ calculated for $[C_{32}H_{29}NO_2F_3S_2]^+$: 580.1590, found 580.1592; **IR ν_{max}** (cm^{-1}): 2966, 2862, 1615, 1599, 1494, 1444, 1375, 1321, 1181, 1174, 1125, 1066, 1015, 948, 905, 843, 760, 663.

Synthesis of 3-((4-(tert-butyl)phenyl)thio)-2-(cyclohex-1-en-1-yl)-1-tosyl-1H-indole, **3ha**

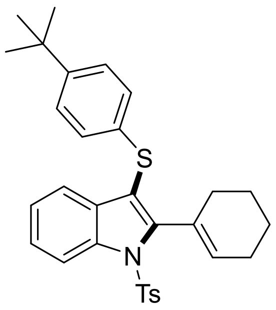

Synthesised in accordance with *General procedure e* using *N*-(2-(cyclohex-1-en-1-ylethynyl)phenyl)-4-methylbenzenesulfonamide (**1h**) (36 mg, 0.1 mmol), 1-((4-(*tert*-butyl)phenyl)thio)pyrrolidine-2,5-dione (**2a**) (27 mg, 0.1 mmol), and $B(C_6F_5)_3$ (5.11 mg, 0.01 mmol). The desired product was **3ha** obtained as a colourless liquid. R_f = 0.40 (20% ethyl acetate in hexane). Yield: 16 mg, 0.032 mmol, 32%. **^{1}H NMR** (400 MHz, $CDCl_3$) δ: 8.27–8.25 (m, 1H), 7.60–7.57 (m, 2H), 7.35–7.30 (m, 2H), 7.20–7.14 (m, 3H), 7.13–7.10 (m, 2H), 6.88–6.84 (m, 2H), 5.50–5.48 (m, 1H), 2.35 (s, 3H), 2.23–2.19 (m, 2H), 1.79–1.72 (m, 4H), 1.28–1.26 (m, 2H), 1.24 (s, 9H); **^{13}C NMR** (101 MHz, $CDCl_3$) δ: 148.5, 147.4, 144.9, 137.0, 135.6, 133.5, 132.5, 131.3, 131.2, 130.0, 129.5, 127.0, 126.8, 125.3, 124.3, 120.5, 115.8, 112.2, 34.5, 31.4, 30.7, 25.7, 22.7, 21.9, 21.7; **HRMS** (ES$^+$) m/z: $[M+H]^+$ calculated for $[C_{31}H_{34}NO_2S_2]^+$: 516.2034, found 516.2031; **IR ν_{max}** (cm^{-1}): 2935, 2871, 1593, 1490, 1443, 1375, 1116, 1095, 1021, 954, 851, 806, 747, 663.

Synthesis of 2-butyl-3-((4-(tert-butyl)phenyl)thio)-1-tosyl-1H-indole, **3ia**

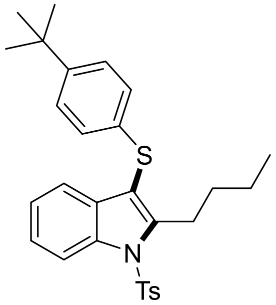

Synthesised in accordance with *General Procedure e* using *N*-(2-(hex-1-yn-1-yl)phenyl)-4-methylbenzenesulfonamide (33 mg, 0.1 mmol) (**1i**), 1-((4-(*tert*-butyl)phenyl)thio)pyrrolidine-2,5-dione (**2a**) (27 mg, 0.1 mmol), and $B(C_6F_5)_3$ (5.11 mg, 0.01 mmol). The desired product **3ia** was obtained as a white solid. R_f = 0.40 (20% ethyl acetate in hexane). Yield: 36 mg, 0.073 mmol, 73%. **^{1}H NMR** (400 MHz, $CDCl_3$) δ: 8.24–8.22 (m, 1H), 7.63 (dd, J = 8.5, 1.8 Hz, 2H), 7.44–7.40 (m, 1H), 7.34–7.28 (m, 1H), 7.23–7.19 (m, 3H), 7.15–7.10 (m, 2H), 6.85–6.81 (m, 2H), 3.24–3.19 (m, 2H), 2.37 (s, 3H), 1.71–1.61 (m, 2H), 1.40 (p, J = 7.3 Hz, 2H), 1.25 (s, 9H), 0.90 (td, J = 7.3, 1.5 Hz, 3H); **^{13}C NMR** (101 MHz, $CDCl_3$) δ: 148.5, 147.6, 145.0, 137.0, 136.0, 133.3, 131.3, 129.1, 126.5, 126.1, 125.1, 124.3, 124.1, 119.9, 115.5, 111.4, 34.4, 33.2, 31.4, 27.3, 22.9, 21.7, 13.9; **HRMS** (ES$^+$) m/z: $[M+H]^+$ calculated for $[C_{29}H_{34}NO_2S_2]^+$: 492.2026, found 492.2031; **IR ν_{max}** (cm^{-1}): 2968, 2860, 1593, 1492, 1448, 1375, 1189, 1112, 1080, 1056, 814, 749, 706, 656.

Synthesis of 3-((4-(tert-butyl)phenyl)thio)-1-tosyl-2-(trimethylsilyl)-1H-indole, **3ja**

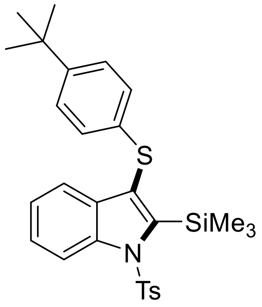

Synthesised in accordance with *General Procedure e* using 4-methyl-*N*-(2-((trimethylsilyl)ethynyl)phenyl)benzenesulfonamide (**1j**) (35 mg, 0.1 mmol), 1-((4-(*tert*-butyl)phenyl)thio)pyrrolidine-2,5-dione (**2a**) (27 mg, 0.1 mmol), and $B(C_6F_5)_3$ (5.11 mg, 0.01 mmol). The desired product **3ja** was obtained as a white solid. R_f = 0.40 (10% ethyl acetate in hexane). Yield: 20 mg, 0.040 mmol, 40%. **^{1}H NMR** (400 MHz, $CDCl_3$) δ: 8.40–8.38 (m, 1H), 7.75–7.70 (m, 3H), 7.43–7.38 (m, 1H), 7.30–7.26 (m, 1H), 7.14–7.11 (m, 2H), 7.07–7.05 (m, 2H), 6.80–6.77 (m, 2H), 2.29 (s, 3H), 1.25 (s, 9H), 0.34 (s, 9H); **^{13}C NMR** (101 MHz, $CDCl_3$) δ: 148.5, 144.5, 139.6, 136.5, 134.8, 133.6, 133.0, 130.9, 129.5, 127.4, 125.8, 125.7, 125.6, 123.2, 122.4, 115.8, 34.5, 31.5, 21.8, 1.2; **HRMS** (ES^+) m/z: $[M+H]^+$ calculated for $[C_{28}H_{34}NO_2SiS_2]^+$ 508.1800, found 508.1800; **IR ν_{max}** (cm^{-1}): 2955, 2898, 1595, 1490, 1435, 1379, 1220, 1175, 1090, 1011, 948, 816, 713, 669, 665.

Synthesis of 3-((4-(tert-butyl)phenyl)thio)-2-phenylbenzofuran, **3ka**

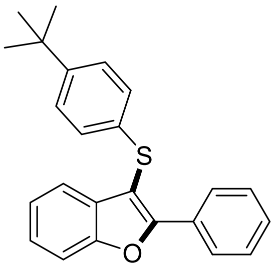

Synthesised in accordance with *General Procedure e* using 2-(phenylethynyl)phenol (**1k**) (19.5 mg, 0.1 mmol), 1-((4-(*tert*-butyl)phenyl)thio)pyrrolidine-2,5-dione (**2a**) (27 mg, 0.1 mmol), and $B(C_6F_5)_3$ (5.11 mg, 0.01 mmol). The desired product **3ka** was obtained as a white oil. R_f= 0.5 (5% ethyl acetate in hexane). Yield: 32 mg, 0.090 mmol, 90%. **^{1}H NMR** (400 MHz, $CDCl_3$) δ: 8.31–8.27 (m, 2H), 7.59–7.53 (m, 2H), 7.50–7.45 (m, 2H), 7.43–7.39 (m, 1H), 7.35 (ddd, *J* = 8.3, 7.2, 1.4 Hz, 1H), 7.26–7.22 (m, 3H), 7.18–7.14 (m, 2H), 1.27 (s, 9H); **^{13}C NMR** (101 MHz, $CDCl_3$) δ: 157.5, 154.0, 148.8, 132.7, 131.1, 130.0, 129.5, 128.7, 127.5, 126.6, 126.3, 125.3, 123.5, 120.6, 111.4, 105.2, 34.5, 31.4; **HRMS** (ES^+) m/z: $[M+H]^+$ calculated for $[C_{24}H_{22}OS]^+$ 358.1396, found 358.1391; **IR ν_{max}** (cm^{-1}): 3073, 2948, 2871, 1589, 1556, 1481, 1451, 1440, 1394, 1364, 1250, 1202, 1198, 1088, 1067, 1011, 890, 820, 745, 736, 687, 674.

Synthesis of 3-((4-(tert-butyl)phenyl)thio)-5-methyl-2-phenylbenzofuran, **3la**

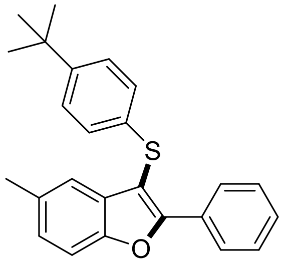

Synthesised in accordance with *General Procedure e* using 4-methyl-2-(phenylethynyl)phenol (**1l**) (21 mg, 0.1 mmol), 1-((4-(*tert*-butyl)phenyl)thio)pyrrolidine-2,5-dione (**2a**) (27 mg, 0.1 mmol), and $B(C_6F_5)_3$ (5.11 mg, 0.01 mmol). The desired product **3la** was obtained as a white oil. R_f = 0.45 (5% ethyl acetate in hexane). Yield: 32 mg, 0.085 mmol, 85%. **^{1}H NMR** (400 MHz, $CDCl_3$) δ: 8.28–8.24 (m, 2H), 7.48–7.43 (m, 3H), 7.42–7.33 (m, 2H), 7.26–7.23 (m, 2H), 7.17–7.13 (m, 3H), 2.42 (s, 3H), 1.27 (s, 9H); **^{13}C NMR** (101 MHz, $CDCl_3$) δ: 157.8, 152.5, 148.7, 133.2, 132.9, 131.2, 130.1, 129.4, 128.6, 127.5, 126.7, 126.31, 126.28, 120.3, 110.9, 104.7, 34.5, 31.4, 21.5; **HRMS** (ES^+) m/z: $[M+H]^+$ calculated for $[C_{25}H_{24}OS]^+$: 372.1556, found 372.1548; **IR ν_{max}** (cm^{-1}): 3076, 3032, 2950, 2871, 1600, 1477, 1442, 1394, 1364, 1272, 1194, 1067, 1011, 913, 823, 764, 687.

Synthesis of 3-((4-(tert-butyl)phenyl)thio)-5-chloro-2-phenylbenzofuran, **3ma**

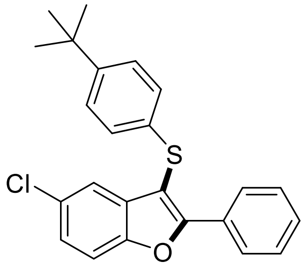

Synthesised in accordance with *General Procedure e* using 4-chloro-2-(phenylethynyl)phenol (**1m**) (23 mg, 0.1 mmol), 1-((4-(*tert*-butyl)phenyl)thio)pyrrolidine-2,5-dione (**2a**) (27 mg, 0.1 mmol), and $B(C_6F_5)_3$ (5.11 mg, 0.01 mmol). The desired product **3ma** was obtained as a colourless liquid. R_f = 0.45 (5% ethyl acetate in hexane). Yield: 29 mg, 0.073 mmol, 73%. **^{1}H NMR** (400 MHz, $CDCl_3$) δ: 8.27–8.24 (m, 2H), 7.49–7.42 (m, 5H), 7.29 (dd, J = 8.6, 2.2 Hz, 1H), 7.26–7.23 (m, 2H), 7.14–7.11 (m, 2H), 1.27 (s, 9H); **^{13}C NMR** (101 MHz, $CDCl_3$) δ: 158.1, 152.4, 149.2, 132.7, 132.2, 129.9, 129.6, 129.4, 128.8, 127.6, 126.6, 126.4, 125.6, 120.2, 112.5, 104.9, 34.6, 31.4; **HRMS** (ES^+) m/z: $[M+H]^+$ calculated for $[C_{24}H_{21}ClOS]^+$: 392.1001, found 392.1000; **IR ν_{max}** (cm^{-1}): 2968, 2875, 1602, 1470, 1466, 1444, 1360, 1246, 1192, 1159, 1011, 918, 905, 820, 728, 695, 689.

Synthesis of 3-((4-(tert-butyl)phenyl)thio)-5-fluoro-2-phenylbenzofuran, **3na**

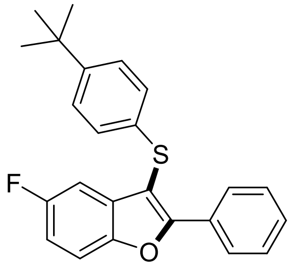

Synthesised in accordance with *General Procedure e* using 4-fluoro-2-(phenylethynyl)phenol (**1n**) (22 mg, 0.1 mmol), 1-((4-(*tert*-butyl)phenyl)thio)pyrrolidine-2,5-dione (**2a**) (27 mg, 0.1 mmol), and $B(C_6F_5)_3$ (5.11 mg, 0.01 mmol). The desired product **3na** was obtained as a colourless liquid. R_f = 0.45 (5% ethyl acetate in hexane). Yield: 26 mg,

0.069 mmol, 69%. **^{1}H NMR** (400 MHz, $CDCl_3$) δ: 8.27–8.24 (m, 2H), 7.49–7.41 (m, 4H), 7.26–7.23 (m, 2H), 7.18–7.11 (m, 3H), 7.04 (td, *J* = 9.0, 2.7 Hz, 1H), 1.26 (s, 9H); **^{13}C NMR** (101 MHz, $CDCl_3$) δ: 161.0, 159.3, 158.6, 150.2, 149.2, 132.5 (d, *J* = 11 Hz), 132.3 (d, *J* = 45 Hz), 129.8 (d, *J* = 4.5 Hz), 128.7, 128.6, 127.6, 126.7, 126.4, 113.1(d, *J*= 26.5 Hz), 112.2 (d, *J*= 8.9 Hz), 106.3 (d, *J* = 25.5 Hz), 34.5, 31.4; **^{19}F NMR** (376 MHz, $CDCl_3$) δ: -119.46; **HRMS** (ES^+) m/z: $[M+H]^+$ calculated for $[C_{24}H_{21}OFS]^+$: 376.1299, found 376.1297; **IR ν_{max}** (cm^{-1}): 2981, 2251, 1796, 1490, 1477, 1403, 1112, 1077, 900, 726, 652.

Synthesis of 3-((4-(tert-butyl)phenyl)thio)-2-(thiophen-3-yl)benzofuran, **3oa**

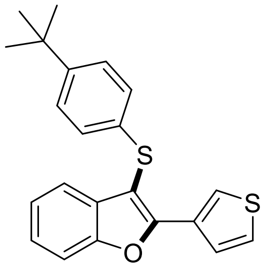

Synthesised in accordance with *General Procedure e* using 2-(thiophen-3-ylethynyl)phenol (**1o**) (21 mg, 0.1 mmol), 1-((4-(*tert*-butyl)phenyl)thio)pyrrolidine-2,5-dione (**2a**) (27 mg, 0.1 mmol), and $B(C_6F_5)_3$ (5.11 mg, 0.01 mmol). The desired product was **3oa** obtained as a yellow liquid. R_f = 0.45 (5% ethyl acetate in hexane). Yield: 33 mg, 0.090 mmol, 90%. **^{1}H NMR** (400 MHz, $CDCl_3$) δ: 8.21 (dd, *J* = 3.2, 1.5 Hz, 1H), 7.99–7.98 (m, 1H), 7.55–7.52 (m, 2H), 7.39 (dd, *J* = 5.1, 3.0 Hz, 1H), 7.36–7.31 (m, 1H), 7.25–7.21 (m, 3H), 7.17–7.13 (m, 2H), 1.26 (s, 9H); **^{13}C NMR** (101 MHz, $CDCl_3$) δ: 154.9, 153.9, 148.9, 132.6, 131.1, 130.9, 126.6, 126.5, 126.3, 126.0, 125.3, 125.2, 123.6, 120.4, 111.3, 104.0, 34.5, 31.4; **HRMS** (ES^+) m/z: $[M+H]^+$ calculated for $[C_{22}H_{20}OS_2]^+$: 364.0959, found 364.0956; **IR ν_{max}** (cm^{-1}): 3121, 2953, 2858, 1587, 1494, 1449, 1399, 1364, 1271, 1190, 1079, 1010, 864, 788, 764, 687.

Synthesis of 3-((4-(tert-butyl)phenyl)thio)-2-cyclopropylbenzofuran, **3pa**

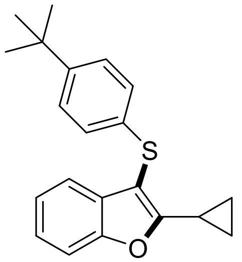

Synthesised in accordance with *General Procedure e* using 2-(cyclopropylethynyl)phenol (**1p**) (16 mg, 0.1 mmol), 1-((4-(*tert*-butyl)phenyl)thio)pyrrolidine-2,5-dione (**2a**) (27 mg, 0.1 mmol), and $B(C_6F_5)_3$ (5.11 mg, 0.01 mmol). The desired product **3pa** was obtained as a colourless liquid. R_f = 0.5 (2% ethyl acetate in hexane). Yield: 24 mg, 0.075 mmol, 75%. **^{1}H NMR** (400 MHz, $CDCl_3$) δ: 7.45–7.37 (m, 2H), 7.25–7.17 (m, 4H), 7.15–7.11 (m, 2H), 2.50–2.43 (m, 1H), 1.28 (s, 9H), 1.26–1.21 (m, 2H), 1.10–1.06 (m, 2H); **^{13}C NMR** (101 MHz, $CDCl_3$) δ: 164.0, 153.6, 148.5, 133.7, 130.5, 123.9, 123.3, 119.3, 110.1, 103.7, 34.5, 31.4, 8.6, 8.4; **HRMS** (ES^+) m/z: $[M+H]^+$ calculated for $[C_{21}H_{22}OS]^+$: 322.1396, found 322.1391; **IR ν_{max}** (cm^{-1}): 2950, 2871, 2858, 1595, 1580, 1494, 1436, 1395, 1248, 1175, 1049, 1011, 929, 741, 721, 676.

*Synthesis of 2-phenyl-3-(phenylthio)-1-tosyl-1H-*indole, **3ab**[20]

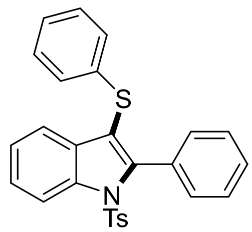

Synthesised in accordance with *General Procedure e* using 4-methyl-*N-(*2-(phenylethynyl)phenyl)benzenesulfonamide (**1a**) (35 mg, 0.1 mmol), 1-(phenylthio)pyrrolidine-2,5-dione (**2b**) (20 mg, 0.1 mmol), and $B(C_6F_5)_3$ (5.11 mg, 0.01 mmol). The desired product **3ab** was obtained as a yellow liquid. R_f = 0.40 (15% ethyl acetate in hexane). Yield: 32 mg, 0.070 mmol, 70%. **^{1}H NMR** (400 MHz, $CDCl_3$) δ: 8.42–8.39 (m, 1H), 7.47–7.34 (m, 7H), 7.34–7.30 (m, 2H), 7.29–7.24 (m, 1H), 7.13–7.05 (m, 5H), 6.84–6.81 (m, 2H), 2.36 (s, 3H); **^{13}C NMR** (101 MHz, $CDCl_3$) δ: 145.2, 145.1, 137.6, 136.9, 135.0, 131.6, 131.3, 130.2, 129.5, 129.4, 128.9, 127.4, 127.0, 126.6, 125.9, 125.4, 124.8, 120.4, 116.6, 113.8, 21.7. The NMR data are in agreement with the previously reported literature values.

*Synthesis of 2-phenyl-3-(p-tolylthio)-1-tosyl-1H-*indole, **3ac**[21]

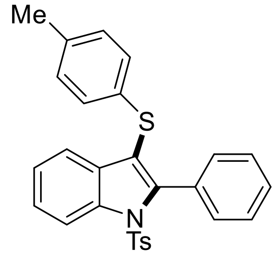

Synthesised in accordance with *General Procedure e* using 4-methyl-*N*-(2-(phenylethynyl)phenyl)benzenesulfonamide (**1a**) (35 mg, 0.1 mmol), 1-(*p*-tolylthio)pyrrolidine-2,5-dione (**2c**) (23 mg, 0.1 mmol), and $B(C_6F_5)_3$ (5.11 mg, 0.01 mmol). The desired product **3ac** was obtained as a white solid. R_f = 0.40 (15% ethyl acetate in hexane). Yield: 35 mg, 0.075 mmol, 75%. **^{1}H NMR** (400 MHz, $CDCl_3$) δ: 8.40–8.36 (m, 1H), 7.49–7.42 (m, 2H), 7.41–7.35 (m, 5H), 7.33–7.29 (m, 2H), 7.27–7.23 (m, 1H), 7.12–7.08 (m, 2H), 6.90–6.87 (m, 2H), 6.75–6.71 (m, 2H), 2.35 (s, 3H), 2.23 (s, 3H); **^{13}C NMR** (101 MHz, $CDCl_3$) δ: 145.0, 144.9, 137.6, 135.4, 135.0, 133.1, 131.7, 131.4, 130.3, 129.7, 129.5, 129.3, 127.3, 127.1, 127.0, 125.8, 124.8, 120.4, 116.6, 114.4, 21.7, 21.0. The NMR data are in agreement with the previously reported literature values.

Synthesis of 3-((2-bromophenyl)thio)-2-phenyl-1-tosyl-1H-indole, **3ad**

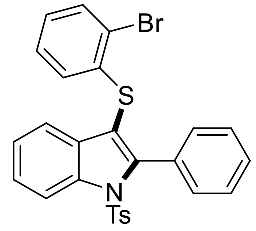

Synthesised in accordance with *General Procedure e* using 4-methyl-*N*-(2-(phenylethynyl)phenyl)benzenesulfonamide (**1a**) (35 mg, 0.1 mmol), 1-((2-bromophenyl)thio)pyrrolidine-2,5-dione (**2d**) (29 mg, 0.1 mmol), and $B(C_6F_5)_3$ (5.11 mg, 0.01 mmol). The desired product **3ad** was obtained as a white solid. R_f = 0.45 (15% ethyl acetate in hexane). Yield: 32 mg, 0.059 mmol, 59%. **^{1}H NMR** (400 MHz, $CDCl_3$) δ: 8.42–8.40 (m, 1H), 7.46–7.43 (m, 2H), 7.42–7.37 (m, 3H), 7.35–7.26 (m, 6H), 7.12–7.10 (m, 2H), 6.92–6.83 (m, 2H), 6.26 (dd, J = 7.8, 1.7 Hz, 1H),

2.35 (s, 3H); **^{13}C NMR** (101 MHz, $CDCl_3$) δ: 146.0, 145.2, 138.2, 137.7, 135.0, 132.9, 131.5, 130.9, 129.9, 129.6, 127.6, 129.57, 129.56, 127.1, 126.8, 126.3, 126.1, 124.1, 120.8, 120.3, 116.7, 113.1, 21.8; **HRMS** (ES^+) m/z: $[M+H]^+$ calculated for $[C_{27}H_{21}NO_2S_2Br]^+$: 536.0178, found 536.0177; **IR ν_{max}** (cm^{-1}): 3075, 2909, 1599, 1444, 1375, 1175, 1088, 1023, 1021, 937, 751, 661.

Synthesis of 3-((4-fluorophenyl)thio)-2-phenyl-1-tosyl-1H-indole, **3ae**[21]

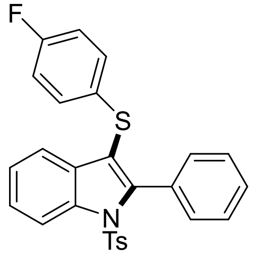

Synthesised in accordance with *General Procedure e* using 4-methyl-*N*-(2-(phenylethynyl)phenyl)benzenesulfonamide (**1a**) (35 mg, 0.1 mmol), *N*-((4-fluorophenyl)thio)succinimide (**2e**) (23 mg, 0.1 mmol), and $B(C_6F_5)_3$ (5.11 mg, 0.01 mmol). The desired product **3ae** was obtained as a white solid. R_f = 0.45 (15% ethyl acetate in hexane). Yield: 35 mg, 0.074 mmol, 74%. **^{1}H NMR** (400 MHz, $CDCl_3$) δ: 8.41–8.38 (m, 1H), 7.50–7.45 (m, 1H), 7.43–7.40 (m, 4H), 7.36–7.28 (m, 5H), 7.11 (dd, *J* = 8.7, 0.7 Hz, 2H), 6.85–6.78 (m, 3H), 6.79–6.76 (m, 1H), 2.36 (s, 3H); **^{13}C NMR** (101 MHz, $CDCl_3$) δ: 161.2 (d, *J* = 245.0 Hz), 145.1, 144.9, 137.5, 135.1, 131.6, 131.0, 130.2, 129.6, 129.4, 129.0 (d, *J* = 8.0 Hz), 127.4, 127.1, 125.0, 124.8, 120.2, 116.7, 116.2, 115.9, 114.2, 21.8; **^{19}F NMR** (376 MHz, $CDCl_3$) δ -116.73. The NMR data are in agreement with the previously reported literature values.

*Synthesis of 3-((4-chlorophenyl)thio)-2-phenyl-1-tosyl-1H-*indole, **3af**[21]

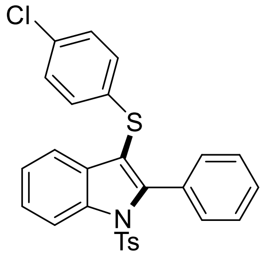

Synthesised in accordance with *General Procedure e* using 4-methyl-*N*-(2-(phenylethynyl)phenyl)benzenesulfonamide (**1a**) (35 mg, 0.1 mmol), 1-((4-chlorophenyl)thio)pyrrolidine-2,5-dione (**2f**) (25 mg, 0.1 mmol), and $B(C_6F_5)_3$ (5.11 mg, 0.01 mmol). The desired product **3af** was obtained as a white solid. R_f = 0.40 (15% ethyl acetate in hexane). Yield: 30 mg, 0.061 mmol, 61%. **^{1}H NMR** (400 MHz, $CDCl_3$) δ: 8.42–8.39 (m, 1H), 7.49–7.38 (m, 5H), 7.34–7.26 (m, 5H), 7.13–7.09 (m, 2H), 7.05–7.02 (m, 2H), 6.76–6.72 (m, 2H), 2.36 (s, 3H); **^{13}C NMR** (101 MHz, $CDCl_3$) δ: 145.4, 145.2, 137.6, 135.5, 135.0, 131.5, 131.3, 130.9, 130.0, 129.6, 129.5, 129.0, 127.9, 127.4, 127.1, 126.0, 124.9, 120.2, 116.6, 113.2, 21.8. The NMR data are in agreement with the previously reported literature values.

Synthesis of 3-((2,4-difluorophenyl)thio)-2-phenyl-1-tosyl-1H-indole, **3ag**

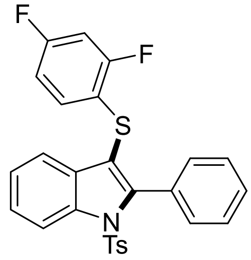

Synthesised in accordance with *General Procedure e* using 4-methyl-*N*-(2-(phenylethynyl)phenyl)benzenesulfonamide (**1a**) (35 mg, 0.1 mmol), 1-((2,4-difluorophenyl)thio)pyrrolidine-2,5-dione (**2g**) (25 mg, 0.1 mmol), and $B(C_6F_5)_3$ (5.11 mg, 0.01 mmol). The desired product **3ag** was obtained as a yellow liquid. R_f = 0.40 (15% ethyl acetate in hexane). Yield: 24 mg, 0.048 mmol, 48%. **^{1}H NMR** (400 MHz, $CDCl_3$) δ: 8.40–8.37 (m, 1H), 7.50–7.40 (m, 5H), 7.34–7.26 (m, 5H), 7.12–7.09 (m, 2H), 6.74–6.69 (m, 1H), 6.56–6.48 (m, 2H), 2.35 (s, 3H); **^{13}C NMR** (101 MHz, $CDCl_3$) δ: 162.9 (d, *J* = 11.3 Hz), 161.1 (d, *J* = 11.8 Hz), 160.4 (d, *J* = 11.1 Hz), 158.7 (d, *J* = 12.1 Hz), 145.2, 137.4, 135.1, 131.7, 130.8, 130.6 (d, *J* = 3.3 Hz), 130.5 (d, *J* = 3.4 Hz), 130.0, 129.6, 129.5, 127.4, 127.1, 126.0, 124.8, 120.0, 118.9 (d, *J* = 4.0 Hz), 118.7 (d, *J* = 4.1 Hz), 116.5, 112.4, 111.9 (d, *J* = 3.6 Hz), 111.7 (d, *J* = 3.6 Hz), 104.6, 104.3, 104.1, 21.7; **^{19}F NMR** (376 MHz, $CDCl_3$) δ: -106.61 (d, *J* = 8.3 Hz), -112.36 (d, *J* = 8.3 Hz); **HRMS** (ES^+) m/z: $[M+H]^+$ calculated for $[C_{27}H_{19}NO_2F_2S_2]^+$: 491.0830, found 491.0825; **IR ν_{max}** (cm^{-1}): 3078, 2911, 1599, 1479, 1485, 1420, 1375, 1174, 1120, 1021, 965, 855, 808, 779, 695, 661.

Synthesis of 2-phenyl-1-tosyl-3-((4-(trifluoromethyl)phenyl)thio)-1H-indole, **3ah**

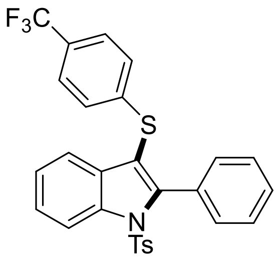

Synthesised in accordance with *General Procedure e* using 4-methyl-*N*-(2-(phenylethynyl)phenyl)benzenesulfonamide (**1a**) (35 mg, 0.1 mmol), 1-((4-(trifluoromethyl)phenyl)thio)pyrrolidine-2,5-dione (**2h**) (28 mg, 0.1 mmol), and $B(C_6F_5)_3$ (5.11 mg, 0.01 mmol). The desired product **3ah** was obtained as a white solid. R_f = 0.45 (15% ethyl acetate in hexane). Yield: 36 mg, 0.068 mmol, 68%. **^{1}H NMR** (400 MHz, $CDCl_3$) δ: 8.44–8.41 (m, 1H), 7.49–7.44 (m, 2H), 7.42–7.38 (m, 3H), 7.34–7.28 (m, 7H), 7.13–7.11 (m, 2H), 6.86–6.83 (m, 2H), 2.37 (s, 3H); **^{13}C NMR** (101 MHz, $CDCl_3$) δ: 146.0, 145.3, 142.4, 137.6, 135.0, 131.4, 130.8, 129.9, 129.6, 127.52, 127.48, 127.2, 127.1, 126.2, 125.79, 125.75, 125.7 (q, *J* = 11.0 Hz, *J* = 3.68 Hz), 122.8, 120.0, 116.8, 111.9, 21.7; **^{19}F NMR** (376 MHz, $CDCl_3$) δ: -62.42; **HRMS** (ES^+) m/z: $[M+H]^+$ calculated for $[C_{28}H_{20}NO_2F_3S_2]^+$: 523.0892, found 523.0888; **IR ν_{max}** (cm^{-1}): 3076, 2914, 1602, 1444, 1382, 1323, 1175, 1125, 1086, 1011, 944, 829, 760, 661, 659.

Synthesis of 3-(naphthalen-1-ylthio)-2-phenylbenzofuran, **3ki**

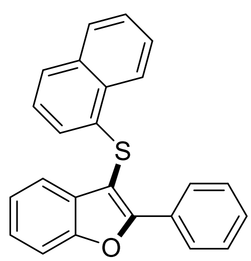

Synthesised in accordance with *General Procedure e* using 2-(phenylethynyl)phenol (**1k**) (20 mg, 0.1 mmol), 1-(4-tolylthio)pyrrolidine-2,5-dione (**2i**) (23 mg, 0.1 mmol), and $B(C_6F_5)_3$ (5.11 mg, 0.01 mmol). The desired product **3ki** was obtained as a white solid. R_f = 0.60 (2% ethyl acetate in hexane). Yield: 25 mg, 0.071 mmol, 71%. **^{1}H NMR** (400 MHz, $CDCl_3$) δ: 8.46 (dd, *J* = 8.3, 2.8 Hz, 1H), 8.29–8.25 (m, 2H), 7.89 (d, *J* = 8.0 Hz, 1H), 7.66–7.55 (m, 4H), 7.48–7.40 (m, 4H), 7.39–7.34 (m, 1H), 7.23–7.18 (m, 2H), 7.12–7.09 (m, 1H); **^{13}C NMR** (101 MHz, $CDCl_3$) δ: 158.1, 154.2, 134.1, 133.2, 131.2, 130.8, 129.9, 129.7, 129.6, 128.8, 127.5, 126.5, 126.4, 126.1, 126.0, 125.5, 124.1, 123.7, 123.6, 120.6, 111.5, 104.2; **HRMS** (ES$^+$) m/z: $[M+H]^+$ calculated for $[C_{24}H_{16}OS]^+$: 352.0926, found 352.0922; **IR** ν_{max} (cm^{-1}): 3324, 3045, 2248, 1599, 1492, 1380, 1334, 1166, 1088, 905, 721, 659.

Synthesis of 3-(cyclohexylthio)-2-phenylbenzofuran, **3kj**[22]

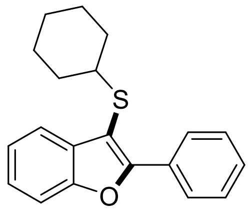

Synthesised in accordance with *General Procedure e* using 2-(phenylethynyl)phenol (**1k**) (20 mg, 0.1 mmol), 1-(cyclohexylthio)pyrrolidine-2,5-dione (**2j**) (22 mg, 0.1 mmol), and $B(C_6F_5)_3$ (5.11 mg, 0.01 mmol). The desired product **3kj** was obtained as a colourless liquid. R_f = 0.60 (2% ethyl acetate in hexane). Yield: 23 mg, 0.074 mmol, 74%. **^{1}H NMR** (400 MHz, $CDCl_3$) δ: 8.44–8.41 (m, 2H), 7.76–7.72 (m, 1H), 7.54–7.47 (m, 3H), 7.43–7.38 (m, 1H), 7.37–7.29 (m, 2H), 3.06 (tt, *J* = 10.8, 3.8 Hz, 1H), 1.97–1.91 (m, 2H), 1.75–1.68 (m, 2H), 1.58–1.51 (m, 1H), 1.44–1.36 (m, 2H), 1.24–1.17 (m, 3H); **^{13}C NMR** (101 MHz, $CDCl_3$) δ: 156.5, 153.7, 132.6, 130.7, 129.0, 128.5, 127.5, 125.0, 123.3, 120.5, 111.3, 107.1, 47.9, 33.8, 26.1, 25.8. The NMR data are in agreement with the previously reported literature values.

Synthesis of 2-phenyl-3-((trifluoromethyl)thio)benzofuran, **3kk**[23]

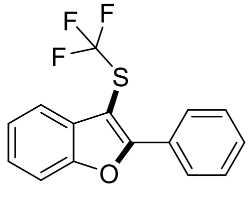

Synthesised in accordance with *General Procedure e* using 2-(phenylethynyl)phenol (**1k**) (20 mg, 0.1 mmol), 1-((trifluoromethyl)thio)pyrrolidine-2,5-dione (**2k**) (23 mg, 0.1 mmol), and $B(C_6F_5)_3$ (5.11 mg, 0.01 mmol). The desired product **3kk** was obtained as a white solid. R_f = 0.50 (2% ethyl acetate in hexane). Yield: 20 mg, 0.068 mmol, 68%. **^{1}H NMR** (400 MHz, $CDCl_3$) δ: 8.24–8.21 (m, 2H), 7.78–7.75 (m, 1H), 7.59–7.46 (m, 4H), 7.43–7.35 (m,

2H); **^{13}C NMR** (101 MHz, $CDCl_3$) δ: 160.2, 153.8, 131.0 (d, J = 4.5 Hz), 130.3, 129.1, 128.8, 128.1, 127.9, 125.8, 124.2, 120.3, 111.6, 97.6; **^{19}F NMR** (376 MHz, $CDCl_3$) δ: -42.05. The NMR data are in agreement with the previously reported literature values.

3.2 Synthesis of 6-membered (hetero)cyclic products 5aa–5ia

General Procedure for the synthesis of 6-membered (hetero)cyclic products **5aa–5ia**

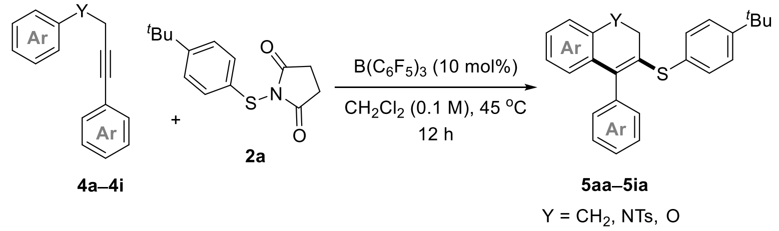

Scheme S12. Synthesis of 6-membered heterocyclic products.

In the glovebox, a glass microwave vial was charged with aryl-containing alkyne **4** (0.1 mmol, 1 equiv.), and 0.5 mL of dry CH_2Cl_2 was added to another vial equipped with the N-thiosuccinimide **2** (0.1 mmol, 1 equiv.) plus $B(C_6F_5)_3$ (0.01 mmol, 10 mol%) with a magnetic stirrer. All reactions were carried out at 45 °C for 12 h. After the reaction time, the solvent was removed in vacuo, and the crude compound was purified via preparative thin-layer chromatography using hexane/ethyl acetate as eluent.

Synthesis of 3-((4-(tert-butyl)phenyl)thio)-4-phenyl-2H-chromene, **5aa**

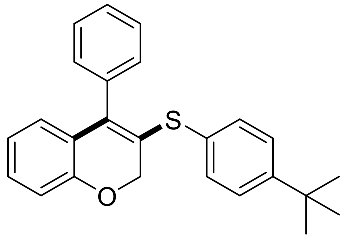

Synthesised in accordance with *General Procedure f* using (3-phenoxyprop-1-yn-1-yl)benzene (**4a**) (21 mg, 0.1 mmol), 1-((4-(*tert*-butyl)phenyl)thio)pyrrolidine-2,5-dione (**2a**) (27 mg, 0.1 mmol), and $B(C_6F_5)_3$ (5.11 mg, 0.01 mmol). The desired product **5aa** was obtained as a white solid. R_f = 0.50 (2% ethyl acetate in hexane). Yield: 20 mg, 0.053 mmol, 53%. **^{1}H NMR** (400 MHz, $CDCl_3$) δ: 7.47–7.40 (m, 3H), 7.31–7.28 (m, 6H), 7.16–7.12 (m, 1H), 6.89–6.81 (m, 2H), 6.74 (dd, J = 7.8, 1.6 Hz, 1H), 4.72 (s, 2H), 1.30 (s, 9H); **^{13}C NMR** (101 MHz, $CDCl_3$) δ: 153.8, 150.6, 138.3, 136.4, 130.5, 130.1, 129.9, 129.2, 128.4, 128.0, 126.5, 126.4, 125.3, 124.2, 121.7, 116.0, 68.4, 34.7, 31.4; **HRMS** (ES$^+$) m/z: [M+H]$^+$

calculated for $[C_{25}H_{23}OS]^+$: 371.1476, found 371.1470; **IR** ν_{max} (cm^{-1}): 2953, 2860, 1815, 1669, 1604, 1481, 1477, 1360, 1224, 1116, 823, 749, 702.

Synthesis of 3-((4-(tert-butyl)phenyl)thio)-6-methyl-4-phenyl-2H-chromene, **5ba**

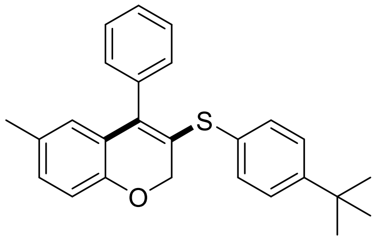

Synthesised in accordance with G*eneral Procedure f* using 1-methyl-4-((3-phenylprop-2-yn-1-yl)oxy)benzene (**4b**) (23 mg, 0.1 mmol), 1-((4-(*tert*-butyl)phenyl)thio)pyrrolidine-2,5-dione (**2a**) (27 mg, 0.1 mmol), and $B(C_6F_5)_3$ (5.11 mg, 0.01 mmol). The desired product **5ba** was obtained as a white solid. R_f = 0.50 (2% ethyl acetate in hexane). Yield: 22 mg, 0.056 mmol, 56%. **^{1}H NMR** (400 MHz, $CDCl_3$) δ: 7.47–7.42 (m, 3H), 7.31–7.29 (m, 6H), 6.95 (dd, *J* = 8.9, 2.3 Hz, 1H), 6.79 (d, *J* = 8.1 Hz, 1H), 6.54 (d, *J* = 2.2 Hz, 1H), 4.69 (s, 2H), 2.17 (s, 3H), 1.30 (s, 9H); **^{13}C NMR** (101 MHz, $CDCl_3$) δ: 151.7, 150.5, 138.5, 136.5, 131.0, 130.5, 130.2, 130.09, 129.7, 128.4, 128.0, 126.9, 126.4, 125.0, 124.3, 115.8, 68.4, 34.7, 31.4, 20.8; **HRMS** (ES^+) m/z: $[M+H]^+$ calculated for $[C_{26}H_{25}OS]^+$: 385.1633, found 385.1626; **IR** ν_{max} (cm^{-1}): 2953, 2892, 2258, 1807, 1490, 1366, 1174, 902, 827, 728, 693.

Synthesis of 3-((4-(tert-butyl)phenyl)thio)-4-(4-methoxyphenyl)-2H-chromene, **5ca**

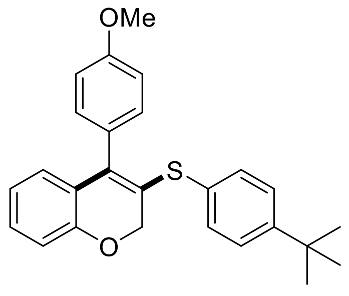

Synthesised in accordance with *General Procedure f* using 1-methoxy-4-(3-phenoxyprop-1-yn-1-yl)benzene (**4c**) (24 mg, 0.1 mmol), 1-((4-(*tert*-butyl)phenyl)thio)pyrrolidine-2,5-dione (**2a**) (27 mg, 0.1 mmol), and $B(C_6F_5)_3$ (5.11 mg, 0.01 mmol). The desired product **5ca** was obtained as a sticky colourless liquid. R_f = 0.50 (2% ethyl acetate in hexane). Yield: 21 mg, 0.053 mmol, 53%. **^{1}H NMR** (400 MHz, $CDCl_3$) δ: 7.30 (d, *J* = 1.5 Hz, 4H), 7.24–7.21 (m, 2H), 7.18–7.12(m, 1H), 6.99–6.96 (m, 2H), 6.88 (dd, *J* = 8.0, 1.3 Hz, 1H), 6.83 (dd, *J* = 7.3, 1.3 Hz, 1H), 6.78 (dd, *J* = 7.8, 1.9 Hz, 1H), 4.70 (s, 2H), 3.86 (s, 3H), 1.30 (s, 9H); **^{13}C NMR** (101 MHz, $CDCl_3$) δ: 159.4, 153.1, 150.5, 137.9, 131.4, 130.5, 130.1, 129.1, 128.6, 126.6, 126.4, 125.5, 123.9, 121.6, 116.0, 113.8, 68.5, 55.4, 34.7, 31.4; **HRMS** (ES^+) m/z: $[M+H]^+$ calculated for $[C_{26}H_{25}O_2S]^+$: 401.1576, found 401.1575; **IR** ν_{max} (cm^{-1}): 3347, 2985, 2260, 1817, 1505, 1248, 1179, 902, 821, 719, 652.

Synthesis of 4-(benzo[d][1,3]dioxol-5-yl)-3-((4-(tert-butyl)phenyl)thio)-2H-chromene, **5da**

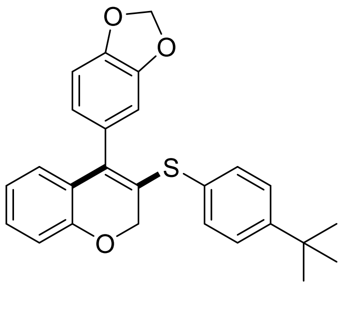

Synthesised in accordance with *General Procedure f* using 5-(3-phenoxyprop-1-yn-1-yl)benzo[d][1,3]dioxole (**4d**) (26 mg, 0.1 mmol), 1-((4-(*tert*-butyl)phenyl)thio)pyrrolidine-2,5-dione (**2a**) (27 mg, 0.1 mmol), and $B(C_6F_5)_3$ (5.11 mg, 0.01 mmol). The desired product **5da** was obtained as a sticky colourless liquid. R_f = 0.50 (5% ethyl acetate in hexane). Yield: 33 mg, 0.080 mmol, 80%. **^{1}H NMR** (400 MHz, $CDCl_3$) δ: 7.32–7.30 (m, 4H), 7.16–7.12 (m, 1H), 6.90–6.87 (m, 2H), 6.86–6.85 (m, 1H), 6.83–6.79 (m, 1H), 6.78–6.76 (m, 2H), 6.01 (s, 2H), 4.69 (s, 2H), 1.30 (s, 9H); **^{13}C NMR** (101 MHz, $CDCl_3$) δ: 153.9, 150.7, 147.7, 147.4, 137.6, 130.6, 130.0, 129.9, 129.2, 126.5, 126.4, 125.3, 124.4, 123.8, 121.7, 116.0, 110.4, 108.4, 101.3, 68.4, 34.7, 31.4; **HRMS** (ES$^+$) m/z: $[M+H]^+$ calculated for $[C_{26}H_{23}O_3S]^+$: 415.1374, found 415.1368; **IR** $\boldsymbol{\nu_{max}}$ (cm^{-1}): 2950, 2900, 1817, 1684, 1613, 1503, 1489, 1444, 1231, 1110, 1041, 940, 820, 762, 667.

Synthesis of 3-((4-(tert-butyl)phenyl)thio)-4-(3-(trifluoromethyl)phenyl)-2H-chromene, **5ea**

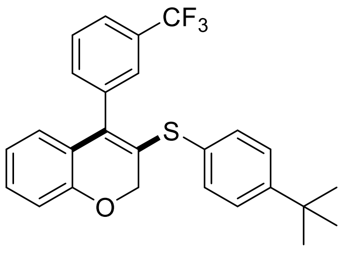

Synthesised in accordance with *General Procedure f* using 1-(3-phenoxyprop-1-yn-1-yl)-3-(trifluoromethyl)benzene (**4e**) (28 mg, 0.1 mmol), 1-((4-(*tert*-butyl)phenyl)thio)pyrrolidine-2,5-dione (**2a**) (27 mg, 0.1 mmol), and $B(C_6F_5)_3$ (5.11 mg, 0.01 mmol). The desired product **5ea** was obtained as a yellow oil. R_f= 0.50 (2% ethyl acetate in hexane). Yield: 18 mg, 0.040 mmol, 40%. **^{1}H NMR** (400 MHz, $CDCl_3$) δ: 7.68–7.64 (m, 1H), 7.60–7.55 (m, 1H), 7.50 (dt, *J* = 7.1, 1.3 Hz, 1H), 7.35–7.28 (m, 5H), 7.19–7.14 (m, 1H), 6.91–6.83 (m, 2H), 6.65 (dd, *J* = 7.8, 1.6 Hz, 1H), 4.73 (s, 2H), 1.31 (s, 9H); **^{13}C NMR** (101 MHz, $CDCl_3$) δ: 153.8, 151.1, 137.2, 136.5, 133.6, 131.1, 130.9, 130.8, 129.6, 129.3, 129.0, 127.0 (q, *J* = 12 Hz), 126.5, 126.1, 125.6, 125.0 (q, *J* = 11 Hz), 124.7, 121.9, 116.2, 68.4, 34.7, 31.4; **^{19}F NMR** (376 MHz, $CDCl_3$) δ: -62.50; **HRMS** (ES$^+$) m/z: $[M+H]^+$ calculated for $[C_{26}H_{22}OF_3S]^+$: 439.1350, found 439.1343; **IR** $\boldsymbol{\nu_{max}}$ (cm^{-1}): 2968, 2881, 1800, 1690, 1585, 1490, 1321, 1170, 1127, 1116, 821, 751, 706.

Synthesis of (4-(tert-butyl)phenyl)(1-phenyl-3,4-dihydronaphthalen-2-yl)sulfane, **5ha**

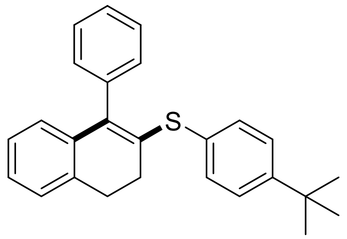

Synthesised in accordance with *General Procedure f* using but-1-yne-1,4-diyldibenzene (**4h**) (21 mg, 0.1 mmol), 1-((4-(*tert*-butyl)phenyl)thio)pyrrolidine-2,5-dione (**2a**) (27 mg, 0.1 mmol), and $B(C_6F_5)_3$ (5.11 mg, 0.01 mmol). The desired product **5ha** was obtained as a sticky colourless liquid. R_f = 0.50 (2% ethyl acetate in hexane). Yield: 35 mg, 0.094 mmol, 94%. **^{1}H NMR** (400 MHz, $CDCl_3$) δ: 7.48–7.44 (m, 2H), 7.41–7.38 (m, 1H), 7.32–7.30 (m, 6H), 7.18–7.12 (m, 2H), 7.10–7.06 (m, 1H), 6.71 (dd, *J* = 7.6, 1.4 Hz, 1H), 2.92–2.90 (m, 2H), 2.57–2.53 (m, 2H), 1.33 (s, 9H); **^{13}C NMR** (101 MHz, $CDCl_3$) δ: 150.2, 139.6, 139.2, 136.5, 135.1, 132.9, 131.6, 131.1, 130.3, 128.4, 127.5, 127.3, 126.9, 126.5, 126.2, 126.0, 34.7, 31.4, 29.6, 29.5; **HRMS** (ES^+) m/z: $[M+H]^+$ calculated for $[C_{26}H_{26}S]^+$: 370.1759, found 370.1755; **IR ν_{max}** (cm^{-1}): 2952, 2258, 1587, 1494, 1377, 1164, 1093, 905, 814, 724.

Synthesis of 3-((4-(tert-butyl)phenyl)thio)-4-phenyl-1-tosyl-1,2-dihydroquinoline, **5ia**

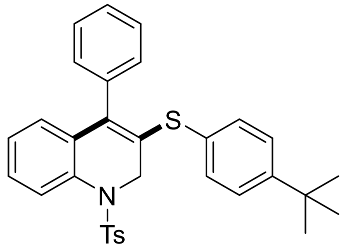

Synthesised in accordance with *General Procedure f* using 4-methyl-*N*-phenyl-*N*-(3-phenylprop-2-yn-1-yl)benzenesulfonamide (**4i**) (37 mg, 0.1 mmol), 1-((4-(*tert*-butyl)phenyl)thio)pyrrolidine-2,5-dione (**2a**) (27 mg, 0.1 mmol), and $B(C_6F_5)_3$ (5.11 mg, 0.01 mmol). The desired product **5ia** was obtained as a yellow liquid. R_f = 0.50 (2% ethyl acetate in hexane). Yield: 14 mg, 0.026 mmol, 26%. **^{1}H NMR** (400 MHz, $CDCl_3$) δ: 7.76 (dd, *J* = 8.0, 1.3 Hz, 1H), 7.40–7.37 (m, 3H), 7.36–7.33 (m, 3H), 7.31–7.28 (m, 4H), 7.20–7.17 (m, 2H), 7.10–7.06 (m, 1H), 6.61–6.56 (m, 3H), 4.41 (s, 2H), 2.42 (s, 3H), 1.33 (s, 9H); **^{13}C NMR** (101 MHz, $CDCl_3$) δ: 151.8, 143.8, 136.9, 135.9, 135.6, 133.8, 132.8, 132.2, 129.8, 129.3, 128.4, 128.2, 127.8, 127.7, 127.6, 127.2, 127.1, 126.8, 126.6, 125.1, 49.4, 34.8, 31.4, 21.6; **HRMS** (ES^+) m/z: $[M+H]^+$ calculated for $[C_{32}H_{31}NO_2S_2]^+$: 525.1799, found 525.1796; **IR ν_{max}** (cm^{-1}): 3076, 2952, 2896, 2098, 1591, 1489, 1453, 1356, 1164, 1114, 1011, 920, 833, 765, 682, 659.

3.3 Synthesis of PAHs

General Procedure g for the synthesis of PAHs **7** and **8**:

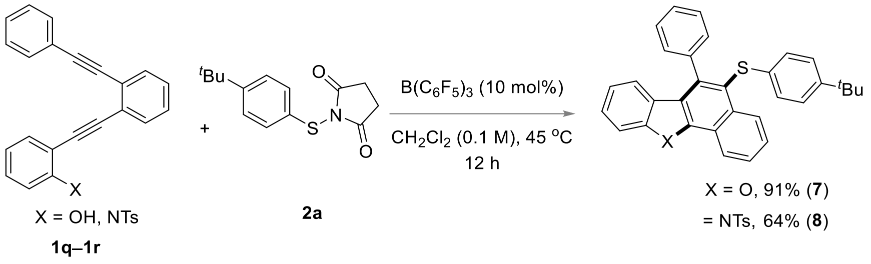

Scheme S13. Synthesis of PAHs.

In the glovebox, a glass microwave vial was charged with dialkyene **1q** or **1r** (0.1 mmol, 1 equiv.), and 0.5 mL of dry CH_2Cl_2 was added to another vial equipped *N*-thiosuccinimide **2** (0.1 mmol, 1 equiv.) plus $B(C_6F_5)_3$ (0.01 mmol, 10 mol%) with a magnetic stirrer. All reactions were carried out at 45 °C for 12 h. After the reaction time, the solvent was removed in vacuo, and the crude compound was purified via preparative thin-layer chromatography using hexane/ethyl acetate as eluent.

Synthesis of 5-((4-(tert-butyl)phenyl)thio)-6-phenylnaphtho[1,2-b]benzofuran, **7**

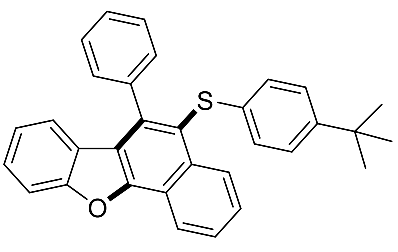

Synthesised in accordance with *General Procedure g* using 2-((2-(phenylethynyl)phenyl)ethynyl)phenol (**1q**) (30 mg, 0.1 mmol), 1-((4-(*tert*-butyl)phenyl)thio)pyrrolidine-2,5-dione (**2a**) (27 mg, 0.1 mmol), and $B(C_6F_5)_3$ (5.11 mg, 0.01 mmol). The desired product **7** was obtained as a sticky yellow liquid. R_f = 0.45 (5% ethyl acetate in hexane). Yield: 42 mg, 0.091 mmol, 91%. **^{1}H NMR** (400 MHz, $CDCl_3$) δ: 9.37 (dd, *J* = 8.1, 1.6 Hz, 1H), 7.91–7.89 (m, 2H), 7.71–7.64 (m, 4H), 7.54 (dd, *J* = 8.3, 1.4 Hz, 1H), 7.49–7.45 (m, 2H), 7.40–7.34 (m, 2H), 7.21–7.08 (m, 5H), 1.24 (s, 9H); **^{13}C NMR** (101 MHz, $CDCl_3$) δ: 153.4, 150.5, 148.3, 144.3, 134.0, 133.7, 132.6, 131.0, 129.8 × 2 129.0, 128.9, 127.2, 126.4, 126.3, 126.2, 125.1, 123.4, 121.5, 120.2, 119.9, 117.8, 117.7, 112.2, 34.5, 31.4. **HRMS** (ES^+) m/z: $[M+H]^+$ calculated for $[C_{32}H_{27}OS]^+$: 459.1772, found 459.1783; **IR** ν_{max} (cm^{-1}): 3069, 2959, 2862, 1634, 1592, 1541, 1444, 1354, 1256, 1114, 1058, 944, 905, 752, 692.

Synthesis of 5-((4-(tert-butyl)phenyl)thio)-6-phenyl-11-tosyl-11H-benzo[a]carbazole, **8**

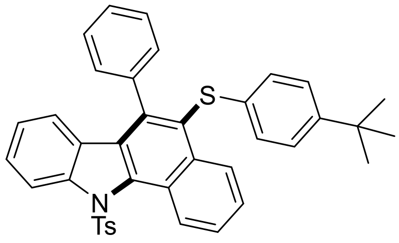

Synthesised in accordance with *General Procedure g* using 4-methyl *N*-(2-((2-(phenylethynyl)phenyl)ethynyl)phenyl) benzenesulfonamide (**1r**) (45 mg, 0.1 mmol), 1-((4-(*tert*-butyl)phenyl)thio)pyrrolidine-2,5-dione (**2a**) (27 mg, 0.1 mmol), and $B(C_6F_5)_3$ (5.11 mg, 0.01 mmol). The desired product **8** was obtained as a colourless liquid. R_f = 0.50 (15% ethyl acetate in hexane). Yield: 39 mg, 0.063 mmol, 64%. **^{1}H NMR** (400 MHz, $CDCl_3$) δ: 9.00 (d, *J* = 8.5 Hz, 1H), 8.73 (d, *J* = 8.5 Hz, 1H), 8.30 (d, *J* = 9.5 Hz, 1H), 7.73 (t, *J* = 7.6 Hz, 1H), 7.67–7.60 (m, 1H), 7.46–7.40 (m, 1H), 7.35 (t, *J* = 7.1 Hz, 3H), 7.13 (dd, *J* = 8.6, 1.9 Hz, 2H), 7.01–6.94 (m, 1H), 6.88 (d, *J* = 8.4 Hz, 2H), 6.84–6.72 (m, 6H), 6.05 (d, *J* = 8.6 Hz, 1H), 2.26 (s, 3H), 1.27 (s, 9H); **^{13}C NMR** (101 MHz, $CDCl_3$) δ: 148.3, 144.4, 142.4, 141.4, 139.1, 138.7, 135.6, 134.5, 130.7, 130.6, 129.1, 128.4, 128.24, 128.21, 128.0, 127.9, 127.8, 127.7, 127.4, 127.3, 126.8, 126.6, 126.5, 126.2, 125.9, 125.8, 121.9, 120.3, 34.4, 31.4, 21.6. **HRMS** (ES^+) m/z: $[M+H]^+$calculated for $[C_{39}H_{34}NO_2S_2]^+$: 612.2029, found 612.2031; **IR ν_{max}** (cm^{-1}): 2966, 2839, 2335, 2258, 1599, 1481, 1379, 1254, 1181, 1170, 1116, 1088, 965, 902, 777, 717, 652.

4. Computational details

Density functional theory (DFT) calculations were carried out with the ORCA quantum chemistry program, version 6.1.0.[24–25] Geometry optimisation and frequency calculations were performed with Grimme's composite method PBEh-3c, using a def2-mSVP basis set for all atoms.[26–27] The electronic energy was refined with Head-Gordon's range-separated hybrid meta-GGA functional with VV10 nonlocal correlation, ωB97M-V,[28] on a def2-TZVPD basis set for all atoms.[29–30]

Geometry optimisations were performed in gas phase, the solvation energy was approximated by a single point calculation on the same level of theory, alongside the conductor-like polarisable continuum model (C-PCM), using the dielectric constant of CH_2Cl_2 ($\varepsilon = 8.93$).[31–32] To account for the conversion of concentrations in gas and solution phase, 1.89 kcal mol^{-1} were added to each species.[33]

For geometry optimisations, tight optimisation criteria were used. For single point calculations, solvation single point calculations, frequency calculations and geometry optimisations, tight self-consistent field convergence criteria were chosen.[34,35] Further, in all cases, the RIJCOSX approximation was used,[36–37] employing Weigend's universal coulomb fitting basis (def2/J) as an auxiliary basis.[38] Thermodynamic corrections (zero point energy, enthalpy and entropy contributions) to the electronic energies were obtained at 313.15 K. The Gibbs free energy was computed according to the equations shown below:

$$\Delta G_{\mathrm{solv}} = E_{\mathrm{SCF,\,solv}} - E_{\mathrm{SCF,\,gas}}$$

$$H_{\mathrm{gas}} = E_{\mathrm{SCF,\,gas}} + ZPE + \mathrm{k_b}T$$

$$G_{\mathrm{sol}} = H_{\mathrm{gas}} - TS_{\mathrm{gas}} + \Delta G_{\mathrm{solv}} + 1.89\ \mathrm{kcal\ mol^{-1}}$$

$$\Delta G_{\mathrm{sol}} = \sum G_{\mathrm{sol,\,products}} - \sum G_{\mathrm{sol,\,reactants}}$$

4.1 Additional reaction energy profiles

As mentioned in the main text, multiple mechanistic scenarios are possible, depending on the preferential coordination of $B(C_6F_5)_3$ to the substances present in the reaction mixture. As outlined in Fig.5 in the main text, the $B(C_6F_5)_3$ Lewis acid can coordinate to the hydroxyl group of the alkynyl phenol **1k**, and thereby acidifying the O–H bond, making the proton available as a Brønsted acid catalyst. This Brønsted acid catalysed pathway can happen either via intramolecular

proton transfer to the alkyne or in an intermolecular fashion by protonation of another alkynyl phenol present in solution. Below shown are the reaction profiles for either scenario. The coordination of $B(C_6F_5)_3$ to the hydroxyl group is unfavoured when compared to the coordination to the imide-oxygen of thiosuccinimide **2a**. This preferential binding of $B(C_6F_5)_3$ to the thiosuccinimide leads to significantly higher barriers for the intra- and intermolecular proton transfer when compared to the formation of the thiirenium-ion formation, as outlined in Fig.5 in the main text. Barrier heights of 27.9 kcal mol^{-1} and 29.3 kcal mol^{-1} make these scenarios unlikely to compete with the formation of the main products through thiirenium-ion intermediates.

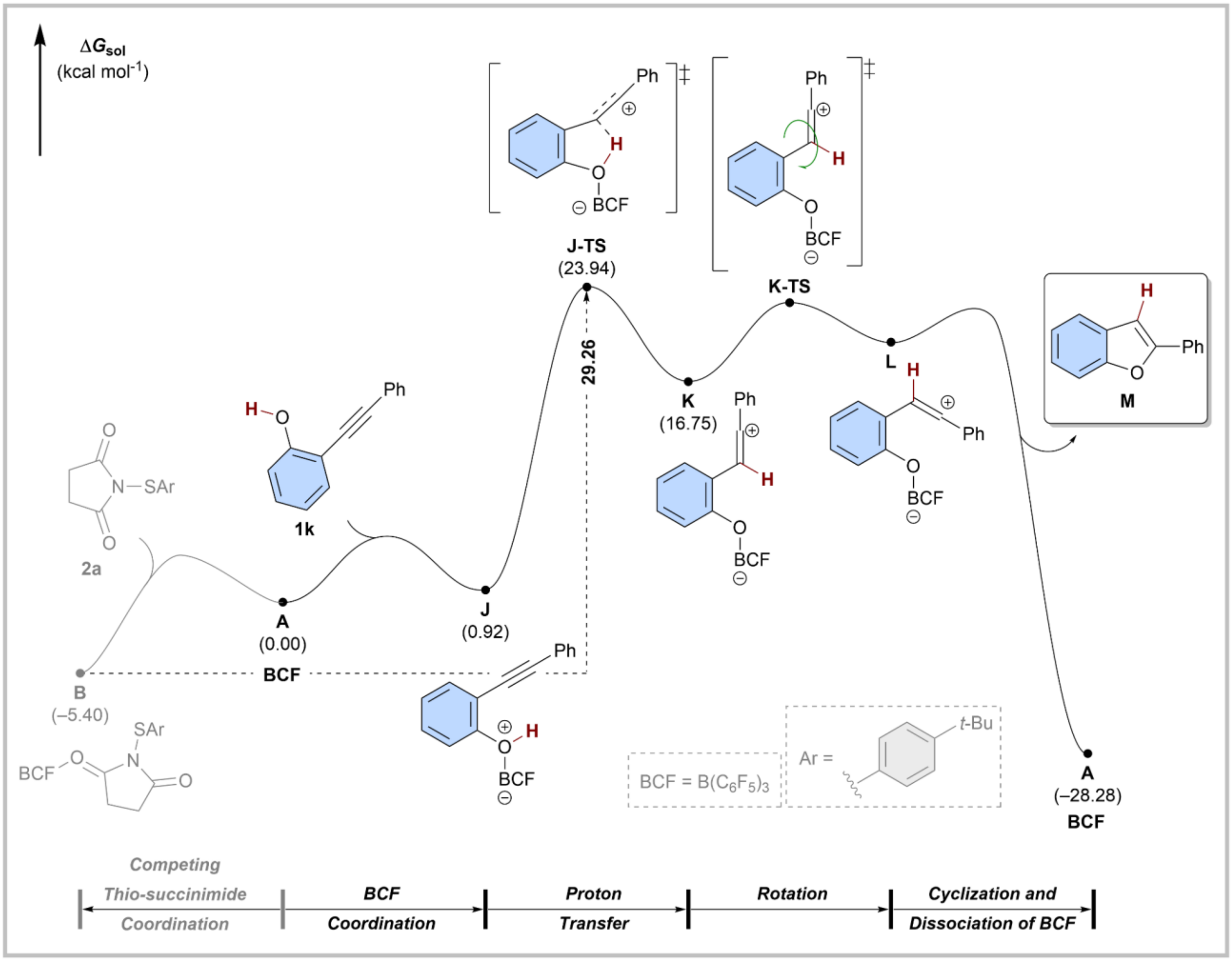

Scheme S14. Reaction energy profile of mechanistic scenario B: $B(C_6F_5)_3$ (BCF) coordination to the hydroxyl group of the alkynyl phenol **1k**, followed by an intramolecular proton transfer. Energies are Gibbs free energies at the ωB97M-V/def2-TZVPD//PBEh-3c/C-PCM(DCM) level of theory.

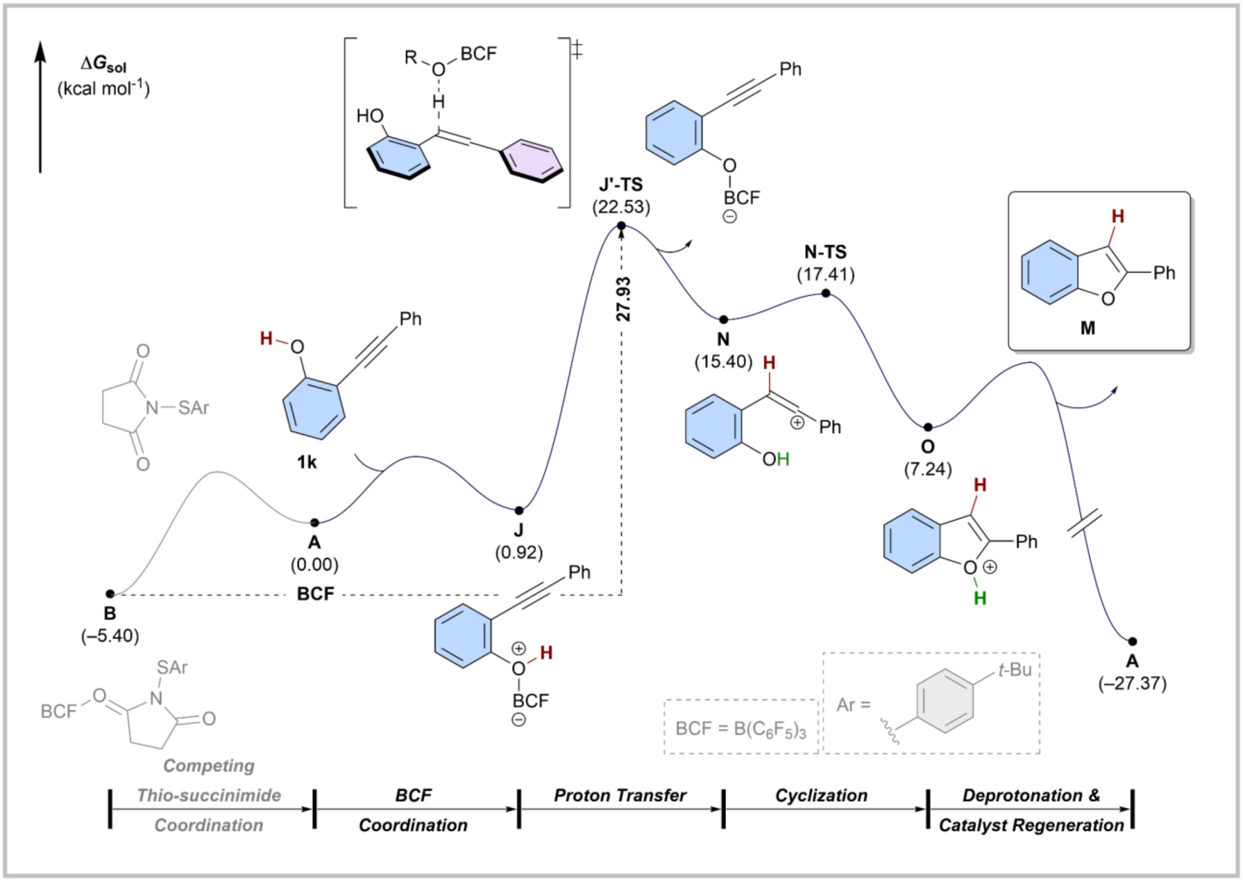

Scheme S15. Reaction energy profile of mechanistic scenario B: $B(C_6F_5)_3$ (BCF) coordination to the hydroxyl group of the alkynyl phenol **1k**, followed by an intermolecular proton transfer. Energies are Gibbs free energies at the ωB97M-V/def2-TZVPD//PBEh-3c/C-PCM(DCM) level of theory.

The last mechanistic scenario that was mentioned in the main text is the coordination of $B(C_6F_5)_3$ to the C–C triple bond of the alkynyl phenol. The associated reaction energy profile of this mechanistic pathway is summarised in Scheme S17. While the initial cyclisation is energetically feasible, the proton transfer from the oxonium ion to the C3 position of intermediate Q is prohibitively high in energy with an associated barrier of 43.6 kcal mol^{-1}. The associated barrier height indicates that this mechanistic pathway is not competing with the one highlighted in the main text.

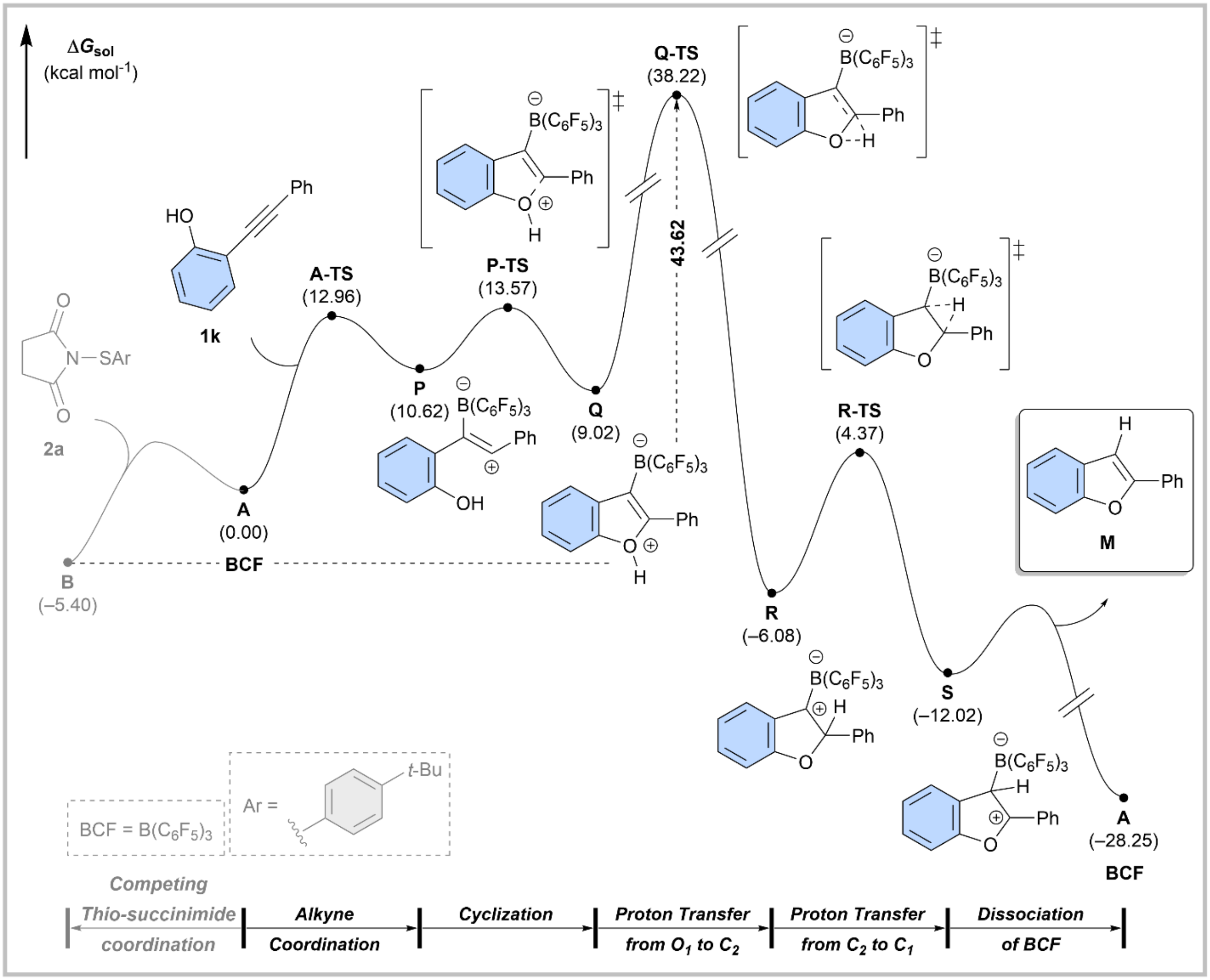

Scheme S16. Reaction energy profile of mechanistic scenario C: $B(C_6F_5)_3$ (BCF) coordination to the C–C triple bond of **1k**. Energies are Gibbs free energies at the ωB97M-V/def2-TZVPD//PBEh-3c/C-PCM(DCM) level of theory.

The intermolecular proton transfer form intermediate **Q** to another alkynyl phenol **1k** would be a plausible alternative pathway to access benzofuran **M** which can then undergo sulfenylation reactions as outlined in **Scheme S18.** This intermolecular proton transfer form **Q** to **1k** is summarized in **Scheme S17**.

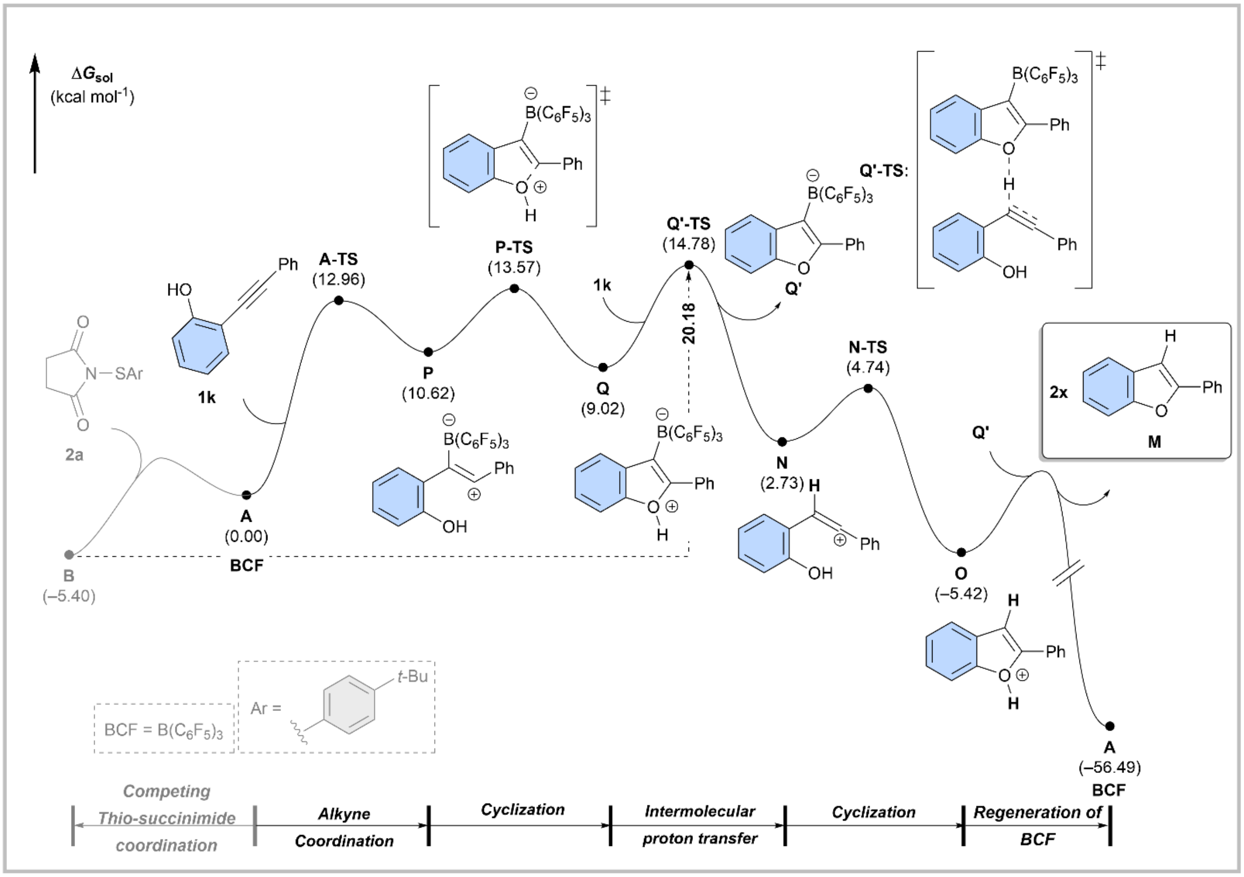

Scheme S17. Reaction energy profile of mechanistic scenario C: $B(C_6F_5)_3$ (BCF) coordination to the C–C triple bond of **1k** with subsequent intermolecular proton transfer from intermediate **Q** to another molecule of **1k**. Energies are Gibbs free energies at the ωB97M-V/def2-TZVPD//PBEh-3c/C-PCM(DCM) level of theory.

While the barrier for the formation of benzofuran **M** is calculated at 20.2 kcal mol^{-1}, which is energetically accessible under the reaction conditions, this mechanistic scenario implies a first-order dependency on $B(C_6F_5)_3$ and a second-order dependency on alkynyl phenol **1k** on the overall reaction rate. This is in disagreement with the experimentally found rate law, and is therefore unlikely to be the operative reaction mechanism under the reported conditions.

For completeness, Scheme S18 summarises the energy profile for the formation of **3ka** from 2-phenyl benzofuran, which would form based on the mechanistic scenarios summarised in Schemes S15–17.

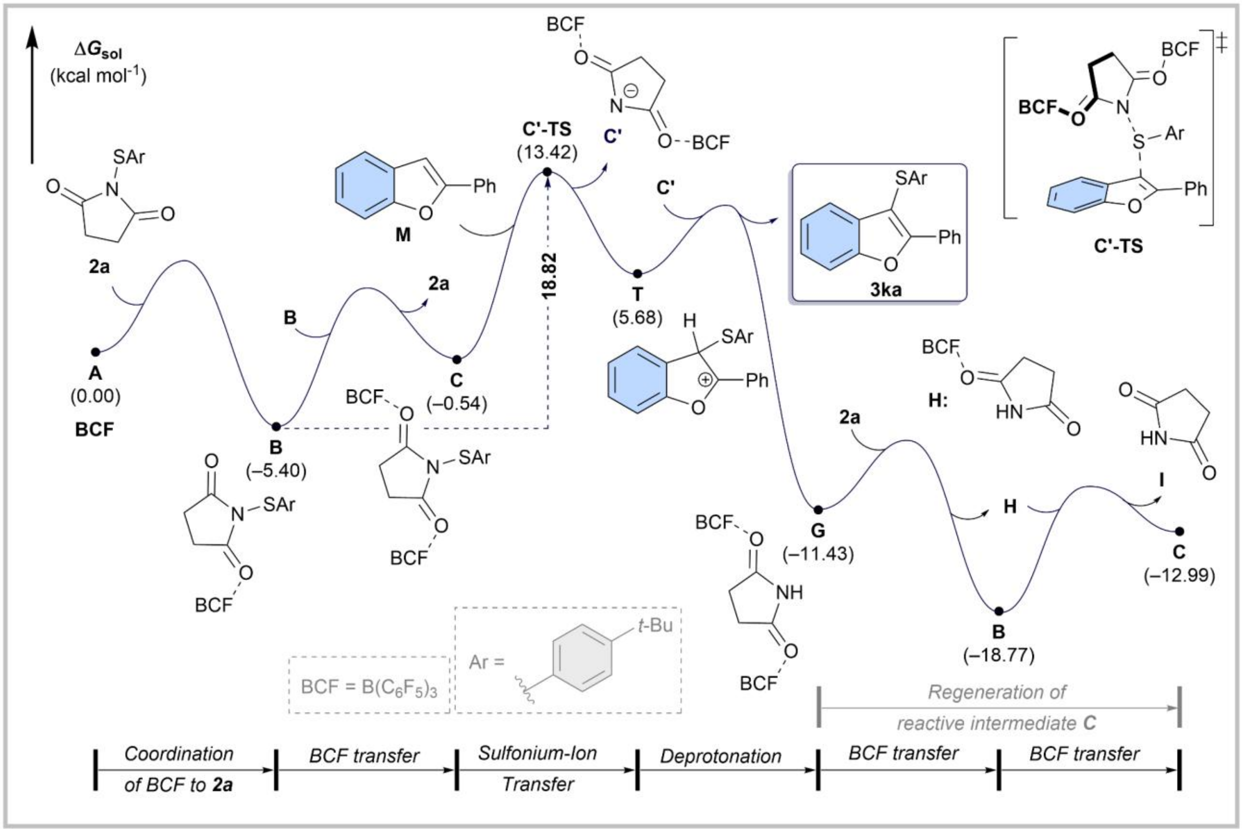

Scheme S18. Reaction energy profile of the formation of **3ka** from benzofuran **M**. Energies are Gibbs free energies at the ωB97M-V/def2-TZVPD//PBEh-3c/C-PCM(DCM) level of theory.

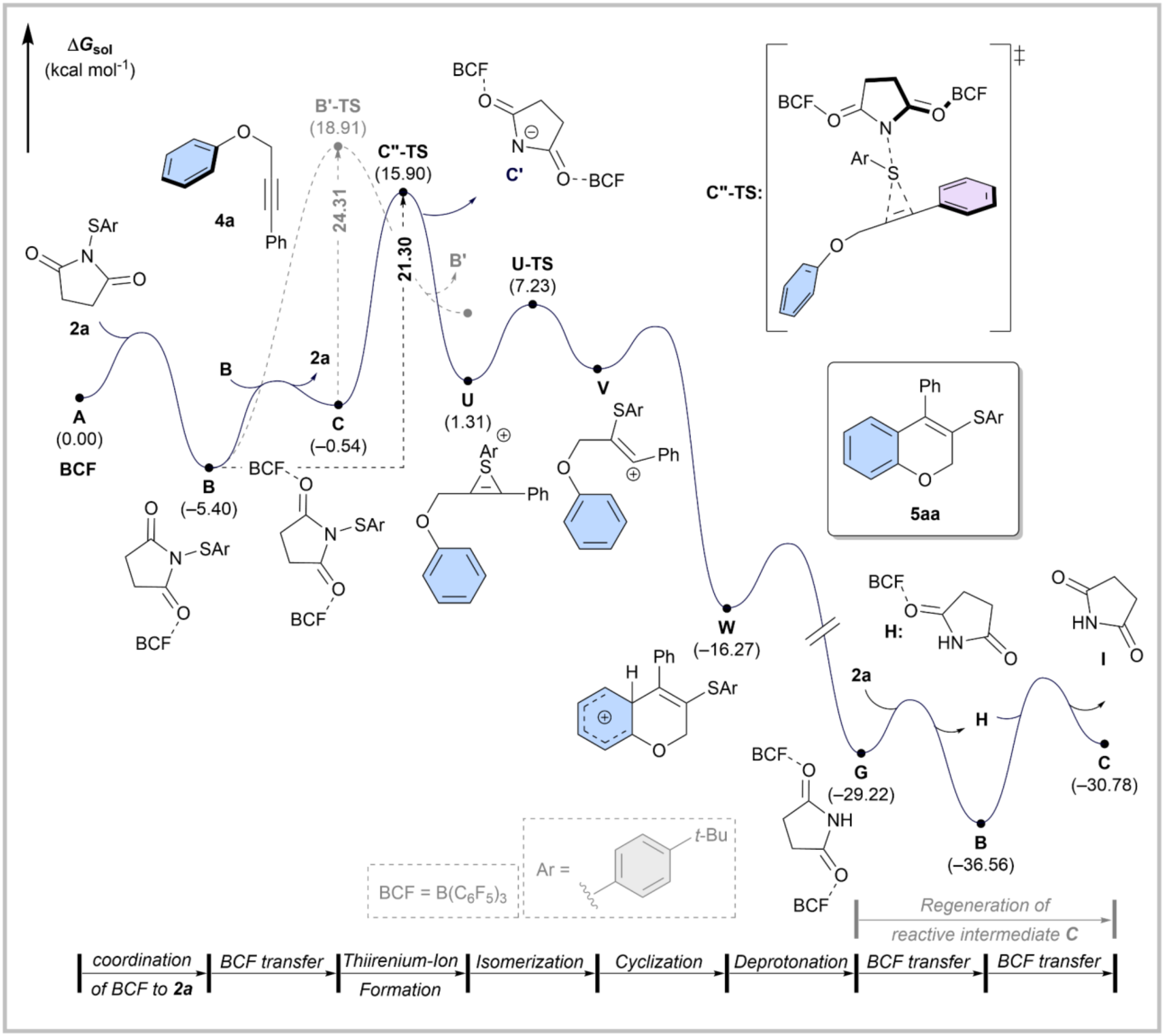

Scheme S19. Reaction energy profile for propargyl ether **4a**, following the same general mechanism as shown in the main text for substrate **1k**. Energies are Gibbs free energies at the ωB97M-V/def2-TZVPD//PBEh-3c/C-PCM(DCM) level of theory.

As mentioned in the main text, different substrates employed in this study show an unchanged mechanism, with largely identical sequences albeit slightly different barrier heights. Scheme S19 summarises the formation of 2*H*–chromene **5aa**, while Scheme S20 summarises the formation of the indole derivative **3aa**.

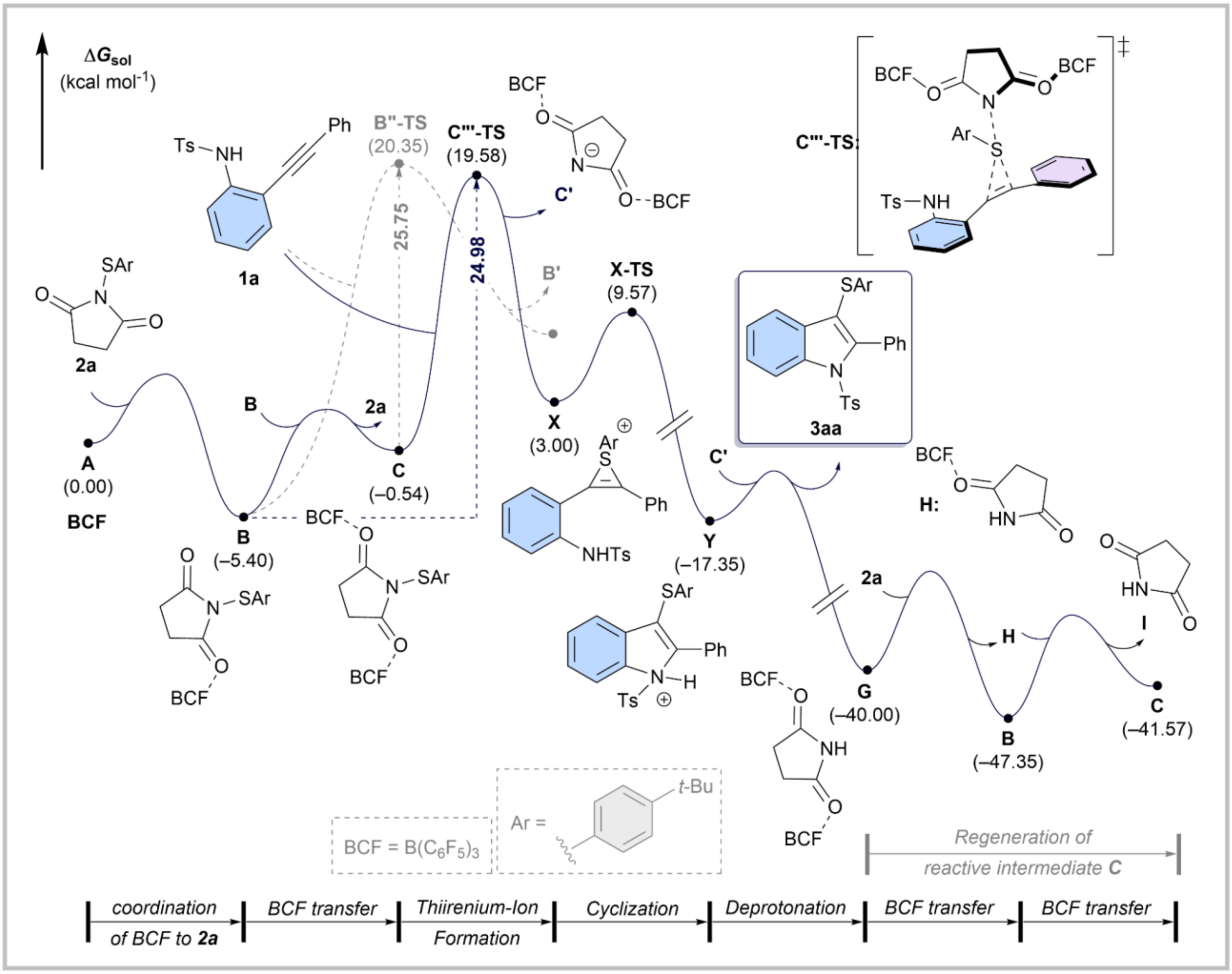

Scheme S20. Reaction energy profile for substrate **1a**, following the same general mechanism as shown in the main text for substrate **1k**. Energies are Gibbs free energies at the ωB97M-V/def2-TZVPD//PBEh-3c/C-PCM(DCM) level of theory.

4.2 Energies of optimised structures

Table S1: Energies of DFT optimised structures.

Label	E(SCF, DZ, Gas) (eV)	E(SCF, DZ, sol) (eV)	E(SCF, TZ, Gas) (eV)	ZPE (kcal mol-1)	Thermal Correction (kcal mol-1)	H (eV)	S (cal mol-1 K-1)	TS (kcal mol-1)	G(gas) (eV)	G(solv) (kcal mol-1)
**A**	-59971.629	-59971.783	-60102.469	101.16	19.17	-60097.224	182.09	57.02	-60099.700	-1385933.26
**B**	-91131.912	-91132.599	-91318.725	287.78	31.44	-91304.855	254.96	79.84	-91308.320	-2105633.27

**B'**	-69752.480	-69754.085	-69905.302	154.12	23.33	-69897.580	206.64	64.71	-69900.390	-1611975.93
**C**	-151104.073	-151104.953	-151421.953	390.21	51.28	-151402.781	378.19	118.43	-151407.920	-3491567.07
**C'**	-129725.815	-129727.136	-130009.568	257.02	43.03	-129996.530	330.07	103.36	-130001.010	-2997922.40
**D**	-38065.758	-38067.483	-38136.906	260.33	16.12	-38124.891	161.49	50.57	-38127.080	-879269.12
**E**	-38065.350	-38067.103	-38136.445	259.76	16.37	-38124.444	163.05	51.06	-38126.660	-879259.95
**F**	-38065.690	-38067.660	-38136.713	261.49	15.44	-38124.677	155.20	48.60	-38126.780	-879267.87
**G**	-129737.922	-129738.870	-130021.546	265.29	43.28	-130008.138	332.11	104.00	-130012.650	-2998182.14
**H**	-69765.948	-69766.599	-69918.545	162.77	23.48	-69910.441	207.60	65.01	-69913.260	-1612250.82
**I**	-9793.587	-9793.985	-9815.208	59.94	3.93	-9812.411	78.01	24.43	-9813.470	-226311.24
**J**	-76661.501	-76661.935	-76829.658	229.37	27.90	-76818.475	234.58	73.46	-76821.660	-1771557.26
**J'**	-76649.050	-76650.617	-76817.151	221.12	27.70	-76806.334	235.10	73.62	-76809.530	-1771303.59
**K**	-76660.422	-76661.308	-76828.440	227.91	27.85	-76817.322	235.61	73.78	-76820.520	-1771541.43
**M**	-16691.134	-16691.414	-16727.833	128.55	7.18	-16721.920	102.47	32.09	-16723.310	-385653.20
**N**	-16698.636	-16700.510	-16735.578	133.94	8.29	-16729.383	109.02	34.14	-16730.860	-385864.12
**O**	-16698.947	-16701.052	-16735.815	135.69	7.50	-16729.579	103.66	32.46	-16730.990	-385872.27
**P**	-76660.927	-76661.547	-76829.048	228.95	27.87	-76817.884	233.37	73.08	-76821.050	-1771547.56
**Q**	-76660.929	-76661.814	-76828.954	230.33	27.13	-76817.762	227.94	71.38	-76820.860	-1771549.16
**Q'**	-76649.990	-76651.469	-76817.952	222.93	27.54	-76807.064	228.32	71.50	-76810.160	-1771316.25

**R**	-76662.129	-76662.628	-76830.024	230.89	26.80	-76807.064	226.54	70.94	-76821.900	-1771564.26
**S**	-76662.396	-76662.868	-76830.307	230.76	26.85	-76818.823	226.38	70.89	-76822.180	-1771570.20
**T**	-38067.259	-38068.951	-38138.052	261.99	15.24	-38126.003	153.95	48.21	-38128.090	-879291.65
**U**	-39132.291	-39134.066	-39205.655	278.8	16.71	-39192.813	163.69	51.26	-39195.040	-903897.84
**W**	-39133.272	-39135.139	-39206.393	279.69	16.20	-39193.535	159.92	50.08	-39195.710	-903915.41
**X**	-59774.272	-59776.028	-59882.615	345.48	22.56	-59866.628	202.08	63.28	-59869.370	-1380658.80
**Y**	-59775.430	-59777.318	-59883.435	346.74	22.11	-59867.413	199.55	62.49	-59870.120	-1380679.15
**A-TS**	-76660.846	-76661.392	-76829.004	228.49	27.65	-76817.870	232.44	72.79	-76821.030	-1771545.22
**B-TS**	-107821.041	-107821.890	-108044.943	414.86	40.47	-108025.171	311.42	97.52	-108029.400	-2491234.24
**C-TS**	-167793.637	-167794.601	-168148.582	517.49	60.30	-168123.500	433.24	135.67	-168129.380	-3877175.09
**E-TS**	-38065.318	-38067.093	-38136.396	259.58	15.82	-38124.427	158.49	49.63	-38126.580	-879258.62
**J-TS**	-76660.219	-76660.821	-76828.359	226.05	27.48	-76817.338	232.41	72.78	-76820.490	-1771534.24
**J'-TS**	-93350.421	-93351.273	-93555.645	353.58	36.48	-93538.703	286.09	89.59	-93542.590	-2157160.57
**N-TS**	-16698.566	-16700.463	-16735.487	133.79	7.77	-16729.321	105.48	33.03	-16730.750	-385862.11
**P-TS**	-76660.793	-76661.498	-76828.870	228.94	27.21	-76817.735	228.68	71.61	-76820.840	-1771544.61
**Q’-TS**	-93350.832	-93351.827	-93555.879	354.58	37.12	-93538.866	288.29	90.28	-93542.780	-2157168.32
**G-TS**	-76659.750	-76660.340	-76827.865	227.37	26.90	-76816.812	226.44	70.91	-76819.890	-1771519.96
**R-TS**	-76661.460	-76661.950	-76829.499	228.7	26.62	-76818.400	224.91	70.43	-76821.450	-1771553.81

**C'-TS**	-167795.569	-167796.488	-168150.11[illegible]	519.35	59.21	-168125.001	425.71	133.31	-168130.780	-3877206.32
**B'-TS**	-108887.768	-108888.545	-109113.83[illegible]	433.31	41.07	-109093.241	315.02	98.65	-109097.520	-2515863.97
**C"-TS**	-168860.504	-168861.409	-169217.51[illegible]	536.25	60.85	-169191.592	436.85	136.80	-169197.520	-3901805.65
**U-TS**	-39132.023	-39133.774	-39205.351	277.85	16.73	-39192.550	165.96	51.97	-39194.800	-903891.91
**B"-TS**	-129529.385	-129530.435	-129790.513	499.45	47.10	-129766.785	352.64	110.43	-129771.570	-2992625.20
**C"'-TS**	-189501.950	-189502.954	-189894.264	602	66.91	-189865.230	473.22	148.19	-189871.660	-4378564.63
**X-TS**	-59774.060	-59775.776	-59882.326	344.6	22.48	-59866.381	202.27	63.34	-59869.130	-1380652.23
**1a**	-38397.784	-38398.362	-38472.051	212.17	14.51	-38462.194	150.66	47.18	-38464.240	-887017.68
**2a**	-31159.605	-31160.063	-31215.315	185.35	11.81	-31206.738	132.94	41.63	-31208.540	-719694.61
**3aa**	-59766.062	-59766.651	-59874.150	338.91	21.83	-59858.480	195.98	61.37	-59861.140	-1380442.07
**5aa**	-39124.549	-39124.934	-39197.643	272.65	15.94	-39185.102	158.14	49.52	-39187.250	-903686.20
**4a**	-17755.986	-17756.346	-17795.192	145.31	8.97	-17788.475	117.29	36.73	-17790.070	-410255.02
**3ka**	-38057.662	-38058.032	-38128.495	254.31	15.19	-38116.781	155.61	48.73	-38118.890	-879049.02
**1k**	-16689.275	-16689.736	-16726.301	126.88	8.17	-16720.418	109.47	34.28	-16721.900	-385624.92

4.3 Vibrational frequencies of optimised structures

A

19.25, 20.4, 31.52, 31.73, 32.93, 35.26, 104.62, 110.95, 111.76, 133.9, 134.23, 134.69, 145.23, 145.91, 160.5, 169.3, 175.65, 175.98, 239.74, 244.74, 245.55, 284.87, 285.31, 285.48, 287.01, 288.06, 289.25, 323.51, 324.3, 324.39, 354.46, 355.31, 365.37, 376.13, 407.47, 408.44, 431.62, 432.28, 433.95, 455.13, 455.56, 455.59, 490.29, 490.34, 515.61, 574.54, 598.78, 598.8, 601.29, 659.01, 659.6, 693.46, 693.69, 695.15, 696.7, 696.93, 698.62, 749.65, 749.77, 751.39, 812.07, 812.67, 825.82, 900.91, 1041.46, 1041.66, 1044.91, 1082.96, 1083.17, 1183.52, 1231.34, 1232.13, 1232.39, 1251.41, 1252.24, 1354.5, 1355.96, 1356.14, 1394.9, 1412.26, 1412.78, 1490.78,

1491.53, 1501.05, 1593.95, 1596.42, 1596.61, 1624.58, 1624.67, 1625.33, 1733.19, 1734.63, 1734.75, 1752.76, 1752.89, 1754.13

B

14.95, 25.32, 26.3, 27.96, 34.73, 40.76, 42.62, 51.94, 52.19, 55.33, 63.36, 66.07, 72.83, 81.72, 89.29, 102.82, 109.86, 121.99, 128.69, 131.46, 138.64, 140.35, 145.7, 151.99, 157.2, 161.39, 168.35, 177.03, 180.92, 186.76, 195.18, 212.06, 243.58, 247.14, 252.95, 257.33, 264.06, 268.14, 279.02, 283.63, 283.99, 285.93, 288.63, 293.86, 294.69, 298.29, 304.78, 329.98, 330.64, 335.15, 340.1, 352.35, 358.59, 362.3, 365.48, 370.58, 375.52, 400.42, 415.73, 416.42, 427.55, 429.85, 432.46, 435.34, 441.86, 454.38, 459.78, 460.08, 461.33, 475.19, 492.98, 494.29, 498.5, 506.68, 551.82, 570.57, 580.9, 592.86, 598.2, 599.1, 601.47, 614.24, 627.28, 637.21, 649.81, 664.85, 665.9, 683.61, 691.41, 693.33, 696.58, 700.43, 705.19, 710.6, 751.41, 753.22, 754.79, 767.78, 778.74, 779.68, 798.84, 807.53, 823.77, 864.73, 865.66, 872.73, 887.16, 897.3, 971.87, 982.13, 994.87, 996.46, 998.05, 1007.29, 1017.21, 1022.48, 1036.38, 1043.94, 1054.95, 1056.26, 1060.2, 1062.66, 1081.64, 1083.49, 1087.43, 1154.26, 1164.9, 1179.17, 1182.69, 1190.32, 1193.2, 1212.53, 1215.32, 1219.18, 1222.29, 1228.81, 1266.9, 1279.26, 1279.91, 1295.35, 1349.09, 1349.55, 1351.96, 1352.89, 1355.13, 1376.2, 1378.12, 1379.33, 1380.28, 1381.73, 1387.29, 1455.96, 1458.16, 1464.57, 1484.39, 1486.79, 1487.88, 1489.49, 1492.7, 1498.86, 1519.55, 1534.58, 1536.5, 1541.54, 1557.86, 1563.99, 1573.98, 1574.72, 1584.88, 1586.58, 1592.47, 1624.25, 1625.59, 1627.79, 1682.23, 1724.75, 1736.48, 1738.07, 1740.1, 1752.71, 1753.98, 1757.77, 1821.12, 2002.93, 3111.02, 3114.79, 3123.35, 3149.73, 3158.14, 3192.41, 3193.99, 3198.26, 3200.47, 3201.47, 3202.62, 3214.78, 3221.56, 3258.03, 3274.19, 3279.14, 3293.38

B'

22.76, 26.59, 31.04, 33.5, 38.11, 40.8, 44.58, 55.59, 72.23, 108.22, 120.92, 125.73, 134.35, 137.35, 142.83, 145.56, 156.63, 157.39, 160.88, 164.03, 177.38, 181.31, 183.58, 192.38, 227.1, 245.07, 254.35, 255.98, 283.71, 284.61, 285.28, 289.86, 293.8, 294.73, 297.99, 329.54, 330.04, 335.52, 360.1, 363.46, 369.25, 396.4, 417.92, 425.9, 431.17, 432.57, 437.02, 460.04, 460.69, 461.21, 482.62, 495.75, 501.19, 515.47, 575.37, 591.43, 597.79, 599.77, 600.74, 606.2, 634.71, 649.75, 663.81, 680.61, 693.72, 695.82, 698.24, 701.84, 714.22, 721.36, 753.58, 771.4, 780.91, 796.55, 802.46, 805.15, 852.87, 875.87, 882.59, 958.99, 972.7, 1003.05, 1033.9, 1039.01, 1047.34, 1059.88, 1065.79, 1068.52, 1157.87, 1162.69, 1172.61, 1201.99, 1208.26, 1213.91, 1214.5, 1284.01, 1317.53, 1327.81, 1348.2, 1350.78, 1353.39, 1355.92, 1364.33, 1367.08, 1368.34, 1476.81, 1482.86, 1484.63, 1503.38, 1514.61, 1536.63, 1568.52, 1580.27, 1582.1, 1617.4, 1618.14, 1620.46, 1704.97, 1735.44, 1738.04, 1739.21, 1747.78, 1749.04, 1752.02, 1923.37, 3140.49, 3145.78, 3198.28, 3209.04

C

5.46, 11.81, 13.01, 18.63, 19.97, 23.73, 24.58, 27.46, 27.62, 31.44, 33.77, 36.38, 38.55, 40.59, 42.75, 44.55, 50.86, 55.25, 56.36, 68.36, 71.7, 74.98, 88.28, 97.78, 102.03, 103.94, 112.55, 118.8, 122.0, 125.32, 127.99, 130.39, 133.26, 139.36, 139.53, 140.45, 142.24, 145.72, 147.6, 151.4, 153.26, 158.01, 161.01, 163.38, 167.33, 170.83, 175.9, 177.28, 178.07, 180.27, 183.27, 185.92, 191.01, 206.5, 223.22, 244.84, 248.25, 248.73, 252.35, 253.73, 257.7, 260.2, 265.0, 273.9, 281.66,

283.57, 283.87, 284.44, 284.86, 285.13, 286.11, 290.23, 292.99, 293.38, 293.65, 294.14, 295.55, 297.01, 309.71, 315.76, 329.06, 329.71, 329.94, 331.39, 335.54, 340.23, 352.4, 354.72, 358.84, 361.48, 363.02, 364.18, 365.13, 370.39, 371.83, 374.22, 398.02, 401.12, 413.61, 418.2, 424.69, 425.19, 429.86, 431.03, 432.18, 433.21, 435.33, 436.74, 440.97, 445.05, 459.53, 459.8, 459.88, 460.62, 460.8, 460.99, 468.49, 476.88, 491.39, 494.12, 495.14, 495.92, 501.44, 506.16, 508.24, 551.61, 574.28, 576.5, 592.28, 598.17, 598.36, 598.91, 599.24, 601.04, 602.08, 610.44, 616.83, 627.28, 637.82, 650.47, 656.69, 662.79, 666.3, 667.85, 682.61, 687.68, 690.4, 692.09, 694.37, 695.67, 696.87, 699.46, 704.93, 707.98, 709.63, 715.9, 731.22, 752.17, 755.21, 756.57, 762.95, 767.85, 776.23, 777.65, 779.32, 783.23, 798.59, 804.9, 808.33, 809.07, 816.63, 823.41, 865.86, 868.21, 874.66, 886.7, 892.87, 894.0, 971.2, 978.43, 981.9, 996.9, 1002.29, 1008.31, 1010.6, 1014.09, 1025.94, 1027.04, 1037.22, 1040.12, 1045.63, 1045.91, 1055.48, 1057.3, 1058.73, 1060.32, 1071.76, 1079.45, 1086.33, 1088.41, 1155.12, 1166.54, 1169.08, 1180.94, 1183.9, 1187.36, 1192.83, 1196.13, 1196.53, 1214.65, 1214.9, 1218.81, 1219.95, 1222.06, 1223.15, 1231.98, 1266.6, 1278.03, 1279.94, 1294.39, 1325.25, 1347.82, 1348.07, 1349.5, 1349.78, 1351.54, 1352.14, 1352.95, 1354.15, 1377.85, 1380.18, 1380.84, 1382.04, 1382.66, 1383.23, 1385.87, 1388.1, 1399.14, 1449.28, 1457.47, 1458.55, 1481.02, 1485.95, 1486.78, 1487.9, 1488.81, 1490.53, 1491.08, 1493.74, 1494.33, 1508.6, 1532.44, 1536.28, 1538.94, 1557.37, 1564.46, 1572.65, 1575.26, 1578.57, 1582.71, 1586.1, 1587.9, 1589.4, 1591.51, 1625.42, 1625.49, 1626.6, 1627.04, 1628.61, 1629.73, 1682.21, 1721.11, 1733.45, 1736.06, 1736.97, 1737.95, 1738.83, 1740.77, 1751.64, 1753.14, 1753.75, 1756.21, 1757.7, 1758.59, 1812.49, 1896.1, 3115.14, 3117.34, 3122.31, 3144.01, 3157.49, 3196.66, 3197.27, 3200.74, 3204.28, 3208.18, 3211.5, 3214.97, 3233.22, 3266.51, 3269.43, 3281.86, 3300.9

C'

6.87, 8.32, 15.37, 21.2, 22.22, 25.05, 27.87, 30.47, 31.24, 35.61, 36.03, 38.49, 41.01, 43.09, 44.28, 51.37, 51.83, 88.93, 104.49, 106.88, 117.45, 122.08, 125.67, 128.35, 133.07, 135.45, 137.47, 138.06, 139.8, 144.91, 145.3, 147.98, 152.9, 154.18, 158.39, 160.0, 160.67, 166.5, 168.83, 176.01, 176.44, 180.0, 180.85, 185.75, 185.82, 217.39, 234.19, 243.6, 244.92, 251.05, 253.36, 255.32, 256.39, 283.3, 283.57, 284.32, 284.9, 285.08, 285.28, 287.48, 293.09, 293.6, 293.88, 294.19, 295.01, 296.81, 304.91, 316.73, 329.46, 329.6, 330.16, 330.77, 336.37, 341.97, 359.76, 362.05, 363.94, 364.9, 369.52, 372.59, 395.67, 403.64, 416.14, 427.02, 427.46, 431.88, 431.96, 432.55, 433.56, 437.03, 439.22, 443.26, 460.08, 460.18, 460.48, 460.58, 461.05, 461.17, 489.4, 495.07, 495.79, 496.91, 504.56, 508.37, 548.26, 577.62, 597.55, 597.75, 598.33, 599.12, 600.79, 600.92, 605.53, 613.95, 636.59, 650.27, 653.98, 656.01, 667.33, 670.19, 684.97, 693.76, 693.95, 694.39, 694.5, 694.99, 698.68, 699.12, 705.33, 707.92, 717.75, 718.73, 754.57, 754.71, 771.7, 771.94, 781.42, 781.99, 796.24, 798.88, 801.87, 806.82, 807.44, 807.5, 840.24, 858.93, 890.44, 898.85, 923.23, 982.29, 986.18, 993.84, 1013.28, 1015.4, 1035.95, 1036.04, 1043.8, 1047.13, 1051.03, 1051.36, 1064.96, 1065.98, 1071.34, 1160.49, 1163.82, 1170.4, 1170.6, 1181.57, 1181.83, 1209.73, 1210.61, 1215.98, 1217.35, 1217.71, 1218.31, 1221.07, 1291.07, 1331.61, 1347.96, 1348.16, 1349.98, 1350.28, 1352.51, 1352.8, 1366.35, 1370.8, 1371.11, 1372.25, 1373.56, 1375.71, 1376.12, 1477.42, 1479.51, 1482.27, 1485.37, 1486.66, 1489.49, 1491.26, 1502.8, 1526.85, 1528.09, 1570.42, 1571.13, 1576.82, 1583.58, 1585.18, 1585.35, 1620.45, 1620.63, 1621.43, 1621.66, 1621.84, 1624.54, 1640.9, 1735.59, 1735.78, 1737.75, 1738.14, 1739.45,

1739.59, 1750.24, 1750.34, 1751.03, 1751.28, 1754.25, 1754.46, 1791.88, 3146.71, 3148.7, 3226.56, 3231.54

D

13.56, 18.82, 20.2, 27.31, 33.9, 53.12, 73.16, 77.88, 113.23, 141.09, 147.81, 185.17, 209.59, 242.22, 250.86, 256.22, 272.9, 284.62, 297.53, 323.08, 333.41, 351.44, 355.49, 370.13, 406.48, 416.2, 417.45, 422.22, 426.34, 457.59, 465.98, 486.52, 505.96, 517.81, 546.62, 564.13, 574.87, 607.74, 610.79, 624.61, 638.66, 657.33, 702.33, 718.26, 723.36, 761.47, 774.94, 775.88, 786.52, 798.73, 803.34, 864.91, 875.67, 879.22, 882.78, 887.15, 901.56, 921.97, 972.93, 982.24, 985.06, 997.99, 1003.14, 1014.4, 1031.46, 1037.36, 1042.17, 1047.26, 1051.25, 1068.86, 1079.32, 1084.96, 1085.08, 1101.75, 1140.35, 1150.62, 1161.75, 1173.09, 1193.14, 1205.43, 1224.69, 1227.78, 1239.87, 1247.85, 1264.89, 1277.14, 1280.15, 1344.66, 1346.52, 1364.93, 1379.93, 1380.28, 1390.5, 1396.18, 1410.53, 1417.16, 1459.0, 1459.05, 1490.28, 1490.66, 1532.44, 1533.72, 1536.11, 1538.59, 1548.65, 1557.84, 1564.68, 1572.63, 1578.47, 1586.76, 1592.76, 1683.15, 1691.35, 1700.61, 1709.04, 1714.73, 1727.39, 1970.33, 3114.92, 3117.94, 3121.43, 3196.78, 3201.34, 3202.79, 3205.35, 3205.79, 3208.81, 3259.41, 3259.71, 3268.95, 3269.55, 3273.59, 3284.15, 3285.56, 3292.42, 3293.99, 3302.49, 3304.85, 3308.84, 3315.09, 3908.01

E

3.42, 25.03, 35.11, 38.85, 43.3, 63.81, 79.92, 85.48, 99.83, 121.19, 126.31, 167.31, 185.0, 217.13, 243.05, 257.65, 265.35, 284.2, 288.55, 292.2, 301.61, 327.32, 354.94, 358.37, 374.33, 404.64, 407.2, 422.58, 432.02, 446.1, 468.23, 474.01, 482.31, 500.84, 538.74, 553.63, 572.57, 587.26, 606.39, 613.87, 630.32, 638.33, 661.66, 686.76, 694.89, 773.18, 778.13, 780.91, 798.14, 819.12, 859.05, 868.12, 883.72, 885.87, 890.21, 890.89, 900.93, 933.58, 971.79, 982.07, 997.18, 1003.81, 1019.72, 1024.3, 1033.52, 1036.26, 1046.95, 1049.58, 1055.56, 1071.97, 1076.15, 1078.25, 1085.02, 1102.77, 1147.43, 1153.16, 1161.92, 1174.16, 1179.63, 1215.89, 1227.53, 1229.26, 1232.37, 1249.59, 1257.34, 1277.72, 1279.99, 1332.84, 1344.99, 1369.03, 1371.24, 1372.22, 1378.9, 1390.27, 1411.14, 1431.41, 1455.09, 1458.27, 1487.06, 1488.22, 1521.64, 1533.19, 1535.63, 1538.61, 1548.4, 1556.68, 1561.34, 1563.66, 1572.38, 1586.34, 1592.77, 1661.51, 1675.05, 1704.19, 1709.04, 1714.73, 1726.26, 1943.36, 3112.66, 3115.54, 3120.88, 3193.73, 3196.78, 3201.15, 3202.3, 3206.6, 3209.82, 3263.17, 3268.27, 3275.54, 3280.36, 3281.64, 3283.23, 3292.23, 3293.98, 3299.14, 3299.65, 3306.56, 3306.92, 3311.97, 3901.7

F

12.14, 30.64, 44.97, 55.98, 63.3, 71.49, 90.37, 109.93, 122.36, 131.24, 185.39, 191.72, 227.78, 260.89, 271.3, 281.22, 285.63, 295.91, 301.21, 335.32, 354.29, 356.05, 371.71, 393.64, 414.36, 420.58, 430.66, 437.86, 458.33, 479.76, 484.48, 493.66, 531.8, 579.58, 582.85, 609.53, 618.58, 632.06, 636.45, 649.04, 661.63, 699.87, 710.27, 732.54, 767.58, 769.55, 780.79, 789.93, 801.46, 811.4, 881.05, 883.14, 884.84, 885.82, 894.71, 912.93, 918.73, 972.25, 982.17, 988.98, 997.33, 1011.97, 1017.33, 1030.25, 1035.27, 1038.42, 1047.33, 1056.16, 1060.27, 1063.1, 1076.33, 1078.64, 1085.47, 1088.53, 1147.81, 1154.35, 1161.09, 1175.69, 1182.25, 1211.85, 1224.32, 1227.49, 1244.84, 1259.07, 1277.2, 1279.83, 1282.27, 1293.43, 1345.42, 1346.13, 1373.34, 1375.34, 1377.66, 1380.91, 1408.94, 1424.87, 1456.37, 1458.36, 1488.54, 1488.69, 1531.01,

1532.03, 1535.39, 1538.25, 1545.91, 1556.64, 1563.42, 1570.5, 1572.04, 1577.09, 1587.66, 1677.48, 1692.83, 1700.07, 1716.33, 1717.96, 1747.46, 1762.69, 3112.84, 3116.21, 3120.49, 3194.29, 3199.21, 3199.86, 3201.02, 3204.97, 3207.6, 3267.42, 3269.73, 3274.02, 3281.03, 3284.31, 3289.51, 3289.56, 3294.23, 3296.7, 3298.57, 3300.99, 3302.76, 3309.09, 3707.55

G

6.32, 7.5, 14.9, 18.54, 20.82, 25.5, 26.56, 28.04, 29.96, 34.07, 35.65, 36.92, 38.95, 41.01, 43.73, 48.06, 49.33, 85.22, 102.76, 104.85, 116.36, 120.78, 123.31, 127.12, 131.15, 134.44, 135.9, 137.27, 138.77, 142.45, 143.57, 146.64, 150.64, 152.95, 157.26, 161.37, 162.7, 167.14, 169.02, 174.88, 175.83, 178.45, 180.01, 183.81, 184.72, 208.25, 235.75, 243.49, 244.04, 248.81, 254.3, 255.67, 256.33, 282.48, 283.51, 284.14, 284.59, 285.13, 285.66, 287.89, 288.36, 292.23, 293.83, 294.28, 294.8, 296.08, 297.52, 312.53, 329.11, 329.41, 329.53, 330.41, 339.48, 344.33, 359.71, 361.27, 364.13, 364.66, 371.09, 373.89, 395.01, 403.15, 414.1, 424.1, 429.2, 430.33, 432.05, 432.33, 433.84, 434.27, 439.02, 440.74, 459.59, 459.62, 459.89, 459.96, 460.83, 460.99, 488.57, 494.61, 494.66, 495.81, 504.67, 506.21, 540.47, 554.84, 591.19, 592.26, 596.63, 597.78, 598.96, 599.29, 600.61, 603.22, 621.69, 629.35, 655.34, 657.06, 666.78, 667.98, 684.55, 685.77, 690.94, 691.3, 693.5, 693.69, 697.94, 698.45, 705.87, 706.88, 715.3, 716.0, 727.31, 753.86, 753.98, 763.94, 769.71, 776.95, 779.85, 780.43, 781.33, 801.78, 803.17, 809.25, 809.54, 819.51, 829.86, 871.51, 896.07, 898.32, 917.86, 1002.95, 1003.71, 1008.79, 1027.58, 1027.83, 1037.71, 1037.79, 1049.71, 1050.23, 1055.86, 1057.56, 1057.62, 1066.42, 1167.13, 1170.06, 1184.8, 1184.99, 1197.99, 1198.19, 1213.01, 1213.89, 1221.7, 1222.36, 1224.58, 1225.09, 1231.1, 1289.4, 1309.9, 1345.18, 1348.11, 1348.4, 1350.17, 1350.46, 1352.49, 1352.67, 1382.12, 1382.51, 1385.86, 1386.46, 1389.59, 1389.69, 1406.02, 1480.16, 1480.81, 1482.66, 1488.02, 1488.87, 1493.5, 1494.06, 1498.68, 1506.47, 1512.48, 1573.64, 1573.76, 1582.97, 1586.19, 1589.73, 1590.34, 1625.94, 1626.09, 1626.93, 1627.13, 1629.38, 1630.53, 1734.06, 1734.36, 1735.38, 1736.34, 1738.18, 1738.27, 1751.88, 1751.98, 1754.21, 1754.55, 1757.54, 1757.82, 1815.78, 1877.82, 3138.16, 3142.86, 3236.58, 3239.62, 3632.04

H

20.15, 26.77, 29.4, 34.0, 37.45, 39.36, 44.4, 51.82, 69.58, 107.17, 120.17, 124.89, 134.33, 136.59, 139.42, 143.36, 151.11, 158.75, 160.69, 166.13, 175.85, 180.42, 182.47, 188.45, 216.76, 245.93, 254.33, 256.37, 283.85, 285.08, 285.5, 290.82, 293.95, 295.32, 300.41, 329.59, 330.2, 339.82, 360.27, 364.04, 370.75, 396.62, 415.89, 429.65, 433.0, 434.22, 438.5, 459.9, 460.21, 461.01, 474.74, 495.36, 499.66, 509.69, 555.86, 587.37, 593.78, 597.43, 600.19, 601.89, 626.89, 655.18, 667.35, 678.05, 683.32, 692.71, 694.0, 698.36, 706.22, 718.16, 751.72, 756.65, 770.64, 772.85, 781.35, 802.42, 808.83, 829.8, 870.02, 893.16, 902.87, 991.49, 1001.42, 1023.72, 1037.5, 1047.96, 1055.35, 1059.29, 1066.31, 1167.47, 1180.91, 1192.81, 1212.85, 1219.97, 1222.6, 1227.56, 1245.36, 1294.44, 1344.98, 1347.97, 1350.32, 1352.65, 1379.88, 1382.45, 1385.51, 1388.01, 1436.25, 1480.75, 1487.5, 1491.87, 1493.6, 1518.19, 1534.81, 1573.55, 1584.16, 1588.48, 1624.6, 1625.53, 1628.34, 1734.77, 1736.57, 1738.31, 1751.54, 1753.63, 1757.07, 1806.66, 1993.61, 3152.37, 3154.9, 3218.8, 3227.73, 3657.84

I

71.65, 146.6, 410.5, 565.29, 569.1, 598.58, 655.16, 659.65, 726.94, 867.69, 884.86, 957.18, 1059.57, 1065.53, 1220.62, 1248.26, 1295.31, 1332.41, 1378.15, 1418.35, 1444.63, 1502.0, 1527.62, 1932.74, 1975.57, 3152.54, 3159.45, 3197.91, 3211.73, 3691.49

J

15.56, 19.64, 22.89, 25.46, 30.11, 35.33, 41.85, 44.12, 52.95, 58.86, 66.72, 76.31, 104.73, 108.23, 117.55, 124.66, 129.06, 133.65, 139.58, 141.46, 147.47, 154.32, 162.46, 168.52, 175.78, 178.55, 181.16, 190.91, 206.55, 246.12, 254.2, 258.11, 277.29, 284.18, 284.72, 285.12, 293.76, 294.27, 294.95, 324.21, 325.32, 329.87, 335.1, 340.41, 361.92, 362.49, 370.55, 378.35, 390.48, 407.0, 419.02, 421.66, 430.99, 433.16, 436.25, 438.66, 459.97, 460.56, 461.29, 479.87, 492.11, 495.79, 503.42, 518.78, 539.65, 564.97, 565.67, 596.77, 597.29, 599.13, 602.78, 603.28, 612.13, 633.48, 650.67, 654.22, 669.0, 685.89, 693.07, 693.67, 698.94, 703.32, 716.01, 725.26, 728.61, 756.39, 763.31, 776.8, 783.77, 800.74, 806.96, 808.57, 808.96, 816.5, 853.09, 891.64, 896.69, 901.42, 921.28, 982.97, 1000.08, 1007.78, 1024.2, 1032.56, 1041.83, 1043.62, 1048.6, 1050.11, 1054.63, 1060.28, 1085.54, 1097.35, 1140.61, 1149.94, 1165.95, 1182.46, 1203.19, 1212.45, 1216.14, 1218.06, 1220.72, 1221.8, 1223.51, 1240.01, 1277.53, 1329.22, 1349.58, 1350.14, 1353.93, 1359.41, 1367.08, 1380.2, 1383.23, 1389.38, 1392.73, 1402.34, 1426.3, 1483.0, 1491.26, 1494.03, 1528.63, 1545.49, 1574.12, 1577.46, 1585.12, 1589.13, 1598.74, 1627.01, 1627.69, 1629.74, 1692.54, 1693.74, 1723.95, 1735.54, 1736.62, 1737.95, 1738.66, 1754.8, 1756.54, 1758.34, 2408.29, 3273.03, 3279.8, 3282.07, 3287.37, 3292.96, 3293.53, 3298.84, 3301.73, 3307.65, 3734.02

J'

9.21, 11.72, 20.32, 27.35, 28.51, 31.95, 37.17, 39.31, 44.13, 48.14, 53.08, 70.53, 100.77, 114.58, 117.57, 125.19, 136.12, 136.95, 141.02, 146.62, 146.95, 152.39, 157.03, 166.66, 174.59, 180.32, 186.03, 195.09, 226.47, 246.38, 253.72, 254.78, 272.29, 283.51, 284.14, 284.35, 290.96, 292.36, 293.37, 298.04, 328.45, 328.81, 329.87, 342.69, 359.39, 362.92, 370.08, 397.61, 413.8, 424.48, 429.45, 430.14, 431.51, 432.78, 438.77, 459.49, 460.34, 460.9, 477.59, 495.28, 496.61, 508.45, 526.13, 541.4, 555.96, 572.96, 596.39, 598.29, 600.34, 606.0, 615.87, 621.74, 644.04, 651.09, 657.97, 672.02, 694.25, 695.27, 696.53, 697.75, 711.2, 726.11, 729.97, 753.05, 766.6, 772.58, 783.57, 788.78, 796.34, 801.68, 802.95, 804.39, 838.38, 872.35, 890.57, 904.7, 908.6, 965.86, 969.33, 985.77, 998.39, 1016.24, 1024.31, 1030.35, 1033.17, 1041.56, 1044.26, 1046.19, 1056.12, 1084.9, 1101.99, 1135.18, 1154.38, 1157.89, 1160.47, 1168.7, 1206.33, 1211.0, 1212.16, 1212.49, 1213.91, 1217.62, 1236.66, 1319.55, 1349.79, 1351.85, 1354.71, 1361.8, 1362.23, 1363.72, 1365.15, 1373.81, 1395.19, 1400.72, 1414.74, 1477.11, 1480.01, 1487.43, 1526.84, 1542.85, 1567.22, 1576.25, 1579.43, 1583.76, 1601.27, 1614.44, 1616.49, 1619.08, 1675.38, 1687.5, 1717.14, 1726.63, 1735.02, 1737.28, 1739.32, 1744.31, 1749.57, 1750.75, 2399.7, 3256.92, 3257.23, 3267.49, 3268.67, 3278.71, 3284.0, 3285.79, 3293.42, 3294.56

K

7.26, 16.62, 24.31, 26.49, 31.71, 35.73, 40.25, 40.76, 44.73, 52.78, 58.24, 72.54, 83.51, 108.19, 115.22, 121.45, 125.35, 135.14, 137.01, 143.53, 146.91, 157.57, 160.74, 162.15, 171.59, 176.38, 181.65, 198.67, 220.12, 242.76, 246.41, 254.97, 266.43, 283.3, 283.69, 284.84, 293.64, 295.38,

295.78, 312.33, 330.09, 331.56, 332.86, 346.86, 359.4, 363.29, 372.01, 378.28, 400.99, 406.97, 429.01, 429.91, 432.17, 435.48, 459.67, 460.38, 461.96, 462.94, 474.96, 480.06, 487.11, 497.34, 497.73, 505.68, 510.63, 568.12, 576.35, 592.03, 598.09, 600.58, 601.37, 636.06, 642.85, 657.42, 670.63, 678.03, 694.07, 696.01, 696.18, 697.87, 700.82, 707.86, 751.05, 755.87, 765.47, 775.16, 782.2, 788.46, 798.6, 802.14, 808.3, 830.96, 840.0, 855.13, 879.42, 896.1, 897.87, 918.99, 972.41, 980.94, 997.91, 1005.63, 1033.81, 1034.92, 1037.09, 1040.44, 1040.91, 1050.33, 1061.36, 1072.63, 1080.91, 1090.76, 1106.18, 1149.69, 1159.25, 1160.83, 1167.46, 1176.14, 1204.52, 1210.37, 1215.75, 1217.02, 1224.28, 1228.77, 1244.45, 1330.56, 1347.33, 1349.53, 1352.08, 1353.2, 1365.18, 1369.99, 1372.82, 1373.65, 1399.88, 1415.76, 1432.49, 1474.17, 1476.78, 1487.17, 1521.2, 1544.87, 1562.63, 1564.52, 1571.1, 1576.04, 1587.01, 1620.8, 1621.65, 1624.31, 1663.07, 1669.87, 1706.9, 1720.51, 1732.86, 1735.42, 1736.67, 1748.28, 1751.62, 1752.5, 1989.04, 3060.67, 3257.78, 3275.14, 3281.96, 3293.09, 3294.57, 3298.94, 3302.25, 3307.51, 3320.75

M

18.58, 64.93, 114.83, 184.97, 257.44, 301.84, 328.59, 340.93, 419.65, 449.97, 521.67, 544.13, 584.19, 600.52, 637.22, 648.32, 704.33, 710.09, 724.68, 782.48, 795.23, 812.34, 854.53, 867.19, 888.36, 905.64, 924.45, 973.18, 973.97, 988.34, 1025.95, 1027.11, 1042.34, 1051.27, 1069.27, 1087.3, 1120.0, 1145.28, 1170.56, 1206.84, 1216.85, 1244.37, 1251.11, 1288.22, 1353.72, 1364.97, 1378.39, 1408.97, 1415.79, 1445.64, 1530.39, 1549.85, 1567.31, 1586.06, 1679.07, 1705.33, 1712.17, 1732.27, 1736.94, 3265.71, 3269.24, 3272.36, 3279.45, 3281.25, 3288.7, 3290.71, 3297.24, 3297.79, 3344.64

N

42.99, 53.49, 55.36, 131.82, 136.41, 230.0, 245.65, 323.41, 329.76, 342.25, 402.5, 442.39, 472.46, 505.44, 514.91, 551.36, 571.61, 591.69, 634.14, 671.52, 689.26, 763.31, 798.04, 799.65, 829.38, 834.59, 860.91, 890.16, 907.27, 908.98, 1006.8, 1031.06, 1035.14, 1048.76, 1055.81, 1069.36, 1081.25, 1096.84, 1149.61, 1153.56, 1174.55, 1220.35, 1226.96, 1232.18, 1234.84, 1253.73, 1344.31, 1370.71, 1385.23, 1404.29, 1414.43, 1438.32, 1522.59, 1548.27, 1566.76, 1594.44, 1657.61, 1704.26, 1710.76, 1727.0, 1981.09, 3177.62, 3264.85, 3276.4, 3285.39, 3292.61, 3295.96, 3297.69, 3307.76, 3307.9, 3310.69, 3903.34

O

43.85, 61.13, 85.31, 186.4, 243.58, 273.76, 296.45, 354.25, 417.13, 429.73, 459.85, 515.39, 556.03, 572.53, 584.79, 622.57, 637.04, 688.46, 697.67, 726.83, 761.32, 777.01, 796.93, 802.32, 876.05, 892.94, 899.8, 904.63, 930.38, 957.62, 987.75, 1011.5, 1037.39, 1043.17, 1060.68, 1071.7, 1072.43, 1087.28, 1135.62, 1149.64, 1188.32, 1217.52, 1226.83, 1242.22, 1248.47, 1282.7, 1294.19, 1336.38, 1379.17, 1390.41, 1410.91, 1434.43, 1532.05, 1544.3, 1569.4, 1580.01, 1691.67, 1701.24, 1717.67, 1746.91, 1788.41, 3266.38, 3278.35, 3288.17, 3291.51, 3296.47, 3297.75, 3303.41, 3304.98, 3309.93, 3339.02, 3709.52

P

19.07, 22.16, 28.5, 32.13, 35.07, 39.3, 44.94, 46.65, 52.29, 59.88, 66.18, 71.17, 103.1, 115.54, 126.4, 130.3, 134.35, 140.43, 143.07, 147.5, 153.83, 157.12, 161.37, 166.67, 171.02, 173.12, 184.49, 189.65, 200.53, 251.09, 254.87, 261.08, 266.64, 270.53, 283.65, 284.88, 285.19, 292.7, 295.87, 297.89, 324.32, 329.2, 332.76, 333.4, 358.92, 361.66, 367.55, 380.5, 406.59, 410.26, 418.53, 430.01, 432.31, 435.02, 437.22, 445.39, 460.06, 462.67, 463.22, 473.18, 490.5, 492.33, 505.38, 506.78, 542.77, 557.04, 561.8, 597.55, 598.26, 598.8, 601.77, 608.34, 618.58, 643.37, 645.88, 663.64, 670.17, 682.88, 693.59, 695.09, 696.39, 702.01, 705.11, 711.67, 753.59, 757.09, 774.07, 779.89, 783.97, 796.95, 800.75, 804.79, 815.67, 822.34, 855.87, 887.1, 894.52, 897.67, 902.12, 983.25, 1004.54, 1011.18, 1012.64, 1035.25, 1041.63, 1042.75, 1044.87, 1046.11, 1052.43, 1068.1, 1080.68, 1106.73, 1146.59, 1153.55, 1164.13, 1172.78, 1183.46, 1211.93, 1216.22, 1218.4, 1219.96, 1223.13, 1224.37, 1232.27, 1242.69, 1324.4, 1346.53, 1347.72, 1351.29, 1366.81, 1373.25, 1375.68, 1375.86, 1378.41, 1385.75, 1407.65, 1412.19, 1478.78, 1482.46, 1491.32, 1525.94, 1547.87, 1568.19, 1571.8, 1580.74, 1584.55, 1598.29, 1623.09, 1624.98, 1628.13, 1680.52, 1706.71, 1712.3, 1729.31, 1733.45, 1735.42, 1737.37, 1751.66, 1753.96, 1755.22, 2048.6, 3248.25, 3277.6, 3281.62, 3291.72, 3296.91, 3297.22, 3308.85, 3324.95, 3329.21, 3924.87

Q

23.06, 28.48, 29.83, 33.84, 39.49, 44.33, 51.24, 52.11, 62.3, 63.94, 70.68, 80.16, 116.37, 118.92, 129.76, 131.13, 139.33, 144.75, 147.58, 155.47, 156.04, 164.25, 167.51, 177.28, 181.14, 185.9, 195.24, 202.14, 251.27, 253.25, 260.34, 275.14, 284.07, 284.33, 285.35, 292.94, 295.13, 296.84, 299.82, 302.55, 330.68, 331.76, 332.32, 360.96, 361.88, 368.58, 406.07, 406.77, 421.06, 428.65, 430.78, 432.96, 435.2, 439.19, 451.34, 460.53, 461.64, 464.14, 478.62, 494.04, 502.27, 505.65, 531.71, 579.89, 584.1, 597.83, 599.15, 601.49, 605.26, 616.95, 631.04, 647.33, 655.29, 664.0, 676.67, 689.23, 693.04, 694.55, 695.73, 703.75, 711.58, 726.99, 734.47, 742.1, 756.59, 761.14, 778.73, 791.29, 796.2, 799.9, 807.4, 809.18, 830.95, 862.64, 897.77, 908.86, 912.1, 921.25, 984.68, 989.76, 992.04, 1012.91, 1033.55, 1036.71, 1040.65, 1045.44, 1047.99, 1060.66, 1068.73, 1069.69, 1082.8, 1089.99, 1145.65, 1160.07, 1165.2, 1168.34, 1174.0, 1208.16, 1214.26, 1215.7, 1218.89, 1220.35, 1226.65, 1244.96, 1262.3, 1281.66, 1328.67, 1346.97, 1349.86, 1353.51, 1367.29, 1370.43, 1371.66, 1372.63, 1374.03, 1404.24, 1421.96, 1475.54, 1480.67, 1488.51, 1529.13, 1544.7, 1565.85, 1571.12, 1576.87, 1579.93, 1582.71, 1621.33, 1622.84, 1626.48, 1695.65, 1701.9, 1719.41, 1733.7, 1735.91, 1737.69, 1746.03, 1750.34, 1752.75, 1754.17, 1777.18, 3273.79, 3280.21, 3283.24, 3289.49, 3290.56, 3297.73, 3301.93, 3325.05, 3333.23, 3752.2

Q’

23.63, 26.96, 30.4, 33.6, 38.96, 45.04, 51.65, 53.24, 60.34, 70.25, 71.54, 81.58, 118.8, 119.63, 130.53, 130.97, 140.03, 144.15, 147.42, 155.79, 157.08, 164.92, 167.36, 179.13, 182.47, 189.0, 199.32, 214.05, 251.64, 253.46, 260.94, 284.11, 284.74, 285.53, 292.27, 294.25, 295.66, 297.71, 303.86, 326.58, 330.9, 332.15, 341.29, 361.48, 364.27, 368.97, 405.1, 407.22, 424.61, 428.01, 430.03, 433.37, 435.36, 443.49, 460.9, 461.6, 464.51, 468.18, 490.11, 496.15, 505.04, 516.18, 587.6, 596.5, 598.64, 601.34, 606.57, 613.68, 625.26, 642.79, 646.75, 660.79, 669.11, 690.45, 692.75, 694.38, 697.23, 703.41, 713.81, 728.44, 733.24, 744.24, 756.33, 761.18, 778.94, 790.14,

796.28, 799.93, 809.82, 818.52, 825.84, 871.74, 892.51, 905.64, 906.56, 929.27, 964.67, 976.93, 982.07, 985.05, 1026.2, 1030.54, 1032.8, 1039.55, 1040.09, 1042.85, 1047.26, 1075.29, 1082.86, 1105.42, 1136.62, 1148.05, 1154.27, 1158.07, 1164.58, 1173.06, 1201.79, 1207.4, 1209.09, 1210.57, 1211.35, 1241.41, 1278.59, 1347.76, 1349.04, 1349.89, 1353.65, 1357.67, 1358.79, 1360.21, 1361.8, 1367.44, 1390.52, 1400.66, 1437.65, 1473.35, 1476.34, 1483.06, 1526.22, 1552.5, 1560.36, 1561.82, 1574.05, 1576.64, 1580.94, 1615.48, 1617.27, 1620.22, 1680.51, 1698.61, 1700.28, 1730.94, 1734.26, 1735.68, 1736.89, 1737.49, 1747.46, 1748.92, 1750.72, 3256.57, 3256.84, 3267.37, 3271.41, 3277.94, 3284.59, 3285.61, 3304.15, 3337.15

R

15.91, 23.48, 28.56, 30.87, 34.54, 44.81, 53.63, 58.64, 64.91, 72.95, 77.28, 84.1, 116.35, 122.13, 127.34, 138.91, 145.85, 149.8, 153.01, 156.87, 162.72, 166.51, 170.8, 181.06, 186.41, 203.74, 204.45, 222.49, 246.83, 253.22, 261.34, 266.18, 282.12, 284.26, 284.91, 285.89, 292.46, 295.52, 298.07, 327.36, 331.91, 332.92, 335.55, 355.88, 361.32, 369.37, 400.78, 405.49, 424.69, 431.81, 432.5, 433.46, 435.49, 445.83, 459.95, 460.97, 463.8, 465.22, 489.61, 496.65, 506.09, 522.29, 544.13, 577.97, 596.79, 599.25, 602.0, 609.18, 617.96, 643.39, 649.84, 653.95, 666.35, 687.56, 692.76, 694.65, 695.48, 700.42, 709.53, 721.03, 732.22, 753.3, 755.68, 777.75, 794.95, 797.95, 801.97, 802.49, 808.44, 820.84, 840.92, 885.34, 888.67, 891.49, 910.98, 913.21, 965.23, 971.78, 988.19, 1025.47, 1029.68, 1033.74, 1040.82, 1044.69, 1048.51, 1049.91, 1051.48, 1056.46, 1063.94, 1072.5, 1093.12, 1134.42, 1150.2, 1161.28, 1174.06, 1182.36, 1196.2, 1212.42, 1217.22, 1218.78, 1221.1, 1224.09, 1247.78, 1250.03, 1273.08, 1301.68, 1344.46, 1347.83, 1350.89, 1352.04, 1372.36, 1375.51, 1379.29, 1395.61, 1408.58, 1414.78, 1436.19, 1457.82, 1465.45, 1482.23, 1494.74, 1539.66, 1546.24, 1565.88, 1579.1, 1585.97, 1588.41, 1596.2, 1622.16, 1624.13, 1626.48, 1670.3, 1712.11, 1727.28, 1730.55, 1733.52, 1735.81, 1736.3, 1747.31, 1751.82, 1753.21, 3173.9, 3263.5, 3265.72, 3277.12, 3279.13, 3287.09, 3296.19, 3299.1, 3307.49, 3336.69

S

20.07, 25.64, 31.18, 34.68, 40.22, 41.33, 51.12, 59.87, 69.05, 73.9, 80.65, 91.51, 110.84, 124.46, 127.77, 135.3, 136.17, 144.06, 148.14, 152.11, 159.14, 167.22, 168.41, 173.02, 182.12, 197.32, 204.79, 225.68, 255.95, 260.71, 262.06, 282.99, 284.55, 284.67, 294.53, 296.84, 297.8, 300.08, 311.02, 330.87, 333.08, 335.45, 337.15, 356.21, 363.63, 368.82, 377.99, 401.69, 413.96, 420.76, 430.59, 431.93, 434.16, 442.51, 458.42, 462.93, 464.0, 465.76, 489.85, 491.84, 499.23, 510.91, 546.74, 573.91, 585.89, 598.69, 600.01, 604.31, 605.5, 636.17, 643.29, 648.99, 662.22, 680.45, 689.04, 691.79, 694.68, 695.47, 704.4, 711.33, 718.11, 753.49, 758.75, 763.3, 771.13, 790.0, 794.28, 802.92, 803.67, 808.58, 842.54, 857.45, 872.58, 894.65, 910.48, 918.89, 930.98, 974.78, 999.24, 1004.02, 1006.27, 1034.18, 1036.25, 1042.68, 1043.85, 1046.75, 1048.83, 1054.83, 1065.01, 1077.02, 1087.27, 1141.45, 1157.67, 1164.76, 1170.45, 1175.13, 1182.31, 1211.42, 1212.32, 1218.36, 1219.8, 1223.18, 1240.06, 1254.35, 1265.96, 1308.27, 1343.55, 1346.21, 1349.76, 1353.74, 1375.27, 1376.99, 1378.26, 1378.9, 1416.16, 1425.8, 1451.35, 1472.62, 1477.22, 1491.31, 1509.8, 1543.24, 1551.8, 1564.23, 1569.1, 1578.77, 1580.65, 1603.18, 1624.98, 1627.0, 1629.06, 1695.82, 1712.9, 1721.58, 1730.99, 1733.53, 1738.83, 1748.34, 1750.1, 1752.98, 1756.97, 3224.24, 3278.4, 3280.53, 3288.46, 3292.85, 3295.2, 3302.1, 3304.33, 3313.37, 3315.78

T

17.72, 31.29, 37.59, 54.4, 74.47, 82.81, 94.43, 104.37, 119.44, 132.63, 192.45, 204.88, 219.94, 244.72, 277.62, 285.7, 287.86, 298.48, 319.64, 328.61, 351.93, 354.92, 360.06, 375.04, 414.74, 426.03, 431.93, 447.62, 465.47, 480.55, 482.78, 547.57, 559.35, 582.26, 585.07, 620.84, 632.48, 641.53, 650.0, 660.68, 703.39, 705.99, 742.75, 771.68, 781.65, 795.82, 804.78, 823.23, 846.15, 882.37, 883.41, 885.39, 887.71, 902.16, 919.28, 932.95, 972.04, 981.61, 996.69, 1000.69, 1011.23, 1020.28, 1033.69, 1038.66, 1041.56, 1055.55, 1055.71, 1071.35, 1071.55, 1077.07, 1077.98, 1084.16, 1096.88, 1135.04, 1154.89, 1157.17, 1164.18, 1177.25, 1181.94, 1218.63, 1228.58, 1231.01, 1253.38, 1259.1, 1260.92, 1275.9, 1279.96, 1302.61, 1344.58, 1354.41, 1370.95, 1378.11, 1382.91, 1420.12, 1428.76, 1447.04, 1455.89, 1458.6, 1488.57, 1489.92, 1525.21, 1532.98, 1535.58, 1538.21, 1541.9, 1556.49, 1557.07, 1563.84, 1570.23, 1571.88, 1583.23, 1607.33, 1665.92, 1683.85, 1710.77, 1714.39, 1718.05, 1749.96, 3112.91, 3115.84, 3121.37, 3193.81, 3196.42, 3202.79, 3204.01, 3207.9, 3209.38, 3226.36, 3264.08, 3278.3, 3280.87, 3283.37, 3288.48, 3292.3, 3295.76, 3298.68, 3301.83, 3304.06, 3304.97, 3310.23, 3311.04

U

23.05, 24.77, 31.75, 33.33, 45.22, 51.41, 66.01, 78.16, 83.7, 91.06, 125.59, 169.88, 183.05, 190.92, 229.58, 251.82, 271.44, 276.25, 295.24, 307.34, 316.5, 343.0, 353.36, 357.75, 372.21, 414.07, 416.45, 420.22, 423.0, 429.28, 445.35, 464.41, 467.48, 491.53, 531.94, 559.52, 571.35, 581.78, 610.65, 633.7, 638.83, 646.34, 659.42, 663.46, 715.9, 723.98, 727.11, 756.95, 777.72, 801.43, 803.66, 820.85, 824.53, 865.94, 867.91, 874.22, 883.03, 885.8, 945.57, 972.21, 982.69, 986.16, 998.04, 1007.17, 1010.4, 1020.12, 1024.3, 1038.5, 1040.83, 1042.47, 1043.72, 1051.29, 1066.36, 1072.09, 1078.69, 1085.0, 1085.57, 1087.14, 1138.41, 1143.29, 1151.84, 1153.9, 1173.64, 1194.74, 1217.63, 1226.22, 1242.95, 1243.65, 1265.8, 1276.82, 1280.61, 1290.0, 1312.77, 1334.86, 1344.51, 1380.17, 1380.68, 1381.89, 1392.36, 1411.23, 1411.57, 1429.93, 1457.64, 1459.69, 1489.79, 1491.24, 1533.0, 1533.07, 1535.32, 1537.93, 1538.3, 1546.02, 1557.09, 1564.16, 1572.38, 1576.97, 1587.14, 1589.31, 1684.02, 1692.84, 1709.81, 1711.1, 1714.35, 1725.13, 1979.8, 3111.73, 3113.83, 3116.22, 3121.26, 3176.27, 3195.82, 3198.63, 3201.84, 3203.16, 3206.57, 3208.13, 3267.55, 3267.81, 3272.73, 3273.46, 3277.46, 3277.78, 3283.62, 3287.36, 3288.35, 3291.5, 3299.1, 3299.55, 3303.17, 3305.47

W

16.4, 28.77, 36.18, 47.25, 59.05, 79.76, 84.92, 87.98, 109.66, 131.59, 149.55, 176.15, 204.5, 223.38, 243.35, 259.98, 274.03, 283.7, 287.01, 309.91, 333.47, 340.36, 355.7, 361.53, 374.7, 394.53, 421.1, 427.44, 430.86, 443.55, 460.52, 482.34, 497.61, 506.1, 521.81, 565.81, 583.84, 608.13, 613.2, 626.12, 639.22, 652.94, 662.7, 728.65, 738.34, 761.03, 771.55, 781.6, 785.23, 810.79, 828.35, 875.55, 881.86, 884.64, 886.78, 894.78, 916.47, 956.0, 971.59, 977.59, 981.29, 995.73, 1012.97, 1025.22, 1026.01, 1029.41, 1037.7, 1043.09, 1046.47, 1055.52, 1056.27, 1058.19, 1073.49, 1078.53, 1078.79, 1085.24, 1094.23, 1122.67, 1139.73, 1152.42, 1155.27, 1174.57, 1178.44, 1182.74, 1219.45, 1227.55, 1237.66, 1240.4, 1257.39, 1277.78, 1279.63, 1284.93, 1314.61, 1345.97, 1349.03, 1368.41, 1372.17, 1379.53, 1399.33, 1401.02, 1416.49,

1455.35, 1457.81, 1484.91, 1487.57, 1487.85, 1525.0, 1528.84, 1532.87, 1534.87, 1535.59, 1539.13, 1556.73, 1563.29, 1572.65, 1583.29, 1588.03, 1599.66, 1645.15, 1679.25, 1698.0, 1717.69, 1721.8, 1750.27, 1760.78, 2948.47, 3105.32, 3112.85, 3115.59, 3119.72, 3194.03, 3197.19, 3199.36, 3201.47, 3205.17, 3206.89, 3214.84, 3262.48, 3264.0, 3269.39, 3279.14, 3279.16, 3282.48, 3283.99, 3288.53, 3296.8, 3297.01, 3300.93, 3302.68, 3311.47

X

13.6, 17.87, 22.67, 24.36, 24.87, 35.98, 38.47, 41.25, 51.92, 58.44, 75.79, 78.24, 91.68, 95.54, 115.53, 138.72, 150.21, 176.08, 183.3, 190.86, 211.83, 243.65, 251.87, 253.36, 270.0, 283.24, 288.67, 307.7, 316.48, 328.08, 334.44, 347.32, 351.75, 354.84, 365.11, 371.28, 417.52, 420.36, 423.29, 426.83, 428.07, 442.59, 459.31, 465.02, 470.72, 491.5, 506.58, 511.2, 548.96, 551.3, 573.48, 577.52, 597.17, 610.28, 622.44, 626.93, 638.26, 655.71, 656.56, 661.29, 701.01, 708.98, 718.64, 720.52, 739.55, 762.7, 768.02, 775.95, 785.64, 800.48, 808.71, 847.01, 860.46, 873.82, 876.12, 883.21, 886.22, 888.7, 893.63, 897.7, 928.72, 939.46, 973.09, 982.55, 983.74, 998.19, 1013.33, 1014.3, 1017.61, 1031.25, 1033.59, 1042.71, 1043.78, 1046.82, 1049.61, 1061.82, 1061.95, 1065.48, 1079.49, 1084.28, 1085.3, 1097.2, 1106.97, 1140.88, 1146.8, 1151.57, 1168.04, 1173.31, 1186.03, 1195.95, 1204.49, 1218.75, 1224.12, 1228.72, 1246.34, 1255.13, 1268.92, 1277.22, 1280.19, 1282.55, 1307.48, 1319.91, 1345.64, 1367.76, 1376.07, 1381.17, 1383.25, 1385.01, 1389.73, 1390.38, 1410.53, 1416.76, 1458.76, 1459.27, 1469.65, 1470.65, 1487.32, 1489.91, 1491.88, 1532.17, 1533.83, 1536.0, 1538.27, 1538.83, 1540.76, 1542.63, 1557.49, 1564.74, 1572.77, 1573.93, 1586.06, 1588.09, 1588.25, 1682.21, 1689.39, 1690.29, 1692.4, 1709.68, 1712.79, 1717.74, 1720.27, 1977.1, 3114.19, 3117.46, 3120.96, 3123.82, 3196.36, 3199.62, 3201.01, 3201.63, 3204.49, 3205.77, 3207.22, 3223.66, 3257.53, 3269.72, 3271.22, 3271.86, 3272.45, 3274.93, 3277.16, 3284.83, 3289.3, 3291.78, 3292.07, 3296.43, 3296.49, 3303.19, 3304.1, 3304.33, 3328.8, 3602.67

Y

2.52, 15.8, 22.91, 31.58, 39.99, 40.93, 43.58, 62.45, 75.31, 82.38, 85.07, 97.82, 105.76, 117.23, 139.84, 147.77, 159.69, 175.71, 179.97, 199.37, 232.46, 236.7, 246.16, 267.11, 281.49, 284.22, 293.6, 298.44, 313.65, 315.64, 322.98, 347.27, 356.31, 361.28, 374.4, 402.49, 417.56, 423.83, 426.41, 429.28, 434.42, 447.01, 460.56, 468.56, 474.93, 484.53, 491.43, 518.14, 532.95, 546.68, 579.04, 598.01, 612.58, 619.72, 639.98, 644.46, 655.03, 662.09, 671.17, 687.21, 704.07, 717.12, 727.45, 736.29, 766.71, 780.35, 786.28, 805.25, 810.77, 843.77, 856.26, 864.98, 879.17, 879.9, 882.31, 885.12, 890.22, 924.5, 928.46, 971.72, 975.7, 981.8, 996.45, 1011.42, 1012.87, 1017.91, 1024.99, 1030.52, 1037.58, 1040.09, 1045.54, 1048.96, 1056.1, 1056.63, 1058.55, 1060.28, 1064.36, 1079.33, 1083.6, 1085.41, 1093.12, 1095.66, 1126.32, 1140.98, 1153.61, 1156.32, 1170.67, 1176.29, 1184.37, 1193.83, 1221.6, 1222.99, 1246.46, 1249.13, 1253.58, 1259.87, 1264.31, 1277.98, 1280.02, 1285.65, 1294.08, 1318.0, 1346.55, 1352.04, 1374.51, 1376.9, 1380.89, 1381.3, 1392.06, 1405.77, 1408.18, 1436.75, 1455.26, 1457.76, 1467.67, 1487.26, 1487.52, 1489.94, 1493.73, 1531.11, 1533.66, 1534.63, 1535.71, 1539.69, 1541.03, 1552.65, 1556.9, 1563.42, 1569.72, 1573.38, 1578.98, 1581.88, 1588.93, 1678.01, 1679.37, 1683.11, 1702.94, 1709.98, 1716.49, 1718.3, 1726.41, 1733.64, 3112.48, 3115.89, 3119.67, 3123.32, 3194.14, 3198.9, 3199.61, 3201.53, 3201.7, 3204.74, 3206.42, 3229.41, 3236.72, 3264.57, 3275.2,

3275.66, 3279.12, 3279.42, 3281.27, 3282.27, 3283.92, 3291.84, 3291.9, 3298.74, 3298.8, 3298.97, 3300.44, 3305.73, 3306.87, 3504.1

A-TS

-66.26, 9.1, 25.8, 29.45, 32.84, 34.3, 40.18, 44.05, 49.47, 55.36, 58.76, 63.83, 69.78, 94.63, 111.35, 119.63, 127.56, 133.51, 138.13, 142.85, 147.52, 153.27, 159.78, 167.23, 167.84, 170.69, 182.89, 192.1, 202.44, 225.87, 253.13, 258.43, 260.39, 283.43, 284.4, 284.7, 285.61, 292.14, 294.54, 296.58, 325.15, 330.89, 333.05, 341.63, 357.49, 362.23, 366.25, 377.53, 380.97, 402.16, 412.74, 421.26, 429.46, 430.62, 432.76, 434.25, 442.42, 459.28, 461.77, 462.88, 489.05, 490.63, 492.57, 503.54, 520.15, 535.69, 547.56, 569.63, 597.9, 598.76, 599.61, 603.09, 603.69, 619.54, 646.74, 648.43, 663.83, 679.74, 691.95, 692.85, 694.43, 696.43, 702.49, 718.07, 736.69, 752.06, 756.56, 770.75, 773.03, 792.92, 796.39, 806.94, 810.13, 814.87, 852.15, 882.83, 885.62, 896.13, 897.44, 979.55, 1002.26, 1003.51, 1025.4, 1026.2, 1036.93, 1041.68, 1044.78, 1049.27, 1059.24, 1063.94, 1082.8, 1108.81, 1145.0, 1152.15, 1168.08, 1186.09, 1200.85, 1213.77, 1216.1, 1221.05, 1221.41, 1223.15, 1224.21, 1238.34, 1241.67, 1333.15, 1347.52, 1347.87, 1351.41, 1369.49, 1374.41, 1379.91, 1382.02, 1384.97, 1387.94, 1407.16, 1407.66, 1484.2, 1487.05, 1494.58, 1527.62, 1546.35, 1571.28, 1577.2, 1585.73, 1587.24, 1602.38, 1625.11, 1626.33, 1627.92, 1686.08, 1705.11, 1716.67, 1730.11, 1733.42, 1736.0, 1737.47, 1752.52, 1754.83, 1756.1, 2221.93, 3245.89, 3276.03, 3281.75, 3288.15, 3294.59, 3297.45, 3303.79, 3328.19, 3328.93, 3926.43

B-TS

-307.09, 13.27, 14.12, 19.31, 22.24, 24.34, 26.83, 30.35, 30.71, 34.09, 37.89, 40.67, 42.82, 45.4, 49.11, 52.01, 55.65, 58.52, 65.24, 66.5, 81.13, 89.31, 101.93, 111.33, 120.7, 124.06, 127.7, 133.08, 138.5, 139.53, 141.59, 144.5, 150.23, 154.65, 157.46, 159.78, 163.9, 166.53, 178.31, 181.99, 183.28, 192.35, 203.72, 232.34, 239.07, 247.83, 249.18, 252.8, 256.96, 263.21, 275.51, 282.49, 283.63, 285.12, 285.98, 292.16, 293.62, 294.51, 295.51, 303.21, 311.03, 329.03, 330.04, 330.44, 337.48, 339.45, 360.62, 361.38, 363.83, 369.08, 369.98, 373.56, 376.99, 397.69, 403.31, 421.24, 422.78, 426.61, 432.38, 433.32, 434.51, 438.18, 438.95, 444.91, 460.09, 461.59, 462.43, 476.75, 483.22, 484.47, 495.95, 500.81, 503.29, 513.73, 521.76, 541.81, 565.77, 579.04, 582.96, 594.11, 597.85, 598.37, 599.19, 602.43, 606.27, 611.01, 617.23, 620.35, 636.98, 646.74, 652.92, 664.1, 668.26, 684.95, 694.11, 695.41, 700.19, 705.65, 715.28, 723.27, 727.9, 730.85, 754.96, 770.54, 772.54, 776.69, 779.61, 780.61, 789.41, 795.4, 803.09, 805.97, 807.31, 844.89, 853.25, 874.64, 876.81, 878.25, 886.87, 896.96, 898.59, 905.55, 907.46, 929.59, 972.73, 981.63, 989.73, 998.02, 999.45, 1001.21, 1008.67, 1014.95, 1020.85, 1025.49, 1036.17, 1041.14, 1043.99, 1047.21, 1051.52, 1054.49, 1060.57, 1063.89, 1068.16, 1070.52, 1082.24, 1085.25, 1086.5, 1105.22, 1147.13, 1151.58, 1156.51, 1164.02, 1172.97, 1178.1, 1184.29, 1188.57, 1206.96, 1209.99, 1214.11, 1217.18, 1218.12, 1218.35, 1223.19, 1239.11, 1247.08, 1263.63, 1278.69, 1279.57, 1288.1, 1324.08, 1338.47, 1338.92, 1347.3, 1348.15, 1350.87, 1353.64, 1365.4, 1373.24, 1374.96, 1375.87, 1376.6, 1377.65, 1378.27, 1381.03, 1388.16, 1407.42, 1409.92, 1454.31, 1456.16, 1480.72, 1483.58, 1487.13, 1488.51, 1489.14, 1498.79, 1525.13, 1528.96, 1533.56, 1536.04, 1536.13, 1543.64, 1545.66, 1557.37, 1561.99, 1571.86, 1577.19, 1579.97, 1581.53, 1583.51, 1592.21, 1597.73, 1621.55, 1623.31, 1625.4, 1682.25, 1691.4, 1702.36, 1704.54, 1718.6, 1719.41, 1728.94, 1734.73, 1737.52, 1737.71, 1751.44, 1752.37, 1755.51, 1927.37, 2211.76, 3108.14,

3115.85, 3125.66, 3148.98, 3155.31, 3190.75, 3193.73, 3198.11, 3200.08, 3204.69, 3214.42, 3220.07, 3223.78, 3248.57, 3271.33, 3271.69, 3275.51, 3279.94, 3285.47, 3286.16, 3293.98, 3296.03, 3298.11, 3299.03, 3302.08, 3310.42, 3924.63

C-TS

-249.57, 8.17, 9.32, 11.47, 15.15, 16.7, 20.98, 22.43, 23.3, 25.18, 27.13, 28.94, 31.14, 31.35, 32.96, 34.87, 37.12, 37.31, 39.07, 41.78, 42.07, 46.15, 48.66, 49.24, 52.77, 54.39, 61.81, 69.03, 72.93, 82.21, 89.23, 92.81, 102.45, 108.09, 109.45, 111.39, 117.0, 121.29, 124.6, 128.58, 128.91, 131.51, 136.83, 138.85, 140.78, 142.4, 143.18, 147.15, 149.59, 150.83, 154.94, 156.55, 159.09, 160.66, 165.47, 168.56, 175.5, 178.49, 179.59, 180.27, 184.46, 187.5, 190.27, 199.59, 214.09, 221.62, 227.0, 238.76, 240.21, 244.19, 246.91, 249.26, 253.62, 257.46, 258.92, 261.07, 272.0, 284.19, 284.5, 285.27, 285.62, 286.06, 286.14, 287.08, 290.24, 293.81, 294.11, 294.18, 294.82, 295.46, 296.58, 298.33, 312.9, 321.32, 329.56, 329.67, 330.6, 331.12, 332.51, 335.11, 337.85, 348.49, 358.05, 360.45, 362.83, 364.39, 365.25, 366.85, 370.34, 372.32, 374.65, 380.42, 396.71, 405.29, 417.18, 418.22, 423.07, 424.84, 429.36, 433.57, 433.86, 434.6, 436.42, 436.78, 438.2, 439.21, 441.89, 445.75, 446.06, 460.09, 460.28, 460.7, 461.11, 461.22, 461.64, 473.12, 483.24, 490.34, 495.51, 495.78, 497.24, 502.64, 504.89, 508.63, 510.64, 533.43, 548.98, 554.45, 575.75, 583.09, 594.34, 597.59, 597.96, 598.72, 599.17, 600.53, 601.02, 601.8, 606.3, 617.83, 622.51, 623.17, 638.43, 650.41, 652.78, 655.3, 658.46, 664.16, 670.02, 672.13, 687.03, 692.42, 693.58, 694.02, 695.0, 695.93, 698.53, 699.72, 706.77, 709.18, 718.27, 719.93, 723.29, 725.45, 754.36, 755.15, 770.62, 771.21, 776.19, 778.38, 780.04, 782.4, 785.39, 786.55, 796.12, 798.11, 801.49, 803.1, 807.17, 808.1, 808.64, 830.57, 849.86, 854.82, 875.12, 883.98, 886.01, 894.14, 895.14, 897.02, 904.05, 907.68, 909.03, 956.96, 972.86, 981.45, 981.82, 992.35, 995.63, 997.22, 1005.66, 1011.2, 1018.72, 1020.7, 1034.16, 1035.98, 1036.34, 1039.37, 1042.71, 1044.79, 1045.49, 1046.88, 1052.7, 1054.11, 1056.52, 1057.67, 1057.94, 1067.9, 1078.35, 1080.56, 1085.93, 1086.34, 1100.41, 1142.5, 1152.05, 1155.64, 1164.42, 1167.42, 1174.98, 1175.27, 1177.11, 1184.68, 1189.0, 1189.38, 1208.47, 1211.45, 1211.73, 1214.09, 1218.16, 1219.66, 1219.85, 1220.61, 1223.09, 1224.79, 1240.97, 1246.85, 1260.71, 1278.26, 1279.42, 1288.13, 1334.98, 1344.5, 1347.2, 1347.9, 1348.74, 1349.54, 1350.8, 1352.24, 1353.18, 1370.45, 1371.13, 1372.24, 1373.52, 1375.94, 1376.91, 1377.81, 1378.54, 1380.84, 1382.63, 1383.33, 1399.71, 1403.26, 1420.39, 1455.42, 1457.72, 1479.14, 1480.65, 1481.31, 1484.44, 1486.42, 1487.62, 1488.27, 1489.92, 1491.61, 1492.09, 1510.8, 1521.21, 1529.96, 1535.05, 1536.63, 1540.52, 1547.02, 1557.29, 1563.64, 1570.75, 1572.18, 1573.44, 1580.67, 1580.87, 1583.27, 1584.68, 1586.73, 1587.16, 1597.86, 1621.35, 1622.05, 1624.74, 1625.13, 1626.03, 1627.48, 1675.79, 1688.08, 1694.57, 1700.71, 1716.34, 1720.42, 1726.71, 1734.0, 1734.97, 1736.29, 1737.01, 1737.67, 1739.13, 1751.33, 1751.91, 1752.36, 1752.88, 1756.23, 1756.61, 1798.54, 2255.54, 3110.65, 3113.77, 3122.87, 3139.13, 3150.5, 3193.12, 3194.82, 3197.01, 3199.57, 3201.67, 3220.61, 3233.58, 3238.48, 3268.11, 3272.39, 3275.05, 3281.06, 3284.41, 3287.23, 3289.6, 3289.89, 3294.36, 3298.17, 3300.68, 3305.79, 3337.24, 3908.06

E-TS

-181.82, 8.83, 23.82, 39.46, 42.38, 49.88, 70.98, 82.71, 100.46, 109.65, 134.85, 153.24, 185.91, 219.62, 235.96, 258.22, 267.12, 283.05, 287.46, 291.02, 308.32, 327.35, 355.04, 358.71, 373.85,

405.52, 410.53, 422.81, 432.07, 457.28, 463.14, 472.92, 482.4, 496.3, 538.5, 569.14, 575.31, 586.12, 608.54, 613.38, 630.4, 641.12, 661.61, 696.09, 699.98, 771.53, 773.74, 780.97, 800.55, 816.79, 862.87, 879.75, 883.7, 887.15, 891.18, 892.73, 908.83, 958.55, 971.48, 982.1, 996.78, 1007.65, 1016.85, 1020.06, 1033.09, 1038.41, 1047.26, 1051.25, 1055.52, 1073.91, 1075.63, 1077.95, 1084.99, 1092.96, 1146.57, 1153.73, 1163.22, 1174.2, 1180.18, 1208.35, 1225.11, 1227.12, 1228.53, 1255.52, 1258.02, 1277.46, 1279.94, 1308.25, 1345.08, 1356.65, 1371.39, 1372.21, 1376.94, 1378.93, 1410.31, 1422.85, 1454.88, 1458.13, 1487.19, 1487.98, 1524.16, 1533.35, 1535.43, 1538.73, 1550.46, 1556.73, 1561.32, 1563.48, 1572.41, 1584.67, 1586.92, 1669.57, 1675.56, 1704.9, 1710.68, 1714.9, 1725.78, 1896.16, 3112.74, 3116.54, 3120.75, 3193.44, 3198.59, 3201.47, 3202.33, 3206.59, 3209.31, 3268.71, 3276.37, 3277.0, 3280.98, 3281.82, 3283.75, 3288.95, 3294.01, 3297.63, 3299.69, 3303.73, 3306.43, 3310.43, 3871.05

J-TS

-742.58, 13.5, 18.51, 21.8, 27.43, 32.4, 36.99, 41.07, 43.9, 51.89, 55.11, 61.63, 71.47, 100.45, 113.28, 120.88, 127.34, 131.87, 134.1, 139.09, 147.91, 152.06, 160.94, 162.71, 172.21, 172.23, 177.13, 183.48, 205.0, 221.46, 244.09, 252.04, 259.49, 280.13, 285.15, 285.74, 286.26, 294.88, 296.05, 298.18, 330.44, 332.65, 334.04, 343.6, 359.1, 363.59, 364.73, 378.39, 402.28, 412.95, 413.76, 429.17, 429.91, 432.78, 436.89, 459.98, 461.13, 462.35, 463.63, 481.09, 489.07, 496.55, 505.87, 509.89, 535.76, 587.44, 590.87, 594.87, 598.63, 601.05, 601.66, 634.15, 642.52, 649.62, 660.08, 673.46, 681.94, 693.25, 697.36, 698.26, 704.61, 709.97, 710.35, 751.67, 755.98, 759.0, 773.97, 782.58, 794.48, 801.32, 802.53, 810.74, 819.57, 845.62, 879.81, 896.32, 904.8, 918.25, 957.66, 986.4, 1003.02, 1007.77, 1013.6, 1026.03, 1040.42, 1041.43, 1042.75, 1043.69, 1049.18, 1054.17, 1072.92, 1079.5, 1086.52, 1146.82, 1148.3, 1164.32, 1175.14, 1183.47, 1208.48, 1209.79, 1214.23, 1219.89, 1220.88, 1225.01, 1243.03, 1319.07, 1346.53, 1349.27, 1350.41, 1367.03, 1371.09, 1374.31, 1375.53, 1377.96, 1378.69, 1393.3, 1416.49, 1477.77, 1479.74, 1491.6, 1526.23, 1552.15, 1554.95, 1567.41, 1574.7, 1578.99, 1586.56, 1622.47, 1623.9, 1626.07, 1678.07, 1679.16, 1711.22, 1719.76, 1733.38, 1736.25, 1736.93, 1749.58, 1752.42, 1754.12, 1933.44, 2186.77, 3277.81, 3280.0, 3287.81, 3291.49, 3298.3, 3299.74, 3304.63, 3312.22, 3319.53

J'-TS

-446.02, 14.44, 19.51, 22.12, 27.13, 28.86, 33.71, 35.98, 36.66, 44.31, 47.47, 50.62, 56.4, 57.79, 65.63, 70.4, 76.9, 78.57, 82.7, 97.53, 98.31, 112.33, 115.63, 121.56, 127.02, 131.16, 134.21, 141.6, 145.86, 147.83, 150.65, 156.99, 158.9, 163.91, 165.93, 173.07, 179.71, 186.21, 188.64, 196.18, 206.59, 254.0, 257.49, 261.26, 264.79, 274.14, 283.76, 284.23, 285.02, 289.43, 295.87, 297.07, 297.54, 330.44, 331.65, 333.27, 342.16, 355.82, 363.58, 364.95, 368.49, 369.45, 381.67, 398.85, 411.62, 415.71, 422.91, 427.71, 430.58, 433.63, 435.29, 438.59, 448.61, 459.54, 461.27, 464.52, 465.3, 485.04, 489.15, 494.8, 495.93, 505.93, 521.36, 529.69, 540.94, 565.33, 571.44, 577.6, 583.95, 593.0, 597.19, 599.78, 601.09, 603.0, 611.46, 621.34, 641.25, 646.01, 650.19, 651.68, 657.21, 666.88, 691.66, 694.98, 696.22, 698.69, 699.55, 705.82, 713.81, 727.84, 729.01, 753.8, 756.41, 765.61, 771.29, 779.57, 787.51, 795.84, 802.31, 803.36, 804.12, 805.82, 821.42, 824.55, 851.03, 860.24, 869.97, 893.04, 895.05, 895.65, 900.16, 909.43, 922.09, 929.93, 977.83, 983.07, 988.45, 1003.21, 1011.66, 1012.48, 1025.91, 1028.42, 1029.43, 1036.92, 1040.61, 1041.58,

1045.26, 1045.69, 1046.93, 1049.54, 1062.38, 1078.37, 1086.18, 1101.53, 1101.79, 1137.0, 1145.78, 1147.46, 1156.84, 1159.96, 1170.58, 1179.71, 1208.78, 1208.95, 1212.21, 1214.64, 1215.33, 1216.44, 1217.8, 1218.7, 1224.8, 1229.58, 1237.55, 1240.57, 1253.0, 1315.6, 1325.22, 1346.34, 1349.75, 1350.94, 1357.68, 1363.54, 1365.62, 1369.34, 1370.84, 1375.44, 1376.03, 1384.71, 1389.56, 1394.45, 1400.13, 1401.17, 1409.71, 1418.37, 1478.38, 1480.1, 1485.34, 1524.77, 1528.28, 1537.32, 1544.58, 1569.26, 1570.98, 1573.79, 1580.68, 1582.53, 1600.51, 1605.82, 1622.49, 1624.81, 1626.87, 1673.98, 1684.12, 1692.46, 1710.4, 1714.52, 1718.15, 1724.86, 1727.25, 1734.65, 1736.11, 1738.66, 1748.92, 1753.42, 1754.46, 2125.9, 2417.48, 3259.68, 3266.29, 3270.19, 3274.22, 3275.34, 3282.3, 3282.67, 3283.53, 3284.67, 3292.07, 3293.04, 3294.2, 3294.59, 3296.13, 3299.27, 3306.95, 3312.61, 3327.12, 3881.77

N-TS

-228.65, 46.9, 54.46, 65.6, 161.97, 233.68, 251.73, 277.71, 318.87, 357.69, 409.14, 451.19, 463.19, 523.9, 545.26, 570.72, 579.73, 589.4, 640.54, 662.63, 701.51, 763.07, 798.97, 814.56, 835.41, 844.06, 883.62, 894.43, 908.4, 919.81, 1010.5, 1020.48, 1038.6, 1052.25, 1053.56, 1074.91, 1079.03, 1085.93, 1148.45, 1154.96, 1166.75, 1206.82, 1224.1, 1230.45, 1233.43, 1258.46, 1306.44, 1355.44, 1374.01, 1388.86, 1420.17, 1424.41, 1525.59, 1552.9, 1565.69, 1582.51, 1669.47, 1704.19, 1712.56, 1727.16, 1917.73, 3238.31, 3280.98, 3284.98, 3286.02, 3290.68, 3294.46, 3295.68, 3304.74, 3307.29, 3308.42, 3861.14

P-TS

-216.85, 21.82, 27.96, 29.53, 33.39, 38.48, 43.13, 50.0, 52.16, 56.84, 62.09, 70.76, 78.26, 112.6, 118.93, 128.82, 132.05, 139.6, 145.73, 148.24, 152.24, 157.26, 162.07, 166.74, 173.93, 175.55, 183.79, 188.72, 201.0, 251.32, 253.68, 259.57, 272.43, 276.65, 283.91, 284.34, 285.33, 293.0, 295.52, 297.86, 318.27, 330.32, 332.39, 332.69, 361.18, 361.69, 368.83, 404.96, 408.78, 415.19, 428.24, 430.74, 432.98, 433.67, 436.8, 453.78, 460.92, 462.1, 464.38, 472.33, 487.59, 494.96, 501.66, 506.24, 562.84, 572.57, 590.25, 597.69, 598.6, 600.86, 602.41, 620.85, 632.54, 647.49, 651.91, 674.22, 679.58, 693.16, 694.77, 695.97, 702.03, 707.05, 718.51, 719.72, 755.67, 759.52, 776.14, 785.88, 796.61, 800.88, 805.49, 808.87, 823.53, 858.04, 876.02, 895.1, 903.52, 917.69, 961.07, 985.36, 1001.49, 1008.77, 1014.98, 1034.61, 1040.23, 1043.72, 1045.36, 1049.14, 1060.67, 1064.02, 1083.66, 1092.61, 1146.07, 1161.79, 1166.02, 1170.32, 1177.35, 1208.99, 1211.0, 1215.86, 1217.07, 1221.96, 1222.16, 1232.2, 1248.17, 1281.94, 1331.76, 1346.95, 1348.8, 1352.51, 1363.98, 1371.81, 1373.24, 1373.41, 1376.0, 1406.89, 1407.64, 1476.75, 1481.66, 1490.43, 1527.38, 1548.05, 1566.82, 1569.78, 1579.11, 1581.28, 1583.73, 1622.03, 1623.61, 1627.19, 1687.03, 1707.37, 1714.46, 1727.66, 1733.66, 1735.73, 1737.75, 1750.56, 1753.43, 1754.75, 1880.49, 3276.49, 3277.62, 3286.4, 3289.3, 3295.65, 3297.99, 3304.3, 3324.57, 3327.66, 3852.35

Q’-TS

-1027.58, 8.58, 11.31, 20.04, 25.36, 28.66, 29.09, 32.29, 37.25, 37.87, 43.56, 44.9, 49.41, 52.33, 56.98, 63.12, 70.64, 73.56, 83.38, 86.99, 104.6, 119.0, 119.51, 130.4, 132.31, 135.07, 141.84, 144.79, 147.71, 149.19, 151.88, 157.5, 159.29, 166.21, 170.07, 181.13, 185.66, 192.35, 212.34, 221.28, 243.69, 253.64, 255.7, 261.23, 273.0, 283.34, 283.97, 284.72, 290.32, 293.5, 295.13,

296.86, 307.03, 329.36, 331.84, 332.97, 352.92, 362.25, 365.59, 369.5, 380.63, 389.9, 405.22, 408.32, 415.31, 420.34, 423.61, 429.98, 433.82, 434.74, 436.12, 444.14, 460.73, 461.9, 464.3, 467.31, 470.31, 488.2, 489.73, 496.96, 505.62, 508.5, 531.85, 544.52, 567.51, 586.51, 595.65, 598.32, 598.59, 599.86, 601.82, 605.63, 610.36, 623.95, 638.9, 645.23, 649.69, 653.24, 662.49, 677.73, 691.97, 694.74, 695.58, 697.43, 703.75, 714.77, 715.46, 728.34, 732.39, 735.38, 756.53, 760.62, 767.03, 778.13, 781.34, 793.72, 796.02, 797.4, 800.57, 809.19, 815.2, 816.44, 826.26, 849.87, 859.37, 892.33, 896.42, 898.96, 900.16, 904.65, 916.72, 930.15, 978.22, 980.49, 981.79, 987.22, 1005.03, 1006.46, 1020.37, 1031.09, 1032.45, 1033.28, 1041.01, 1042.53, 1043.97, 1045.58, 1046.53, 1051.52, 1060.07, 1068.25, 1078.82, 1080.75, 1082.86, 1098.49, 1106.29, 1141.15, 1144.32, 1146.32, 1157.38, 1164.51, 1168.01, 1171.0, 1180.83, 1203.79, 1211.55, 1213.53, 1215.3, 1215.67, 1217.79, 1222.37, 1228.22, 1240.68, 1241.96, 1243.31, 1259.69, 1310.33, 1327.82, 1347.63, 1349.04, 1350.02, 1353.26, 1357.19, 1363.86, 1367.16, 1368.25, 1370.4, 1371.53, 1375.14, 1385.68, 1393.94, 1400.92, 1410.29, 1411.22, 1428.49, 1473.41, 1479.46, 1486.85, 1526.25, 1526.81, 1549.35, 1549.9, 1563.08, 1566.5, 1576.06, 1576.81, 1579.81, 1580.91, 1603.34, 1620.3, 1621.49, 1624.27, 1682.88, 1694.36, 1706.28, 1706.59, 1711.17, 1715.54, 1730.92, 1733.46, 1736.17, 1737.3, 1739.16, 1747.38, 1749.54, 1752.17, 1753.59, 2218.93, 3249.65, 3267.33, 3275.69, 3277.31, 3277.47, 3285.03, 3285.09, 3285.36, 3287.68, 3291.51, 3292.69, 3295.69, 3296.01, 3299.48, 3303.16, 3317.44, 3318.4, 3322.05, 3924.97

G-TS

-1696.5, 25.72, 29.03, 30.6, 32.09, 43.01, 44.31, 53.48, 58.63, 62.5, 69.52, 77.2, 80.48, 117.24, 121.62, 127.34, 135.62, 142.65, 147.72, 149.59, 157.86, 159.85, 167.75, 168.43, 178.28, 183.73, 191.18, 202.51, 203.87, 253.5, 255.93, 263.21, 271.45, 284.09, 284.45, 285.52, 292.99, 294.78, 295.48, 298.67, 313.93, 330.08, 332.04, 333.18, 360.01, 362.7, 368.97, 405.39, 407.62, 420.45, 430.93, 434.09, 435.46, 436.35, 441.62, 458.82, 461.35, 462.15, 464.24, 491.13, 494.87, 505.26, 510.38, 553.46, 577.58, 589.93, 596.86, 599.41, 601.95, 605.21, 624.95, 638.03, 651.87, 652.73, 662.94, 686.12, 692.28, 693.78, 695.82, 696.44, 710.4, 722.78, 728.62, 742.01, 756.78, 761.34, 781.28, 796.67, 801.05, 803.77, 809.14, 817.26, 833.82, 867.49, 895.26, 908.29, 916.35, 924.79, 987.77, 987.92, 990.18, 1029.1, 1033.18, 1034.8, 1042.14, 1046.2, 1048.68, 1058.55, 1064.28, 1068.79, 1076.32, 1088.92, 1144.95, 1161.14, 1169.19, 1176.0, 1183.22, 1191.23, 1209.34, 1215.64, 1217.85, 1220.34, 1227.06, 1244.15, 1254.71, 1312.55, 1346.84, 1350.53, 1353.55, 1356.81, 1371.77, 1373.23, 1375.59, 1377.18, 1379.76, 1404.17, 1441.86, 1475.21, 1481.88, 1490.96, 1531.9, 1547.69, 1557.95, 1566.76, 1572.0, 1581.05, 1584.59, 1622.03, 1623.65, 1626.89, 1634.08, 1695.91, 1699.47, 1723.96, 1727.44, 1733.3, 1736.22, 1737.85, 1751.93, 1752.76, 1754.56, 2258.36, 3275.26, 3282.66, 3283.02, 3290.8, 3295.13, 3298.47, 3305.5, 3313.21, 3321.02

R-TS

-676.29, 25.42, 27.84, 30.94, 34.47, 42.18, 45.37, 53.25, 58.85, 63.82, 73.9, 75.09, 91.82, 120.58, 121.83, 129.43, 134.97, 142.78, 146.71, 149.57, 157.3, 159.03, 166.44, 171.21, 179.42, 183.62, 190.7, 203.9, 221.29, 255.03, 256.5, 262.73, 283.77, 284.53, 285.25, 293.14, 296.01, 297.02, 299.85, 300.43, 326.91, 332.51, 333.51, 344.69, 360.92, 364.33, 369.77, 407.27, 412.2, 419.0,

430.25, 433.2, 433.77, 436.85, 445.46, 461.09, 462.01, 464.36, 467.21, 491.32, 495.98, 505.97, 523.6, 585.61, 591.72, 596.92, 600.78, 601.67, 605.8, 625.68, 643.67, 652.1, 666.87, 673.22, 692.56, 694.48, 696.01, 696.73, 703.71, 713.04, 723.43, 728.96, 753.9, 760.42, 779.29, 790.02, 796.3, 801.36, 805.86, 812.36, 827.29, 861.15, 890.94, 891.4, 912.89, 922.76, 930.76, 983.03, 986.08, 998.64, 1027.48, 1031.25, 1033.1, 1042.03, 1045.32, 1049.49, 1057.75, 1065.51, 1074.54, 1075.9, 1095.91, 1149.56, 1160.43, 1168.13, 1175.25, 1176.06, 1185.23, 1199.73, 1209.27, 1215.93, 1217.97, 1220.32, 1220.98, 1252.36, 1289.41, 1345.88, 1346.64, 1349.35, 1353.36, 1360.63, 1370.55, 1373.96, 1374.84, 1376.88, 1395.27, 1408.62, 1427.73, 1472.82, 1481.52, 1490.56, 1493.71, 1535.99, 1556.42, 1565.53, 1571.75, 1580.51, 1584.76, 1598.23, 1623.19, 1625.02, 1629.55, 1704.49, 1711.15, 1730.14, 1733.01, 1735.83, 1737.32, 1738.71, 1752.3, 1754.59, 1756.38, 2181.55, 3276.0, 3282.07, 3282.67, 3290.92, 3295.45, 3298.98, 3306.11, 3310.6, 3329.63

C'-TS

-213.3, 7.27, 9.12, 15.39, 17.91, 19.56, 19.73, 22.49, 23.93, 26.49, 28.94, 29.31, 30.94, 33.85, 35.24, 35.52, 36.45, 41.09, 42.31, 43.35, 46.52, 48.1, 49.84, 51.62, 54.16, 64.7, 77.93, 78.96, 89.45, 92.46, 93.65, 103.61, 106.76, 107.34, 116.1, 118.18, 121.27, 126.62, 126.67, 133.14, 136.41, 137.16, 138.75, 139.68, 141.22, 145.33, 146.63, 150.03, 150.74, 153.86, 159.72, 160.89, 166.02, 167.44, 173.84, 174.84, 177.84, 179.13, 181.65, 184.38, 188.34, 192.49, 211.7, 213.99, 218.96, 225.98, 241.11, 244.19, 250.05, 251.13, 254.55, 255.08, 259.41, 274.83, 282.85, 283.87, 284.19, 284.35, 285.01, 285.91, 286.41, 287.04, 291.83, 292.46, 293.69, 294.47, 294.52, 295.89, 296.98, 298.39, 303.2, 316.37, 319.85, 326.11, 329.51, 329.9, 330.18, 331.89, 335.77, 337.0, 344.85, 360.17, 362.09, 362.43, 363.86, 364.9, 365.28, 369.84, 373.24, 374.84, 380.05, 397.16, 407.17, 416.01, 416.56, 426.98, 431.63, 432.8, 433.39, 434.36, 435.17, 436.96, 438.6, 438.85, 442.68, 445.25, 452.93, 460.16, 460.32, 460.85, 460.86, 461.34, 461.94, 475.79, 483.45, 489.73, 495.07, 495.68, 497.02, 503.97, 505.3, 508.54, 547.53, 557.01, 579.42, 582.17, 594.97, 597.37, 597.8, 598.0, 598.41, 599.18, 600.59, 601.68, 601.94, 616.63, 626.6, 635.07, 636.29, 646.9, 647.35, 654.11, 655.18, 663.92, 668.93, 670.06, 687.23, 691.35, 691.74, 693.01, 694.32, 697.98, 698.04, 700.23, 704.37, 706.13, 708.03, 709.36, 717.34, 717.51, 719.04, 754.15, 756.4, 769.41, 772.23, 778.65, 780.01, 781.3, 783.98, 786.41, 793.45, 796.98, 798.99, 802.41, 807.72, 808.17, 808.72, 814.54, 830.11, 851.6, 855.34, 880.27, 880.94, 885.76, 891.36, 893.95, 904.34, 907.55, 911.15, 918.03, 937.23, 956.77, 966.66, 972.17, 981.17, 982.05, 994.48, 995.74, 995.77, 1003.61, 1018.78, 1020.39, 1020.65, 1031.48, 1036.56, 1037.37, 1041.19, 1041.97, 1042.9, 1044.41, 1046.58, 1048.39, 1053.92, 1054.56, 1057.48, 1059.85, 1065.5, 1071.99, 1076.38, 1079.1, 1085.45, 1085.48, 1119.31, 1152.97, 1155.17, 1164.63, 1166.73, 1167.27, 1176.39, 1176.65, 1177.16, 1179.98, 1188.92, 1190.05, 1211.4, 1211.89, 1212.76, 1215.81, 1217.82, 1219.79, 1220.67, 1222.87, 1225.07, 1244.27, 1251.7, 1258.13, 1269.15, 1276.58, 1279.81, 1288.96, 1325.69, 1334.71, 1347.58, 1348.01, 1348.08, 1349.72, 1350.63, 1352.6, 1352.71, 1358.34, 1371.54, 1372.89, 1374.78, 1377.4, 1377.97, 1378.32, 1379.05, 1383.06, 1383.49, 1386.14, 1414.61, 1426.2, 1445.74, 1453.44, 1457.21, 1475.72, 1480.87, 1482.03, 1484.35, 1485.35, 1487.34, 1488.5, 1489.56, 1491.69, 1492.29, 1509.08, 1517.71, 1521.28, 1534.3, 1535.61, 1539.77, 1547.39, 1555.61, 1556.6, 1562.68, 1571.73, 1572.32, 1572.79, 1574.13, 1580.68, 1583.42, 1584.47, 1585.04, 1585.7, 1616.76, 1621.11, 1621.51, 1624.68, 1624.97, 1626.24,

1627.58, 1670.61, 1691.46, 1696.7, 1714.8, 1717.09, 1722.39, 1733.98, 1734.5, 1736.28, 1737.07, 1737.37, 1737.83, 1742.65, 1749.77, 1750.26, 1751.95, 1752.28, 1756.34, 1756.86, 1802.12, 3110.45, 3114.18, 3122.71, 3144.1, 3150.53, 3190.61, 3194.45, 3197.54, 3199.18, 3202.2, 3223.35, 3229.11, 3234.5, 3268.25, 3274.87, 3283.51, 3285.6, 3290.74, 3294.57, 3297.93, 3299.38, 3299.95, 3303.21, 3304.27, 3307.66, 3318.06, 3336.6

B'-TS

-123.57, 13.35, 17.63, 19.81, 23.97, 26.36, 28.8, 29.48, 31.36, 35.82, 36.28, 38.21, 42.86, 43.43, 46.79, 52.12, 58.52, 61.86, 64.74, 76.93, 81.94, 94.59, 96.45, 105.29, 110.74, 116.61, 117.5, 120.97, 129.3, 138.06, 139.52, 142.71, 145.55, 147.5, 149.7, 152.1, 155.37, 167.37, 175.35, 177.64, 180.51, 185.22, 190.01, 194.6, 214.89, 228.3, 241.92, 248.74, 251.96, 254.18, 257.66, 279.0, 283.94, 284.23, 284.97, 287.14, 289.41, 292.31, 293.82, 296.73, 297.96, 301.25, 329.73, 330.27, 332.55, 334.94, 345.26, 361.89, 362.74, 363.5, 367.34, 370.29, 373.11, 392.2, 400.63, 417.49, 423.02, 431.5, 432.26, 433.53, 434.11, 435.32, 437.39, 439.37, 453.58, 460.39, 460.8, 462.62, 467.41, 478.16, 483.04, 485.25, 496.93, 499.66, 511.95, 528.21, 535.31, 567.66, 577.15, 579.83, 587.22, 595.67, 598.93, 599.22, 601.37, 603.63, 620.3, 629.98, 640.38, 645.38, 649.16, 662.15, 663.36, 687.5, 694.28, 696.94, 698.43, 699.24, 702.33, 710.11, 714.72, 716.68, 729.99, 749.64, 758.03, 768.12, 775.35, 779.59, 782.02, 796.26, 803.77, 805.55, 806.32, 808.74, 826.28, 849.5, 876.11, 881.68, 884.52, 888.01, 892.85, 899.05, 902.32, 930.36, 952.24, 972.66, 981.8, 991.34, 996.24, 999.72, 1005.47, 1007.16, 1017.2, 1018.42, 1032.17, 1036.09, 1036.83, 1037.99, 1040.81, 1042.72, 1043.04, 1050.14, 1053.23, 1059.57, 1059.79, 1062.46, 1064.11, 1068.3, 1080.91, 1084.19, 1085.65, 1086.65, 1138.96, 1146.77, 1151.21, 1156.33, 1161.03, 1174.72, 1177.14, 1177.4, 1185.15, 1209.17, 1213.18, 1213.29, 1213.65, 1220.47, 1223.64, 1239.73, 1240.69, 1260.78, 1277.7, 1280.78, 1291.04, 1298.64, 1318.53, 1326.72, 1341.41, 1343.92, 1348.46, 1349.09, 1351.22, 1354.12, 1367.19, 1368.62, 1372.23, 1374.76, 1376.31, 1377.49, 1377.92, 1379.19, 1409.36, 1411.34, 1440.44, 1456.67, 1457.41, 1479.8, 1482.5, 1486.93, 1487.94, 1488.76, 1498.37, 1527.04, 1528.09, 1533.04, 1535.79, 1537.78, 1541.11, 1545.26, 1546.5, 1557.08, 1563.38, 1570.75, 1572.53, 1574.81, 1579.42, 1583.48, 1589.23, 1589.95, 1620.77, 1622.62, 1624.55, 1678.48, 1683.18, 1692.08, 1707.86, 1712.19, 1717.39, 1725.97, 1735.25, 1736.9, 1737.68, 1751.22, 1753.0, 1755.73, 1921.65, 2152.58, 3095.8, 3111.89, 3112.97, 3117.63, 3151.67, 3168.92, 3175.55, 3190.73, 3194.31, 3197.1, 3201.91, 3205.16, 3207.58, 3207.92, 3232.88, 3267.05, 3268.67, 3274.84, 3276.6, 3279.13, 3283.22, 3283.43, 3287.68, 3288.75, 3295.09, 3295.46, 3303.38, 3306.0, 3312.73

C''-TS

-249.59, 7.24, 9.43, 11.79, 15.17, 17.92, 18.95, 22.53, 23.47, 26.01, 28.05, 29.51, 30.6, 31.29, 32.21, 34.66, 35.44, 37.95, 39.38, 41.66, 42.66, 44.77, 45.67, 50.15, 51.87, 57.34, 57.81, 61.48, 63.9, 79.96, 89.38, 92.78, 98.45, 106.83, 110.57, 116.06, 118.56, 119.96, 120.78, 126.05, 128.85, 129.76, 135.41, 137.51, 138.86, 140.77, 142.33, 142.5, 146.2, 148.53, 150.17, 150.89, 156.54, 159.33, 161.98, 165.37, 167.72, 174.2, 175.81, 177.47, 180.35, 182.62, 184.97, 187.59, 206.23, 209.99, 216.32, 224.76, 235.47, 245.78, 248.71, 251.35, 254.88, 256.3, 257.22, 260.08, 264.9, 282.95, 283.93, 284.43, 284.71, 285.3, 285.53, 285.77, 286.24, 288.17, 292.28, 293.32, 294.18, 294.51, 294.76, 295.72, 298.35, 315.1, 319.97, 328.9, 329.19, 330.41, 331.63, 332.9, 336.42,

343.34, 358.8, 359.64, 361.8, 362.56, 363.91, 364.94, 367.73, 370.81, 372.74, 377.54, 386.15, 397.44, 408.05, 416.93, 425.0, 428.63, 429.23, 431.49, 433.6, 434.11, 434.7, 435.24, 436.1, 437.91, 440.03, 442.38, 444.76, 452.58, 459.95, 460.49, 460.52, 460.62, 461.47, 462.27, 462.45, 477.3, 485.37, 489.66, 494.75, 496.06, 497.16, 504.64, 508.51, 531.32, 539.82, 546.05, 565.51, 577.28, 585.01, 590.51, 597.65, 598.29, 599.12, 599.39, 601.38, 601.87, 604.1, 613.72, 621.96, 628.02, 637.08, 640.12, 646.85, 652.23, 656.4, 660.68, 664.44, 665.13, 668.81, 687.61, 689.72, 693.19, 695.08, 695.51, 697.07, 697.64, 698.81, 707.19, 708.05, 714.22, 717.59, 718.98, 728.16, 748.18, 755.3, 758.64, 767.75, 769.13, 774.44, 780.49, 780.95, 781.07, 784.1, 799.93, 800.37, 803.3, 806.74, 808.17, 808.59, 809.92, 825.7, 826.39, 851.05, 871.43, 884.47, 885.64, 893.97, 897.69, 899.76, 902.71, 906.71, 946.33, 947.52, 971.93, 982.37, 995.81, 996.48, 996.85, 1000.27, 1003.56, 1016.48, 1017.43, 1019.81, 1026.92, 1031.5, 1036.87, 1037.47, 1040.57, 1040.92, 1041.85, 1042.14, 1043.2, 1046.2, 1053.98, 1054.65, 1056.7, 1059.06, 1060.8, 1068.71, 1069.13, 1076.97, 1079.75, 1084.89, 1086.0, 1086.95, 1144.26, 1144.44, 1153.89, 1155.6, 1164.55, 1167.76, 1177.61, 1179.22, 1182.82, 1183.59, 1185.78, 1190.76, 1212.57, 1212.85, 1213.44, 1217.06, 1217.8, 1220.17, 1220.78, 1222.9, 1231.18, 1239.45, 1245.15, 1260.24, 1275.91, 1280.52, 1291.12, 1312.09, 1329.51, 1338.87, 1347.51, 1348.15, 1348.43, 1349.13, 1349.76, 1350.54, 1352.14, 1353.56, 1372.2, 1373.52, 1374.63, 1375.69, 1378.29, 1378.89, 1379.06, 1379.97, 1383.67, 1384.45, 1386.23, 1408.58, 1412.52, 1442.45, 1456.76, 1457.08, 1457.8, 1479.01, 1481.26, 1483.76, 1485.57, 1487.07, 1488.05, 1488.44, 1491.36, 1493.97, 1507.06, 1516.92, 1528.09, 1529.75, 1532.41, 1534.83, 1541.28, 1545.34, 1557.13, 1562.85, 1572.38, 1574.41, 1575.24, 1576.18, 1581.35, 1584.06, 1584.79, 1586.45, 1587.6, 1592.64, 1623.7, 1623.84, 1624.65, 1625.41, 1626.27, 1628.06, 1673.76, 1684.75, 1708.9, 1711.27, 1714.1, 1715.42, 1728.39, 1734.01, 1734.93, 1736.55, 1736.61, 1737.2, 1738.79, 1751.11, 1752.39, 1752.54, 1755.03, 1756.88, 1757.83, 1815.55, 2233.71, 3106.93, 3113.19, 3114.93, 3119.79, 3149.08, 3160.99, 3188.12, 3194.8, 3196.21, 3199.32, 3202.9, 3205.33, 3206.68, 3225.69, 3244.09, 3264.7, 3273.56, 3274.55, 3279.51, 3279.88, 3285.1, 3286.7, 3292.32, 3294.14, 3295.5, 3296.84, 3301.53, 3304.74, 3309.59

U-TS

-150.98, 9.15, 14.89, 21.15, 27.44, 43.87, 52.21, 58.56, 62.48, 67.65, 99.31, 124.71, 144.79, 192.7, 193.68, 220.76, 246.25, 264.33, 280.72, 290.15, 304.65, 318.9, 341.7, 354.93, 362.08, 368.78, 409.87, 411.03, 422.82, 427.5, 434.76, 449.51, 476.86, 482.52, 504.99, 523.23, 531.45, 575.08, 582.69, 605.8, 618.85, 640.41, 645.52, 659.78, 710.21, 724.96, 744.16, 764.6, 781.08, 795.97, 806.15, 811.54, 827.37, 870.91, 874.43, 882.59, 883.29, 890.51, 953.52, 972.92, 982.44, 996.78, 998.16, 1012.15, 1012.7, 1022.87, 1030.02, 1040.19, 1041.2, 1043.35, 1046.31, 1053.03, 1072.19, 1072.91, 1079.02, 1082.47, 1084.7, 1084.97, 1144.55, 1148.04, 1148.99, 1154.24, 1174.57, 1187.24, 1218.05, 1226.5, 1238.82, 1239.28, 1261.69, 1276.69, 1279.86, 1292.51, 1308.61, 1333.91, 1344.05, 1376.14, 1376.89, 1377.42, 1382.87, 1408.5, 1412.34, 1439.06, 1458.77, 1459.29, 1490.39, 1490.47, 1528.86, 1533.34, 1535.62, 1538.04, 1541.84, 1548.91, 1557.3, 1564.41, 1569.16, 1572.22, 1584.42, 1588.98, 1672.65, 1682.3, 1707.87, 1708.38, 1709.55, 1723.41, 2077.39, 3082.17, 3114.89, 3118.36, 3121.89, 3183.63, 3196.71, 3202.31, 3203.4, 3204.84, 3206.89, 3209.04, 3262.98, 3265.42, 3268.18, 3275.81, 3279.76, 3283.65, 3285.47, 3286.08, 3289.58, 3294.41, 3300.28, 3301.09, 3302.82, 3306.69

B''-TS

-196.7, 9.84, 14.5, 17.62, 19.38, 21.31, 22.69, 25.81, 28.64, 30.24, 32.96, 34.14, 34.33, 38.32, 40.17, 42.22, 47.05, 50.27, 52.45, 53.88, 56.74, 61.53, 66.47, 72.81, 79.81, 84.09, 86.07, 103.75, 111.72, 114.22, 116.39, 122.31, 125.08, 125.83, 127.88, 132.63, 139.87, 143.77, 146.17, 149.31, 153.57, 161.53, 164.43, 167.79, 175.68, 179.25, 179.82, 181.51, 189.64, 193.27, 198.78, 224.05, 231.91, 241.21, 247.69, 253.1, 254.31, 257.23, 260.2, 277.17, 282.53, 283.43, 284.09, 285.65, 289.51, 293.09, 294.27, 297.18, 299.34, 302.56, 312.31, 327.14, 329.35, 332.17, 339.63, 340.53, 346.26, 352.62, 355.31, 362.89, 363.37, 369.3, 371.1, 380.25, 398.4, 404.04, 418.86, 422.05, 425.03, 427.32, 427.92, 428.62, 429.78, 433.03, 438.78, 446.38, 450.97, 459.37, 463.06, 464.14, 473.11, 481.8, 484.26, 495.12, 496.85, 500.1, 501.96, 512.73, 530.38, 535.0, 553.49, 562.55, 577.33, 583.98, 587.34, 589.36, 594.42, 596.25, 599.16, 600.3, 601.27, 604.37, 621.93, 626.18, 629.84, 648.02, 649.71, 652.14, 659.23, 662.0, 662.89, 686.89, 689.0, 691.45, 694.36, 701.62, 704.95, 707.34, 717.6, 724.12, 735.72, 744.11, 754.7, 772.69, 774.49, 775.44, 775.81, 783.95, 790.84, 803.33, 804.15, 805.28, 807.02, 845.75, 846.54, 860.46, 864.1, 875.79, 877.61, 882.05, 886.01, 887.12, 888.32, 890.36, 897.2, 921.6, 924.11, 941.94, 971.93, 977.65, 981.3, 989.87, 995.85, 1007.27, 1011.41, 1015.3, 1016.52, 1027.35, 1028.1, 1030.33, 1037.86, 1039.56, 1043.34, 1044.22, 1048.14, 1051.89, 1052.35, 1056.58, 1056.71, 1061.74, 1063.98, 1073.1, 1080.15, 1085.48, 1086.44, 1098.71, 1100.62, 1144.37, 1149.27, 1155.54, 1158.49, 1161.03, 1171.66, 1179.5, 1179.99, 1183.25, 1185.27, 1203.64, 1211.49, 1212.56, 1214.78, 1218.38, 1218.76, 1220.97, 1226.11, 1242.71, 1253.71, 1261.61, 1278.43, 1279.06, 1281.61, 1289.83, 1292.19, 1309.41, 1335.31, 1348.11, 1348.33, 1349.28, 1350.37, 1352.87, 1365.74, 1367.89, 1368.68, 1373.09, 1374.88, 1375.54, 1377.53, 1378.41, 1379.57, 1383.49, 1388.7, 1404.5, 1428.24, 1455.66, 1457.21, 1468.97, 1474.53, 1479.47, 1481.77, 1485.61, 1486.72, 1490.51, 1494.52, 1495.1, 1523.31, 1530.6, 1533.67, 1536.32, 1537.36, 1540.09, 1540.69, 1541.82, 1546.08, 1556.56, 1563.32, 1571.05, 1572.99, 1574.27, 1577.55, 1582.37, 1586.61, 1590.47, 1591.83, 1623.05, 1624.47, 1626.75, 1679.9, 1682.82, 1690.25, 1693.74, 1696.59, 1717.23, 1721.73, 1722.44, 1724.77, 1735.79, 1737.24, 1738.85, 1751.67, 1752.6, 1756.61, 1934.92, 2190.44, 3111.54, 3113.38, 3117.06, 3117.84, 3151.43, 3169.35, 3188.15, 3192.87, 3194.67, 3196.34, 3199.68, 3202.15, 3207.52, 3207.95, 3218.14, 3223.85, 3261.37, 3265.86, 3269.17, 3269.65, 3271.9, 3279.8, 3281.16, 3282.82, 3288.53, 3288.81, 3294.34, 3294.94, 3296.76, 3300.31, 3301.51, 3322.4, 3325.04, 3616.87

C'''-TS

-290.15, 4.4, 11.16, 12.61, 14.24, 16.06, 18.06, 18.44, 21.32, 23.41, 27.41, 28.19, 28.97, 29.52, 30.05, 30.4, 32.69, 35.0, 35.22, 38.34, 39.96, 43.94, 45.46, 48.03, 48.84, 49.88, 53.52, 57.09, 59.41, 62.78, 66.2, 67.97, 72.34, 79.27, 87.63, 91.65, 94.08, 106.46, 107.67, 112.78, 114.02, 115.79, 119.57, 125.21, 128.3, 129.75, 132.37, 133.35, 137.08, 139.02, 140.66, 141.29, 142.22, 146.02, 147.06, 150.31, 153.52, 155.84, 159.24, 161.85, 162.64, 169.36, 171.14, 175.87, 178.49, 180.62, 181.78, 182.71, 183.99, 189.87, 193.52, 198.51, 212.38, 223.59, 226.51, 232.81, 238.1, 243.0, 249.2, 251.57, 255.3, 259.79, 260.87, 262.22, 273.8, 280.7, 283.55, 284.48, 284.66, 284.75, 285.54, 285.86, 286.68, 292.78, 293.42, 294.72, 294.95, 295.61, 297.34, 297.82, 298.33, 299.3, 307.51, 323.68, 328.89, 330.88, 331.76, 333.34, 334.03, 335.53, 340.8, 342.64, 347.43, 354.69,

355.99, 359.02, 362.93, 363.32, 363.73, 366.39, 369.1, 371.98, 375.71, 384.6, 387.04, 398.11, 408.38, 421.05, 422.73, 424.88, 427.57, 431.43, 432.47, 433.59, 433.83, 434.63, 435.2, 435.75, 437.57, 438.43, 446.57, 447.78, 455.82, 460.13, 460.95, 461.16, 461.56, 462.35, 465.09, 472.8, 484.33, 485.56, 488.65, 494.14, 495.79, 497.2, 504.69, 507.47, 508.57, 522.36, 530.36, 536.86, 547.43, 563.07, 571.48, 577.96, 585.22, 591.5, 596.16, 597.28, 597.99, 598.3, 599.58, 600.76, 601.29, 610.26, 610.95, 616.59, 623.42, 640.09, 648.29, 651.13, 651.6, 655.53, 660.73, 662.54, 664.0, 670.66, 682.51, 688.13, 693.15, 693.26, 694.42, 695.43, 697.59, 698.05, 701.66, 703.7, 707.84, 709.62, 715.97, 721.2, 727.92, 744.25, 753.07, 759.66, 761.74, 765.79, 771.9, 772.66, 777.04, 779.0, 781.72, 782.78, 796.73, 799.26, 803.13, 804.03, 805.39, 807.41, 808.67, 818.55, 844.76, 848.61, 859.92, 863.05, 878.14, 880.69, 885.09, 885.55, 889.93, 890.74, 891.69, 902.97, 909.32, 927.04, 940.9, 945.55, 972.48, 981.0, 981.48, 992.88, 996.43, 999.53, 1008.18, 1009.56, 1013.3, 1016.54, 1024.53, 1028.67, 1033.47, 1035.04, 1036.49, 1039.14, 1041.44, 1044.03, 1044.27, 1044.37, 1045.52, 1047.43, 1048.31, 1053.11, 1054.68, 1057.88, 1062.97, 1074.95, 1079.79, 1083.78, 1084.65, 1085.31, 1099.13, 1105.53, 1145.46, 1147.56, 1151.82, 1153.35, 1161.44, 1163.61, 1172.9, 1177.58, 1178.98, 1184.08, 1185.55, 1186.23, 1188.9, 1206.59, 1209.14, 1210.48, 1212.24, 1214.63, 1215.07, 1217.13, 1218.19, 1222.57, 1225.85, 1241.44, 1242.72, 1254.12, 1266.91, 1277.85, 1280.07, 1281.65, 1291.98, 1296.21, 1339.89, 1344.32, 1348.37, 1348.51, 1349.74, 1350.34, 1351.72, 1352.56, 1354.41, 1366.6, 1370.08, 1370.86, 1371.63, 1372.54, 1373.88, 1374.16, 1375.24, 1377.66, 1378.84, 1379.29, 1380.8, 1382.89, 1389.78, 1403.24, 1406.37, 1440.22, 1456.29, 1456.96, 1470.03, 1477.16, 1477.82, 1480.26, 1480.42, 1484.57, 1484.93, 1487.09, 1488.76, 1488.87, 1489.31, 1491.25, 1508.31, 1511.47, 1528.39, 1533.72, 1536.39, 1538.64, 1540.04, 1542.22, 1544.51, 1556.77, 1563.24, 1570.03, 1570.52, 1573.01, 1575.19, 1580.12, 1582.17, 1584.04, 1585.72, 1588.34, 1589.66, 1591.1, 1622.82, 1623.39, 1624.67, 1625.51, 1626.44, 1627.54, 1678.53, 1682.83, 1688.43, 1692.95, 1693.78, 1716.05, 1717.55, 1724.04, 1724.33, 1735.28, 1735.96, 1738.04, 1739.44, 1740.86, 1741.17, 1752.6, 1752.91, 1754.27, 1755.31, 1755.51, 1758.73, 1793.75, 2242.63, 3111.9, 3114.5, 3119.64, 3122.22, 3169.78, 3178.69, 3193.83, 3196.35, 3196.69, 3197.0, 3200.22, 3201.96, 3210.93, 3220.65, 3235.59, 3247.87, 3269.71, 3270.72, 3275.07, 3278.68, 3279.5, 3283.54, 3284.7, 3291.94, 3293.67, 3296.01, 3299.87, 3303.57, 3304.38, 3309.75, 3312.91, 3317.76, 3322.72, 3638.22

X-TS

-78.77, 7.65, 16.43, 20.03, 24.46, 29.34, 37.73, 40.34, 42.87, 54.32, 57.54, 61.97, 72.56, 93.23, 101.25, 113.33, 132.27, 142.04, 160.33, 184.5, 191.22, 214.52, 240.19, 254.1, 263.07, 268.89, 283.08, 292.94, 309.51, 322.81, 329.97, 337.06, 348.92, 353.53, 357.64, 368.15, 380.93, 408.19, 417.64, 422.53, 426.7, 434.28, 445.63, 468.78, 474.28, 487.66, 501.26, 508.36, 513.28, 532.19, 548.81, 566.78, 575.98, 590.66, 611.67, 618.06, 625.14, 646.79, 649.8, 660.25, 660.88, 699.49, 702.34, 707.05, 739.56, 763.67, 771.36, 779.57, 807.44, 810.83, 840.54, 846.6, 860.82, 865.22, 871.9, 877.08, 883.98, 887.74, 888.7, 894.46, 938.3, 946.02, 971.95, 981.88, 996.75, 1007.83, 1008.48, 1013.03, 1022.46, 1023.79, 1033.6, 1036.75, 1042.99, 1047.01, 1054.04, 1061.02, 1064.86, 1072.23, 1077.92, 1079.34, 1084.77, 1097.27, 1101.22, 1146.09, 1146.95, 1149.94, 1170.7, 1177.08, 1185.69, 1193.29, 1213.21, 1217.47, 1226.02, 1227.76, 1245.47, 1254.91, 1267.69, 1277.22, 1280.0, 1282.46, 1303.59, 1321.81, 1346.75, 1368.88, 1375.41, 1376.09,

1381.11, 1381.34, 1384.58, 1389.84, 1412.6, 1419.41, 1457.9, 1458.41, 1462.81, 1470.76, 1487.33, 1488.94, 1492.22, 1525.88, 1534.15, 1536.08, 1538.86, 1538.91, 1542.25, 1544.08, 1557.65, 1564.28, 1565.41, 1572.45, 1585.87, 1586.98, 1588.22, 1672.69, 1674.63, 1689.23, 1698.23, 1707.23, 1712.76, 1718.17, 1720.37, 2052.67, 3113.77, 3116.74, 3120.35, 3123.77, 3196.49, 3200.15, 3200.46, 3200.81, 3204.12, 3205.43, 3206.5, 3223.46, 3267.01, 3269.45, 3271.06, 3271.38, 3274.87, 3276.11, 3283.62, 3290.4, 3290.54, 3291.64, 3291.68, 3298.32, 3298.86, 3301.84, 3302.69, 3305.17, 3311.46, 3610.9

1a

19.76, 24.83, 37.19, 42.71, 55.66, 61.9, 78.34, 92.79, 106.38, 134.26, 152.36, 179.38, 188.95, 237.88, 251.69, 297.27, 311.64, 323.79, 334.24, 354.56, 405.16, 421.34, 427.2, 428.41, 449.48, 487.7, 502.01, 522.67, 542.8, 555.3, 570.42, 586.75, 600.6, 613.97, 628.58, 652.8, 655.06, 662.92, 699.54, 728.22, 733.31, 742.63, 780.04, 804.97, 805.81, 847.35, 857.31, 871.38, 876.7, 886.42, 893.35, 922.35, 940.38, 974.08, 1008.99, 1012.02, 1027.15, 1029.48, 1042.15, 1044.76, 1045.3, 1051.64, 1065.16, 1085.09, 1099.34, 1099.94, 1138.54, 1149.21, 1153.01, 1182.1, 1214.85, 1215.5, 1219.79, 1223.35, 1238.7, 1251.94, 1281.29, 1288.12, 1350.49, 1360.98, 1365.55, 1373.77, 1389.79, 1395.58, 1400.82, 1424.43, 1467.66, 1484.67, 1490.29, 1527.93, 1538.12, 1543.3, 1547.32, 1574.36, 1590.6, 1597.21, 1685.67, 1690.58, 1693.67, 1721.26, 1726.5, 1729.59, 2400.38, 3116.97, 3189.78, 3213.21, 3263.26, 3265.71, 3267.33, 3273.76, 3273.95, 3282.67, 3285.22, 3288.48, 3292.73, 3294.19, 3294.73, 3295.27, 3309.59, 3608.89

2a

20.88, 32.47, 35.31, 55.59, 63.01, 86.04, 107.55, 159.09, 201.31, 236.46, 247.97, 262.71, 287.6, 308.16, 334.97, 354.14, 354.95, 364.3, 391.7, 433.91, 446.65, 482.83, 485.46, 544.4, 588.7, 591.83, 596.89, 612.66, 633.55, 664.1, 684.13, 687.26, 780.79, 787.71, 867.88, 886.04, 886.49, 889.41, 972.23, 981.23, 996.0, 999.81, 1020.31, 1031.5, 1059.17, 1060.46, 1063.99, 1064.89, 1080.23, 1086.94, 1157.47, 1176.73, 1181.05, 1220.16, 1258.27, 1265.96, 1278.69, 1278.82, 1296.63, 1332.17, 1348.26, 1371.5, 1375.61, 1376.16, 1391.35, 1455.1, 1456.31, 1485.72, 1486.27, 1499.6, 1526.05, 1534.15, 1536.74, 1540.49, 1556.39, 1563.17, 1573.26, 1588.67, 1675.57, 1719.11, 1920.21, 1974.59, 3111.45, 3112.63, 3117.41, 3151.29, 3158.25, 3192.8, 3193.22, 3196.22, 3197.51, 3198.89, 3203.28, 3205.12, 3211.45, 3263.34, 3282.37, 3289.92, 3296.8

3aa

14.42, 18.27, 23.58, 29.86, 36.01, 43.73, 49.42, 59.37, 61.7, 72.33, 80.73, 90.51, 118.29, 131.89, 145.09, 158.85, 170.44, 192.22, 193.95, 217.23, 218.97, 253.2, 258.94, 266.0, 279.58, 283.11, 289.15, 299.92, 315.31, 324.11, 335.8, 351.58, 358.31, 365.02, 368.1, 393.52, 420.19, 421.84, 425.47, 430.93, 435.04, 456.3, 468.94, 477.15, 487.83, 498.55, 512.64, 521.05, 555.47, 577.24, 590.42, 605.65, 612.27, 618.98, 643.17, 655.44, 660.57, 665.84, 693.18, 717.38, 729.78, 736.57, 738.49, 766.14, 769.06, 777.26, 797.45, 809.61, 820.94, 844.89, 850.41, 872.84, 876.1, 880.65, 883.53, 886.61, 896.43, 914.89, 969.09, 979.13, 981.03, 985.88, 994.74, 998.6, 1001.97, 1003.79, 1017.16, 1024.89, 1029.22, 1038.93, 1044.62, 1044.9, 1049.21, 1057.97, 1063.35, 1078.68, 1079.52, 1084.11, 1086.24, 1097.47, 1104.37, 1144.59, 1151.72, 1154.01, 1177.99, 1181.79,

1183.91, 1188.48, 1203.81, 1215.42, 1217.93, 1230.23, 1246.69, 1253.21, 1263.65, 1277.69, 1279.53, 1281.62, 1294.61, 1337.85, 1349.34, 1368.24, 1372.45, 1376.71, 1378.21, 1384.06, 1393.56, 1405.57, 1415.68, 1432.39, 1442.8, 1452.44, 1455.59, 1468.44, 1484.11, 1488.39, 1489.33, 1528.66, 1532.34, 1535.37, 1536.72, 1539.15, 1541.31, 1544.79, 1555.89, 1562.52, 1564.83, 1572.57, 1580.6, 1591.29, 1591.64, 1655.52, 1676.62, 1694.28, 1695.72, 1700.2, 1723.97, 1725.27, 1727.68, 1731.65, 3107.71, 3111.15, 3115.87, 3119.41, 3188.91, 3192.03, 3192.65, 3193.64, 3195.6, 3197.54, 3204.78, 3216.66, 3252.06, 3261.83, 3264.31, 3265.02, 3268.99, 3271.03, 3276.75, 3277.38, 3278.05, 3278.86, 3285.56, 3288.43, 3289.9, 3296.01, 3301.28, 3303.84, 3337.06

5aa

19.71, 30.16, 38.98, 57.05, 59.02, 83.11, 86.39, 95.34, 107.82, 142.6, 160.74, 178.7, 214.76, 223.14, 260.26, 263.7, 279.73, 284.52, 292.58, 313.1, 336.48, 347.34, 353.86, 367.06, 369.18, 396.76, 424.9, 427.27, 436.28, 459.57, 475.17, 482.07, 503.11, 551.17, 558.3, 564.08, 582.22, 616.75, 624.99, 640.99, 655.62, 664.77, 691.86, 732.95, 737.86, 771.75, 781.95, 791.58, 800.26, 811.68, 845.58, 873.24, 877.87, 886.36, 892.74, 894.84, 917.16, 970.45, 971.88, 980.71, 983.81, 994.29, 998.0, 1007.3, 1017.73, 1024.56, 1029.07, 1043.05, 1045.74, 1058.36, 1070.57, 1079.52, 1086.16, 1089.83, 1093.21, 1115.56, 1137.52, 1150.28, 1158.96, 1178.85, 1181.3, 1192.61, 1213.11, 1214.98, 1238.32, 1251.87, 1257.35, 1278.56, 1278.82, 1308.53, 1334.38, 1348.82, 1353.71, 1367.15, 1371.18, 1377.33, 1392.33, 1398.36, 1400.87, 1453.83, 1455.33, 1461.93, 1484.7, 1487.47, 1527.56, 1533.43, 1536.37, 1540.13, 1540.52, 1550.2, 1556.19, 1562.18, 1572.81, 1583.51, 1589.93, 1590.78, 1680.43, 1690.11, 1698.55, 1723.2, 1724.94, 1727.66, 1752.76, 3028.65, 3110.06, 3111.4, 3116.32, 3160.09, 3190.46, 3191.36, 3195.09, 3197.03, 3201.43, 3204.15, 3256.64, 3260.27, 3267.48, 3268.89, 3275.39, 3276.36, 3279.03, 3280.18, 3281.96, 3286.51, 3288.75, 3294.12, 3295.53

4a

3.82, 27.93, 35.61, 71.38, 125.96, 134.15, 179.95, 242.86, 296.71, 333.51, 406.39, 421.78, 434.06, 473.88, 506.04, 540.85, 564.79, 588.71, 639.44, 643.04, 650.39, 726.25, 728.56, 767.9, 798.4, 808.38, 864.72, 880.97, 893.88, 938.31, 979.98, 1012.21, 1030.85, 1033.1, 1037.11, 1040.7, 1045.35, 1053.24, 1074.09, 1085.77, 1091.5, 1125.16, 1138.51, 1151.49, 1213.29, 1215.83, 1238.39, 1242.48, 1306.1, 1336.11, 1351.04, 1366.21, 1386.2, 1400.63, 1412.77, 1470.26, 1528.69, 1544.32, 1556.14, 1587.48, 1597.04, 1693.37, 1706.98, 1726.56, 1732.24, 2436.66, 3055.08, 3094.74, 3265.73, 3267.58, 3272.24, 3275.68, 3283.68, 3284.27, 3287.97, 3290.75, 3294.56, 3295.38

3ka

7.06, 16.78, 35.05, 46.07, 54.41, 64.55, 71.77, 100.49, 136.03, 172.61, 185.03, 197.83, 217.74, 256.17, 261.42, 284.78, 296.64, 303.71, 318.44, 343.64, 351.1, 353.96, 366.44, 411.28, 423.56, 425.58, 429.96, 454.81, 472.95, 481.09, 501.06, 537.2, 570.24, 592.47, 611.91, 616.17, 618.73, 643.88, 665.88, 686.75, 716.06, 727.07, 742.09, 763.96, 775.93, 793.58, 805.44, 820.5, 866.77, 872.9, 876.81, 886.43, 888.89, 907.71, 930.92, 969.94, 976.73, 980.6, 993.63, 994.28, 1001.34, 1016.62, 1027.7, 1032.19, 1042.52, 1052.27, 1057.02, 1067.58, 1070.24, 1079.62, 1087.45,

1094.42, 1146.93, 1152.39, 1170.61, 1181.0, 1188.5, 1194.5, 1209.2, 1217.81, 1251.48, 1263.91, 1278.61, 1279.02, 1284.03, 1346.78, 1350.01, 1364.07, 1368.68, 1374.61, 1378.03, 1409.12, 1415.41, 1437.65, 1454.84, 1455.59, 1485.1, 1489.1, 1528.96, 1534.14, 1536.82, 1540.66, 1546.97, 1556.81, 1562.84, 1566.7, 1573.08, 1582.02, 1591.13, 1665.29, 1677.93, 1702.27, 1710.63, 1725.12, 1730.55, 1735.46, 3110.01, 3111.07, 3116.1, 3190.86, 3191.52, 3194.33, 3197.18, 3202.29, 3203.94, 3256.45, 3258.41, 3267.96, 3271.23, 3277.7, 3281.27, 3281.84, 3286.87, 3288.46, 3290.72, 3294.18, 3298.08, 3302.4

1k

17.67, 40.14, 51.83, 133.77, 142.21, 229.61, 251.27, 317.18, 374.25, 402.41, 422.35, 429.73, 504.84, 512.3, 548.01, 570.62, 596.49, 615.79, 623.1, 653.14, 722.03, 728.45, 780.27, 792.1, 804.79, 849.4, 891.71, 892.22, 901.2, 974.11, 988.73, 1023.17, 1028.21, 1042.24, 1049.29, 1085.38, 1102.76, 1137.67, 1148.86, 1213.85, 1214.57, 1218.33, 1236.34, 1239.16, 1344.24, 1364.51, 1385.35, 1399.16, 1400.71, 1408.87, 1528.04, 1543.24, 1582.77, 1609.59, 1690.2, 1704.59, 1721.55, 1731.87, 2404.08, 3242.45, 3265.98, 3273.95, 3275.45, 3283.49, 3284.53, 3289.97, 3294.6, 3297.48, 3927.53

5. DFT calculations for PAHs

All ground-state optimisations have been carried out at the Density Functional Theory (DFT) level with Gaussian16[39] using the PBE0 functional[40] and the 6-31G(d,p) basis set.[41] Excited-state calculations have been performed at Time-Dependent DFT (TD-DFT) within the Tamm-Dancoff approximation (TDA)[42,43] using the same functional and basis set as for ground state geometry optimisation. Spin-orbit coupling matrix elements (ξ) were calculated based on the optimised singlet excited state geometry. Molecular orbitals were visualised using GaussView 6.0.[44] Calculations were submitted and processed using the Digichem (version 6) software package,[45]which incorporates a number of publicly available software libraries, including: cclib[46] for parsing of result files, VMD[47] and Tachyon[48] for 3D rendering, Matplotlib[49] for the plotting of graphs, Open Babel[50] and Pybel[51] for file interconversion, and PySOC[52] for the calculation of spin-orbit coupling.

6. Electrochemistry

Cyclic Voltammetry (CV) and Differential pulse voltammetry (DPV) analysis were performed on an Electrochemical Analyser potentiostat model 620E from CH Instruments. Samples were prepared in dichloromethane solution, which were degassed by sparging with CH_2Cl_2-saturated nitrogen gas for 5 minutes prior to measurements. All measurements were performed using 0.1M tetra-*n*-butylammonium hexafluorophosphate, $[^nBu_4N]PF_6$, as an electrolyte, in CH_2Cl_2 at scan rate of 100 mV s^{-1}. A Ag/Ag^+ electrode was used as the reference electrode, a platinum disk electrode was used as the working electrode, and a platinum wire was used as the counter electrode. The redox potentials are reported relative to a saturated calomel electrode (SCE) with a ferrocene/ferrocenium/ (F_c/F_c^+) redox couple as the internal standard (0.46 V vs SCE).[53]The HOMO and LUMO energies were determined using $E_{HOMO/LUMO} = -(E^{ox}/E^{red} + 4.8)eV$,[54] where E^{ox} is anodic peak potential and E^{red} is cathodic peak potential determined from the DPV relative to F_c/F_c^+.

7. Photophysical measurements

Absorption spectra were recorded at room temperature using a Shimadzu UV-2600 double beam spectrophotometer. The steady-state emission, and time-resolved emission spectra or PL decays were recorded at room temperature using an Edinburgh Instruments FS5 spectrofluorometer. An

Edinburgh Instruments FS5 spectrofluorometer equipped with a 150 W ozone free Xenon lamp was used to collect steady-state emission and an EPL UV picosecond pulsed diode laser (EPL-375, λ_{exc} = 375 nm) was used to collect time-resolved decays.

7.1 Photophysical measurements in solution

Optically diluted solutions of concentrations on the order of 10^{-5} or 10^{-6} M were prepared in spectroscopic or HPLC grade solvents for absorption and emission analysis. Molar absorptivity values were determined in toluene from at least four solutions, followed by linear regression analysis, having concentration 1.45 × 10^{-4} M to 2.07 × 10^{-5} M, with corresponding absorbances of 2.31 to 0.35, respectively, at an absorption wavelength of 330 nm for **7**, at 1.09 × 10^{-4} M to 1.56 × 10^{-5} M, with corresponding absorbances of 1.5 to 0.25 respectively, at an absorption wavelength of 304 nm for **8**.

The steady-state PL spectra and time-resolved PL decays of emitters under an air atmosphere were measured in toluene solutions. PL decays were measured using time-correlated single photon counting (TCSPC) mode, and data were fitted with respect to the instrument response function (IRF).

7.2 Photoluminescence quantum yields (PLQY) in solution

Photoluminescence quantum yields for degassed and aerated solutions were determined using the optically dilute method[55] in which four sample solutions with absorbance of 0.093, 0.062, 0.034 and 0.013 at 330 nm were used for **7**, 0.090, 0.060, 0.032 and 0.013 at 305 nm were used for, their emission intensities were compared with those of a reference, quinine sulphate whose quantum yield (Φ_r) in 0.1 M H_2SO_4 was determined to be 54.0%[56,57] using the absolute method. The quantum yield of the sample, Φ_{PL}, can be determined by the equation $\Phi_{PL} = \Phi_r(A_r/A_s)(I_s/I_r)(n_s/n_r)^2$, where A stands for the absorbance at the excitation wavelength, I is the integrated area under the corrected emission curve and n is the refractive index of the solvent with the subscripts “s” and “r” representing sample and reference respectively. The experimental uncertainty in the Φ_{PL} is conservatively estimated to be 10%, though we have found that statistically we can reproduce Φ_{PL} values to within 3% relative error.

Figure S1. The HOMO and LUMO distributions (isovalue = 0.02) of **7** and **8**, together with the transition energies for the relevant lowest singlet and triplet states calculated at the PBE0/6-31G(d,p) level in the gas phase. Blue lobes indicate electron density of HOMO, and red lobes indicate electron density of LUMO. For singlet state, blue colour represents hole, and red represents particle. *f* denotes the oscillator strength for the transition to the excited singlet state.

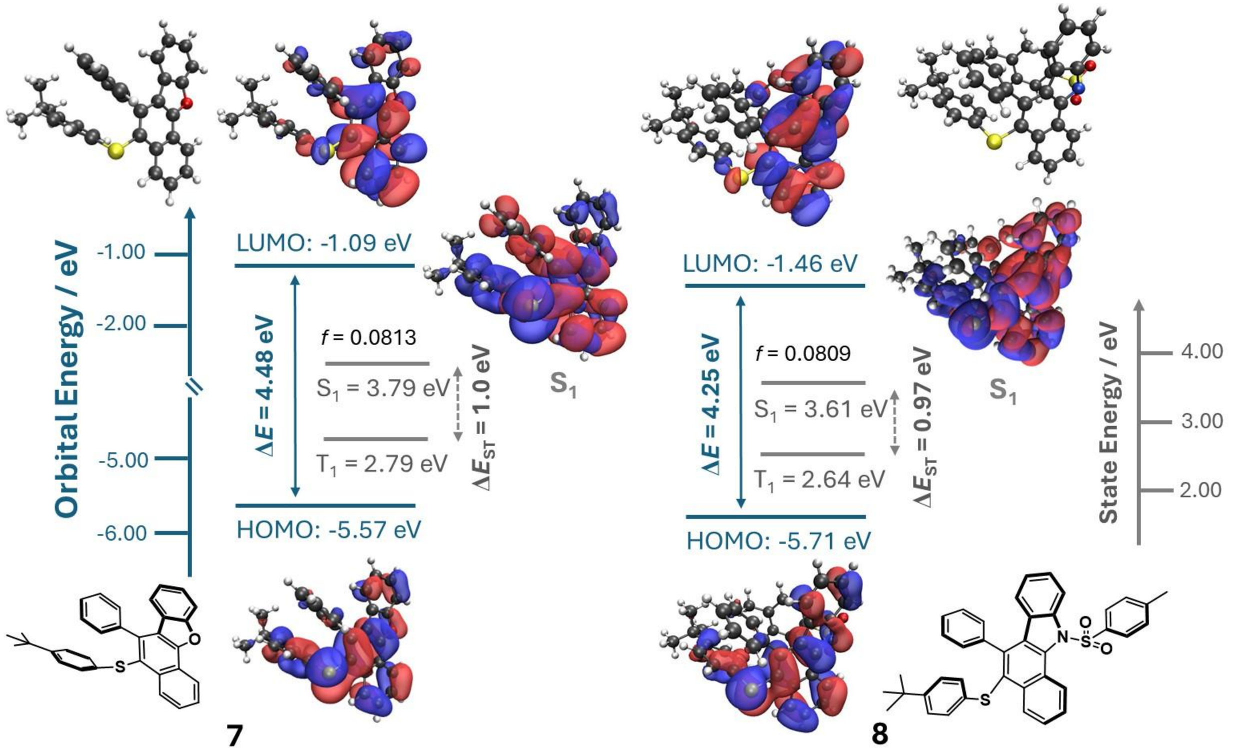

Figure S2. TDA-DFT simulation and experimental UV-Vis. absorption spectrum in the gas phase of (a) **7** and (b) **8**.

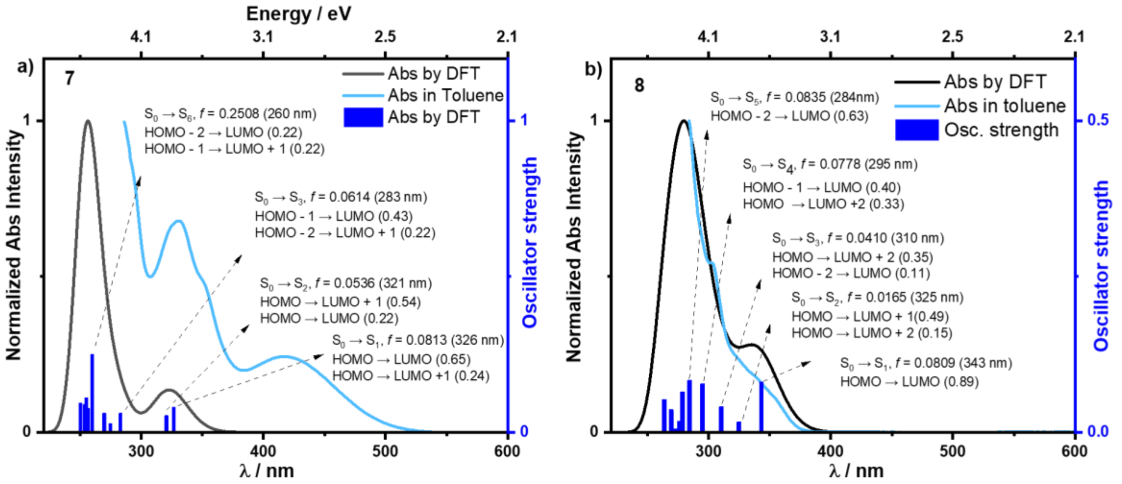

Figure S3. Cyclic and differential pulse voltammograms of a) **7** and b) **8** in degassed CH_2Cl_2 (scan rate = 100 mV s^{-1}).

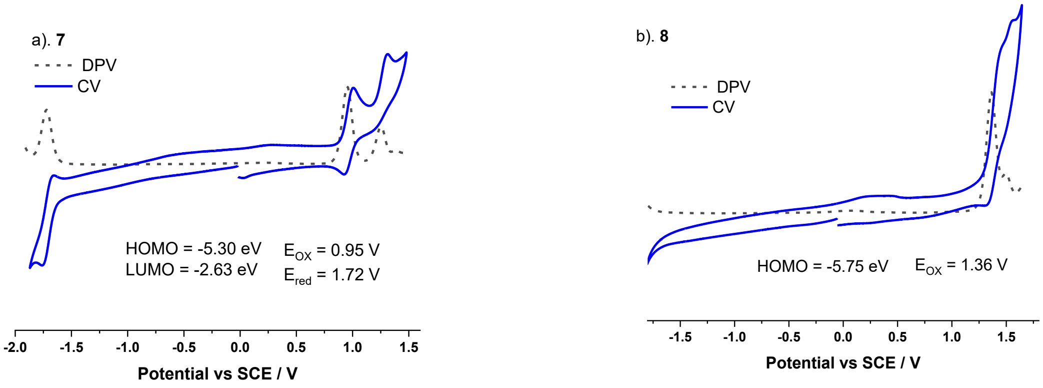

Table S2. Electrochemical data and HOMO-LUMO levels for **7** and **8**.

Emitters	***E_{ox}^{a} / V***	***E_{red}^{a}/ V***	***HOMO^b /eV***	***LUMO^b / eV***	***E_{opt}/ eV***
**7**	0.95	−1.72	−5.30	−2.63/ 2.03^c	3.27
**8**	1.36	-	−5.75	−2.36^c	3.39

[a] E_{ox} and E_{red} are anodic and cathodic peak potentials, respectively, obtained from DPV using F_c/F_c^+ as the internal reference and referenced versus SCE (0.46 V vs. SCE) in CH_2Cl_2 with 0.1 M [^{n}Bu$_4$N]PF$_6$ as the supporting electrolyte. [b] $E_{HOMO/LUMO} = -(E^{ox}/E^{red} + 4.8)eV$, where E^{ox} is anodic peak potential and $E^{red}$is cathodic peak potential calculated from DPV relative to F_c/F_c^+. [c]$E_{HOMO} = |E_{LUMO} - E_{opt}|$ *where* E_{opt} is the optical band gap obtained from the intersection of the normalised absorption and emission spectra in toluene (Figure S4).

Figure S4. Normalised absorbance and PL spectra of a) **7** (λ_{exc} = 330 nm) and b) **8** (λ_{exc} = 305 nm) in toluene.

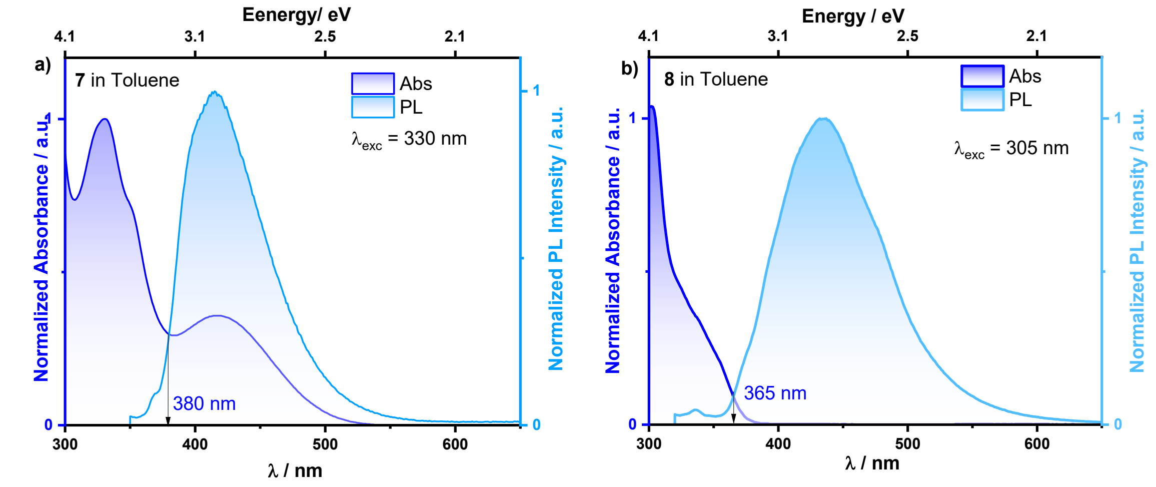

Table S3. Photophysical measurements of **7** and **8** in toluene.

Emitters	λ_{abs} / nm (ε/ ×10³ M⁻¹cm⁻¹)	λ_{PL}[a] / nm	Φ_{PL}[a] / %	$\tau_{avg, PL}$[b] / ns
**7**	330 (16.7), 415 (6.0)	415	0.5	3.84
**8**	305 (15.2), 345 (4.2)	437	0.4	4.47

[a] At 298 K, values quoted under air atmosphere and photoluminescence quantum yields (Φ_{PL}) of emitters in toluene were measure under air (λ_{exc} = 330 nm for **7** and λ_{exc} = 305 nm for **8**). [b] Lifetime was measured by TCSPC (λ_{exc} = 375 nm), reported lifetime values are average lifetime ($\tau_{avg} = \Sigma A_i \tau_i^2 / \Sigma A_i \tau_i$, where A_i is the pre-exponential for lifetime τ_i.

Figure S5. Time-resolved PL decay of (a) **7** and (b) **8** in toluene (λ_{exc}= 375 nm).

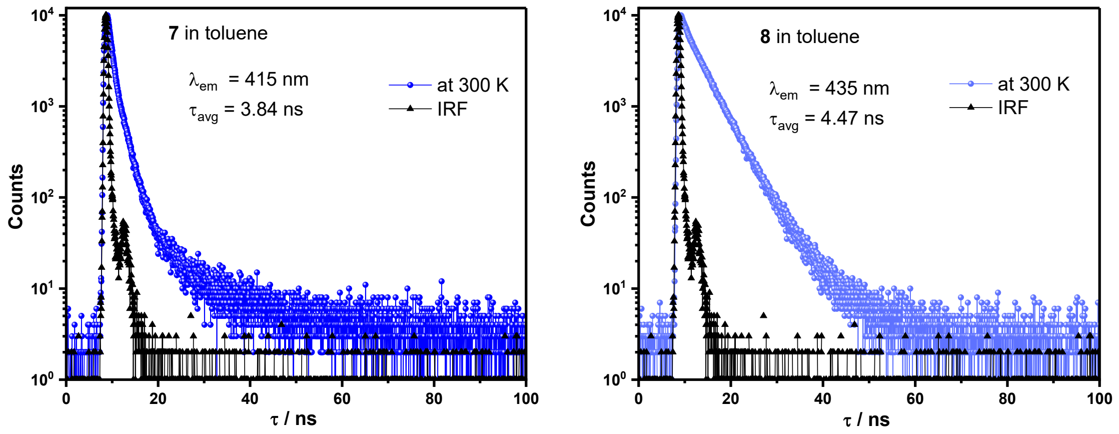

Figure S6. Excitation spectra of 7at λem= 415 nm and at λem= 650 nm in toluene.

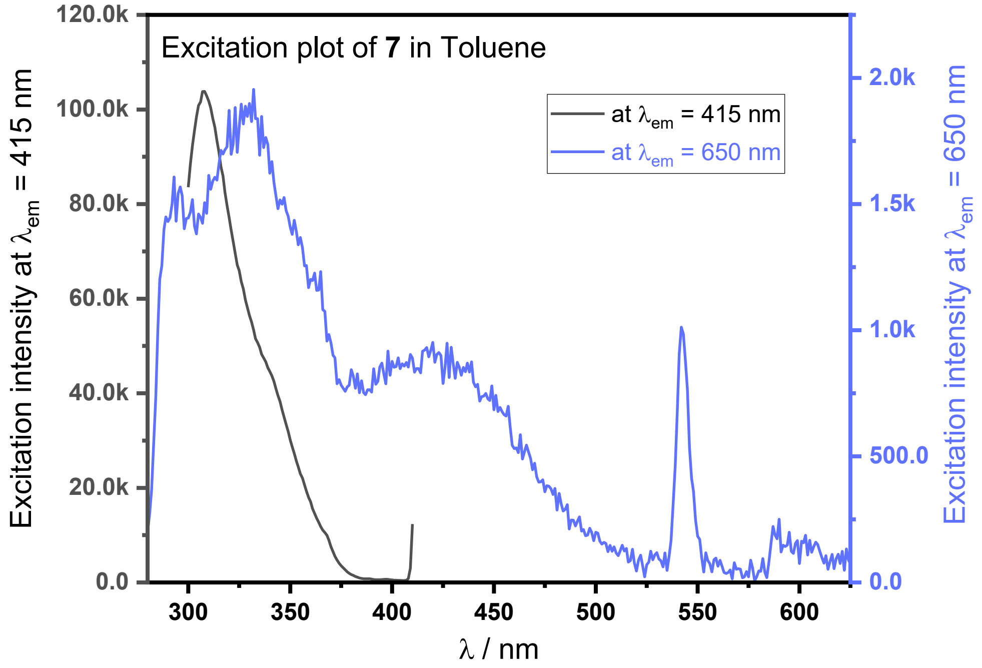

Figure S7. Normalised PL spectra of **7** λ_{exc} = 415 nm in toluene. Sharp band at 475 nm related to scattered peak.

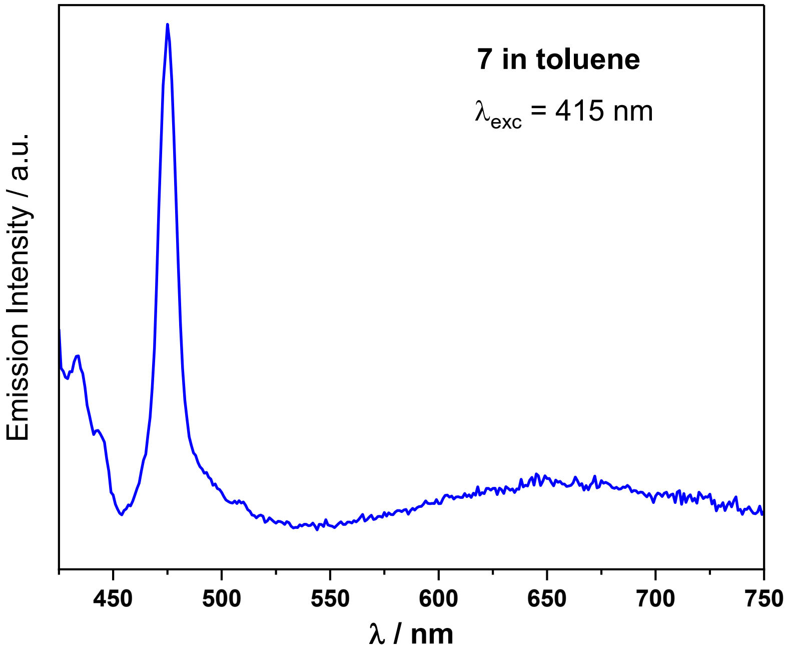

Figure S8. Excitation spectra of **8** at λ_{em} = 445 nm in toluene.

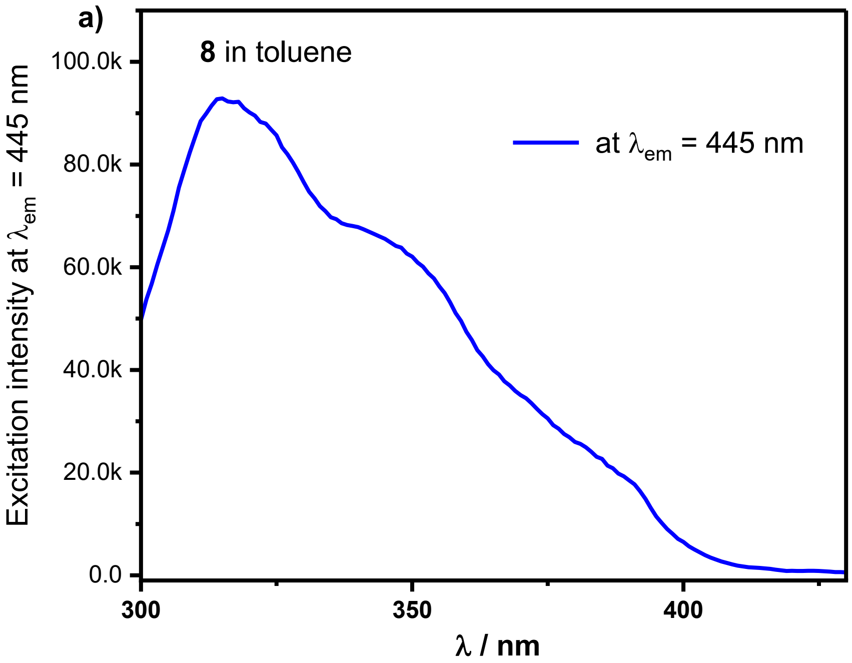

Figure S9. Normalised PL spectra of **8** with different excitation.

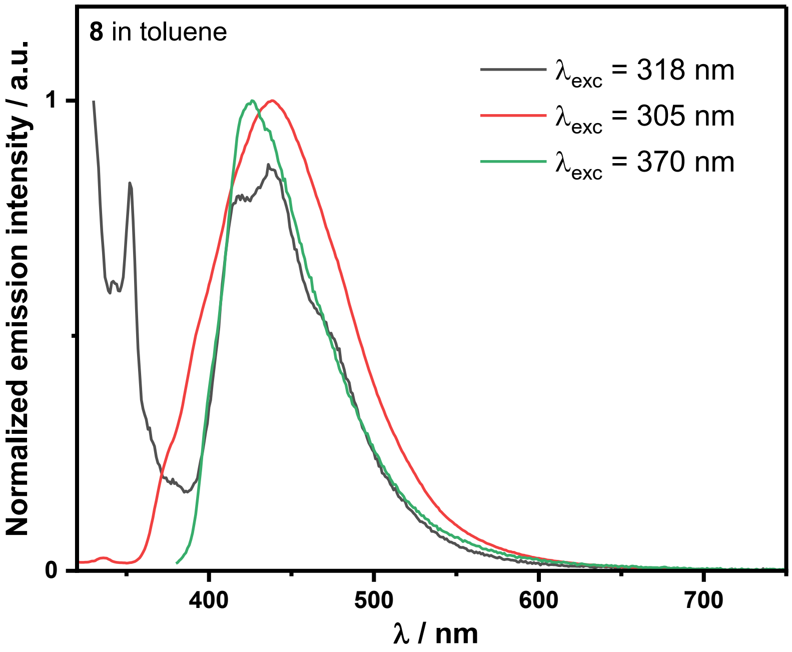

8. References

1 M. Santi, D. M. C. Ould, J. Wenz, Y. Soltani, R. L. Melen and T. Wirth, *Angew. Chem. Int. Ed.*, 2019, **58**, 7861–7865.
2 J. Liu, X. Xie and Y. Liu, *Chem. Commun.*, 2013, **49**, 11794–11796.
3 J. Lv, B. Zhao, L. Liu, Y. Han, Y. Yuan and Z. Shi, *Adv. Synth. Catal.*, 2018, **360**, 4054–4059.
4 Y. Ye, K. P. S. Cheung, L. He and G. C. Tsui, *Org. Chem. Front.*, 2018, **5**, 1511–1515.
5 J. McNulty and K. Keskar, *Eur. J. Org. Chem.*, 2014, **2014**, 1622–1629.
6 F. Zhao, W. Hagui, M. Ballarín-Marión, C. Ollivier, V. Mouriès-Mansuy and L. Fensterbank, *ACS Org. Inorg. Au*, 2023, **3**, 19–26.
7 Y.-P. He, J. Cao, H. Wu, Q. Wang and J. Zhu, *Angew. Chem. Int. Ed.*, 2021, **60**, 7093–7097.
8 G. da Silva, A. F. S. Luz, D. Duarte, D. Fontinha, V. L. M. Silva, F. A. Almeida Paz, A. M. Madureira, S. Simões, M. Prudêncio, F. Nogueira, A. M. S. Silva and R. Moreira, *ChemMedChem*, 2023, **18**, e202300264.
9 S. Taschinski, R. Döpp, M. Ackermann, F. Rominger, F. de Vries, M. F. S. J. Menger, M. Rudolph, A. S. K. Hashmi and J. E. M. N. Klein, *Angew. Chem. Int. Ed.*, 2019, **58**, 16988–16993.
10 Z. Wang, T. Cao and S. Zhu, *Org. Lett.*, 2022, **24**, 9296–9300.
11 M. Pramanik, S. Das, R. Babaahmadi, S. Pahar, T. Wirth, E. Richards and R. L. Melen, *Chem.*, 2024, **10**, 2901–2915.
12 C.-V. T. Vo, T. A. Mitchell and J. W. Bode, *J. Am. Chem. Soc.*, 2011, **133**, 14082–14089.
13 P. S. Hellwig, A. M. Barcellos, R. Cargnelutti, T. Barcellos and G. Perin, *J. Org. Chem.*, 2022, **87**, 15050–15060.
14 J.-H. Liang, L. Yang, S. Wu, S.-S. Liu, M. Cushman, J. Tian, N.-M. Li, Q.-H. Yang, H.-A. Zhang, Y.-J. Qiu, L. Xiang, C.-X. Ma, X.-M. Li and H. Qing, *Eur. J. Med. Chem.*, 2017, **136**, 382–392.
15 Y.-L. Wang, W.-M. Zhang, J.-J. Dai, Y.-S. Feng and H.-J. Xu, *RSC Adv.*, 2014, **4**, 61706–61710.
16 A. Arcadi, F. Blesi, S. Cacchi, G. Fabrizi, A. Goggiamani and F. Marinelli, *Org. Biomol. Chem.*, 2012, **10**, 9700–9708.
17 I. Medina-Mercado, A. Colin-Molina, J. E. Barquera-Lozada, B. Rodríguez-Molina and S. Porcel, *ACS Catal.*, 2021, **11**, 8968–8977.
18 H. Huang, G. Zhang, L. Gong, S. Zhang and Y. Chen, *J. Am. Chem. Soc.*, 2014, **136**, 2280–2283.
19 A. J. Warner, J. R. Lawson, V. Fasano and M. J. Ingleson, *Angew. Chem. Int. Ed.*, 2015, **54**, 11245–11249.
20 B. Zhang, X. Li, X. Li, Z. Yu, B. Zhao, X. Wang, Y. Du and K. Zhao, *J. Org. Chem.*, 2021, **86**, 17274–17281.
21 Q. Shi, P. Li, Y. Zhang and L. Wang, *Org. Chem. Front.*, 2017, **4**, 1322–1330.
22 M. Xu, X.-H. Zhang and P. Zhong, *Tetrahedron Lett.,*2011, **52**, 6800–6804.
23 J. Sheng, C. Fan and J. Wu, *Chem. Commun.*, 2014, **50**, 5494–5496.
24 F. Neese, *WIREs Comput.Mol. Sci.* 2022, **12**, e1606.
25 F. Neese, F. Wennmohs, U. Becker and C. Riplinger, *J. Chem. Phys.*, 2020, **152**, 224108.
26 S. Grimme, J. Antony, S. Ehrlich and H. Krieg, *J. Chem. Phys.*, 2010, **132**, 154104.
27 S. Grimme, J. G. Brandenburg, C. Bannwarth and A. Hansen, *J. Chem. Phys.*, 2015, **143**, 054107.
28 N. Mardirossian and M. Head-Gordon, *J. Chem. Phys.*, 2016, **144**, 214110.
29 F. Weigend and R. Ahlrichs, *Phys. Chem. Chem. Phys.*, 2005, **7**, 3297–3305.
30 D. Rappoport and F. Furche, *J. Chem. Phys.*, 2010, **133**, 134105.
31 M. Garcia-Ratés and F. Neese, *J. Comput. Chem.*, 2020, **41**, 922–939.
32 M. Cossi, N. Rega, G. Scalmani and V. Barone, *J. Comput. Chem.*, 2003, **24**, 669–681.
33 H. Ryu, J. Park, H. K. Kim, J. Y. Park, S.-T. Kim and M.-H. Baik, *Organometallics*, 2018, **37**, 3228–3239.
34 F. Neese, *Chem. Phys. Lett.*, 2000, **325**, 93–98.
35 F. Neese, *J. Comput. Chem.*, 2023, **44**, 381–396.
36 F. Neese, *J. Comput. Chem.*, 2003, **24**, 1740–1747.
37 D. Bykov, T. Petrenko, R. Izsák, S. Kossmann, U. Becker, E. Valeev and F. Neese, *Mol. Phys.*,2015, **113**, 1961–1977.
38 F. Weigend, *Phys. Chem. Chem. Phys.*, 2006, **8**, 1057–1065.
39 M. J. Frisch, G. W. Trucks, H. B. Schlegel, G. E. Scuseria, M. A. Robb, J. R. Cheeseman, G. Scalmani, V. Barone, G. A. Petersson, H. Nakatsuji, X. Li, M. Caricato, A. V. Marenich, J. Bloino, B. G. Janesko, R. Gomperts, B. Mennucci, H. P. Hratchian, J. V. Ortiz, A. F. Izmaylov, J. L. Sonnenberg, Williams, F. Ding, F. Lipparini, F. Egidi, J. Goings, B. Peng, A. Petrone, T. Henderson, D. Ranasinghe, V. G. Zakrzewski, J. Gao, N. Rega, G. Zheng, W. Liang, M. Hada, M. Ehara, K. Toyota, R. Fukuda, J. Hasegawa, M. Ishida, T. Nakajima, Y. Honda, O. Kitao, H. Nakai, T. Vreven, K. Throssell, J. A. Montgomery Jr., J. E. Peralta, F. Ogliaro, M. J.

Bearpark, J. J. Heyd, E. N. Brothers, K. N. Kudin, V. N. Staroverov, T. A. Keith, R. Kobayashi, J. Normand, K. Raghavachari, A. P. Rendell, J. C. Burant, S. S. Iyengar, J. Tomasi, M. Cossi, J. M. Millam, M. Klene, C. Adamo, R. Cammi, J. W. Ochterski, R. L. Martin, K. Morokuma, O. Farkas, J. B. Foresman and D. J. Fox, Gaussian 16 Rev. C.01, Wallingford, CT , 2016..
40 C. Adamo and V. Barone, *J. Chem. Phys.*, 1999, **110**, 6158–6170.
41 T. H. Dunning Jr., *J. Chem. Phys.*, 1989, **90**, 1007–1023.
42 S. Grimme, *Chem. Phys. Lett.*, 1996, **259**, 128–137.
43 S. Hirata and M. Head-Gordon, *Chem. Phys. Lett.*, 1999, **314**, 291–299.
44 K. Abdoulaye, B. A. Lucie, O. Lamoussa, K. Soleymane and B. Kafoumba, *J. Mater. Sci. Chem. Eng.*, 2024, **12**, 31–50.
45 O. S. Lee, M. C. Gather and E. Zysman-Colman, *Digital Discovery*, 2024, **3**, 1695–1713.
46 N. M. O'boyle, A. L. Tenderholt and K. M. Langner, *J. Comput. Chem.*,2008, **29**, 839–845.
47 W. Humphrey, A. Dalke and K. Schulten, *J. Mol. Graphics*, 1996, **14**, 33–38.
48 J. Stone, *An efficient library for parallel ray tracing and animation.,* 1998.
49 J. D. Hunter, *Comput. Sci. Eng.*, 2007, **9**, 90–95.
50 A. Drefahl, *J. Cheminform*, 2011, **3**, 1.
51 N. M. O'Boyle, C. Morley and G. R. Hutchison, *Chem. Cent. J.*, 2008, **2**, 5.
52 X. Gao, S. Bai, D. Fazzi, T. Niehaus, M. Barbatti and W. Thiel, *J. Chem. Theory Comput.*, 2017, **13**, 515–524.
53 N. G. Connelly and W. E. Geiger, *Chem. Rev.*, 1996, **96**, 877–910.
54 C. M. Cardona, W. Li, A. E. Kaifer, D. Stockdale and G. C. Bazan, *Adv. Mater.*, 2011, **23**, 2367–2371.
55 G. A. Crosby and J. N. Demas, *J. Phys. Chem.*, 1971, **75**, 991–1024.
56 A. M. Brouwer, *Pure Appl. Chem.*, 2011, **83**, 2213–2228.
57 W. H. Melhuish, *J. Phys. Chem.*, 1961, **65**, 229–235.

9. NMR spectra

Figure S10. ^{1}H NMR (400 MHz, $CDCl_3$, 298 K) spectrum of *4-methyl-N-(2-(phenylethynyl)phenyl)benzenesulfonamide*, **1a**.

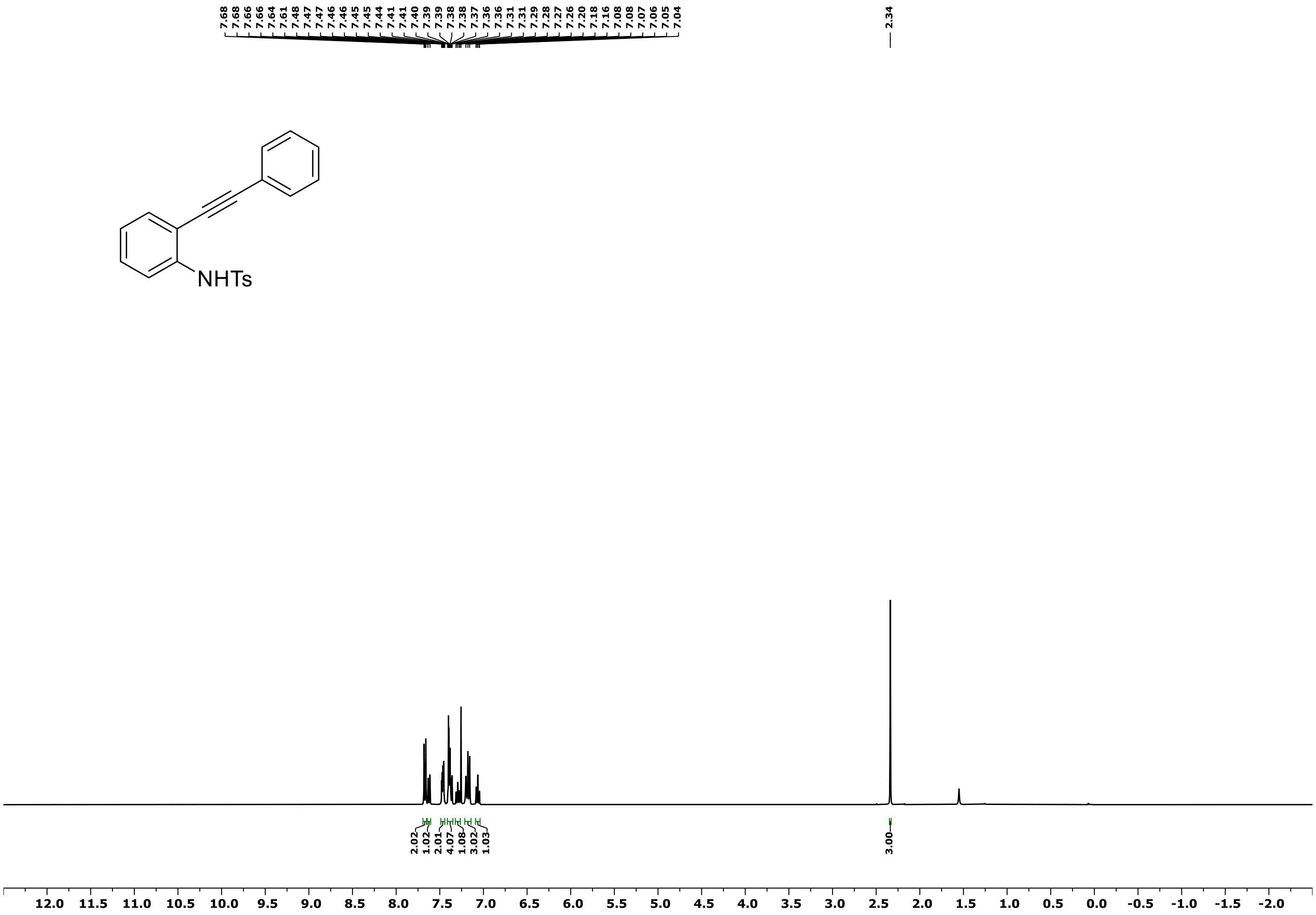

Figure S11. ^{13}C NMR (101 MHz, $CDCl_3$, 298 K) spectrum of *4-methyl-N-(2-(phenylethynyl)phenyl)benzenesulfonamide*, **1a**.

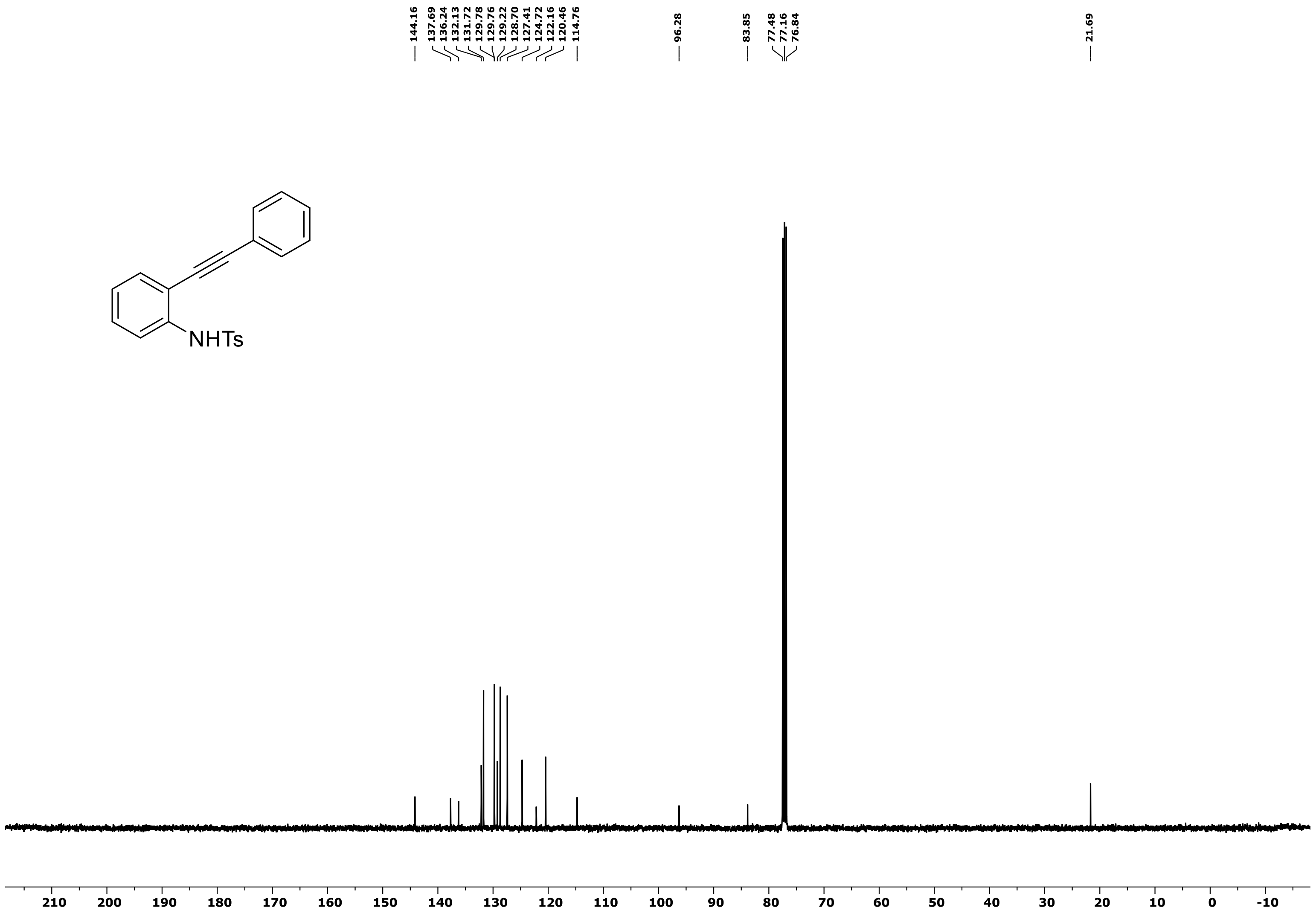

Figure S12. ^{1}H NMR (400 MHz, $CDCl_3$, 298 K) spectrum of *4-methyl-N-(2-(p-tolylethynyl) phenyl)benzenesulfonamide*, **1b**.

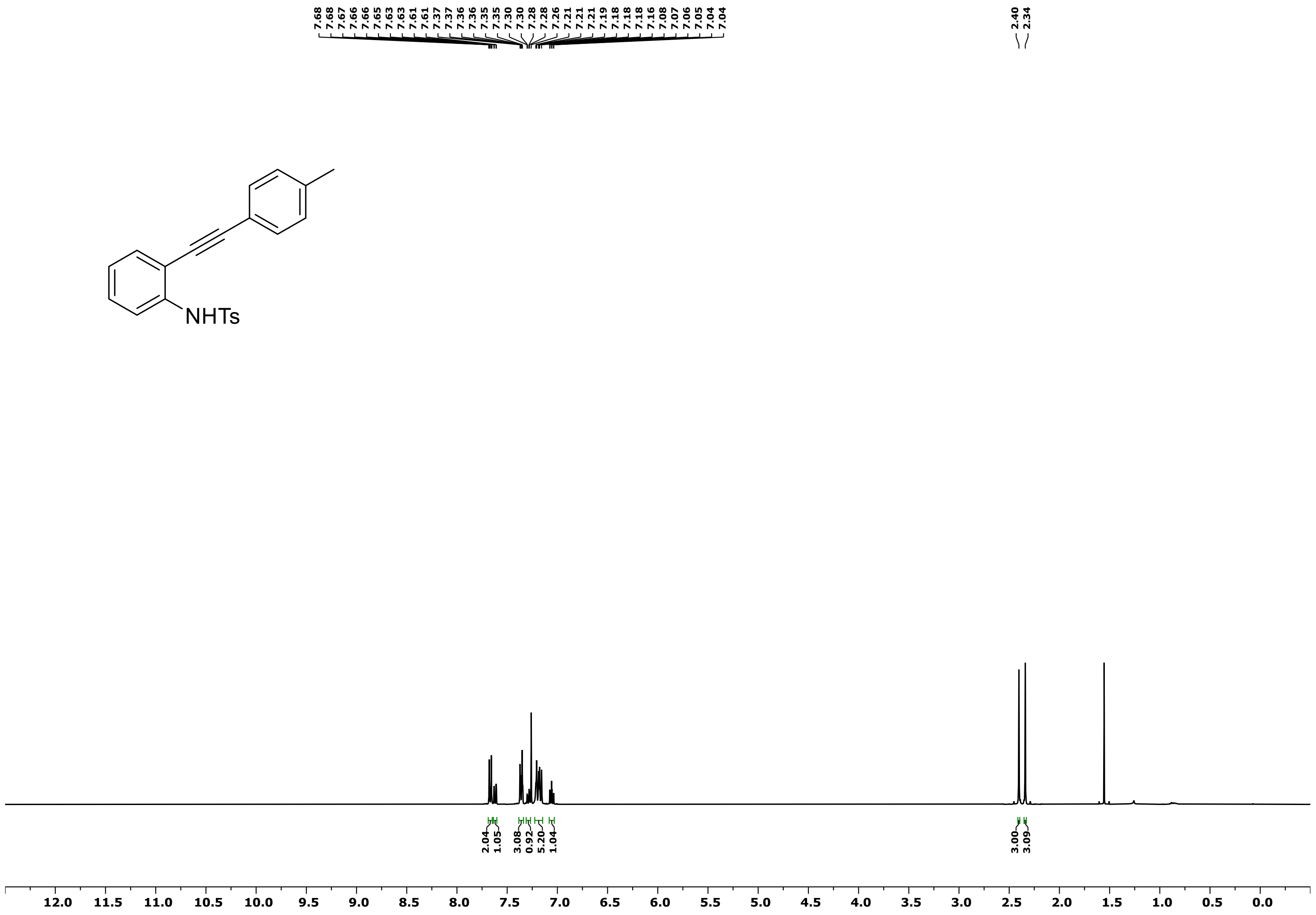

Figure S13. ^{13}C NMR (101 MHz, $CDCl_3$, 298 K) spectrum of *4-methyl-N-(2-(p-tolylethynyl) phenyl)benzenesulfonamide,* **1b**.

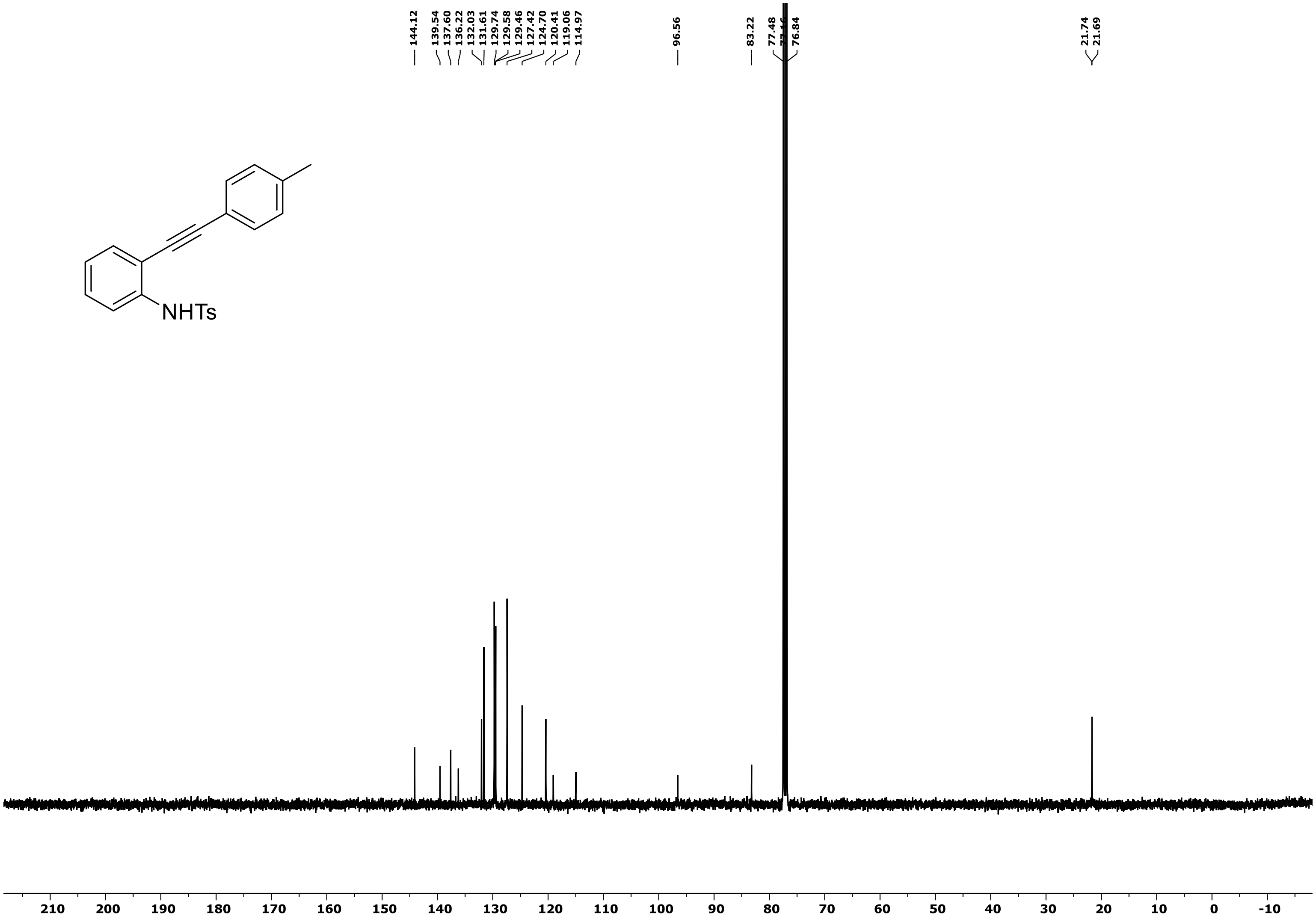

Figure S14. ^{1}H NMR (400 MHz, $CDCl_3$, 298 K) spectrum of *N-(2-([1,1'-biphenyl]-4-ylethynyl)phenyl)-4-methylbenzenesulfonamide*, **1c**.

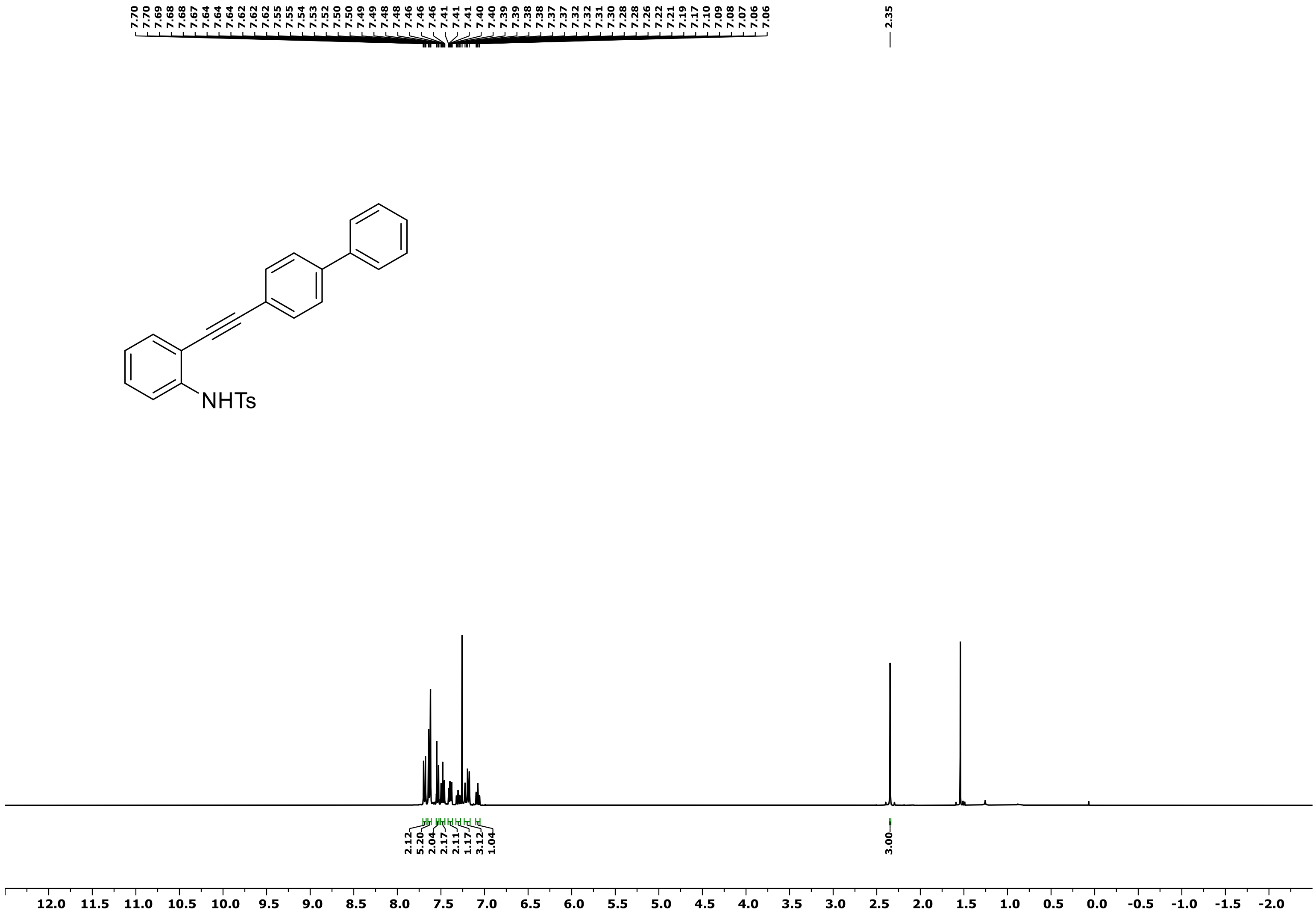

Figure S15. ^{13}C NMR (101 MHz, $CDCl_3$, 298 K) spectrum of *N-(2-([1,1'-biphenyl]-4-ylethynyl)phenyl)-4-methylbenzenesulfonamide*, **1c**.

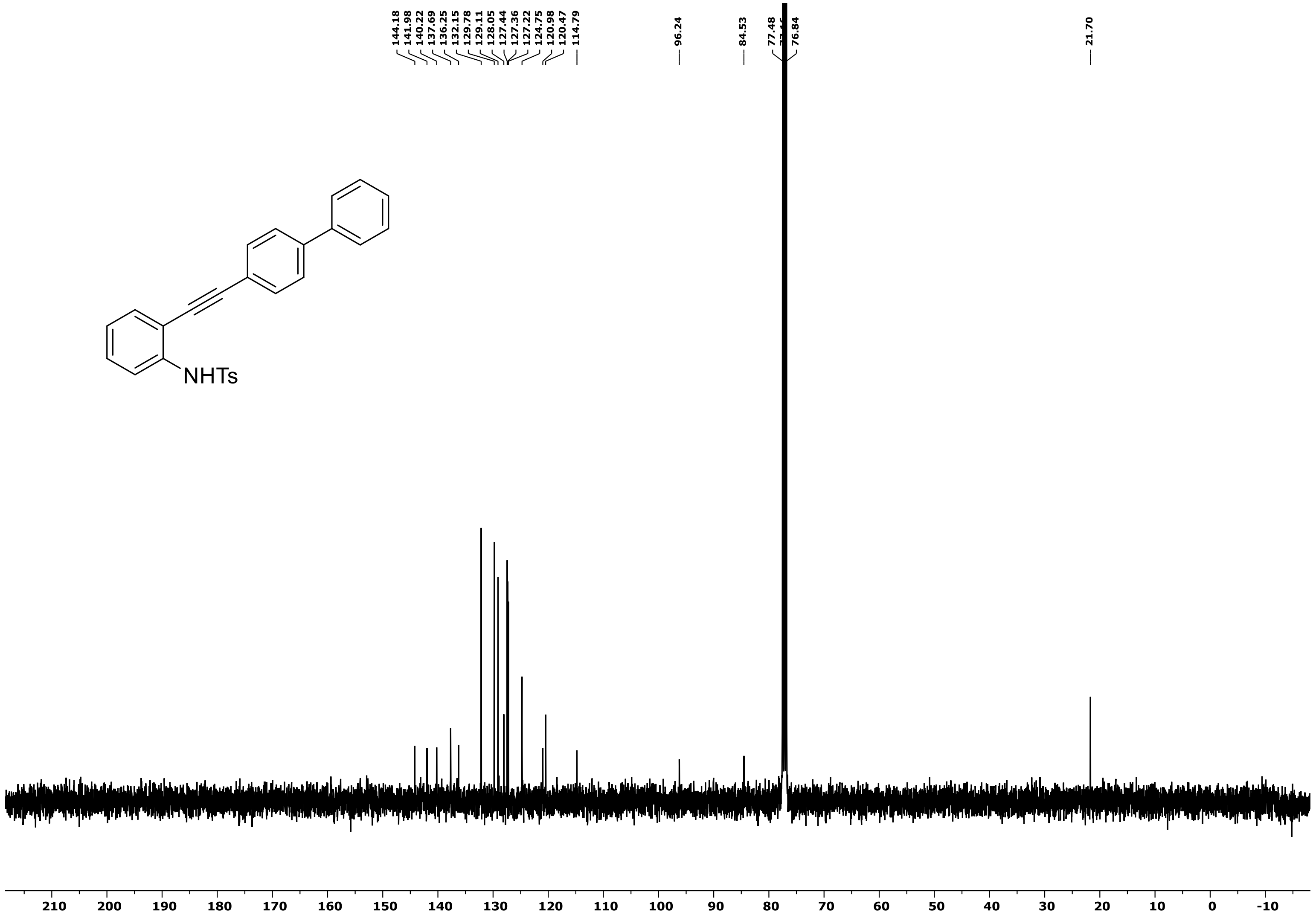

Figure S16. ^{1}H NMR (400 MHz, $CDCl_3$, 298 K) spectrum of *4-methyl-N-(2-(phenanthren-9-ylethynyl)phenyl)benzenesulfonamide*, **1d**.

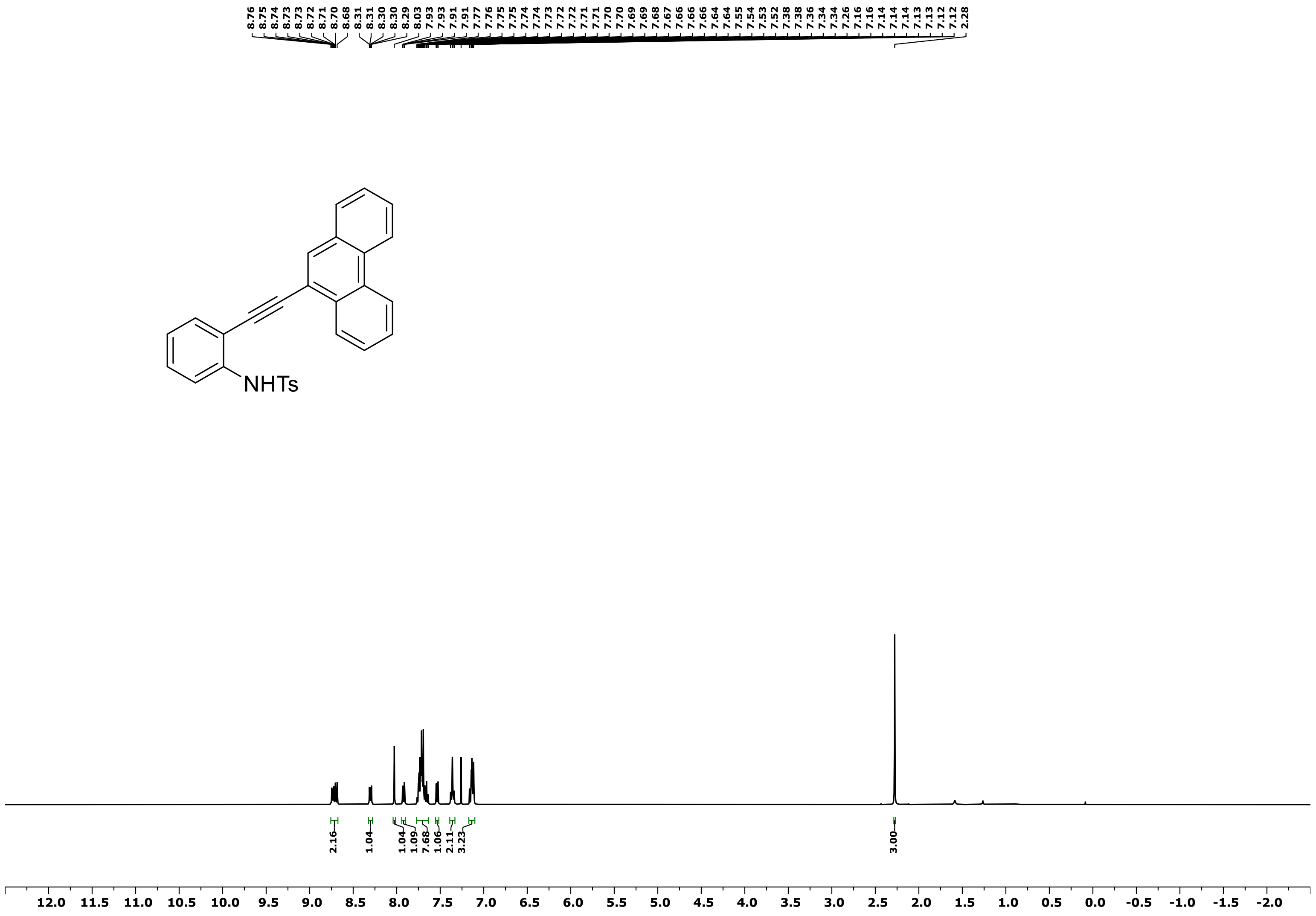

Figure S17. ^{13}C NMR (101 MHz, $CDCl_3$, 298 K) spectrum of *4-methyl-N-(2-(phenanthren-9-ylethynyl)phenyl)benzenesulfonamide*, **1d**.

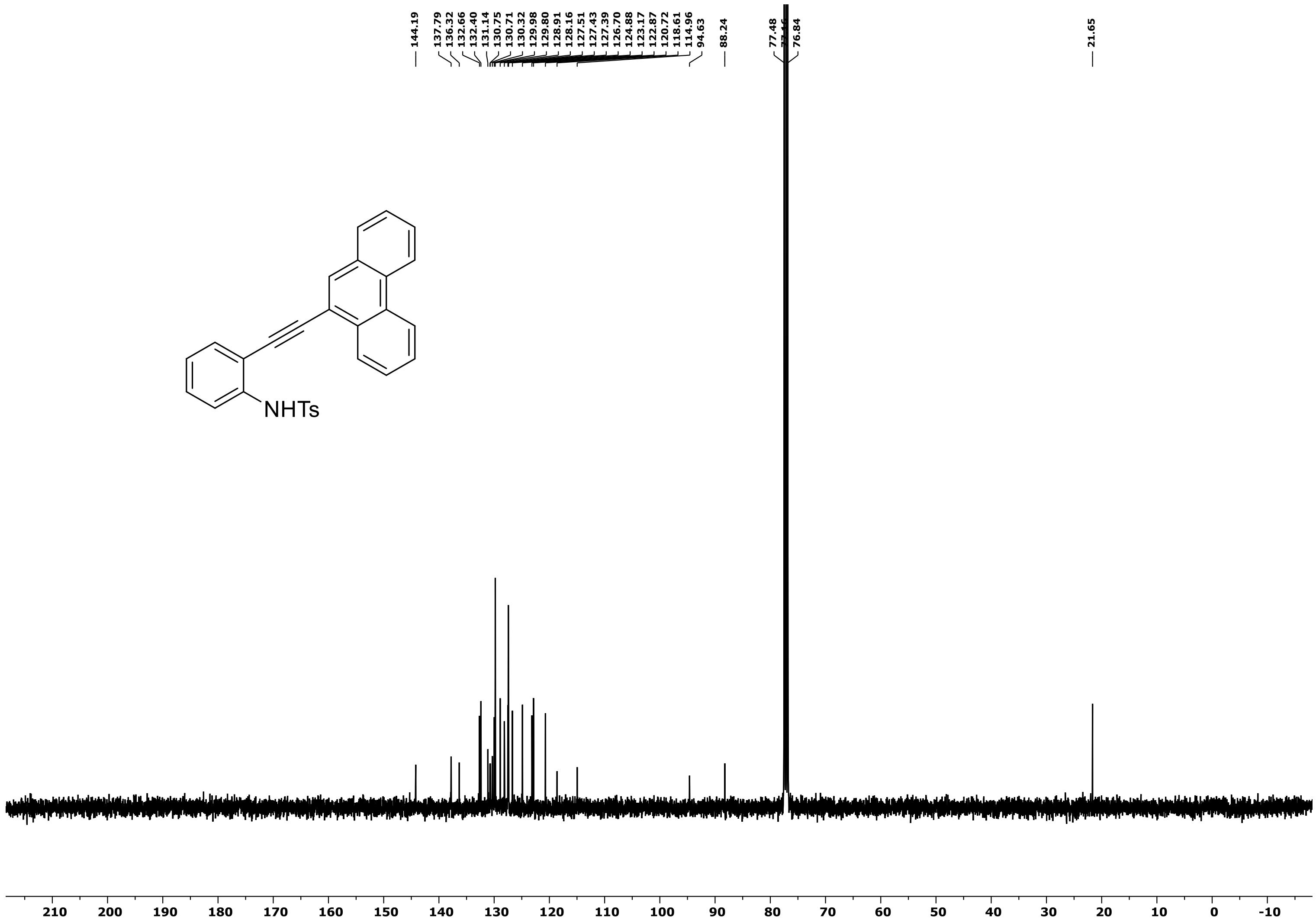

Figure S18. ^{1}H NMR (400 MHz, $CDCl_3$, 298 K) spectrum of *N-(2-((4-(dimethylamino)phenyl)ethynyl)phenyl)-4-methylbenzenesulfonamide*, **1e**.

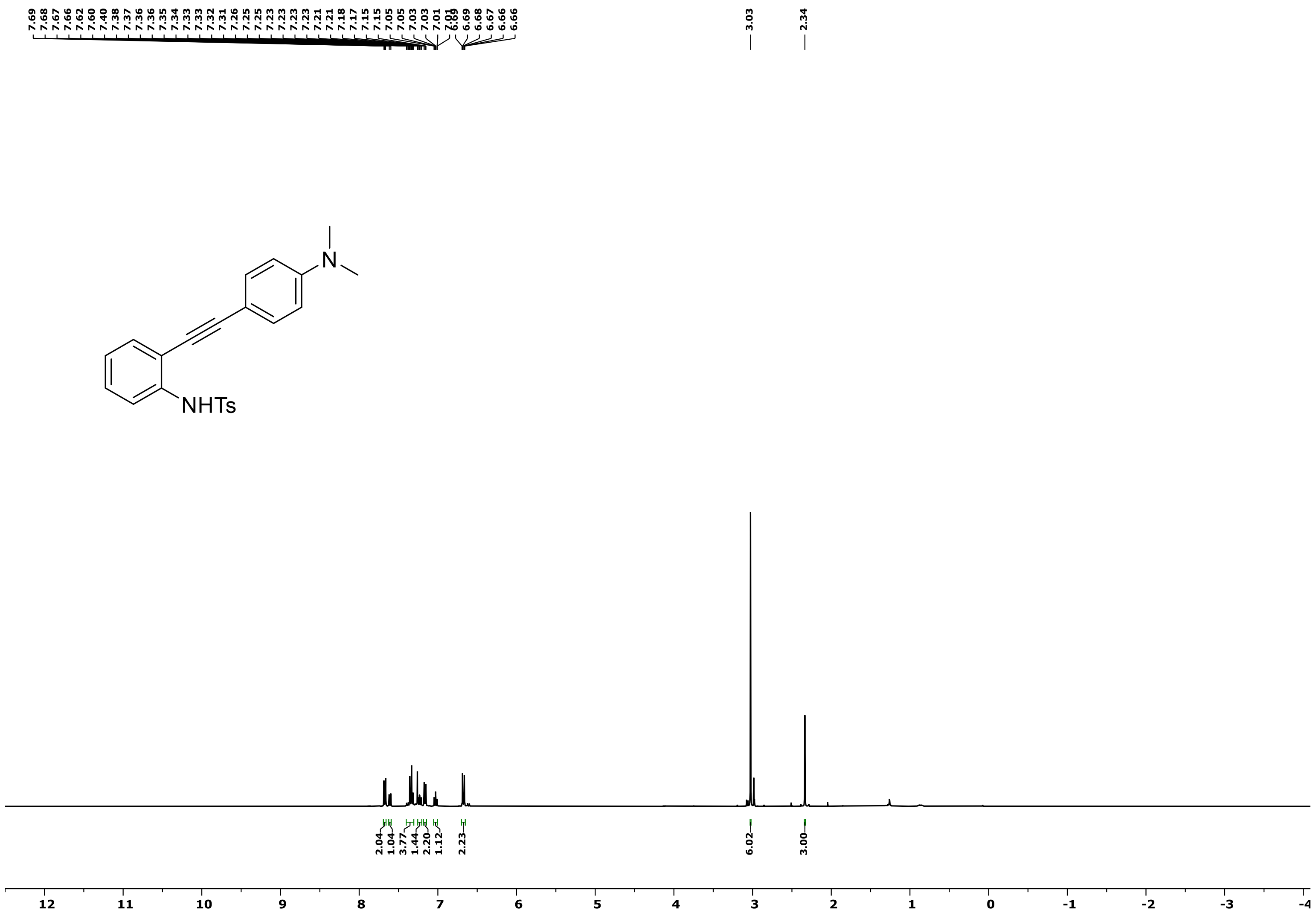

Figure S19. ^{13}C NMR (101 MHz, $CDCl_3$, 298 K) spectrum of *N-(2-((4-(dimethylamino)phenyl)ethynyl)phenyl)-4-methylbenzenesulfonamide*, **1e**.

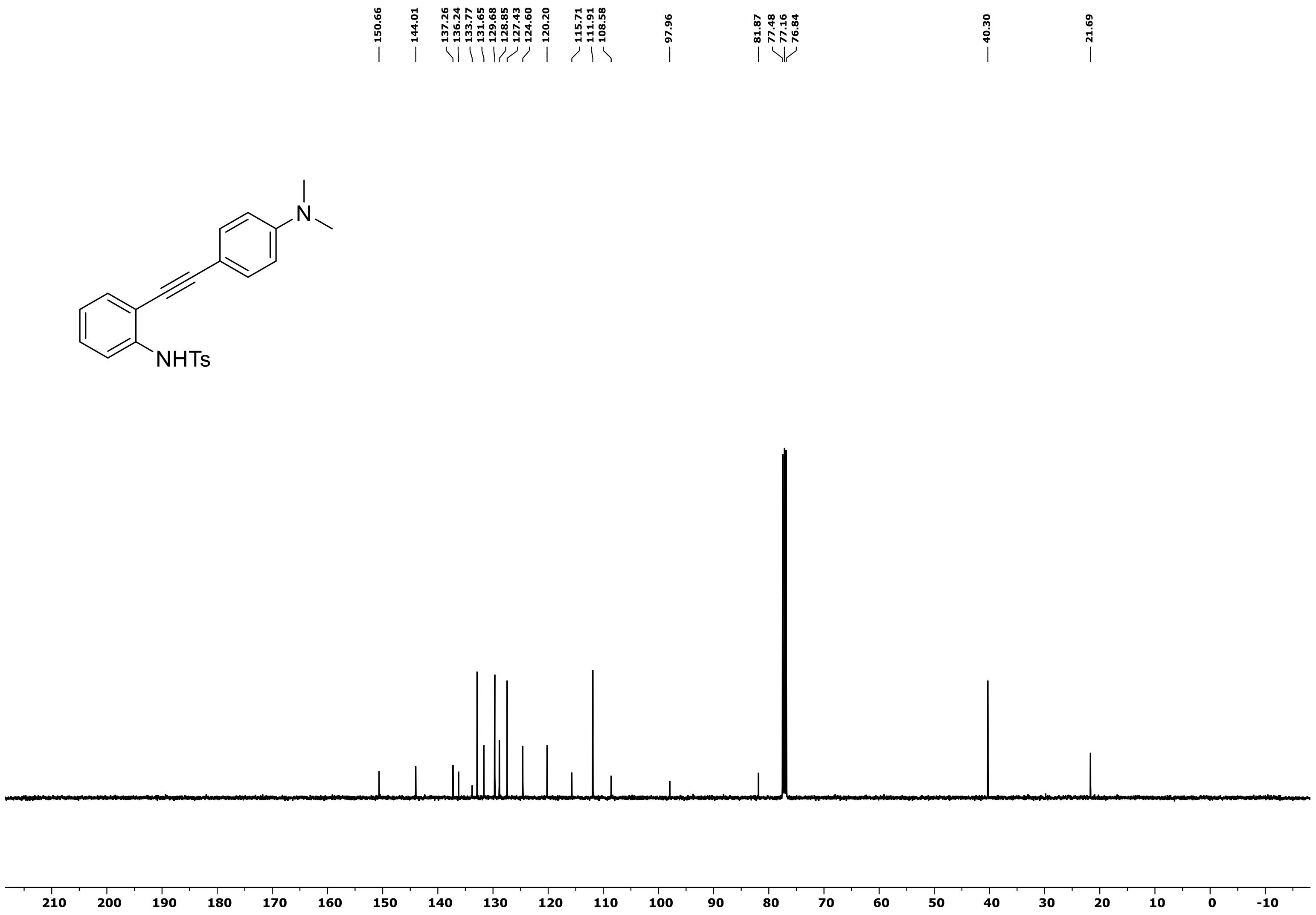

Figure S20. ^{1}H NMR (400 MHz, $CDCl_3$, 298 K) spectrum of *N-(2-((4-bromophenyl)ethynyl)phenyl)-4-methylbenzenesulfonamide*, **1f**.

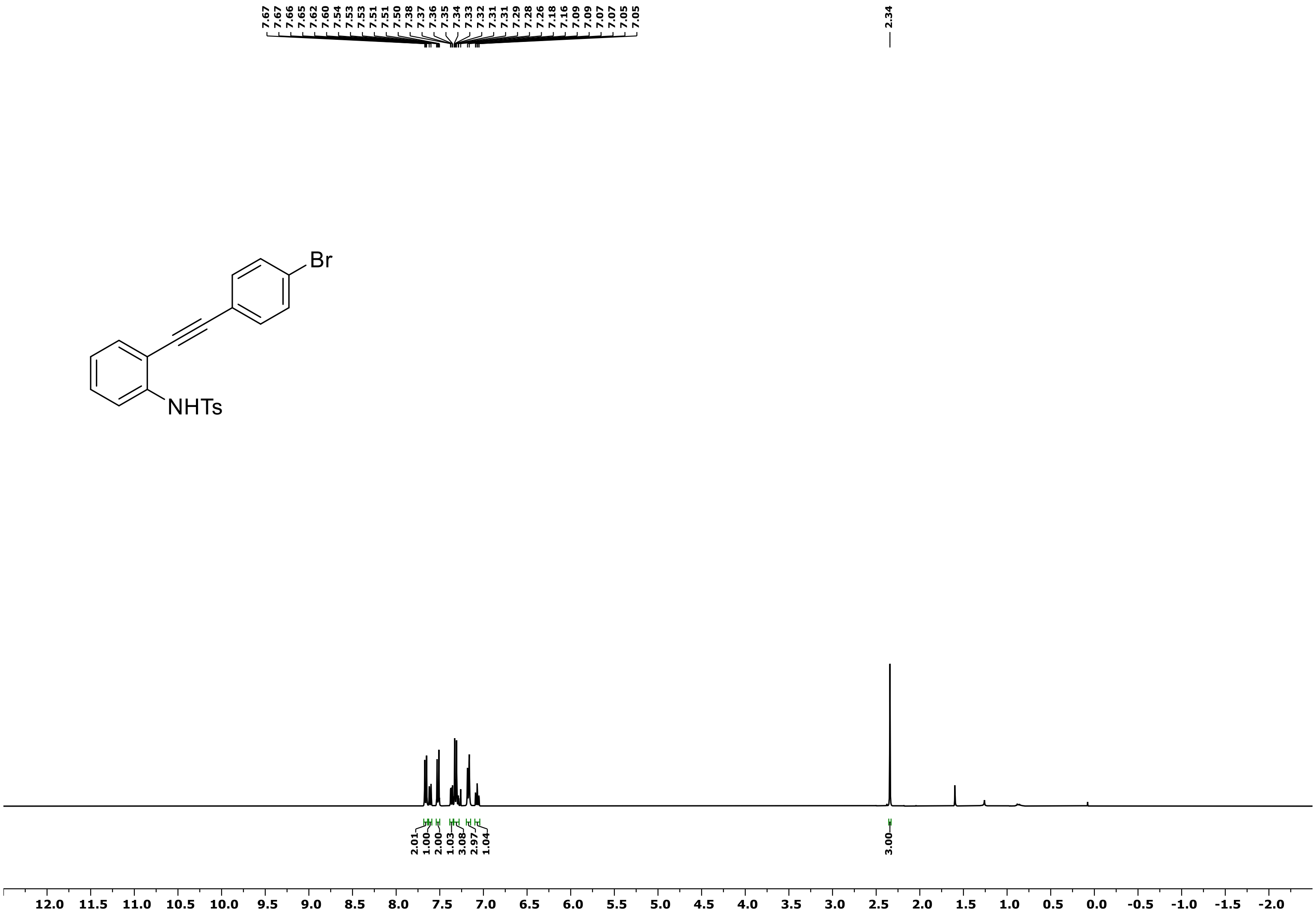

Figure S21. ^{13}C NMR (101 MHz, $CDCl_3$, 298 K) spectrum of *N-(2-((4-bromophenyl)ethynyl)phenyl)-4-methylbenzenesulfonamide*, **1f**.

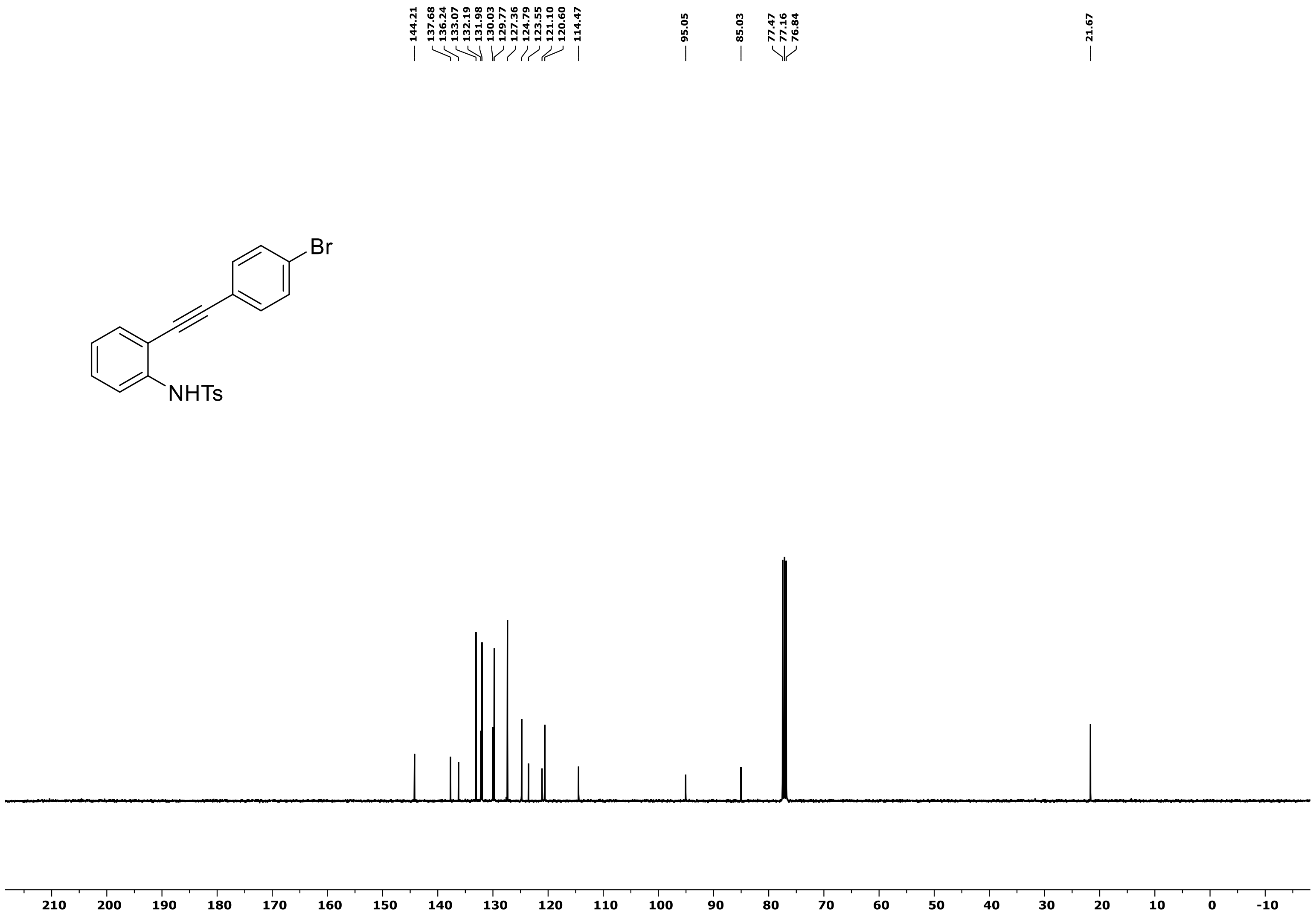

Figure S22. ^{1}H NMR (400 MHz, $CDCl_3$, 298 K) spectrum of *4-methyl-N-(2-((4-(trifluoromethyl)phenyl)ethynyl)phenyl)benzenesulfonamide*, **1g**

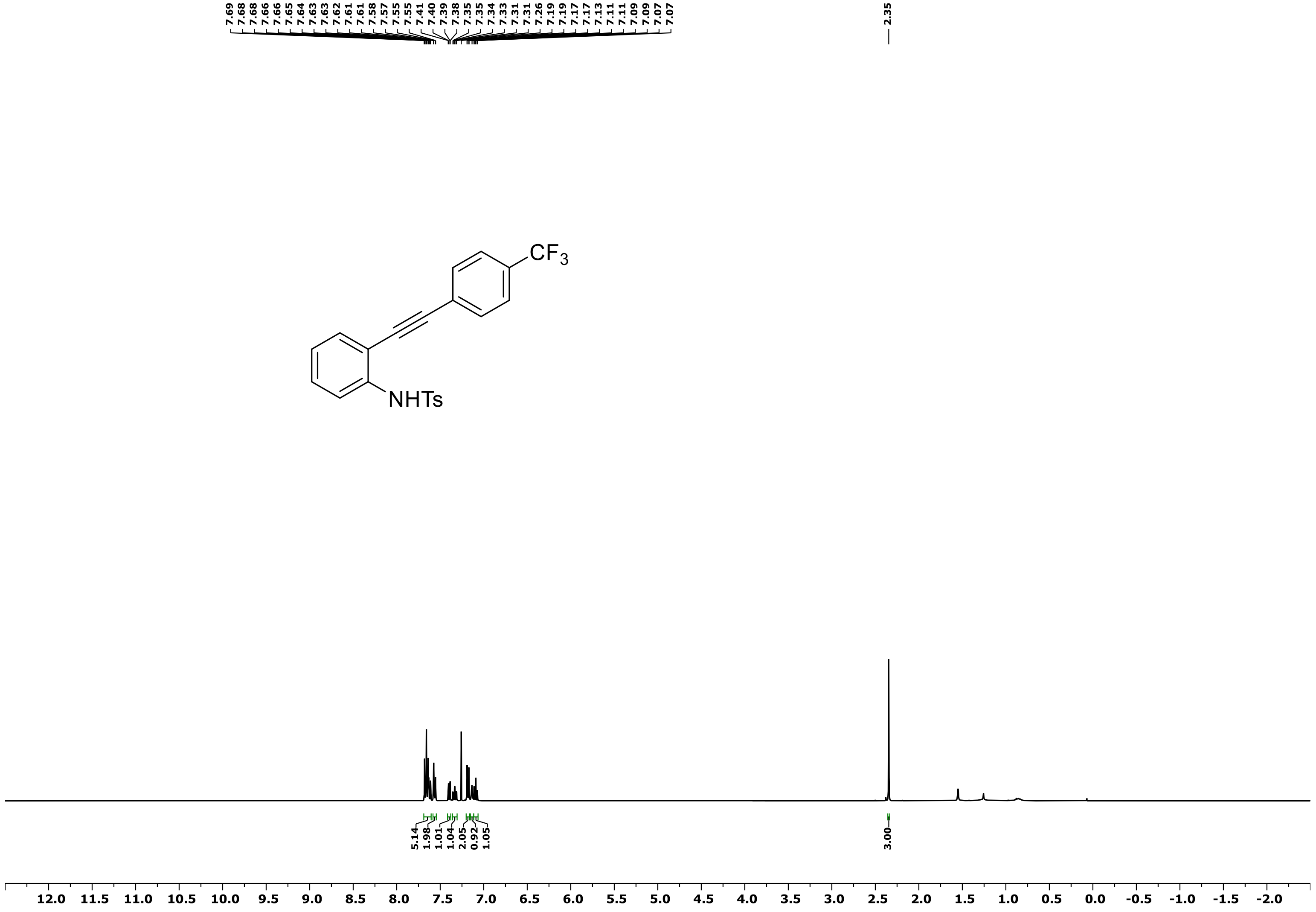

Figure S23. ^{13}C NMR (101 MHz, $CDCl_3$, 298 K) spectrum of *4-methyl-N-(2-((4- (trifluoromethyl)phenyl)ethynyl)phenyl)benzenesulfonamide*, **1g**.

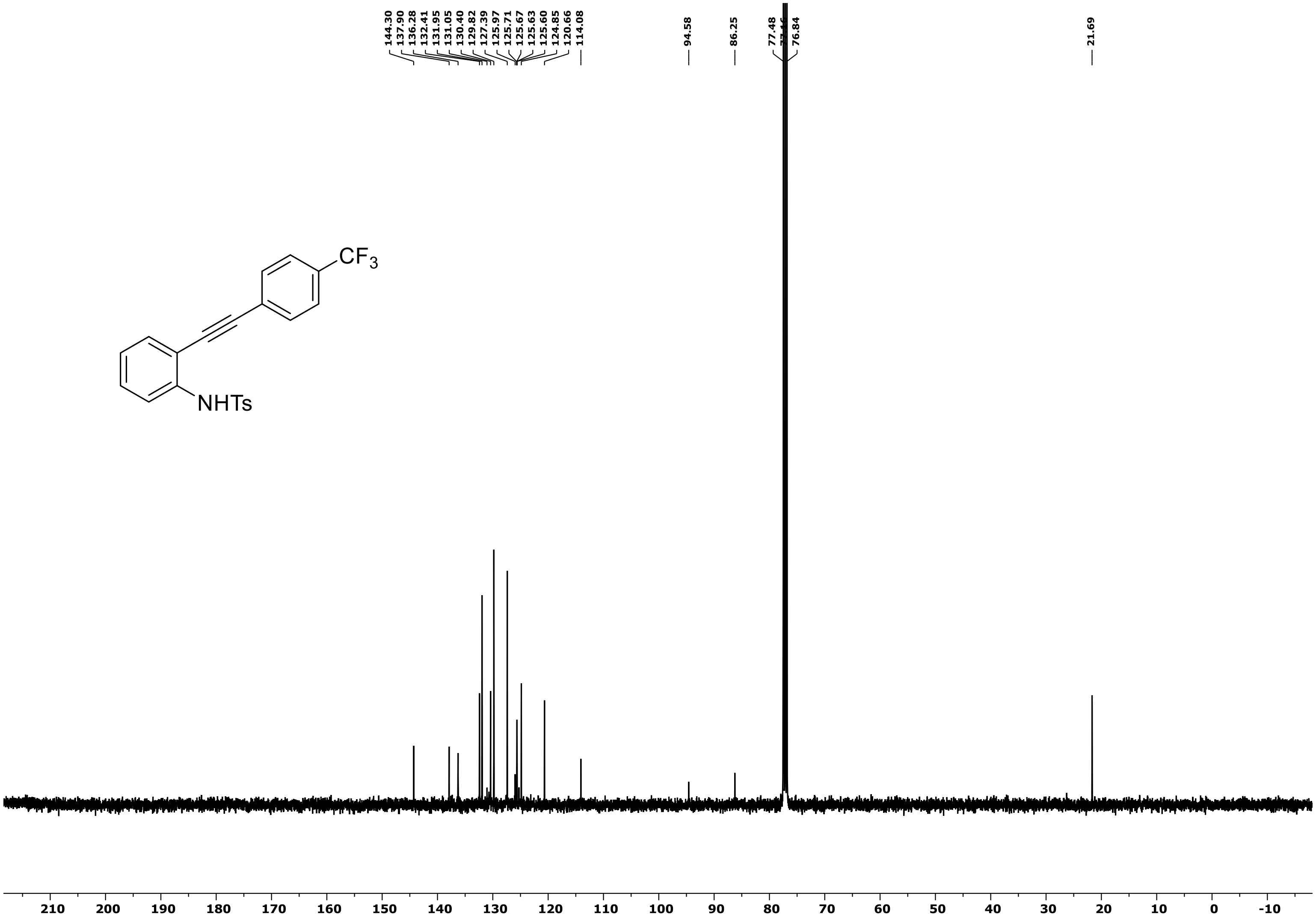

Figure S24. ^{19}F NMR (376 MHz, $CDCl_3$, 298 K) spectrum of *4-methyl-N-(2-((4- (trifluoromethyl)phenyl)ethynyl)phenyl)benzenesulfonamide*, **1g**.

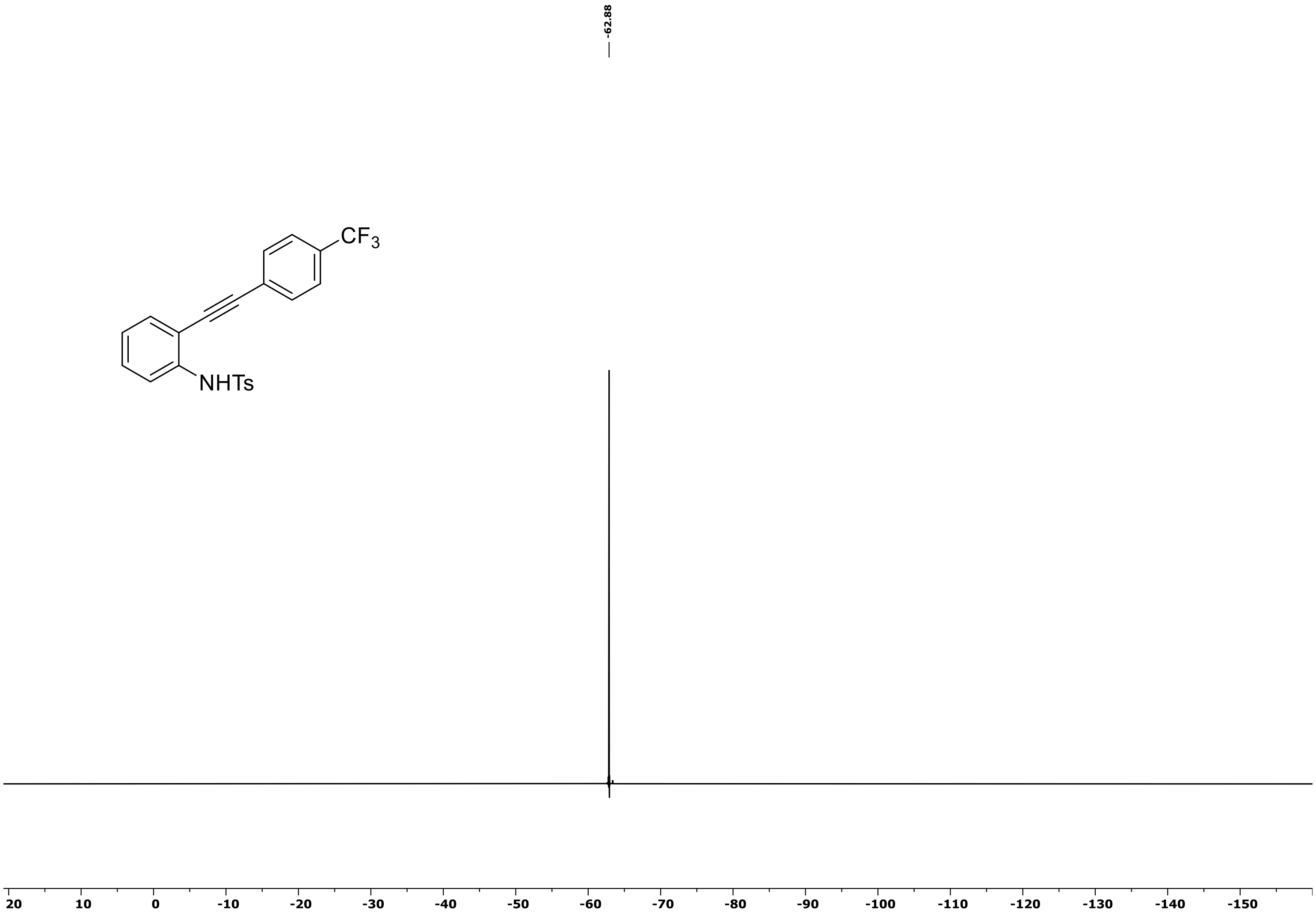

Figure S25. ^{1}H NMR (400 MHz, $CDCl_3$, 298 K) spectrum of *N-(2-(cyclohex-1-en-1-ylethynyl)phenyl)-4-methylbenzenesulfonamide*, **1h**.

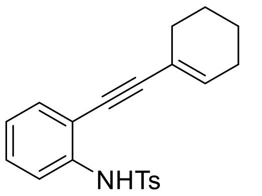

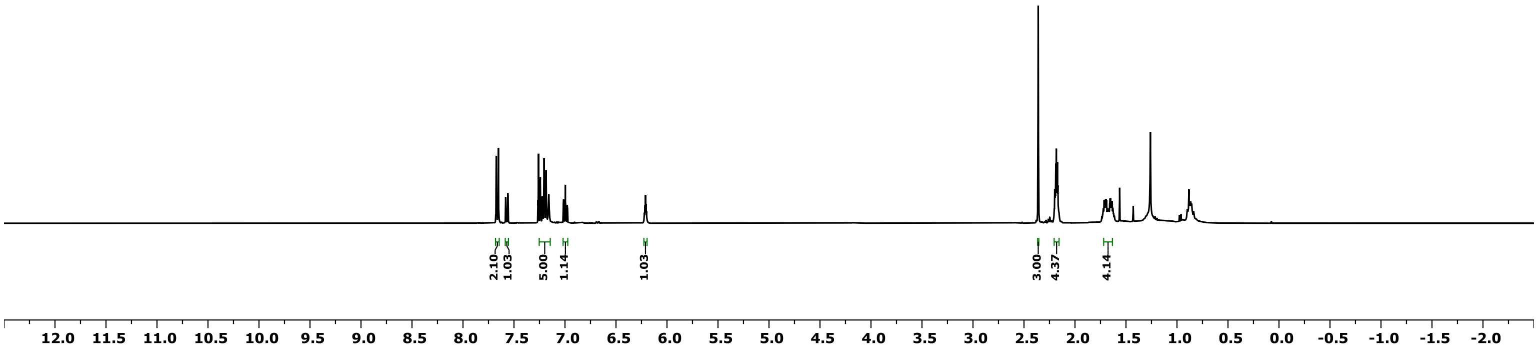

Figure S26. ^{13}C NMR (101 MHz, $CDCl_3$, 298 K) spectrum of *N-(2-(cyclohex-1-en-1-ylethynyl)phenyl)-4-methylbenzenesulfonamide*, **1h**.

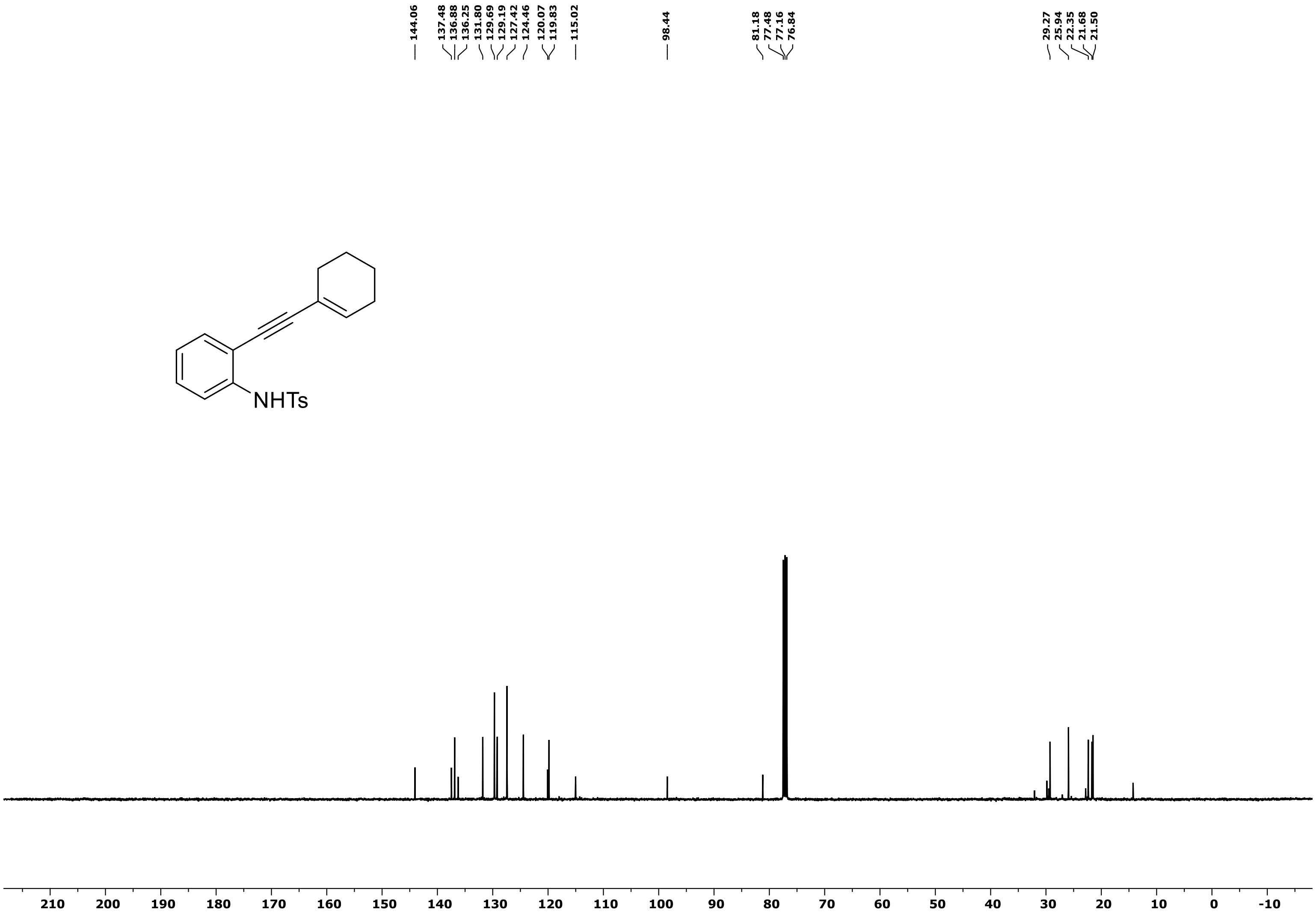

Figure S27. ^{1}H NMR (400 MHz, $CDCl_3$, 298 K) spectrum of *N-(2-(hex-1-yn-1-yl)phenyl)-4-methylbenzenesulfonamide*, **1i**.

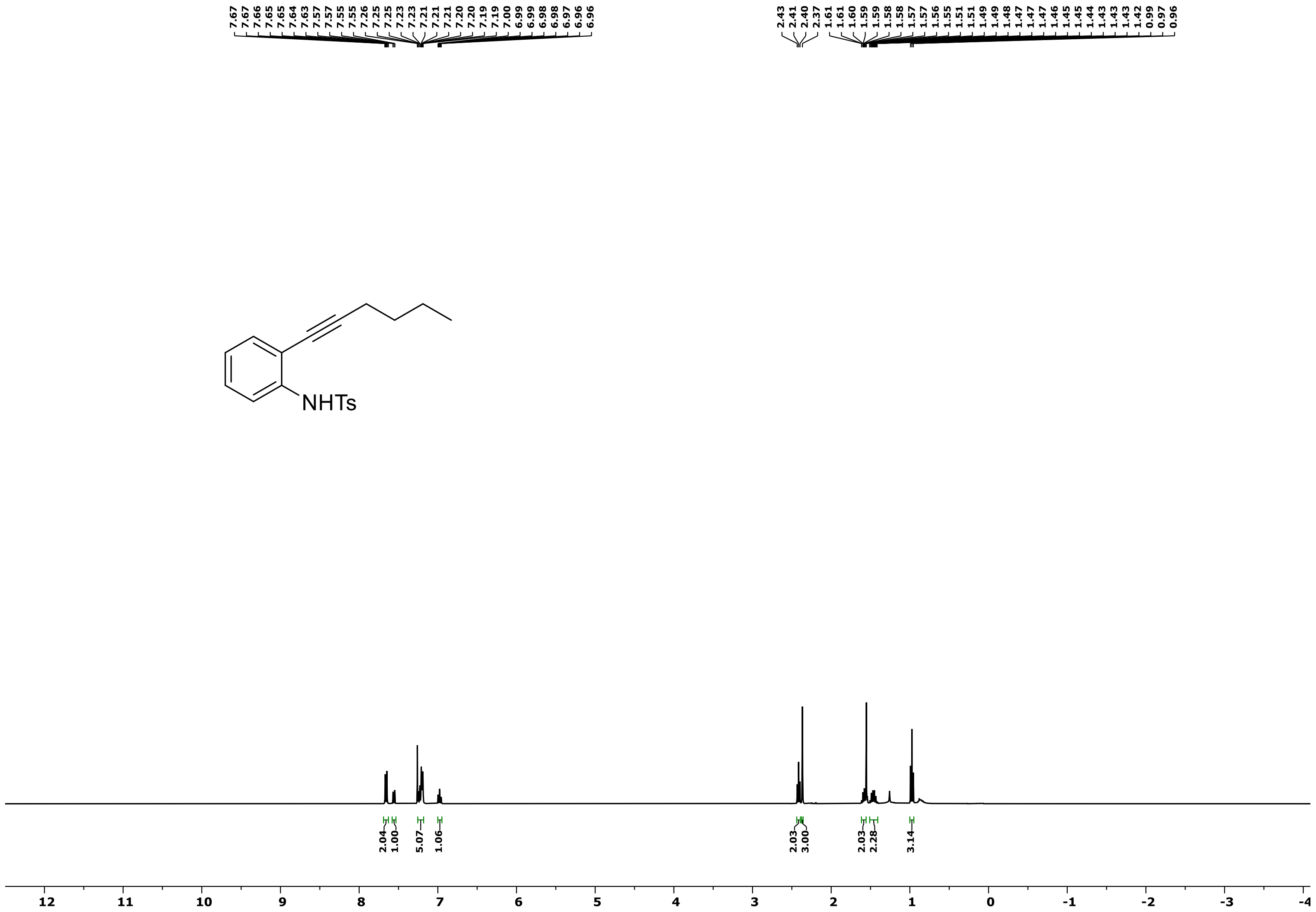

Figure S28. ^{13}C NMR (101 MHz, $CDCl_3$, 298 K) spectrum of *N-(2-(hex-1-yn-1-yl)phenyl)-4-methylbenzenesulfonamide*, **1i**.

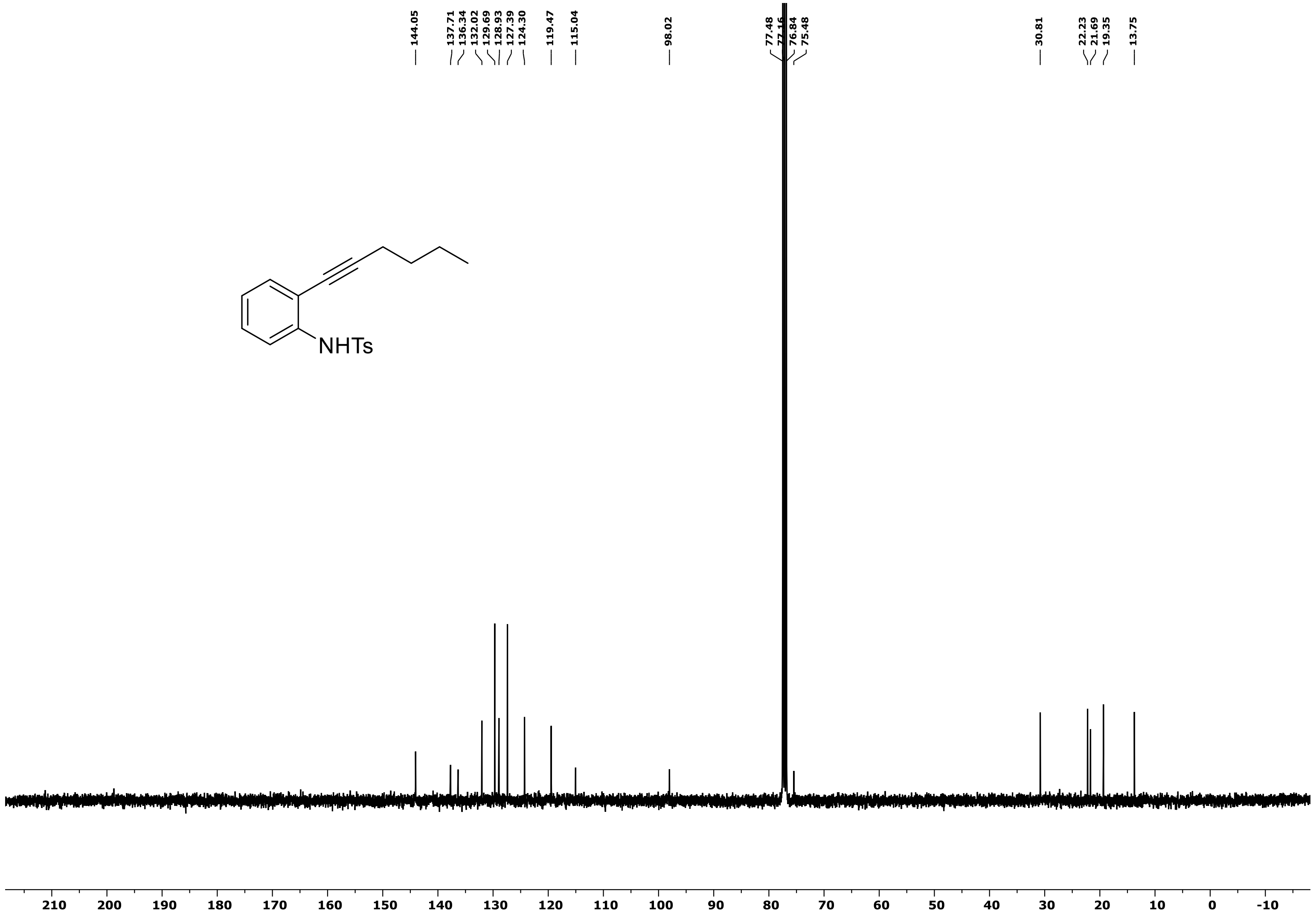

Figure S29. ^1H NMR (400 MHz, $CDCl_3$, 298 K) spectrum of *4-methyl-N-(2-((trimethylsilyl)ethynyl)phenyl)benzenesulfonamide*, **1j**.

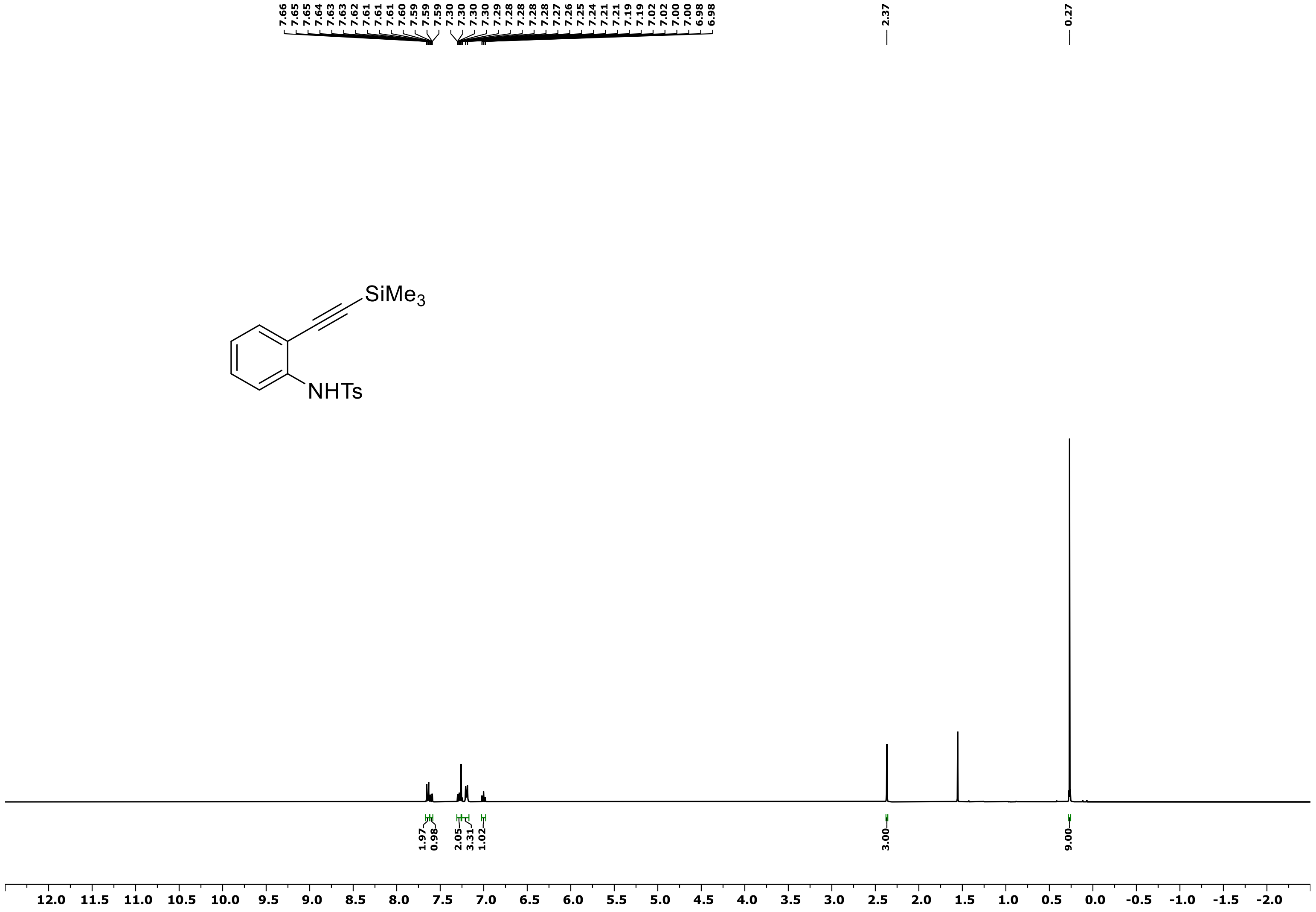

Figure S30. ^{13}C NMR (101 MHz, $CDCl_3$, 298 K) spectrum of *4-methyl-N-(2-((trimethylsilyl)ethynyl)phenyl)benzenesulfonamide*, **1j**.

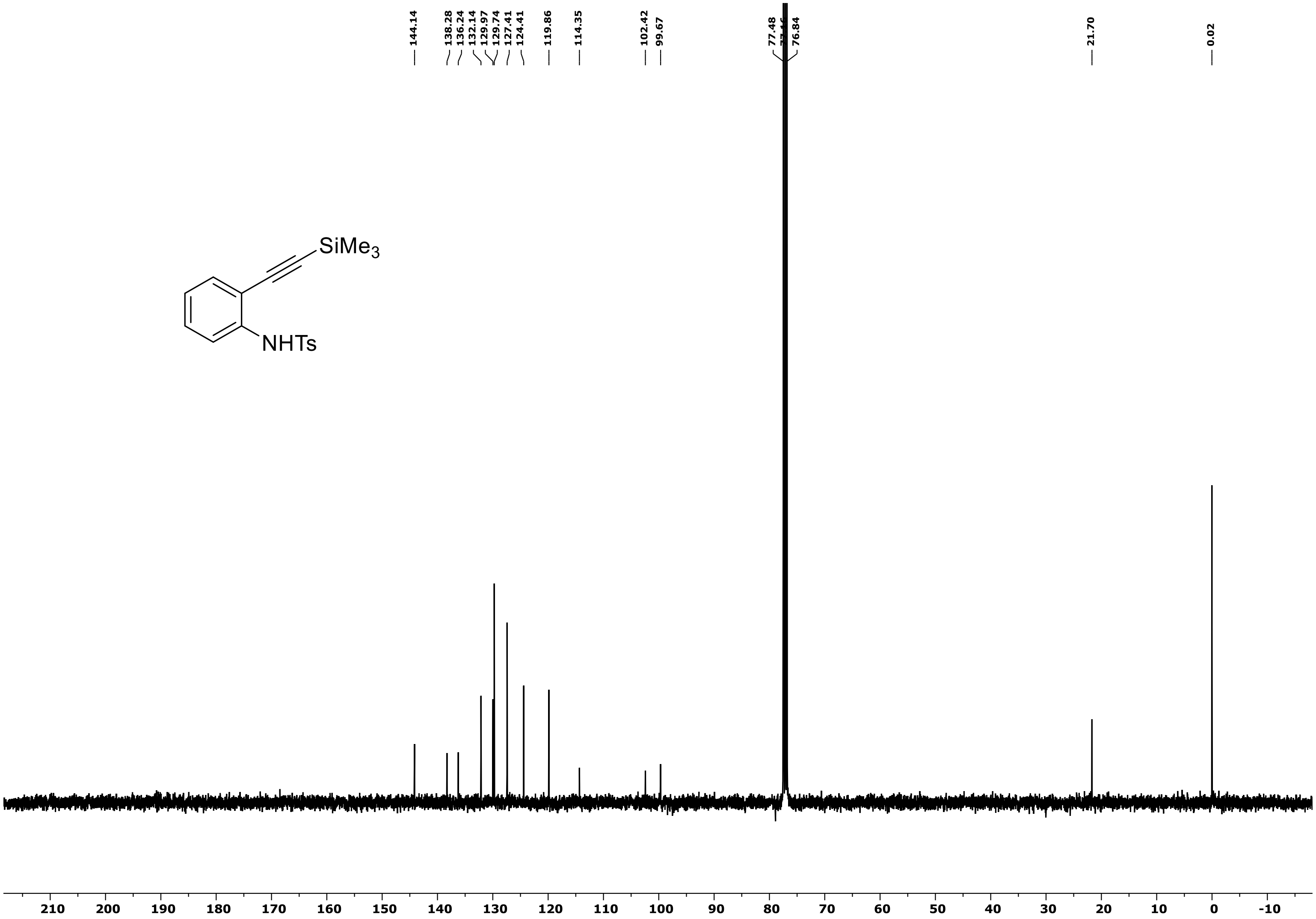

Figure S31. ^{1}H NMR (400 MHz, $CDCl_3$, 298 K) spectrum of *2-(phenylethynyl)phenol*, **1k**.

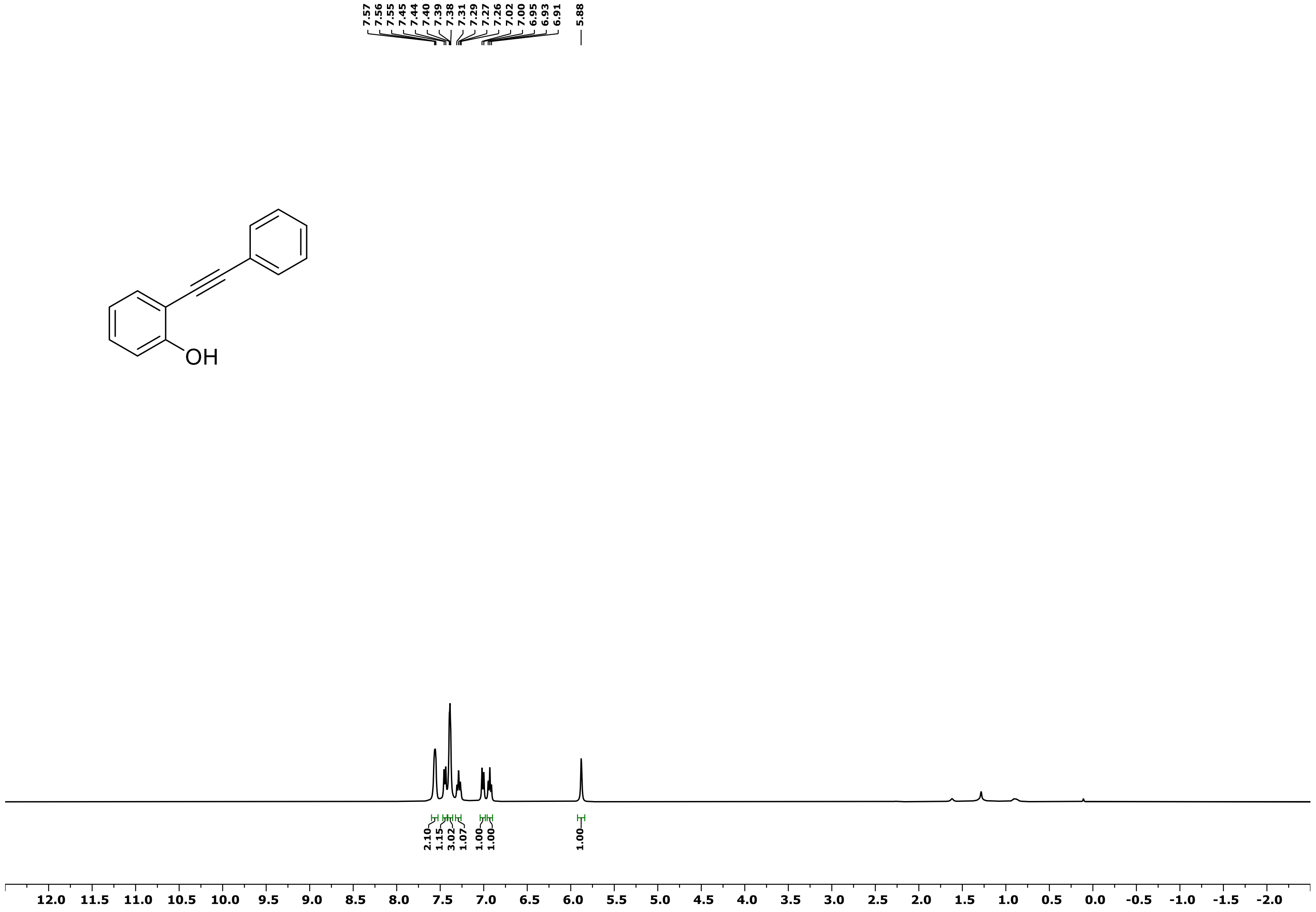

Figure S32. ^{13}C NMR (101 MHz, $CDCl_3$, 298 K) spectrum of *2-(phenylethynyl)phenol*, **1k**.

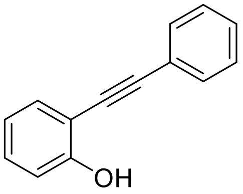

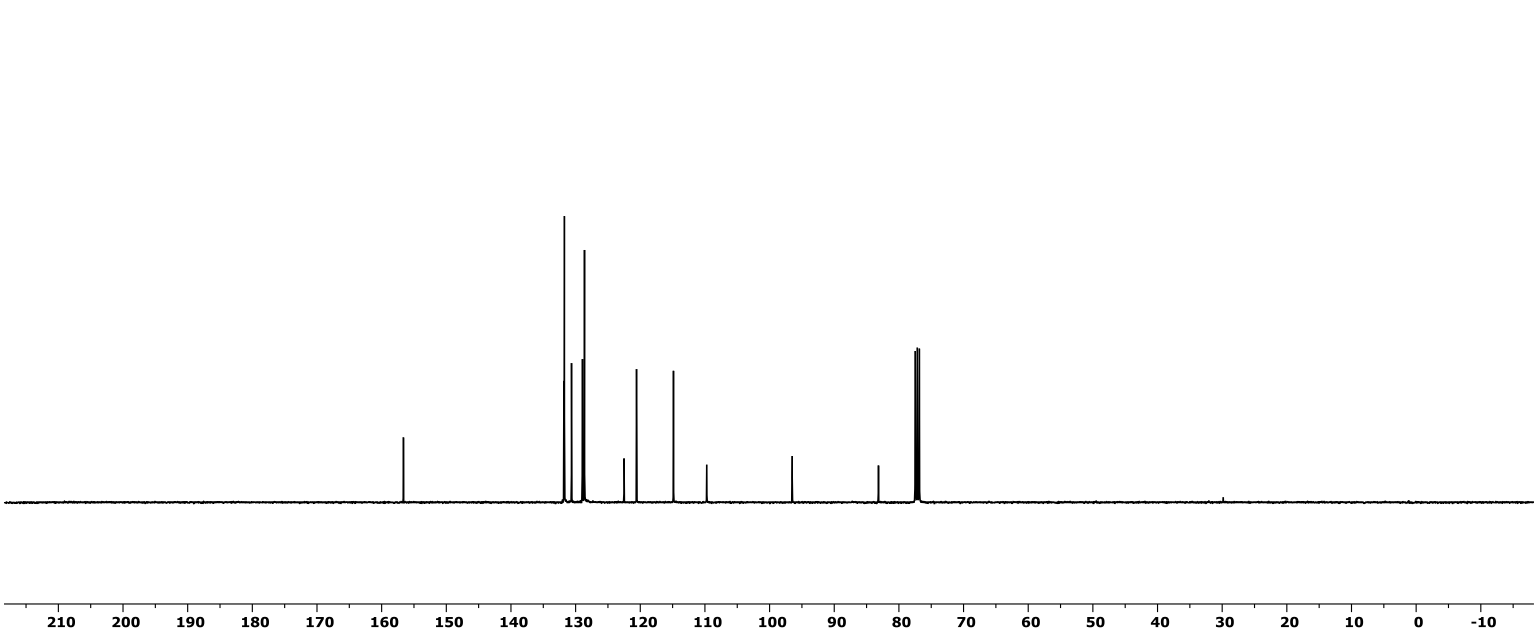

Figure S33. ^{1}H NMR (400 MHz, $CDCl_3$, 298 K) spectrum of *4-methyl-2-(phenylethynyl)phenol,* **1l**.

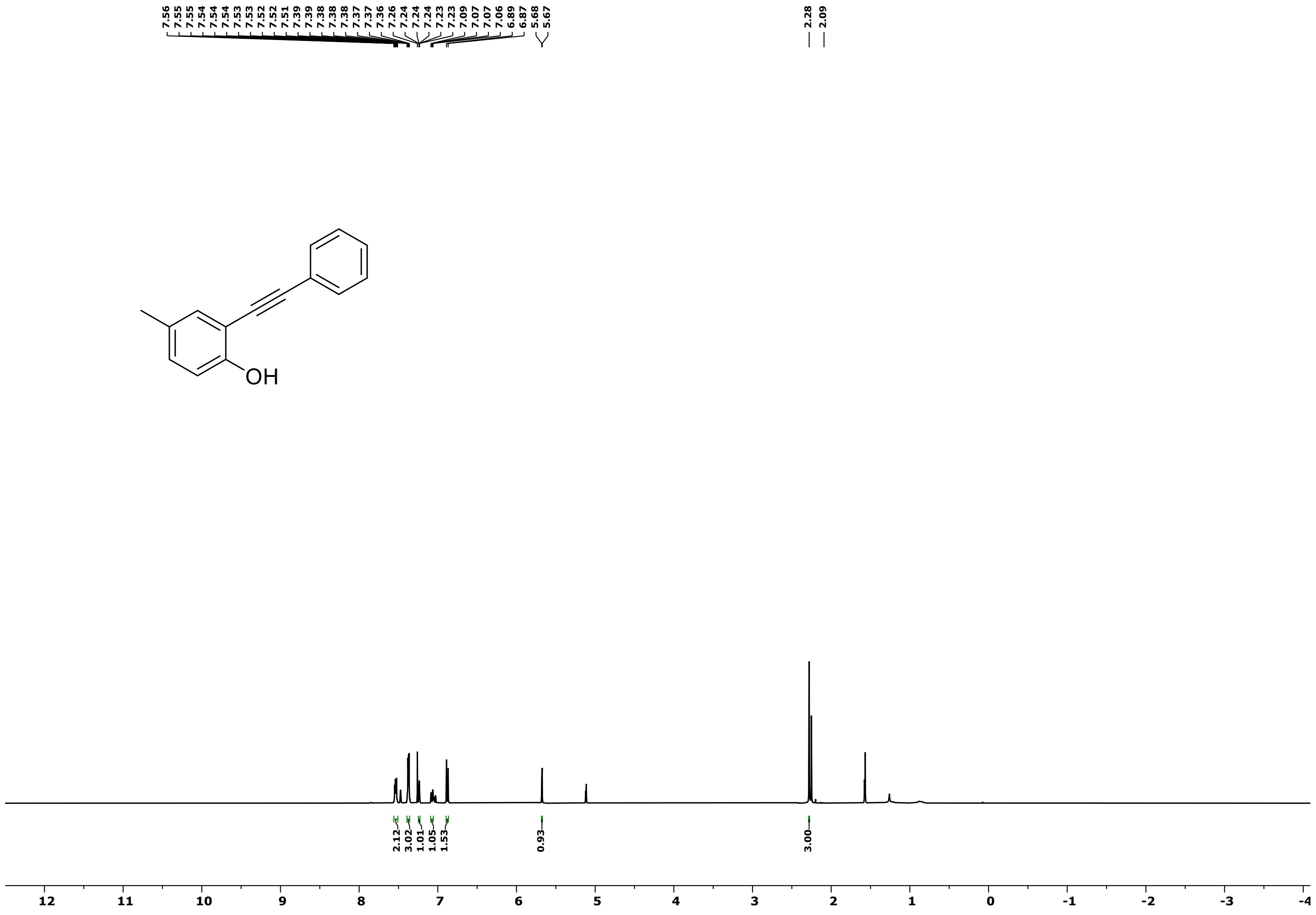

Figure S34. ^{13}C NMR (101 MHz, $CDCl_3$, 298 K) spectrum of *4-methyl-2-(phenylethynyl)phenol*, **1l**.

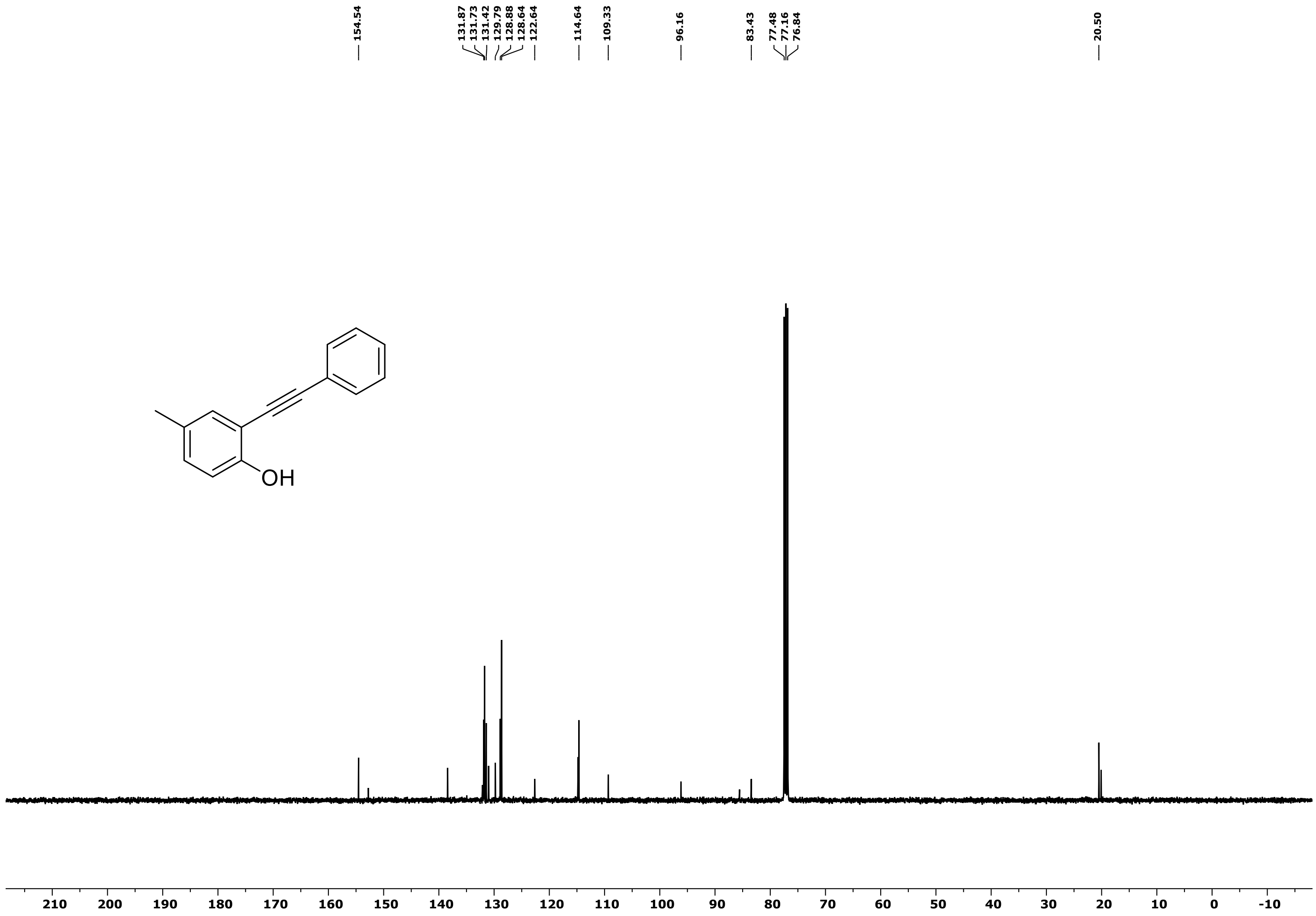

Figure S35. ^{1}H NMR (400 MHz, $CDCl_3$, 298 K) spectrum of *4-chloro-2-(phenylethynyl)phenol*, **1m**.

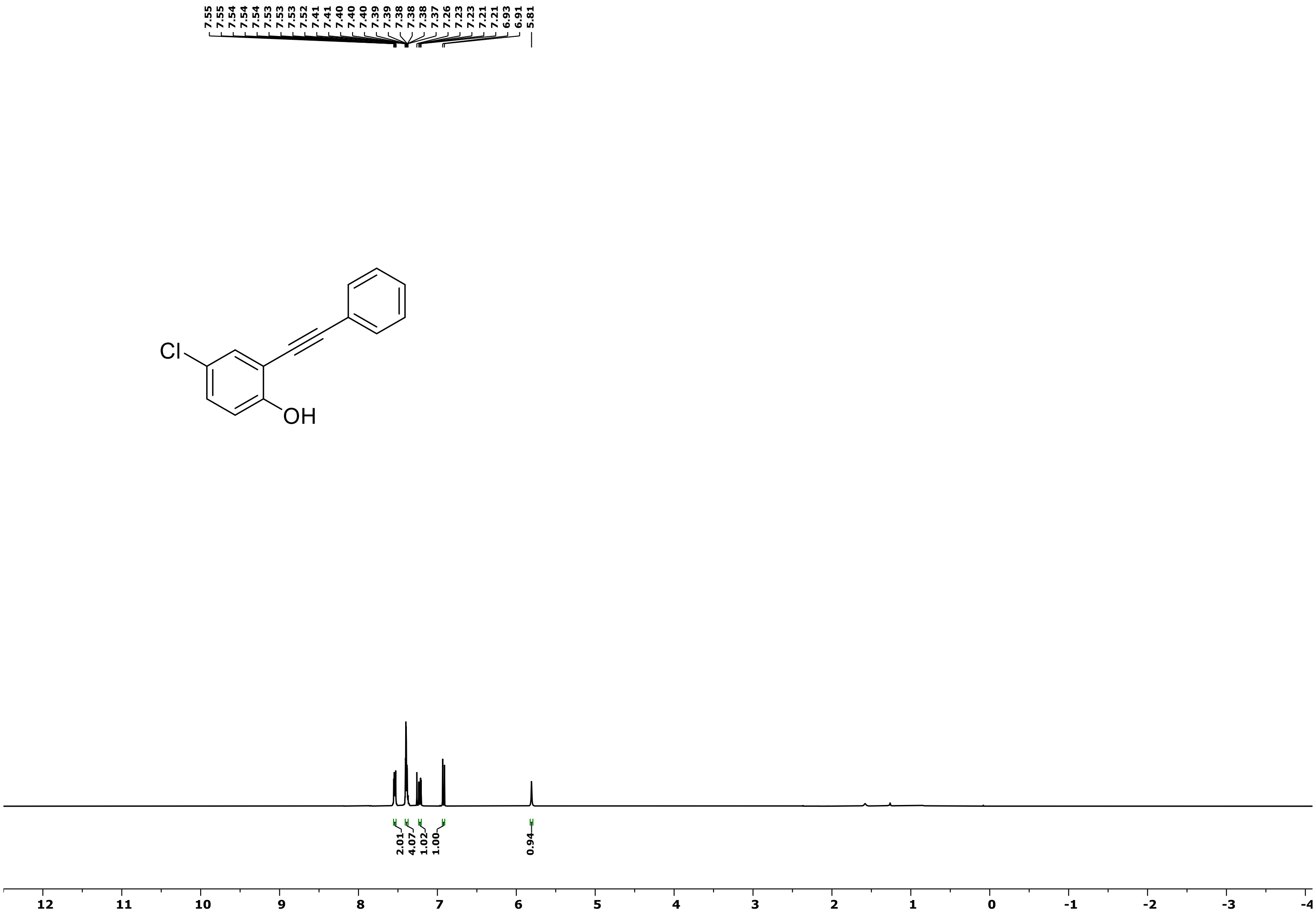

Figure S36. ^{13}C NMR (101 MHz, $CDCl_3$, 298 K) spectrum of *4-chloro-2-(phenylethynyl)phenol*, **1m**.

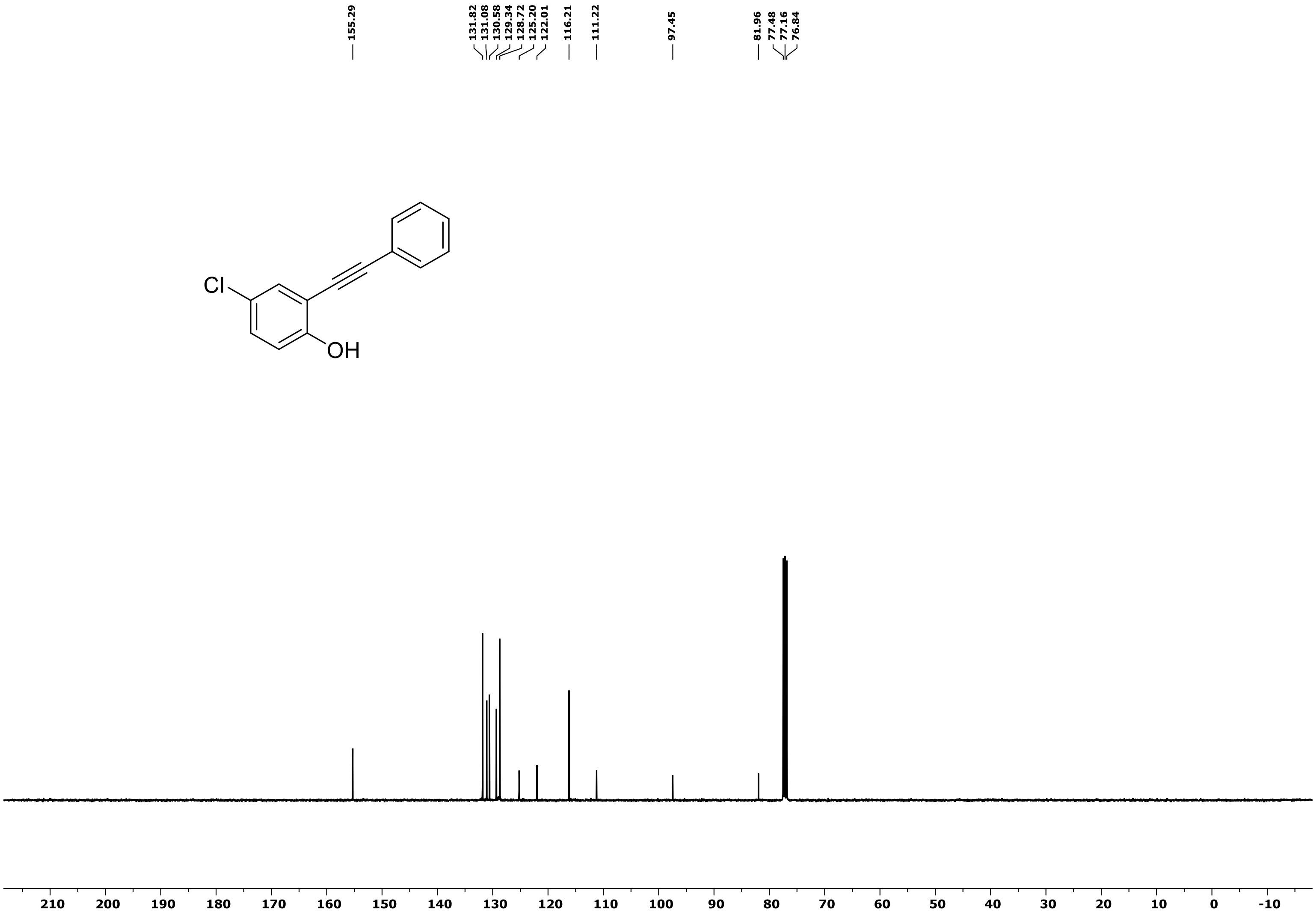

Figure S37. ^{1}H NMR (400 MHz, $CDCl_3$, 298 K) spectrum of *4-fluoro-2-(phenylethynyl)phenol*, **1n**

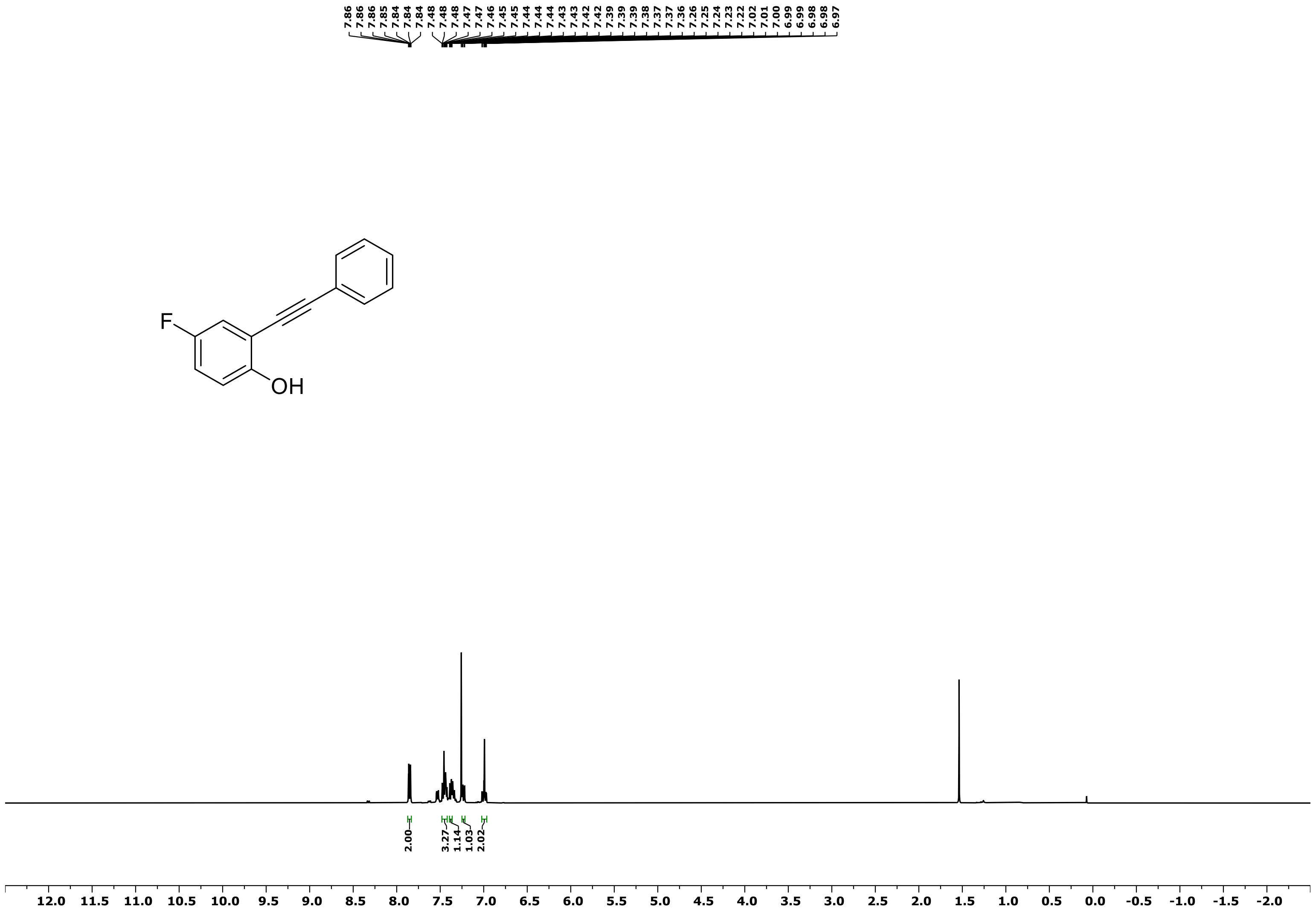

Figure S38. ^{13}C NMR (101 MHz, $CDCl_3$, 298 K) spectrum of *4-fluoro-2-(phenylethynyl)phenol*, **1n**.

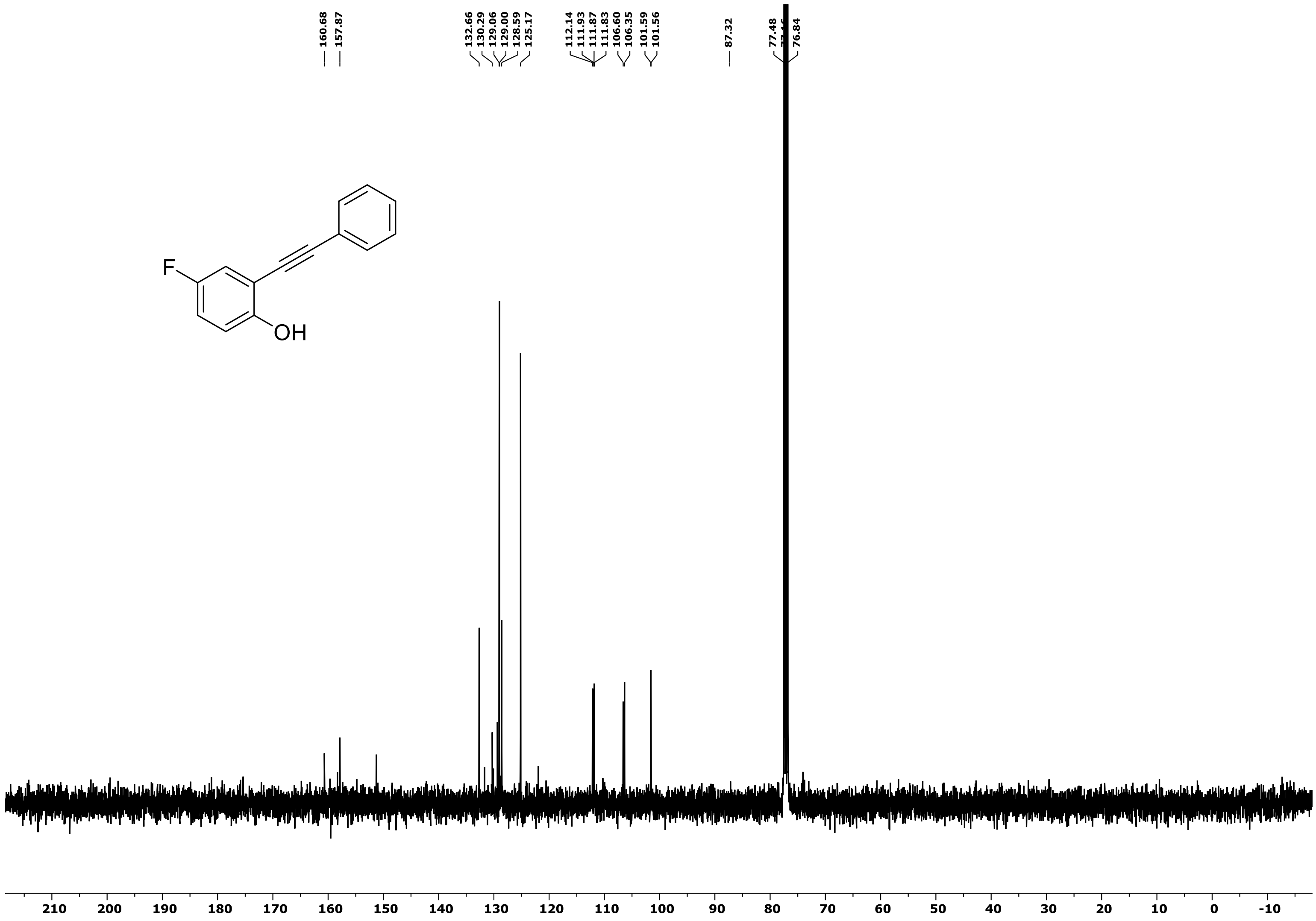

Figure S39. ^{19}F NMR (376 MHz, $CDCl_3$, 298 K) spectrum of *4-fluoro-2-(phenylethynyl)phenol*, **1n**.

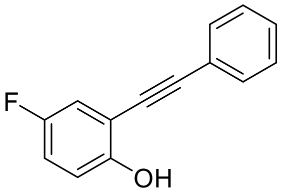

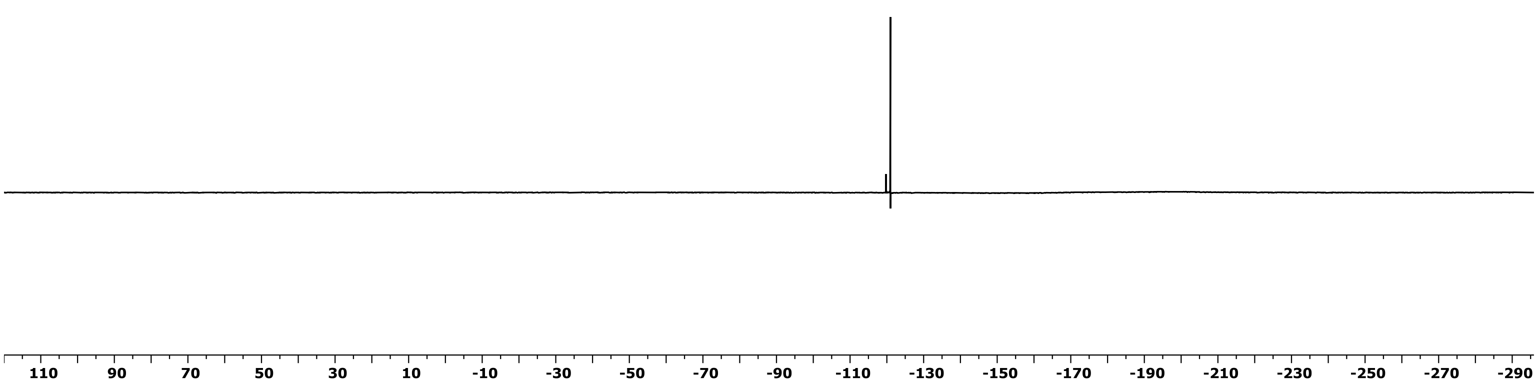

Figure S40: ^{1}H NMR (400 MHz, $CDCl_3$, 298 K) spectrum of *2-(thiophen-3-ylethynyl)phenol*, **1o**.

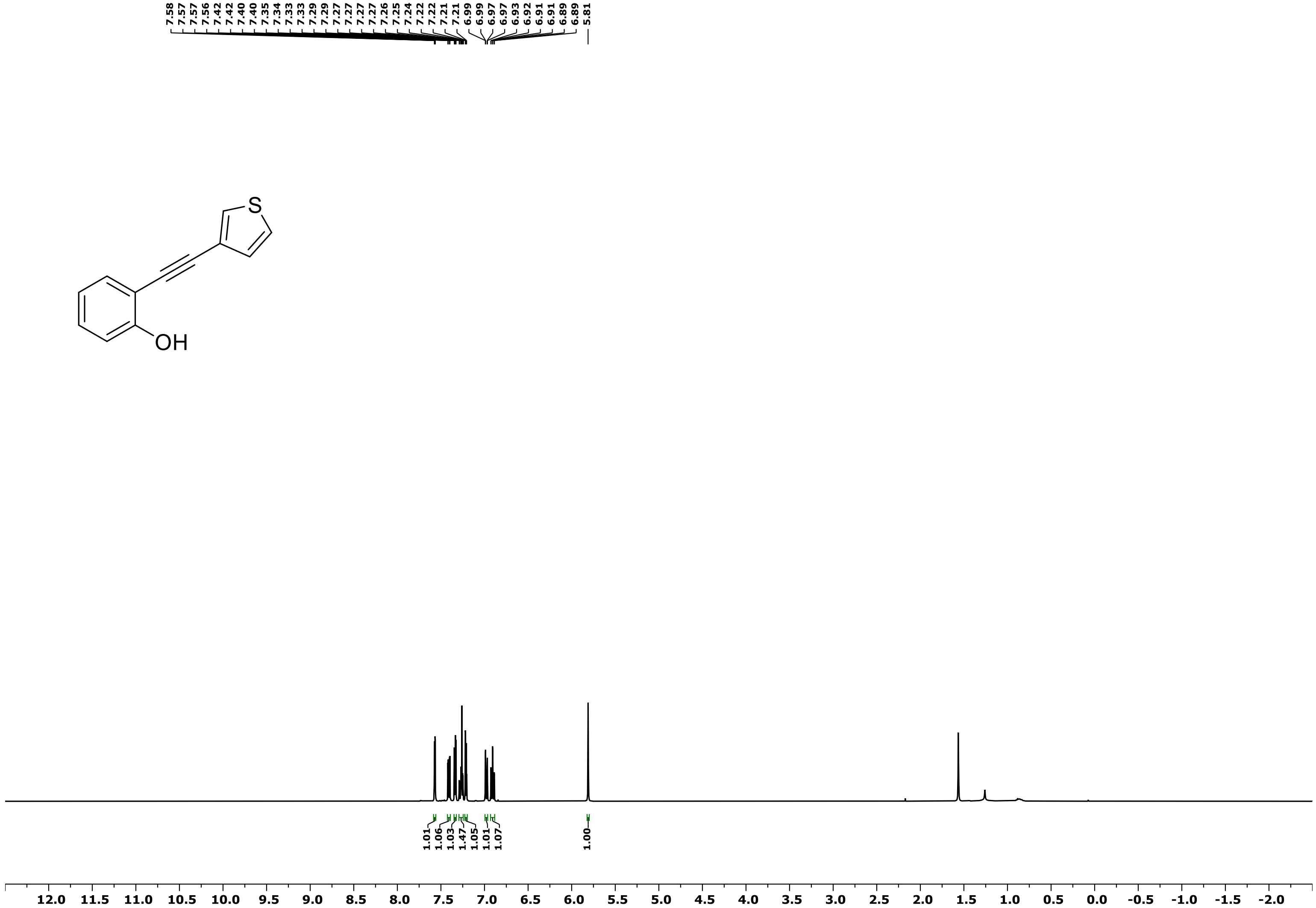

Figure S41: ^{13}C NMR (101 MHz, $CDCl_3$, 298 K) spectrum of *2-(thiophen-3-ylethynyl)phenol*, **1o**.

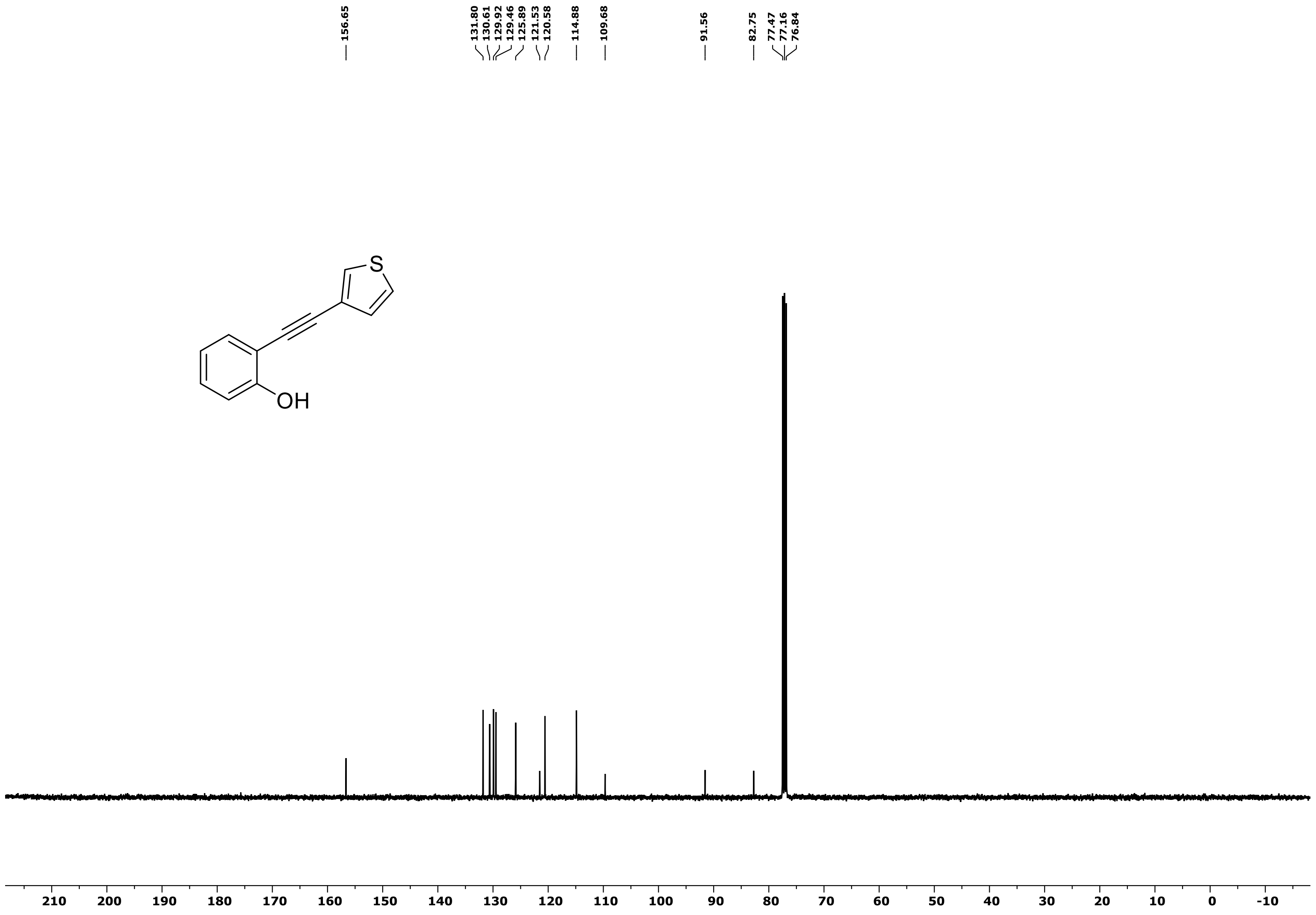

Figure S42: ^{1}H NMR (400 MHz, $CDCl_3$, 298 K) spectrum of *2-(cyclopropylethynyl)phenol*, **1p**.

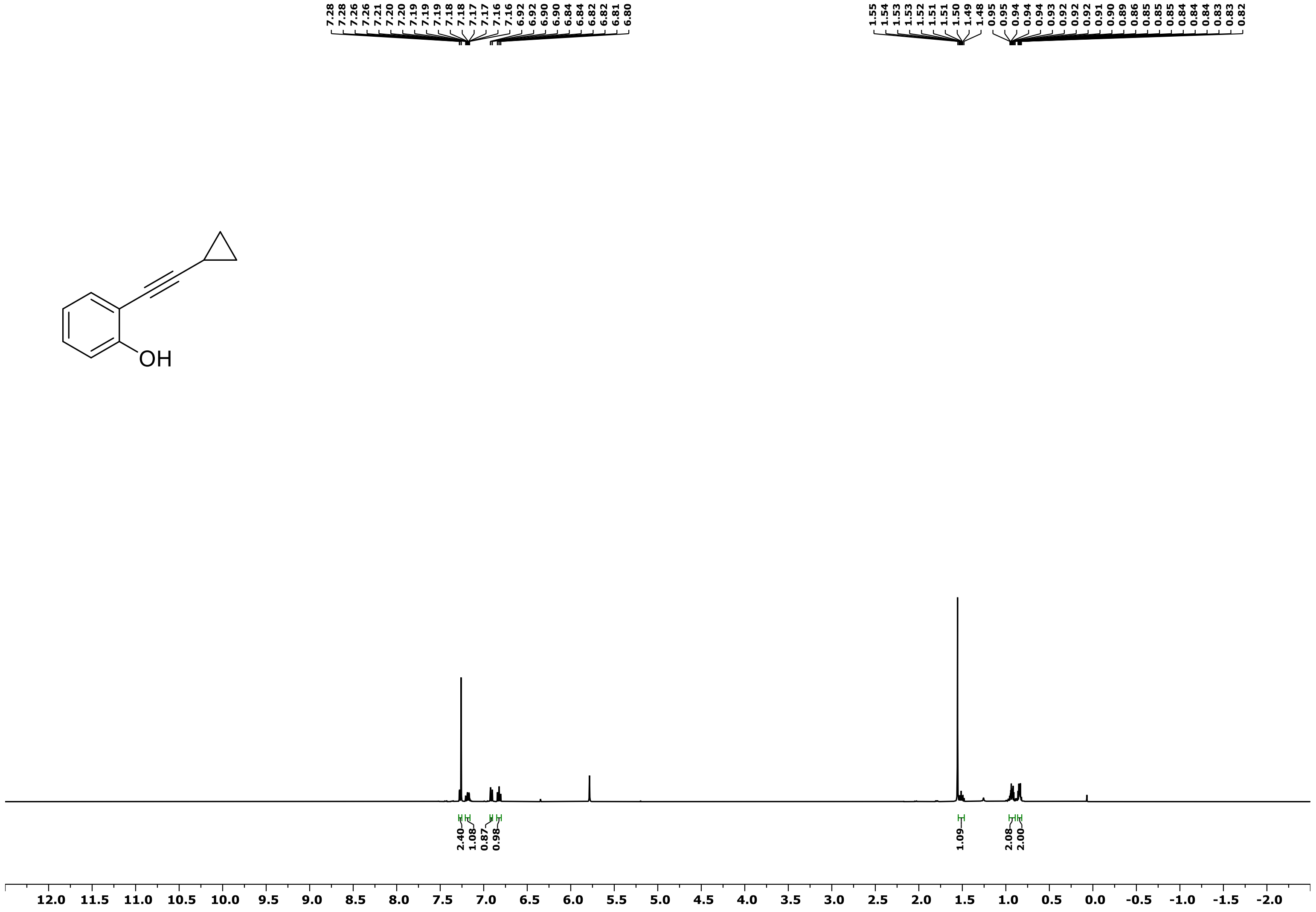

Figure S43: ^{13}C NMR (101 MHz, $CDCl_3$, 298 K) spectrum of *2-(cyclopropylethynyl)phenol*, **1p**.

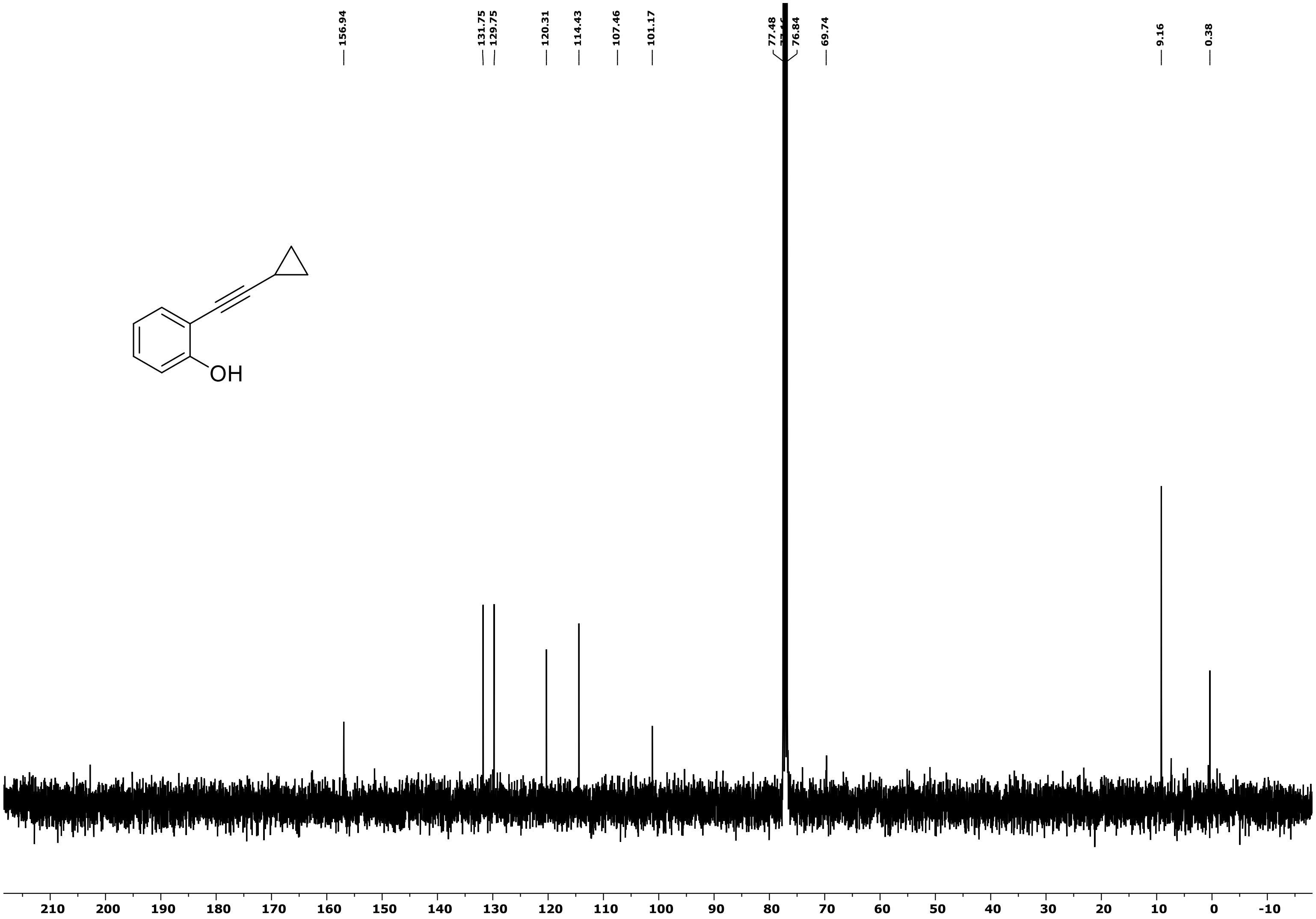

Figure S44: ^{1}H NMR (400 MHz, $CDCl_3$, 298 K) spectrum of *2-((2-(phenylethynyl)phenyl)ethynyl)phenol*, **1q**.

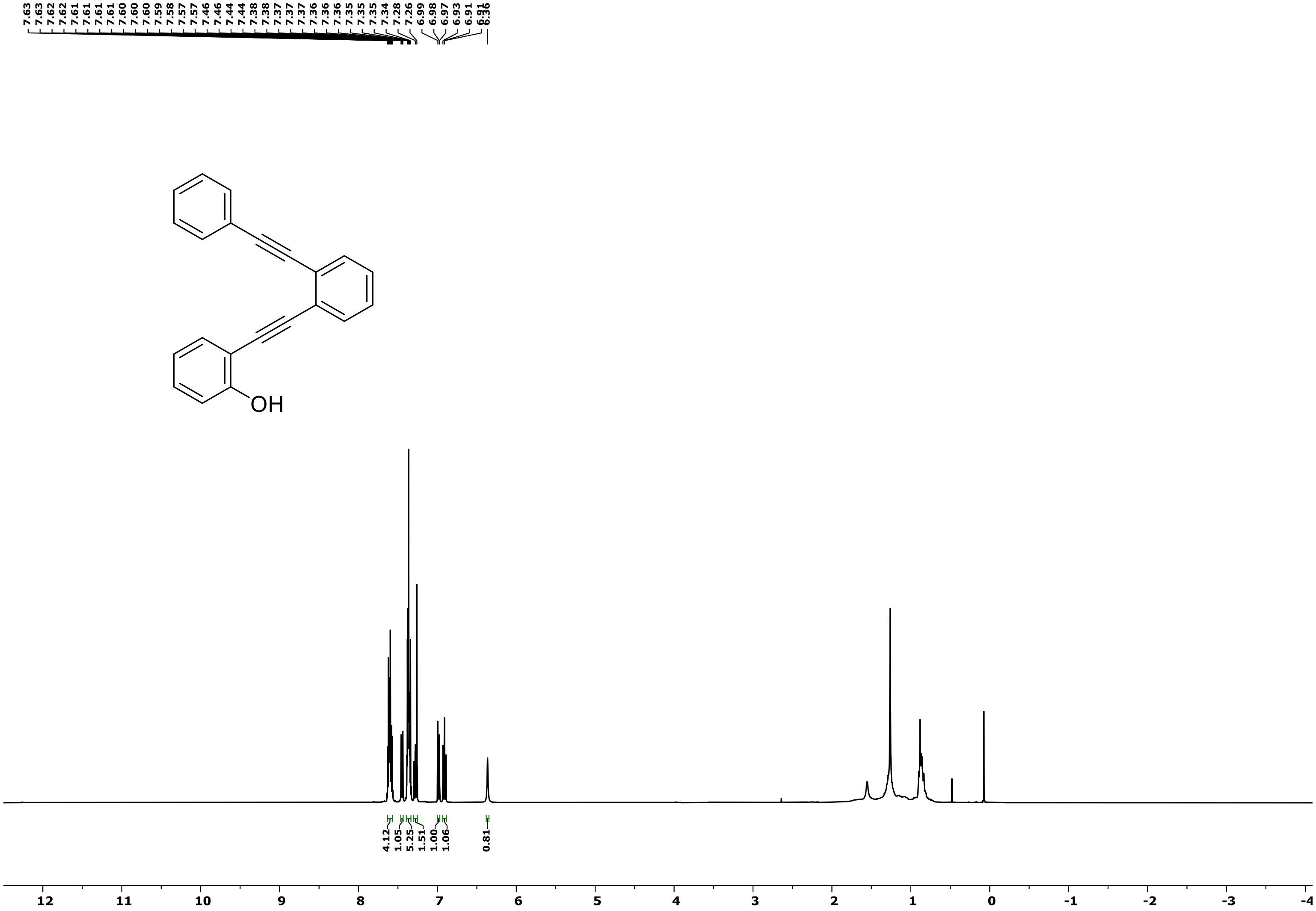

Figure S45: ^{13}C NMR (101 MHz, $CDCl_3$, 298 K) spectrum of *2-((2-(phenylethynyl)phenyl)ethynyl)phenol*, **1q**.

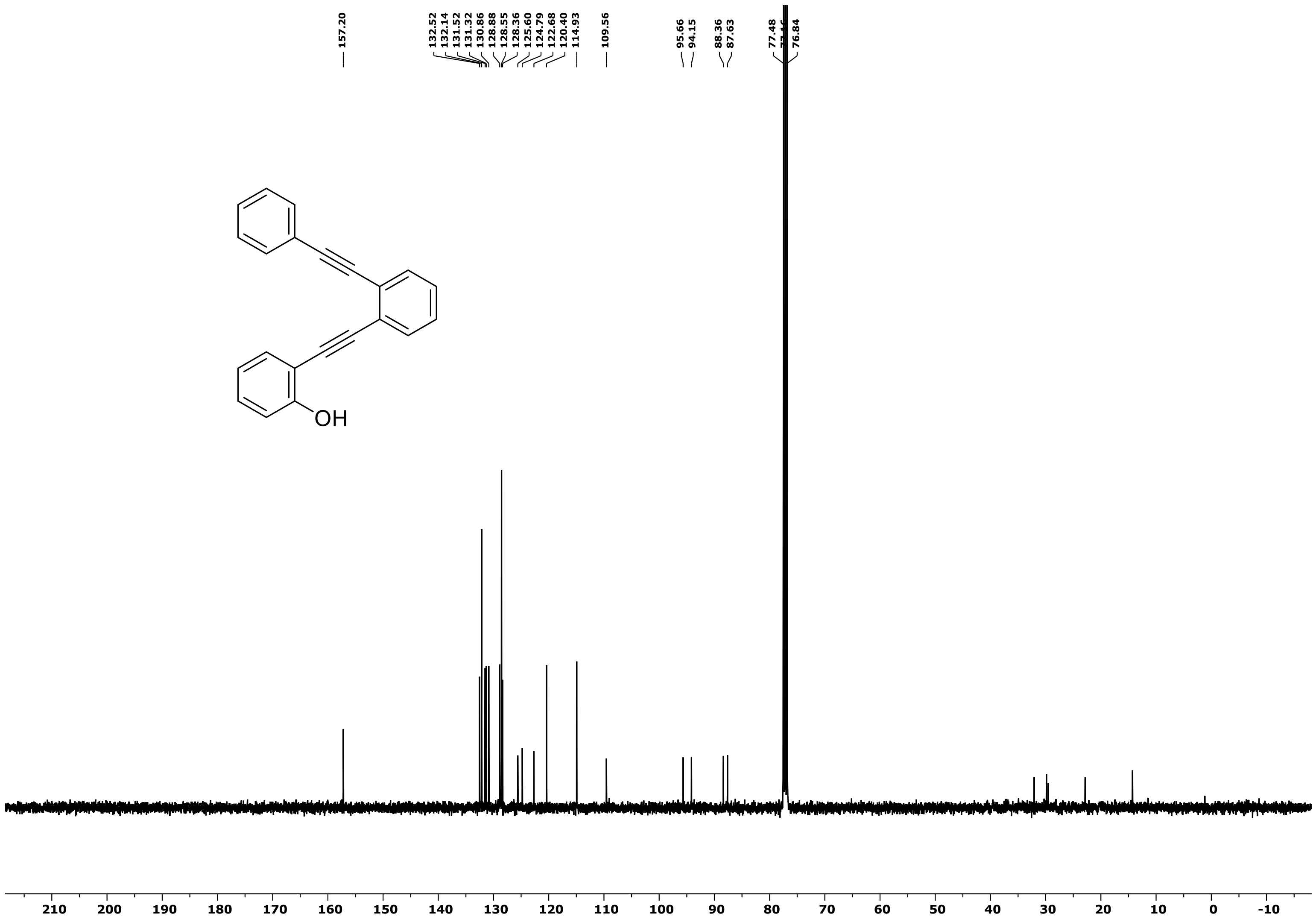

Figure S46: ^{1}H NMR (400 MHz, $CDCl_3$, 298 K) spectrum of *4-methyl-N-(2-((2-(phenylethynyl)phenyl)ethynyl)phenyl)benzenesulfonamide*, **1r**.

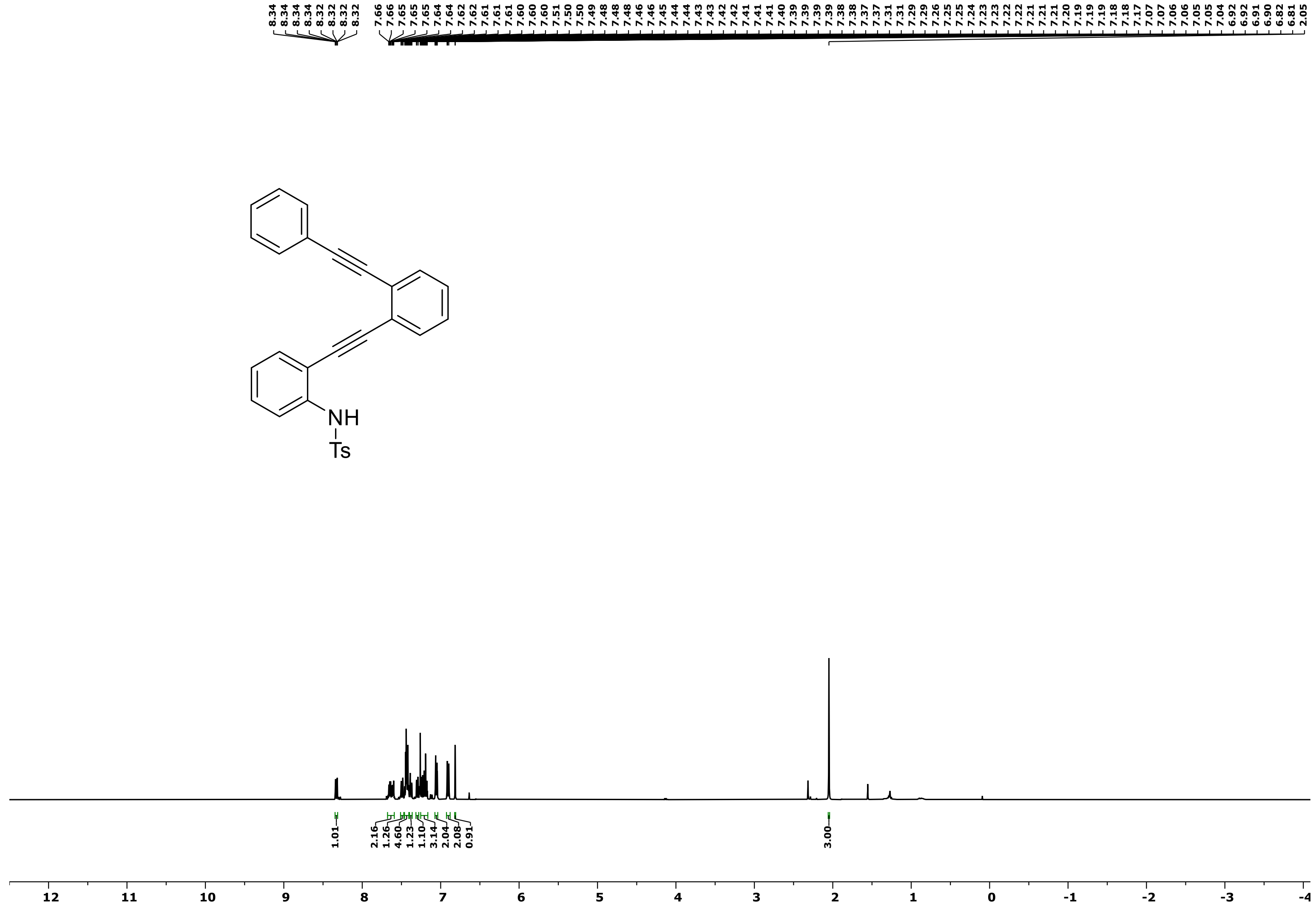

Figure S47: ^{13}C NMR (101 MHz, $CDCl_3$, 298 K) spectrum of *4-methyl-N-(2-((2-(phenylethynyl)phenyl)ethynyl)phenyl)benzenesulfonamide*, **1r**.

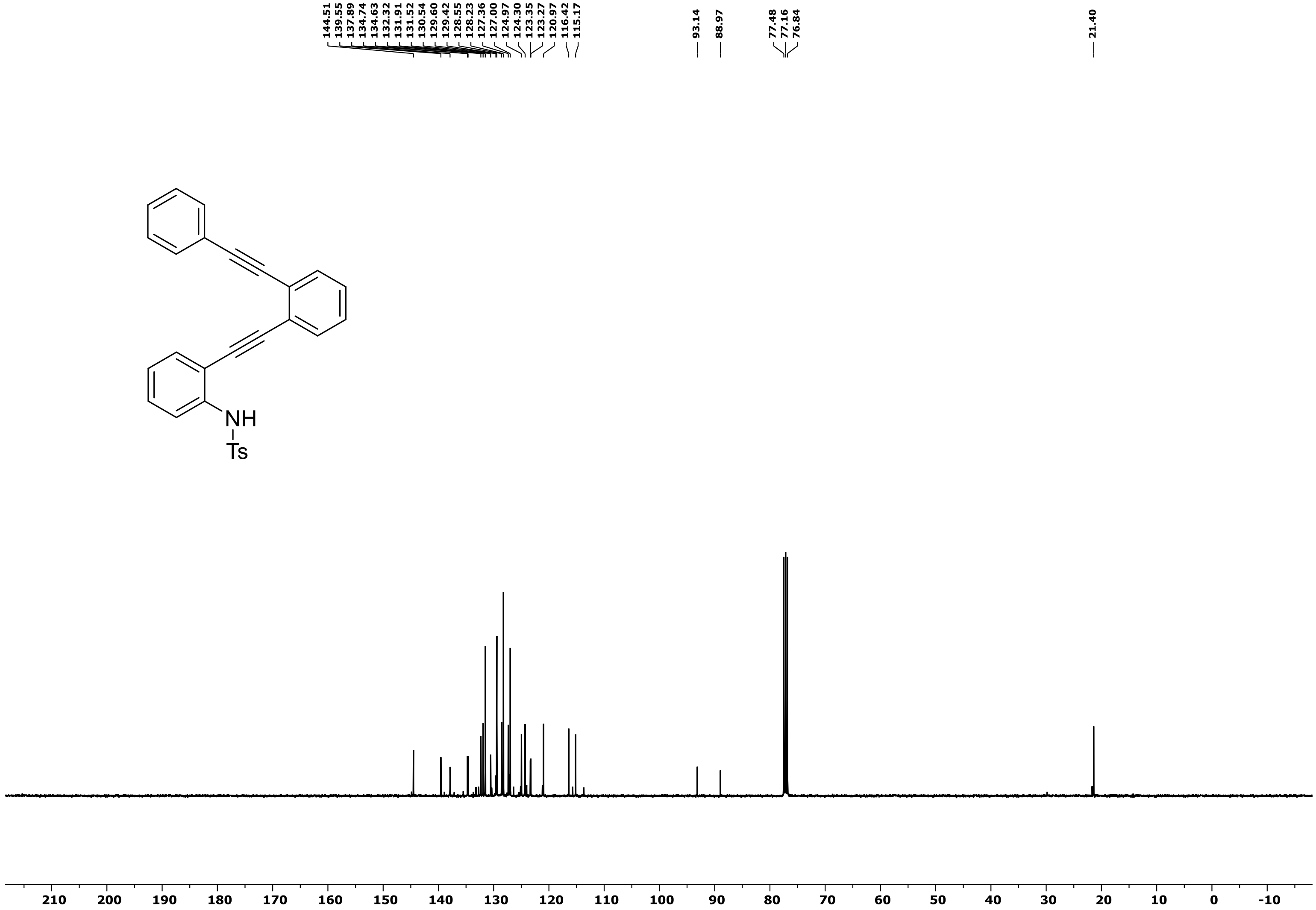

Figure S48: ^{1}H NMR (400 MHz, $CDCl_3$, 298 K) spectrum of *(3-phenoxyprop-1-yn-1-yl)benzene*, **4a**.

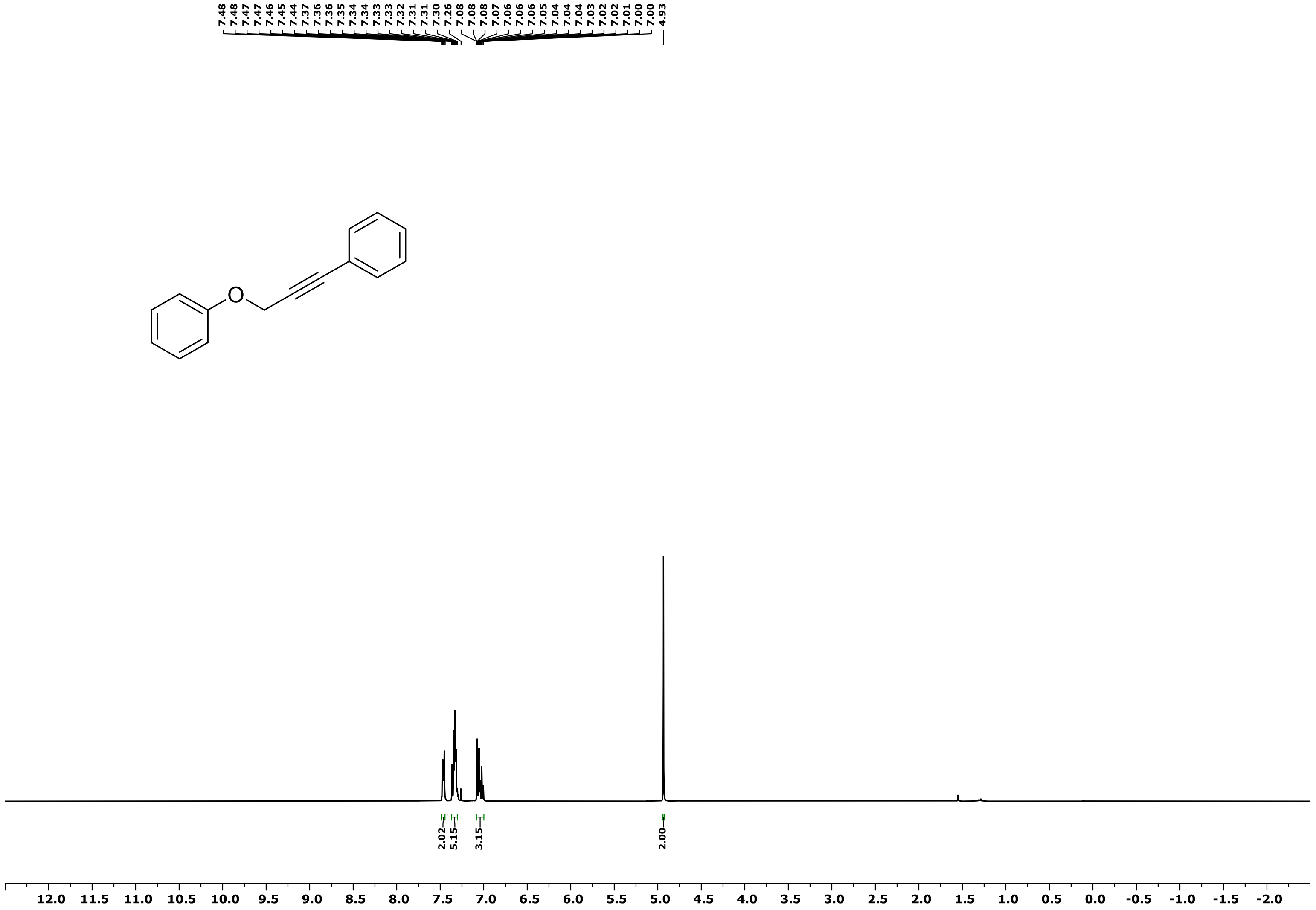

Figure S49: ^{13}C NMR (101 MHz, $CDCl_3$, 298 K) spectrum of *(3-phenoxyprop-1-yn-1-yl)benzene*, **4a**.

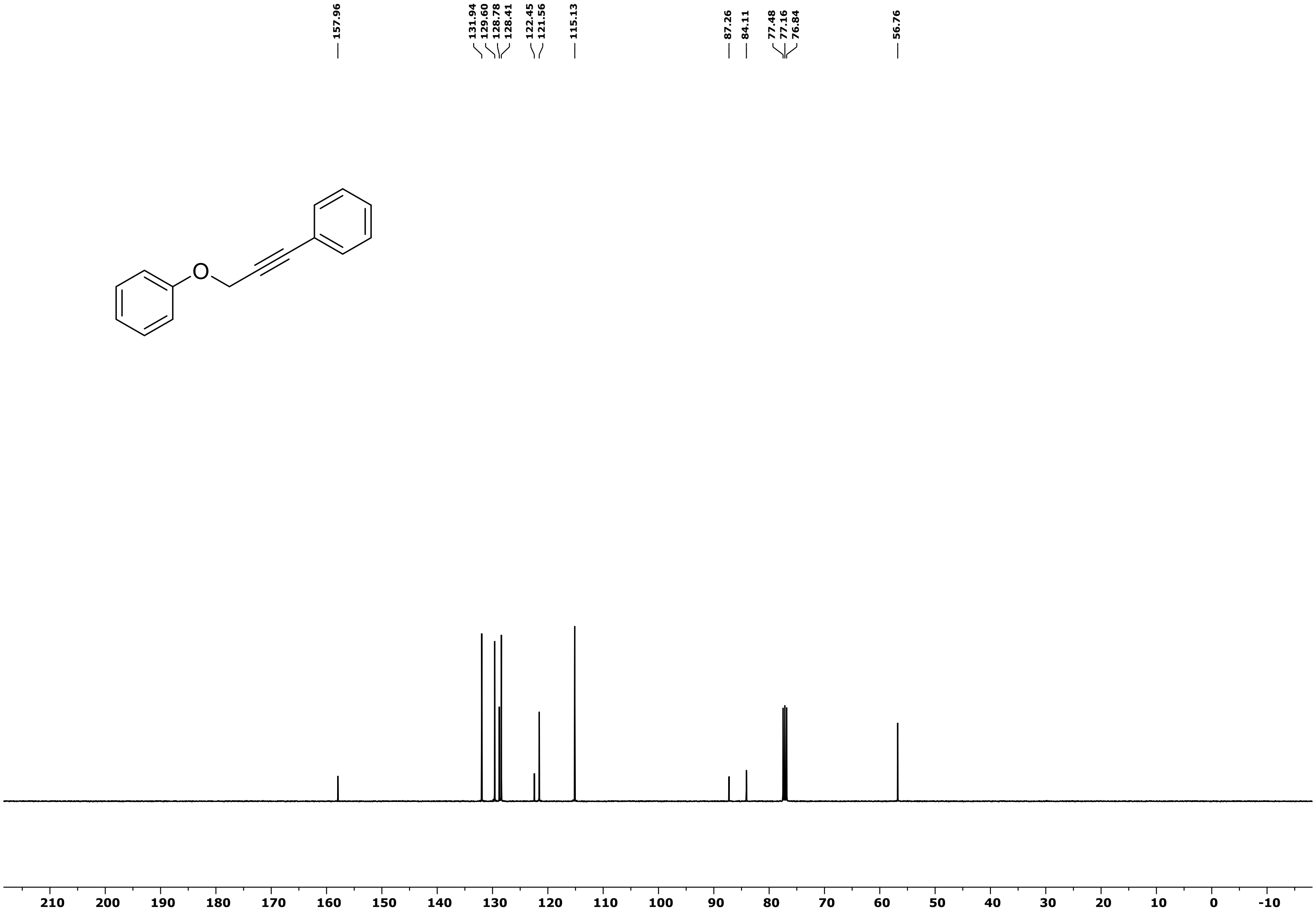

Figure S50: ^{1}H NMR (400 MHz, $CDCl_3$, 298 K) spectrum of *1-methyl-4-((3-phenylprop-2-yn-1-yl)oxy)benzene*, **4b**.

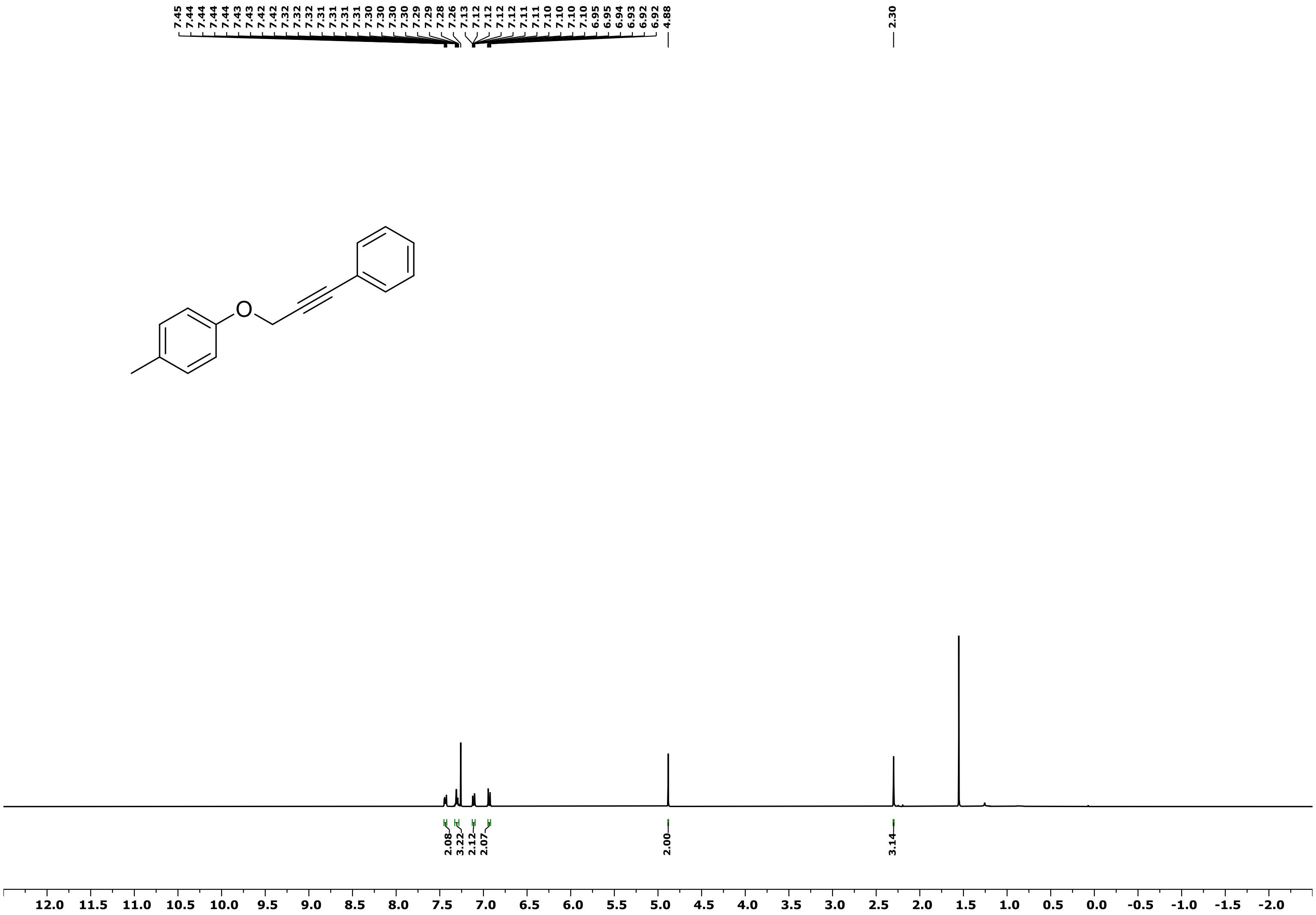

Figure S51: ^{13}C NMR (101 MHz, $CDCl_3$, 298 K) spectrum of *1-methyl-4-((3-phenylprop-2-yn-1-yl)oxy)benzene*, **4b**.

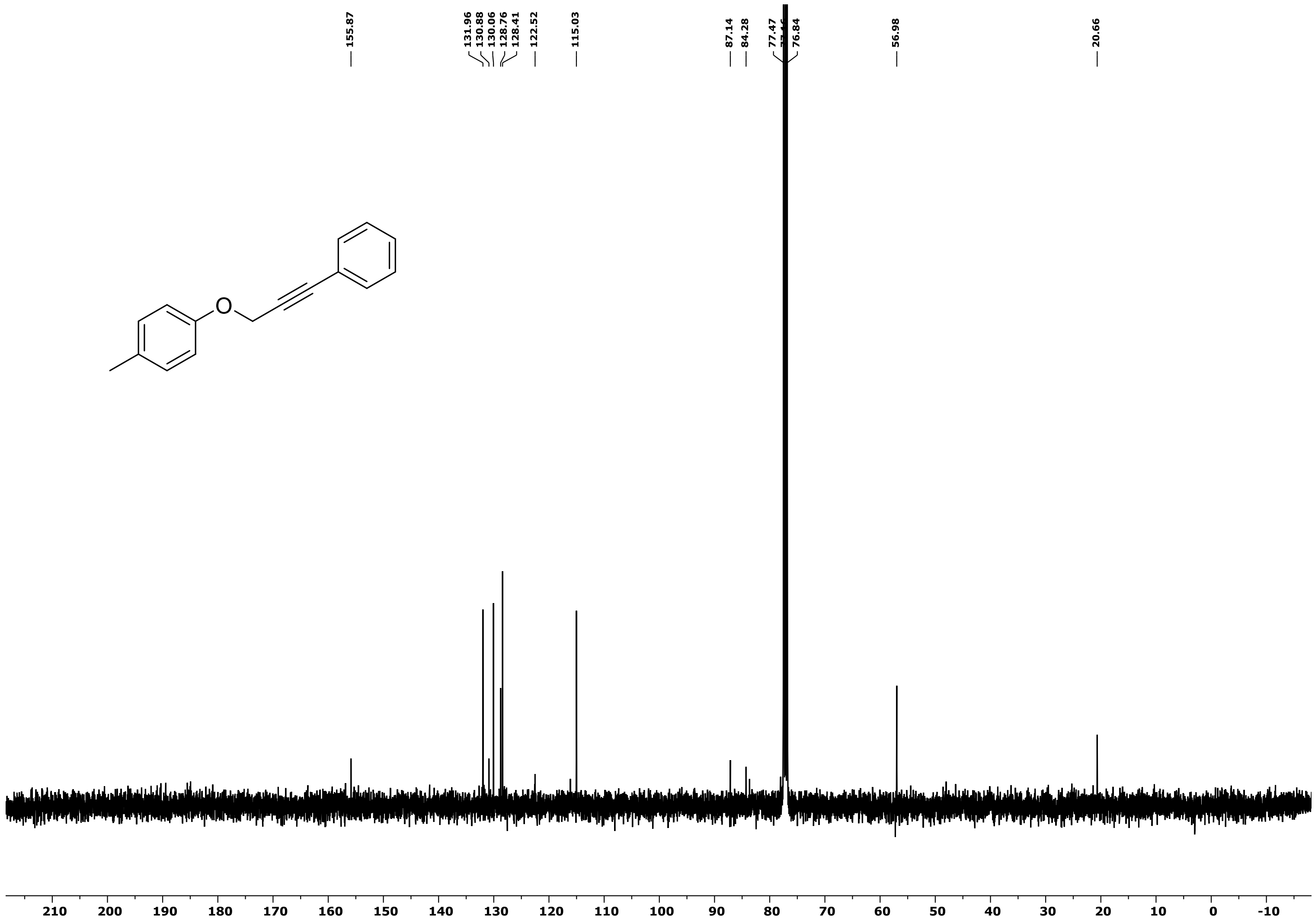

Figure S52: ^{1}H NMR (400 MHz, $CDCl_3$, 298 K) spectrum of *1-methoxy-4-(3-phenoxyprop-1-yn-1-yl)benzene*, **4c**.

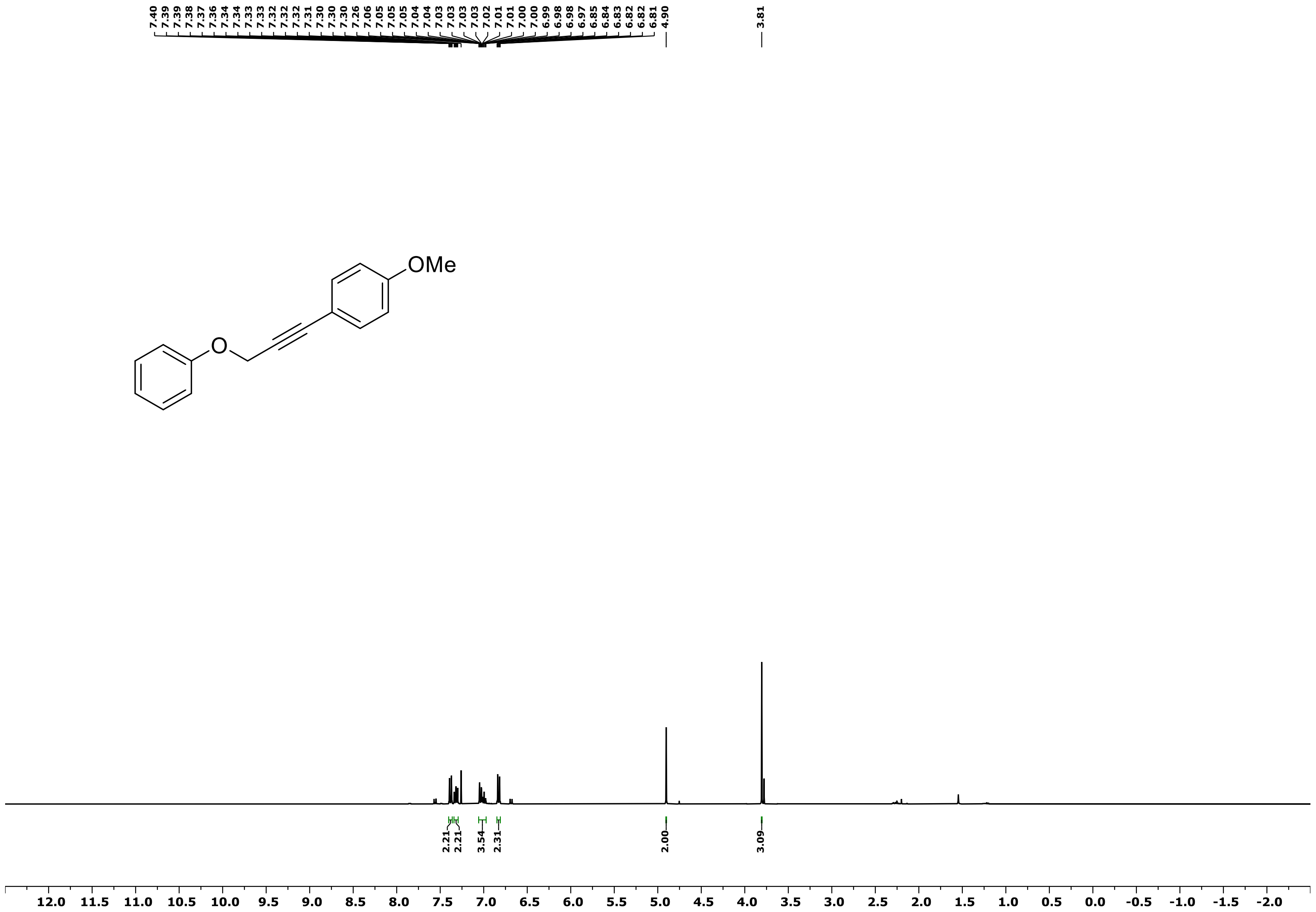

Figure S53: ^{13}C NMR (101 MHz, $CDCl_3$, 298 K) spectrum of *1-methoxy-4-(3-phenoxyprop-1-yn-1-yl)benzene*, **4c**.

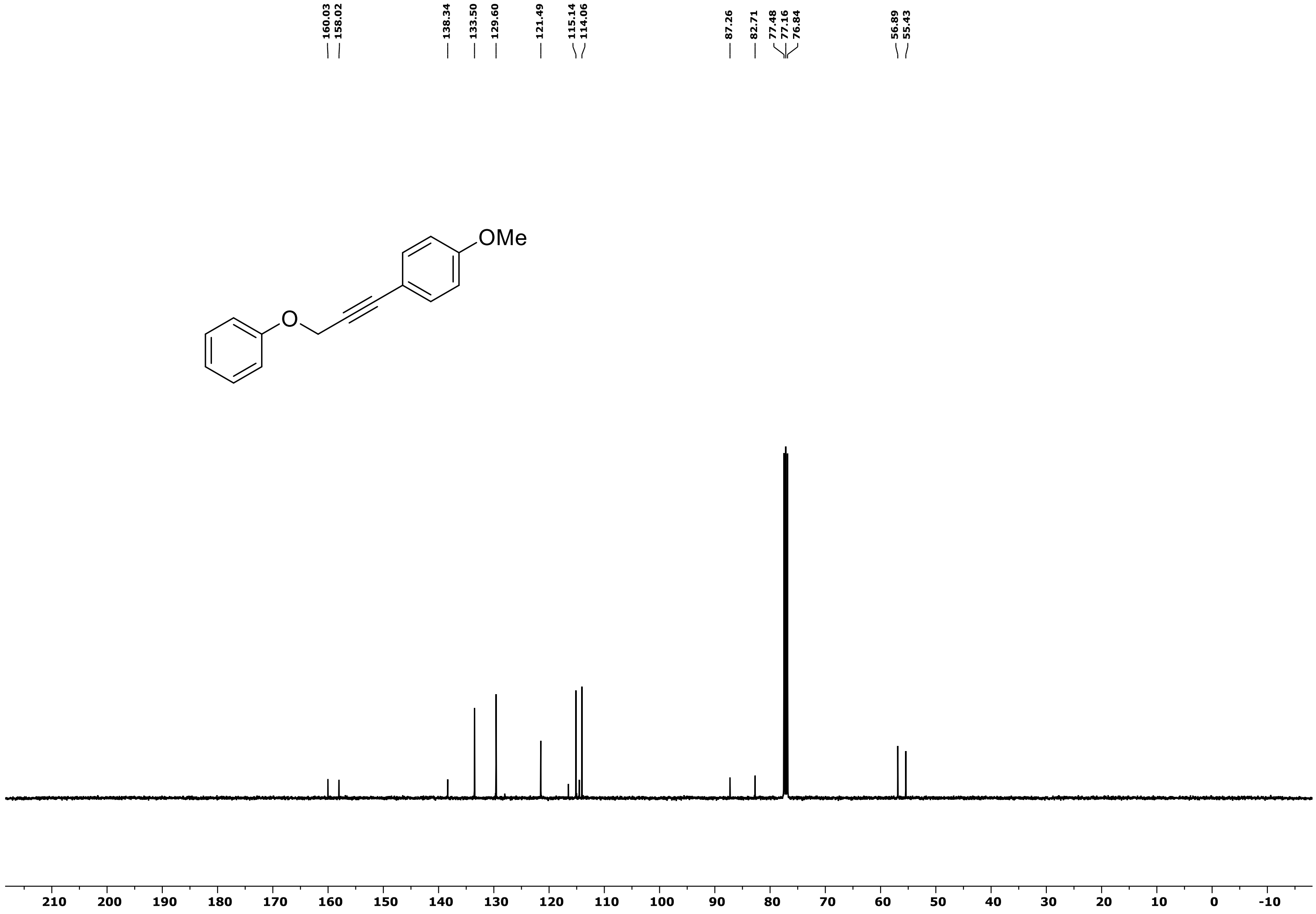

Figure S54: ^{1}H NMR (400 MHz, $CDCl_3$, 298 K) spectrum of *5-(3-phenoxyprop-1-yn-1-yl)benzo[d][1,3]dioxole*, **4d**.

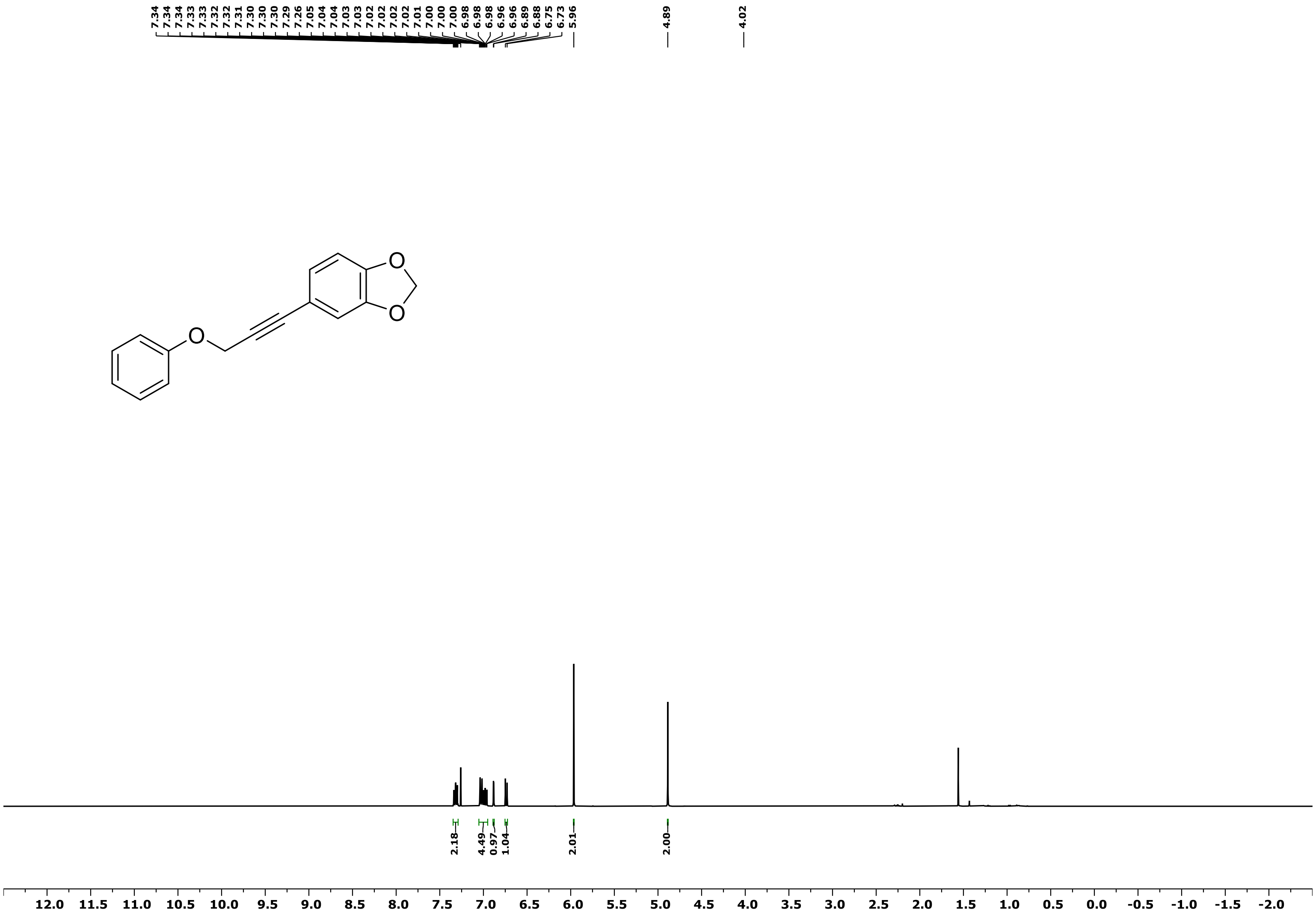

Figure S55: ^{13}C NMR (101 MHz, $CDCl_3$, 298 K) spectrum of *5-(3-phenoxyprop-1-yn-1-yl)benzo[d][1,3]dioxole*, **4d**.

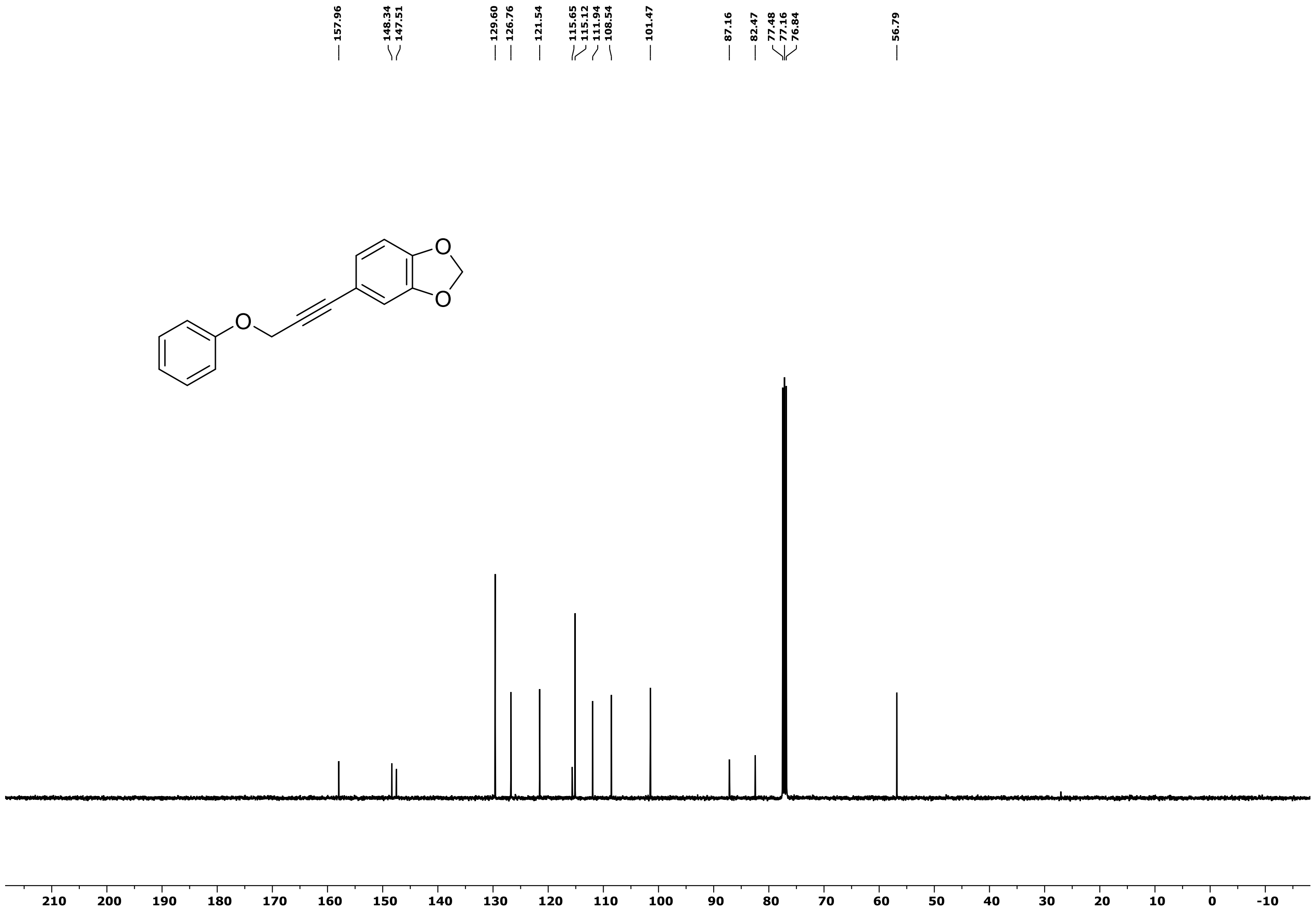

Figure S56: ^{1}H NMR (400 MHz, $CDCl_3$, 298 K) spectrum of *1-((3-phenylprop-2-yn-1-yl)oxy)-3-(trifluoromethyl)benzene*, **4e**.

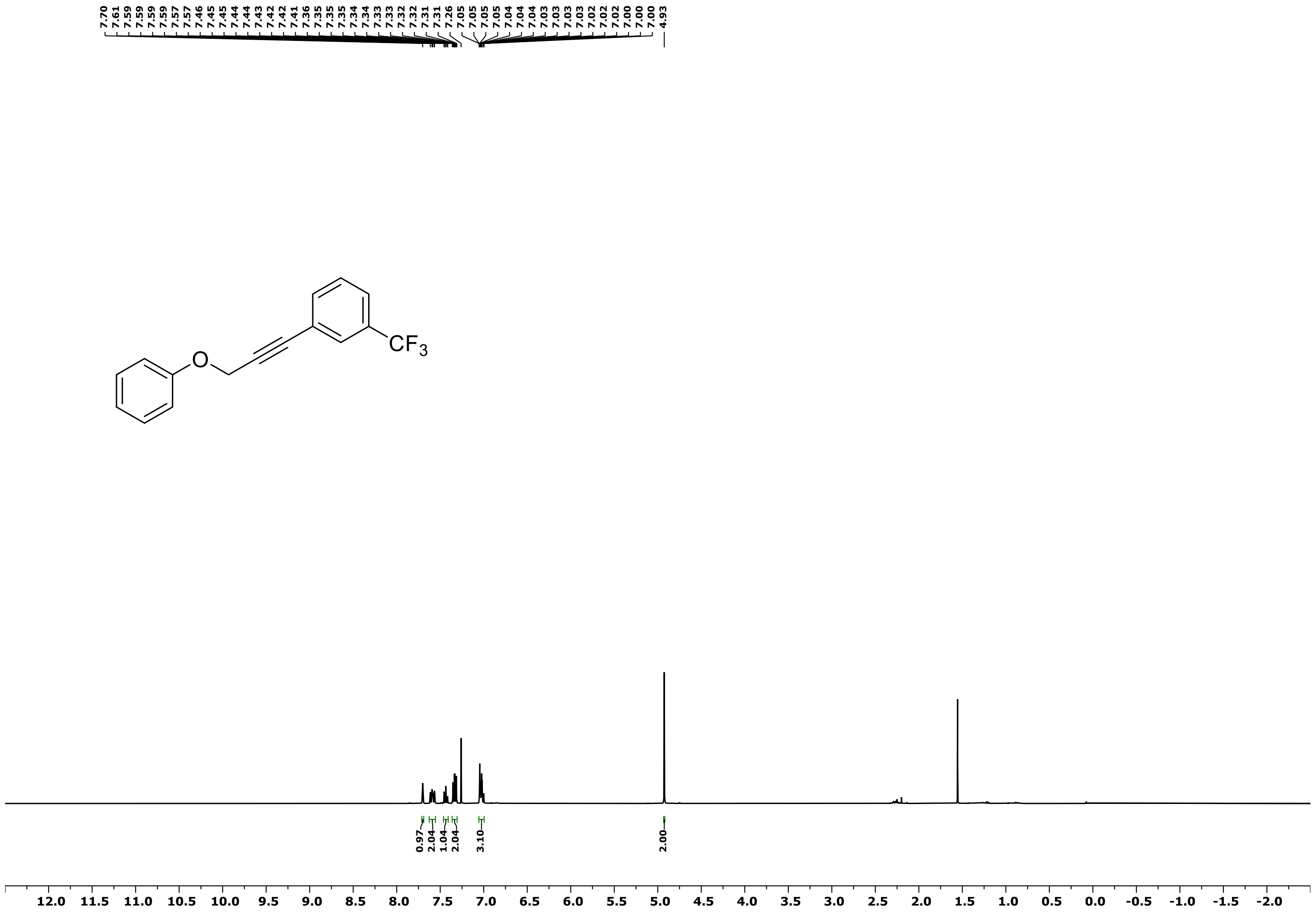

Figure S57: ^{13}C NMR (101 MHz, $CDCl_3$, 298 K) spectrum of *1-((3-phenylprop-2-yn-1-yl)oxy)-3-(trifluoromethyl)benzene*, **4e**.

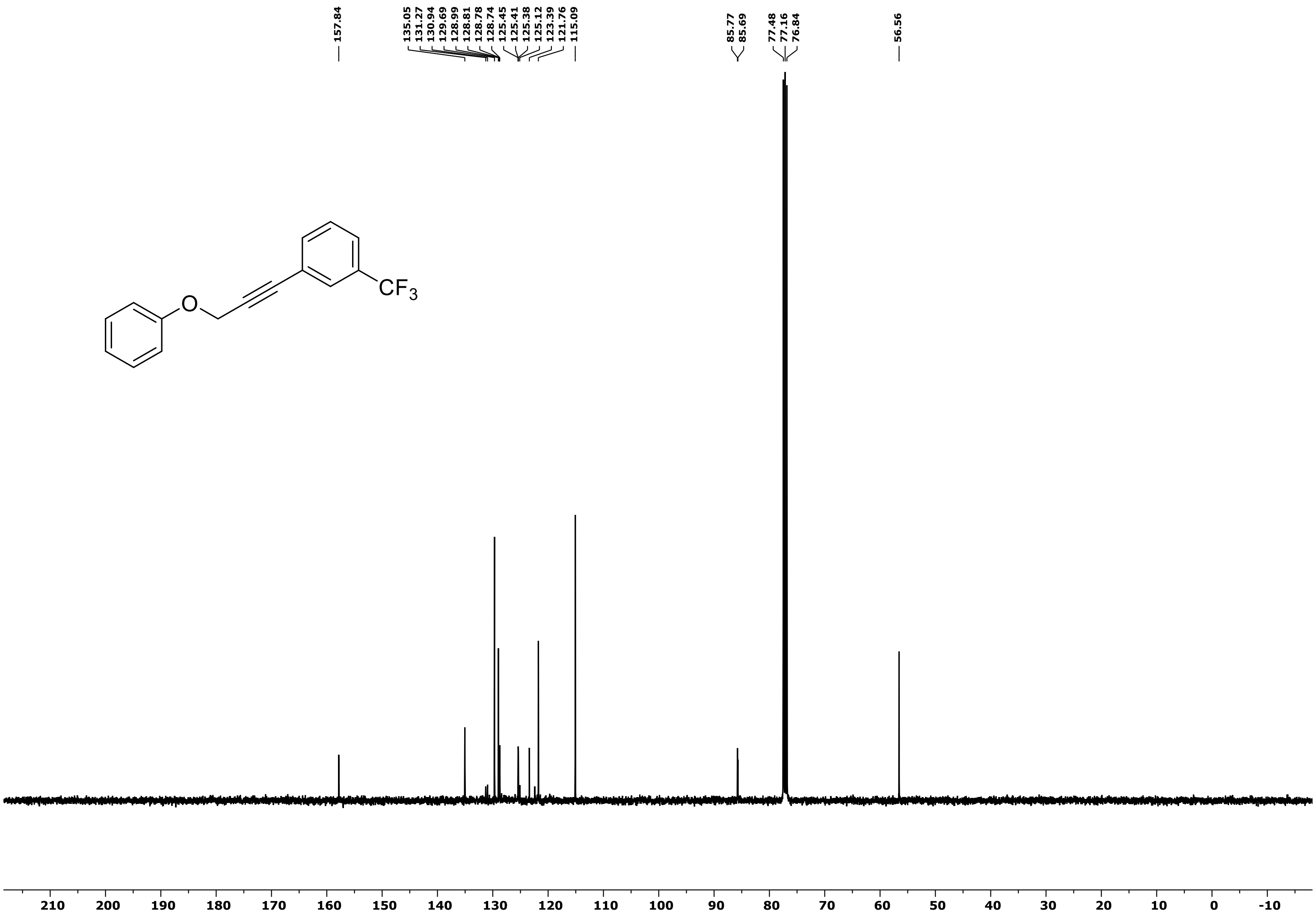

Figure S58: ^{19}F NMR (376 MHz, $CDCl_3$, 298 K) spectrum of *1-((3-phenylprop-2-yn-1-yl)oxy)-3-(trifluoromethyl)benzene*, **4e**.

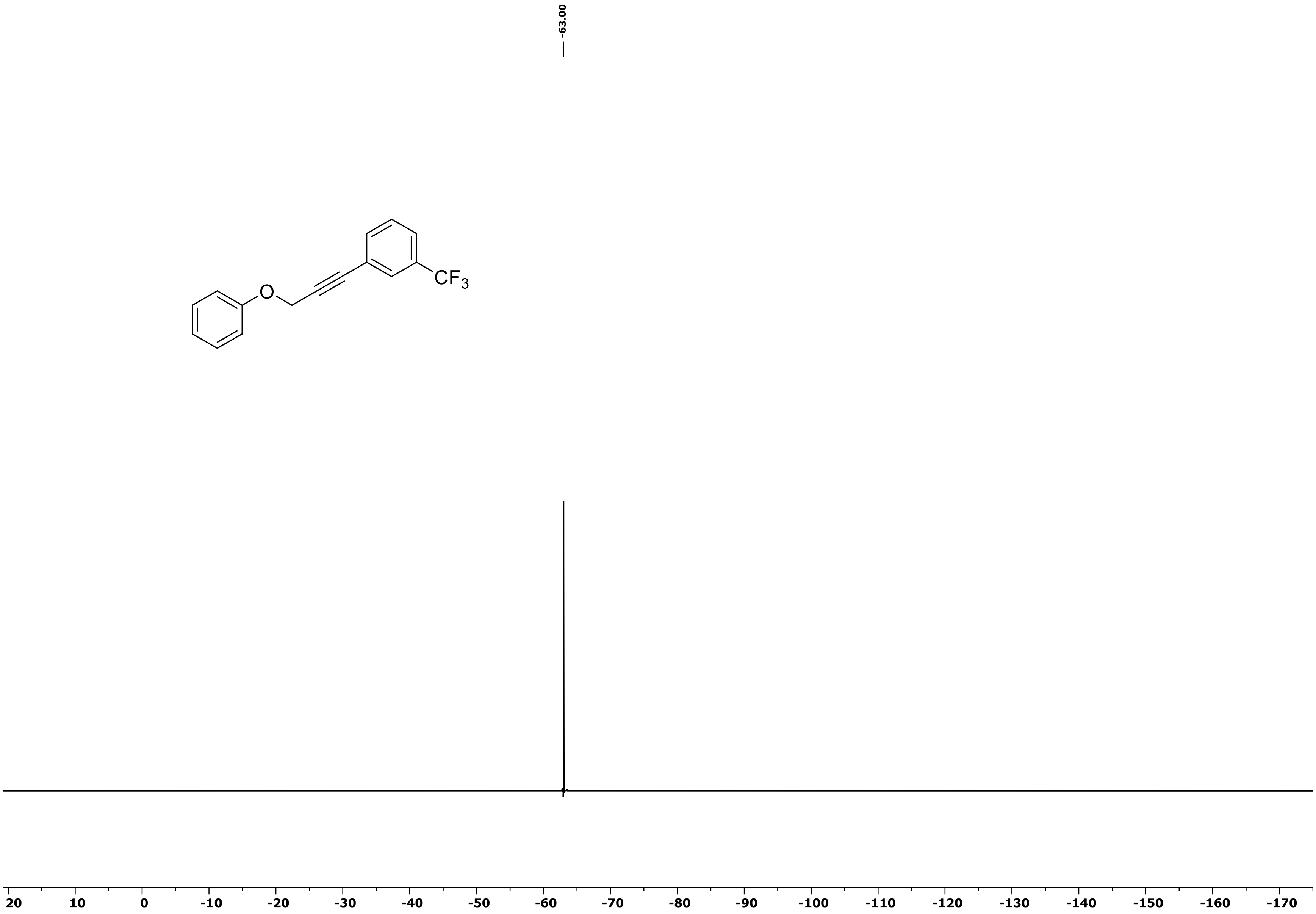

Figure S59: ^{1}H NMR (400 MHz, $CDCl_3$, 298 K) spectrum *of 1-methoxy-4-((3-phenylprop-2-yn-1-yl)oxy)benzene*, **4f**

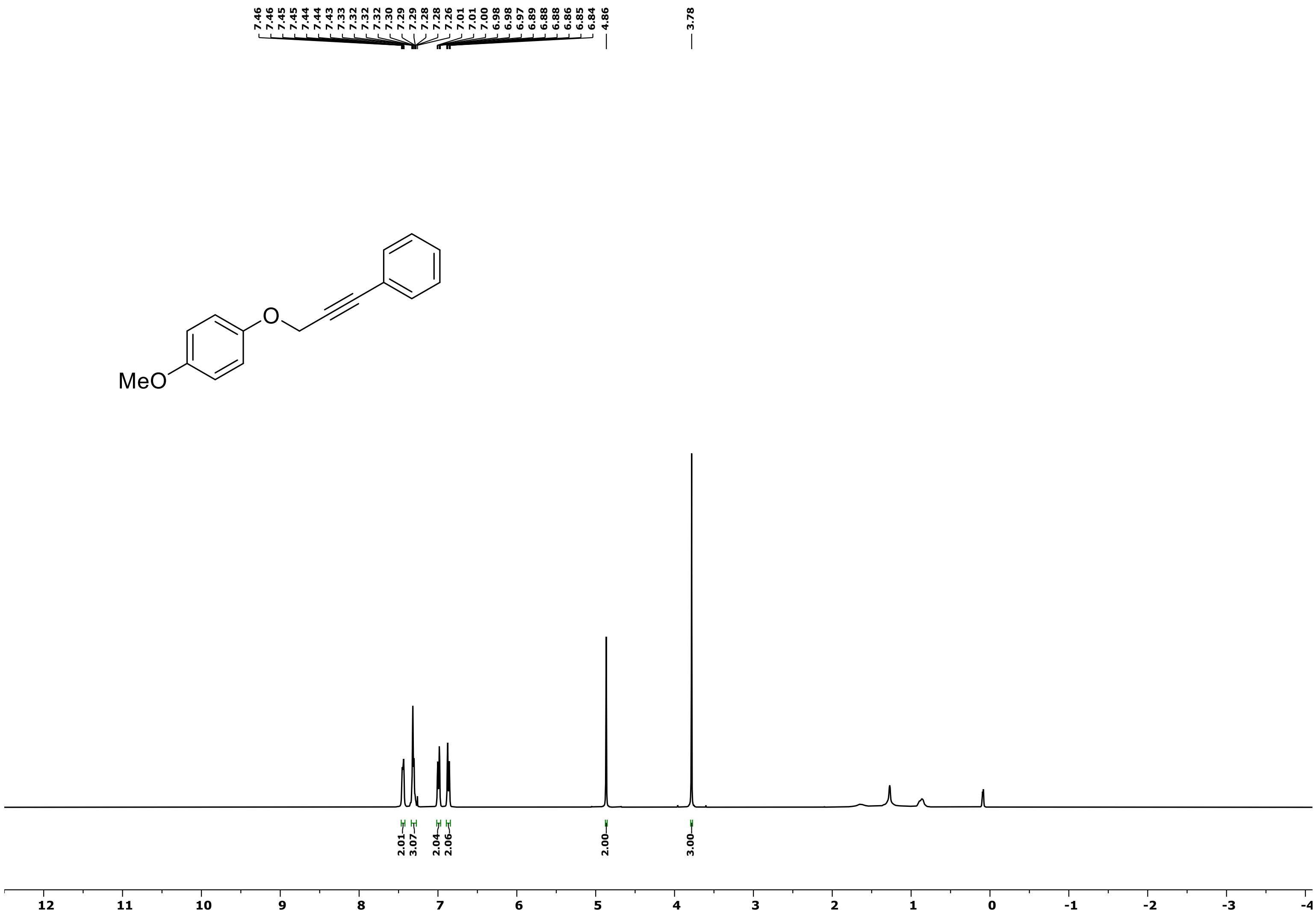

Figure S60: ^{13}C NMR (101 MHz, $CDCl_3$, 298 K) spectrum of *1-methoxy-4-((3-phenylprop-2-yn-1-yl)oxy)benzene*, **4f**

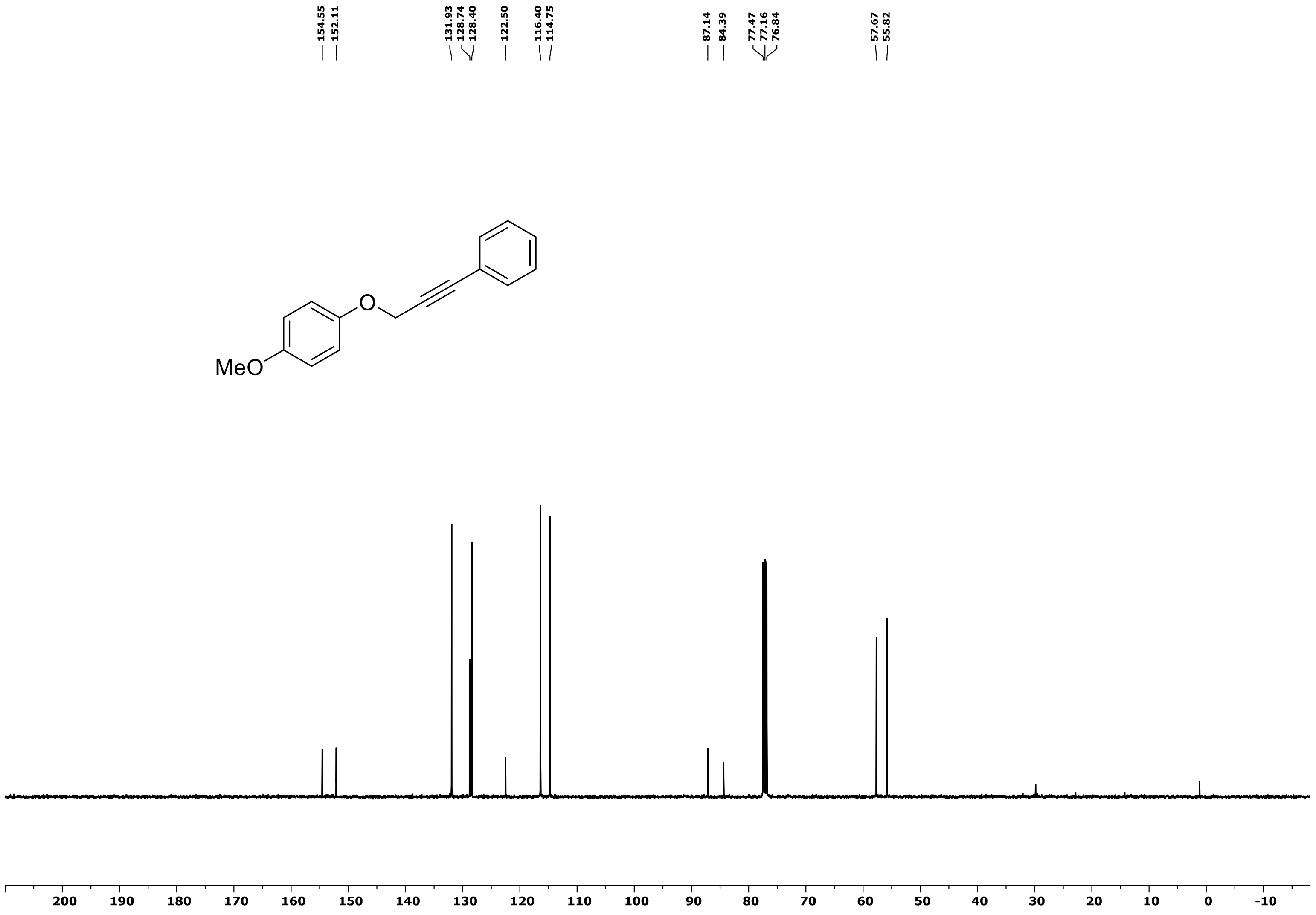

Figure S61: ^{1}H NMR (400 MHz, $CDCl_3$, 298 K) spectrum of *2-(3-phenoxyprop-1-yn-1-yl)thiophene,* **4g**

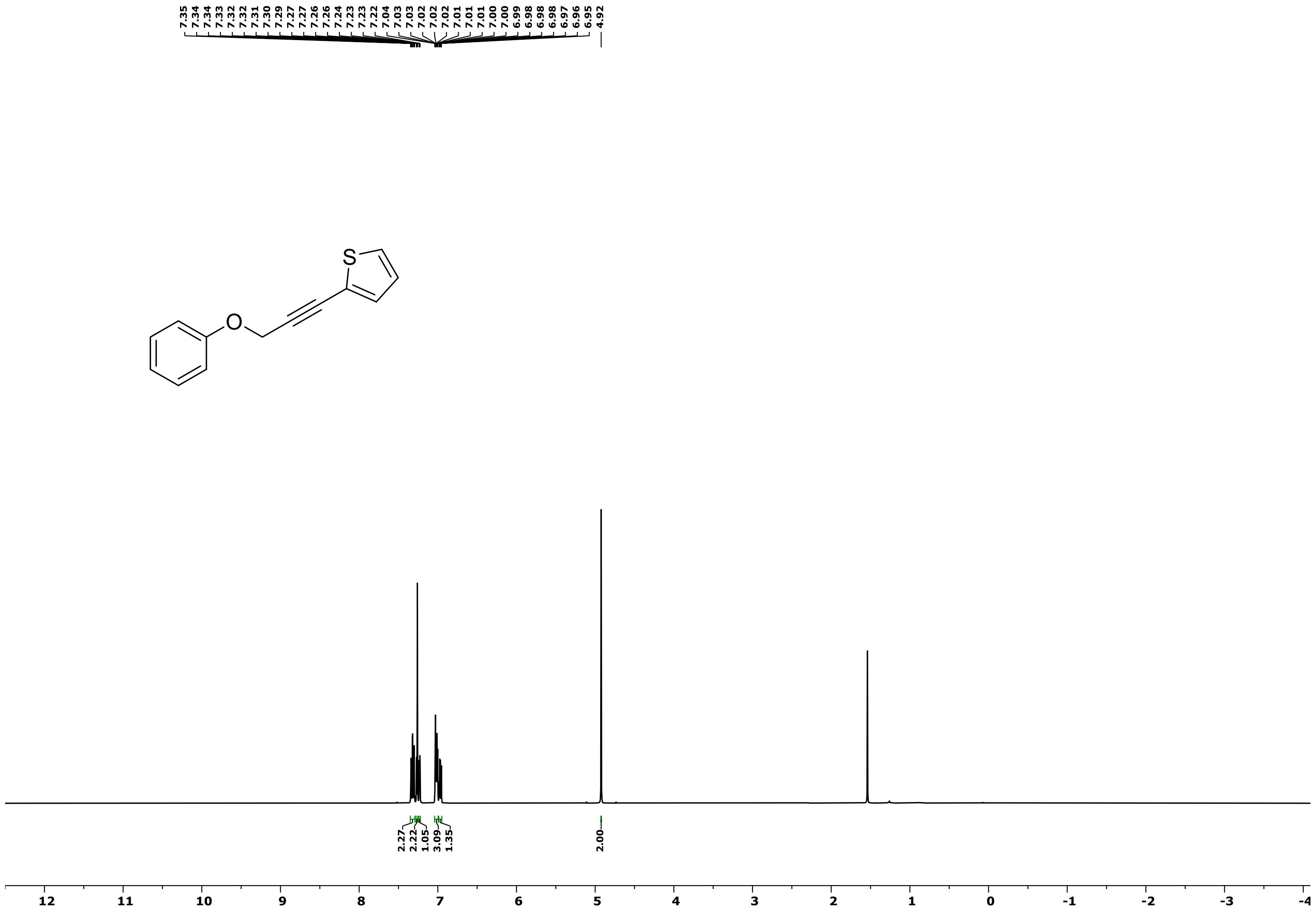

Figure S62: ^{13}C NMR (101 MHz, CDCl$_3$, 298 K) spectrum of *2-(3-phenoxyprop-1-yn-1-yl)thiophene,* **4g**

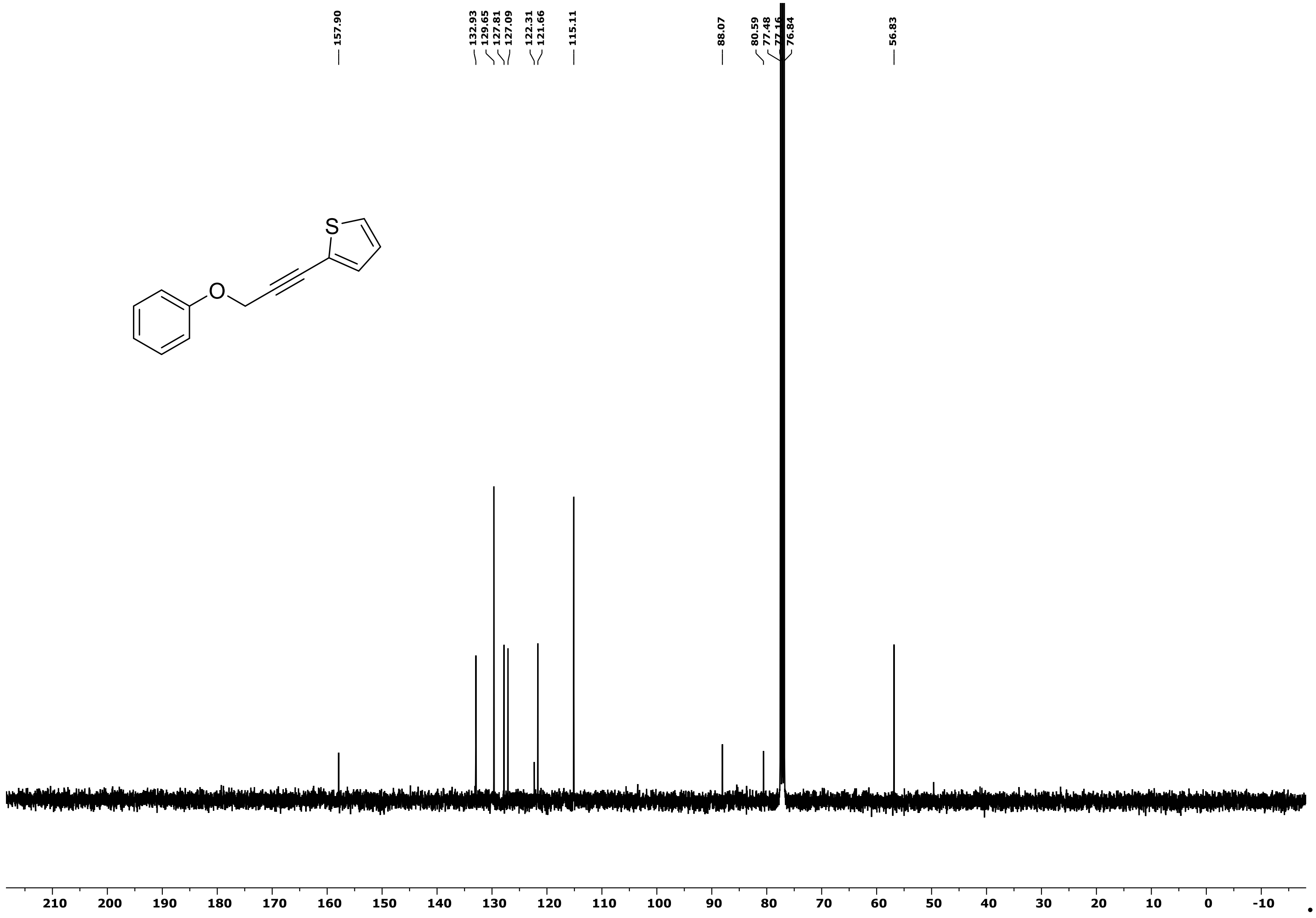

Figure S63: ^{1}H NMR (400 MHz, $CDCl_3$, 298 K) spectrum of *but-1-yne-1,4-diyldibenzene*, **4h**.

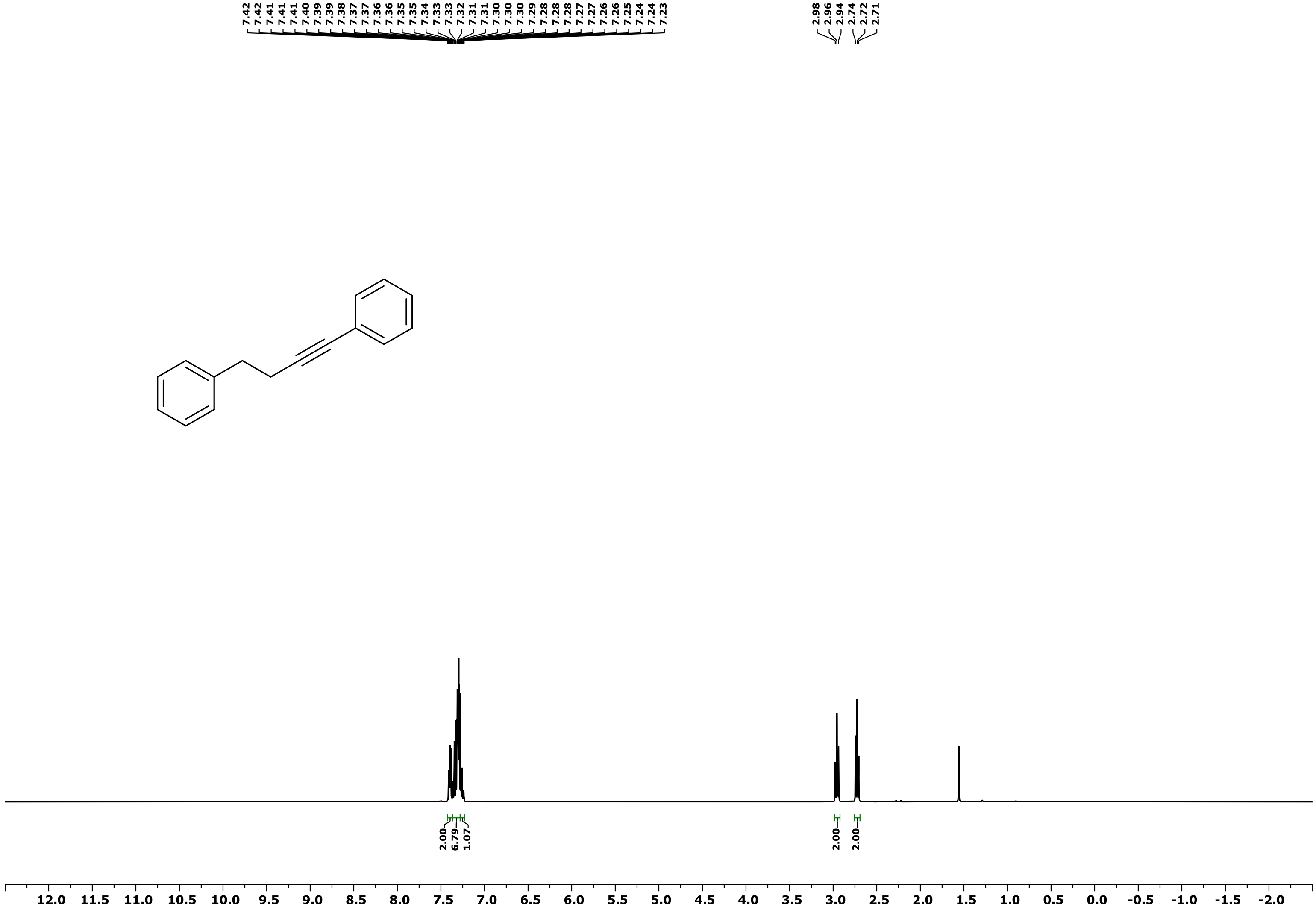

Figure S64: ^{13}C NMR (101 MHz, $CDCl_3$, 298 K) spectrum of *but-1-yne-1,4-diyldibenzene*, **4h**.

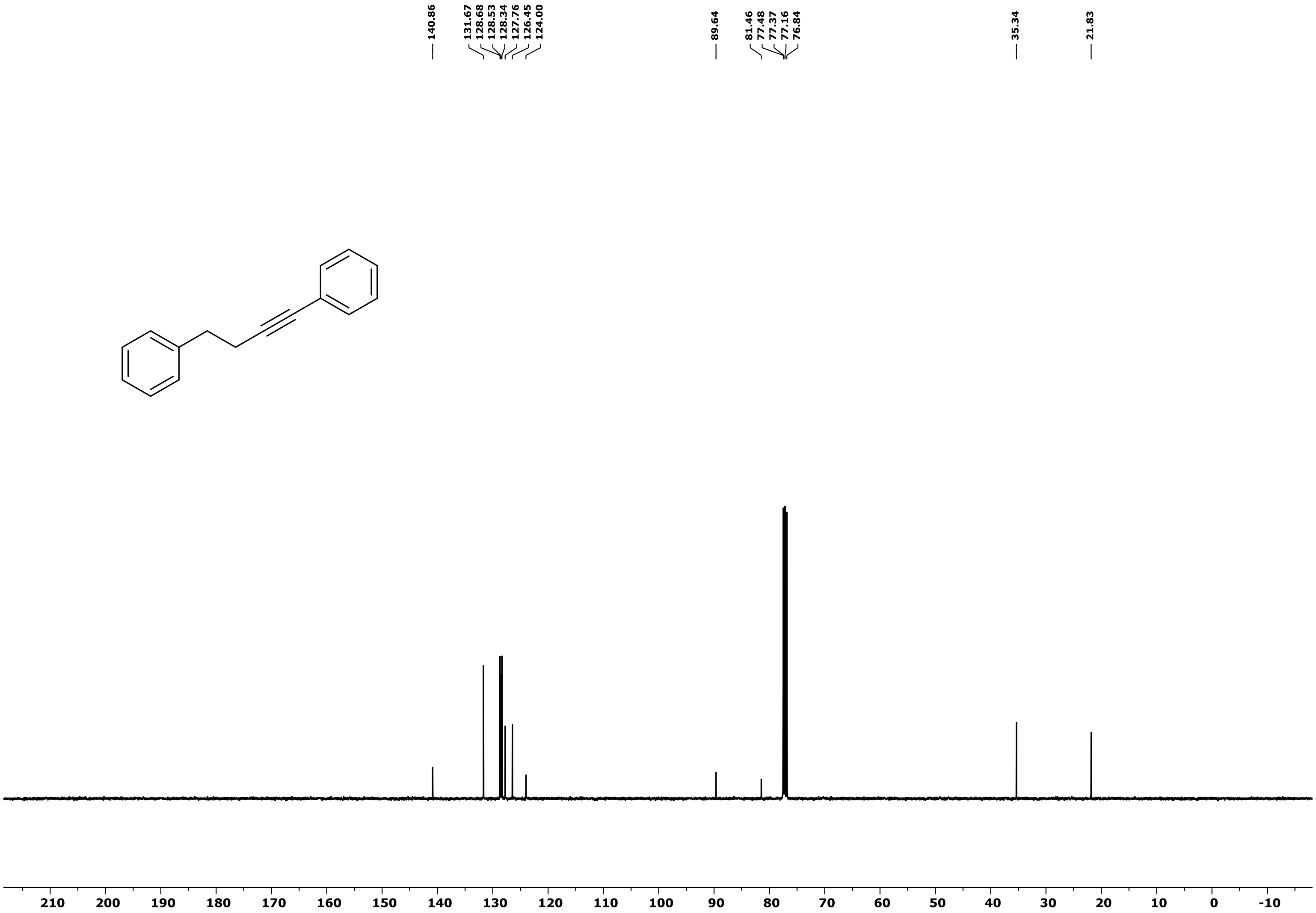

Figure S65: ^{1}H NMR (400 MHz, $CDCl_3$, 298 K) spectrum of *4-methyl-N-phenyl-N-(3-phenylprop-2-yn-1-yl)benzenesulfonamide*, **4i**.

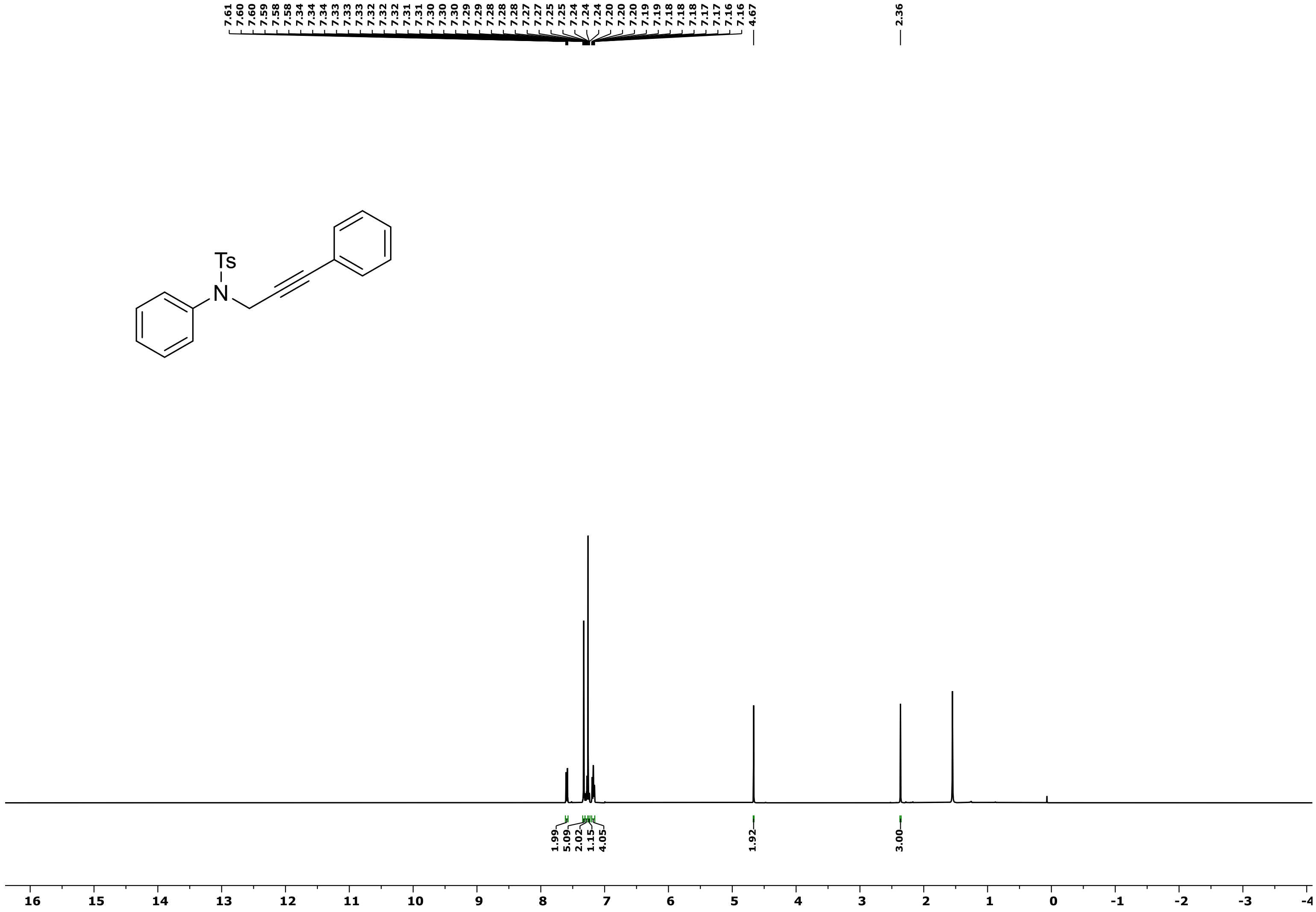

Figure S66: ^{13}C NMR (101 MHz, $CDCl_3$, 298 K) spectrum of *4-methyl-N-phenyl-N-(3-phenylprop-2-yn-1-yl)benzenesulfonamide*, **4i**.

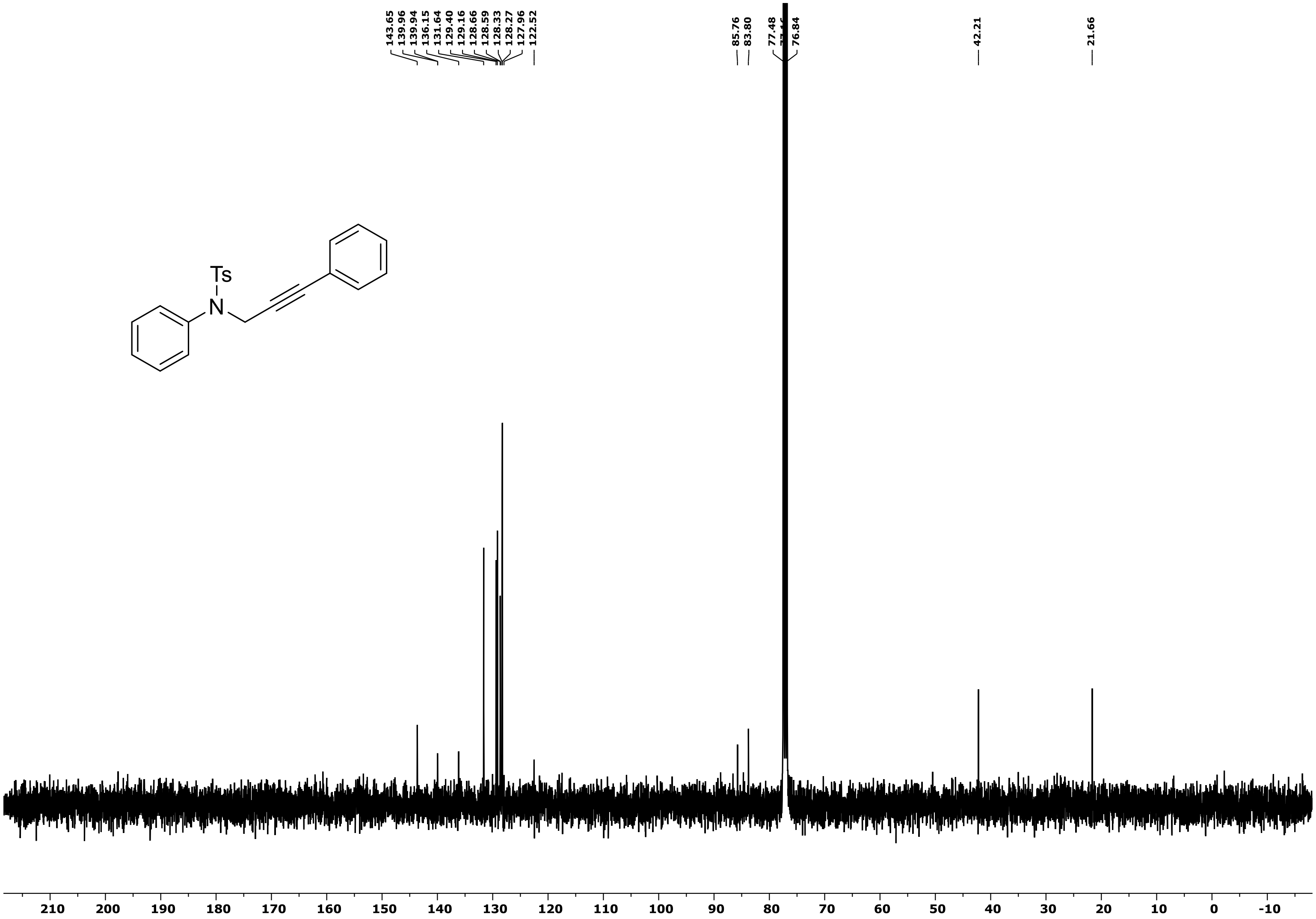

Figure S67: ^{1}H NMR (400 MHz, $CDCl_3$, 298 K) spectrum of *3-((4-(tert-butyl)phenyl)thio)-2-phenyl-1-tosyl-1H-indole*, **3aa**.

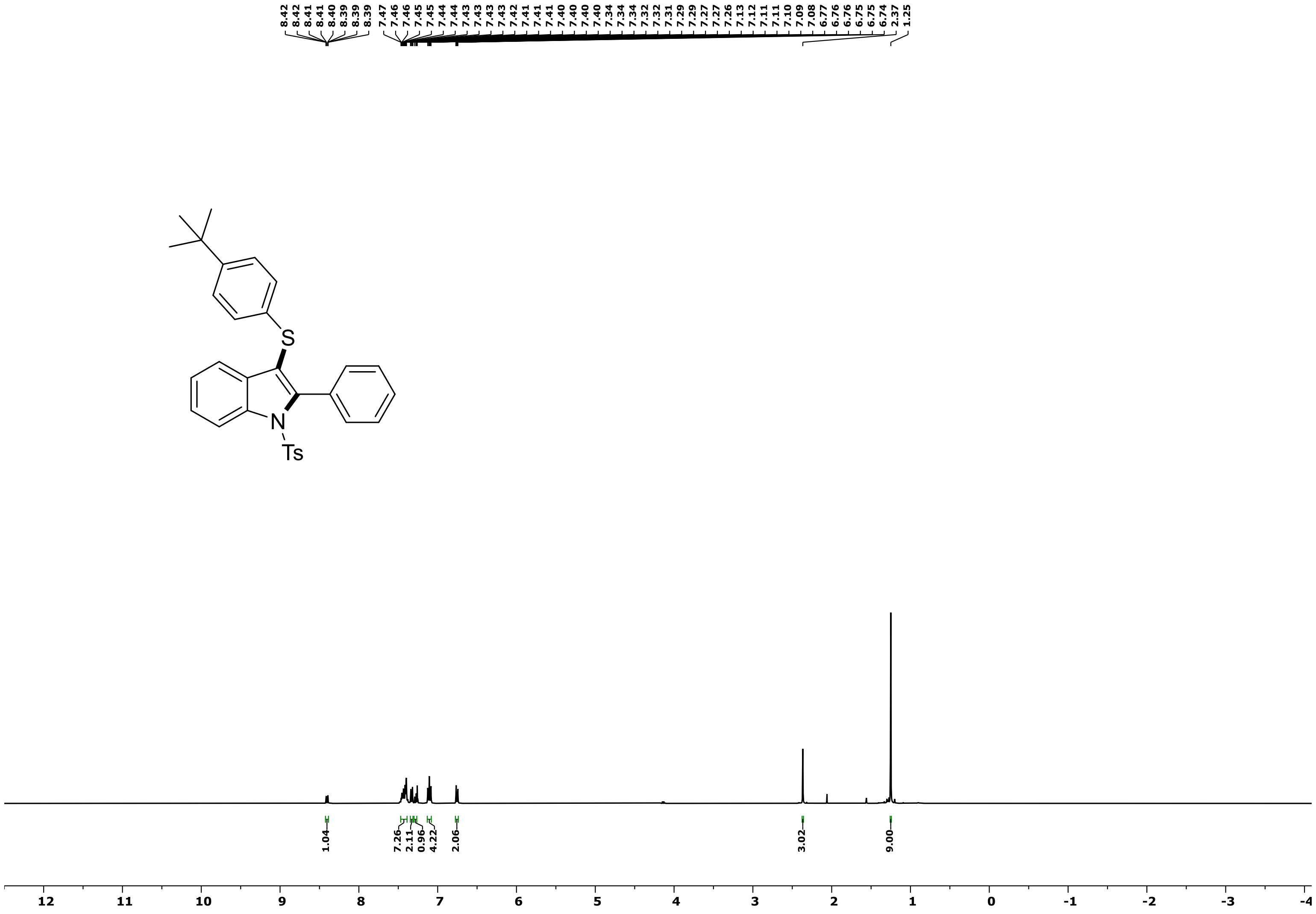

Figure S68: ^{13}C NMR (101 MHz, $CDCl_3$, 298 K) spectrum of *3-((4-(tert-butyl)phenyl)thio)-2-phenyl-1-tosyl-1H-indole*, **3aa**.

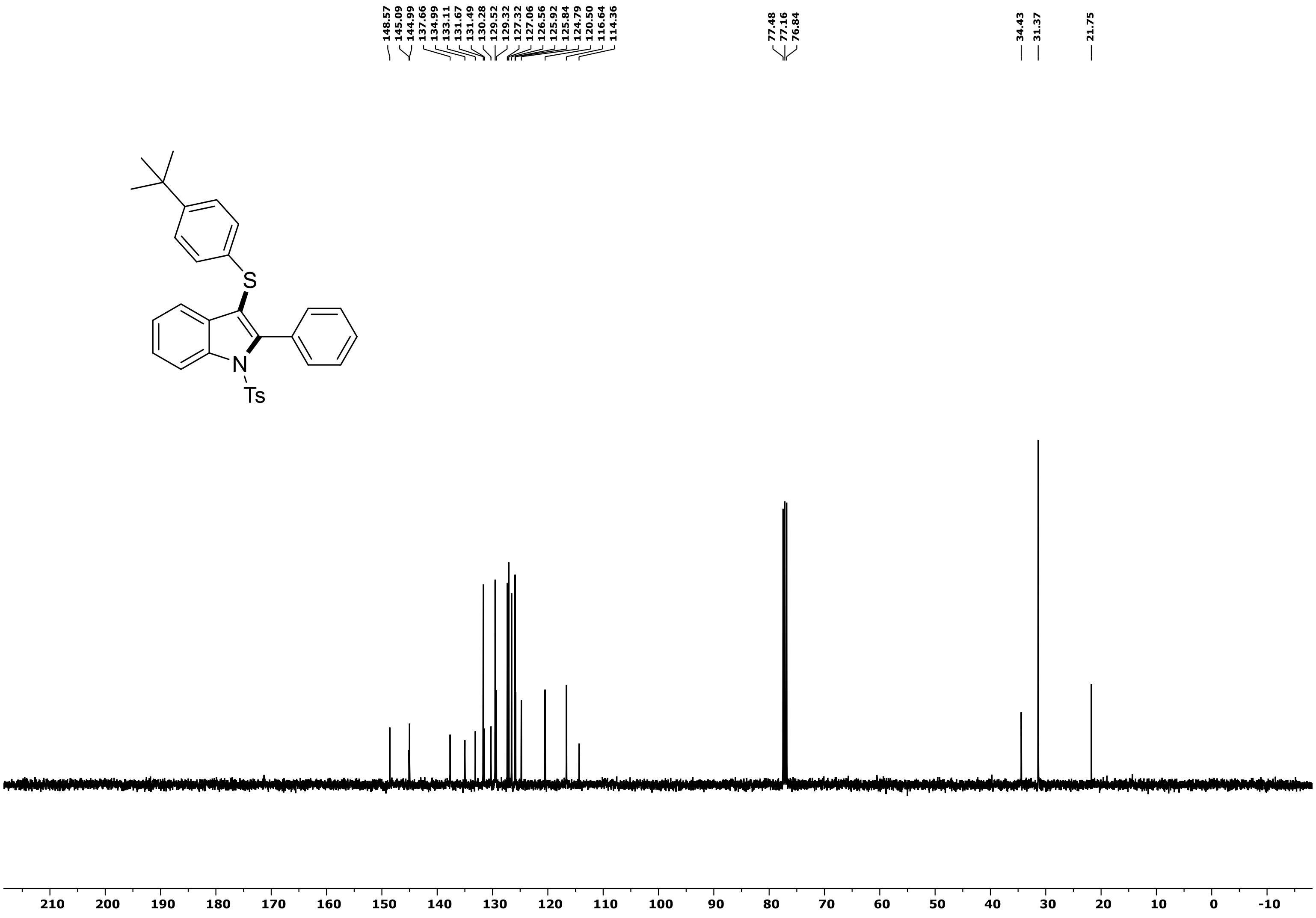

Figure S69: ^{1}H NMR (400 MHz, $CDCl_3$, 298 K) spectrum of *3-((4-(tert-butyl)phenyl)thio)-2-(p-tolyl)-1-tosyl-1H-indole*, **3ba**.

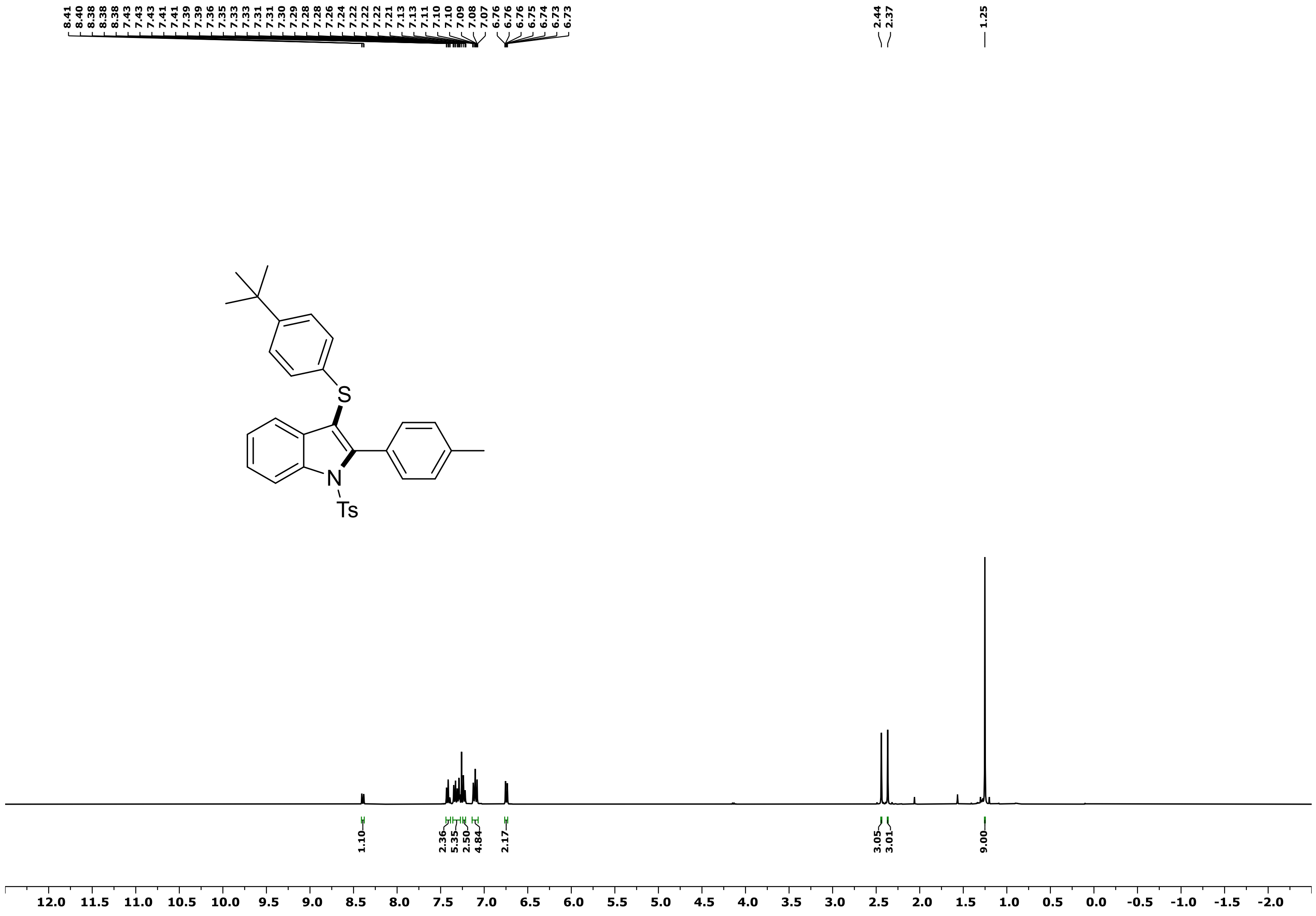

Figure S70: ^{13}C NMR (101 MHz, CDCl$_3$, 298 K) spectrum of *3-((4-(tert-butyl)phenyl)thio)-2-(p-tolyl)-1-tosyl-1H-indole*, **3ba**.

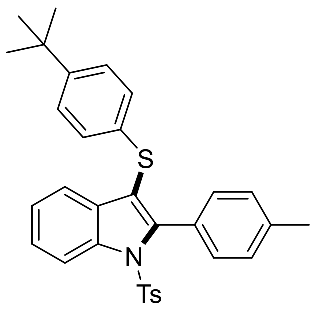

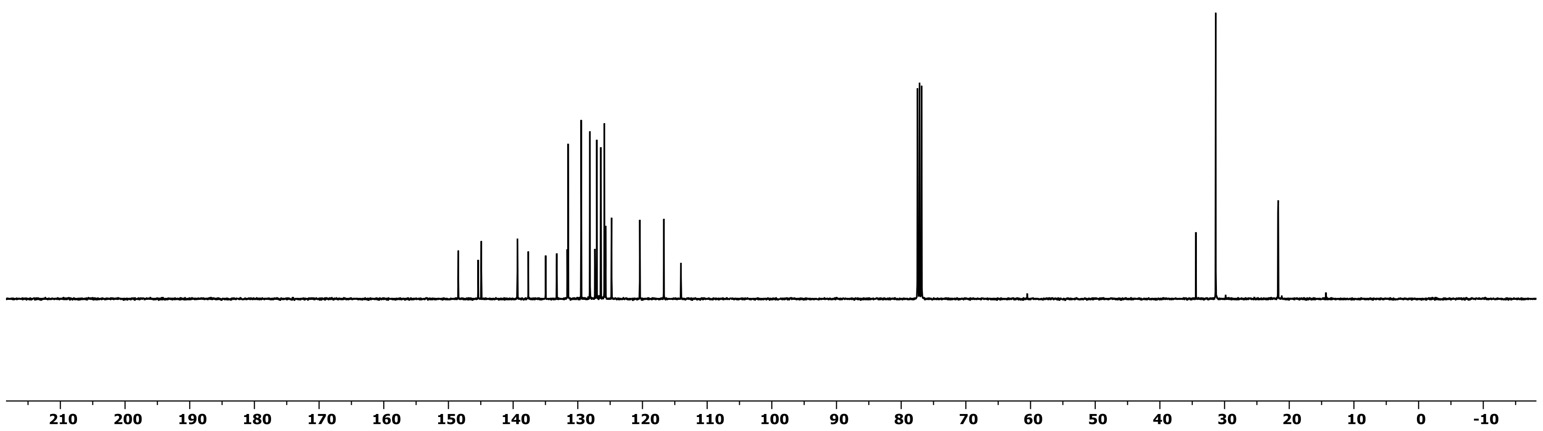

Figure S71: ^{1}H NMR (400 MHz, $CDCl_3$, 298 K) spectrum of *2-([1,1'-biphenyl]-4-yl)-3-((4-(tert-butyl)phenyl)thio)-1-tosyl-1H-indole*, **3ca**.

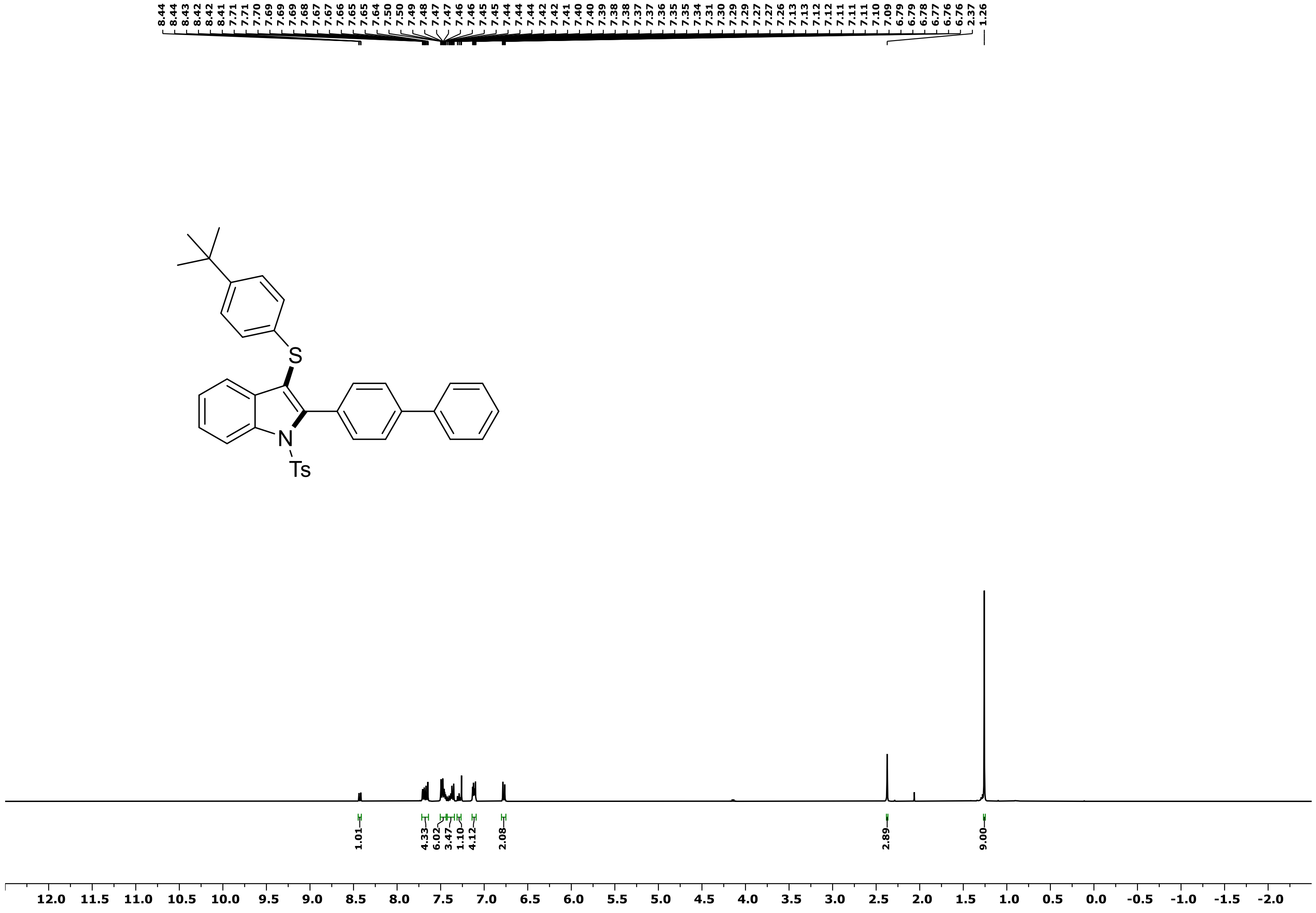

Figure S72: ^{13}C NMR (101 MHz, $CDCl_3$, 298 K) spectrum of *2-([1,1'-biphenyl]-4-yl)-3-((4-(tert-butyl)phenyl)thio)-1-tosyl-1H-indole*, **3ca**.

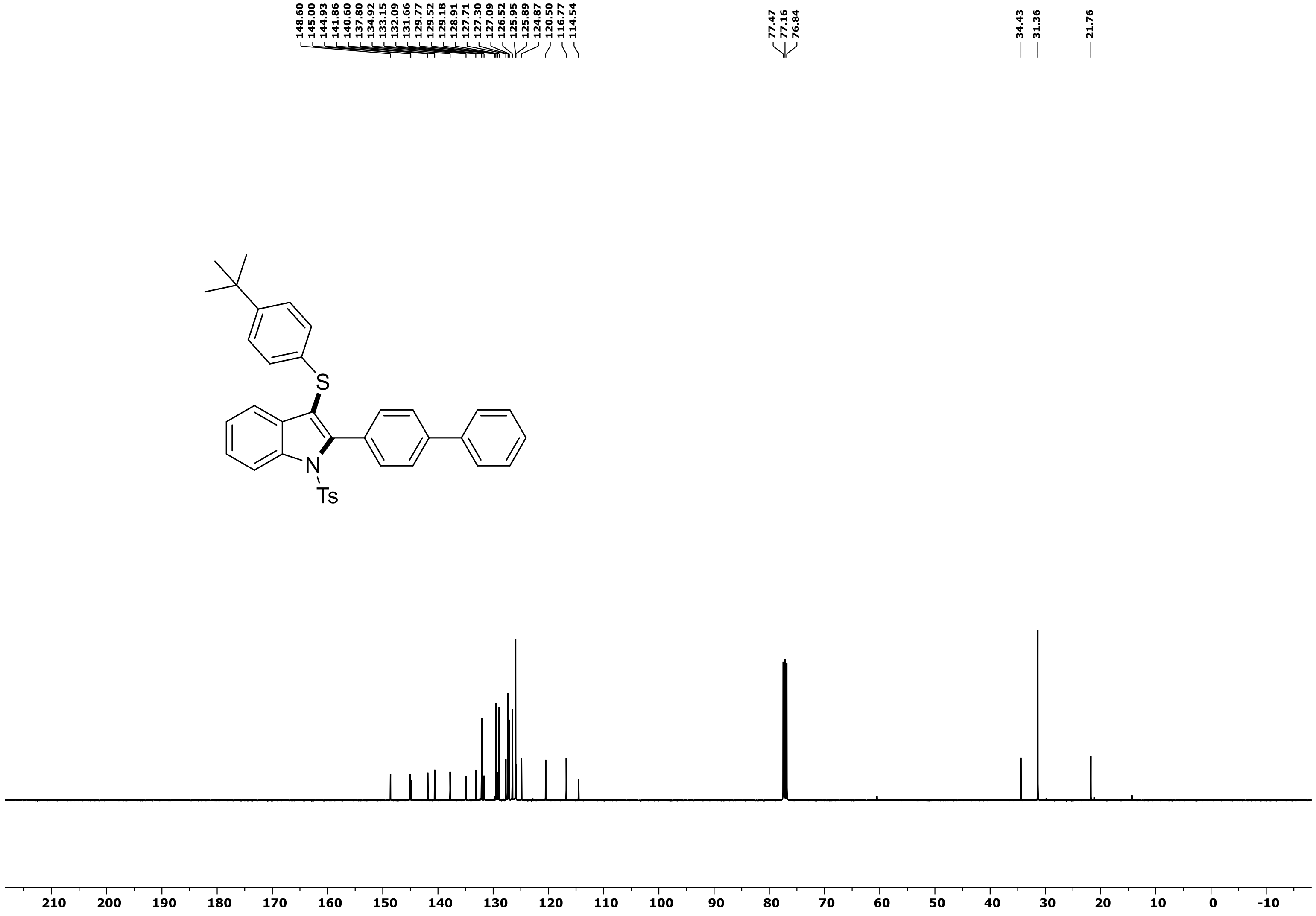

Figure S73: ^{1}H NMR (400 MHz, $CDCl_3$, 298 K) spectrum of *3-((4-(tert-butyl)phenyl)thio)-2-(phenanthren-9-yl)-1-tosyl-1H-indole*, **3da**.

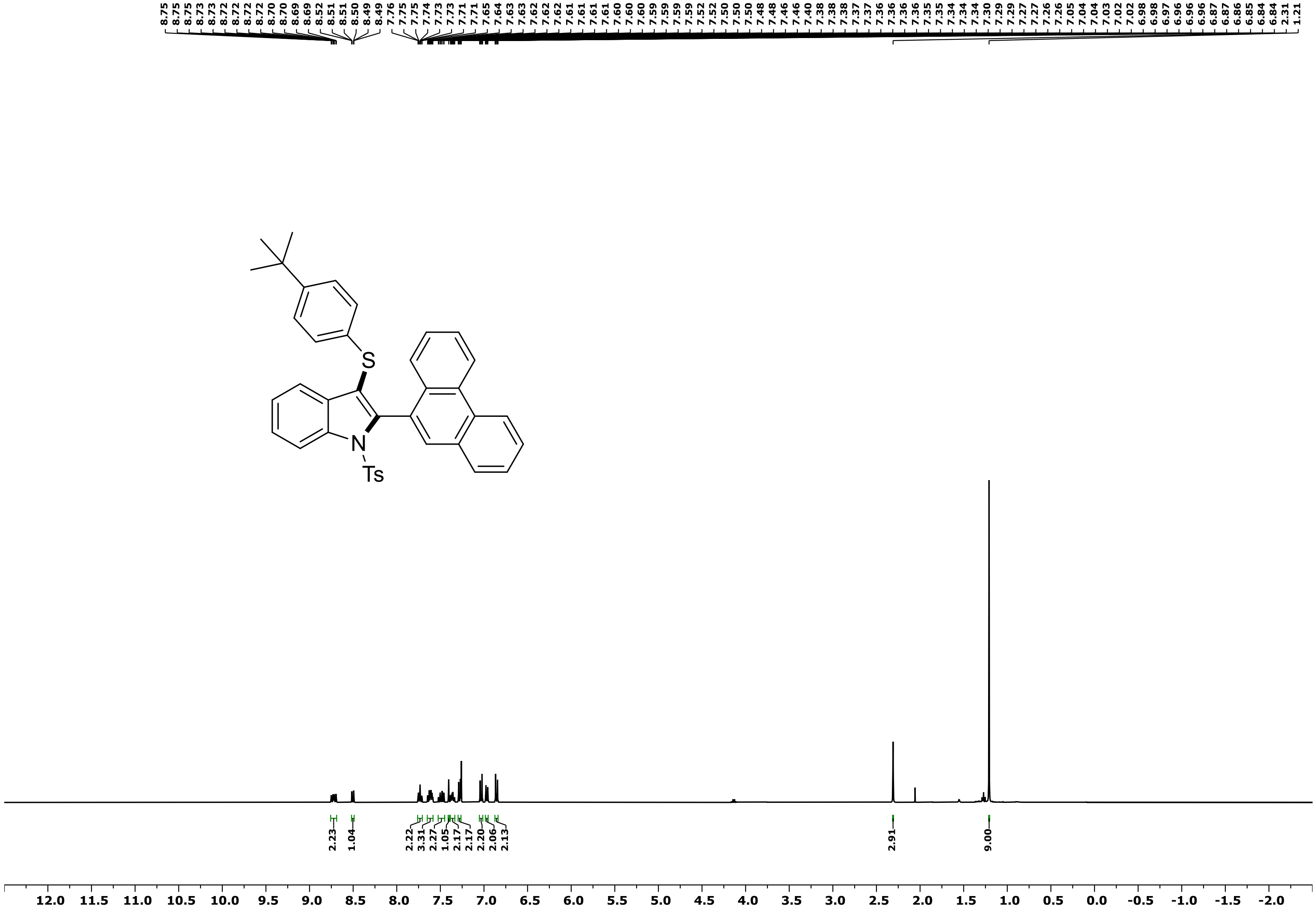

Figure S74: ^{13}C NMR (101 MHz, $CDCl_3$, 298 K) spectrum of *3-((4-(tert-butyl)phenyl)thio)-2-(phenanthren-9-yl)-1-tosyl-1H-indole*, **3da**.

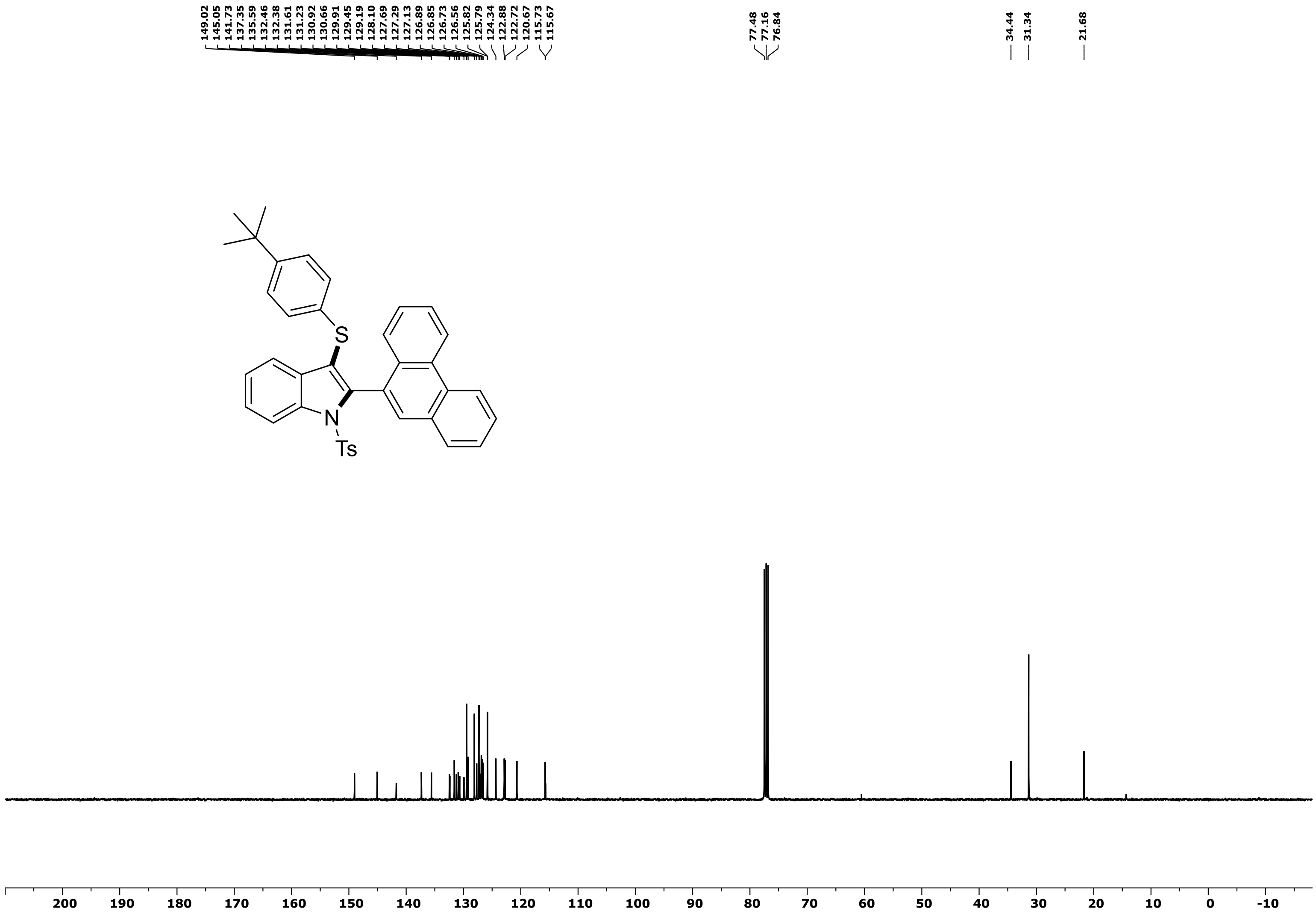

Figure S75: ^{1}H NMR (400 MHz, $CDCl_3$, 298 K) spectrum of *4-(3-((4-(tert-butyl)phenyl)thio)-1-tosyl-1H-indol-2-yl)-N,N-dimethylaniline*, **3ea**.

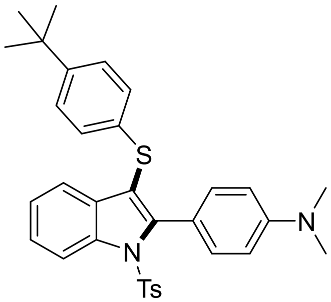

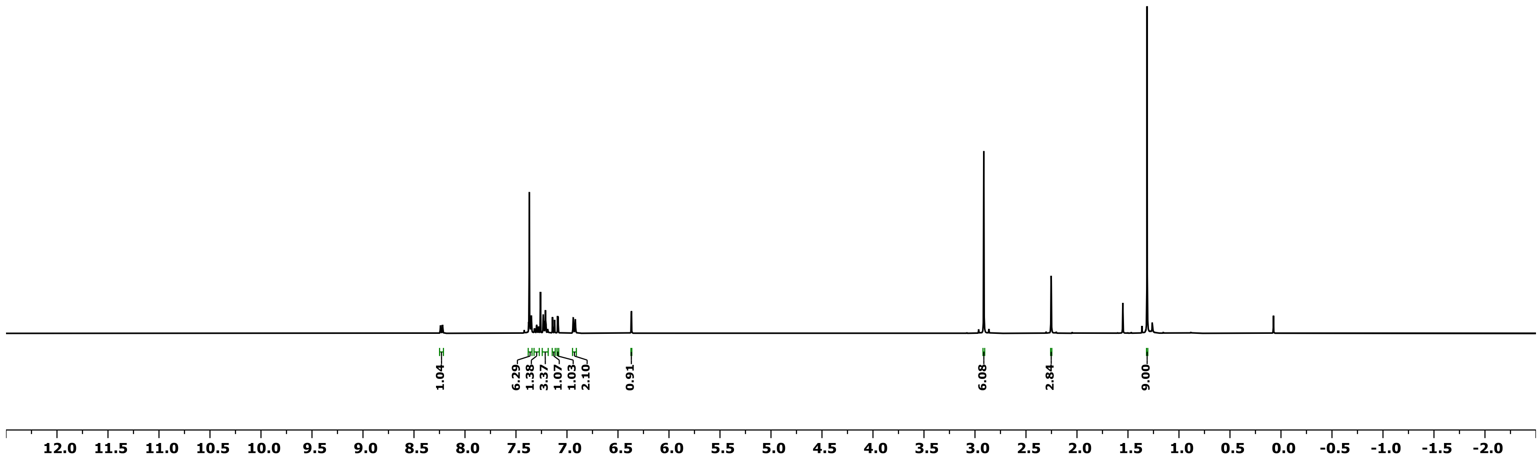

Figure S76: ^{13}C NMR (101 MHz, $CDCl_3$, 298 K) spectrum of *4-(3-((4-(tert-butyl)phenyl)thio)-1-tosyl-1H-indol-2-yl)-N,N-dimethylaniline*, **3ea**.

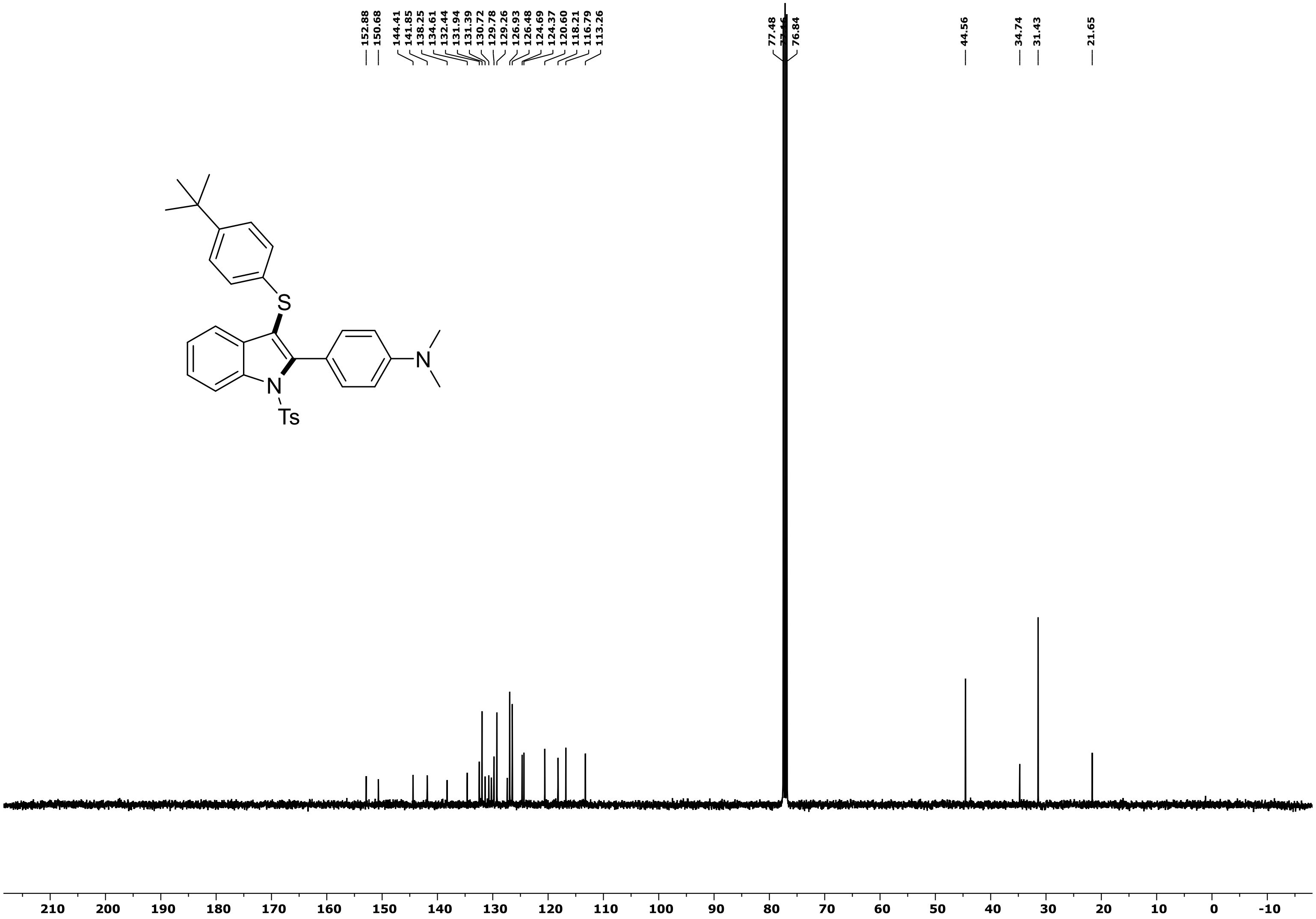

Figure S77: ^{1}H NMR (400 MHz, $CDCl_3$, 298 K) spectrum of *3-((4-(tert-butyl)phenyl)thio)-2-(4-bromophenyl)-1-tosyl-1H-indole,* **3fa**.

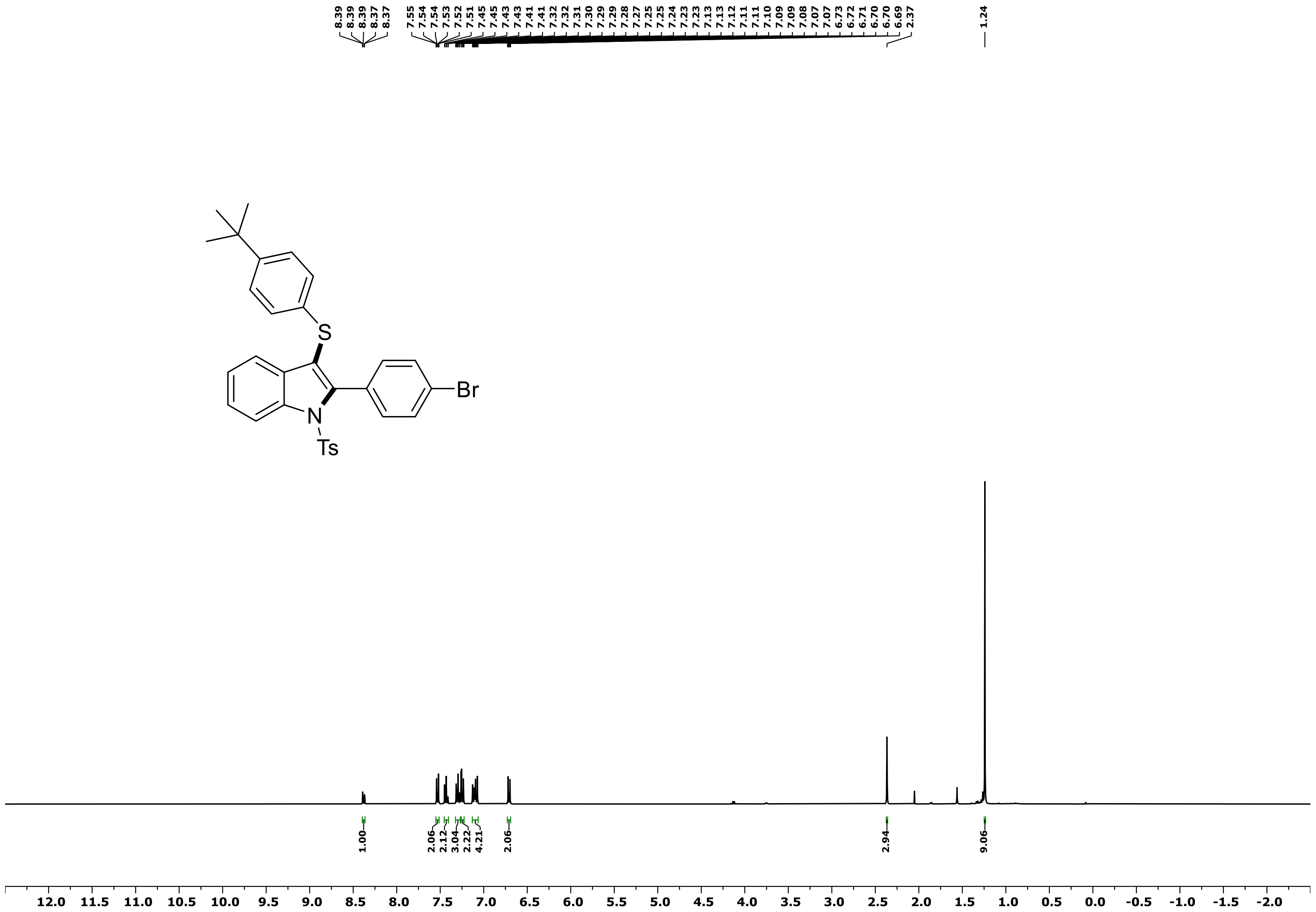

Figure S78: ^{13}C NMR (101 MHz, $CDCl_3$, 298 K) spectrum of *3-((4-(tert-butyl)phenyl)thio)-2-(4-bromophenyl)-1-tosyl-1H-indole,* **3fa**.

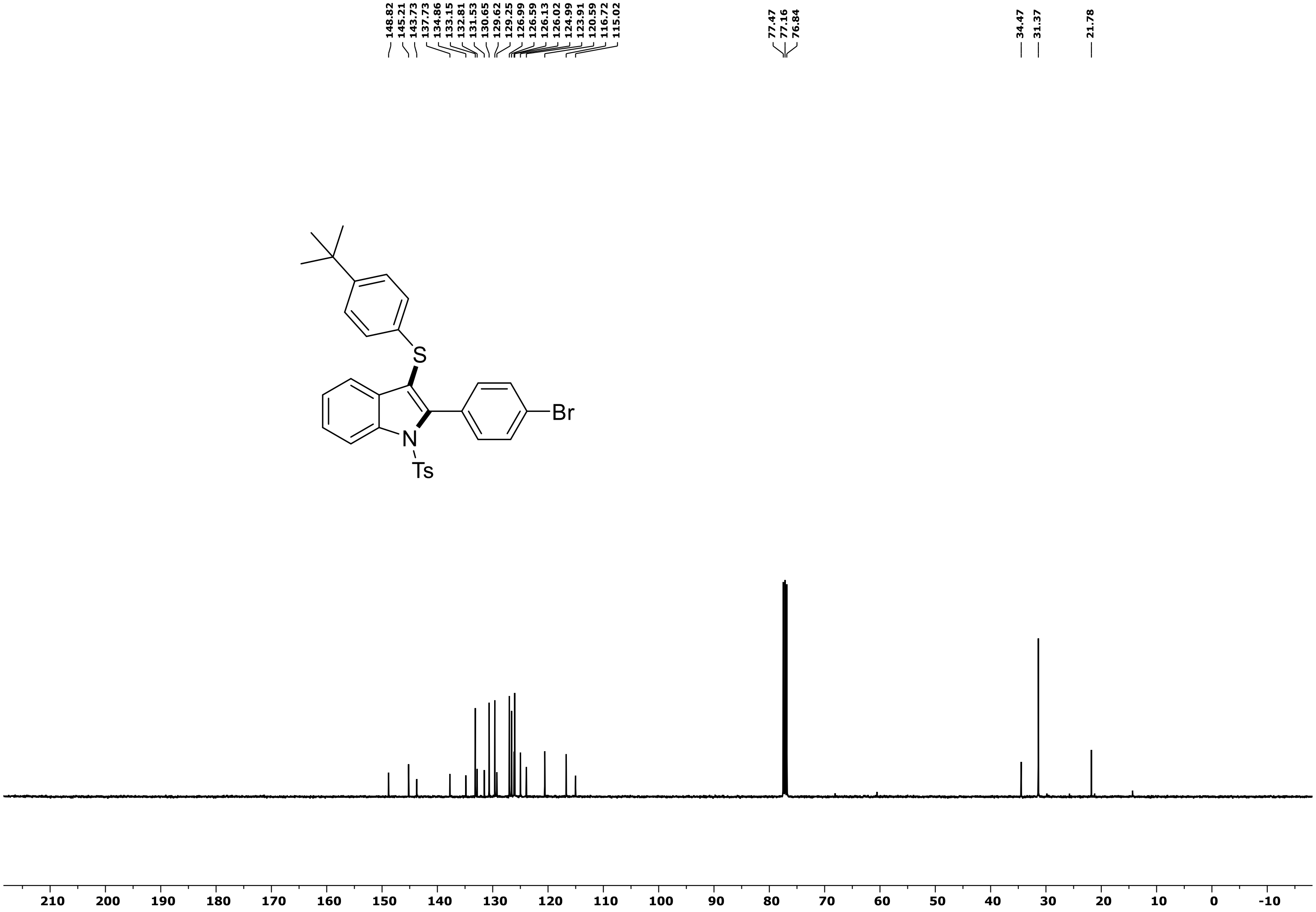

Figure S79: ^{1}H NMR (400 MHz, $CDCl_3$, 298 K) spectrum of *3-((4-(tert-butyl)phenyl)thio)-1-tosyl-2-(4-(trifluoromethyl)phenyl)-1H-indole*, **3ga**.

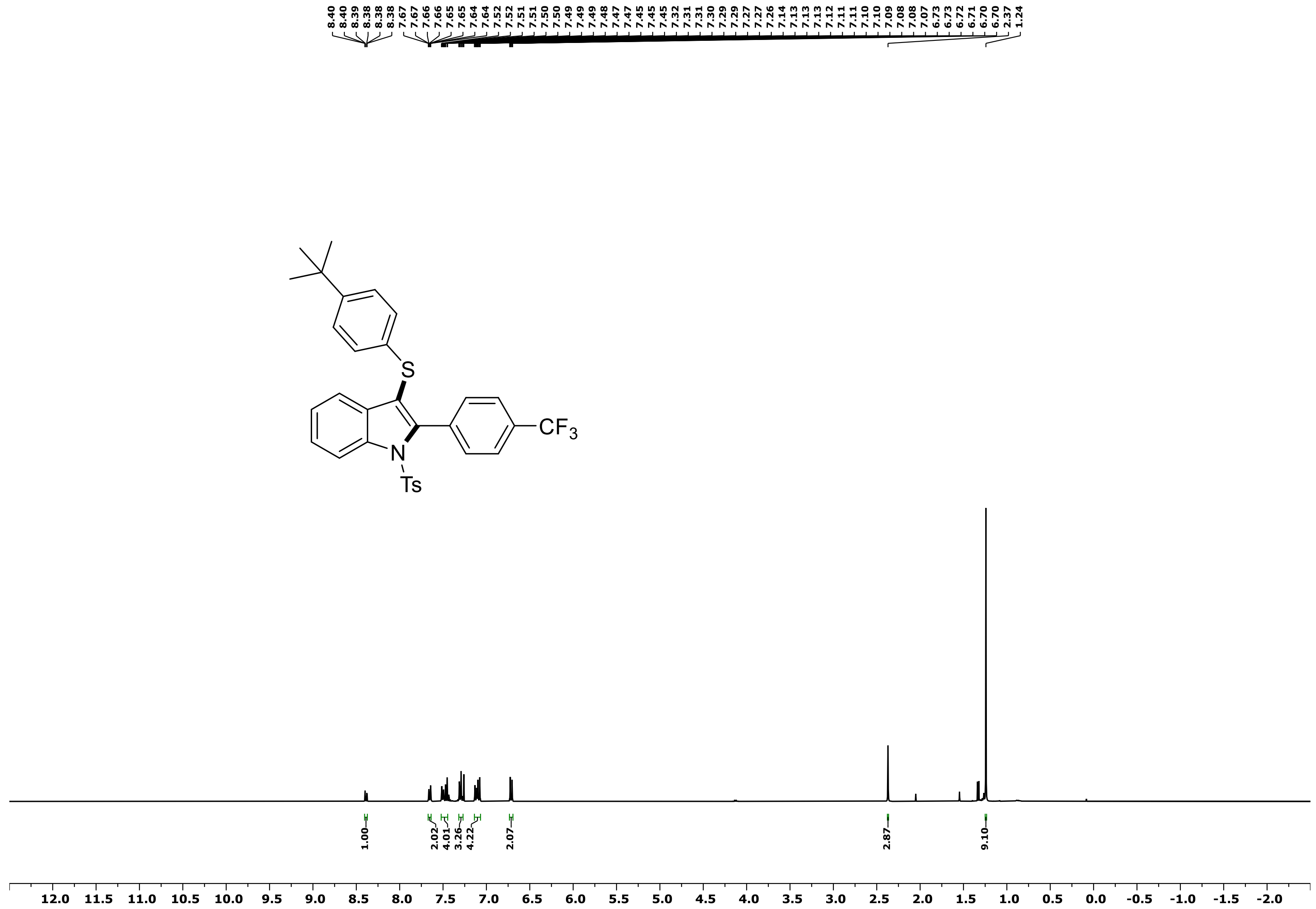

Figure S80: ^{13}C NMR (101 MHz, $CDCl_3$, 298 K) spectrum of *3-((4-(tert-butyl)phenyl)thio)-1-tosyl-2-(4-(trifluoromethyl)phenyl)-1H-indole*, **3ga**.

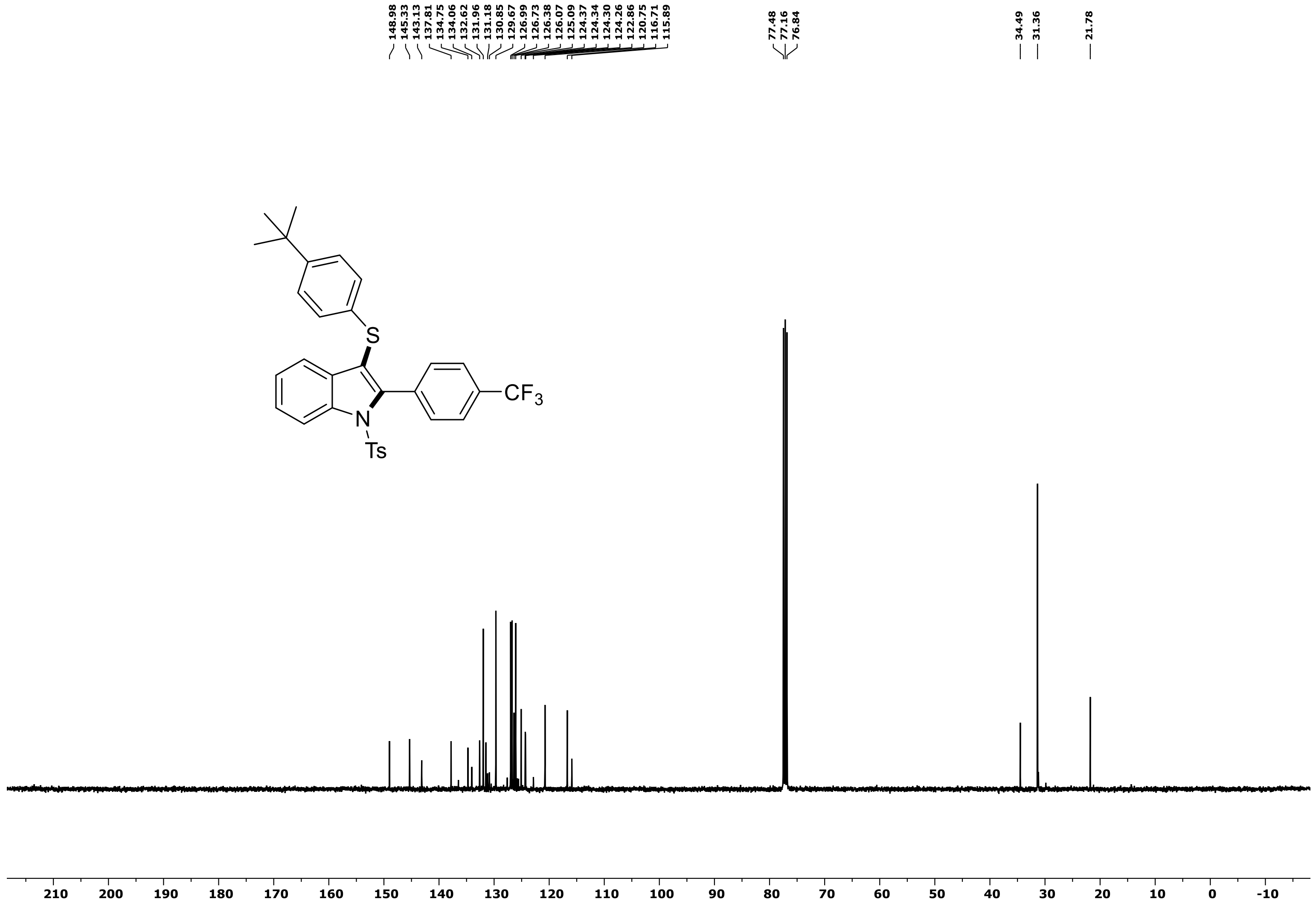

Figure S81: ^{19}F NMR (376 MHz, $CDCl_3$, 298 K) spectrum of *3-((4-(tert-butyl)phenyl)thio)-1-tosyl-2-(4-(trifluoromethyl)phenyl)-1H-indole*, **3ga**.

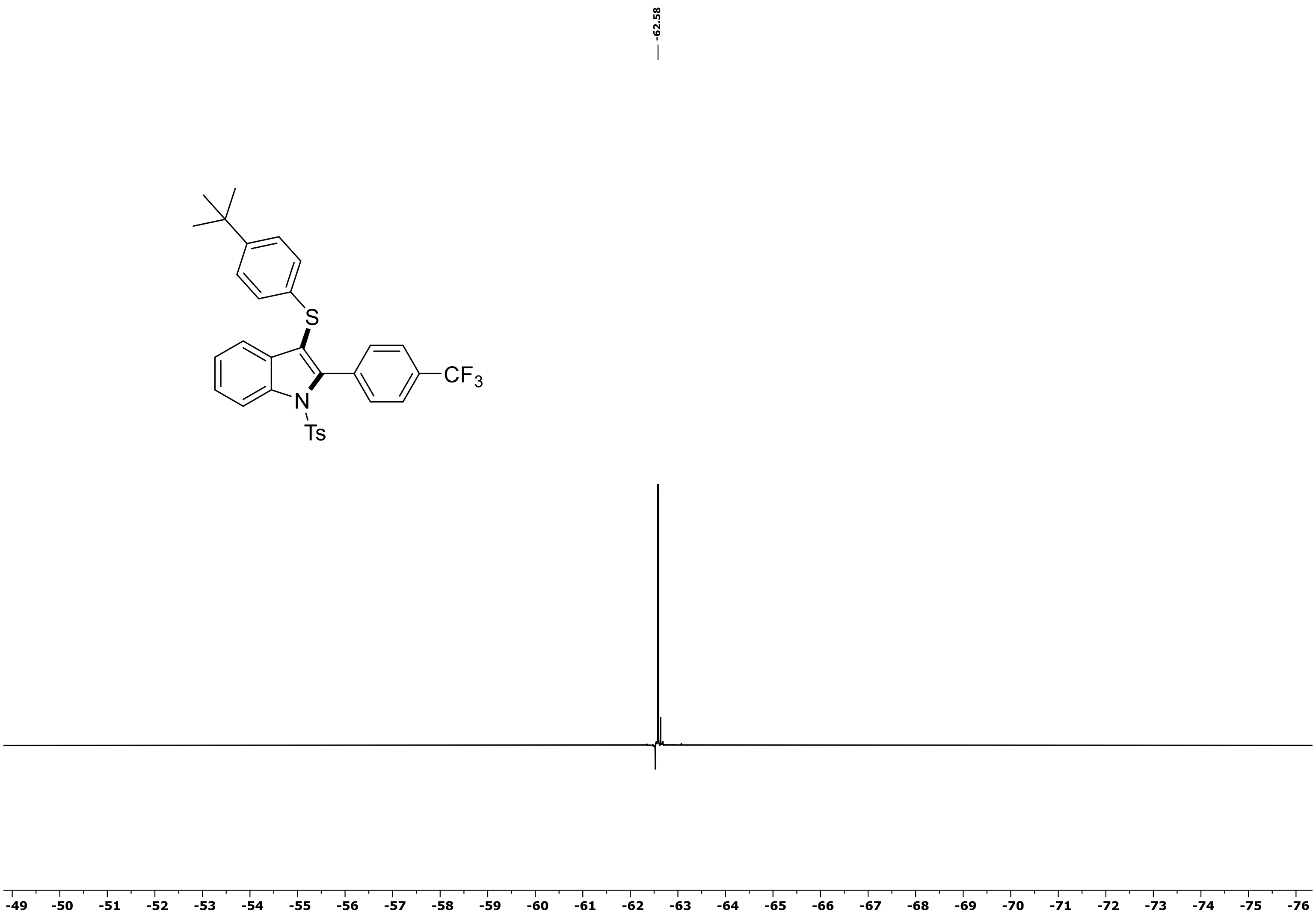

Figure S82: ^{1}H NMR (400 MHz, $CDCl_3$, 298 K) spectrum of *3-((4-(tert-butyl)phenyl)thio)-2-(cyclohex-1-en-1-yl)-1-tosyl-1H-indole*, **3ha**.

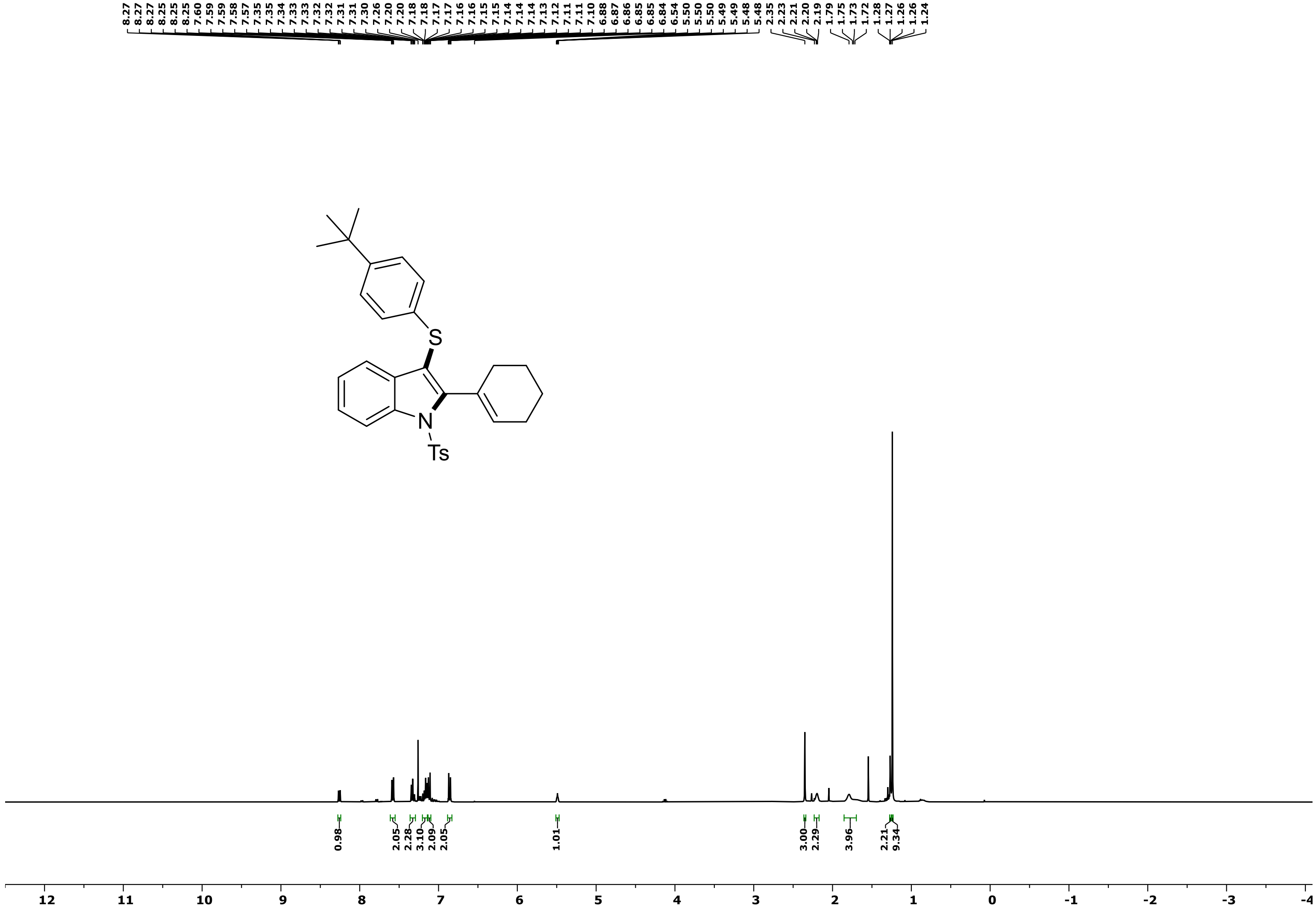

Figure S83: ^{13}C NMR (101 MHz, $CDCl_3$, 298 K) spectrum of *3-((4-(tert-butyl)phenyl)thio)-2-(cyclohex-1-en-1-yl)-1-tosyl-1H-indole*, **3ha**.

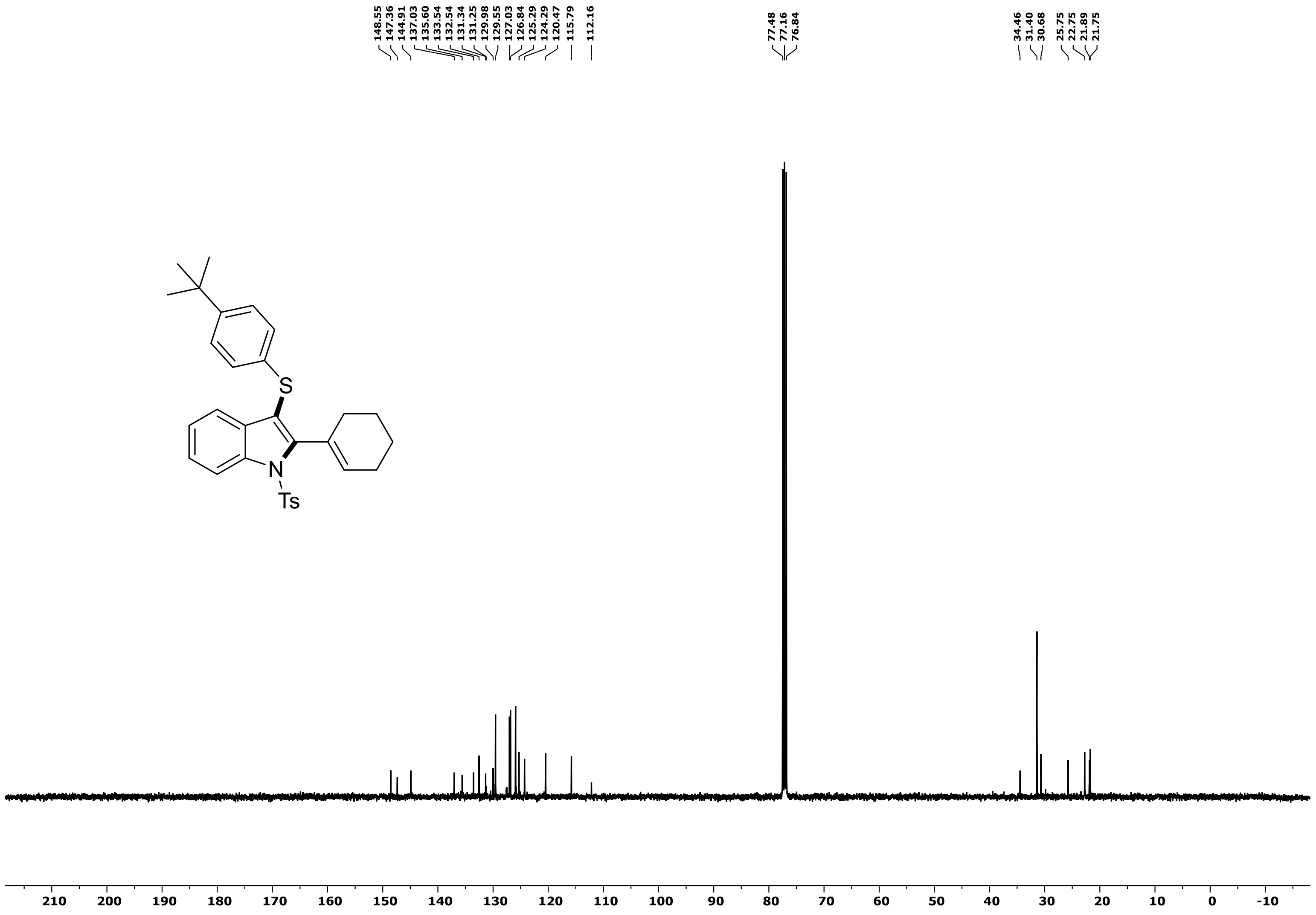

Figure S84: ^{1}H NMR (400 MHz, $CDCl_3$, 298 K) spectrum of *2-butyl-3-((4-(tert-butyl)phenyl)thio)-1-tosyl-1H-indole*, **3ia**.

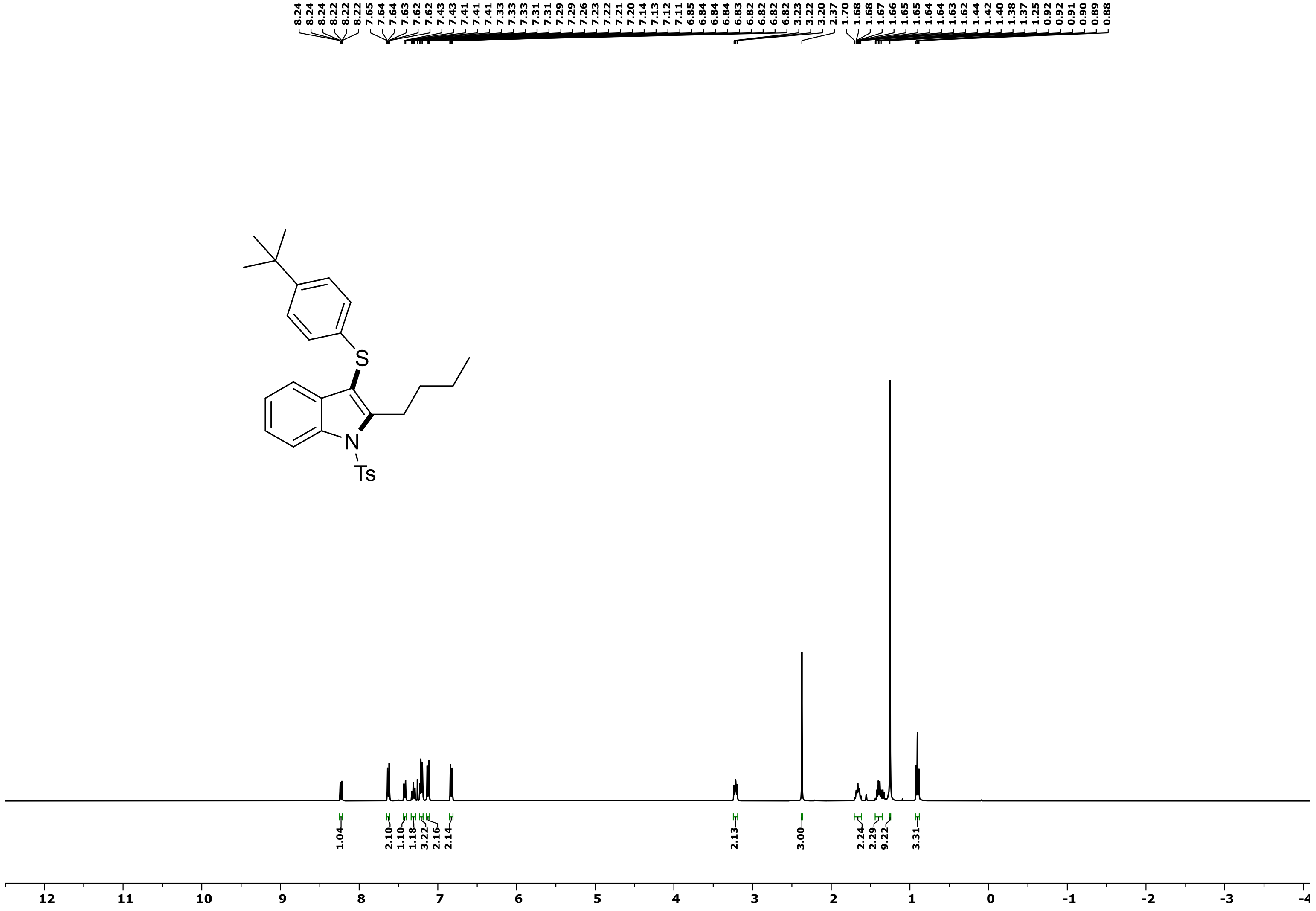

Figure S85: ^{13}C NMR (101 MHz, $CDCl_3$, 298 K) spectrum of *2-butyl-3-((4-(tert-butyl)phenyl)thio)-1-tosyl-1H-indole*, **3ia**.

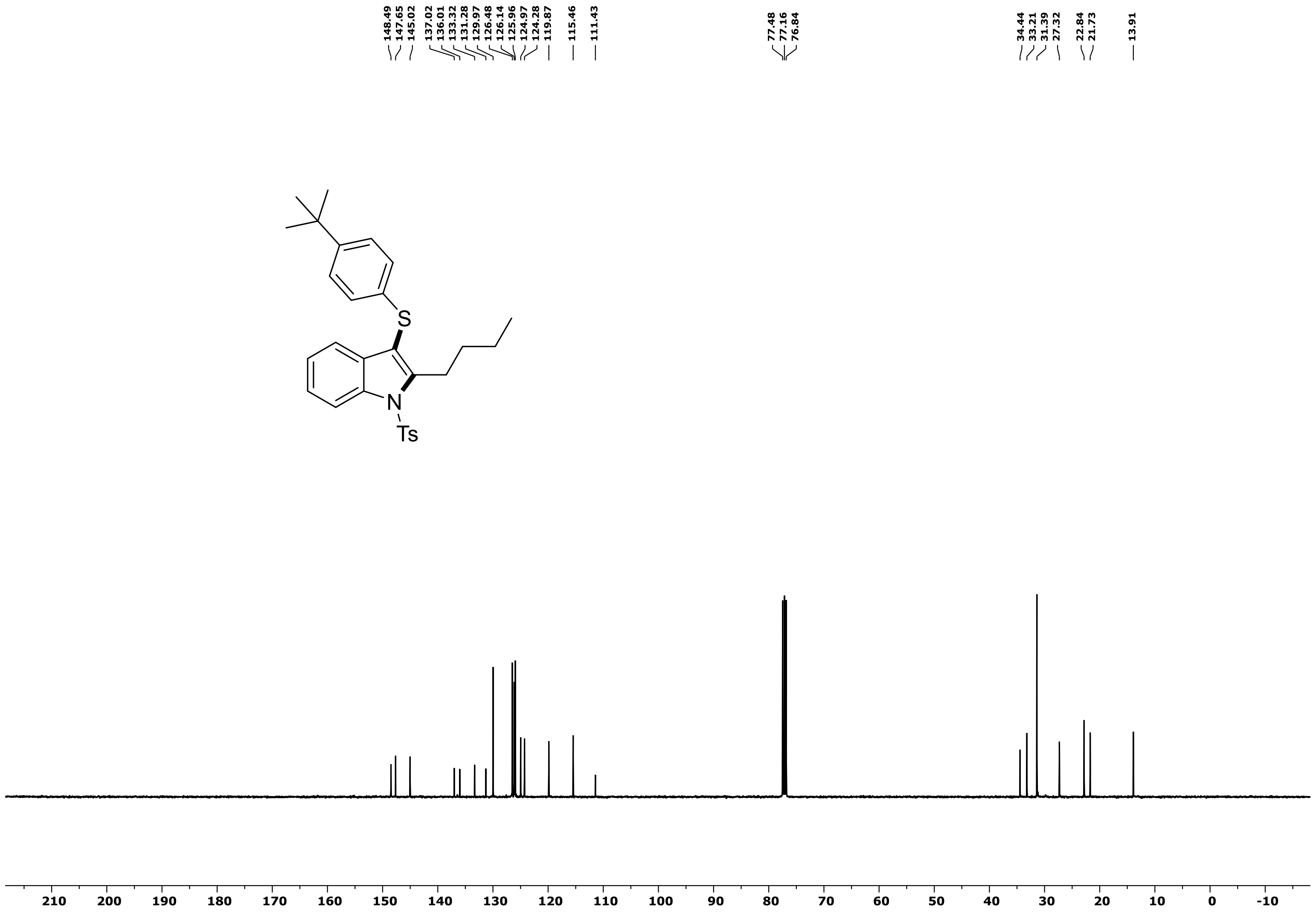

Figure S86: ^{1}H NMR (400 MHz, $CDCl_3$, 298 K) spectrum of *3-((4-(tert-butyl)phenyl)thio)-1-tosyl-2-(trimethylsilyl)-1H-indole*, **3ja**.

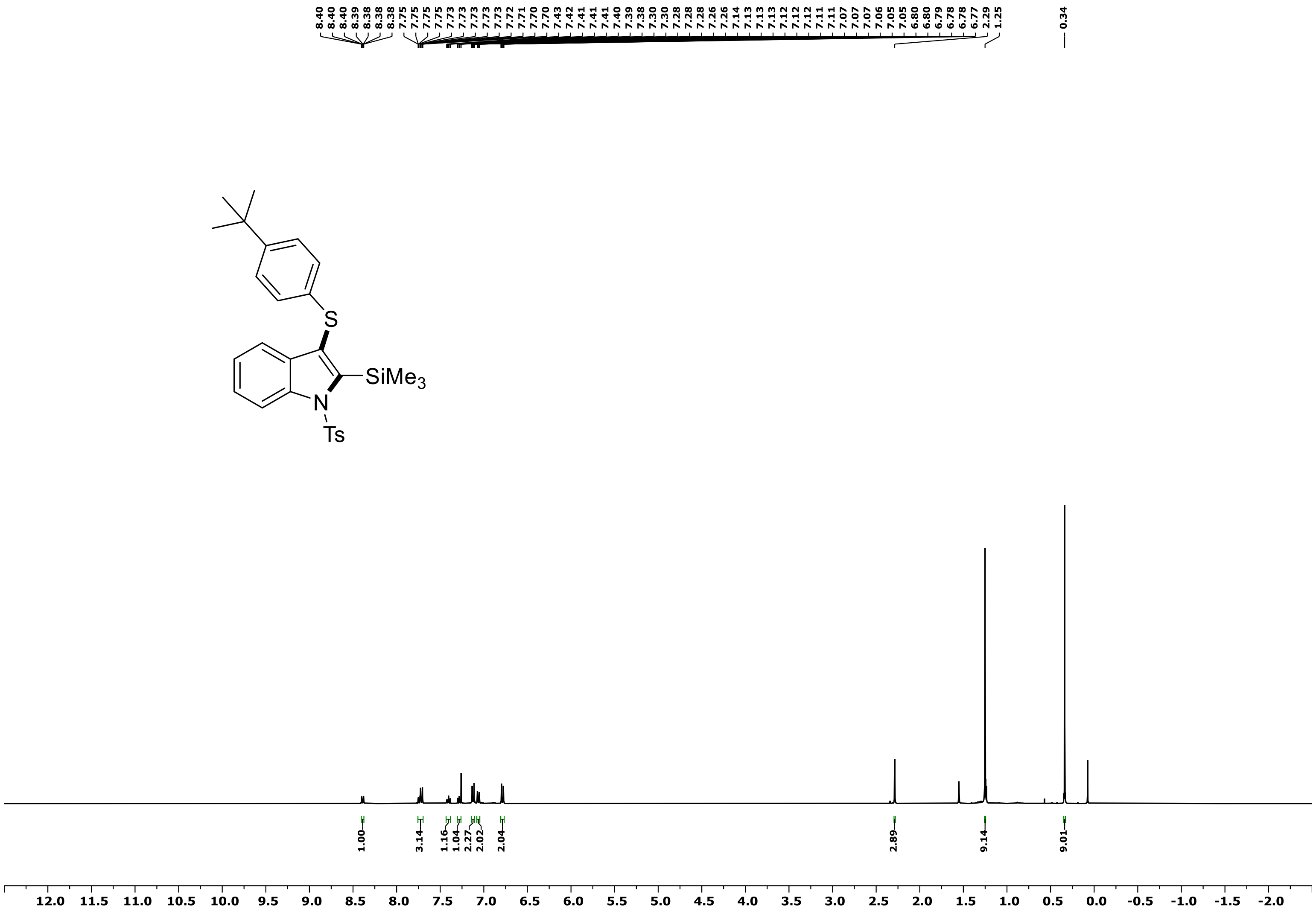

Figure S87: ^{13}C NMR (101 MHz, $CDCl_3$, 298 K) spectrum of *3-((4-(tert-butyl)phenyl)thio)-1-tosyl-2-(trimethylsilyl)-1H-indole*, **3ja**.

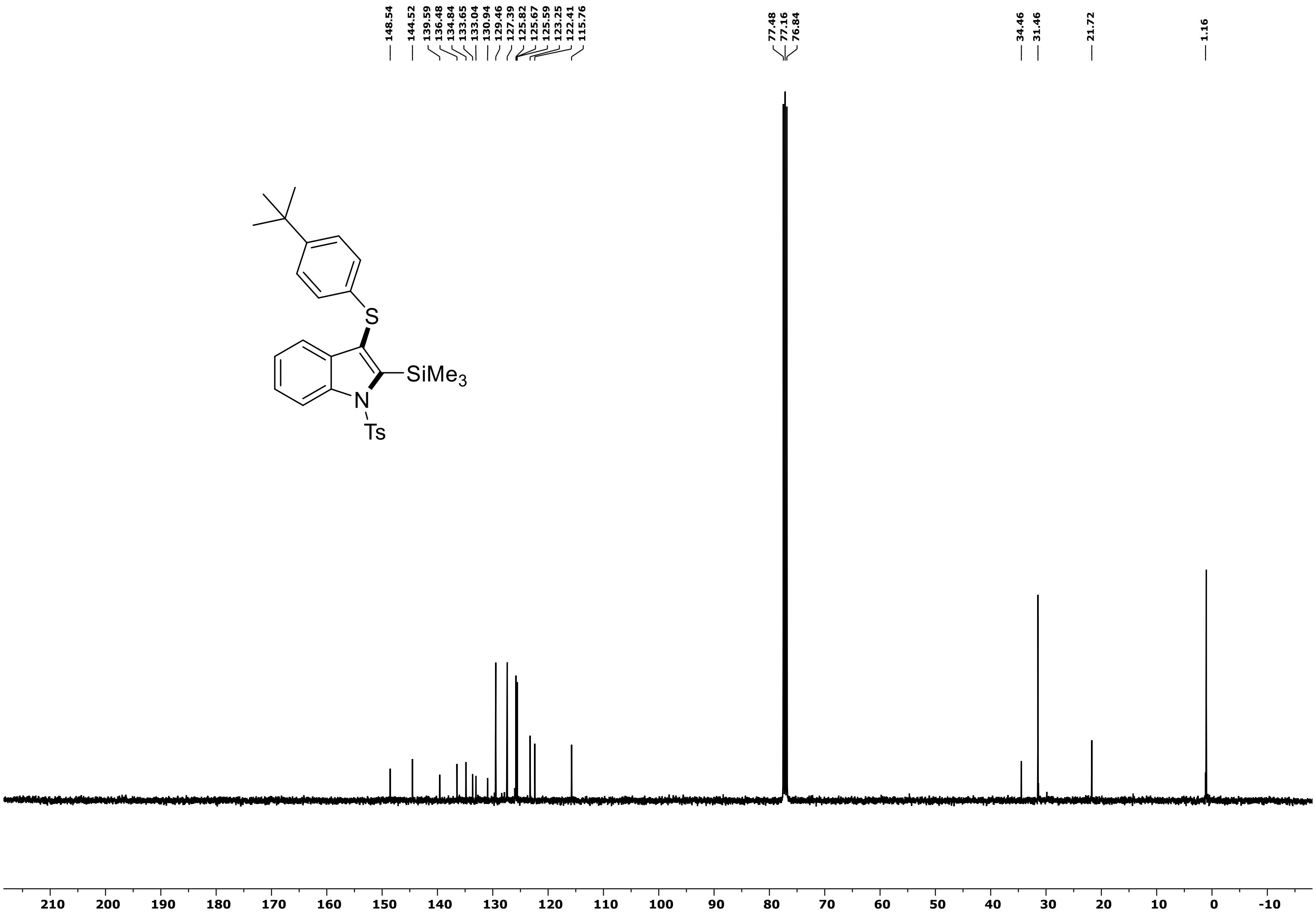

Figure S88: ^{1}H NMR (400 MHz, $CDCl_3$, 298 K) spectrum of *3-((4-(tert-butyl)phenyl)thio)-2-phenylbenzofuran*, **3ka**.

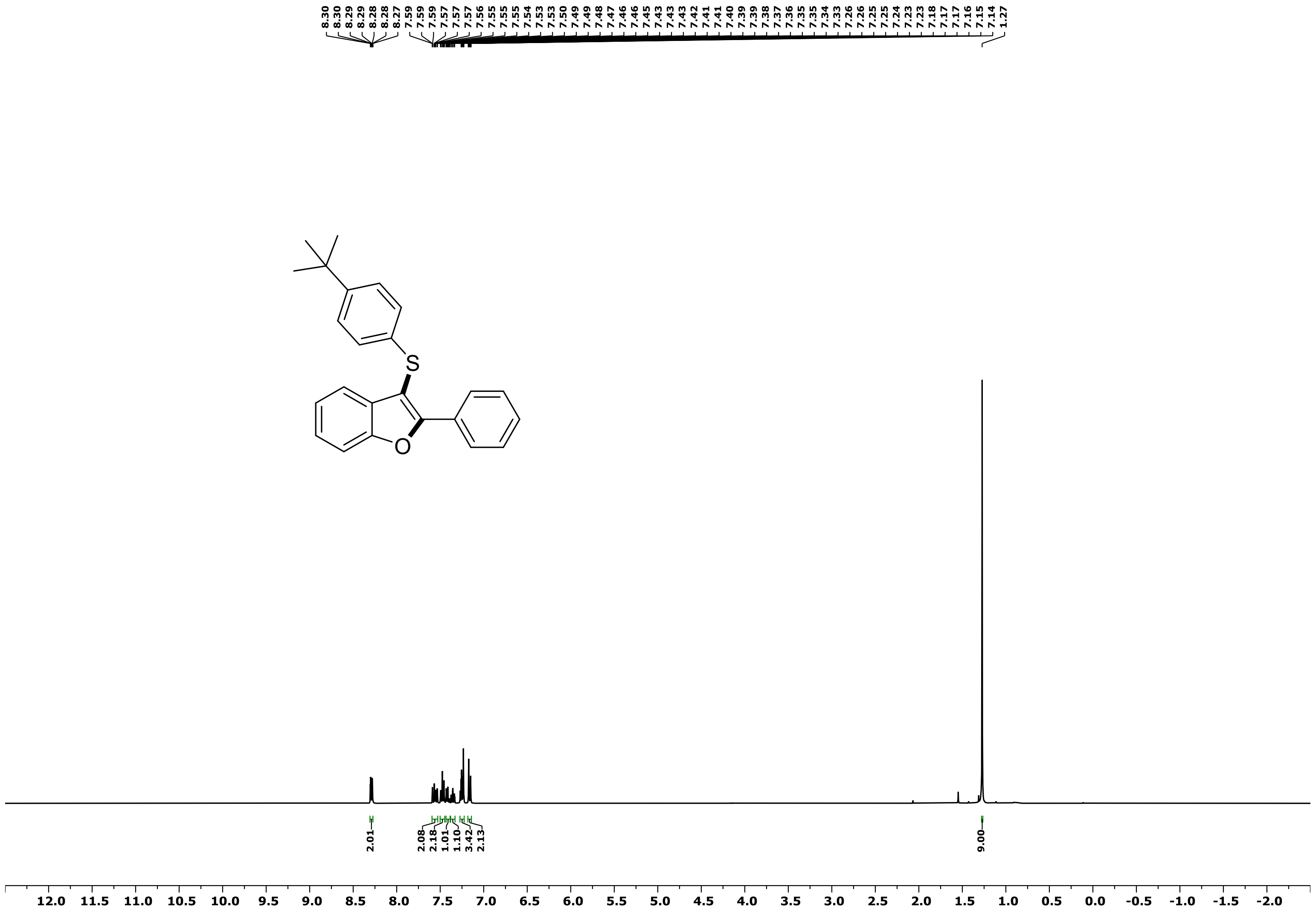

Figure S89: ^{13}C NMR (101 MHz, $CDCl_3$, 298 K) spectrum of *3-((4-(tert-butyl)phenyl)thio)-2-phenylbenzofuran*, **3ka**.

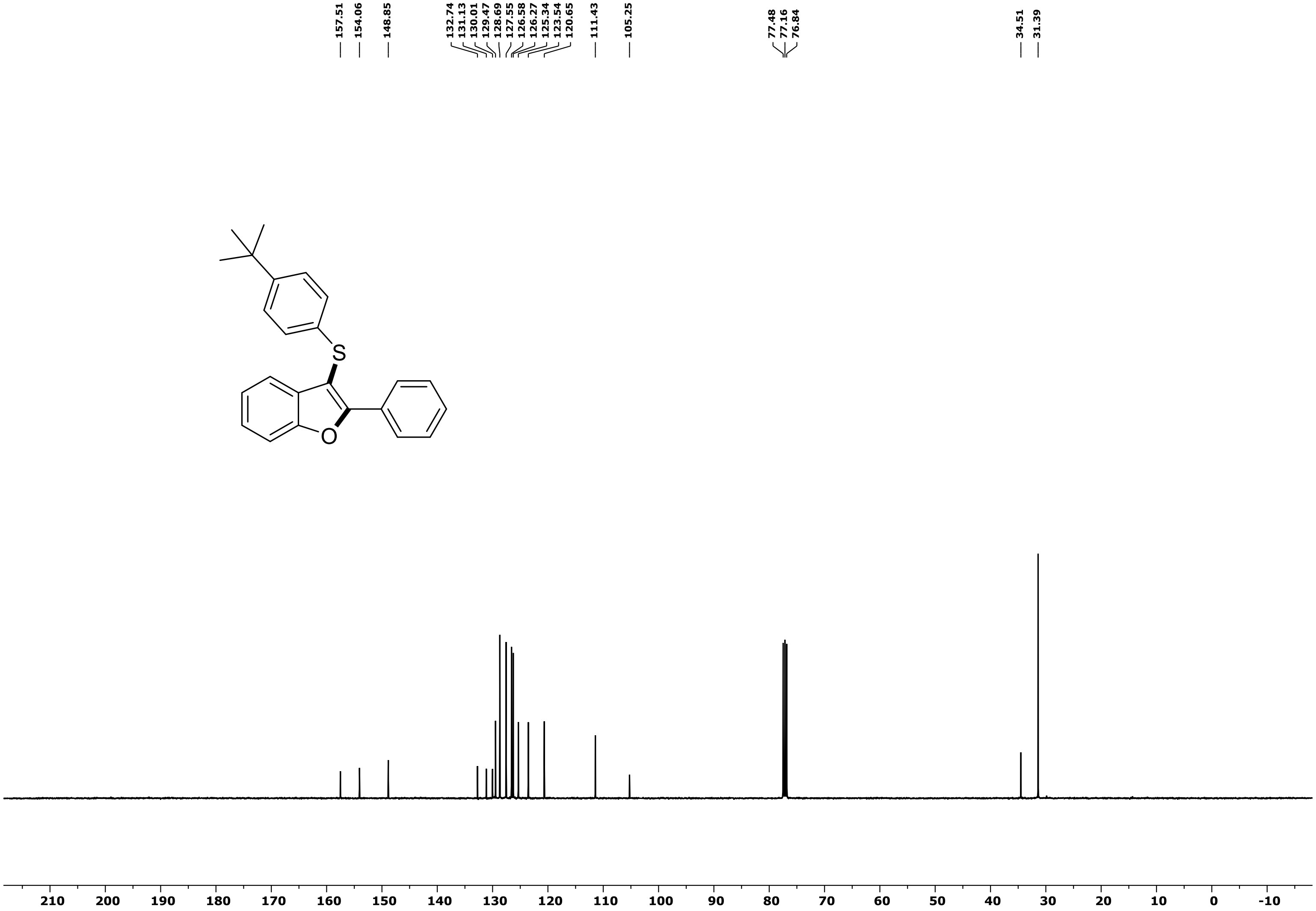

Figure S90: ^{1}H NMR (400 MHz, $CDCl_3$, 298 K) spectrum of *3-((4-(tert-butyl)phenyl)thio)-5-methyl-2-phenylbenzofuran*, **3la**.

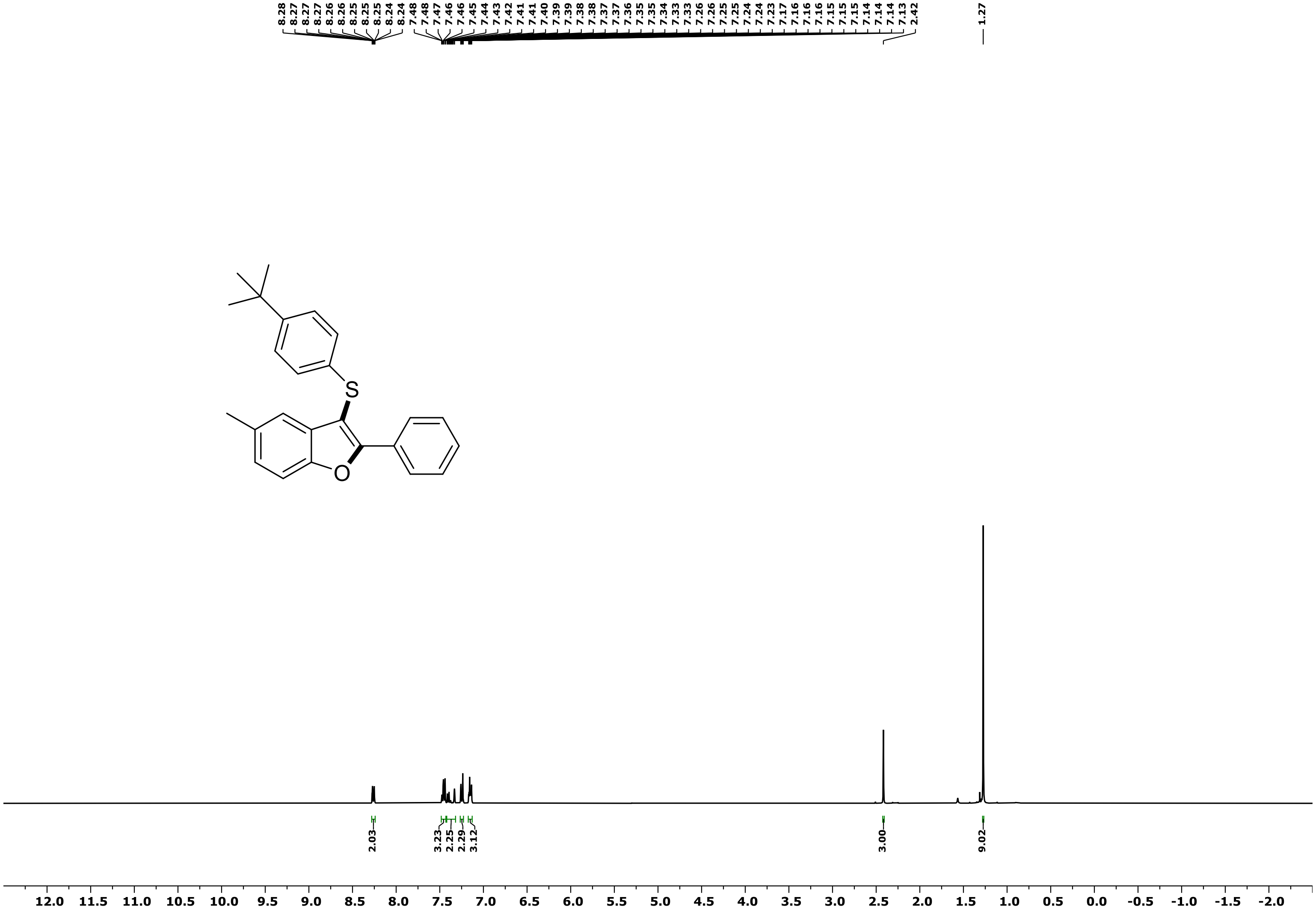

Figure S91: ^{13}C NMR (101 MHz, $CDCl_3$, 298 K) spectrum of *3-((4-(tert-butyl)phenyl)thio)-5-methyl-2-phenylbenzofuran*, **3la**.

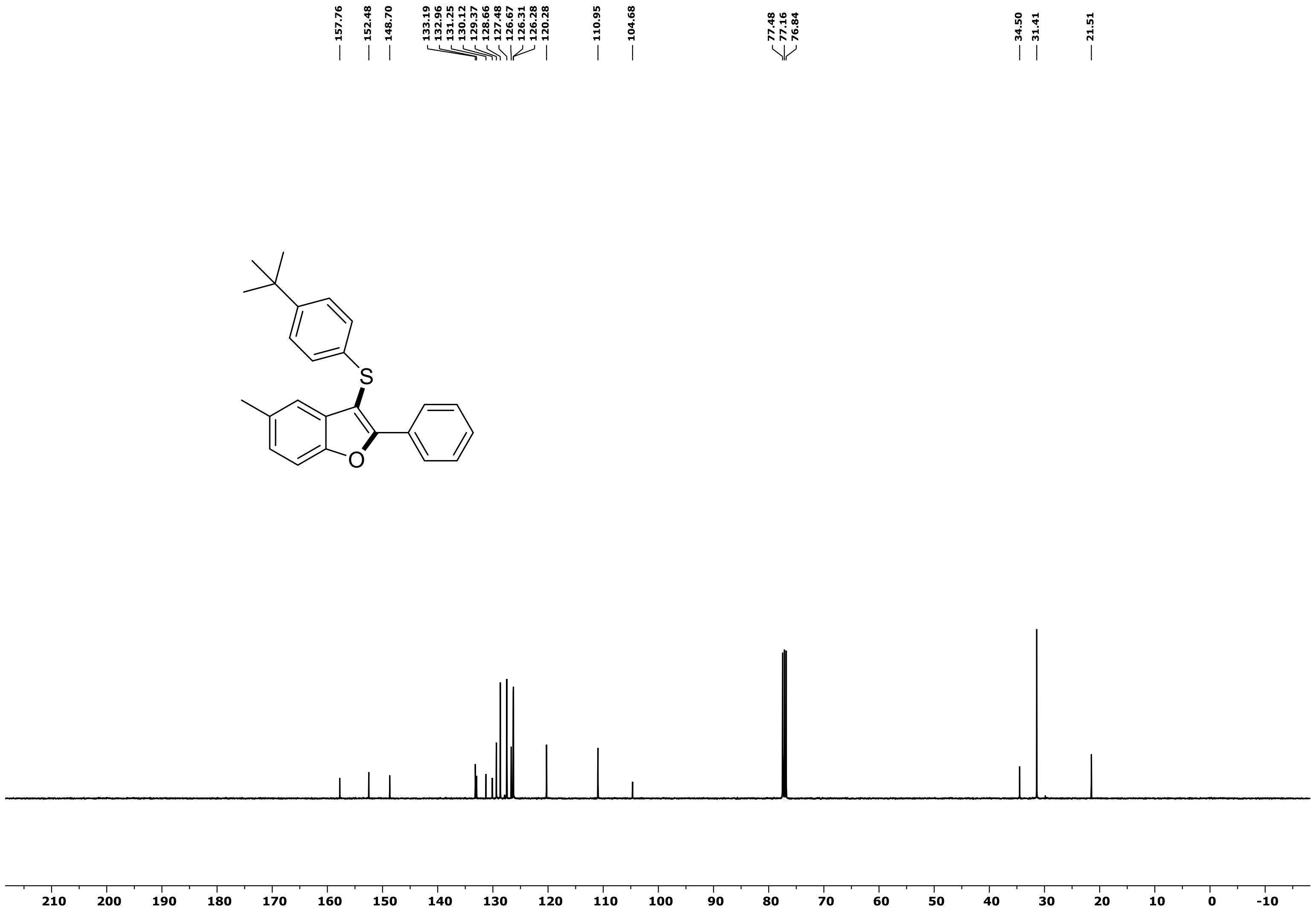

Figure S92: ^{1}H NMR (400 MHz, $CDCl_3$, 298 K) spectrum of *3-((4-(tert-butyl)phenyl)thio)-5-chloro-2-phenylbenzofuran*, **3ma**.

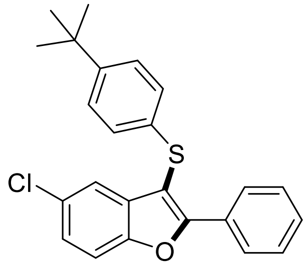

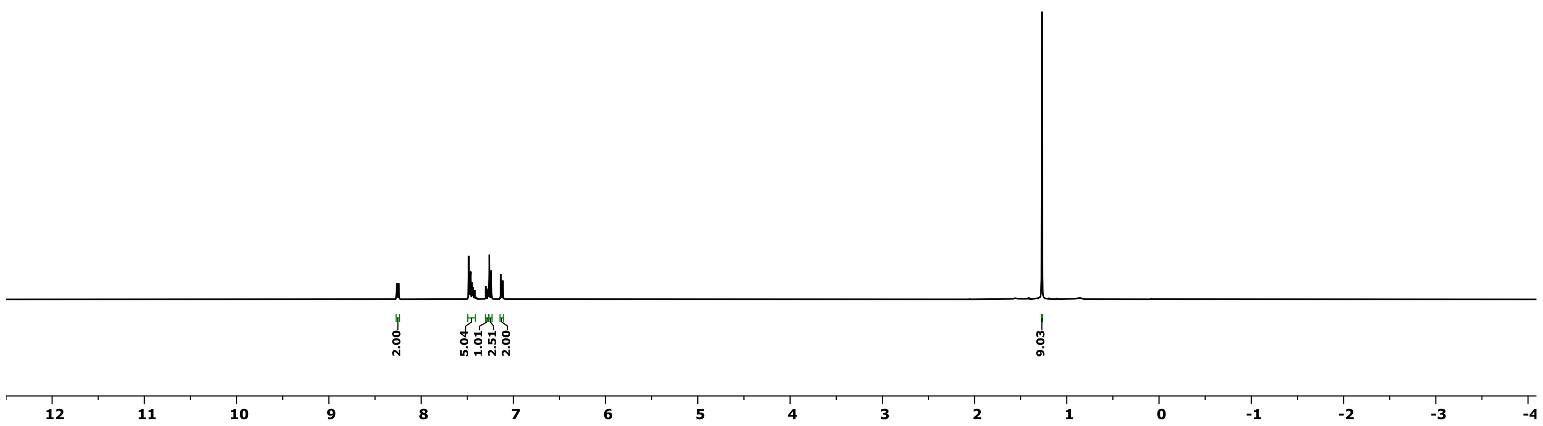

Figure S93: ^{13}C NMR (101 MHz, $CDCl_3$, 298 K) spectrum of *3-((4-(tert-butyl)phenyl)thio)-5-chloro-2-phenylbenzofuran*, **3ma**.

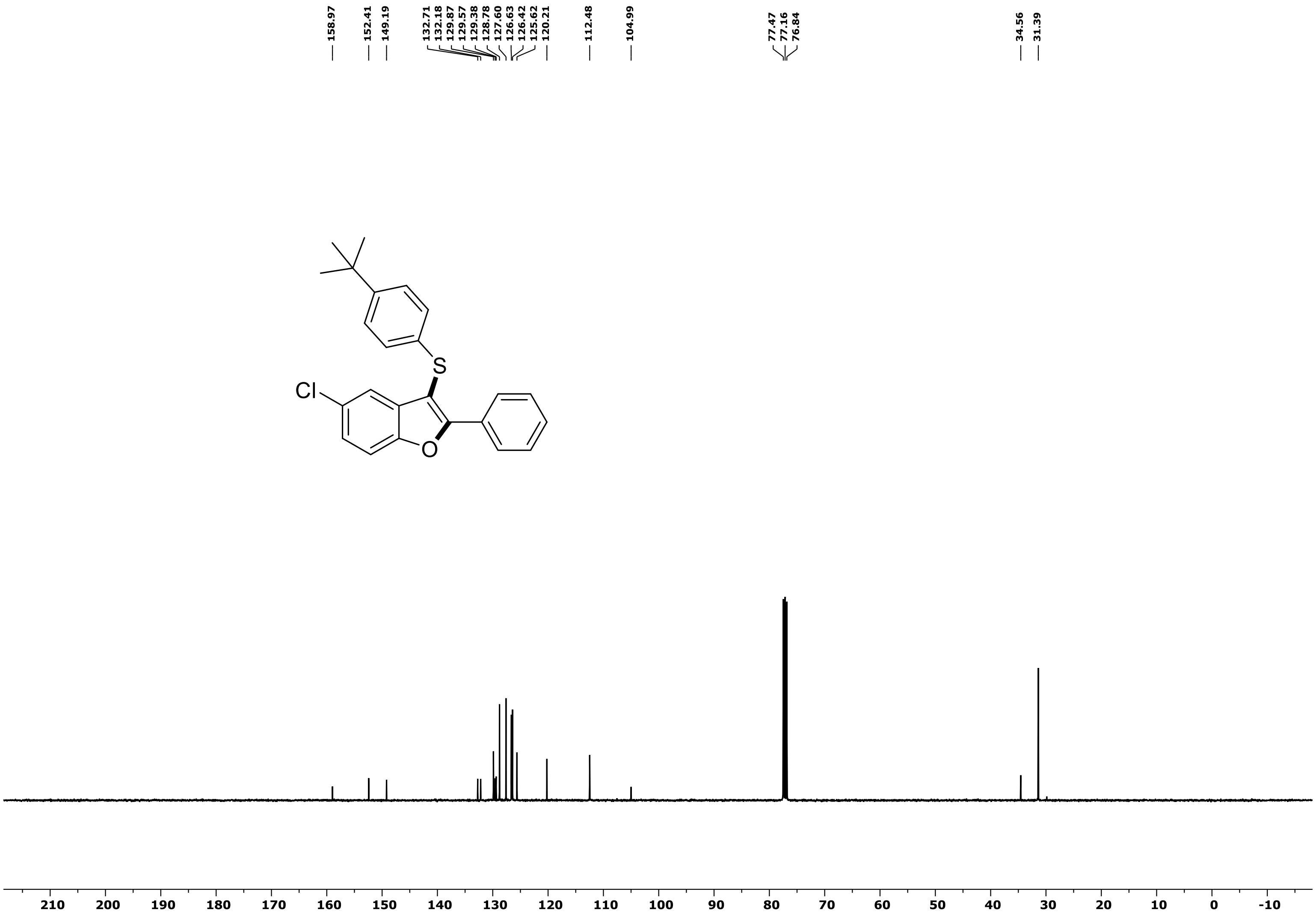

Figure S94: ^{1}H NMR (400 MHz, $CDCl_3$, 298 K) spectrum of *3-((4-(tert-butyl)phenyl)thio)-5-fluoro-2-phenylbenzofuran*, **3na**.

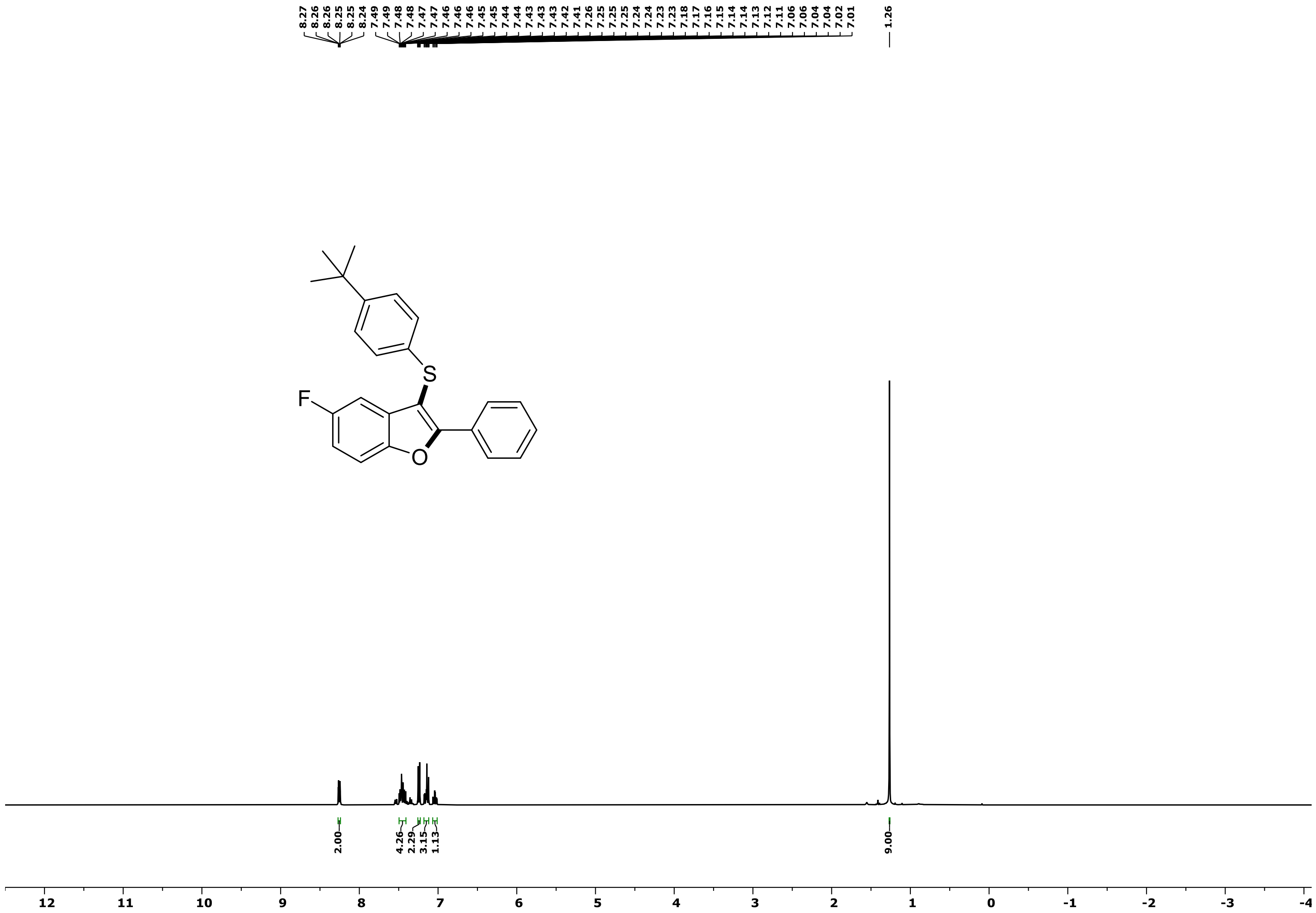

Figure S95: ^{13}C NMR (101 MHz, $CDCl_3$, 298 K) spectrum of *3-((4-(tert-butyl)phenyl)thio)-5-fluoro-2-phenylbenzofuran*, **3na**.

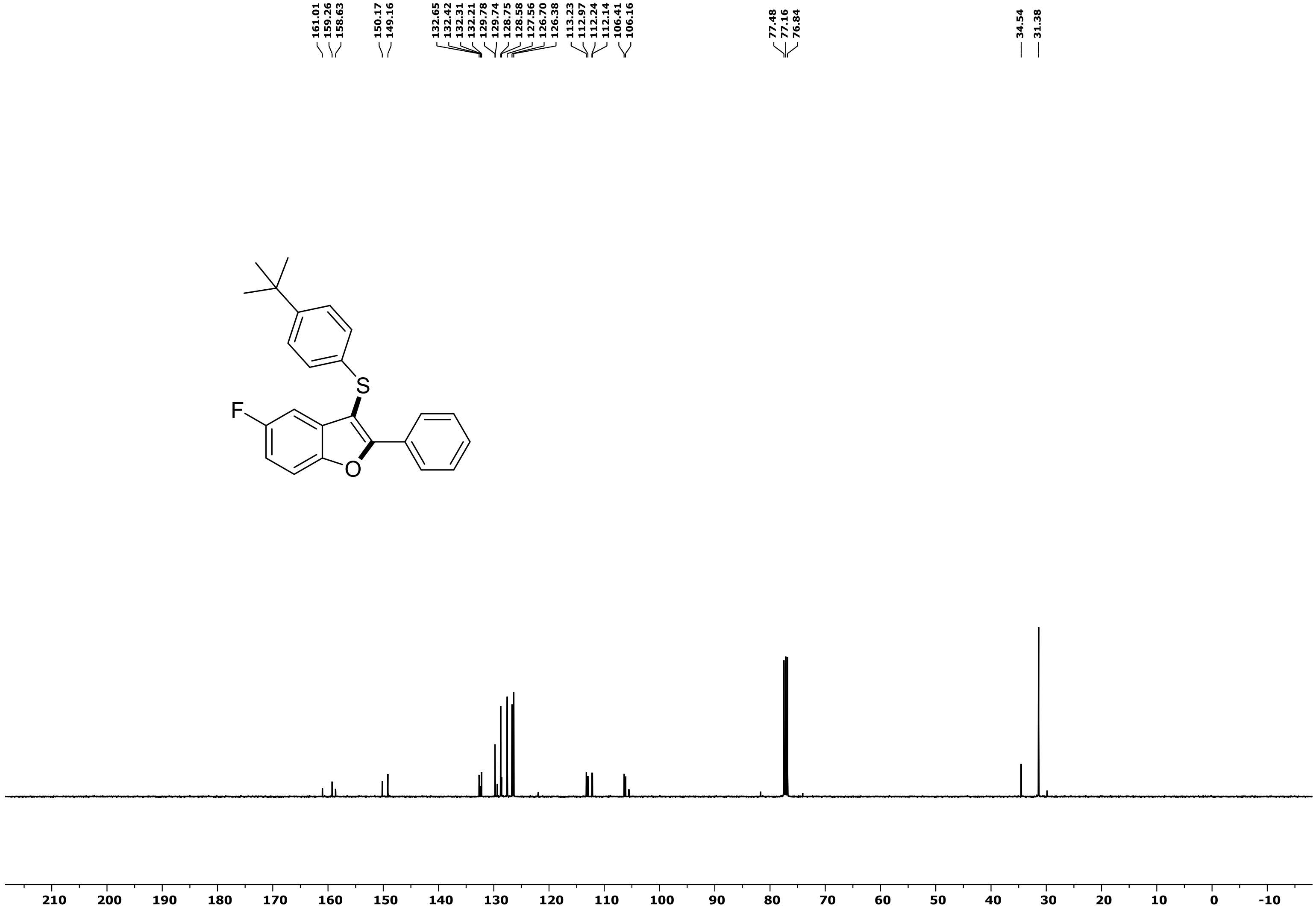

Figure S96: ^{19}F NMR (376 MHz, $CDCl_3$, 298 K) spectrum of *3-((4-(tert-butyl)phenyl)thio)-5-fluoro-2-phenylbenzofuran*, **3na**.

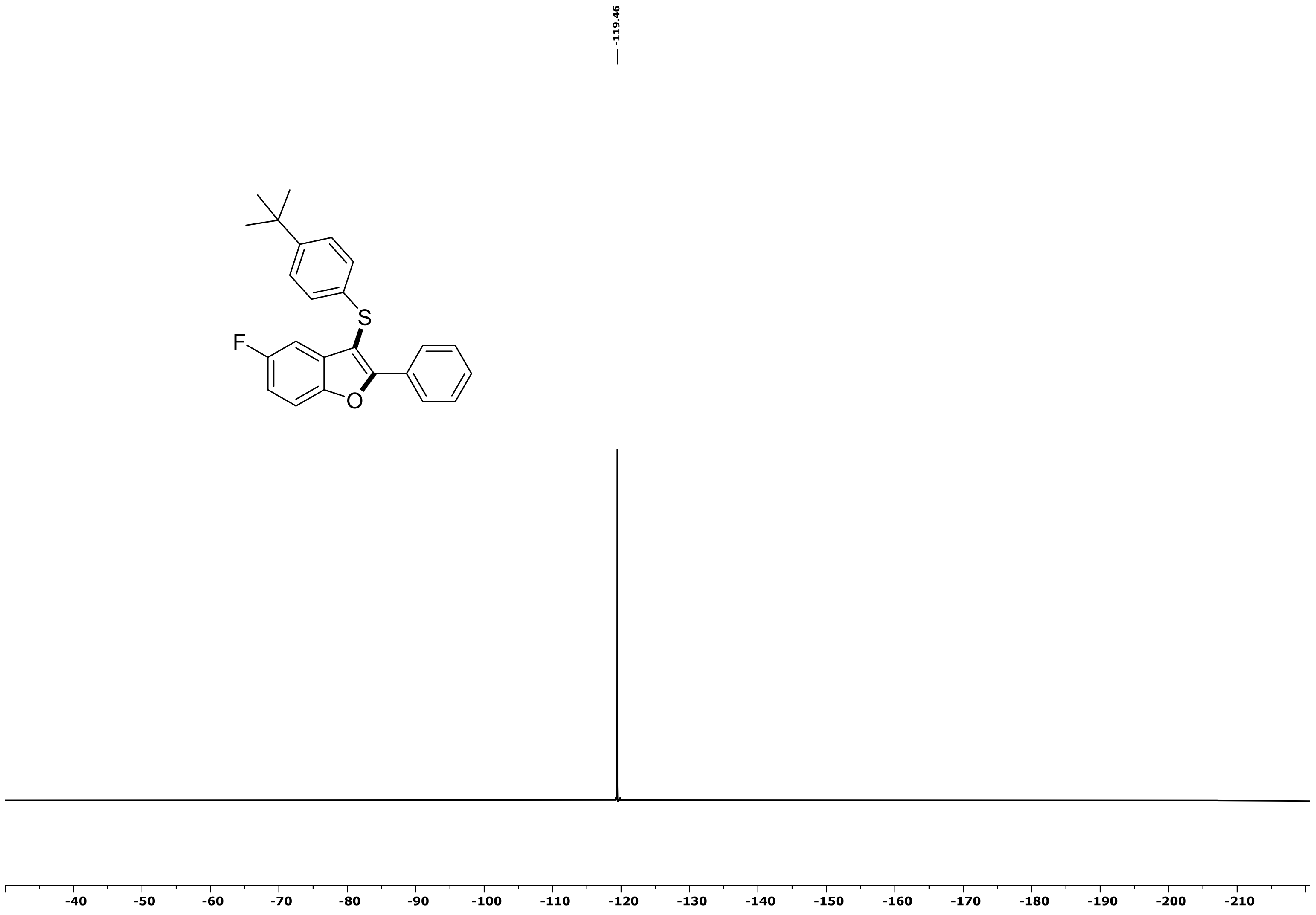

Figure S97: ^{1}H NMR (400 MHz, $CDCl_3$, 298 K) spectrum of *3-((4-(tert-butyl)phenyl)thio)-2-(thiophen-3-yl)benzofuran*, **3oa**.

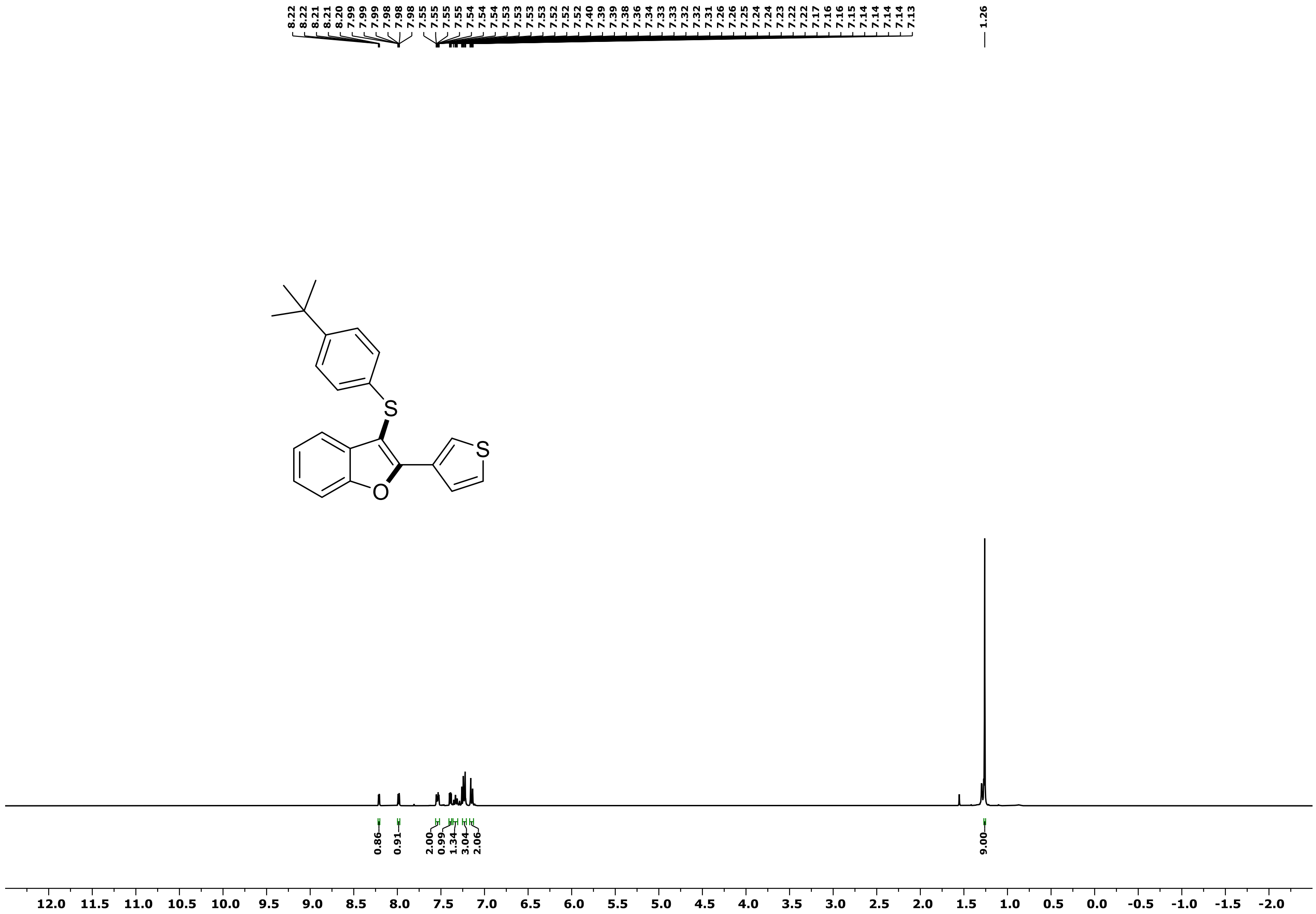

Figure S98: ^{13}C NMR (101 MHz, $CDCl_3$, 298 K) spectrum of *3-((4-(tert-butyl)phenyl)thio)-2-(thiophen-3-yl)benzofuran*, **3oa**.

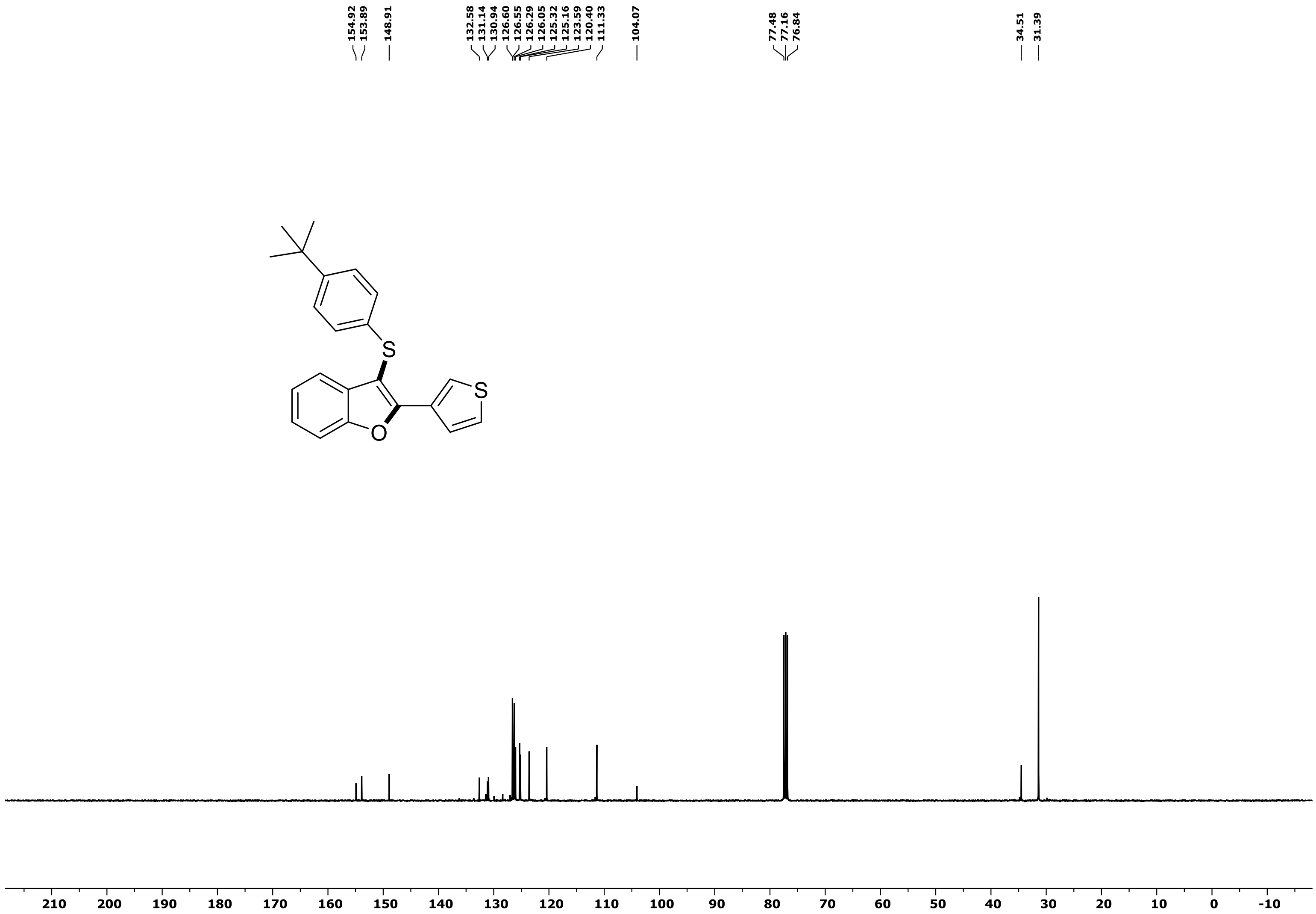

Figure S99: ^{1}H NMR (400 MHz, $CDCl_3$, 298 K) spectrum of *3-((4-(tert-butyl)phenyl)thio)-2-cyclopropylbenzofuran*, **3pa**.

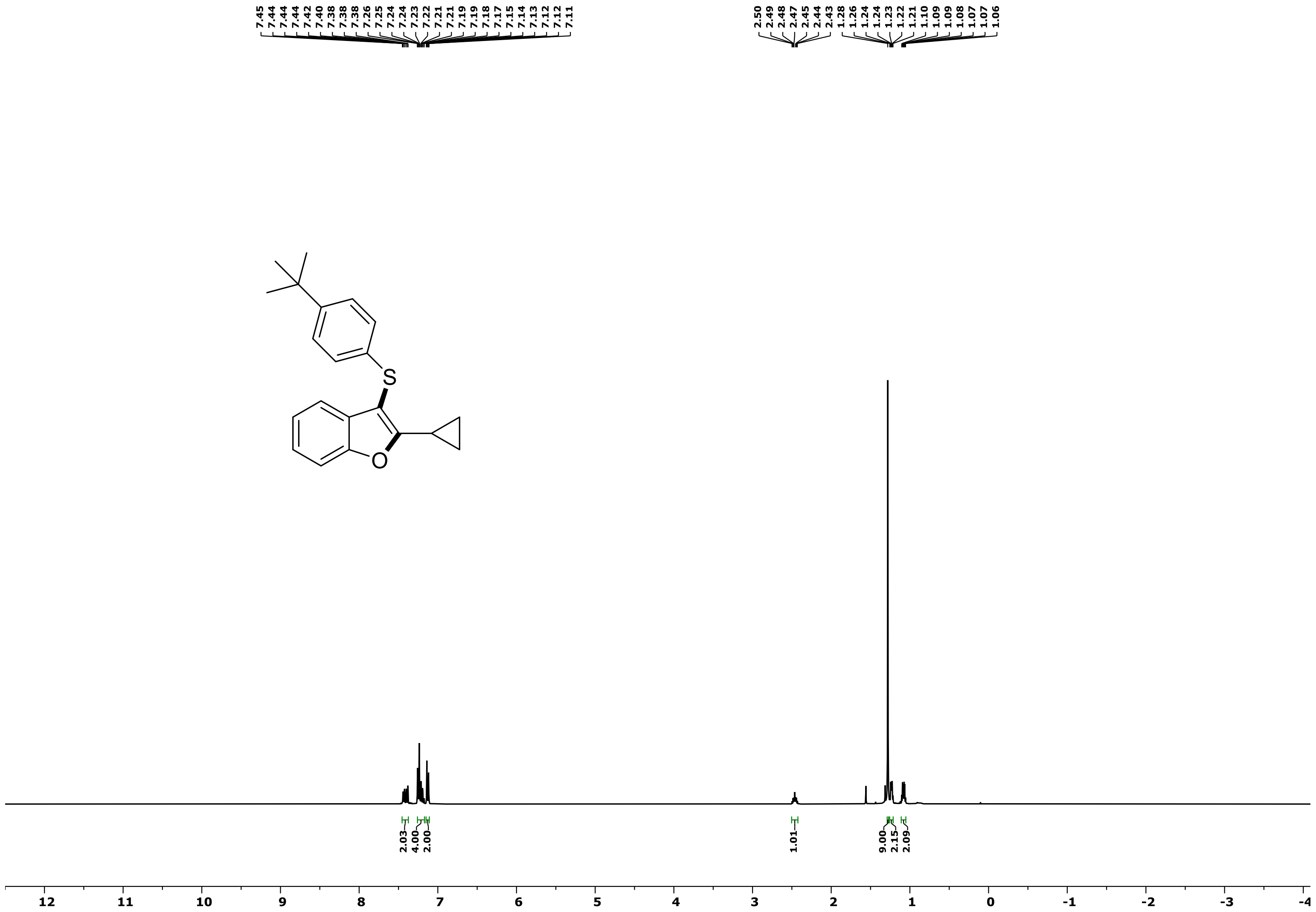

Figure S100: ^{13}C NMR (101 MHz, $CDCl_3$, 298 K) spectrum of *3-((4-(tert-butyl)phenyl)thio)-2-cyclopropylbenzofuran*, **3pa**.

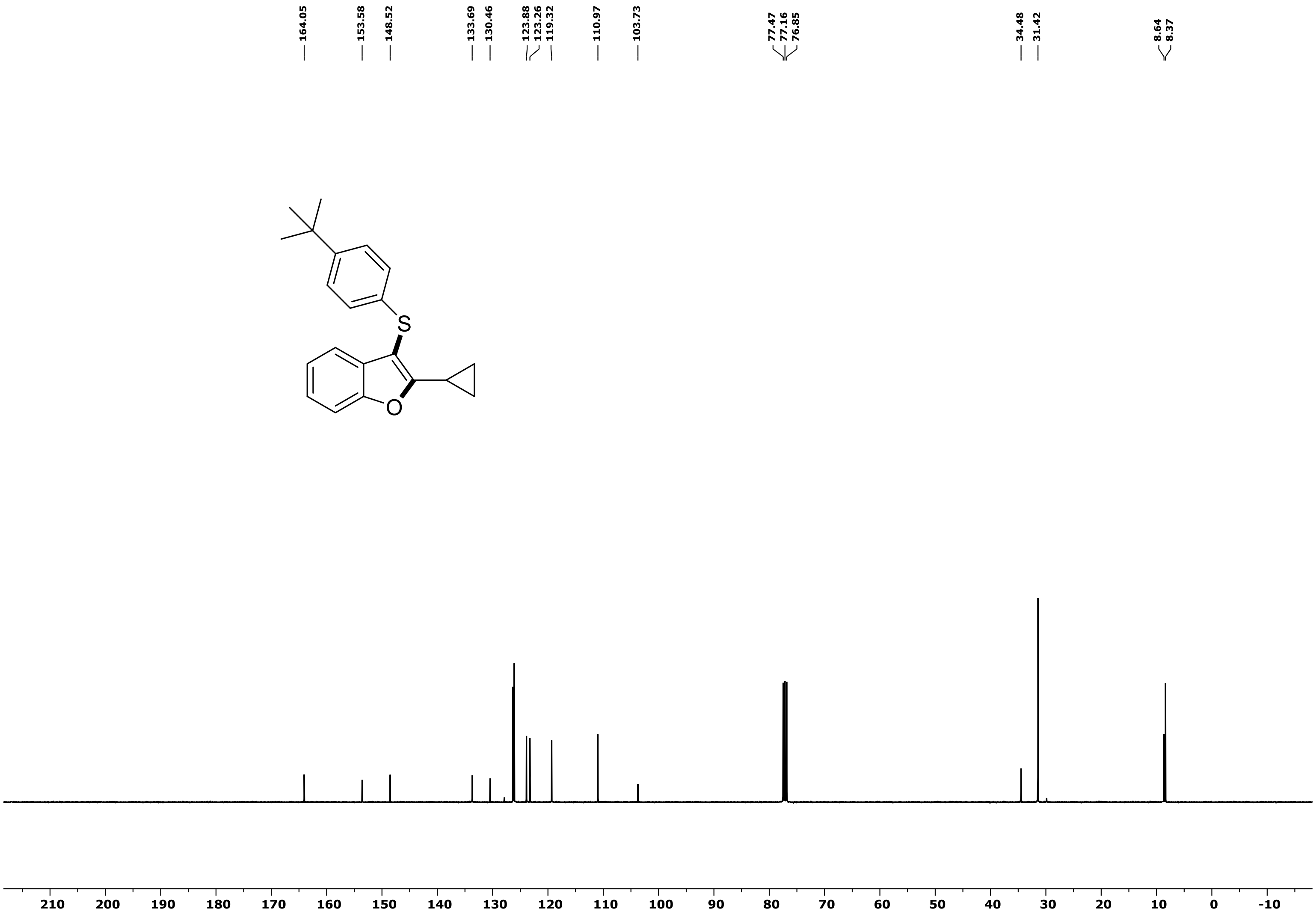

Figure S101: ^{1}H NMR (400 MHz, $CDCl_3$, 298 K) spectrum of *2-phenyl-3-(phenylthio)-1-tosyl-1H-indole*, **3ab**.

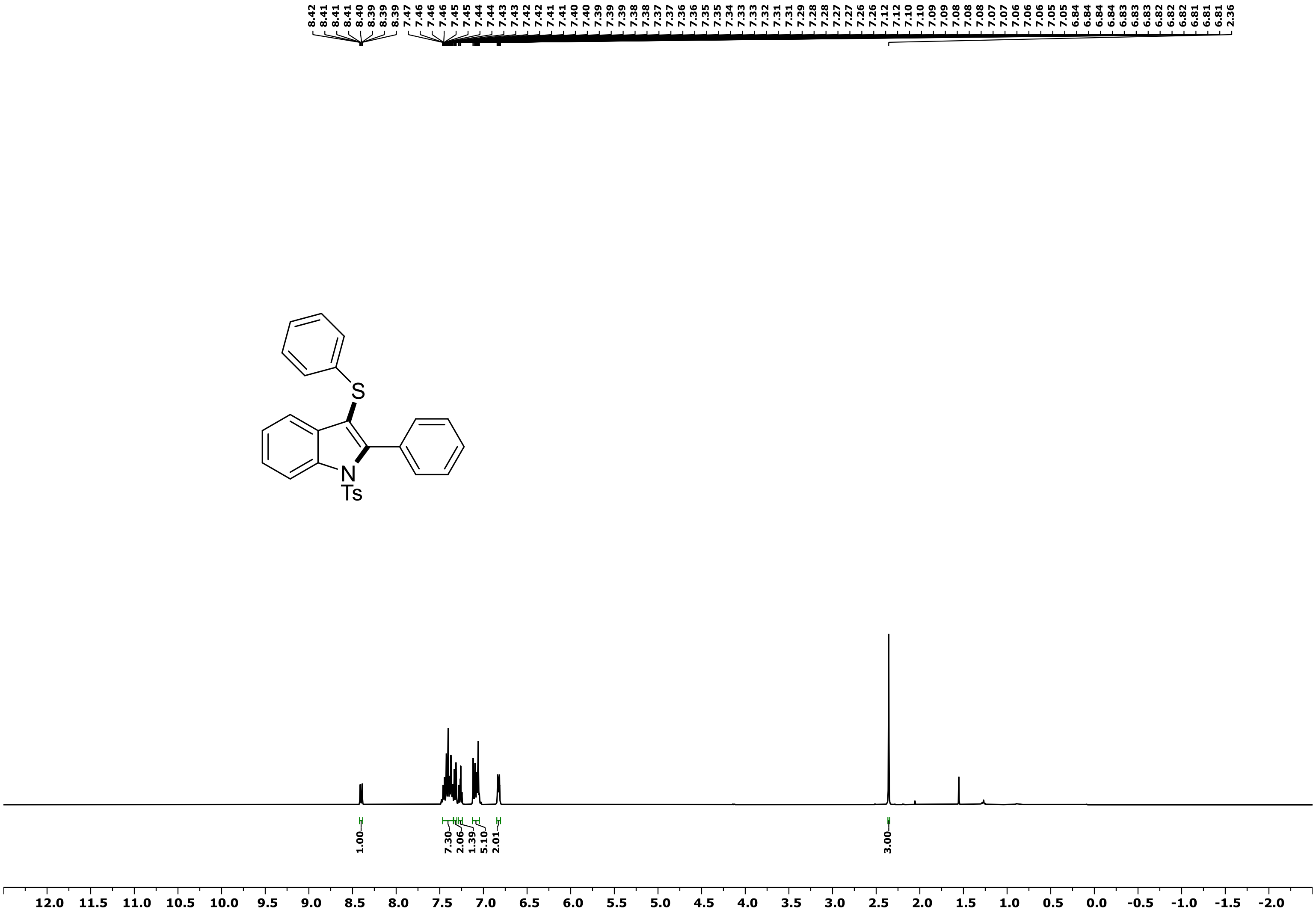

Figure S102: ^{13}C NMR (101 MHz, $CDCl_3$, 298 K) spectrum of *2-phenyl-3-(phenylthio)-1-tosyl-1H-indole*, **3ab**.

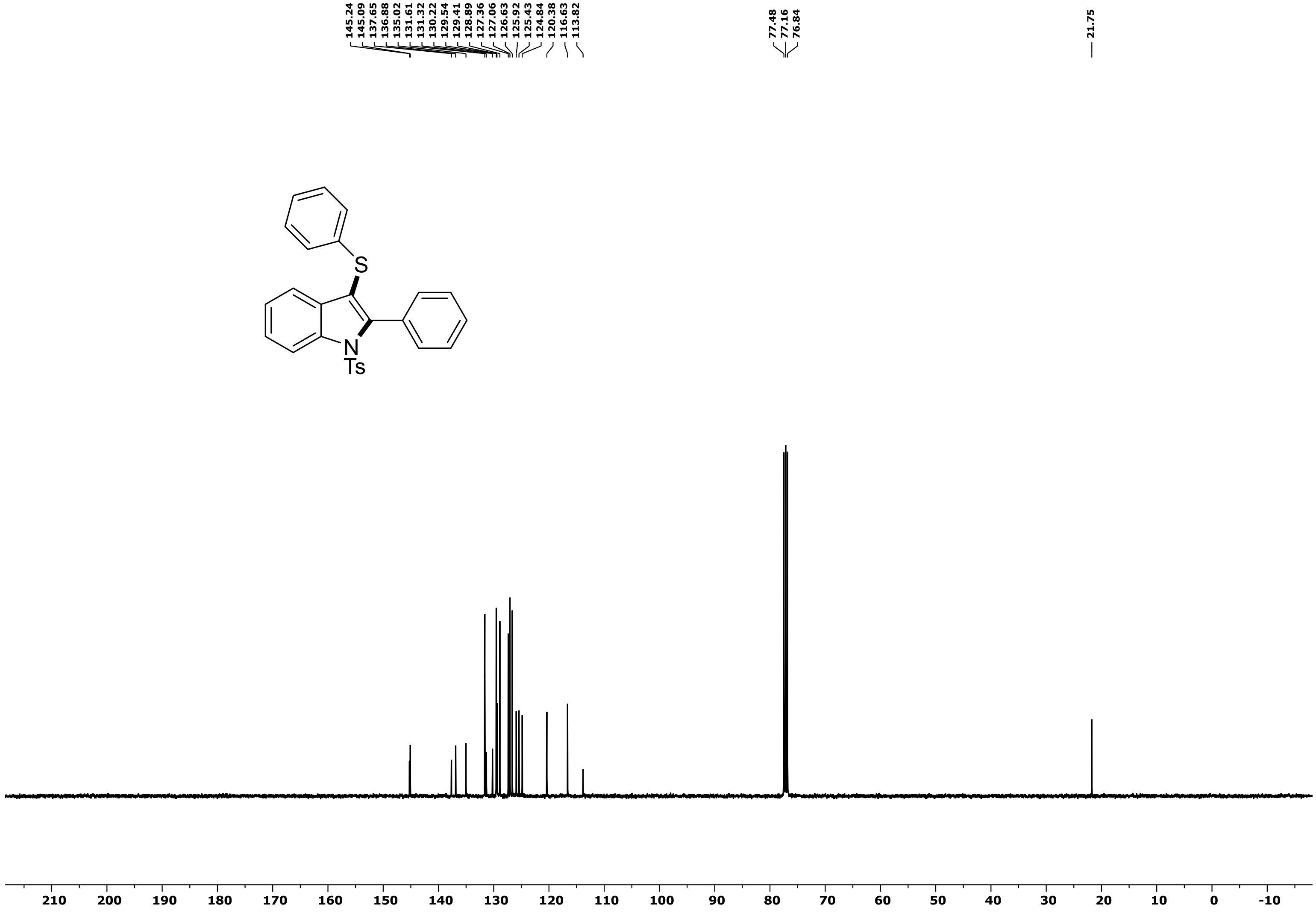

Figure S103: ^{1}H NMR (400 MHz, $CDCl_3$, 298 K) spectrum of *2-phenyl-3-(p-tolylthio)-1-tosyl-1H-indole*, **3ac**.

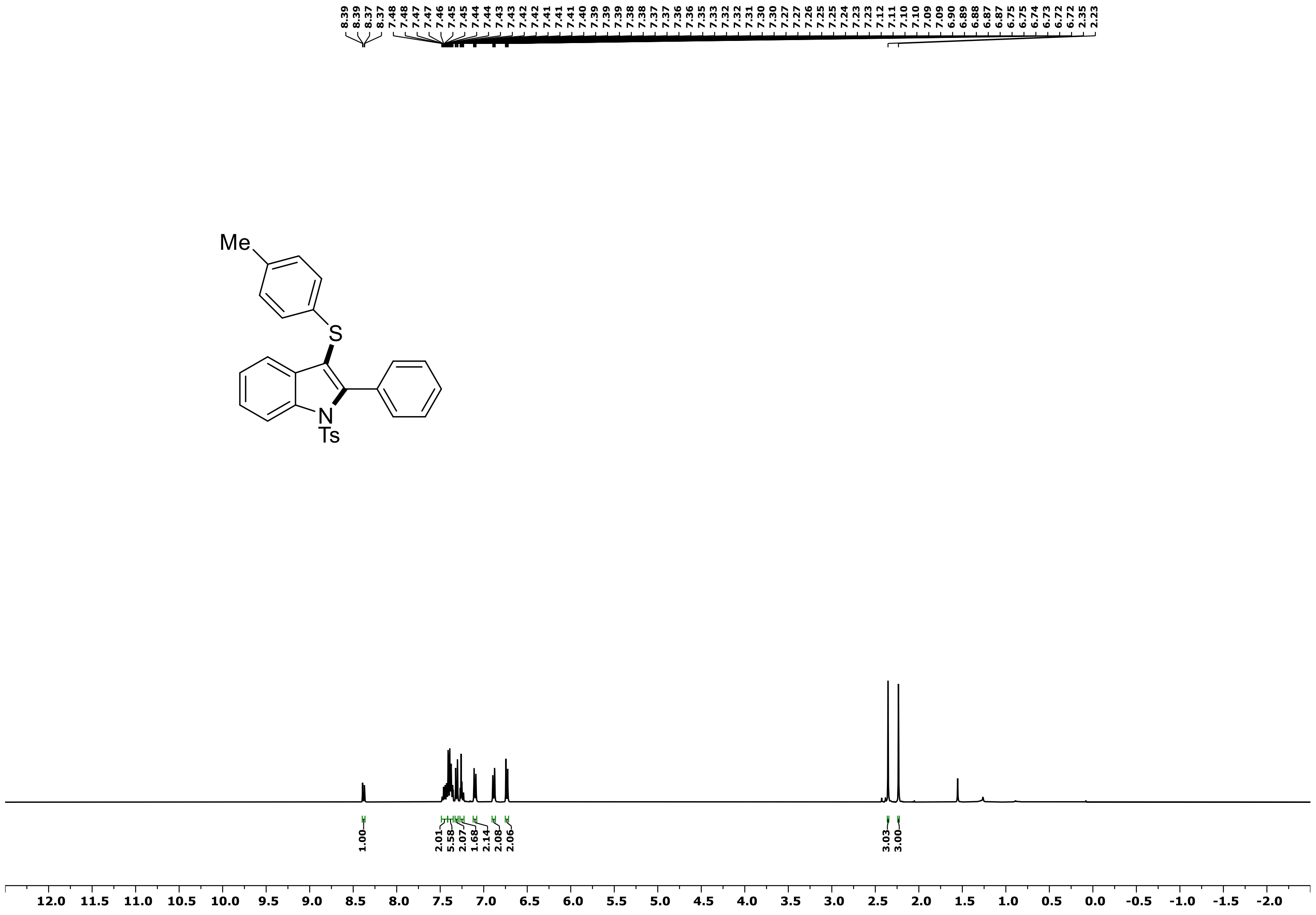

Figure S104: ^{13}C NMR (101 MHz, $CDCl_3$, 298 K) spectrum of *2-phenyl-3-(p-tolylthio)-1-tosyl-1H-indole*, **3ac**.

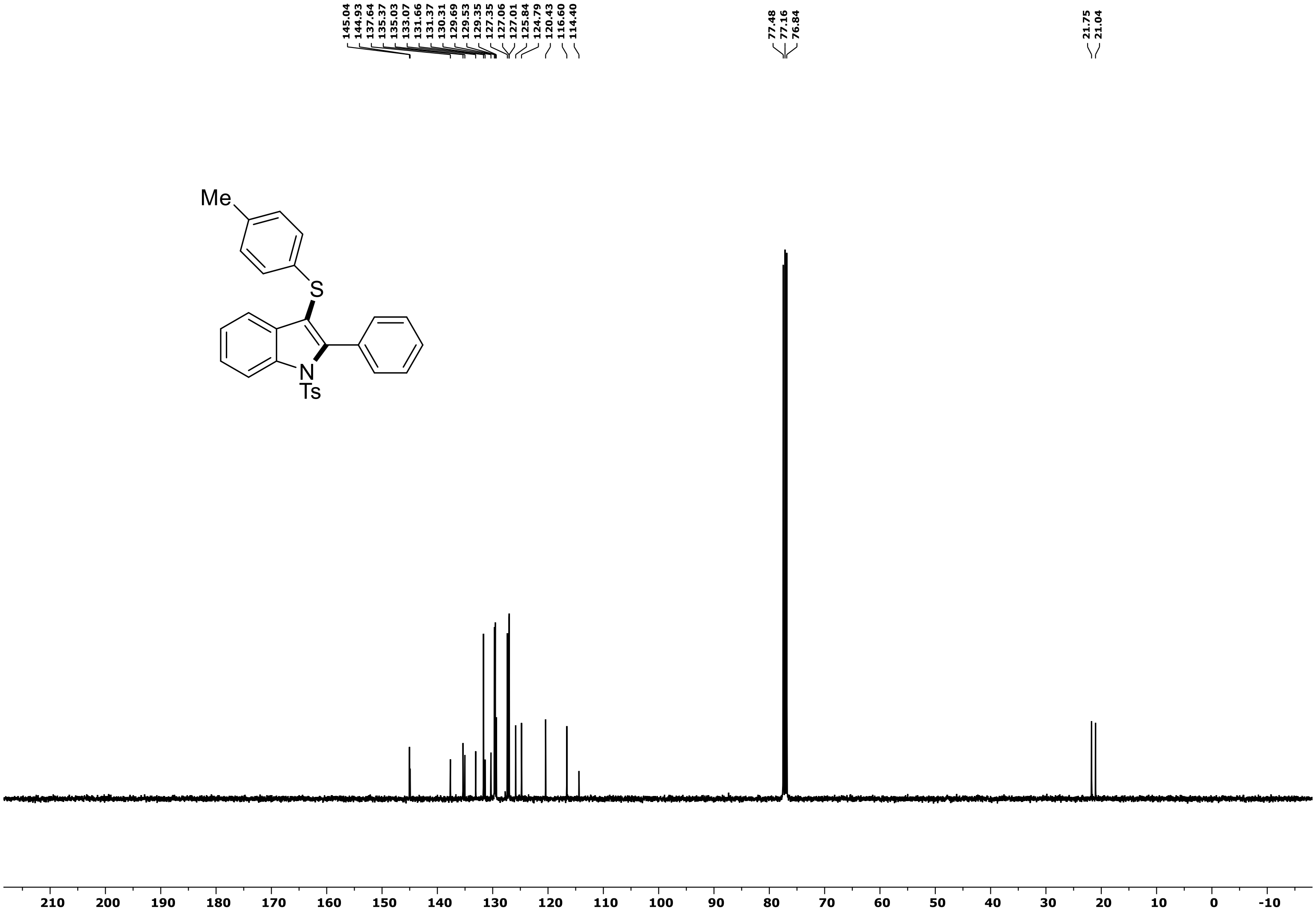

Figure S105: ^{1}H NMR (400 MHz, $CDCl_3$, 298 K) spectrum of *3-((2-bromophenyl)thio)-2-phenyl-1-tosyl-1H-indole*, **3ad**.

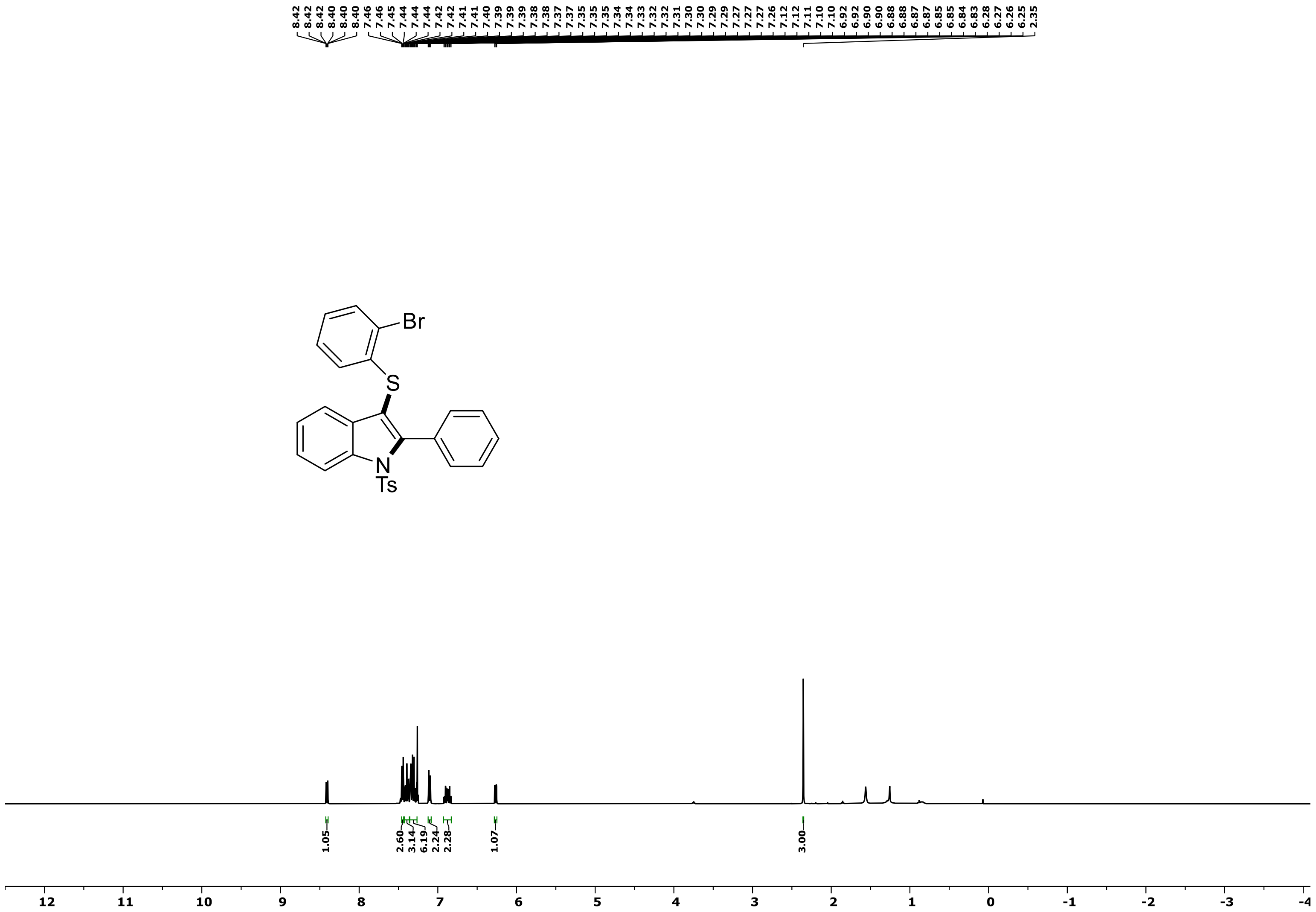

Figure S106: ^{13}C NMR (101 MHz, $CDCl_3$, 298 K) spectrum of *3-((2-bromophenyl)thio)-2-phenyl-1-tosyl-1H-indole*, **3ad**.

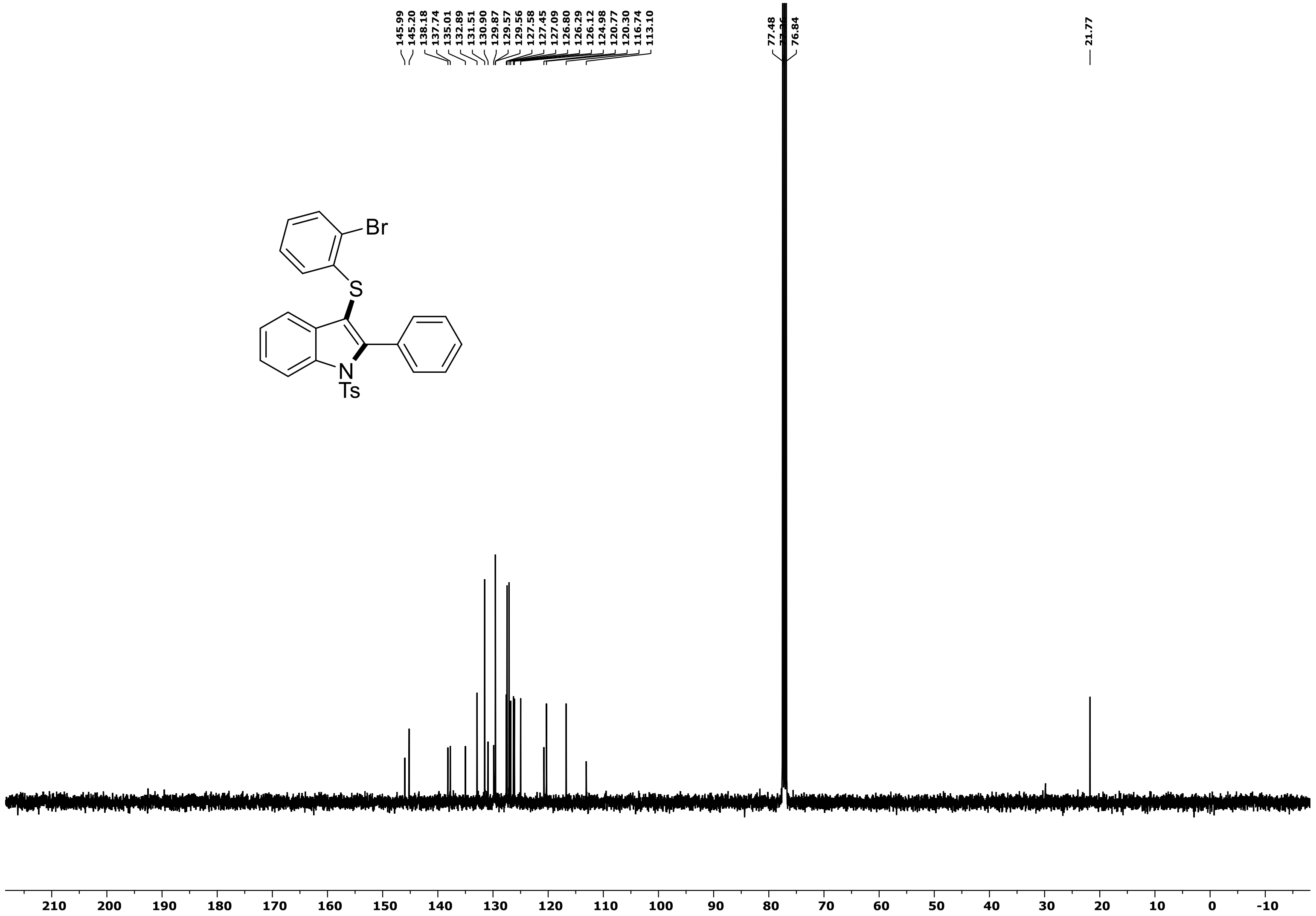

Figure S107: ^{1}H NMR (400 MHz, $CDCl_3$, 298 K) spectrum of *3-((4-fluorophenyl)thio)-2-phenyl-1-tosyl-1H-indole*, **3ae**.

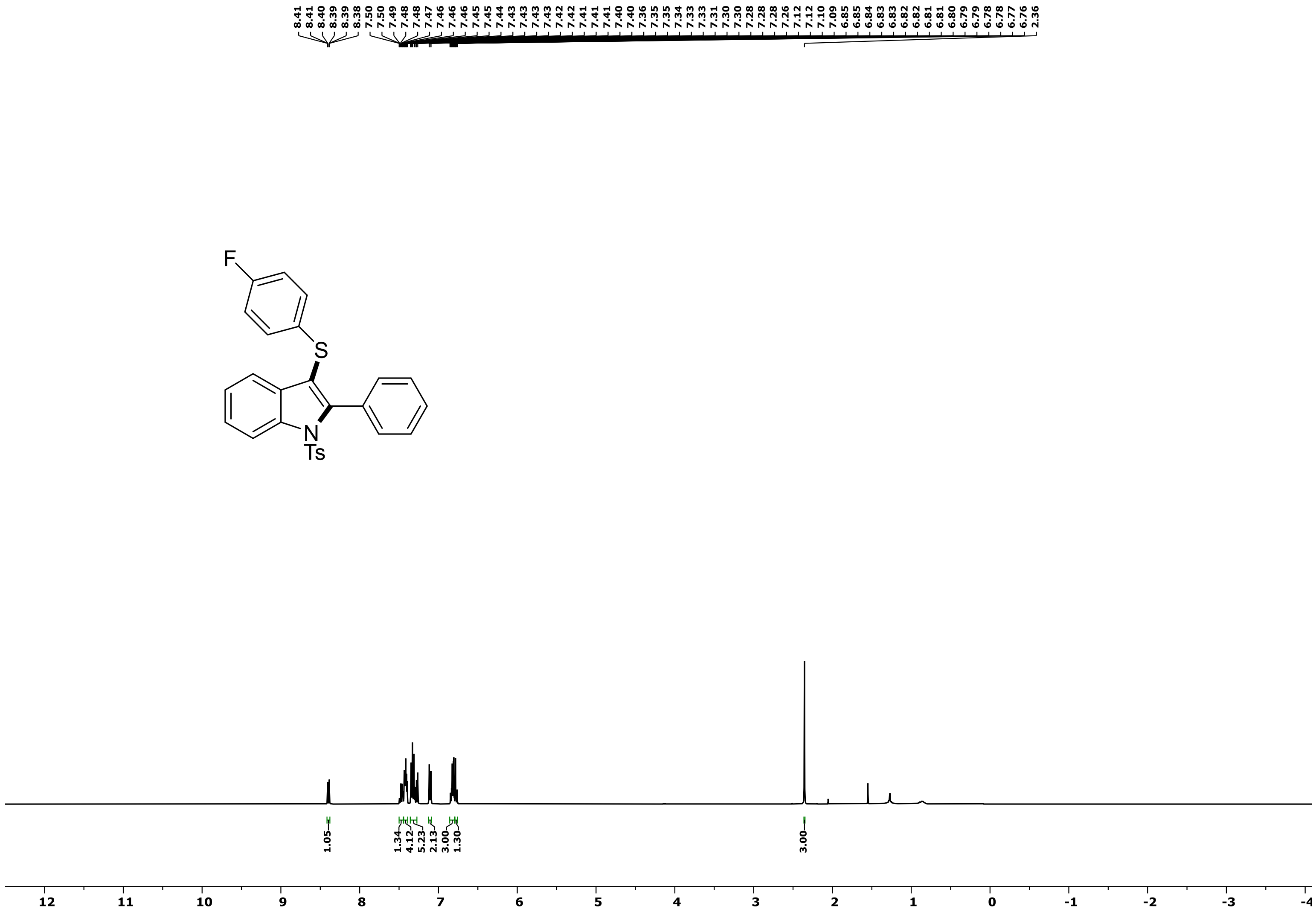

Figure S108: ^{13}C NMR (101 MHz, $CDCl_3$, 298 K) spectrum of *3-((4-fluorophenyl)thio)-2-phenyl-1-tosyl-1H-indole*, **3ae**.

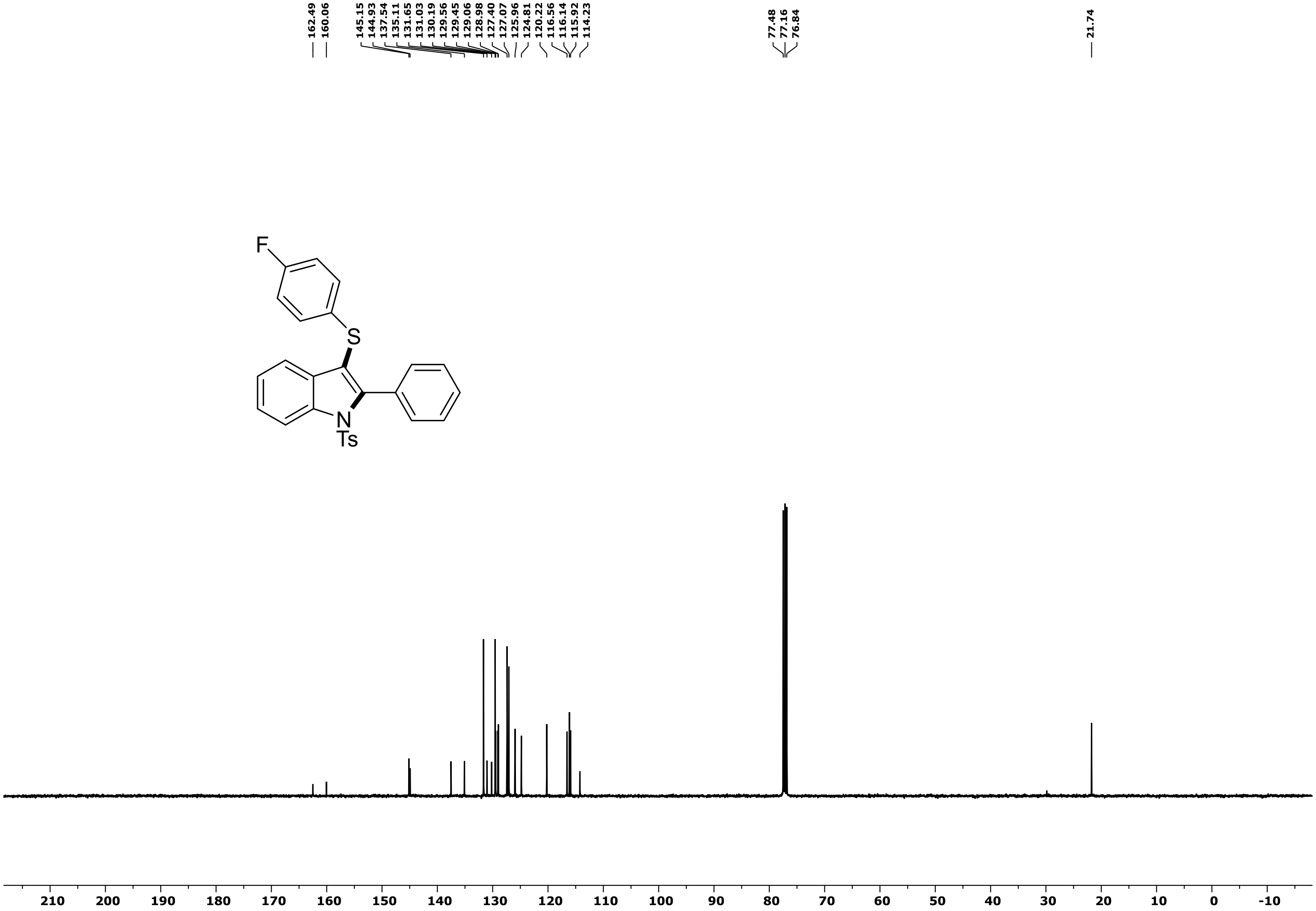

Figure S109: ^{19}F NMR (376 MHz, $CDCl_3$, 298 K) spectrum of *3-((4-fluorophenyl)thio)-2-phenyl-1-tosyl-1H-indole*, **3ae**.

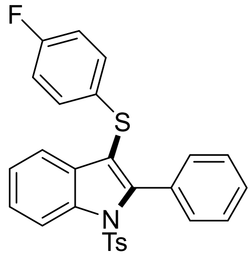

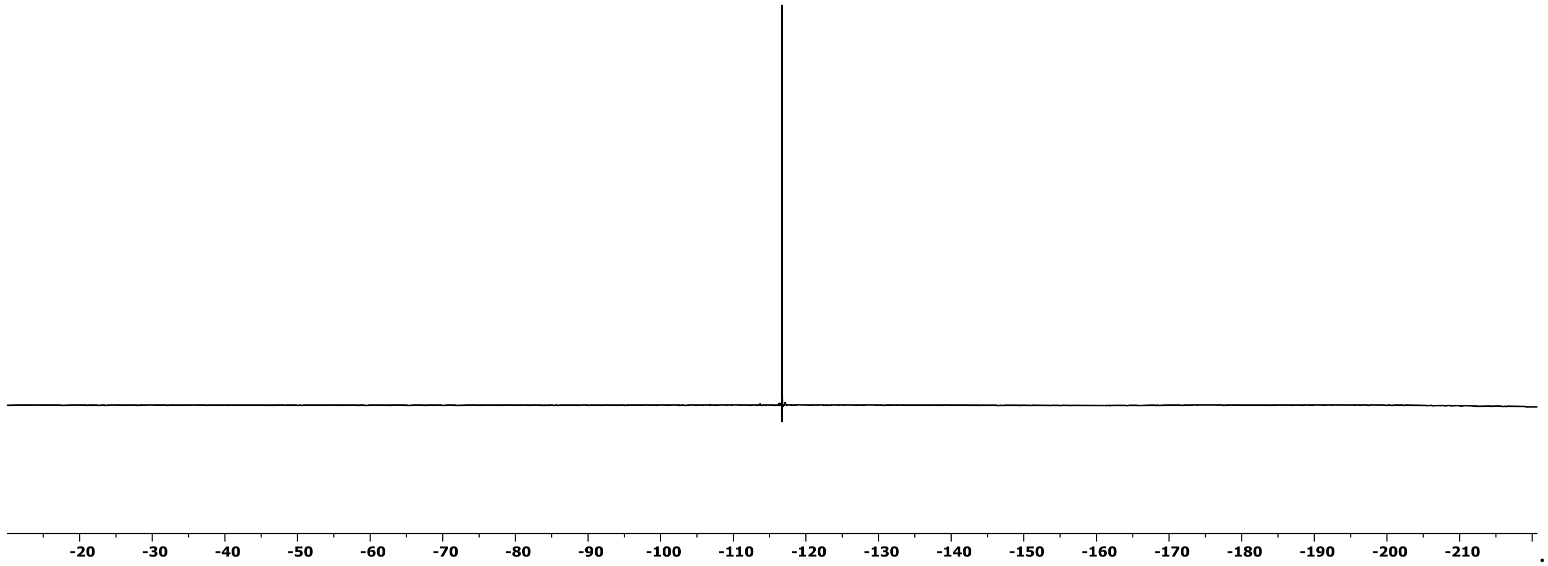

Figure S110: ^{1}H NMR (400 MHz, $CDCl_3$, 298 K) spectrum of *3-((4-chlorophenyl)thio)-2-phenyl-1-tosyl-1H-indole*, **3af**.

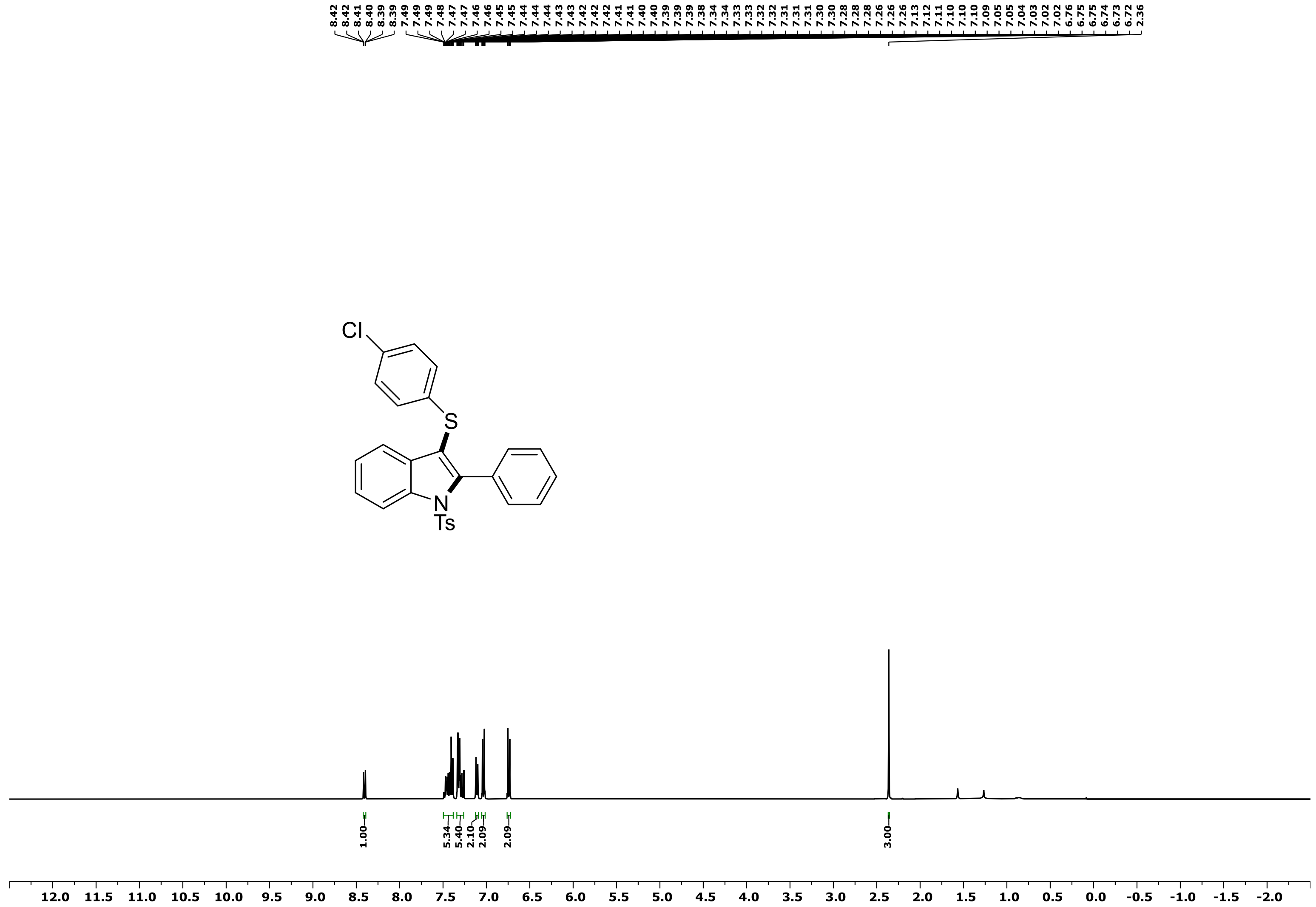

Figure S111: ^{13}C NMR (101 MHz, $CDCl_3$, 298 K) spectrum of *3-((4-chlorophenyl)thio)-2-phenyl-1-tosyl-1H-indole*, **3af**.

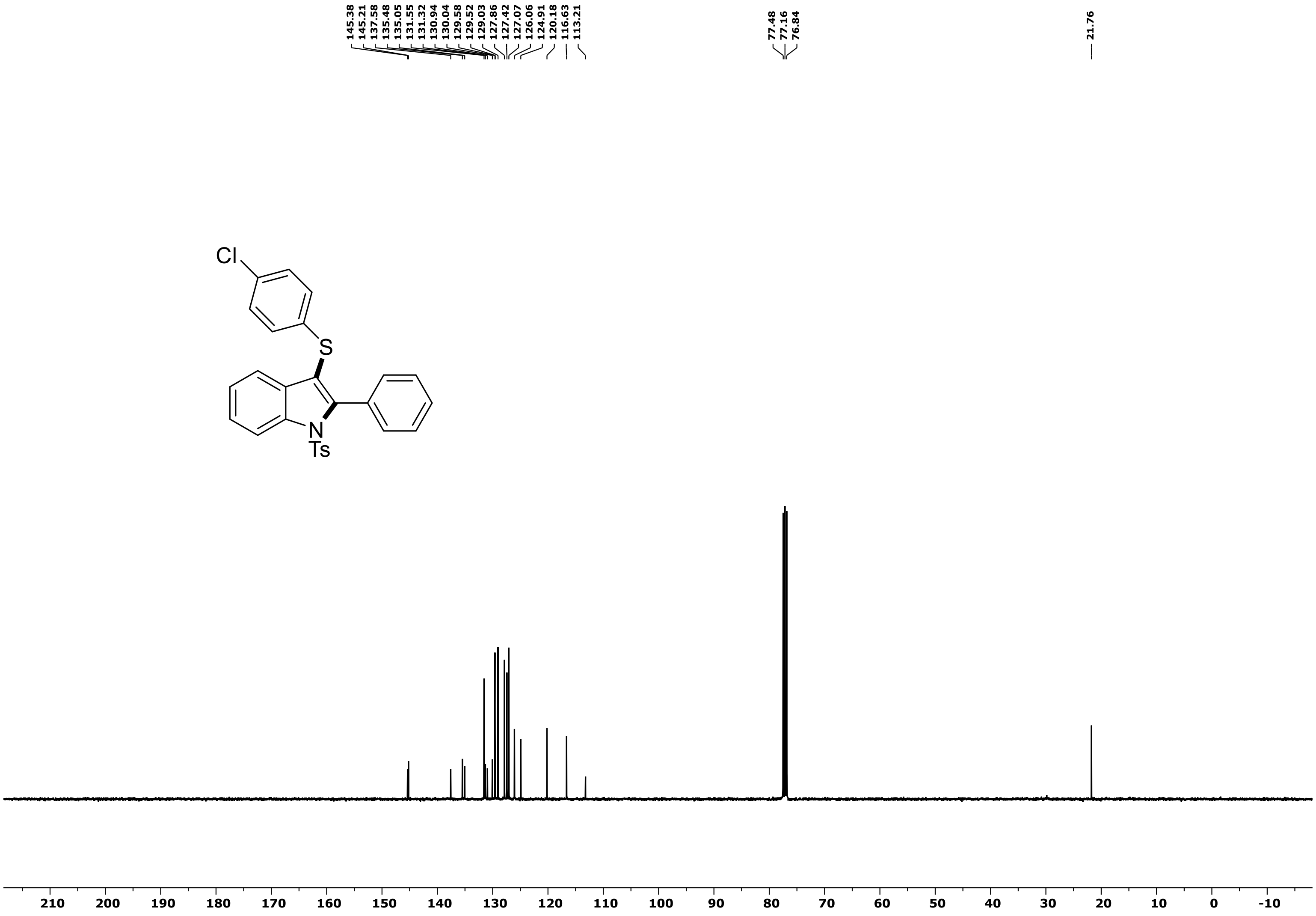

Figure S112: ^{1}H NMR (400 MHz, $CDCl_3$, 298 K) spectrum of *3-((2,4-difluorophenyl)thio)-2-phenyl-1-tosyl-1H-indole*, **3ag**.

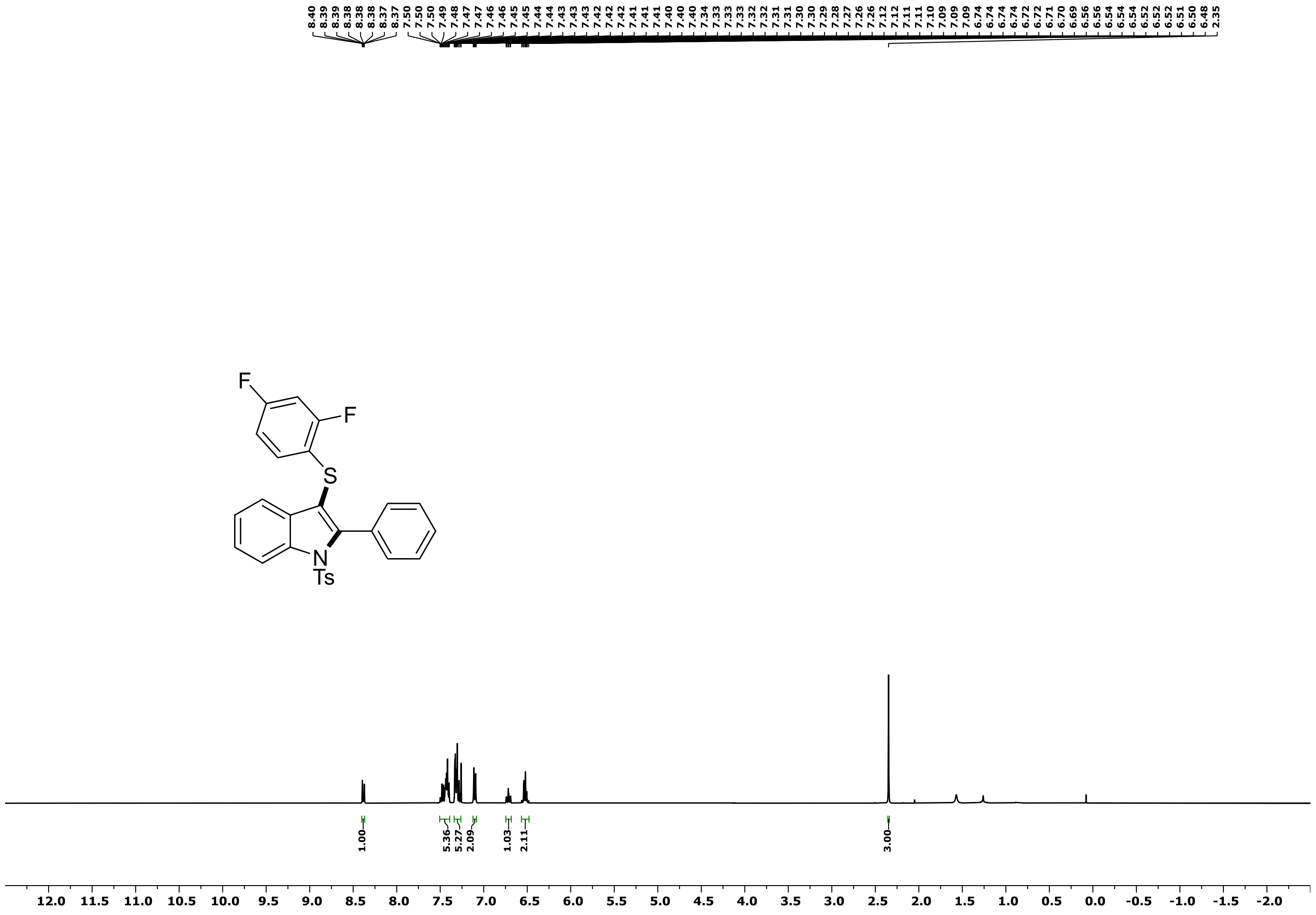

Figure S113: ^{13}C NMR (101 MHz, $CDCl_3$, 298 K) spectrum of *3-((2,4-difluorophenyl)thio)-2-phenyl-1-tosyl-1H-indole*, **3ag**.

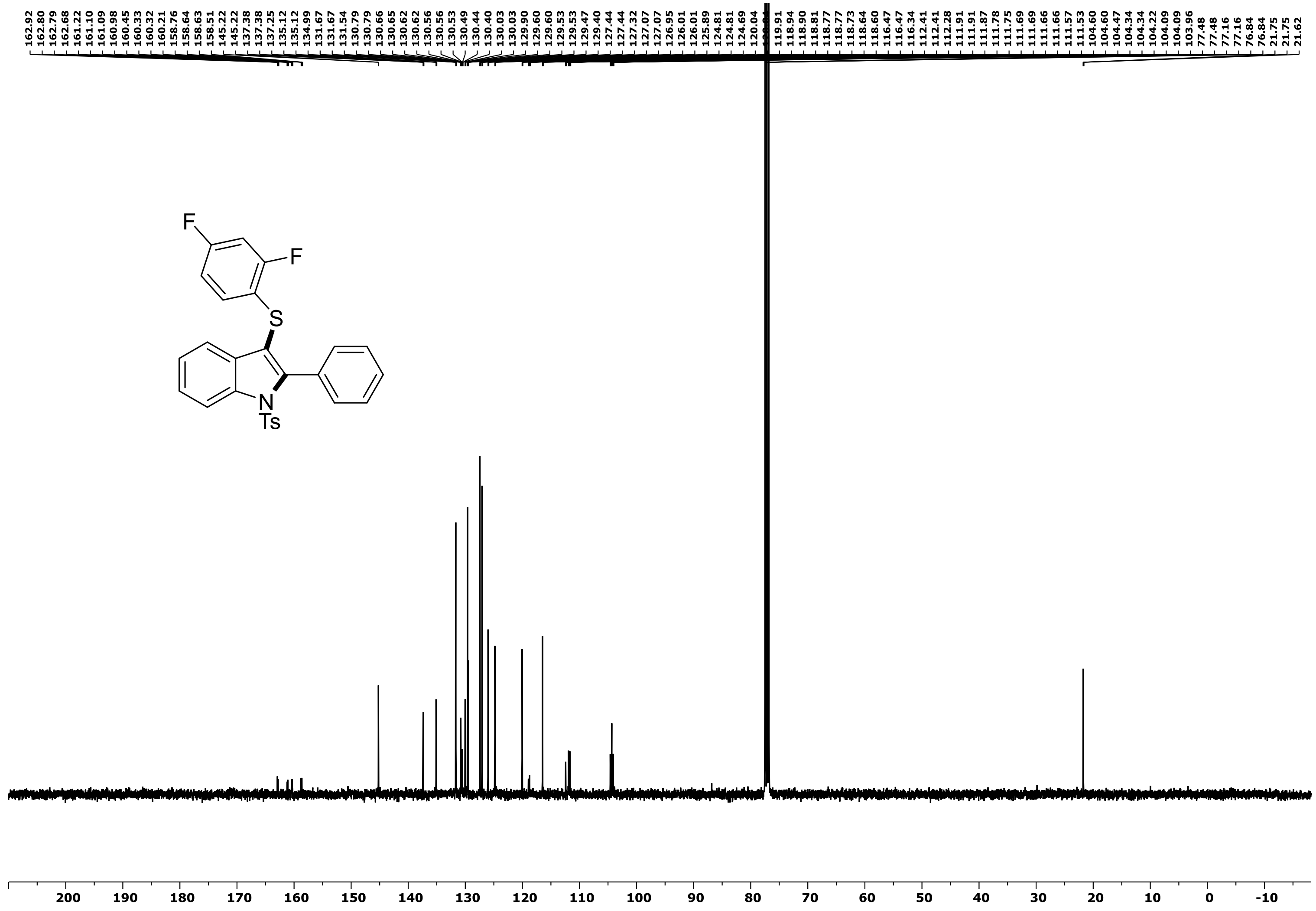

Figure S114: ^{19}F NMR (376 MHz, $CDCl_3$, 298 K) spectrum of *3-((2,4-difluorophenyl)thio)-2-phenyl-1-tosyl-1H-indole,* **3ag**

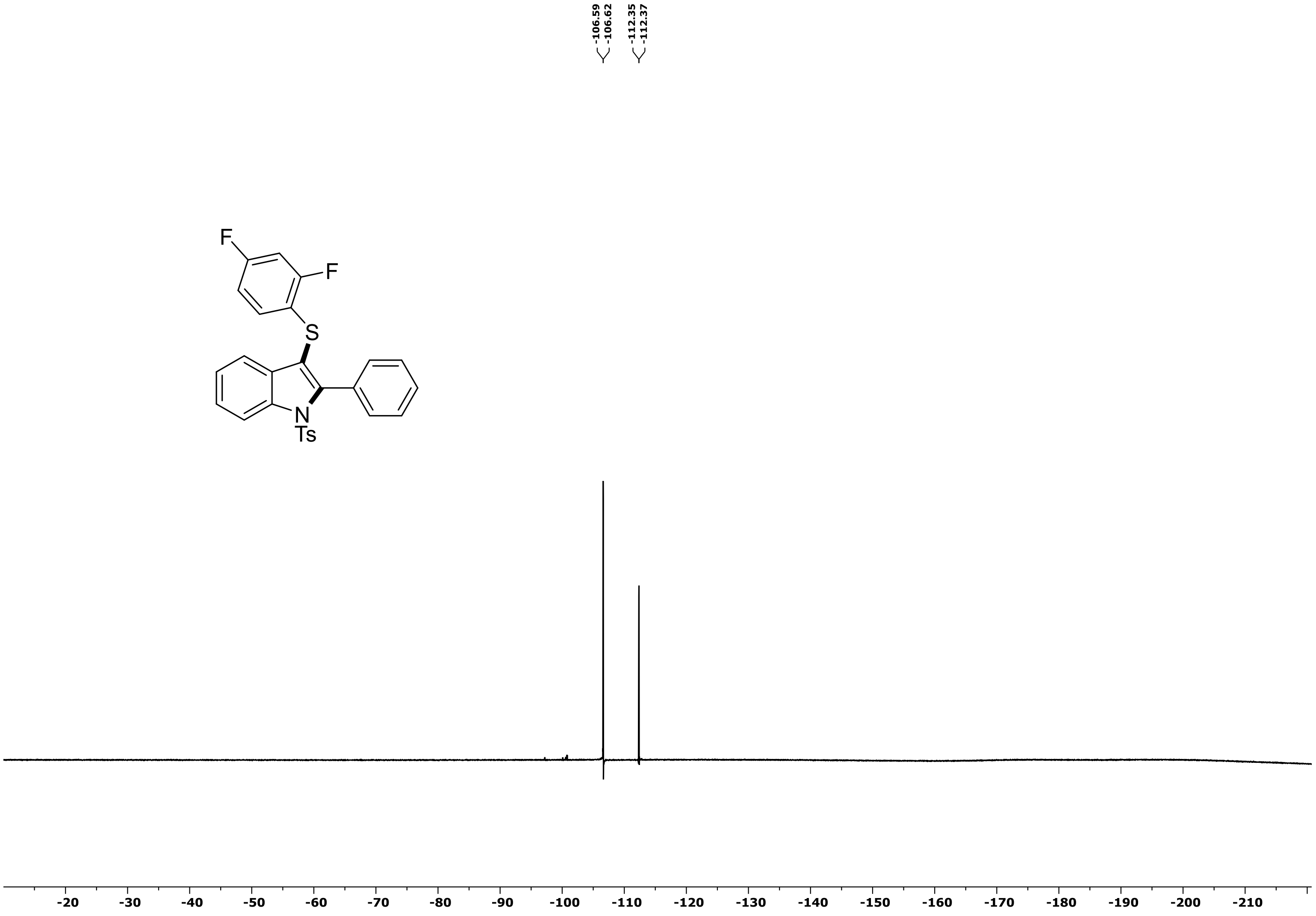

Figure S115: ^{1}H NMR (400 MHz, $CDCl_3$, 298 K) spectrum of *2-phenyl-1-tosyl-3-((4-(trifluoromethyl)phenyl)thio)-1H-indole*, **3ah**.

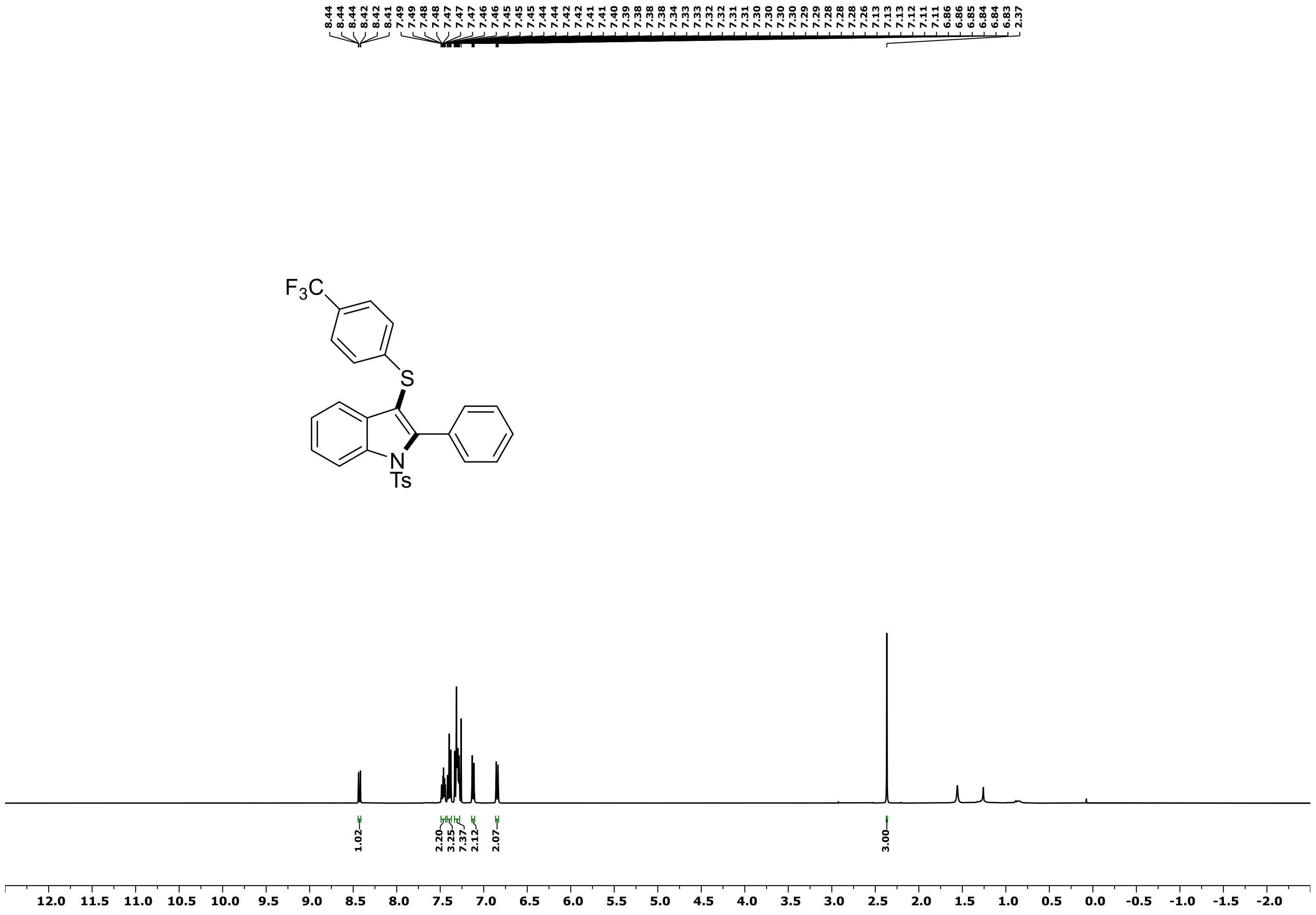

Figure S116: ^{13}C NMR (101 MHz, $CDCl_3$, 298 K) spectrum of *2-phenyl-1-tosyl-3-((4-(trifluoromethyl)phenyl)thio)-1H-indole*, **3ah**.

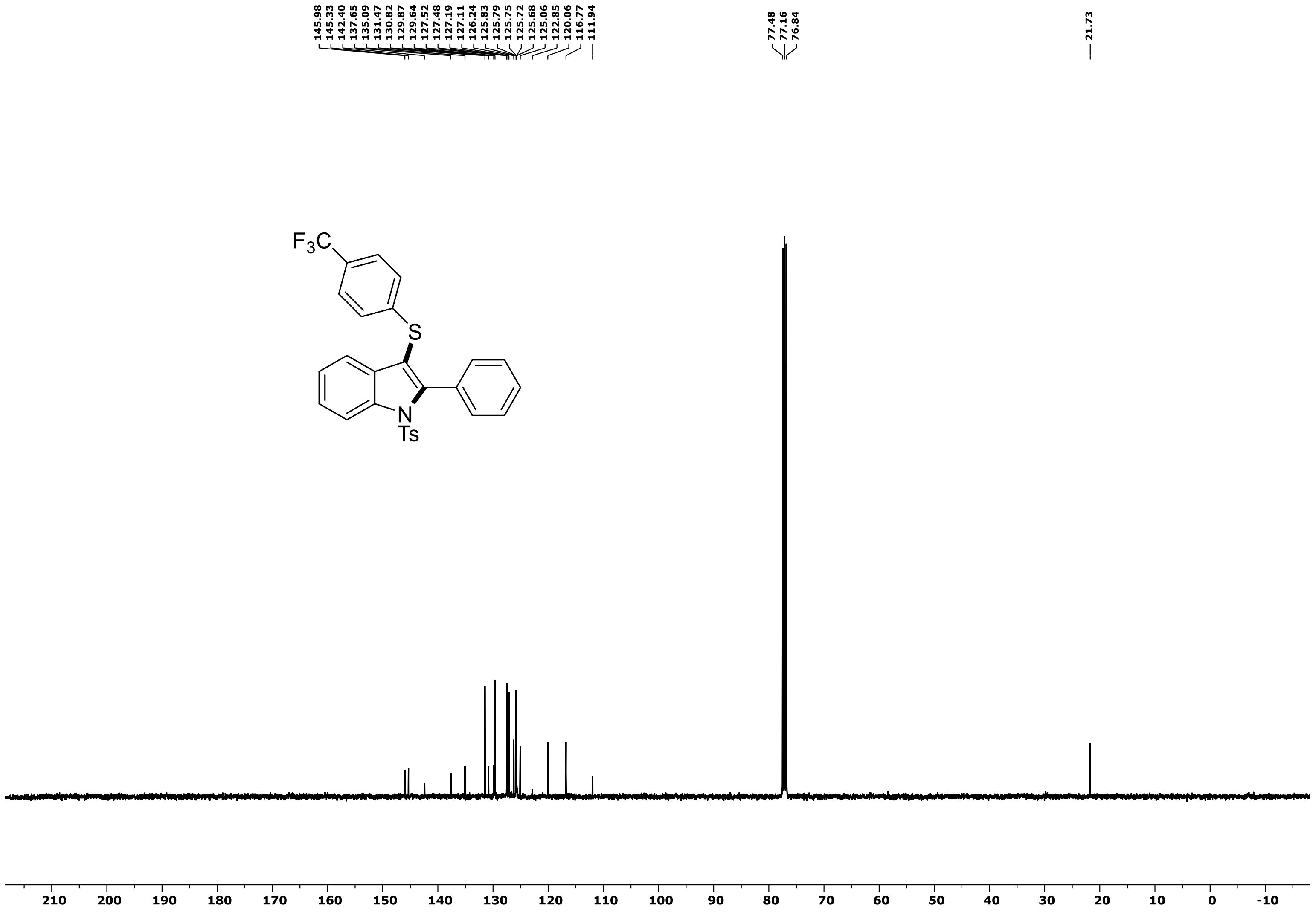

Figure S117: ^{19}F NMR (376 MHz, $CDCl_3$, 298 K) spectrum of *2-phenyl-1-tosyl-3-((4-(trifluoromethyl)phenyl)thio)-1H-indole*, **3ah**.

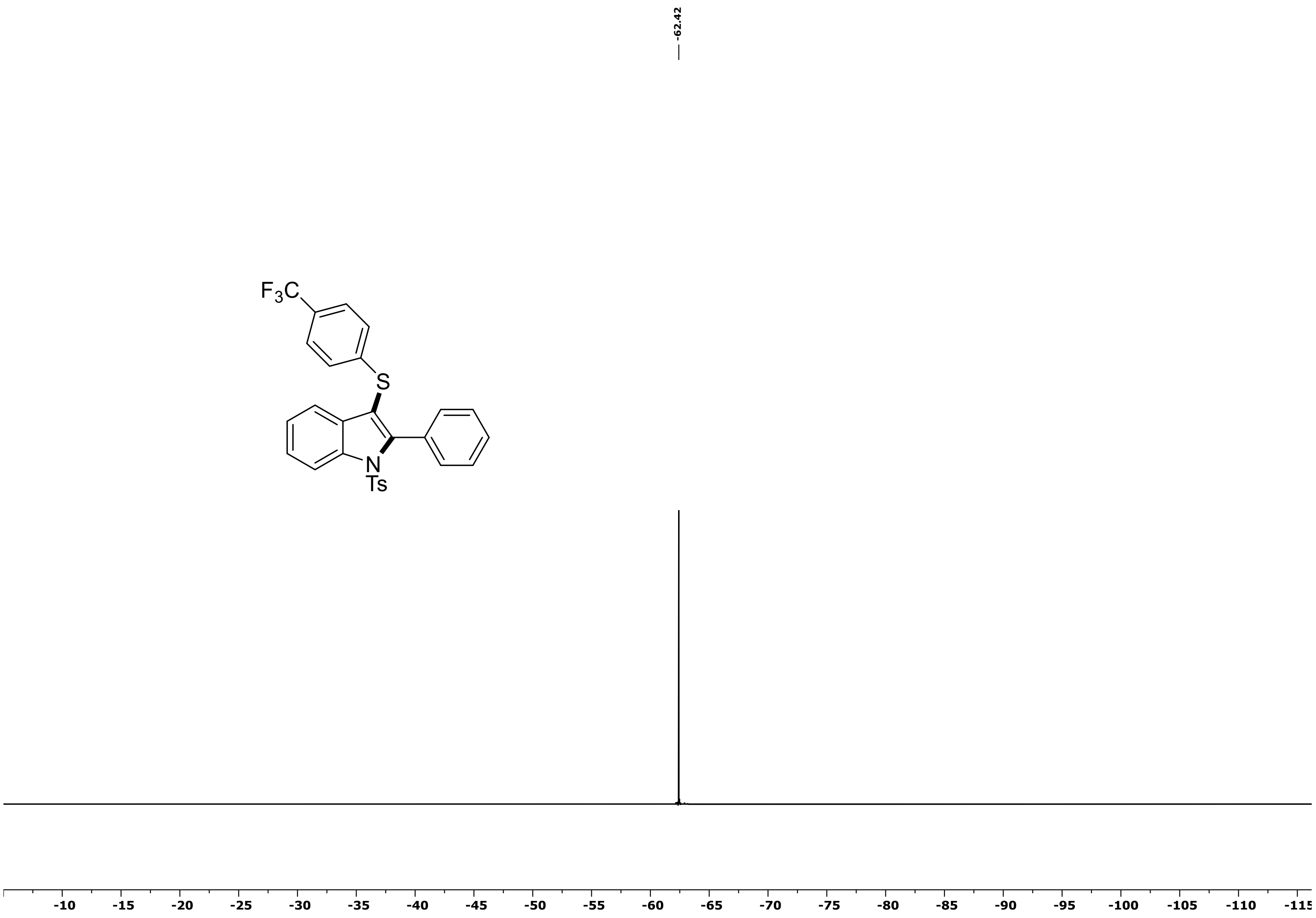

Figure S118: ^{1}H NMR (400 MHz, $CDCl_3$, 298 K) spectrum of *3-(naphthalen-1-ylthio)-2-phenylbenzofuran*, **3ki**.

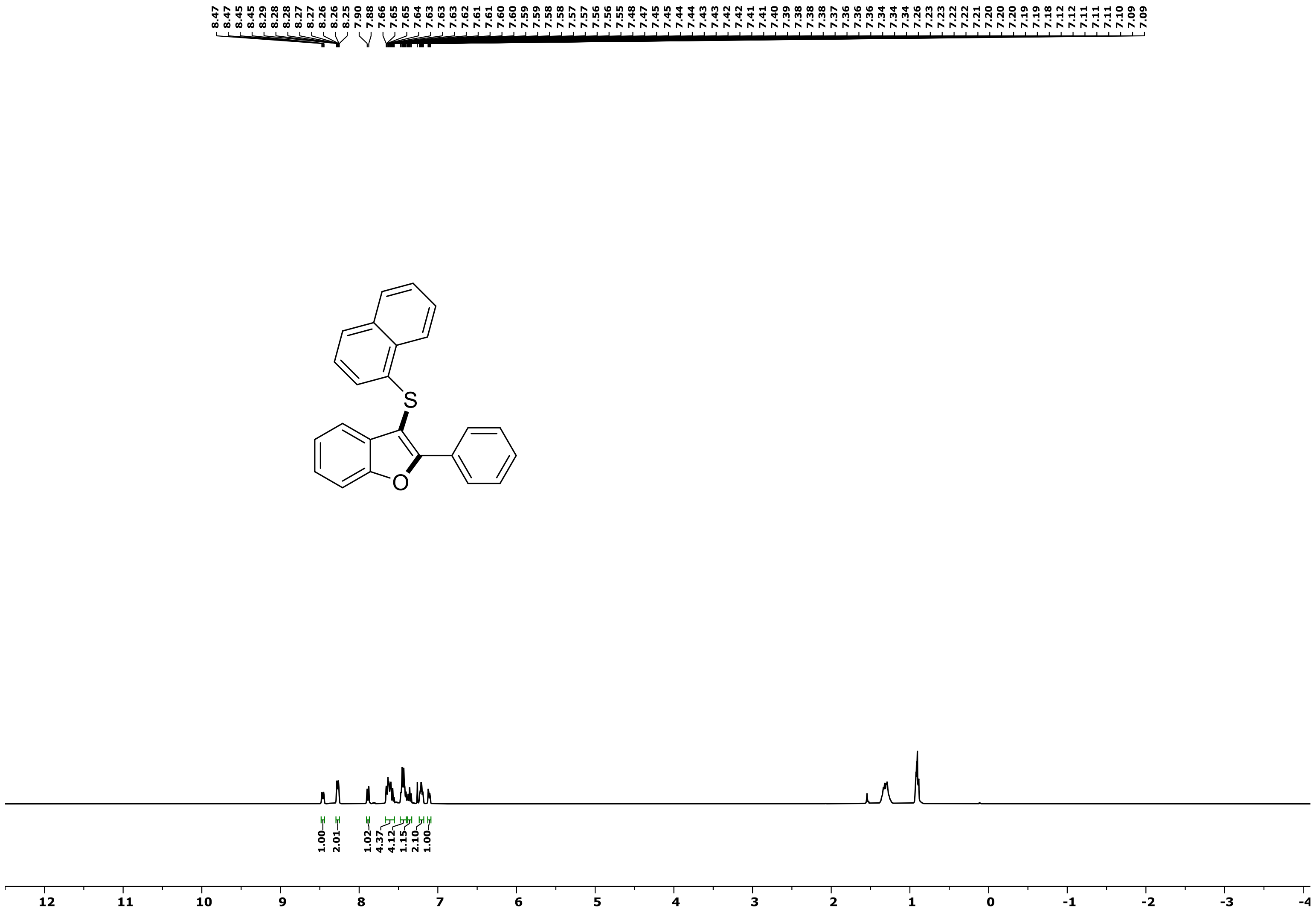

Figure S119: ^{13}C NMR (101 MHz, $CDCl_3$, 298 K) spectrum of *3-(naphthalen-1-ylthio)-2-phenylbenzofuran*, **3ki**.

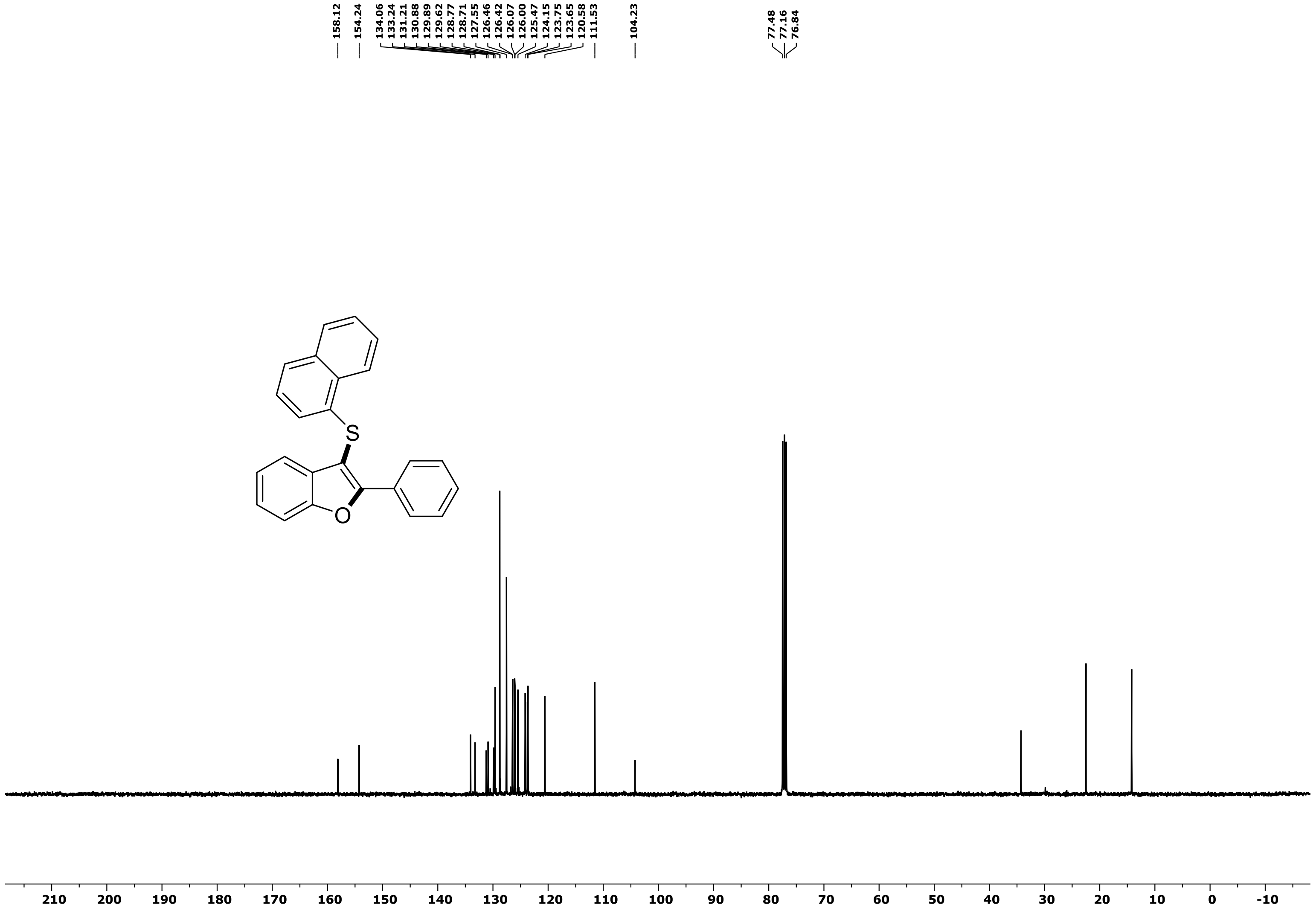

Figure S120: ^{1}H NMR (400 MHz, $CDCl_3$, 298 K) spectrum of *3-(cyclohexylthio)-2-phenylbenzofuran*, **3kj**.

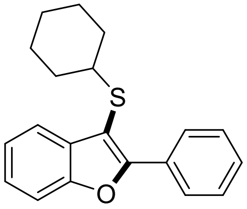

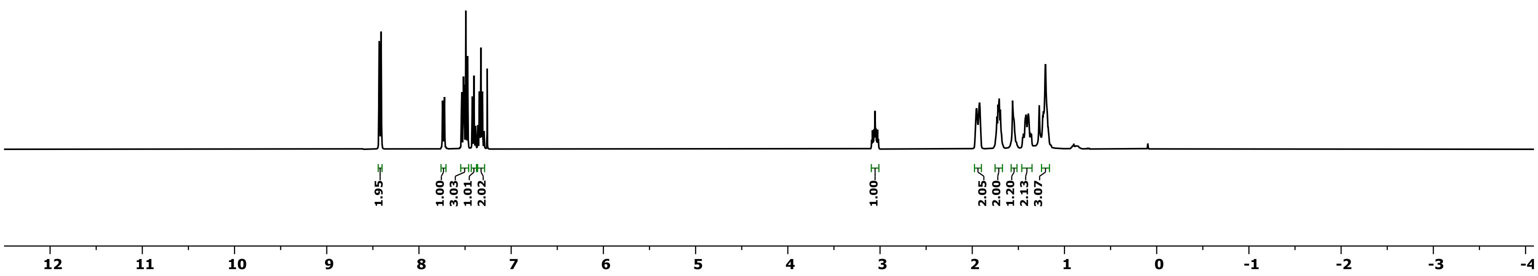

Figure S121: ^{13}C NMR (101 MHz, $CDCl_3$, 298 K) spectrum of *3-(cyclohexylthio)-2-phenylbenzofuran*, **3kj**.

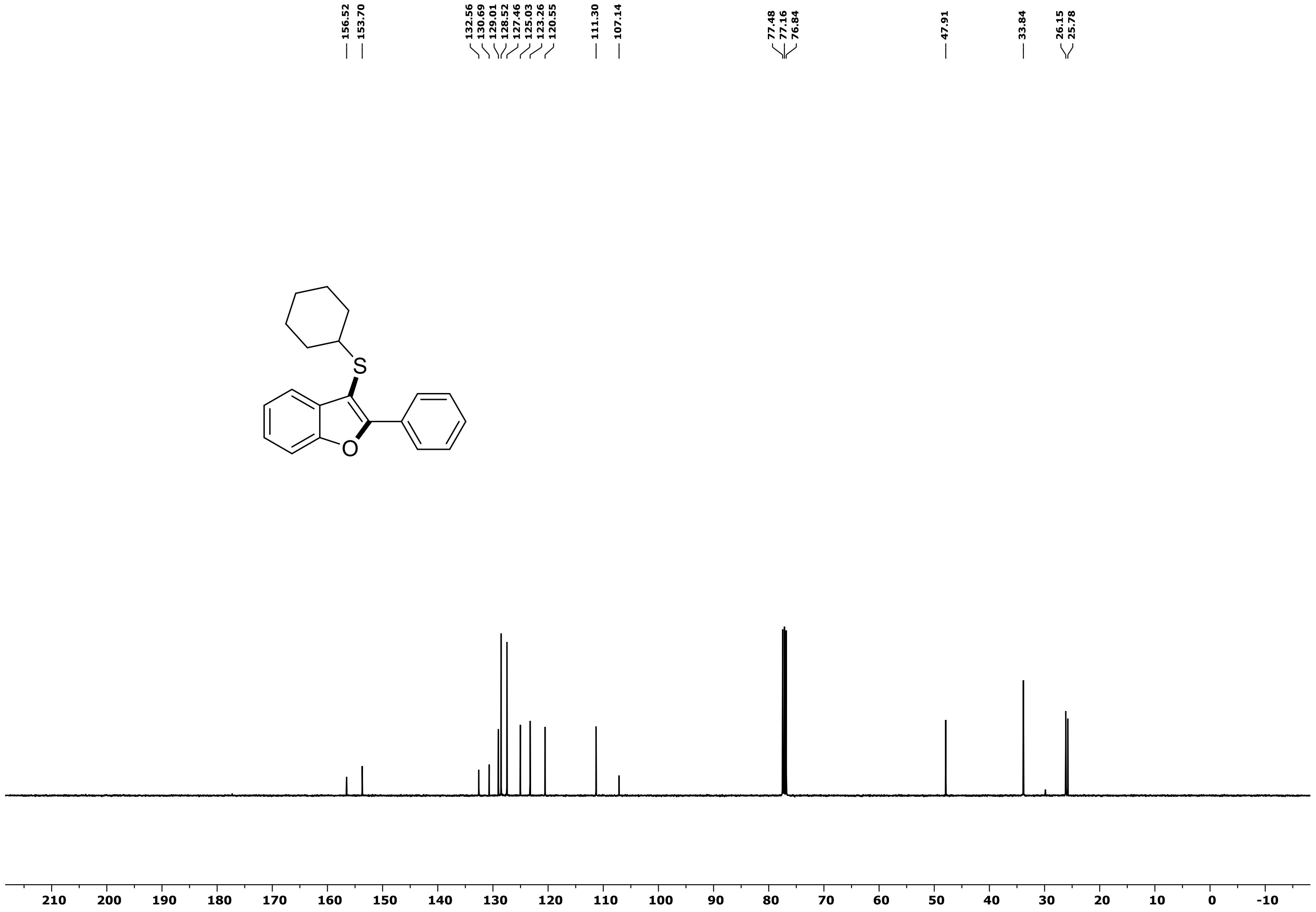

Figure S122: ^{1}H NMR (400 MHz, $CDCl_3$, 298 K) spectrum of *2-phenyl-3-((trifluoromethyl)thio)benzofuran*, **3kk**.

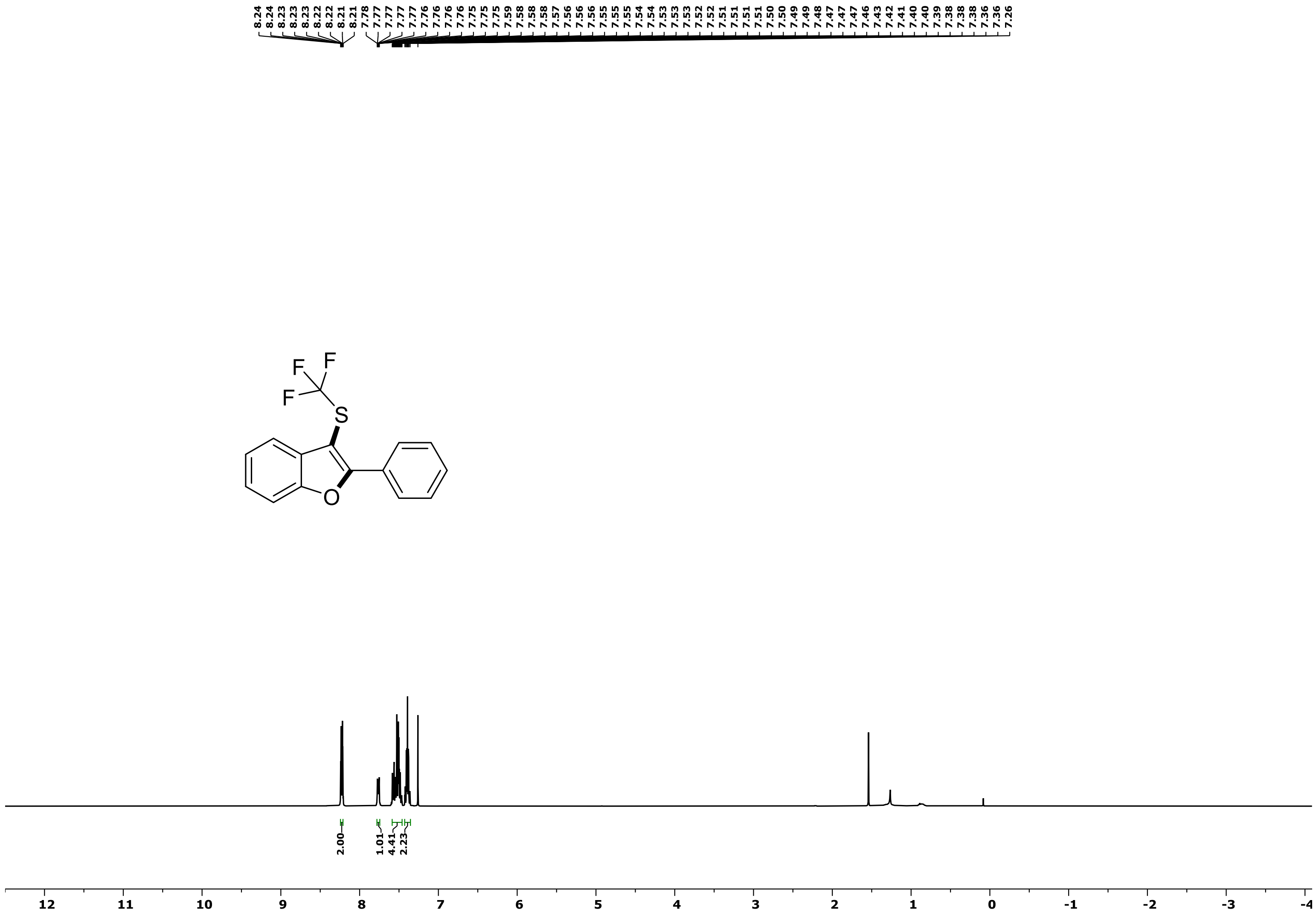

Figure S123: ^{13}C NMR (101 MHz, $CDCl_3$, 298 K) spectrum of *2-phenyl-3-((trifluoromethyl)thio)benzofuran*, **3kk**.

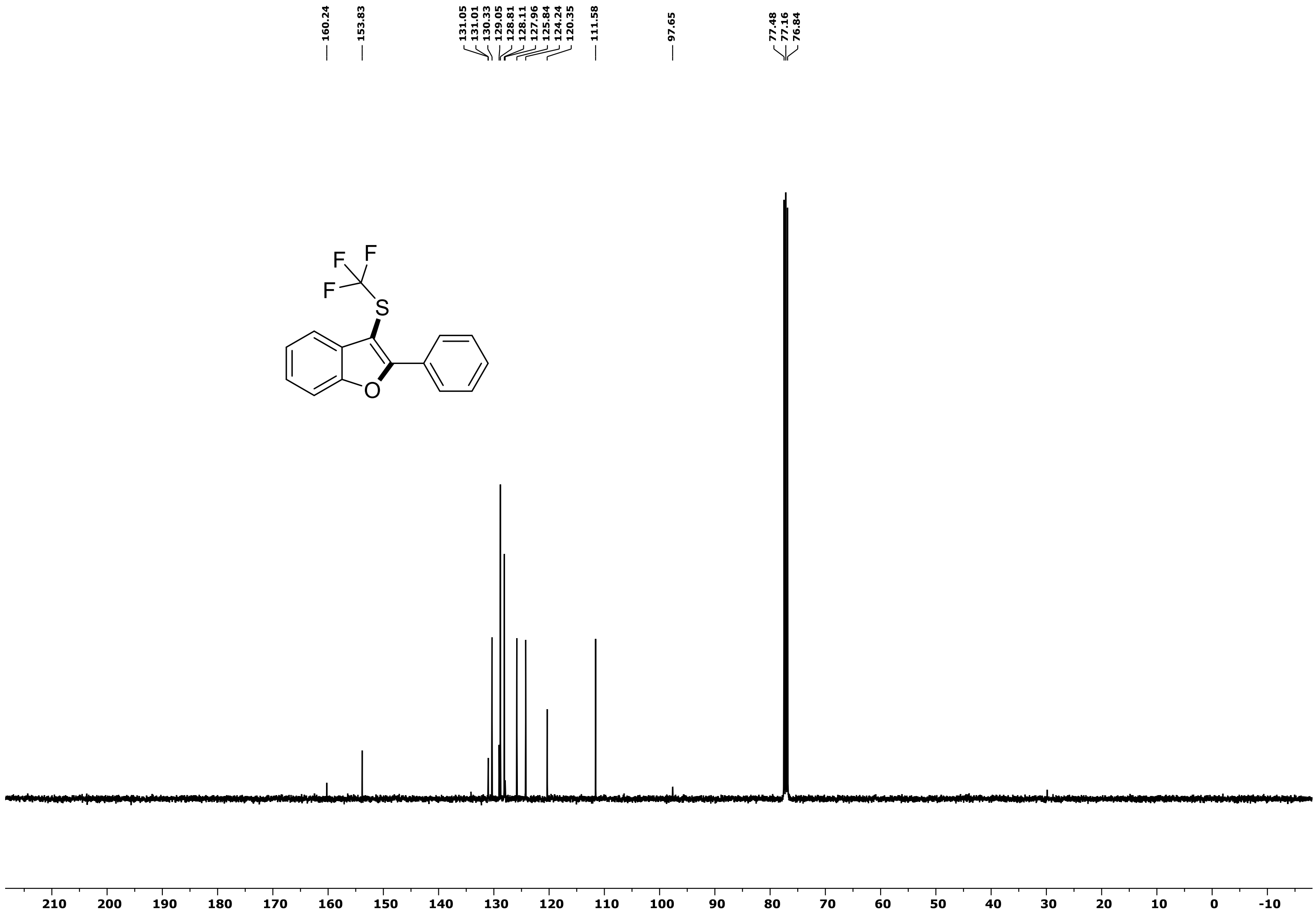

Figure S124: ^{19}F NMR (376 MHz, $CDCl_3$, 298 K) spectrum of *2-phenyl-3-((trifluoromethyl)thio)benzofuran*, **3kk**.

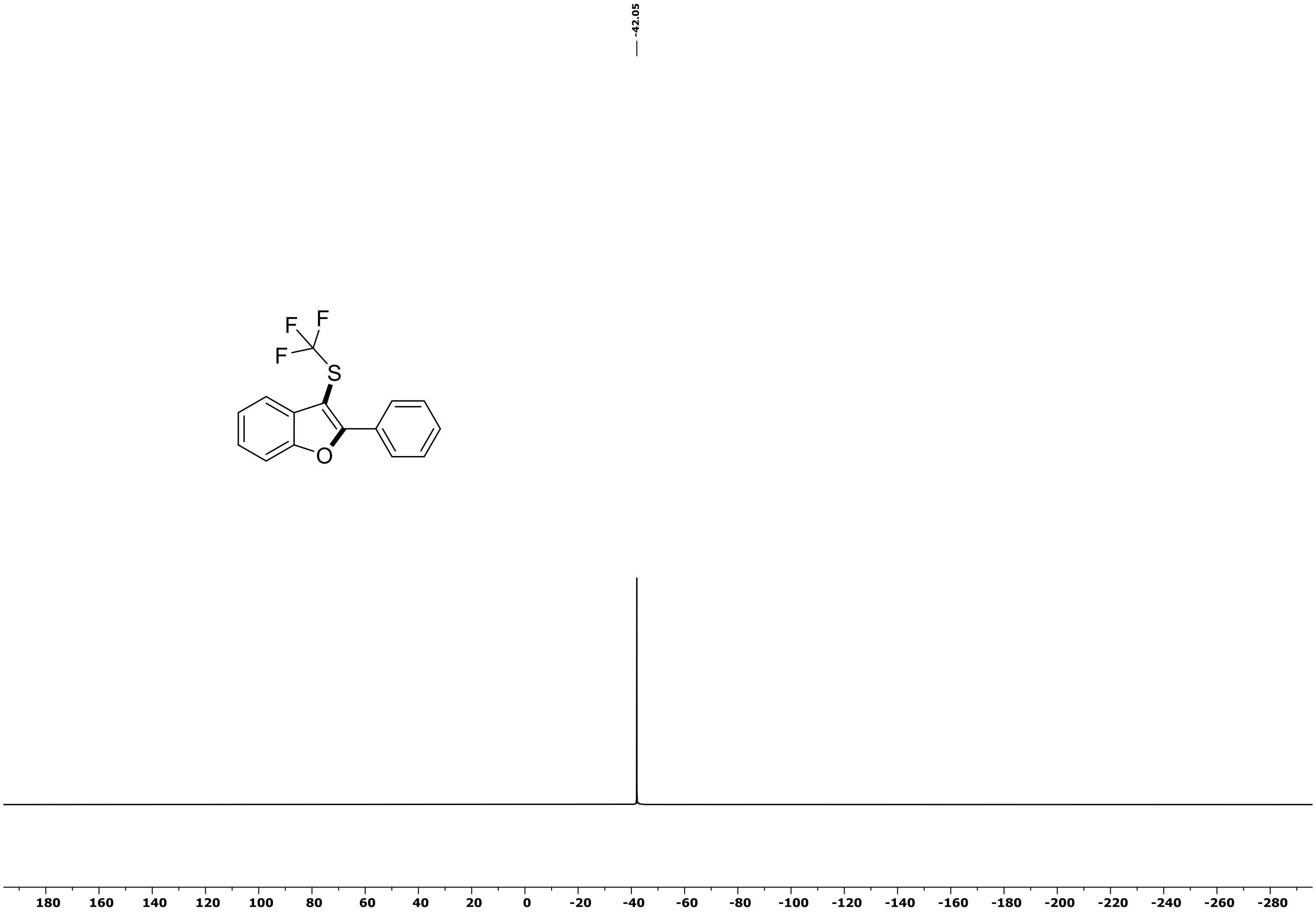

Figure S125: ^{1}H NMR (400 MHz, $CDCl_3$, 298 K) spectrum of *3-((4-(tert-butyl)phenyl)thio)-4-phenyl-2H-chromene*, **5aa**.

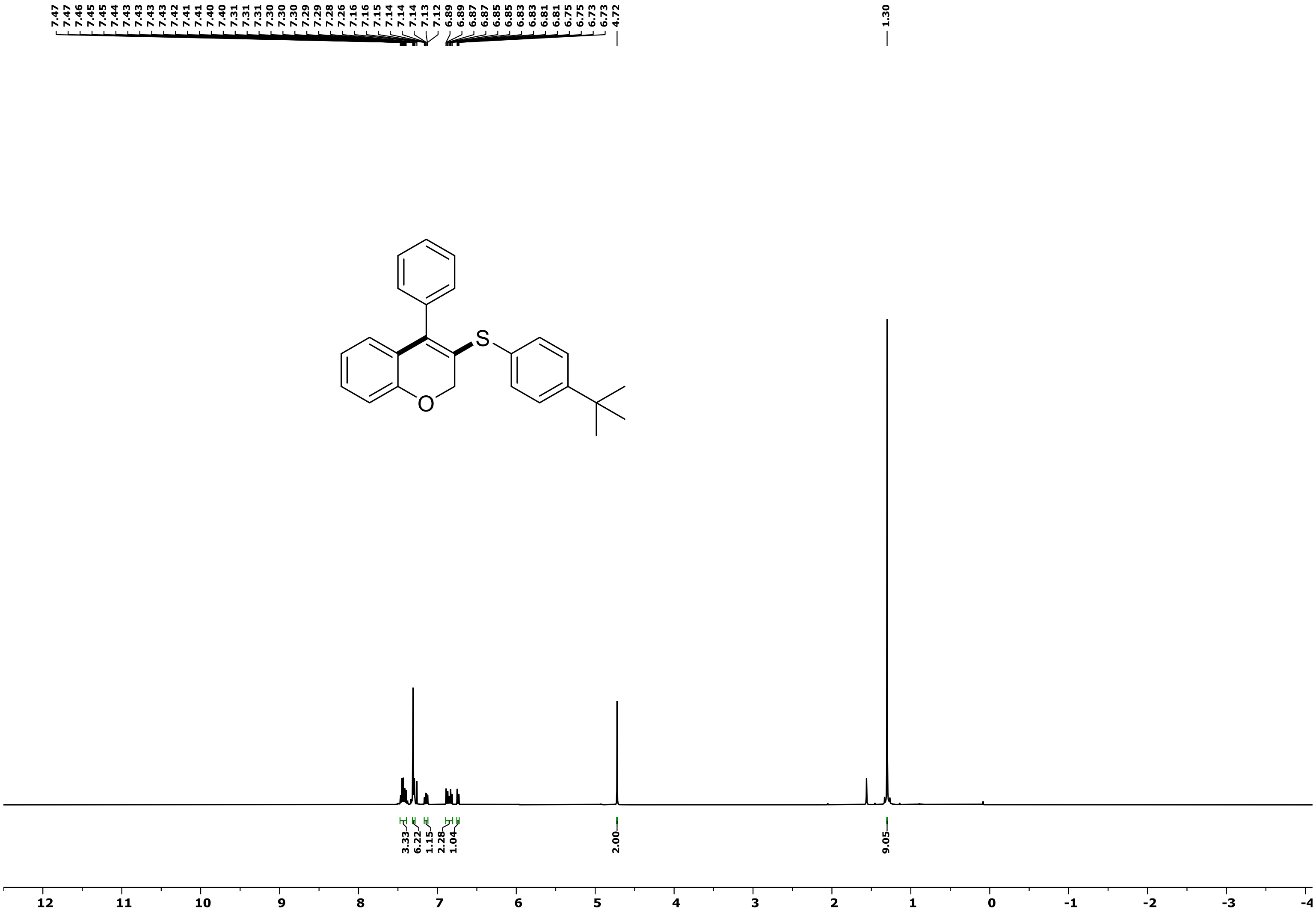

Figure S126: ^{13}C NMR (101 MHz, $CDCl_3$, 298 K) spectrum of *3-((4-(tert-butyl)phenyl)thio)-4-phenyl-2H-chromene*, **5aa**.

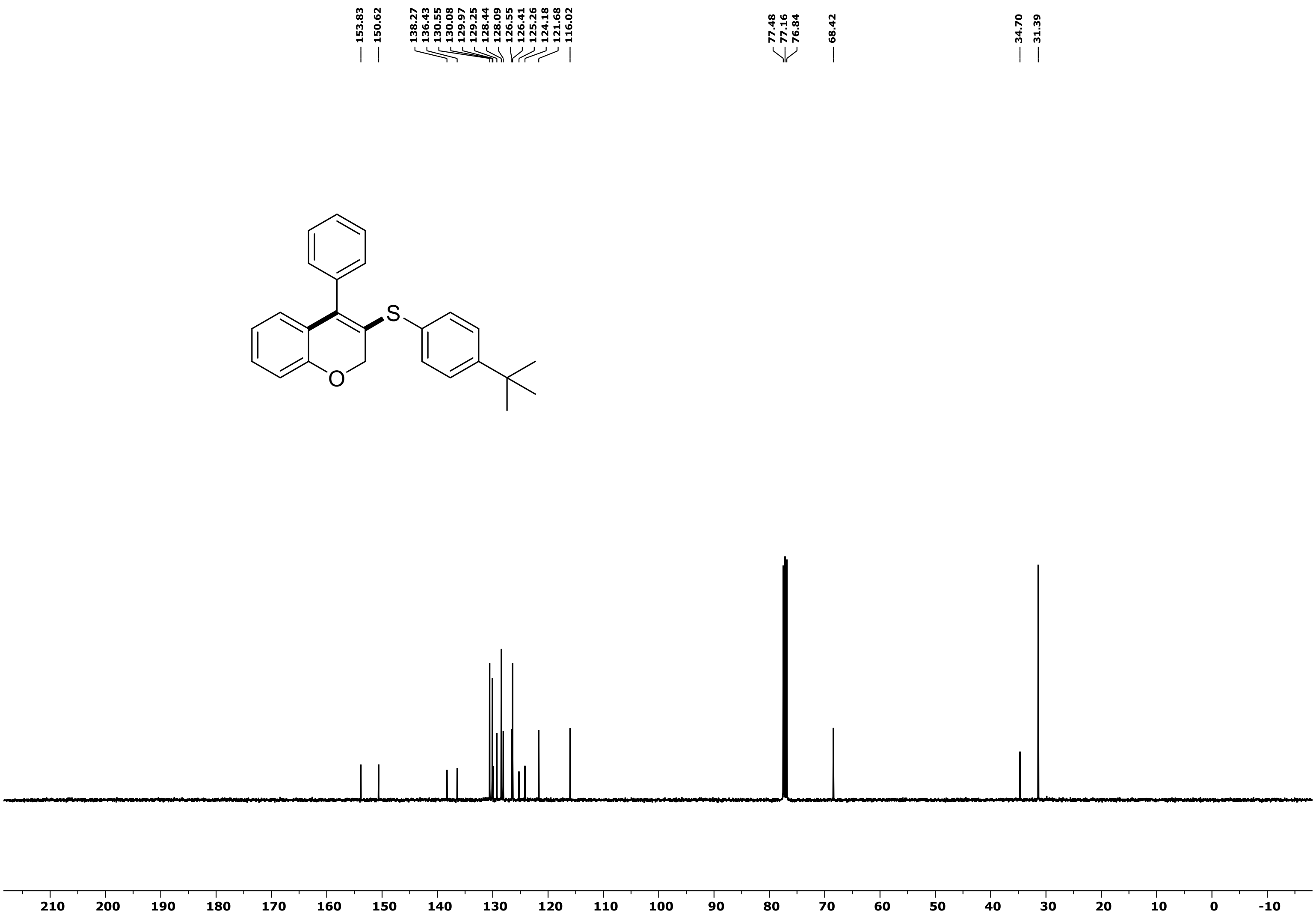

Figure S127: ^{1}H NMR (400 MHz, $CDCl_3$, 298 K) spectrum of *3-((4-(tert-butyl)phenyl)thio)-6-methyl-4-phenyl-2H-chromene*, **5ba**.

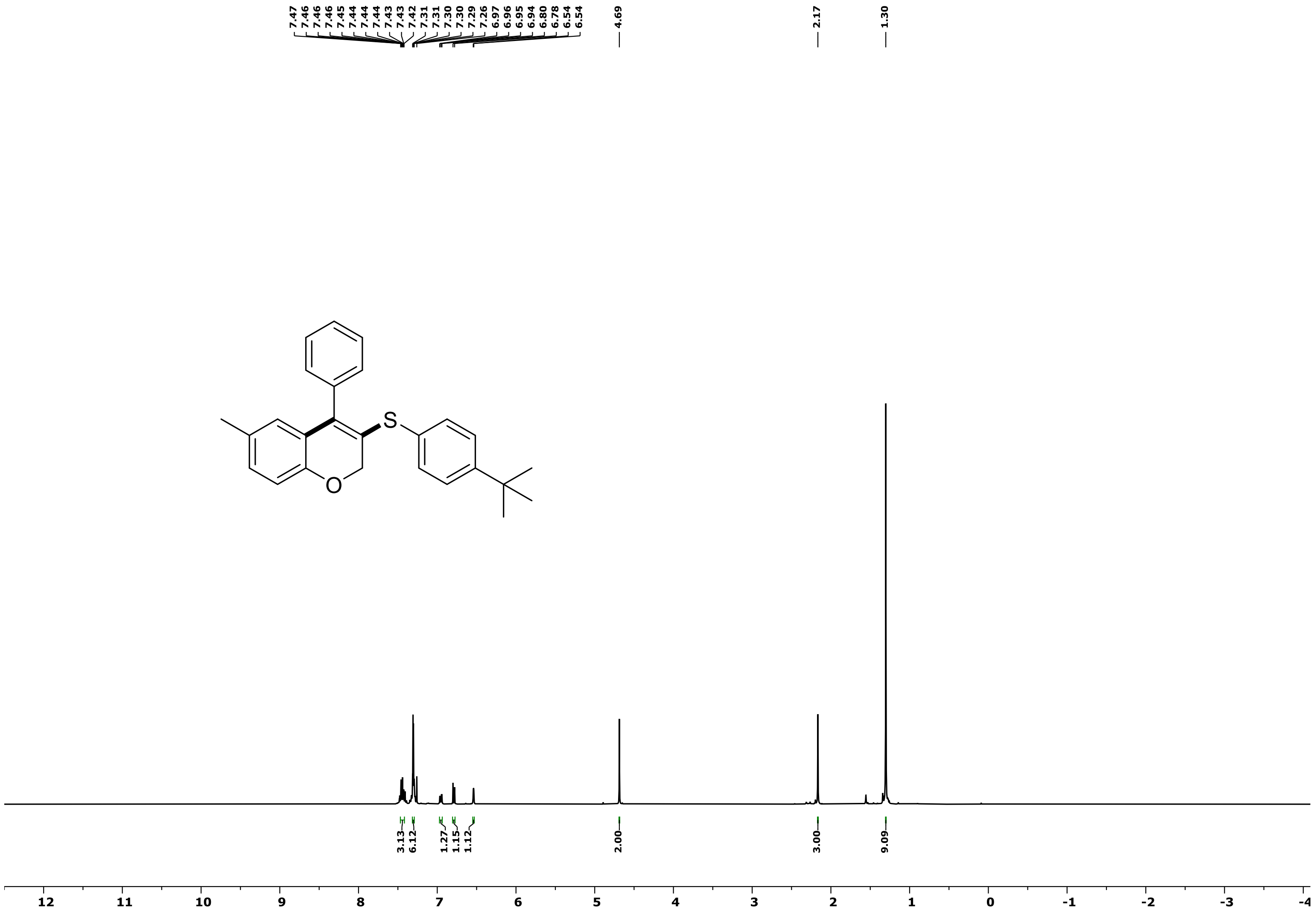

Figure S128: ^{13}C NMR (101 MHz, $CDCl_3$, 298 K) spectrum of *3-((4-(tert-butyl)phenyl)thio)-6-methyl-4-phenyl-2H-chromene*, **5ba**.

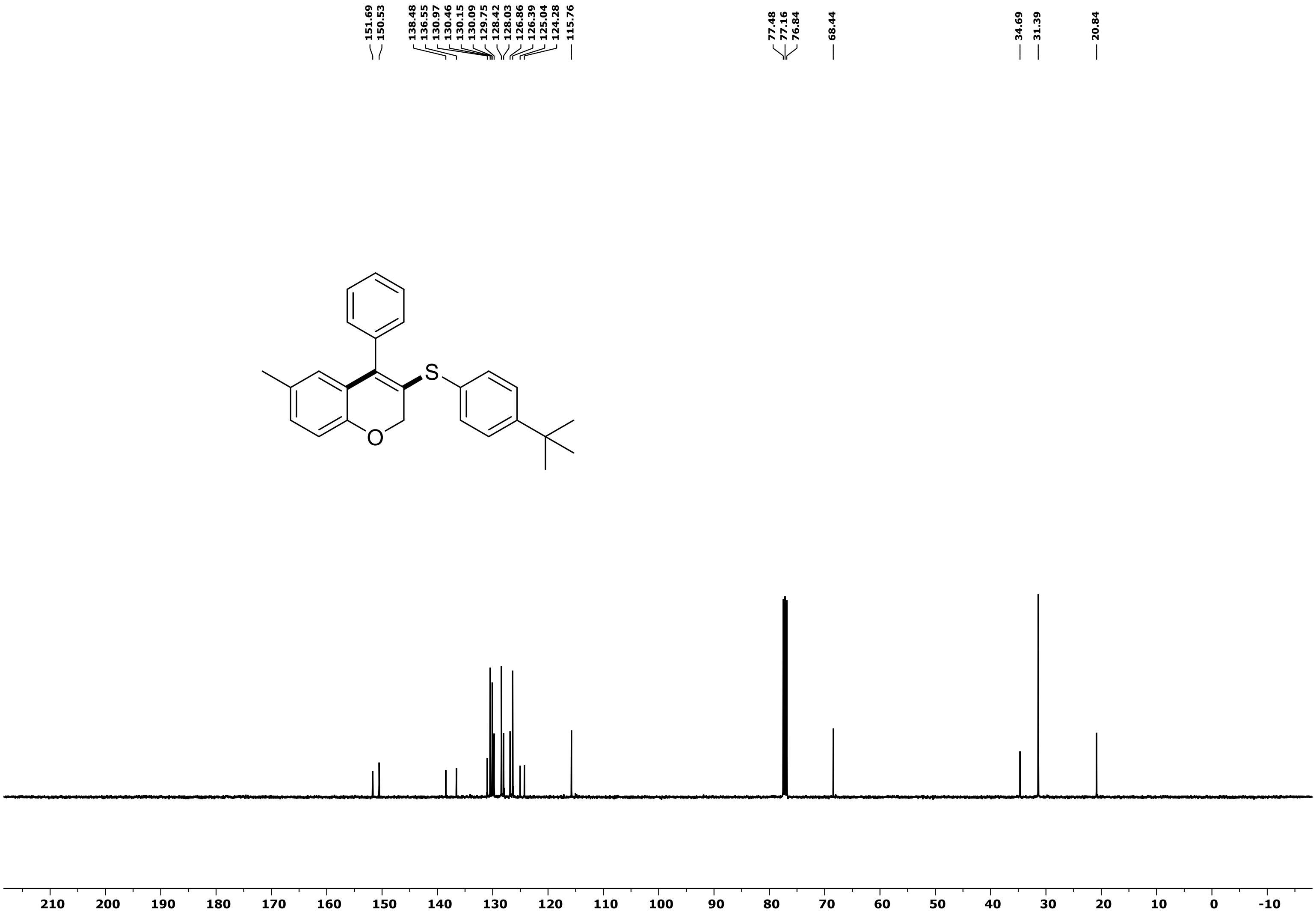

Figure S129: ^{1}H NMR (400 MHz, $CDCl_3$, 298 K) spectrum of *3-((4-(tert-butyl)phenyl)thio)-4-(4-methoxyphenyl)-2H-chromene*, **5ca**.

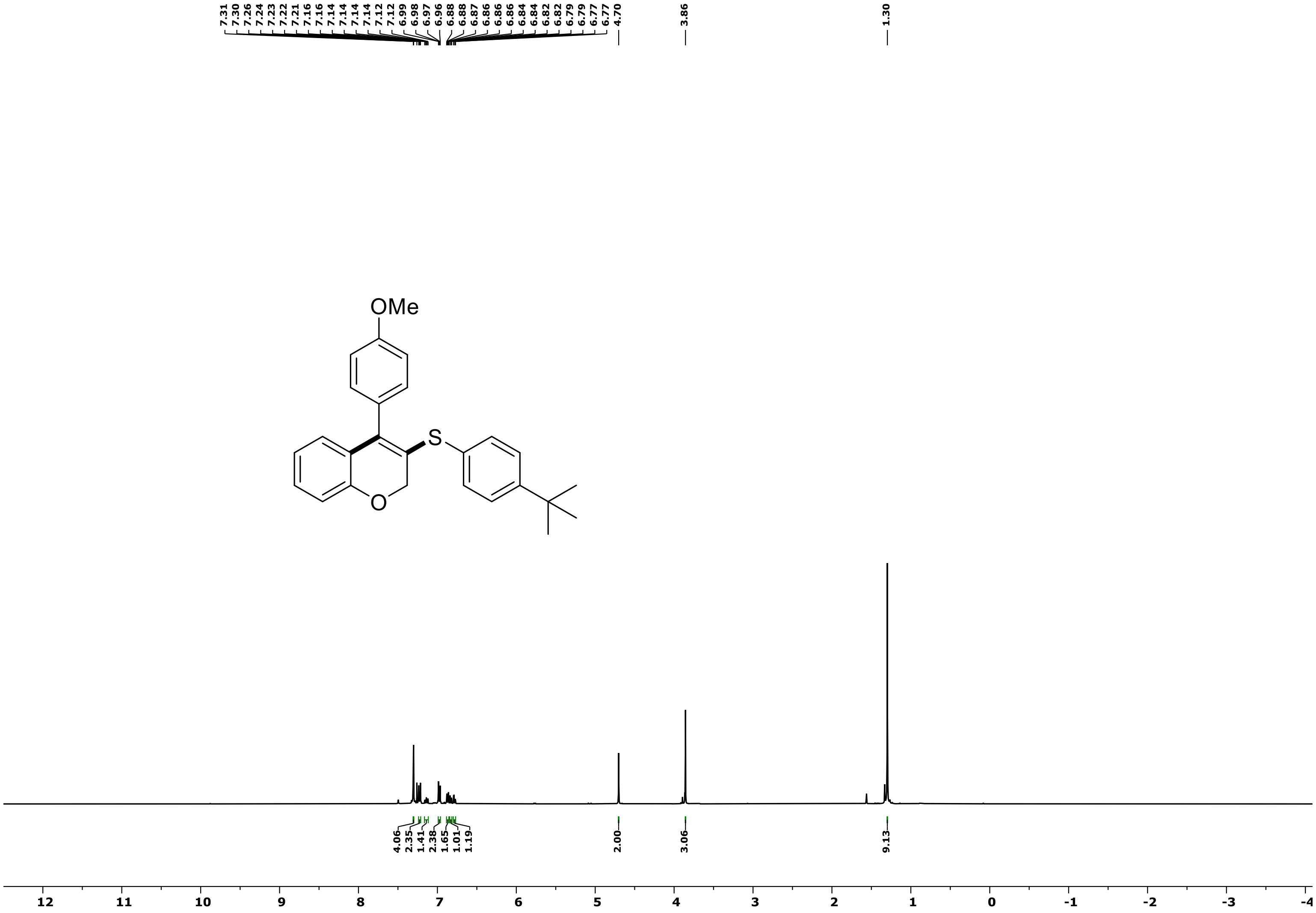

Figure S130: ^{13}C NMR (101 MHz, $CDCl_3$, 298 K) spectrum of *3-((4-(tert-butyl)phenyl)thio)-4-(4-methoxyphenyl)-2H-chromene*, **5ca**.

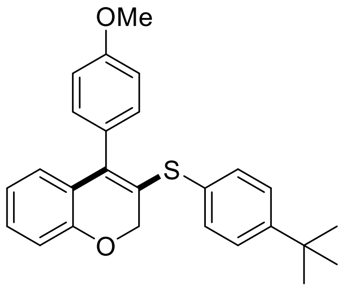

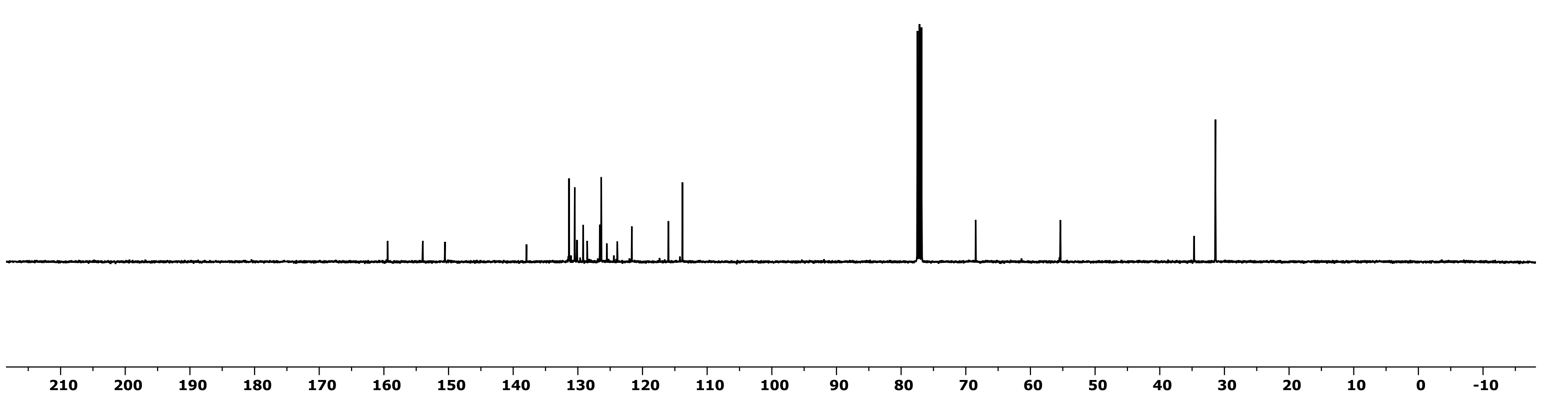

Figure S131: ^{1}H NMR (400 MHz, $CDCl_3$, 298 K) spectrum of *4-(benzo[d][1,3]dioxol-5-yl)-3-((4-(tert-butyl)phenyl)thio)-2H-chromene*, **5da**.

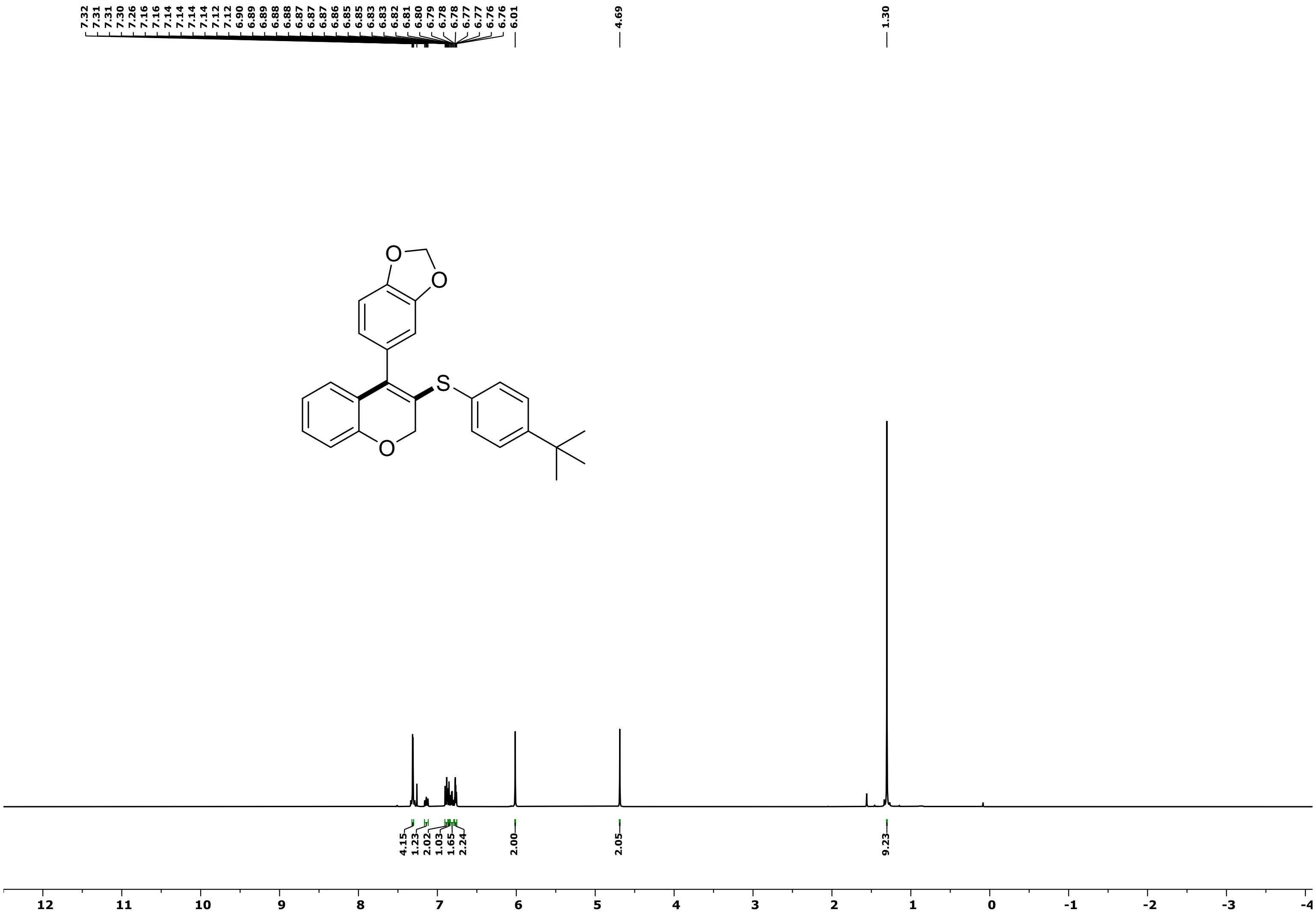

Figure S132: ^{13}C NMR (101 MHz, $CDCl_3$, 298 K) spectrum of *4-(benzo[d][1,3]dioxol-5-yl)-3-((4-(tert-butyl)phenyl)thio)-2H-chromene*, **5da**.

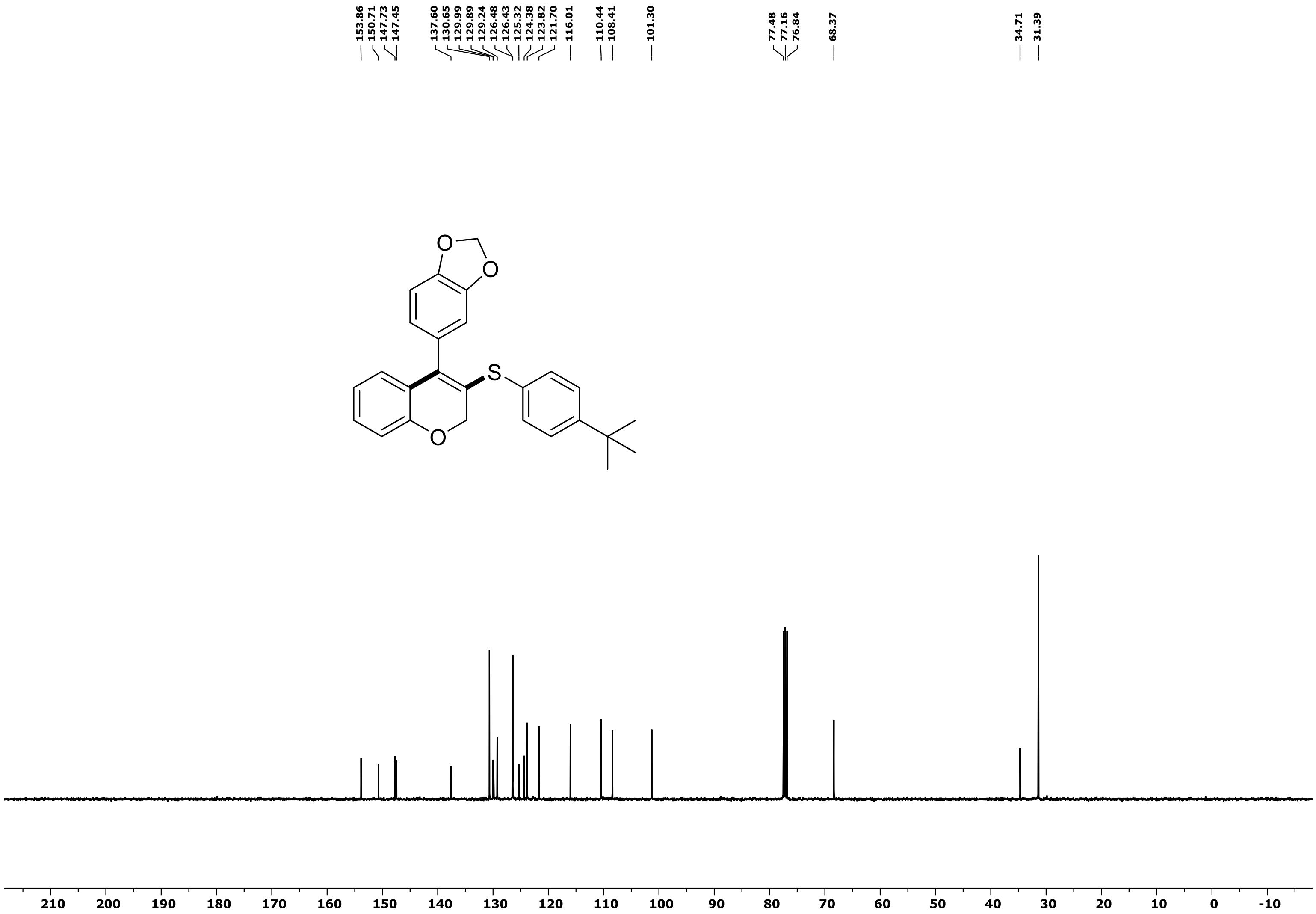

Figure S133: ^{1}H NMR (400 MHz, $CDCl_3$, 298 K) spectrum of *3-((4-(tert-butyl)phenyl)thio)-4-(3-(trifluoromethyl)phenyl)-2H-chromene*, **5ea**.

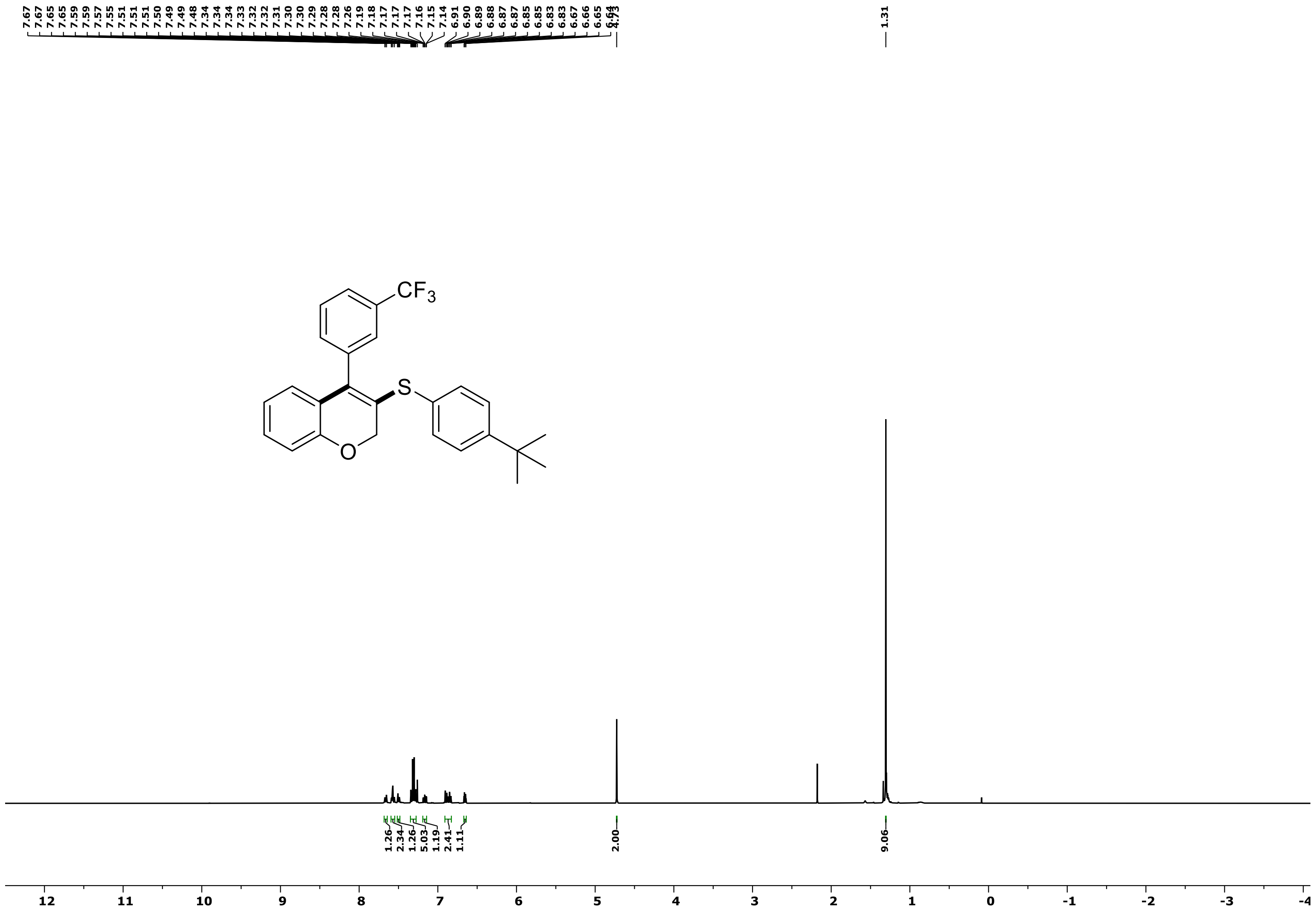

Figure S134: ^{13}C NMR (101 MHz, $CDCl_3$, 298 K) spectrum of *3-((4-(tert-butyl)phenyl)thio)-4-(3-(trifluoromethyl)phenyl)-2H-chromene*, **5ea**.

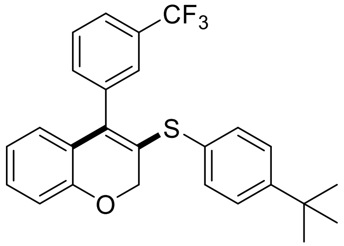

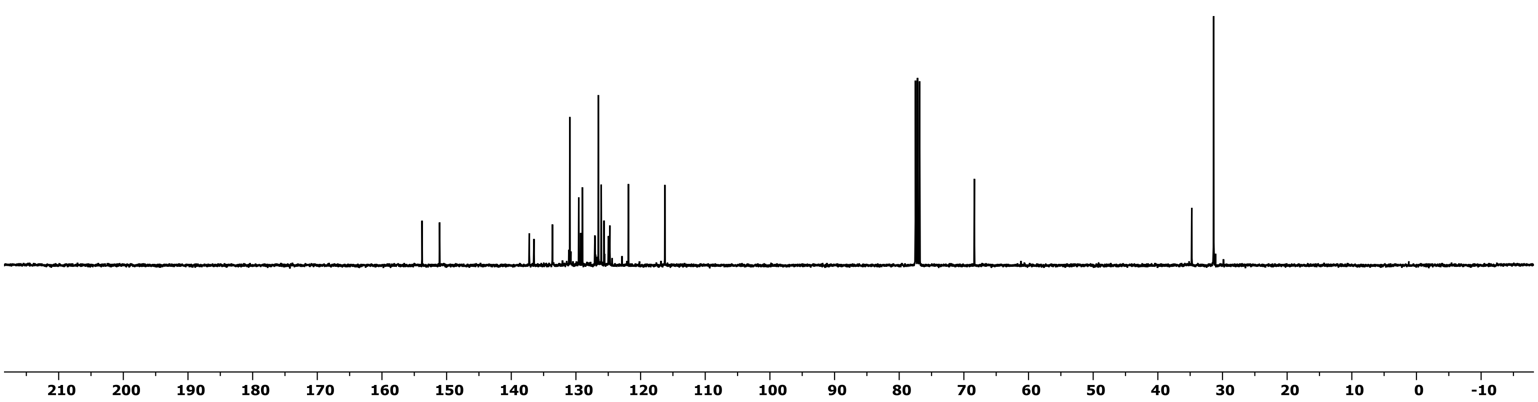

Figure S135: ^{19}F NMR (376 MHz, $CDCl_3$, 298 K) spectrum of *3-((4-(tert-butyl)phenyl)thio)-4-(3-(trifluoromethyl)phenyl)-2H-chromene*, **5ea**.

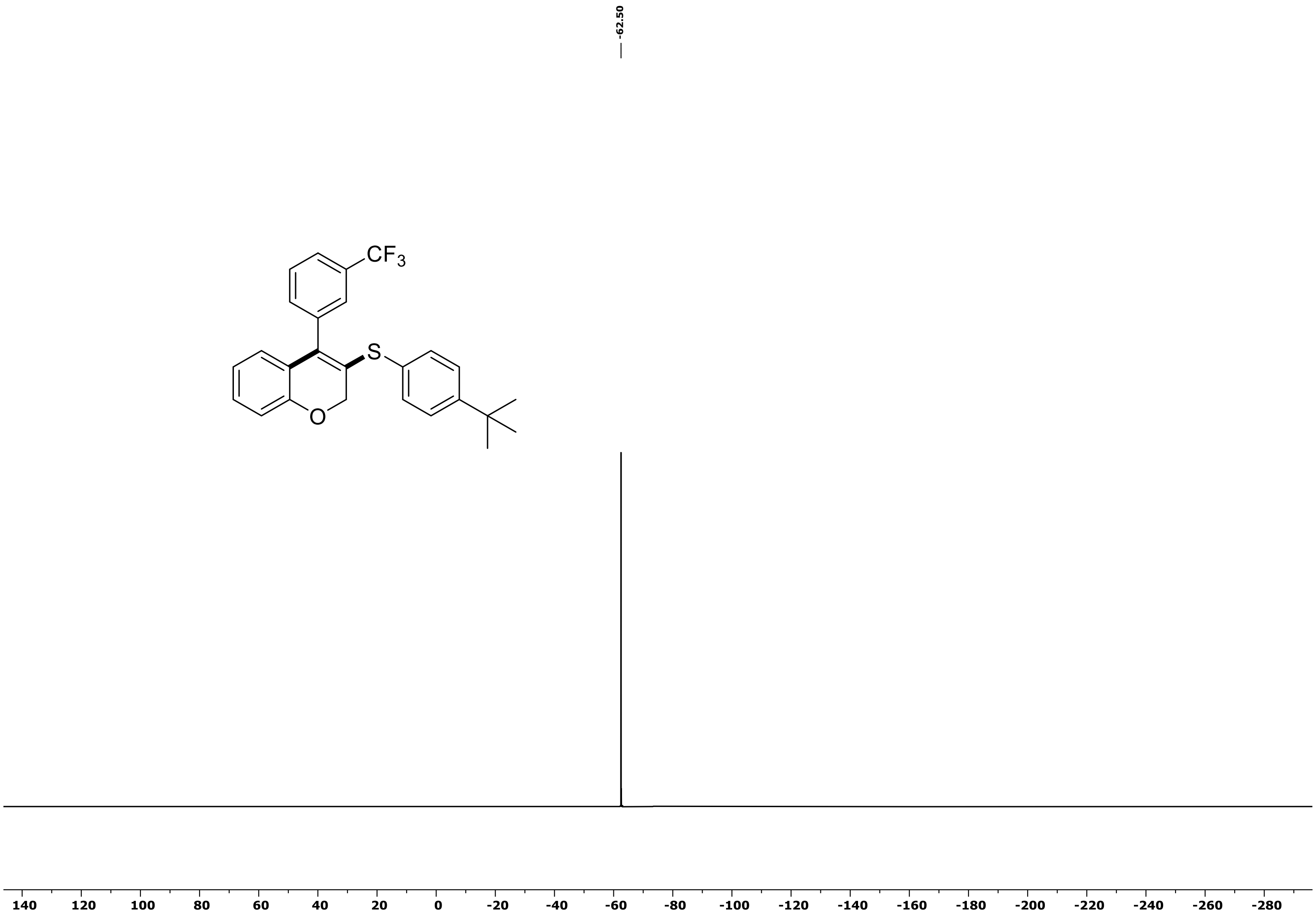

Figure S136: ^{1}H NMR (400 MHz, $CDCl_3$, 298 K) spectrum of *(4-(tert-butyl)phenyl)(1-phenyl-3,4-dihydronaphthalen-2-yl)sulfane*, **5ha**.

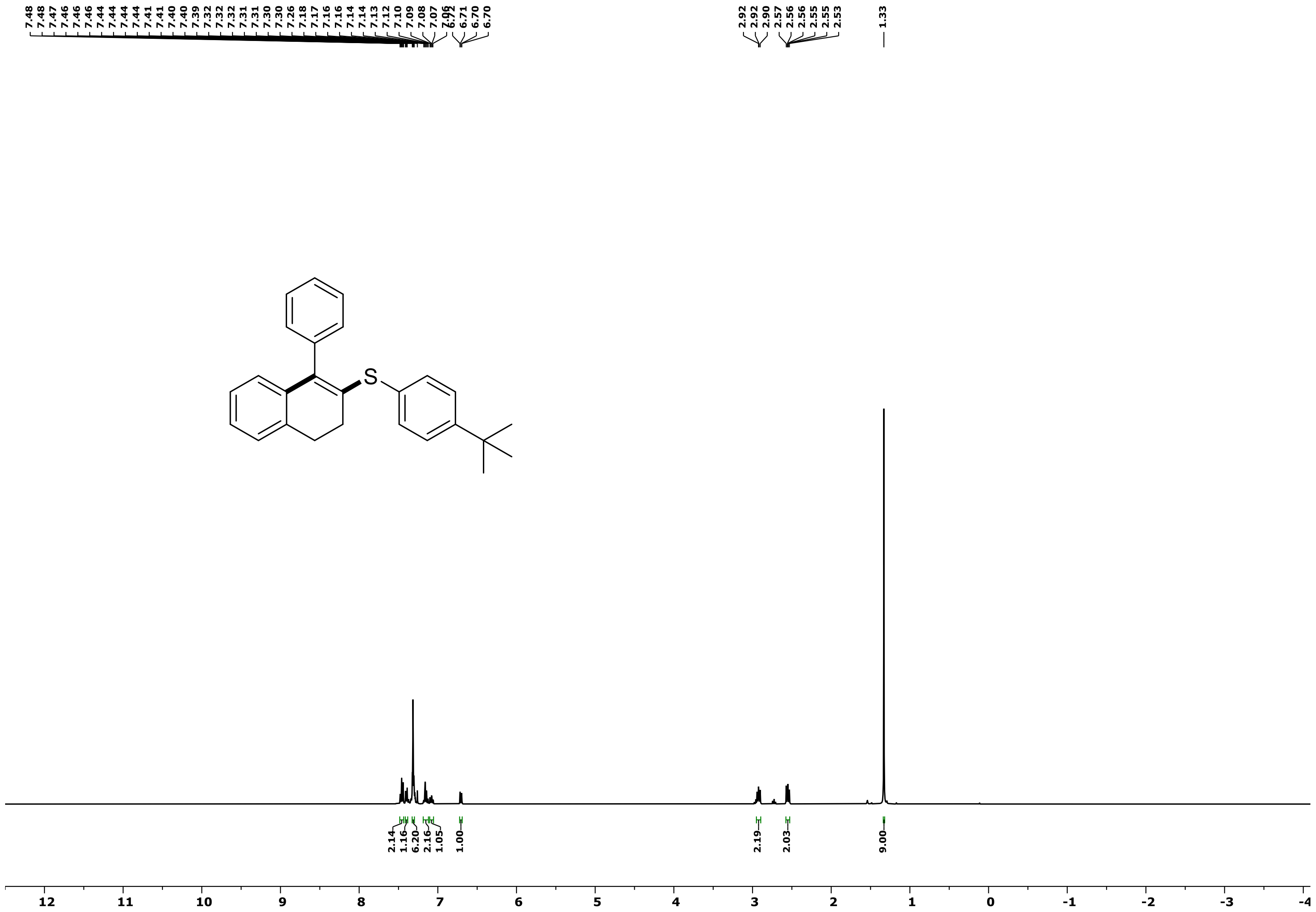

Figure S137: ^{13}C NMR (101 MHz, $CDCl_3$, 298 K) spectrum of *(4-(tert-butyl)phenyl)(1-phenyl-3,4-dihydronaphthalen-2-yl)sulfane*, **5ha**.

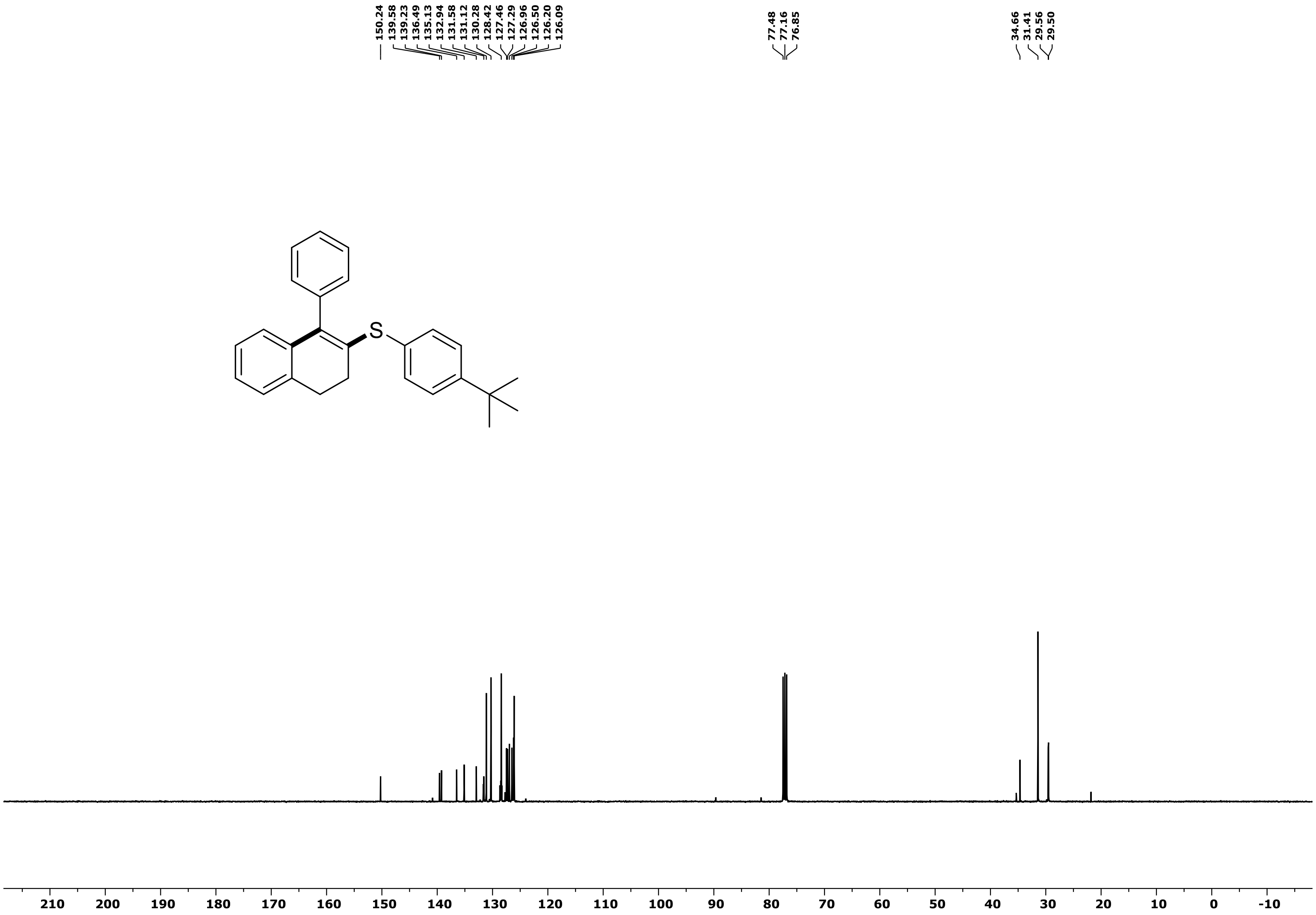

Figure S138: ^{1}H NMR (400 MHz, $CDCl_3$, 298 K) spectrum of *N-((4-(tert-butyl)phenyl)thio)-4-phenyl-1-tosyl-1,2-dihydroquinoline,* **5ia**.

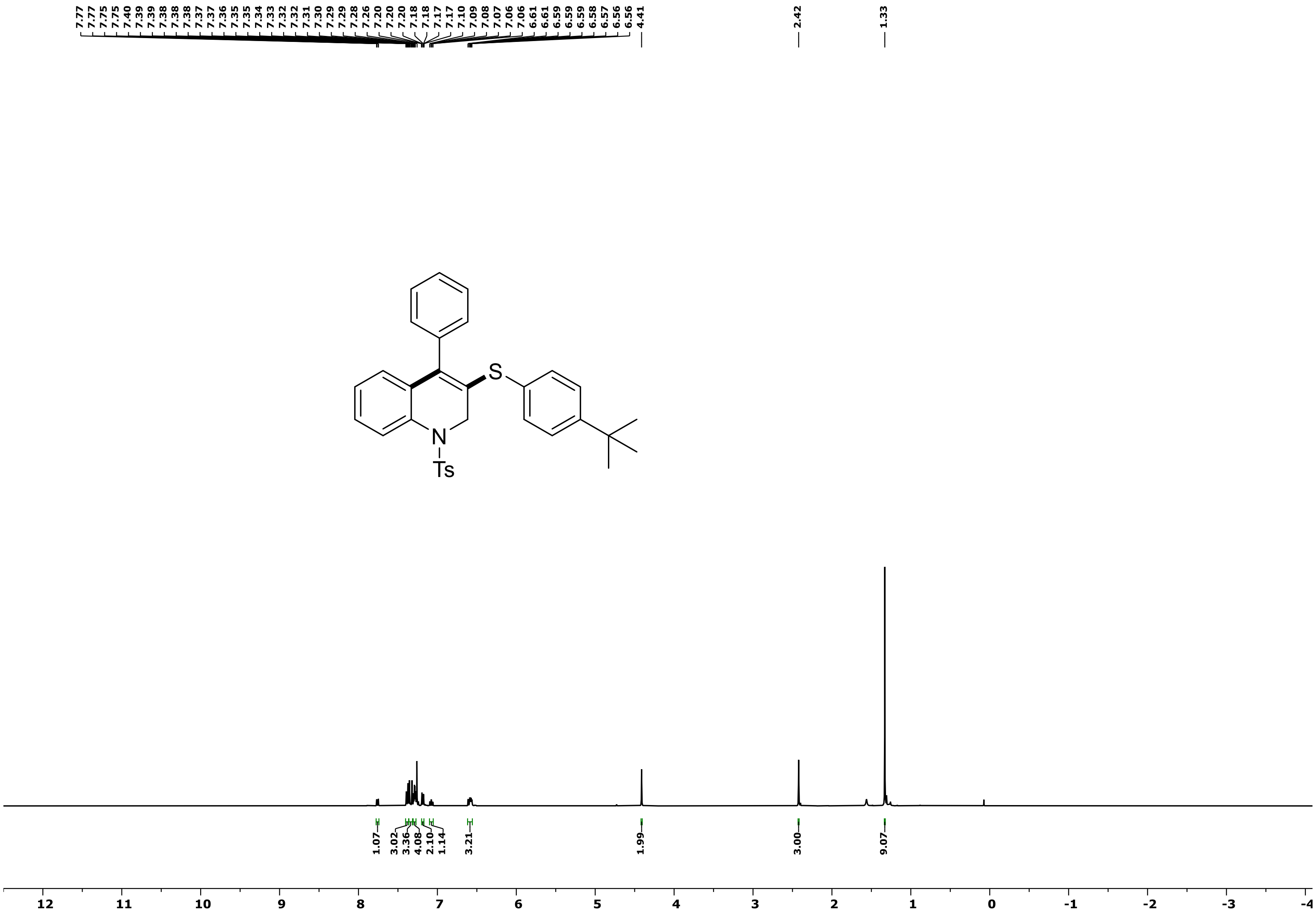

Figure S139: ^{13}C NMR (101 MHz, $CDCl_3$, 298 K) spectrum of *N-((4-(tert-butyl)phenyl)thio)-4-phenyl-1-tosyl-1,2-dihydroquinoline,* **5ia**.

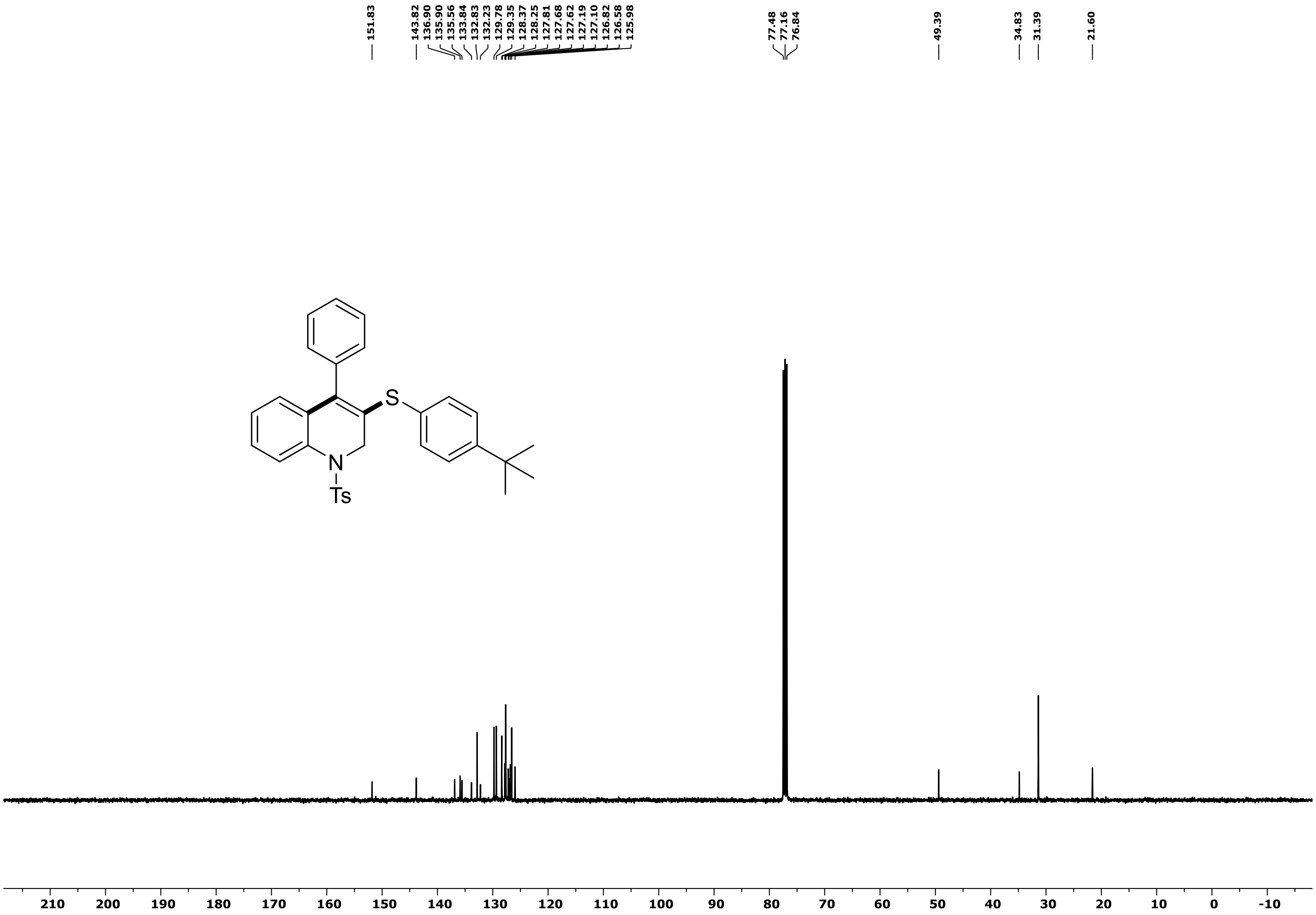

Figure S140: ^{1}H NMR (400 MHz, $CDCl_3$, 298 K) spectrum of *5-((4-(tert-butyl)phenyl)thio)-6-phenylnaphtho[1,2-b]benzofuran*, **7**.

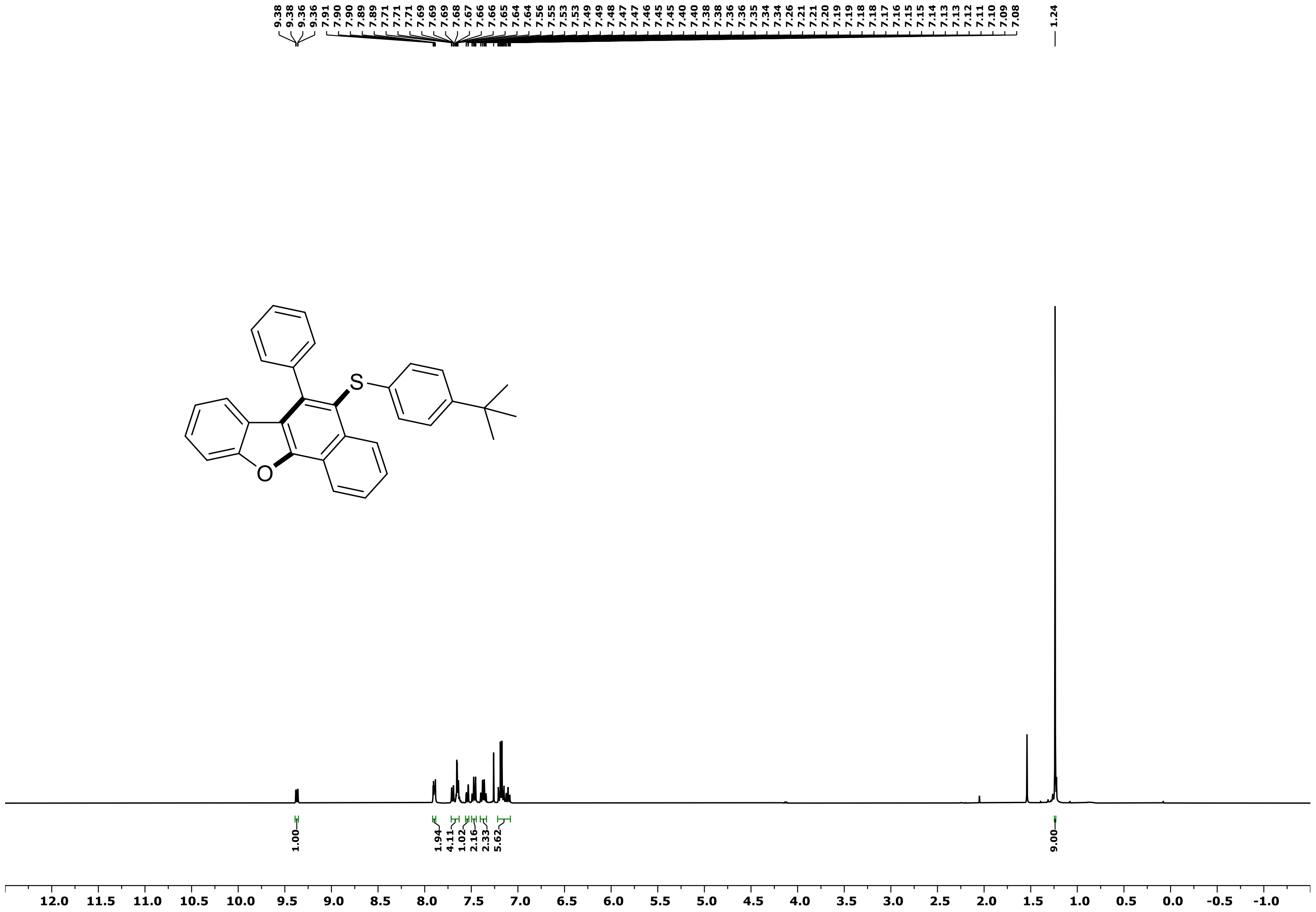

Figure S141: ^{13}C NMR (101 MHz, $CDCl_3$, 298 K) spectrum of *5-((4-(tert-butyl)phenyl)thio)-6-phenylnaphtho[1,2-b]benzofuran*, **7**.

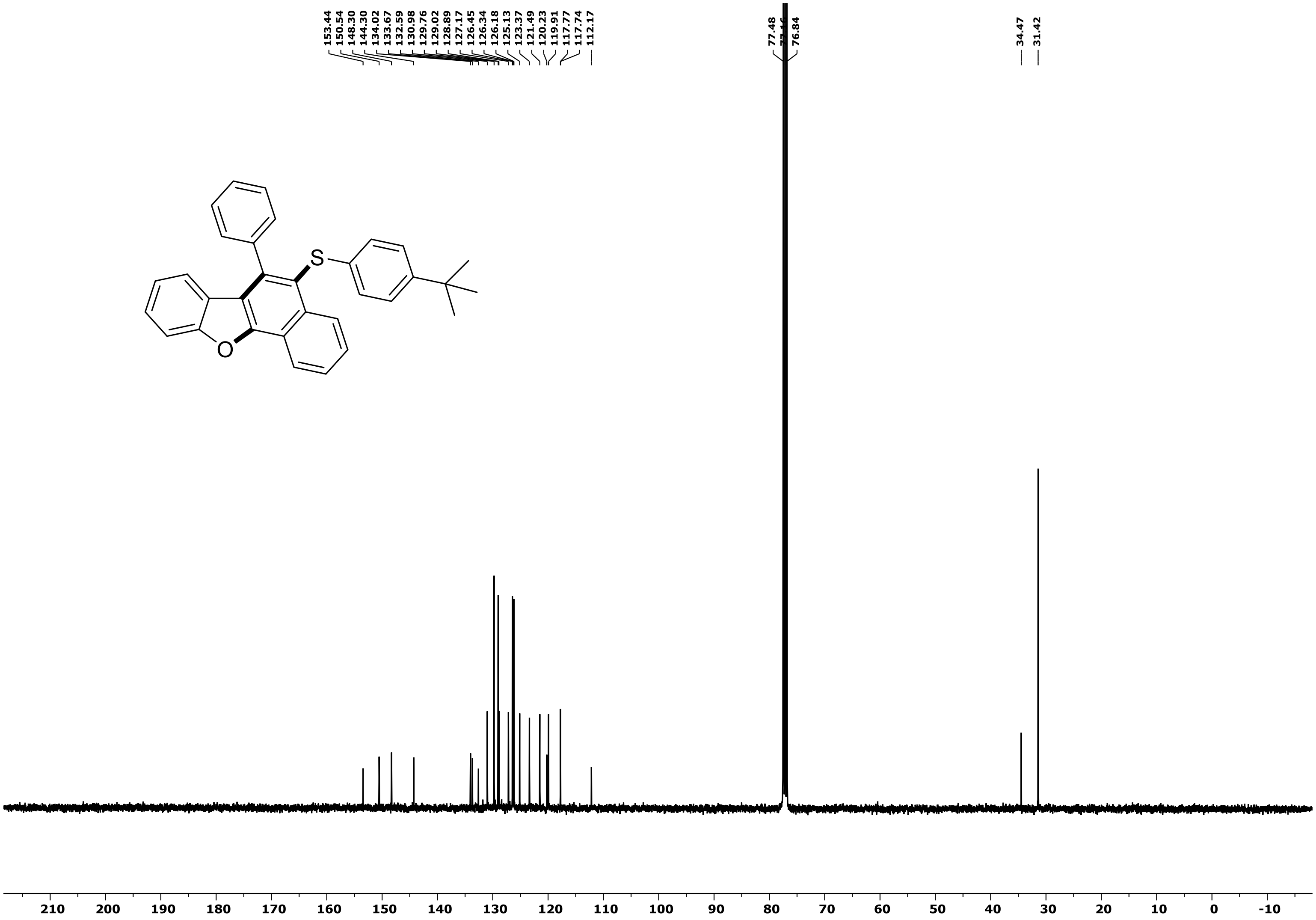

Figure S142: ^{1}H NMR (400 MHz, $CDCl_3$, 298 K) spectrum of *5-((4-(tert-butyl)phenyl)thio)-6-phenyl-11-tosyl-11H-benzo[a]carbazole*, **8**.

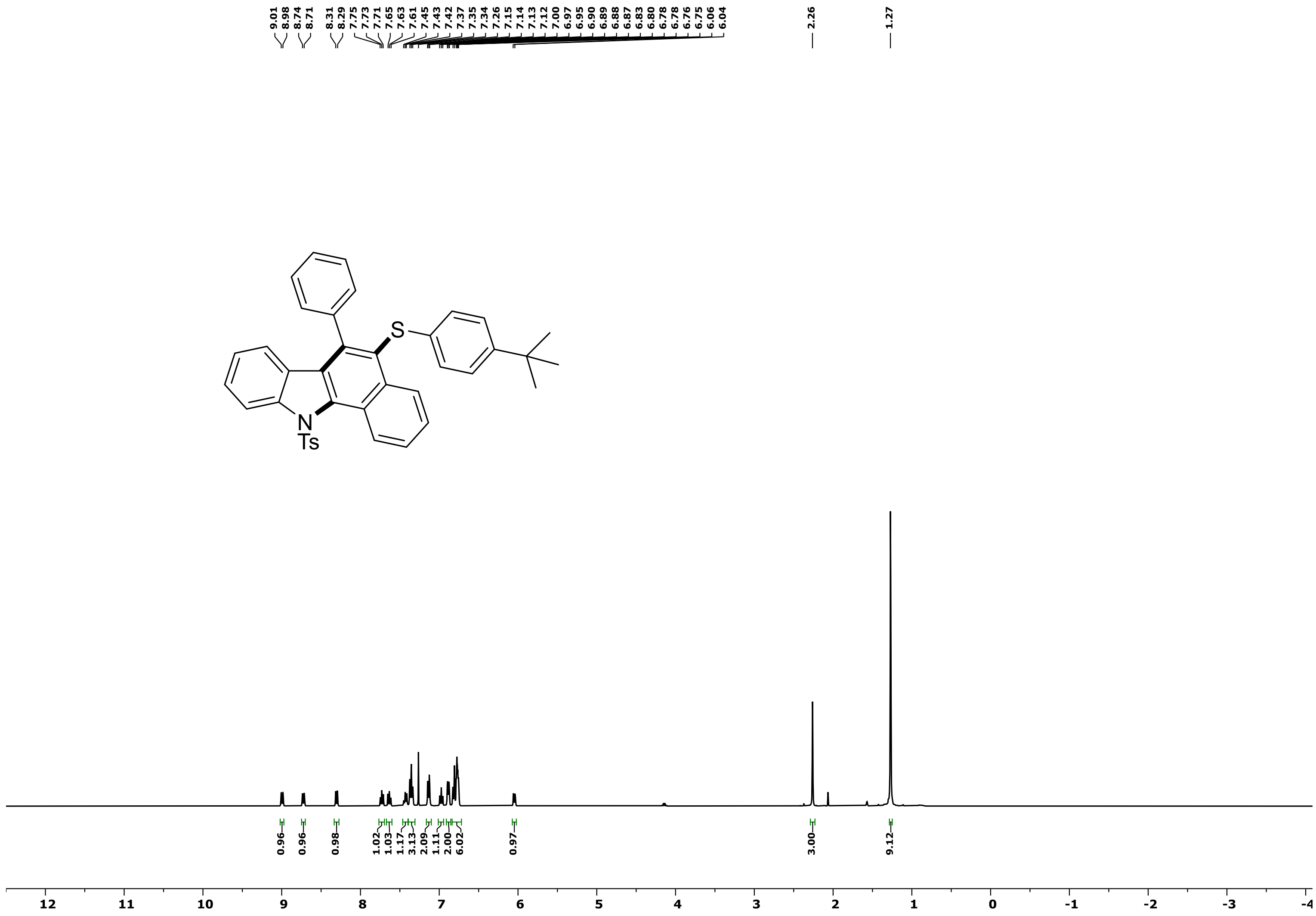

Figure S143: ^{13}C NMR (101 MHz, CDCl$_3$, 298 K) spectrum of *5-((4-(tert-butyl)phenyl)thio)-6-phenyl-11-tosyl-11H-benzo[a]carbazole*, **8**.

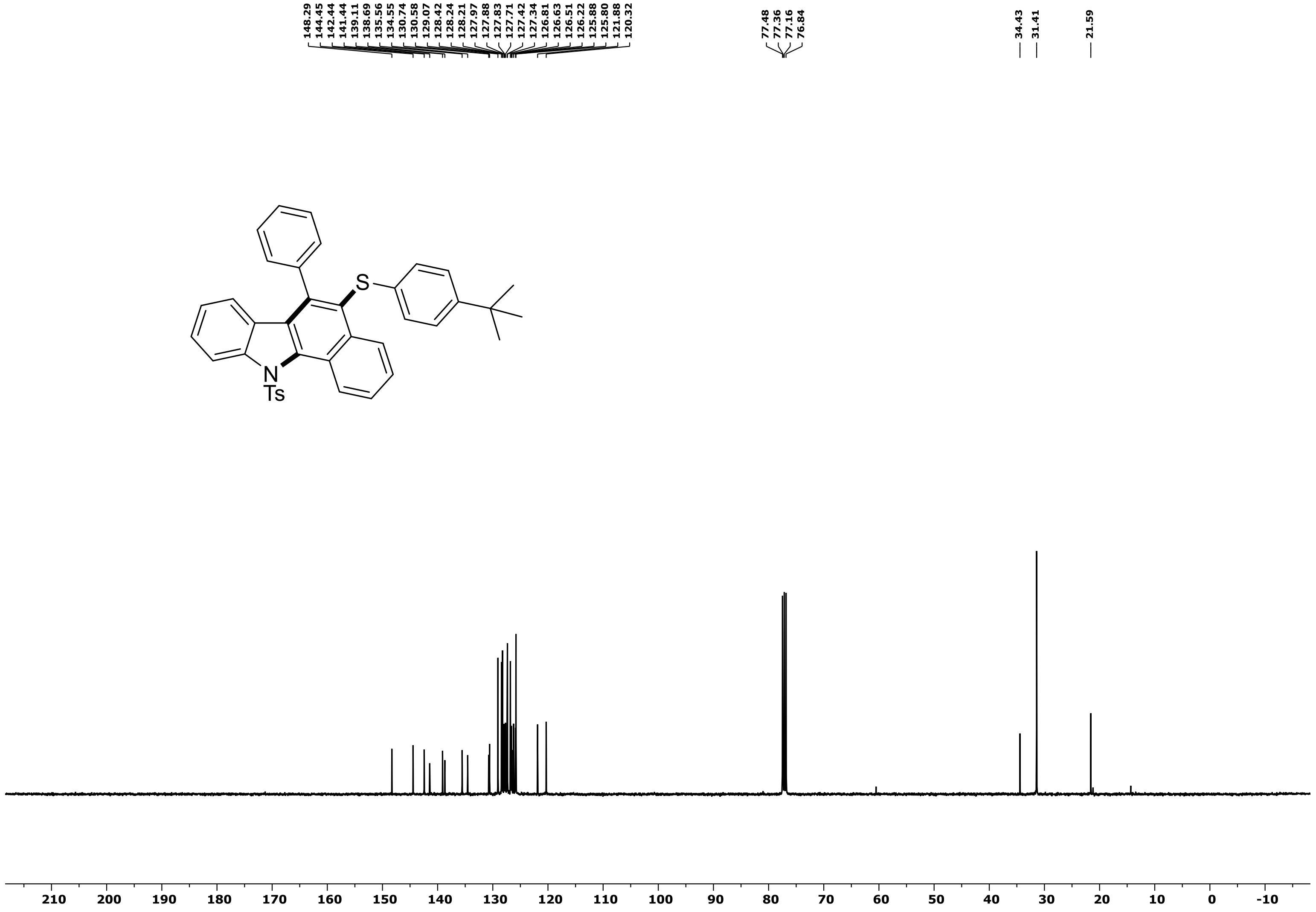

10. Kinetic studies

10.1 Experimental

Kinetic studies were carried out by preparing a series of solutions containing (0.02 mmol, 15 mg), i.e., 58 mM $B(C_6F_5)_3$ (BCF) in the presence of different concentrations of **2a** (0.1, 0.2, 0.5, 1.0, 2.0 equiv.) in 0.5 mL $CDCl_3$ in a glove box. Samples were kept at 45 °C for 5 mins before recording the ^{11}B and ^{19}F NMR spectra. Data were analysed using the Reaction Monitoring tool in MestreNova (version 16). The chemical shifts of the various peaks were recorded and plotted in OriginLab.

Kinetic experiments were carried out by preparing the required solutions of 100 mM **1a**, 100 mM **2a**, 10 mM BCF in $CDCl_3$, at 45 °C and using DCE as an internal standard in a Young's NMR tube inside a glove box. Data was analysed using the Reaction Monitoring tool in MestreNova (version 16) and integrated automatically over the time course of an experiment. The NMR spectra were automatically integrated and time points extracted using the Reaction Monitoring tool in MestReNova. Integrals for the signal 6.6-6.7 ppm for product **3aa** were selected as these are clearly baseline-separated from other signals. Integrals were exported and referencing against DCE was carried out in Excel. Resulting concentrations of **3aa** were plotted as a function of time using OriginLab Origin.

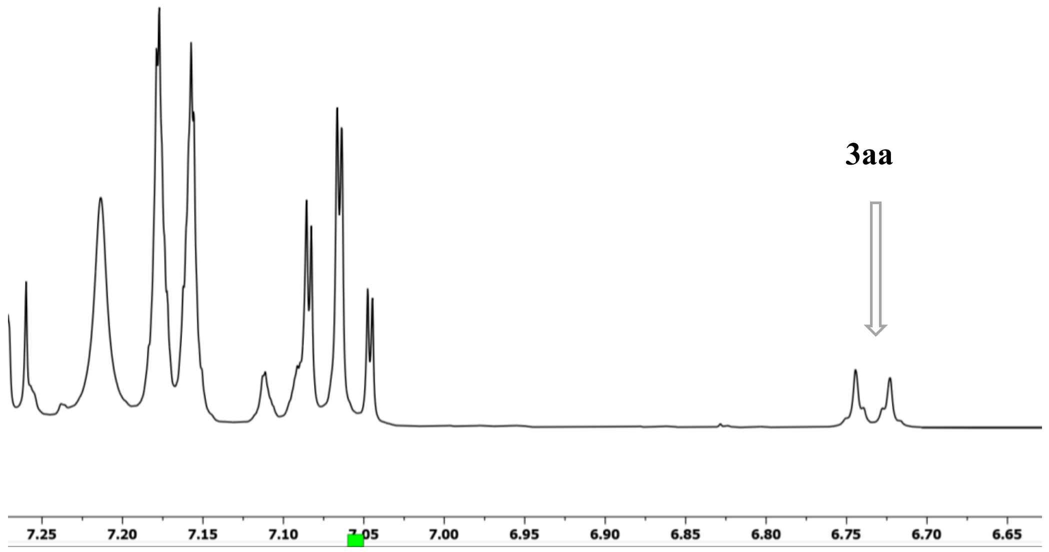

10.2 Titrations confirming the interaction between 2a and $B(C_6F_5)_3$.

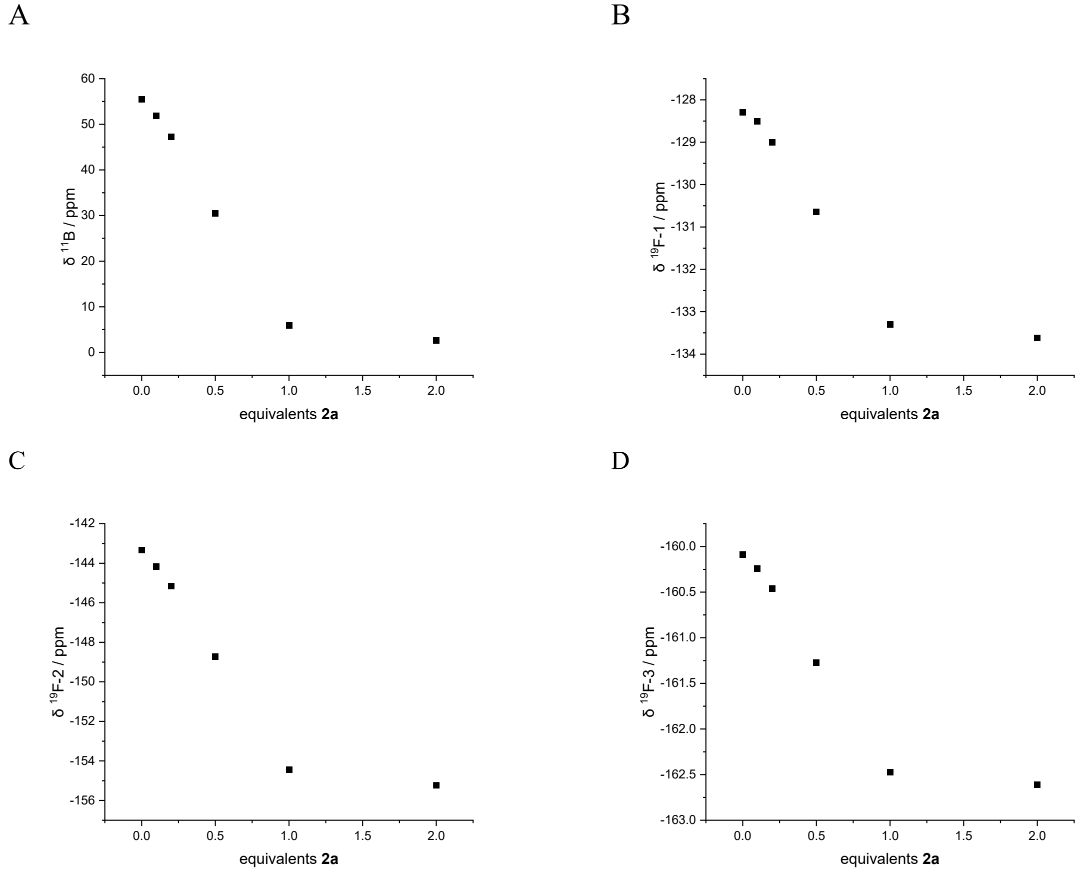

Figure S144: ^{11}B and ^{19}F NMR chemical shifts for a 58 mM solution of $B(C_6F_5)_3$ in the presence of different concentrations of **2a** in $CDCl_3$ at 45 °C.

10.3 Initial rate kinetics under reaction conditions.

The first set of kinetic experiments was carried out under the optimised reaction conditions (Figure S145).

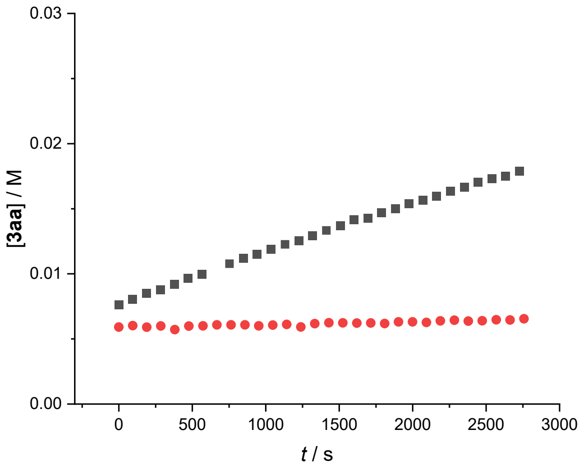

Figure S145: Concentration of **3aa** as a function of time in experiments involving 100 mM **1a**, 100 mM **2a**, 10 mM BCF (reference experiment under optimised reaction conditions, ●), 100 mM **1a**, 100 mM **2a**, 10 mM BCF, 100 mM succinimide (■); both in $CDCl_3$, at 45 °C and using DCE as internal standard. The estimated monitoring dead time is indicated as the extension of the time axis before *t*=0 s.

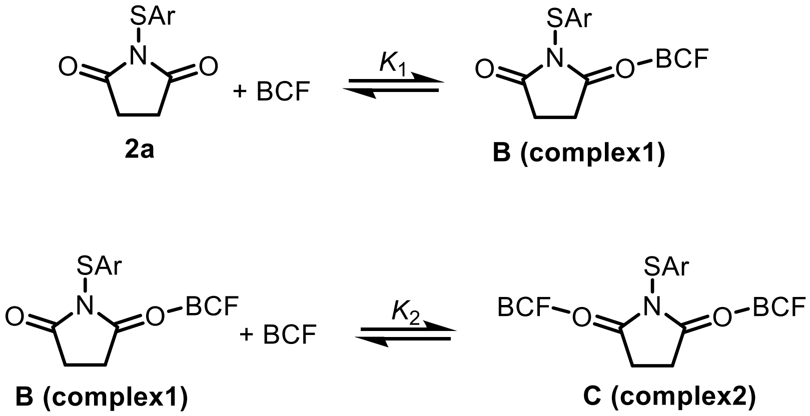

Scheme S21. The relevant equilibria where C formed a pre-r.d.s. equilibrium.

The equilibrium is then described by equations 1 and 2.

$$[\text{complex1}] = K_1 \times [2\text{a}]_{\text{free}} \times [\text{BCF}]_{\text{free}} \quad \text{Eq. 1}$$

$$[\text{complex2}] = K_1 \times K_2 \times [2\text{a}]_{\text{free}} \times [\text{BCF}]^2_{\text{free}} \quad \text{Eq. 2}$$

The mass balance equations are given by equations 3 and 4:

$$[2\text{a}]_{\text{total}} = [2\text{a}]_{\text{free}} + [\text{complex1}] + [\text{complex2}] \quad \text{Eq. 3}$$

$$[\text{BCF}]_{\text{total}} = [\text{BCF}]_{\text{free}} + [\text{complex1}] + 2 \times [\text{complex2}] \quad \text{Eq. 4}$$

Substituting Equations 1 and 2 into Equation 3 yields equation 5

$$[2a]_{\text{total}} = [2a]_{\text{free}} + K_1 \times [2a]_{\text{free}} \times [\text{BCF}]_{\text{free}} + K_1 \times K_2 \times [2a]_{\text{free}} \times [\text{BCF}]_{\text{free}}^2 \qquad \text{Eq. 5}$$

Which rearranges to Equation 6,

$$[2a]_{\text{total}} = \{1 + K_1 \times [\text{BCF}]_{\text{free}} + K_1 \times K_2 \times [\text{BCF}]_{\text{free}}^2\} \times [2a]_{\text{free}} \qquad \text{Eq. 6}$$

Giving Equation 7 for [**2a**]$_{\text{free}}$.

$$[2a]_{\text{free}} = \frac{[2a]_{\text{total}}}{\{1 + K_1 \times [\text{BCF}]_{\text{free}} + K_1 \times K_2 \times [\text{BCF}]_{\text{free}}^2\}} \qquad \text{Eq. 7}$$

Substituting Equations 1 and 2 into Equation 4 yields equation 8.

$$[\text{BCF}]_{\text{total}} = [\text{BCF}]_{\text{free}} + K_1 \times [2a]_{\text{free}} \times [\text{BCF}]_{\text{free}} + 2 \times K_1 \times K_2 \times [2a]_{\text{free}} \times [\text{BCF}]_{\text{free}}^2 \qquad \text{Eq. 8}$$

Inserting Eqn. 7 into Eqn. 8, followed by multiplication of both sides of the equation by the divisor of Eqn. 7 gives Eqn. 9.

$$\begin{aligned}&[\text{BCF}]_{\text{total}} \times \{1 + K_1 \times [\text{BCF}]_{\text{free}} + K_1 \times K_2 \times [\text{BCF}]_{\text{free}}^2\} = \\ &[\text{BCF}]_{\text{free}} \times \{1 + K_1 \times [\text{BCF}]_{\text{free}} + K_1 \times K_2 \times [\text{BCF}]_{\text{free}}^2\} + K_1 \times [2a]_{\text{total}} \times [\text{BCF}]_{\text{free}} \\ &+ 2 \times K_1 \times K_2 \times [2a]_{\text{total}} \times [\text{BCF}]_{\text{free}}^2\end{aligned} \qquad \text{Eq. 9}$$

Writing out the multiplications gives Eqn. 10.

$$\begin{aligned}&[\text{BCF}]_{\text{total}} + K_1 \times [\text{BCF}]_{\text{total}} \times [\text{BCF}]_{\text{free}} + K_1 \times K_2 \times [\text{BCF}]_{\text{total}} \times [\text{BCF}]_{\text{free}}^2 = \\ &[\text{BCF}]_{\text{free}} + K_1 \times [\text{BCF}]_{\text{free}}^2 + K_1 \times K_2 \times [\text{BCF}]_{\text{free}}^3 + K_1 \times [2a]_{\text{total}} \times [\text{BCF}]_{\text{free}} \\ &+ 2 \times K_1 \times K_2 \times [2a]_{\text{total}} \times [\text{BCF}]_{\text{free}}^2\end{aligned} \qquad \text{Eq. 10}$$

Equation 10 rearranges to the standard cubic form Equation 11.

$$\begin{aligned}&0 = K_1 \times K_2 \times [\text{BCF}]_{\text{free}}^3 + 2 \times K_1 \times K_2 \times [2a]_{\text{total}} \times [\text{BCF}]_{\text{free}}^2 + K_1 \times [\text{BCF}]_{\text{free}}^2 - \\ &K_1 \times K_2 \times [\text{BCF}]_{\text{total}} \times [\text{BCF}]_{\text{free}}^2 + \\ &[\text{BCF}]_{\text{free}} + K_1 \times [2a]_{\text{total}} \times [\text{BCF}]_{\text{free}} - K_1 \times [\text{BCF}]_{\text{total}} \times [\text{BCF}]_{\text{free}} - [\text{BCF}]_{\text{total}}\end{aligned} \qquad \text{Eq. 11}$$

Which can be rewritten as Equation 12 by collecting similar power terms.

$$\begin{aligned}&0 = K_1 \times K_2 \times [\text{BCF}]_{\text{free}}^3 + \{2 \times K_1 \times K_2 \times [2a]_{\text{total}} + K_1 - K_1 \times K_2 \times [\text{BCF}]_{\text{total}}\} \times [\text{BCF}]_{\text{free}}^2 \\ &+ \{1 + K_1 \times [2a]_{\text{total}} - K_1 \times [\text{BCF}]_{\text{total}}\} \times [\text{BCF}]_{\text{free}} - [\text{BCF}]_{\text{total}}\end{aligned} \qquad \text{Eq. 12}$$

Equation 12 is solved numerically to find [BCF]$_{\text{free}}$ using custom-written Python code, which also calculates [**2a**]$_{\text{free}}$ (Eq. 7), [complex1] (Eq. 1) and [complex2] (Eq. 2). The Python code is available at https://github.com/niekbuurmah2o/equilibria-in-double-BCF-catalysis.

Calculated concentrations for selected combinations of total concentrations of **2a** and BCF are presented in Table S4.

Table S4. Calculated concentrations of free 2a and BCF as well as complex1 and complex2.

2a_total	BCF_total	2a_free	BCF_free	complex1	complex2
0.0001	0.005	2E-06	0.004900622	9.66E-05	1.3733E-06
0.0002	0.005	4.07E-06	0.004801383	0.000193	2.69062E-06
0.0005	0.005	1.08E-05	0.004504542	0.000483	6.30746E-06
0.001	0.005	2.43E-05	0.0040131	0.000964	1.12243E-05
0.002	0.005	6.38E-05	0.0030468	0.001919	1.69583E-05
0.0025	0.005	9.38E-05	0.002575999	0.002388	1.78417E-05
0.003	0.005	0.000136	0.002118508	0.002847	1.74881E-05
0.005	0.005	0.000666	0.000657587	0.004326	8.24953E-06
0.01	0.005	0.005099	9.72704E-05	0.0049	1.3822E-06
0.02	0.005	0.015034	3.34305E-05	0.004966	4.81408E-07
0.05	0.005	0.045011	1.12173E-05	0.004988	1.62275E-07
0.1	0.005	0.095005	5.32095E-06	0.004995	7.70693E-08
0.2	0.005	0.195003	2.59383E-06	0.004997	3.75904E-08
0.5	0.005	0.495001	1.02215E-06	0.004999	1.48181E-08
0.05	0.01	0.040026	2.52198E-05	0.009973	7.29422E-07
